# Comparative Genomics Provides Insights into the Evolutionary Origin and Structural Diversification of Steroid Receptor Coactivators (SRCs 1–3)

**DOI:** 10.3390/molecules31152698

**Published:** 2026-08-03

**Authors:** Phelelani Erick Ngcobo, Kwanele Zulu, Nondumiso Silindokuhle Mabuyakhulu, Noxolo Princess Nkosi, Suresh Babu Pakala, Khajamohiddin Syed

**Affiliations:** 1 Department of Biochemistry and Microbiology, Faculty of Science, Agriculture and Engineering, University of Zululand, Empangeni 3886, South Africa; erickphelelani5@gmail.com (P.E.N.); kwanelezulu94@gmail.com (K.Z.); nonduh.ndiyema@gmail.com (N.S.M.); princessmlotshwa41@gmail.com (N.P.N.); pakalasb@uohyd.ac.in (S.B.P.); 2 Department of Biochemistry, School of Life Sciences, University of Hyderabad, Hyderabad 500046, India; 3 National Institute for Theoretical and Computational Sciences (NITheCS), Stellenbosch 7602, South Africa

**Keywords:** steroid receptor coactivators (SRCs), gene duplication and diversification, phylogenetic analysis, signature sequences, nuclear receptor coactivators

## Abstract

Steroid Receptor Coactivators (SRCs) are members of the p160 nuclear receptor coactivator family and play essential roles in regulating transcription, development, metabolism, reproduction, and disease. However, their evolutionary origin and diversification remain poorly understood. Here, we employed a comprehensive comparative genomics approach to investigate the evolution of SRC-1, SRC-2, and SRC-3 across the domains of life. Evaluation of domain-based screening approaches showed that the NCBI Batch Web CD-Search Tool clearly distinguished domains among the three SRC family members. Genome-wide analyses identified 298 canonical SRC proteins in vertebrates, revealing distinct taxonomic distributions among the three paralogs. Comparative analyses of LXXLL motifs suggested both conserved and paralog-specific patterns associated with functional diversification. Analysis of more than 20,000 proteins, including over 4000 SRC-associated domain-containing proteins, revealed a widespread distribution of SRC-associated domains across diverse taxa. These findings support the hypothesis that pre-existing protein modules distributed across diverse taxa may have contributed to the assembly of canonical vertebrate SRC proteins through progressive domain acquisition and recombination. Phylogenetic analyses showed that SRC-1, SRC-2, and SRC-3 form distinct monophyletic clades, consistent with diversification through ancient gene duplication. Overall, these findings provide a comparative genomic framework for investigating the origin, structural evolution, and functional diversification of vertebrate SRC proteins while generating testable hypotheses regarding their evolutionary history.

## 1. Introduction

Nuclear receptors comprise one of the largest and most extensively studied families of ligand-activated transcription factors in animals. These receptors regulate diverse physiological processes, including development, reproduction, metabolism, immune responses, cellular differentiation, and maintenance of homeostasis [1]. The transcriptional activity of nuclear receptors is controlled through interactions with a complex network of coregulatory proteins that either enhance or repress gene expression [2,3]. Among these regulatory molecules, Steroid Receptor Coactivators (SRCs), also known as the p160 coactivator family, represent one of the most important groups of transcriptional coactivators involved in nuclear receptor signaling pathways [3,4].

The SRC family was first identified with the discovery of SRC-1, a protein capable of enhancing steroid hormone receptor-mediated transcriptional activation [4]. Subsequent studies led to the identification of two additional paralogs, SRC-2 and SRC-3, collectively forming the p160 steroid receptor coactivator family [5]. These proteins are encoded by the nuclear receptor coactivator genes *NCOA1*, *NCOA2*, and *NCOA3*, respectively, and function as transcriptional integrators that bridge ligand-activated nuclear receptors with the basal transcription machinery and chromatin-remodeling complexes [3,6]. Through these interactions, SRC proteins facilitate transcriptional activation of target genes and contribute to the regulation of numerous physiological processes.

SRC proteins are characterized by a conserved modular architecture that underlies their biological functions. The N-terminal region contains a basic helix–loop–helix (bHLH) domain and Per–Arnt–Sim (PAS) domains that mediate protein–protein interactions, cellular localization, and transcriptional regulation. The central receptor interaction domain contains multiple LXXLL motifs, commonly referred to as nuclear receptor boxes, which directly interact with ligand-bound nuclear receptors [7]. The C-terminal region contains activation domains that recruit additional coactivators, histone acetyltransferases, methyltransferases, and chromatin-remodeling complexes required for efficient transcriptional activation [3,5]. This modular organization enables SRC proteins to function as molecular scaffolds that coordinate multiple signaling pathways and transcriptional networks.

Although members of the SRC family share a common structural framework, extensive evidence indicates that they possess both overlapping and distinct biological functions. SRC-1 plays critical roles in reproductive physiology, neuroendocrine signaling, and hormone-responsive gene regulation [8,9]. Studies using knockout mouse models have demonstrated that SRC-1 contributes to fertility, mammary gland development, and behavioral responses mediated by steroid hormones [5,8]. SRC-2, also known as transcriptional intermediary factor 2 (TIF2) or glucocorticoid receptor-interacting protein 1 (GRIP1), is involved in metabolic regulation, energy homeostasis, glucose metabolism, and reproductive function [5,10]. In contrast, SRC-3, also known as amplified in breast cancer 1 (AIB1) or activator of thyroid and retinoid receptors (ACTR), has been strongly associated with cellular proliferation, developmental regulation, and oncogenic signaling pathways [11,12].

The physiological significance of SRC proteins extends far beyond their classical role as nuclear receptor coactivators. These proteins interact with numerous transcription factors and signaling molecules, including Nuclear Factor Kappa B (NF-κB), Activator Protein 1 (AP-1), Signal Transducer and Activator of Transcription (STAT proteins), Early Region 2 Binding Factor (E2F) transcription factors, and other regulators of cellular growth and differentiation [6,13,14]. Consequently, SRC proteins influence a broad spectrum of biological processes, including embryonic development, tissue differentiation, immune regulation, inflammatory responses, and metabolic adaptation [13,14,15]. Their ability to integrate signals from multiple pathways has established SRC proteins as central regulators of cellular physiology [3].

Given their widespread regulatory functions, dysregulation of SRC proteins has been implicated in numerous human diseases. Elevated expression or aberrant activation of SRC family members has been reported in breast cancer, prostate cancer, ovarian cancer, endometrial cancer, and several other malignancies [13,14,16]. SRC-3 has emerged as a prominent oncogenic coactivator whose amplification and overexpression contribute to tumor progression, metastasis, and therapeutic resistance [11,16]. Similarly, alterations in SRC-1 and SRC-2 have been associated with endocrine disorders, reproductive abnormalities, obesity, metabolic syndrome, and inflammatory diseases [9,13,17]. These observations have stimulated considerable interest in SRC proteins as potential therapeutic targets and biomarkers for disease diagnosis and prognosis.

Despite considerable advances in understanding the evolution of vertebrate steroid receptor families [18] and the molecular functions of SRC proteins [5,14], the evolutionary origin, structural diversification, and conservation of the SRC family across vertebrates remain incompletely understood. Most previous studies have focused on the molecular mechanisms, physiological functions, and pathological significance of SRC family members [5,6,19], whereas comparatively little attention has been devoted to their evolutionary history, structural diversification, and genome-wide conservation across vertebrate lineages. Previous evolutionary investigations have largely examined steroid receptor signaling pathways and the functional specialization of nuclear receptor coactivators following gene duplication events [5,8,18], rather than the evolutionary diversification of the SRC family itself.

Comparative genomics and phylogenetic analyses provide powerful approaches for reconstructing the evolutionary history of protein families by examining sequence conservation, domain architecture, conserved motifs, and phylogenetic relationships across diverse taxa [19]. Such analyses have successfully elucidated the origin and diversification of several vertebrate regulatory protein families, including steroid receptors [18]. Applying these approaches to the SRC family can provide valuable insights into the evolutionary mechanisms underlying the emergence of complex transcriptional regulatory systems and identify conserved structural domains and functional motifs associated with SRC-mediated transcriptional regulation.

However, comprehensive genome-wide studies integrating SRC protein identification, comparative domain architecture, conserved receptor-interaction motifs, amino acid conservation, structural analyses, and phylogenetic reconstruction across diverse vertebrate lineages remain limited. Furthermore, the evolutionary origin of the modular SRC protein architecture and the potential contribution of ancient protein domains to the emergence of modern SRC proteins have not been systematically investigated.

To address these knowledge gaps, the present study integrates genome-wide SRC identification, comparative domain architecture, LXXLL motif characterization, amino acid conservation, structural analyses, and phylogenetic reconstruction to investigate the origin, structural diversification, and evolutionary relationships of vertebrate SRC family proteins, thereby providing a comparative genomic framework for investigating the evolutionary origin, structural diversification, and functional evolution of vertebrate SRC proteins.

## 2. Results and Discussion

### 2.1. Evaluation of Domain-Screening Tools for SRC Protein Identification

The structural characteristics, conserved domains, and functional features of SRCs are well defined in the literature [9,17,20]. These proteins are characterized by distinct domain architectures that enable their identification across diverse species. However, the large-scale identification of SRC proteins requires a domain-screening approach capable of distinguishing among the three closely related SRC paralogs. Therefore, before conducting genome-wide analyses, we compared three commonly used domain annotation tools to determine which most effectively differentiated the three well-characterized human SRC reference proteins used in this study. This comparison was intended to guide tool selection for the present analysis rather than to establish the overall performance of these programs for all protein families.

**Figure 1:**
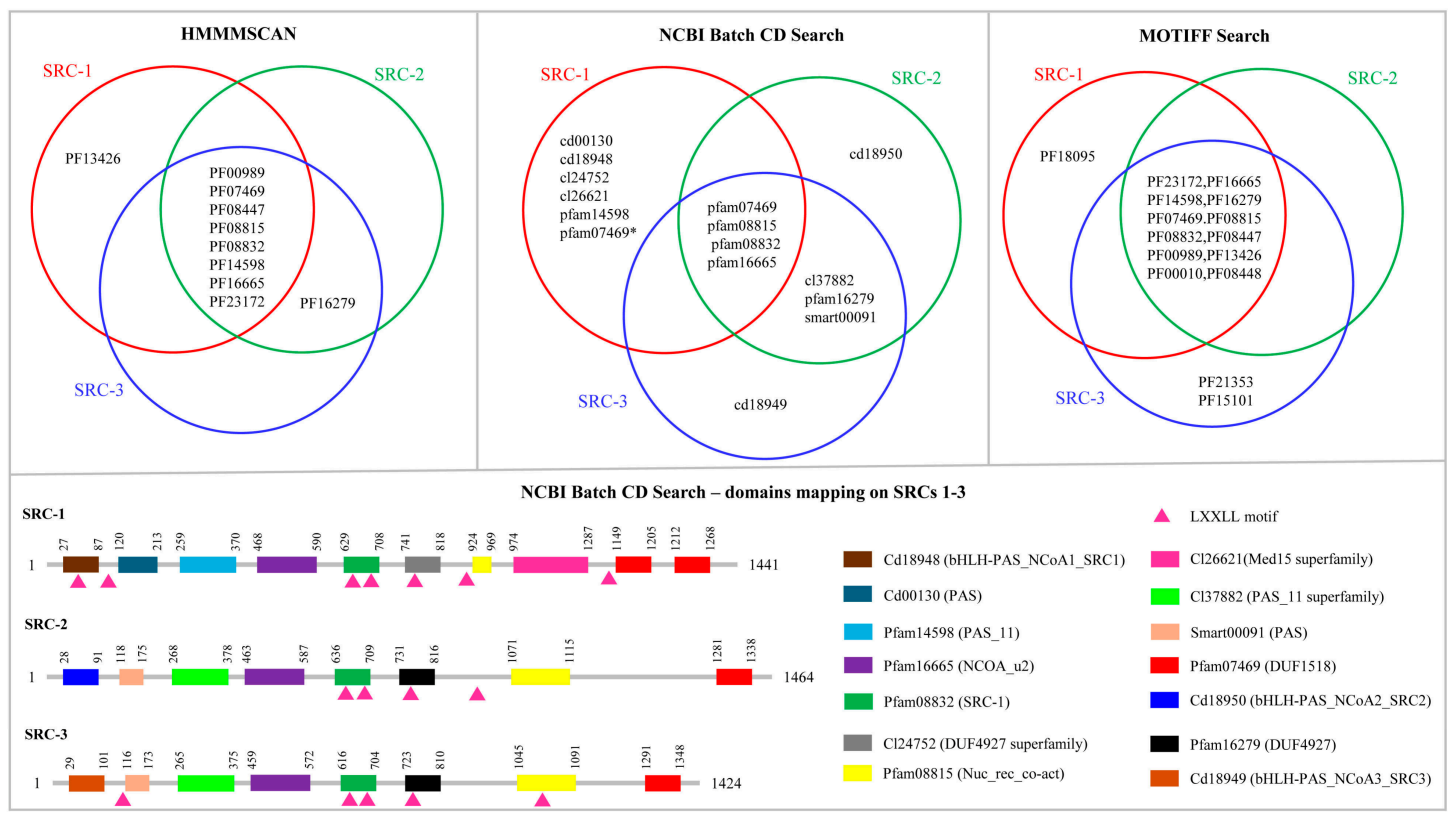
Comparative analysis of domain architecture in steroid receptor coactivators 1–3 (SRCs 1–3) using multiple domain prediction tools. Domain composition and organization of SRC proteins were analyzed using the MOTIF Search tool (https://www.genome.jp/tools/motif/, accessed on 23 March 2026), the Hidden Markov Model Scan (HMMSCAN) [21,22] implemented via the HMMER website [23], and the National Center for Biotechnology Information (NCBI) Batch Web CD-Search Tool [24]. The domain annotations shown correspond to those identified by the NCBI Batch CD-Search Tool, with conserved domain accession numbers provided alongside their common identifier names (in parentheses). Human SRCs 1–3 proteins (UniProt IDs: Q15788, Q15596, and Q9Y6Q9, respectively) were used as reference sequences to define domain boundaries. Domain start and end positions are indicated based on amino acid residue numbering. An asterisk symbol indicates the presence of an additional pfam07469 domain in SRC-1 compared to SRCs 2 and 3.

To address this, human SRC-1, SRC-2, and SRC-3 proteins (UniProt IDs: SRC-1: Q15788; SRC-2: Q15596; SRC-3: Q9Y6Q9) were used as reference sequences to evaluate the performance of different domain-based screening tools. Three widely used computational programs were assessed: the MOTIF Search tool (https://www.genome.jp/tools/motif/, accessed on 23 March 2026), the Hidden Markov Model Scan (HMMSCAN) [21,22] implemented via the HMMER website [23], and the National Center for Biotechnology Information (NCBI) Batch Web CD-Search Tool [24] (Figure 1).

Comparative analysis of the three domain annotation tools showed that, among the reference SRC-1, SRC-2, and SRC-3 proteins examined in this study, the NCBI Batch CD-Search Tool provided the clearest differentiation of the characteristic domain architectures associated with each paralog (Figure 1). Therefore, this tool was selected for subsequent genome-wide SRC identification and classification. This comparison was performed solely to select the most suitable tool for the present study and should not be interpreted as a comprehensive benchmark of domain-screening software. According to the domain annotations generated by the NCBI Batch CD-Search Tool [24], all three SRC proteins share a common core of four conserved domains, reflecting their evolutionary relatedness. In addition, SRC-2 and SRC-3 share three additional domains, further highlighting their closer relationship with each other compared with SRC-1.

SRC-1 exhibited the most complex domain architecture among the proteins analyzed, containing five unique domains and an additional pfam07469 domain, which may contribute to its broader functional versatility (Figure 1). In contrast, SRC-2 and SRC-3 each possess one unique domain, consistent with their more specialized and lineage-restricted roles. Notably, the reliability and applicability of the NCBI Batch CD-Search Tool [24] for large-scale transcription factor family analysis have been demonstrated in recent studies, including its successful use in the identification and classification of NF-κB transcription factor family members [25].

### 2.2. Distribution and Taxonomic Representation of SRC Family Proteins Across Vertebrates

**Figure 2:**
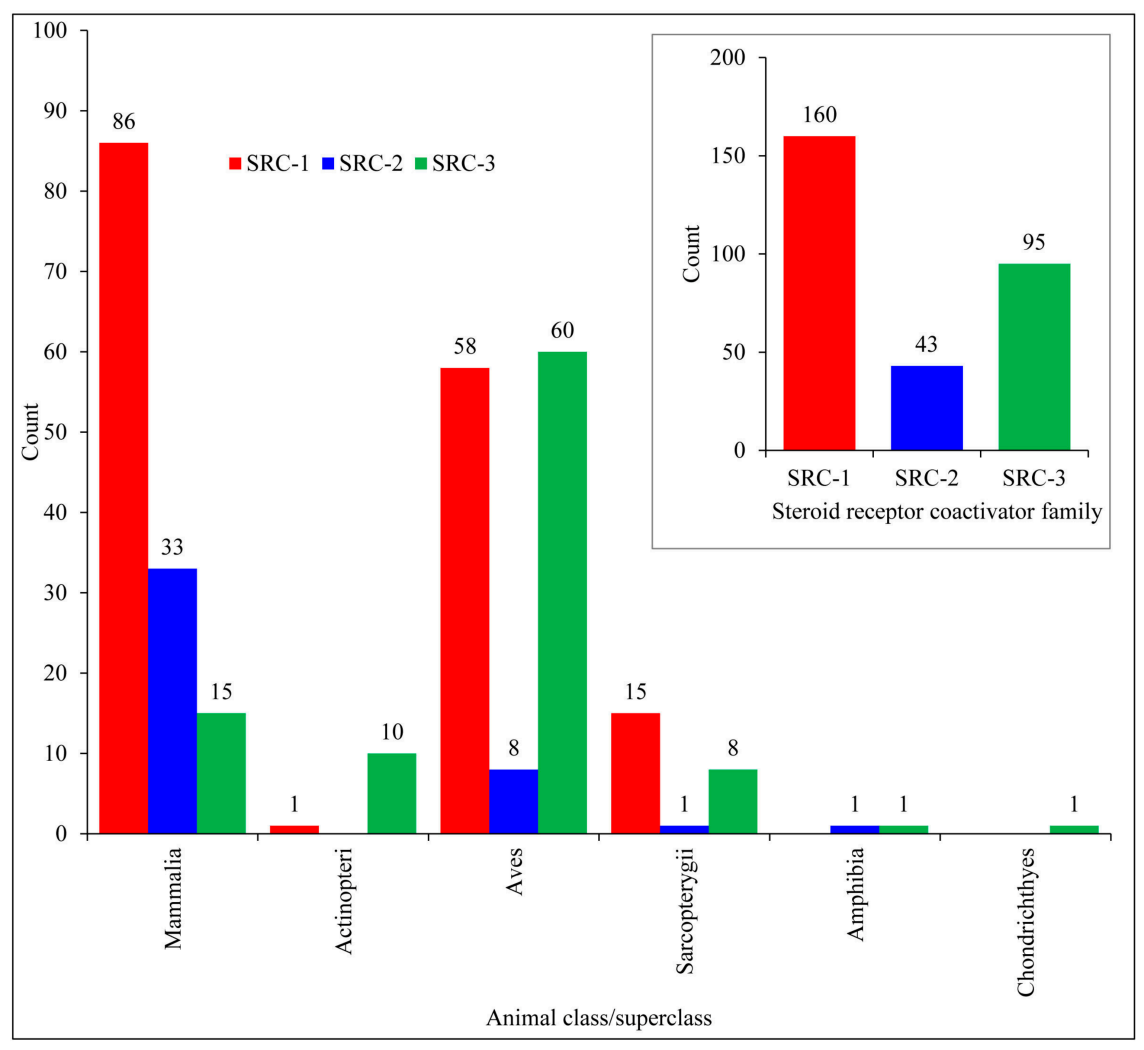
Genome-wide identification, classification, and distribution of steroid receptor coactivators 1–3 (SRCs 1–3) across Chordata. A comprehensive genome-mining approach was employed to identify SRC proteins, which were then classified into the SRC families 1–3. The inset displays the total number of SRC proteins identified for each family. Chordate species were classified according to the National Center for Biotechnology Information (NCBI) taxonomy [26]. Detailed information on individual SRC members 1–3 is provided in Tables S1–S3. Numbers next to each bar represent the total number of SRC proteins identified within the corresponding taxonomic category.

Genome-wide analysis of canonical SRC family proteins (SRC-1, SRC-2, and SRC-3) retrieved from the UniProt database revealed distinct distribution patterns among currently available vertebrate protein records (Figure 2 and Tables S1–S3).

A total of 298 SRC proteins were identified, comprising 160 SRC-1 proteins, 43 SRC-2 proteins, and 95 SRC-3 proteins, indicating that SRC-1 represented the largest number of retrieved protein records, followed by SRC-3, whereas SRC-2 represents the least abundant paralog (Figure 2 and Tables S1–S3). The distribution analysis further suggested that all three SRC paralogs are associated with vertebrate lineages, particularly Mammalia and Aves. However, SRC-3 exhibited the broadest taxonomic spread, extending into Actinopteri, Amphibia, Chondrichthyes, and Sarcopterygii (Figure 2 and Tables S1–S3). The broader taxonomic representation of SRC-3 compared with SRC-1 and SRC-2 highlights differences in the distribution patterns of SRC family members across vertebrate lineages, indicating considerable variation in their taxonomic representation.

The observed distribution patterns are consistent with the known biological functions of SRC family members. SRC-1 was the most abundant paralog identified in this study and was particularly well represented in mammalian species. Previous studies have demonstrated that SRC-1 participates in steroid hormone signaling, reproductive physiology, and neuroendocrine regulation, functions that are especially prominent in vertebrates with complex endocrine systems [4,8]. The widespread occurrence of SRC-1 across mammalian taxa therefore reflects its important role in regulating hormone-responsive transcriptional networks.

SRC-2 exhibited the lowest abundance among the three paralogs and was detected in a more restricted range of vertebrate taxa. Functional studies have shown that SRC-2 is involved in metabolic regulation, energy homeostasis, and reproductive physiology [27,28]. The relatively limited distribution of SRC-2 observed in this study may reflect greater functional specialization than that of other members of the SRC family.

In contrast, SRC-3 was identified across a broader range of vertebrate groups, including fishes, amphibians, birds, reptiles, and mammals. SRC-3 has been implicated in developmental regulation, cellular growth, and transcriptional activation of numerous signaling pathways [16,29]. Its extensive distribution suggests that SRC-3 participates in fundamental biological processes that are widely conserved across vertebrates.

### 2.3. Comparative Domain Architecture of SRC Family Proteins

**Figure 3:**
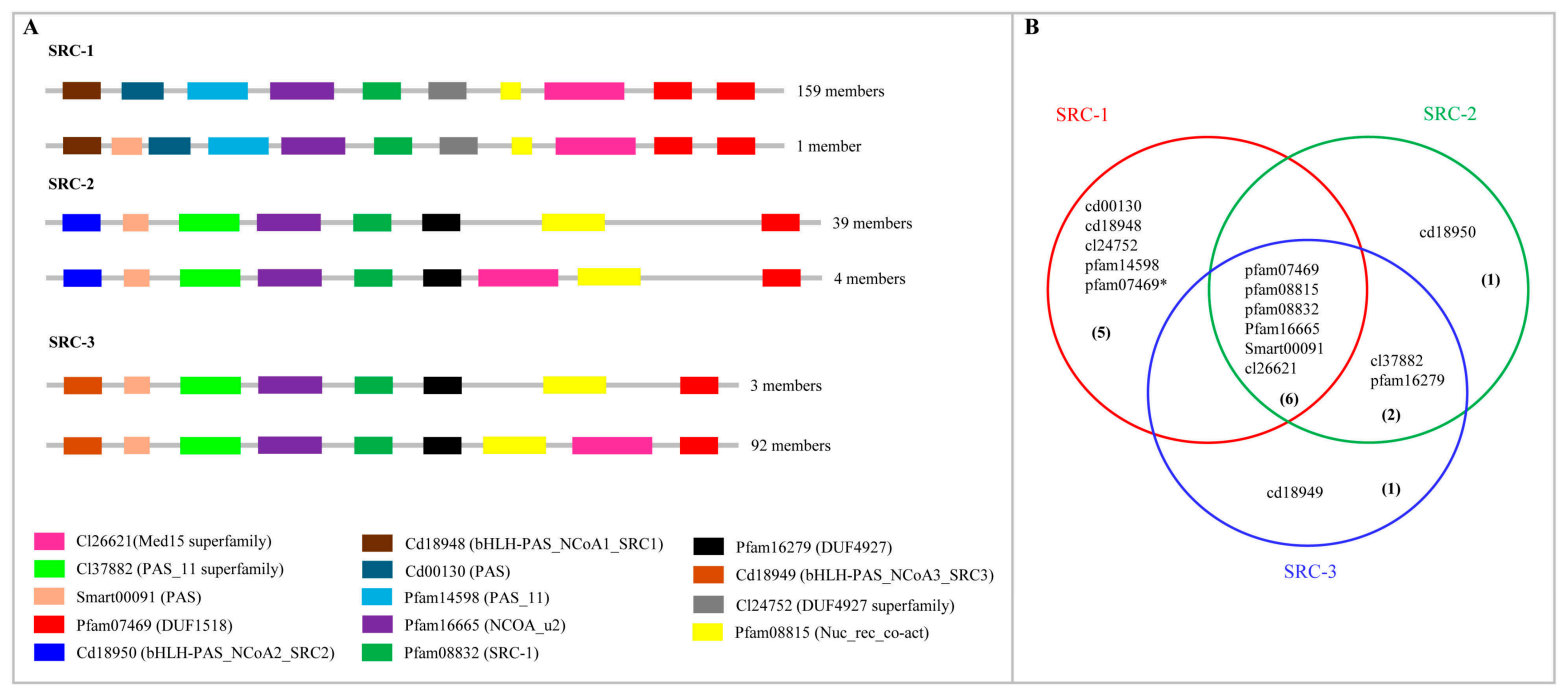
Protein domain analysis in steroid receptor coactivators 1–3 (SRCs 1–3). (**A**) Schematic diagram representing the domain organization in SRCs 1–3. The domain analysis was carried out using the National Center for Biotechnology Information (NCBI) Batch Web CD-Search Tool [24]. The domain annotations shown correspond to those identified by the NCBI Batch CD-Search Tool, with conserved domain accession numbers provided alongside their common identifier names (in parentheses). (**B**) Comparative domain analysis among SRCs 1–3. An asterisk symbol indicates the presence of an additional pfam07469 domain in SRC-1 compared to SRCs 2 and 3. The number in the parentheses indicates the number of domains. Detailed information on domain analysis among SRCs 1–3 is presented in Tables S1–S3.

Domain architecture analysis of SRC-1, SRC-2, and SRC-3 proteins suggested a high degree of structural conservation across the SRC family while also revealing notable paralog-specific differences in domain organization (Figure 3). All three SRC proteins retained the characteristic modular arrangement associated with the p160 nuclear receptor coactivator family, including conserved bHLH and PAS domains, central receptor interaction regions, and C-terminal transcriptional activation domains (Figure 3). The conservation of these domains across SRC paralogs suggests that the fundamental molecular functions of nuclear receptor recognition and transcriptional coactivation have been evolutionarily maintained. However, clear differences in the number, arrangement, and extension of specific domains were observed among the paralogs, suggesting functional divergence following ancestral gene duplication events (Figure 3). Differences in domain architecture compared with human SRC family members include the presence of Smart00091 (PAS) domains in one of the SRC-1 members, which are common to both SRC-2 and SRC-3 (Figure 3A). Four SRC-2 members have the CL26621 domain, which is characteristic of SRC-1 members (Figure 3A). Only 3 SRC-3 members have the domain architecture as human SRC-3 proteins, and the rest of the SRC-3 members (92) have the CL26621 domain, which is characteristic of SRC-1 members (Figure 3A). Overall, SRC-1 displayed the most structurally elaborate architecture, with multiple conserved domains distributed throughout the protein, whereas SRC-2 exhibited a comparatively reduced, compact domain organization. SRC-3 retained a moderately complex architecture that shared structural features with both SRC-1 and SRC-2, indicating a combination of conserved and lineage-specific domain characteristics within this paralog (Figure 3A). Overall, six domains were shared among all SRC family members, whereas two additional domains were common only to SRC-2 and SRC-3 (Figure 3B). SRC-1 contained four unique domains together with the additional pfam07469 domain (Figure 3B). These differences in domain composition highlight the structural diversification that has occurred within the SRC family while preserving a conserved core architecture required for coactivator function.

### 2.4. Evolutionary Conservation and Functional Variation of LXXLL Nuclear Receptor Interaction Motifs in SRC Family Proteins

**Figure 4:**
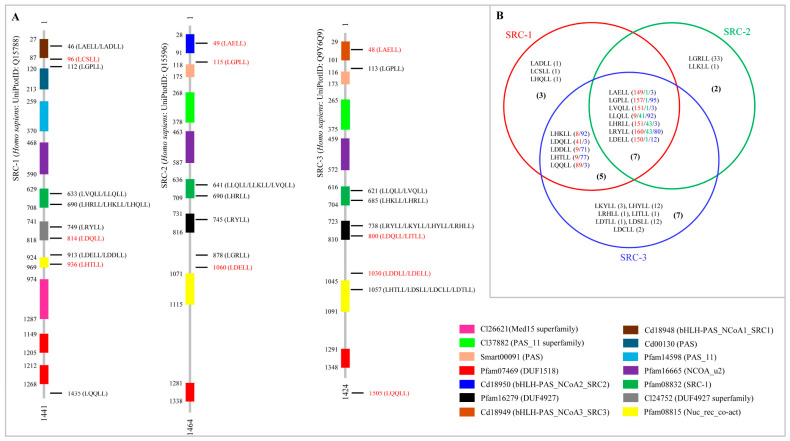
Steroid receptor coactivators 1–3 (SRCs 1–3) characteristics LXXLL motif amino acid pattern analysis. (**A**) Mapping of SRC characteristics LXXLL motifs on human SRC proteins. Domain start and end positions and the LXXLL motif position are indicated based on human SRCs’ amino acid residue numbering. The LXXLL motifs shown in black are present in human SRCs and the majority of other SRCs (dominantly present in SRCs), and the red-colored motifs are present in a few SRCs and are absent in human SRCs. One of the SRC-3 LXXLL motifs (LQQLL) is found in a protein longer than human SRC-3, and thus it is mapped using that protein’s amino acid numbering. The domain annotations shown correspond to those identified by the National Center for Biotechnology Information (NCBI) Batch Web CD-Search Tool [24], with conserved domain accession numbers are provided alongside their common identifier names (in parentheses). (**B**) Comparative analysis of LXXLL motifs among SRCs 1–3. The numbers next to LXXLL motifs indicate the number of proteins that have particular motifs, and different colors represent different SRCs (red, SRC-1; green, SRC-2; and blue, SRC-3). Numbers in bold and in parentheses indicate the number of motifs. Detailed information on LXXLL motif analysis among SRCs 1–3 is presented in Tables S4–S6.

Analysis of SRC proteins revealed that SRC-1, SRC-2, and SRC-3 possess multiple LXXLL motifs distributed throughout their sequences (Figure 4A and Tables S4–S6). The number of LXXLL motifs in SRC-1 proteins ranged from 3–8 (22 SRC-1 had 8 motifs, 84 SRC-1 had 7 motifs, 53 SRC-1 had 6 motifs and one SRC-1 had only 3 motifs); in SRC-2 proteins the motif number ranged from 3–6 (one SRC-3 had 6 motifs, 33 SRC-3 had 4 motifs and 9 SRC-3 had 3 motifs); and in SRC-3 proteins the motif number ranged from 5–8 (3 SRC-3 had 8 motifs; one SRC-3 had 7 motifs; 79 SRC-3 had 6 motifs and 12 SRC-3 had 5 motifs) (Figure 4A and Tables S4–S6). Analysis of motif amino acids revealed the presence of a specific pattern of amino acids in these motifs across all the SRCs, where in some motifs a single pattern was present, and in some motifs more than one pattern of amino acid sequences was observed (Figure 4A).

Seven motif sequence patterns at relevant positions are shared among all three SRC families (Figure 4B), suggesting the presence of highly conserved ancestral receptor-interaction sites that have been maintained throughout vertebrate evolution. Five motif sequence patterns were shared between SRC-1 and SRC-3 (Figure 4B). In contrast, additional motifs were found exclusively within individual SRC paralogs. SRC-1 contained 3 unique motif patterns, whereas SRC-2 and SRC-3 exhibited 3 and 7 distinct motif patterns (Figure 4B). The presence of both shared and lineage-specific LXXLL motif patterns suggests that, following duplication of an ancestral SRC gene, the paralogs retained a conserved core set of receptor-binding motifs while simultaneously acquiring novel interaction motifs through sequence diversification. Such diversification may have expanded the spectrum of nuclear receptors and transcriptional complexes that each SRC protein could engage, thereby promoting functional specialization. Similar evolutionary patterns have been reported in other nuclear receptor coactivator families, in which duplication and modification of receptor-interaction motifs have contributed to increased regulatory complexity and tissue-specific functions [3,30].

The distribution of motifs further suggests differential evolutionary pressures among the SRC paralogs. SRC-3 appears to possess the greatest motif complexity (19 LXXLL motif patterns), including both conserved and unique LXXLL motifs, which may explain its broad functional involvement in development, growth signaling, and oncogenic pathways. SRC-1 exhibits a combination of conserved motifs and several unique motif patterns (3), consistent with its prominent role in steroid hormone signaling and reproductive regulation. Conversely, SRC-2 appears to retain a more restricted repertoire of motifs, supporting previous observations that it is evolutionarily more conserved and functionally specialized for metabolic regulation and energy homeostasis. Collectively, these findings suggest that the evolution of SRC proteins involved both the preservation of ancestral nuclear receptor interaction motifs and the acquisition of paralog-specific LXXLL motifs, suggesting one possible molecular mechanism for the functional diversification of the vertebrate SRC/p160 coactivator family.

The observed diversity of LXXLL motif patterns suggests that SRC family members possess distinct capacities to interact with nuclear receptors and associated transcriptional complexes. The coexistence of highly conserved motifs and paralog-specific motif variants suggests that the fundamental mechanism of receptor recognition has been maintained throughout evolution while allowing flexibility in receptor specificity and regulatory interactions. Such diversification may contribute to differences in transcriptional activity, tissue specificity, and signaling pathway utilization among SRC paralogs. These findings further support the importance of LXXLL motifs as key structural determinants of SRC-mediated transcriptional regulation.

### 2.5. Structural Conservation and Sequence Variation of SRC Family Proteins

The domain architectures and LXXLL motif compositions described above provide insight into the functional organization of SRC proteins. To determine whether these conserved structural features are also reflected at the sequence level, amino acid conservation patterns among SRC family members were subsequently investigated using Profile Multiple Alignment with predicted Local Structures and 3D constraints (PROMALS3D) [31], which integrates sequence and structural information to provide a robust assessment of conservation patterns. The analysis revealed distinct differences in amino acid conservation among SRCs (Table 1). Analysis of all SRC proteins revealed 143 completely conserved amino acids (score 9) across three different family members. Comparative analysis of individual members revealed the highest amino acid conservation in SRC-2 (500 amino acids), followed by SRC-1 (306 amino acids) and SRC-3 (235 amino acids). These observations suggest that the conservation patterns reflect intrinsic evolutionary properties of each SRC family rather than differences in sequence representation. This high amino acid conservation suggests that SRC-2 has been subject to greater evolutionary constraints, indicating the preservation of essential structural and functional features throughout vertebrate evolution. In contrast, SRC-1 and SRC-3 contained fewer highly conserved amino acids than SRC-2, indicating comparatively greater sequence variability that may contribute to differences in structural flexibility and biological function. Overall, the findings suggest that SRC-2 is the most structurally conserved member of the SRC family, retaining ancestral sequence features important for its biological function.

**Figure 5:**
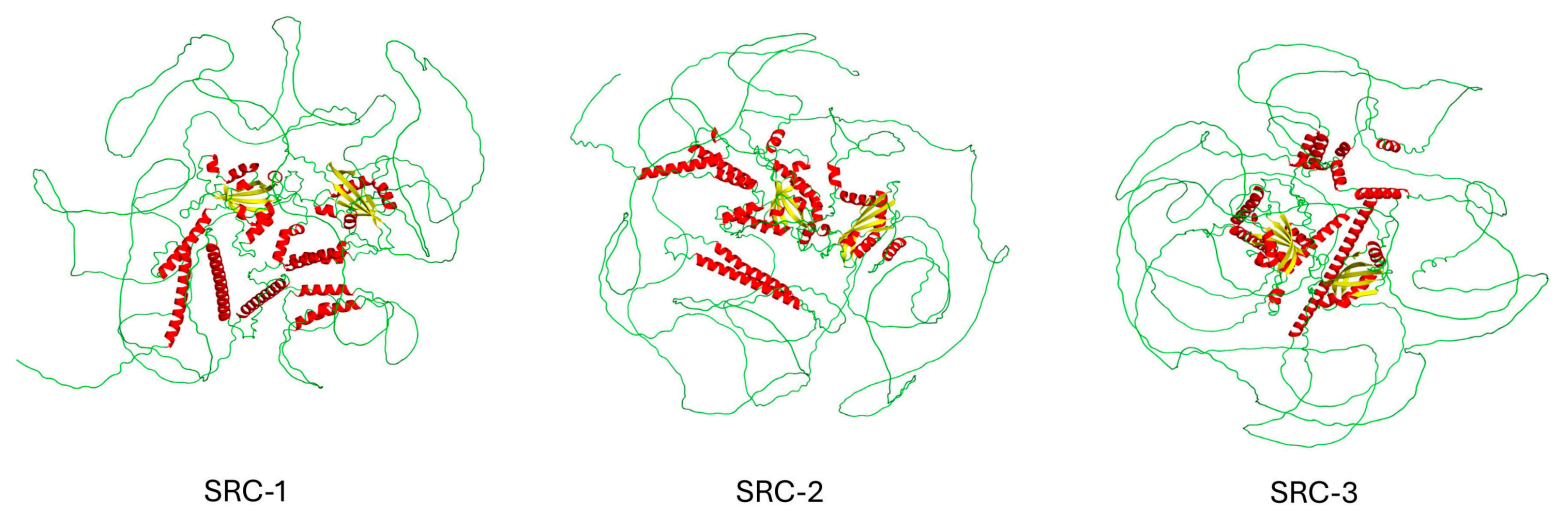
Structural analysis of Steroid receptor coactivators (SRCs 1–3) proteins from *Homo sapiens*. The UniProt protein IDs for SRCs: SRC-1 (Q15788), SRC-2 (Q15596), and SRC-3 (Q9Y6Q9). Alpha helices are shown in red, beta sheets are shown in yellow, and loops are shown in green. A multiple sequence alignment showing the respective secondary structural elements, SRC characteristic domains, and the conserved amino acids is presented in Figure S1.

To visualize the spatial distribution of conserved amino acid residues and secondary structural elements, predicted three-dimensional models of human SRC-1, SRC-2, and SRC-3 were obtained from the AlphaFold3 database [32] (Figure 5). Because SRC proteins are known to contain extensive intrinsically disordered regions, these models were used primarily to visualize local secondary-structure features rather than to interpret detailed full-length tertiary-structure information.

Structural analysis revealed that SRCs 1–3 proteins are predominantly composed of intrinsically disordered regions, characterized by abundant loops, with only limited regions forming defined secondary structural elements, such as α-helices and β-sheets (Figure 5). This structural organization is consistent with the known properties of transcriptional coactivators, which often exhibit structural flexibility to facilitate interactions with multiple binding partners. However, the predicted three-dimensional models exhibited relatively low overall confidence, with predicted template modeling (pTM) and interface predicted template modeling (ipTM) scores ranging from 0.23 to 0.28. These low confidence values are consistent with the intrinsically disordered nature of SRC proteins and therefore limit the reliability of the predicted full-length tertiary structures. Consequently, the structural interpretation presented in this study is limited to local secondary-structure elements and conserved domains. These limitations likely stem from the lack of experimentally resolved crystal structures of SRCs and are consistent with the intrinsically disordered nature of SRC proteins, which substantially reduces the confidence of full-length structural predictions despite recent advances in AlphaFold3 modelling [32]. Despite the relatively low confidence of the full-length structural models, AlphaFold predictions were considered sufficiently reliable for the limited purpose of visualizing local secondary structural features, including α-helices and β-sheets, within conserved regions of the proteins. Consequently, the structural interpretation presented in this study is restricted to these higher-confidence local structural features rather than the overall three-dimensional architecture.

To further investigate the distribution of these secondary structural elements across SRCs 1–3 members, a multiple sequence alignment (MSA) of these proteins was performed, and the secondary structural elements were mapped alongside conserved amino acids and domains (Figure S1). The MSA showed that several predicted α-helices and β-sheets were conserved among SRC family members and frequently coincided with conserved domains and highly conserved amino acid residues (Table 1 and Figure S1). Notably, all 306 highly conserved amino acids identified in SRC-1 were located within conserved domains, several of which corresponded to predicted α-helical and β-sheet regions (Table 1 and Figure S1). In contrast, only 50% of the conserved amino acids were found to be part of different domains in SRC-2 and SRC-3 (Table 1 and Figure S1).

Although AlphaFold3 [32] provides valuable structural predictions for proteins lacking experimentally determined structures, the extensive intrinsically disordered regions present in SRC proteins limit the confidence of full-length structural models. Therefore, the present analysis should be interpreted primarily as a visualization of conserved secondary-structure features and domain organization, rather than as definitive evidence of the complete tertiary structure. Future experimental structural studies will be required to validate these predicted conformations.

### 2.6. Phylogenetic Relationships and Evolutionary History of SRC Family Proteins

**Figure 6:**
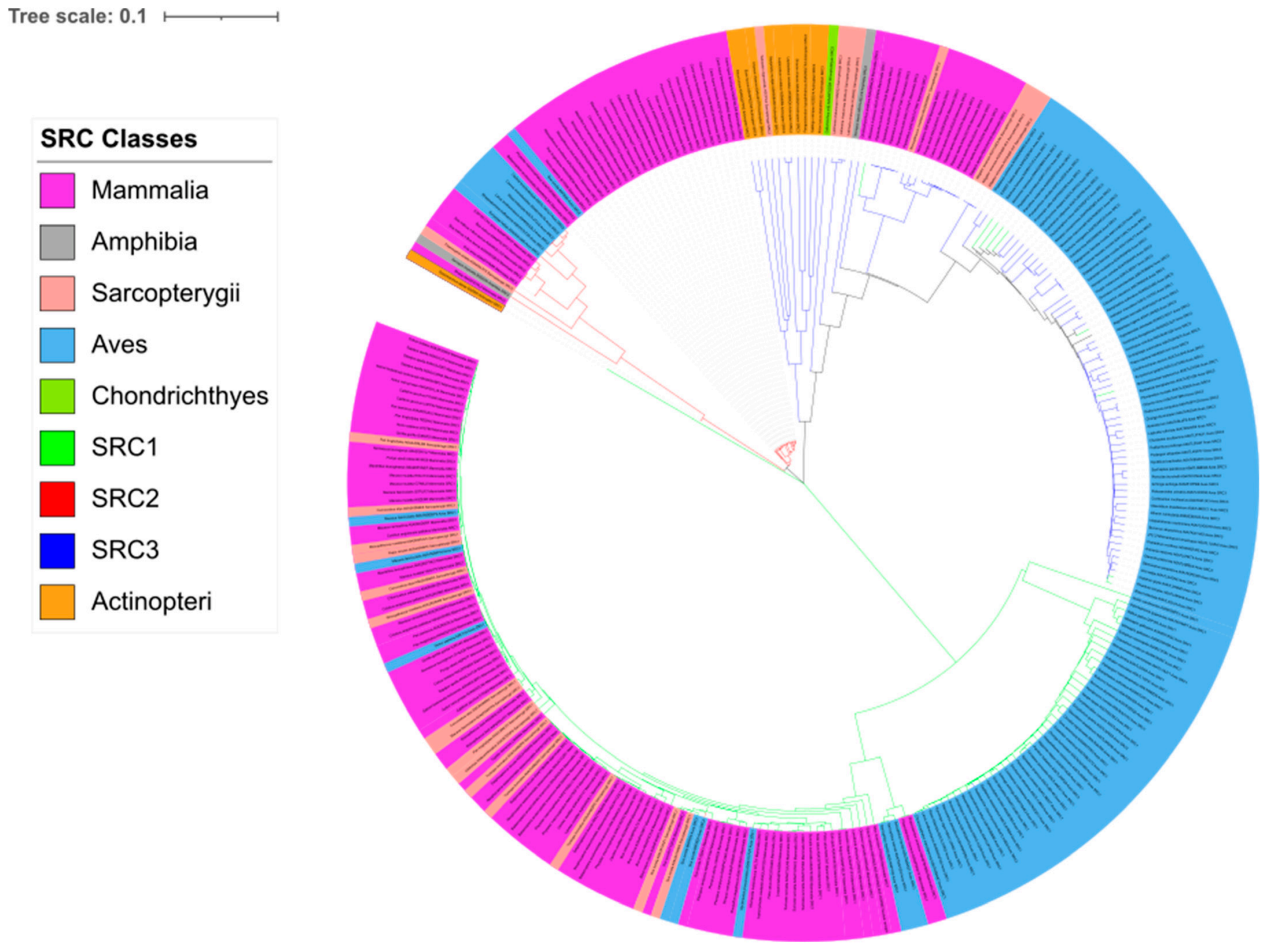
Phylogenetic analysis of steroid receptor coactivator family members (SRCs 1–3). The phylogenetic tree was constructed using 298 SRC protein sequences (SRCs 1–3). SRC family members are distinguished by branch color coding, while node colors represent different chordate classes/superclasses, as indicated in the figure. Taxonomic classification of chordate species was based on the National Center for Biotechnology Information (NCBI) taxonomy [26]. A high-resolution version of the phylogenetic tree, including detailed node annotations, is shown in Figure S2, and the protein sequences used to construct the tree and corresponding alignment file are presented in Supplementary Datasets S1 and S2.

Phylogenetic analysis of SRCs 1–3 revealed clear clustering of the three paralogous protein families, suggesting distinct evolutionary trajectories across vertebrate lineages (Figure 6). The phylogenetic tree showed that SRC-1, SRC-2, and SRC-3 form separate monophyletic clades, supporting the hypothesis that the modern SRC family arose through ancient gene duplication events early during vertebrate evolution. Among the three paralogs, SRC-3 displayed the broadest phylogenetic distribution, containing representatives from Chondrichthyes, Actinopteri, Amphibia, Aves, Sarcopterygii, and Mammalia, whereas SRC-1 and SRC-2 were comparatively enriched in mammalian taxa as described in Section 2.2.

The extensive distribution of SRC-3 across basal and derived vertebrate groups suggests that this coactivator has been broadly retained throughout vertebrate evolution and across diverse vertebrate lineages. SRC-3 sequences from cartilaginous fishes, ray-finned fishes, coelacanths, amphibians, birds, and mammals formed a large and deeply branching clade, suggesting ancient functional importance. This observation agrees with previous studies showing that members of the p160/SRC family originated before vertebrate diversification and subsequently evolved specialized physiological functions while maintaining conserved nuclear receptor interaction domains [3,5]. The retention of SRC-3 across highly divergent taxa suggests that this paralog performs fundamental regulatory functions that were required throughout vertebrate evolution. Furthermore, the broader phylogenetic distribution and deeper branching topology of SRC-3 suggests that this paralog has retained evolutionary features that have persisted across a wide range of vertebrate lineages.

SRC-1 exhibited strong diversification within Mammalia, particularly among primates, carnivores, cetaceans, bats, rodents, and marsupials. Although avian SRC-1 sequences were also present, mammalian sequences dominated this clade, suggesting extensive lineage-specific expansion and adaptation after the mammalian radiation. SRC-1 is known to function prominently in steroid hormone signaling, reproductive biology, and neuroendocrine regulation [4,8]. The high abundance and diversification of mammalian SRC-1 sequences observed in the tree may therefore reflect adaptive evolution associated with increasingly complex endocrine and developmental systems in mammals. In addition, the clustering of closely related mammalian taxa within SRC-1 suggests relatively recent diversification events superimposed on an ancient conserved framework.

SRC-2 formed a comparatively smaller and more compact clade dominated by mammals, with fewer representatives from birds, amphibians, and reptiles. This restricted phylogenetic spread suggests stronger evolutionary constraints on SRC-2 than on SRC-1 and SRC-3. Previous studies have demonstrated that SRC-2 plays major roles in energy metabolism, reproductive physiology, and systemic metabolic homeostasis [27,28]. Because metabolic regulation is highly conserved across vertebrates, the relatively compact topology of SRC-2 may reflect functional conservation and slower evolutionary divergence. The reduced taxonomic breadth of SRC-2 relative to SRC-3 is consistent with greater functional specialization following duplication of an ancestral SRC-like gene.

Collectively, the phylogenetic topology supports an evolutionary scenario in which an ancestral SRC-like coactivator existed before vertebrate diversification and subsequently gave rise to the modern SRC family through ancient gene duplication events. Following duplication, SRC-1 underwent substantial expansion and diversification, particularly within mammals, whereas SRC-2 and SRC-3 followed distinct evolutionary trajectories characterized by differences in taxonomic representation and lineage-specific divergence. Together, these findings suggest that gene duplication, divergence, and functional specialization were major evolutionary forces that shaped the modern SRC family and may have contributed to the increasing complexity of vertebrate nuclear receptor signaling networks.

Although phylogenetic analysis provides comparative evidence for the duplication and divergence of the modern SRC paralogs, it does not reveal how the ancestral SRC architecture originally arose. To address this question, a broader evolutionary investigation was conducted by examining the occurrence and combinations of SRC-associated domains across the domains of life. This approach enabled reconstruction of the potential evolutionary steps that preceded the emergence of the canonical vertebrate SRC proteins.

### 2.7. Evolutionary Origin and Structural Diversification of the SRC Family

**Figure 7:**
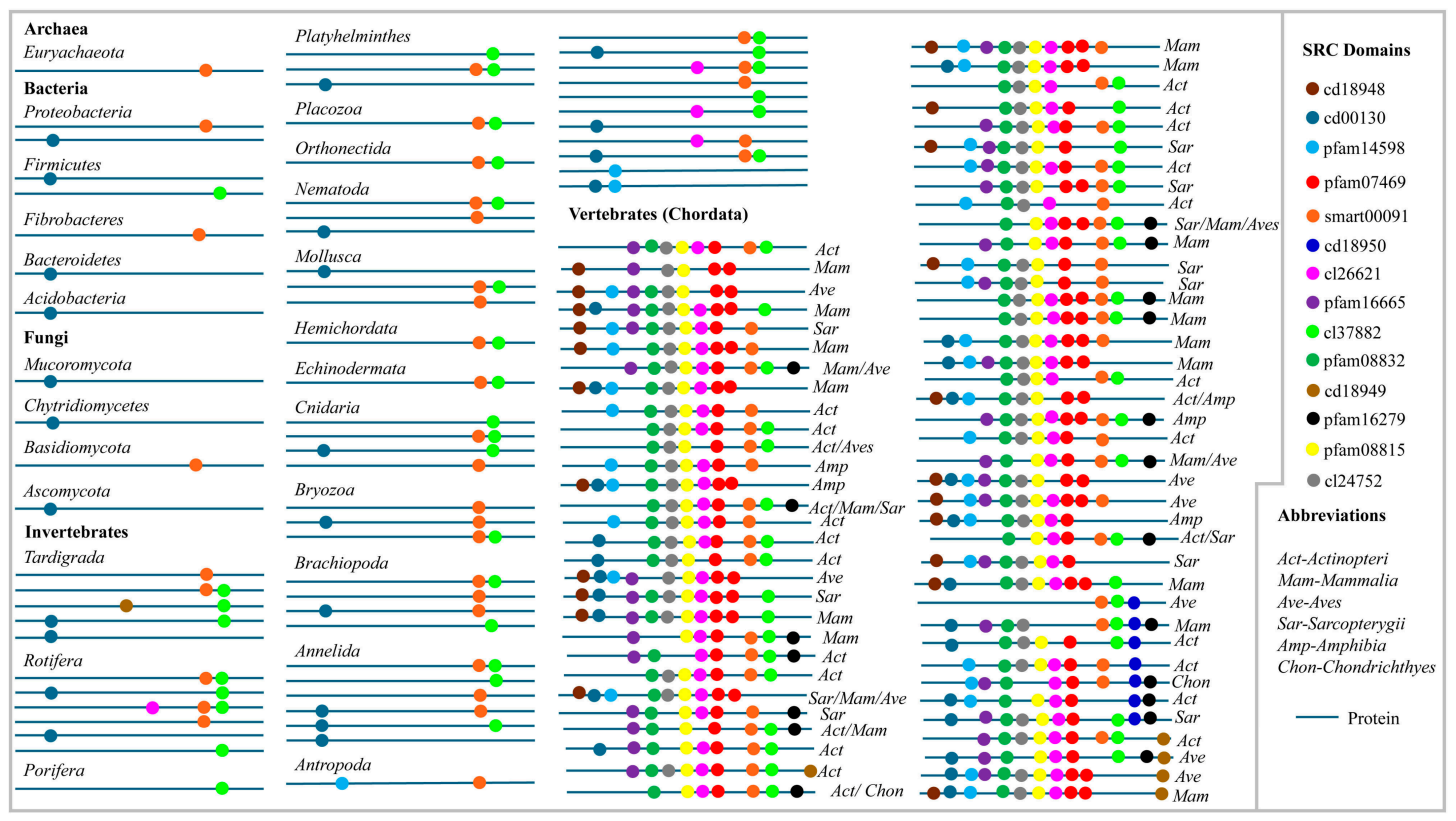
Schematic diagram representing the analysis of characteristics of steroid receptor coactivator members 1–3 (SRCs 1–3) domains in proteins from the species of different domains of life. The characteristic domains of SRCs 1–3 were presented using differently colored dots. The characteristic SRC domains from the National Center for Biotechnology Information (NCBI) Batch Web CD-Search Tool [24] are presented in the figure, and the species taxonomy is followed as per the NCBI taxonomy [26]. The information on different hit proteins with characteristic domain patterns is presented in Table S7.

The combined evidence from domain architecture, motif composition, amino acid conservation, and phylogenetic analyses supports a common evolutionary origin for the SRC family and provides a framework for reconstructing its emergence across the domains of life. To investigate the evolutionary origin and emergence of SRCs across the domains of life, a comprehensive domain architecture analysis was conducted using more than 20,000 initially identified protein hits. Among these, over 4000 proteins contained one or more domains characteristic of SRC proteins, in addition to the canonical SRC-1, SRC-2, and SRC-3 proteins identified in this study (Figure 7 and Table S7). The analysis revealed a complex evolutionary history possibly involving progressive domain acquisition, recombination, and conservation across diverse lineages.

Among the three life domains, the earliest SRC-associated signature identified was the Smart00091 domain, detected in archaeal proteins and conserved across all three vertebrate SRC paralogs (Figure 7). In Bacteria, proteins containing Smart00091 were identified alongside additional domains, including cd00130 and cl37882, both characteristic of modern SRC-1 proteins. Similarly, fungal proteins exhibited combinations of Smart00091 and cd00130, suggesting that these domains were retained and propagated during the transition from prokaryotic to lower-eukaryotic lineages. The occurrence of these domains across Archaea, Bacteria, and Fungi suggests that several structural components of modern SRC proteins predate the emergence of multicellular animals and likely served as evolutionary building blocks for the subsequent development of the SRC family.

A substantial increase in the complexity of SRC-associated domains was observed in invertebrates. Six characteristic SRC domains, including Smart00091, cd00130, cl37882, cd18949, cl26621, and Pfam14598, were identified in various combinations (Figure 7). Proteins containing only a single SRC-associated domain, as observed in Archaea, Bacteria, and Fungi, were also detected in invertebrates, suggesting the inheritance of ancestral domain architectures followed by gradual structural elaboration. In addition, numerous proteins containing combinations of two or three SRC-associated domains were identified across multiple invertebrate phyla (Figure 7). The widespread occurrence of these domain arrangements across taxonomically diverse invertebrates may represent ancestral modules that were successfully retained during speciation and subsequently served as substrates for further evolutionary innovation.

The greatest diversification of SRC domain architectures was observed within chordates. Analysis revealed numerous proteins with distinct combinations of SRC-associated domains that differed from those of the canonical vertebrate SRC-1, SRC-2, and SRC-3 proteins. Notably, the remaining eight characteristic SRC domains were detected exclusively in chordate lineages, indicating that the assembly of complete SRC architectures occurred relatively late during metazoan evolution. The presence of multiple intermediate domain combinations in chordates suggests that these proteins may represent transitional evolutionary forms that preceded the emergence of the canonical vertebrate SRC paralogs. Furthermore, the appearance of canonical SRC structural arrangements coincided with the emergence of vertebrate lineages, supporting the hypothesis that extensive domain shuffling and recombination preceded the formation of the contemporary SRC family.

Interestingly, several vertebrate groups contained proteins harbouring characteristic SRC domains despite the apparent absence of specific SRC paralogs (Figure 7 and Table S7). For example, although SRC-2 proteins were not identified in Actinopteri, proteins containing the SRC-2-associated domain cd18950 were detected. Similarly, Amphibia lacked identifiable SRC-1 proteins but contained proteins possessing characteristic SRC-1 domains. Likewise, Chondrichthyes lacked detectable SRC-1 and SRC-2 proteins, yet proteins carrying domains diagnostic of both paralogs were present. These observations suggest that key SRC structural modules may have evolved before the emergence of the complete SRC proteins. Two possible explanations could account for this observation: either these vertebrate lineages diverged before the complete assembly of specific SRC paralogs, or the corresponding SRC proteins were subsequently lost or remain undiscovered. However, given the extensive dataset analyzed in this study, comprising more than 20,000 protein sequences, the former explanation appears more consistent with the extensive taxonomic sampling employed here.

Overall, the results suggest that the evolution of SRC proteins was not a simple linear process but rather involved the gradual accumulation, rearrangement, and recombination of ancestral domains across the domains of life. The presence of SRC-associated domains in Archaea, Bacteria, Fungi, Invertebrates, and Vertebrates (Chordates) is consistent with a stepwise evolutionary model in which ancient protein modules were progressively combined to generate increasingly complex architectures. This process might have played a role in the emergence of the vertebrate SRC-1, SRC-2, and SRC-3 proteins, which subsequently diversified to fulfil specialized roles in nuclear receptor signaling and transcriptional regulation.

When considered together, the domain architecture, LXXLL motif composition, amino acid conservation, and phylogenetic analyses presented in this study support a model of progressive SRC evolution. Conserved domains and receptor-interaction motifs may represent ancient ancestral modules that were gradually assembled through domain acquisition and recombination events. Subsequent gene duplication and lineage-specific evolution gave rise to the modern SRC-1, SRC-2, and SRC-3 paralogs, each retaining a conserved functional core while acquiring distinct structural, regulatory, and functional characteristics. This integrated evolutionary framework provides new insight into the emergence and functional specialization of the vertebrate SRC family.

## 3. Materials and Methods

### 3.1. Genome Data Mining of Steroid Receptor Coactivator Members 1–3 (SRCs 1–3) Across the Domains of Life

Genome data mining of SRCs 1–3 proteins was carried out following the general workflow previously described by our laboratory [25,33]. Human SRC proteins with experimentally characterized domain architectures were used as reference sequences for the analysis, including SRC-1 (UniProt ID: Q15788), SRC-2 (Q15596), and SRC-3 (Q9Y6Q9). These reference proteins were initially compared using three domain annotation programs (Section 2.1) to determine the most suitable tool for distinguishing the characteristic domain architectures of the three SRC paralogs. Based on this comparison, the NCBI Batch Web CD-Search Tool was selected for subsequent genome-wide screening. The comparison of the three domain annotation tools was intended to determine which most effectively differentiated the three reference human SRC proteins used in this study. The selection of the NCBI Batch CD-Search Tool was based on its practical ability to distinguish the domain architectures of the three reference SRC proteins examined in this study. It should not be interpreted as validation of its superiority for all SRC-like proteins. A comprehensive benchmarking analysis using curated positive and negative datasets was beyond the scope of the present work.

Genome-wide sequence retrieval was performed using the HMMER search platform against [23] the UniProt protein database with the reference human SRC-1 (UniProt ID: Q15788) as the query. Human SRC-1 was selected because it contains domains shared with both SRC-2 and SRC-3 and is an ideal candidate for BLAST (version 2.17.0) analysis. More than 20,000 candidate proteins were retrieved and subsequently analyzed using the NCBI Batch Web CD-Search Tool [24]. Each candidate protein was examined for the presence and organization of the characteristic conserved domains associated with canonical SRC proteins (Figure 1). Only proteins exhibiting the characteristic domain architecture of SRC-1, SRC-2, or SRC-3 were retained for further analyses. This standardized domain-based filtering strategy reduced the initial dataset to 298 canonical SRC proteins, which served as the basis for all subsequent comparative analyses, including phylogenetic reconstruction, amino acid conservation, motif analysis, and structural characterization. Although the initial HMMER search was initiated using the human SRC-1 sequence, final protein classification was determined solely by conserved domain architecture identified using the NCBI Batch Web CD-Search Tool [24] (Figure 1), enabling discrimination of canonical SRC-1, SRC-2, and SRC-3 proteins independent of the initial query sequence. Hit proteins were classified as members of the SRC family based on the presence of characteristic conserved domains (Figure 1). Proteins containing all 10 characteristic domains, including two copies of pfam07469 (cd00130, cd18948, cl24752, cl26621, pfam14598, pfam08815, pfam08832, pfam16665), were classified as SRC-1. Proteins containing the eight characteristic domains (pfam07469, pfam08815, pfam08832, pfam16665, cd18950, cl37882, pfam16279, smart00091) were classified as SRC-2, whereas those containing the eight characteristic domains (pfam07469, pfam08815, pfam08832, pfam16665, cd18949, cl37882, pfam16279, smart00091) were classified as SRC-3. Hit proteins lacking the characteristic SRC domains were excluded from further analysis. Proteins containing one or more conserved SRC domains were retained for subsequent evolutionary analyses.

### 3.2. Phylogenetic Analysis

Phylogenetic analysis of SRC family proteins was performed following the workflow previously established by our laboratory [25,33] using the full-length SRC sequences. The amino acid sequences of the identified SRC proteins were aligned using MAFFT version 6.864 [34], implemented through the T-REX web server [35]. Sequence alignment was performed using the default Auto strategy, which selects an appropriate alignment algorithm based on the dataset’s size and complexity. In this case, the multiple alignment strategy was the progressive method Fast Fourier Transform-Normalized Similarity-1-cycle (FFT-NS-1). BLOSUM 62 was used as the substitution matrix, with a gap-opening penalty of 1.53 and an offset penalty of 0.123.

The resulting multiple sequence alignment was subsequently used to reconstruct the phylogeny using the Maximum Likelihood (ML) method available through the T-REX platform [35]. The ML tree inferred from the aligned SRC protein sequences was selected as the best-supported topology generated by the server and used for subsequent evolutionary analyses. The best-fitting amino acid substitution model selected by the software was used for tree reconstruction, and the tree file generated was visualized and annotated using the Interactive Tree of Life (iTOL) platform [36], where branch colours were assigned according to SRC paralog classification and node colours were used to represent vertebrate taxonomic groups. The tree was further formatted for publication-quality presentation.

### 3.3. Analysis of Amino Acid Conservation

The number of conserved amino acids in SRC-1 proteins was analyzed using the method previously described by our laboratory [25]. Amino acid conservation among SRC-1 proteins was examined using PROMALS3D [31]. PROMALS3D is a multiple sequence alignment tool that aligns protein sequences by incorporating homologous crystal structures, secondary structure prediction, and three-dimensional structural constraints. The output alignment assigns conservation indices ranging from 4 to 9, where 9 represents the most highly conserved amino acid positions among the analyzed SRC protein sequences. The conservation analysis was performed on full-length SRC protein sequences to provide an overall comparison of amino acid conservation among SRC family members. The conservation scores reported in this study were those generated directly by PROMALS3D [31].

### 3.4. Protein Modeling

Three-dimensional protein models for Human SRC-1, SRC-2, and SRC-3 proteins were generated using the AlphaFold3 database [32]. The 3D protein structures were visualized, and high-quality 3D protein model images were generated using the PyMol software, version 2.2.5 [37]. The human SRCs 1–3 sequences were aligned using the Clustal Omega program (version 1.2.4) [38]. Secondary structural elements inferred from the predicted 3D models and conserved amino acid residues by PROMALS3D were mapped onto the multiple sequence alignment.

### 3.5. Identification of LXXLL Motifs in SRC Proteins

The occurrence of LXXLL motifs, which represent the canonical nuclear receptor interaction motifs of steroid receptor coactivators (SRCs), was identified using a custom Microsoft Excel-based motif scanning tool developed for this study. The spreadsheet workflow was developed with assistance from ChatGPT (OpenAI, San Francisco, CA, USA), which was used solely to assist in implementing the computational workflow for automated motif identification. The computational logic and all generated outputs were independently tested, reviewed, and validated by the author before their application in this study. Protein sequences were obtained from publicly available databases and imported into the worksheet as plain text. Before analysis, sequences were automatically standardized by removing spaces, line breaks, and other non-amino-acid characters, and converted to uppercase.

The motif scanner systematically examined each protein sequence using a sliding five-residue window. At each position, the algorithm evaluated whether the first, fourth, and fifth residues were leucine (L) and allowed any amino acid at the second and third positions, consistent with the consensus motif LXXLL. When a match was detected, the program recorded the motif’s starting position and extracted the corresponding five-amino-acid sequence. The output generated a concise list of validated LXXLL motifs and their positions in the protein sequence. This approach enabled rapid, reproducible, and transparent identification of nuclear receptor-binding motifs across large SRC datasets without requiring specialized bioinformatics software. To verify the accuracy of the automated motif detection, all LXXLL motifs identified by the Excel-based workflow were manually inspected for every SRC protein analyzed in this study. Each reported motif position and corresponding amino acid sequence were independently checked against the original protein sequences to confirm correct identification of the canonical LXXLL motif. No differences were observed between the manual checking of LXXLL motifs and those identified by the program. The complete Excel-based motif scanner used in this study is provided as Supplementary Dataset S3 to facilitate reproducibility of the analysis.

### 3.6. Use of Generative AI

ChatGPT 5.3 version (OpenAI, San Francisco, CA, USA) and Grammarly (premium version) were used solely to assist with language editing, formatting, graphical abstract preparation, and development of Microsoft Excel formulas for the LXXLL motif-scanning workflow described in Section 3.5. These tools were not used for scientific interpretation, data analysis, data validation, or drawing scientific conclusions. All computational workflows, analyses, motif identifications, interpretation of results, and the final manuscript were independently reviewed and verified by the authors, who accept full responsibility for the content of this publication.

## 4. Conclusions

This study presents a comparative genomic framework of the steroid receptor coactivator (SRC) family across the domains of life. Using a combination of genome-wide screening, domain architecture analysis, phylogenetic reconstruction, motif characterization, and amino acid conservation analyses, the evolutionary history of SRC proteins was systematically investigated. The results demonstrated that the NCBI Batch CD-Search Tool was suitable for identifying and classifying the reference SRC proteins examined in this study and was therefore used for subsequent genome-wide analyses. Although more than 20,000 proteins were initially examined during genome-wide data mining, only proteins that satisfied the predefined conserved-domain criteria were classified as canonical SRC family members, thereby minimizing the inclusion of unrelated proteins despite potential differences in database representation.

Domain-based evolutionary reconstruction suggested that the ancestral SRC architecture likely emerged through the progressive accumulation, recombination, and structural refinement of pre-existing protein domains traceable to lineages predating the emergence of vertebrates. The presence of SRC-associated domains across Archaea, Bacteria, Fungi, Invertebrates, and Chordates supports a stepwise evolutionary model in which ancient protein modules were gradually assembled into increasingly complex architectures. Following the establishment of this ancestral SRC framework, phylogenetic analyses suggested that ancient vertebrate gene duplication events may have given rise to the modern SRC paralogs. Subsequent lineage-specific evolution resulted in distinct taxonomic distributions, structural characteristics, and functional properties among SRC-1, SRC-2, and SRC-3. Although full-length protein sequences provide an overview of the evolutionary relationships among SRC paralogs, these proteins contain extensive intrinsically disordered regions that may reduce phylogenetic resolution. Future studies employing conserved-domain-only phylogenies together with orthology-based analyses may provide additional insights into the evolutionary history of the SRC family.

Comparative analyses of LXXLL nuclear receptor interaction motifs revealed extensive conservation of core receptor-binding motifs, alongside the emergence of paralog-specific motif patterns that may have contributed to functional specialization. Structural analyses further suggested that, despite extensive diversification, members of the SRC family retain highly conserved domain organization, secondary structural elements, and amino acid residues possibly associated with essential biological functions. Notably, SRC-2 exhibited the highest amino acid conservation, suggesting stronger evolutionary constraints than SRC-1 and SRC-3.

Collectively, these findings suggest an evolutionary scenario in which ancestral protein modules progressively assembled into an early SRC architecture that subsequently evolved through gene duplication, structural innovation, and functional specialization. Integrating domain architecture, motif composition, sequence conservation, and phylogenetic evidence provides a unified framework for understanding the emergence and evolutionary history of the SRC family across the domains of life. Although the widespread occurrence of SRC-associated domains across bacteria, archaea, fungi, invertebrates, and vertebrates is consistent with a stepwise domain acquisition model, these observations alone do not establish a direct evolutionary pathway leading to modern SRC proteins. Alternative evolutionary scenarios, including convergent evolution, independent domain shuffling, repeated domain reuse, incomplete lineage sampling, and annotation bias, may also explain the observed distribution of individual domains. Therefore, the evolutionary model proposed in this study should be regarded as a hypothesis, supported by comparative genomic observations, that warrants further validation through reciprocal homology analyses, domain-specific phylogenies, and orthology-based evolutionary reconstruction.

Furthermore, because this study is based on publicly available protein records, the observed distribution patterns should be interpreted in the context of current database coverage. Differences in genome sequencing, annotation quality, and taxonomic sampling may influence the number of proteins retrieved for individual vertebrate groups. Nevertheless, all proteins included in this study satisfied the same domain-based identification criteria, providing a consistent framework for comparative evolutionary analyses.

## Figures and Tables

**Table 1:** Comparative analysis of amino acid conservation in steroid receptor coactivators 1–3 (SRCs 1–3). Amino acid conservation was assessed using Profile Multiple Alignment with predicted Local Structures and 3D constraints (PROMALS3D) [31], and conservation scores of 4–9 are presented in the table. Furthermore, the number of amino acids conserved in different domains, if any, and their secondary structural features are also indicated in the table.

		Combined All SRCs	SRC-1	SRC-2	SRC-3
	No. of proteins	298	160	43	95
PROMALS3D					
	5	94	77	0	199
	6	112	210	615	17
	7	190	122	0	294
	8	0	0	0	0
	9	143	306	500	235
Domains	Cd18948		33/α-Helix		
	Cd18950			40/α-Helix	
	Cd18949				32/α-Helix
	Cd00130		52/α-Helix		
	Smart00091			42/α-Helix & β-Sheet	31/α-Helix & β-Sheet
	Pfam14598		31/α-Helix & β-Sheet		
	Cl37882		N/A	43/α-Helix & β-Sheet	20/α-Helix & β-Sheet
	Pfam16665		37	37	14
	Pfam08832		26/α-Helix	26/α-Helix	14/α-Helix
	Cl24752		13/α-Helix		
	Pfam16279			23/α-Helix	9/α-Helix
	Pfam08815		20/α-Helix	14/α-Helix	15/α-Helix
	Cl26621		63/α-Helix		
	Pfam07469		19 & 12	22	7

## Data Availability

All data supporting the results of this study are provided in the Supplementary Material (Tables S1–S7; Figures S1 and S2, Supplementary Datasets S1–S3).

## Supplementary Materials

The following supporting information can be downloaded at: https://www.mdpi.com/article/10.3390/molecules31152698/s1, Figure S1: Multiple sequence alignment of Steroid receptor coactivators (SRCs 1–3) proteins from *Homo sapiens*. The secondary structural elements were mapped, with amino acids underlined in red for alpha helices and blue for beta sheets. The conserved amino acids are capitalized and bold. Amino acids in different domains are indicated with different colors. The color-coded representation of the different domains is shown at the bottom of the figure; Figure S2: A high-resolution phylogenetic tree of steroid receptor coactivator family members (SRC-1 to SRC-3); Table S1: Information on steroid receptor coactivator-1 (SRC-1) proteins identified in this study and their domain analysis. NCBI taxonomy was followed for species classification; Table S2: Information on steroid receptor coactivator-2 (SRC-2) proteins identified in this study and their domain analysis. NCBI taxonomy was followed for species classification; Table S3: Information on steroid receptor coactivator-3 (SRC-3) proteins identified in this study and their domain analysis. NCBI taxonomy was followed for species classification; Table S4: LXXLL motif analysis in steroid receptor coactivator-1 (SRC-1) proteins identified in this study; Table S5: LXXLL motif analysis in steroid receptor coactivator-2 (SRC-2) proteins identified in this study; Table S6: LXXLL motif analysis in steroid receptor coactivator-3 (SRC-3) proteins identified in this study; Table S7: Steroid receptor coactivator characteristics domain analysis in proteins from species across the domains of life. NCBI taxonomy was followed for species classification; Supplementary Dataset S1: Protein sequences of steroid receptor coactivator (SRC) family members (SRC-1, SRC-2, and SRC-3) used for phylogenetic tree construction. For each SRC sequence, the species name, UniProt protein accession ID, phylum, and SRC subtype are provided. A unique numerical identifier preceding each species name is included solely for reference purposes and to facilitate tracking of individual SRC sequences throughout the dataset; Supplementary Dataset S2: CLUSTAL-format multiple sequence alignment of SRC-1, SRC-2, and SRC-3 protein sequences used for phylogenetic analysis; Supplementary Dataset S3: The Excel file with the LXXLL motif scanner program.
