# Supplementary material for: Comparative Genomics Provides Insights into the Evolutionary Origin and Structural Diversification of Steroid Receptor Coactivators (SRCs 1–3)
Source: Molecules. 2026 Aug 3;31(15):2698. doi: 10.3390/molecules31152698 (PMC13468300; doi:10.3390/molecules31152698)
Supplement: Supplementary file 1 [file molecules-31-02698-s001.zip › Figure S1.pdf]

Figure S1: Multiple sequence alignment of Steroid receptor coactivators (SRCs 1-3) proteins from Homo sapiens. The secondary structural elements were mapped, with amino acids underlined in red for alpha helices and blue for beta sheets. The conserved amino acids are capitalized and bold. Amino acids in different domains are indicated with different colors. The color-coded representation of the different domains is shown at the bottom of the figure.

```
Q15788|SRC1      MSGLGDSSSDPANPDSHKRKGSPCDT----LASSTEKRRREQENKYLEELAELLSANISD     56
Q15596|SRC2      MSGMGENTSDPSRAETRKRKE-CPDQLGPSPKRNTEKRNREQENKYIEELAELIFANFND    59
Q9Y6Q9|SRC3      MSGLGENLD-PLASDSRKRKL-PCDTPGQGLTCSGEKRRREQESKYIEELAELISANLSD     58

Q15788|SRC1      IDSLSVKPDKCKILKKTVDQIQLMKRMEQEKSTTDDDVQKSDISSSSQGVIEKESLGPLL     116
Q15596|SRC2      IDNFNFKPDKCAILKETVKQIRQIKEQEKAAAANIDEVQKSDVSSTGQGVIDKDALGPMM    119
Q9Y6Q9|SRC3      IDNFNVKPDKCAILKETVRQIRQIKEQGKTI-SNDDDVQKADVSSTGQGVIDKDSLGPLL    117

Q15788|SRC1      LEALDGFFFVVNCEGRIVFVSENVTSYLGYNQEELMNTSVYSILHVGDHAEFVKNLLPKS  176
Q15596|SRC2      LEALDGFFFVVNLEGNVVFVSENVTQYLRYNQEELMNKSVYSILHVGDHTEFVKNLLPKS 179
Q9Y6Q9|SRC3      LQALDGFLFVVNRDGNIVFVSENVTQYLQYKQEDLVNTSVYNILHEEDRKDFLKN-LPKS    176

Q15788|SRC1      LVNGVPWPQEATRRNSHTFNCRMLIHPPD---EPGTENQEACQRYEVMQCFTVSQPKSIQ     233
Q15596|SRC2      IVNGGSWSGEPPRRNSHTFNCRMLVKPLPDSEEEGHDNQEAHQKYETMQCFAVSQPKSIK     239
Q9Y6Q9|SRC3      TVNGVSWTNETQRQKSHTFNCRMLMKTPHDILEDINASPEMRQRYETMQCFALSQPRAMM 236
```

```
Q15788|SRC1    EDGEDFQSCLICIARRLPRP--PAITGVESFMTKQDTTGKIISIDTSSLRAAGRTGWEDL 291
Q15596|SRC2    EEGEDLQSCLICVARRVPMKERPVLPSSESFTTRQDLQGKITSLDTSTMRAAMKPGWEDL 299
Q9Y6Q9|SRC3    EEGEDLQSCMICVARRITTGERTFPSNPESFITRHDLSGKVVNIDTNSLRSSMRPGFEDI 296

Q15788|SRC1    VRKCIYAFFQP-QGREPSYARQLFQEVMTRGTASSPSYRFILNDGTMLSAHTKCKLCYPQ 350
Q15596|SRC2    VRRCIQKFHAQHEGESVSYAKRHHHEVLRQGLAFSQIYRFSLSDGTLVAAQTKSKLIRSQ 359
Q9Y6Q9|SRC3    IRRCIQRFFSLNDGQSWSQ-KRHYQEAYLNGHAETPVYRFSLADGTIVTAQTKSKLFRNP 355

Q15788|SRC1    SPDMQPFIMGIHIIDREHSGLSPQDDTNSGMSIPRVNPSVNPSISPAHGVARSSTLPPSN 410
Q15596|SRC2    TTNEPQLVISLHMLHREQNVCVMNPDLTGQTMGKPLNPISS--NSPAHQALC-SGNPGQD 416
Q9Y6Q9|SRC3    VTNDRHGFVSTHFLQREQNGYRPNPNPVGQGIRPPMAGCNS--SVG----GM-SMSPNQG 408

Q15788|SRC1    SNMVSTRINRQQSSDLHSSSHSNSSNSQGSFGCSPGSQIVANVALNQGQASSQSSNPSLN 470
Q15596|SRC2    MTLSS-NINF----PIN-GPKEQMGMPMGRFGGSGGMNHVS---GMQ-ATTPQGSNYALK 466
Q9Y6Q9|SRC3    LQMPS-SRAY----GLA-DPSTTGQMSGARYGGSSNIASLTPGPGMQSPSSYQNNNYGLN 462

Q15788|SRC1    LNNSPMEGTGISL---AQFMSPRRQVTSGLATRPRMPNNSFPPNISTLSSPVGMTSSACN 527
Q15596|SRC2    MNSPSQSSPGMNPGQPTSMLSPRHRMSPGVAGSPRIPPSQFSPAGS-LHSPVGVCS--ST 523
Q9Y6Q9|SRC3    MSSPPHGSPGLAPNQQNIMISPRNR------GSPKIASHQFSPVAG-VHSPMASSG--NT 513

Q15788|SRC1    NNNRSYSNIPVTSLQGMNEGPNNSVGFSASSPVLRQMSSQNSPSRLNIQPAKA-ESKDNK 586
Q15596|SRC2    GNSHSYTNSSLNALQALSEGHGVSLGSSLASPDLKMGNLQNSPVNMNPPPLSKMGSLDSK 583
Q9Y6Q9|SRC3    G-NHSFSSSSLSALQAISEGVGTSLLSTLSSPGPKLDNSPN----MNITQPSKVSNQDSK 568
```

```
Q15788|SRC1      EIASILNEMIQSDNSS--SDGKPLDSGLLHNNDR---------LSDG--DSKYSQTSHKL   633
Q15596|SRC2      DCFGLYGEPSEGTTGQAESSCHPGE--QKETNDPNLPPAVSSERADGQSRLHDSKGQTKL   641
Q9Y6Q9|SRC3      SPLGFYCDQNPVES----SMCQSNS--RDHLSDK-ESKESSVEGAENQRGPLESKGHKKL   621

Q15788|SRC1      VQLLTTTAEQQ----LRHADIDTSCKDVLSCTGTSNSASANSS---GGSCPSSHSSLTER   686
Q15596|SRC2      LQLLTTKSDQMEPSPLASSLS---------------DTNKDSTGSLPGSGSTHGTSLKEK   686
Q9Y6Q9|SRC3      LQLLTCSSDDRGHSSLTNSPLDSSCKESSVSVTSPSGVSSSTSGGVSSTSNMHGSLLQEK   681

Q15788|SRC1      HKILHRLLQEGS-PSDITTLSVEPDKKDSASTSVSVTGQVQGNSSIKLELDASKKKESKD 745
Q15596|SRC2      HKILHRLLQDSSSPVDLAKLTAEATGKDLSQESSSTA--PGSEVTIKQ--EPVSPKKK-E     741
Q9Y6Q9|SRC3      HRILHKLLQNGNSPAEVAKITAEATGKDTSSIT---S--CGDGNVVKQ--EQLSPKKKEN 734

Q15788|SRC1      HQLLRYLLDKDEKDLRSTPNLSLDDVKVKVEK-KEQMDPCNTNPTPMTK-PTPEEIKLEA 803
Q15596|SRC2      NALLRYLLDKDDTKDIGLPEITP-----KLERLDSKTDPASNTKLIAMKTEK-EEMSFEP 795
Q9Y6Q9|SRC3      NALLRYLLDRDDPSDALSKELQP-----QVEGVDNKMSQCTSSTIPSSSQEKDPKIKTET   789

Q15788|SRC1      QSQFTADLDQFDQLLPTLEKAAQLPGLCETD-----------------------------   834
Q15596|SRC2      GDQPGSELDNLEEILDDLQNSQL-PQLFPDTRPGAPAGSVDKQAIINDLMQLTAENSPVT 854
Q9Y6Q9|SRC3      SEEGSGDLDNLDAILGDLTSSDF-YNNSISS-NGSHLGT--KQQVFQ-------------   832

Q15788|SRC1      ------------------------------------------------------------   834
Q15596|SRC2      PVGAQKTALRISQSTFNNPRPGQLGRLLPNQNLPLDITLQSPTGAGPFPPIRNSSPYSVI     914
Q9Y6Q9|SRC3      --GTNSLGLKSSQS---------VQSIRP--PYNRAVSLDSPVSVGSSPPVKNISAFPML   879
```

```
Q15788|SRC1     ----------RM-----------DGAVTSVTIKSE-----I-------------------   849
Q15596|SRC2     PQPGMMG-NQGMIGNQGNLGNSSTGMIGNSASRPTMPSGEWAPQSSAVRVTCAATTS---  970
Q9Y6Q9|SRC3     PKQPMLGGNPRMMDSQENYGSSMGGPNRNVTVTQTPSSGDWGLPNSKAGRMEPMNSNSMG  939

Q15788|SRC1     ---------LPA-----S----LQSATARPTSRLN--------------RLPELELEAID  877
Q15596|SRC2     --------AMNRPVQGGMIRNPAASIPMRPSSQPGQRQTLQ--SQVMNIGPSELEMNMGG  1020
Q9Y6Q9|SRC3     RPGGDYNTSLPRPALGGS----IPTLPLRSNSIPGARPVLQQQQQMLQMRPGEIPMGMGA  995

Q15788|SRC1     NQFGQP--GTGDQIPWTNNTVTA--INQSKSEDQCISSQLDELLCPPTTVEGRNDEKALL  933
Q15596|SRC2     PQYSQQQAPPNQTAPWPESILPIDQASFASQNRQPFGSSPDDLLCPHPAAESPSDEGALL 1080
Q9Y6Q9|SRC3     NPYGQA-AASNQLGSWPDGMLSMEQVSHGTQNRPLLRNSLDDLVGPPSNLEGQSDERALL  1054

Q15788|SRC1     EQLVSFLSGKDETELAELDRALGIDKLVQGGG-LDVLSERFPPQQATPPLIMEERPNLYS     992
Q15596|SRC2     DQLYLALRN--FDGLEEIDRALGIPELVSQSQAVDPEQ--FSSQ--DSNIMLEQKAPVFP 1134
Q9Y6Q9|SRC3     DQLHTLLSNTDATGLEEIDRALGIPELVNQGQALEPKQDAFQGQ--EAAVMMDQKAGLYG 1112

Q15788|SRC1     QPYSSPSPTANLP-----SPFQGMVRQKPSLGTMPVQVTPPRGAFSPGMGMQPRQTLN--  1045
Q15596|SRC2     QQYASQAQMAQGSYSPMQDPNFHTMGQRPSYATL---------------RMQPRPGLRPT  1179
Q9Y6Q9|SRC3     QTYPAQGPPMQGG--------FHLQGQSPSFNSMMNQMNQQ-GN-FPLQGMHPRANIM--  1160

Q15788|SRC1     -RPPAAPNQLRLQLQQRLQGQQQLIHQNRQAILNQFAATA-PVGINMRSGMQQQI-TPQP     1102
Q15596|SRC2     GLVQNQPNQLRLQLQHRLQAQQNRQ-----PLMNQISNVS-----NVNLTLRPGV-PTQA 1228
Q9Y6Q9|SRC3     RPRTNTPKQLRMQLQQRLQGQQFLNQ-SRQALELKMENPTAGGAAVMRPMMQPQVSSQQG 1219
```

```
Q15788|SRC1    PLNAQMLAQRQRELYSQQHRQRLIQQ--------------------------QRAMLMRQQS 1139
Q15596|SRC2    PINAQMLAQRQREILNQHLRQRQMHQQ-------------------QQVQQRTLMMRGQG 1269
Q9Y6Q9|SRC3    FLNAQMVAQRSRELLSHHFRQQRVAMMMQQQQQQQQQQQQQQQQQQQQQQQQQQQQQQTQA 1279

Q15788|SRC1    F--GNNLPPSSGLPVQMGNPRLPQGAPQQFPYPPNYGTNPGTPPASTSPFSQLAANPEAS 1197
Q15596|SRC2    LNMTPSMVAPSGMPATMSNPRIPQANAQQFPFPPNYGISQQPDPGFT------------- 1316
Q9Y6Q9|SRC3    FSPPPNVTASPSMDGLLAGPTMPQAPPQQFPYQPNYGMGQQPDPAFG------------- 1326

Q15788|SRC1    LANRNSMVSRGMTGNIGGQFGTGINPQMQQNVFQYPGAGMVPQGEANFAPSLSPGSSMVP 1257
Q15596|SRC2    ------------------------------------------------GATTPQSPLMS 1327
Q9Y6Q9|SRC3    ------------------------------------------------RVSSPPNAMMS 1337

Q15788|SRC1    MPIPPPQSSLLQQTPPASGYQS-PDMKAWQQGAIGNNNVFSQAVQNQ-PTPAQP-GVYNN 1314
Q15596|SRC2    PRMAHTQSPMMQQSQANPAYQAPSDINGWAQGNMGGNSMFSQQSPPHFGQQANTSMYSNN 1387
Q9Y6Q9|SRC3    SRMGPSQNPMMQHPQAASIYQS-SEMKGWPSGNLARNSSFSQQQFAHQGNPAVYSMVH-- 1394

Q15788|SRC1    MSITVSMAGGNTNVQNMNPMMAQM---QMSSLQMPGMNTVCPEQINDPALRHTGLYCNQL 1371
Q15596|SRC2    MNINVSMATNTGGMSSMNQMTGQISMTSVTSVPTSGLSSMGPEQVNDPALRGGNLFPNQL 1447
Q9Y6Q9|SRC3    ------MNGSSGHMGQM----------NMNPMPMSGM-PMGPD----------------- 1420

Q15788|SRC1    SSTDLLKTEADGTQQVQQVQVFADVQCTVNLVGGDPYLNQPGPLGTQKPTSGPQTPQAQQ 1431
Q15596|SRC2    PGMDMIKQEGDTTRKYC------------------------------------------ 1464
Q9Y6Q9|SRC3    -------------QKYC------------------------------------------ 1424
```

```
Q15788|SRC1        KSLLQQLLT    1440
Q15596|SRC2        ----------   1464
Q9Y6Q9|SRC3        ----------   1424
```

Domain color codes:

- Cl26621(Med15 superfamily)
- Cl37882 (PAS_11 superfamily)
- Smart00091 (PAS)
- Pfam07469 (DUF1518)
- Cd18950 (bHLH-PAS_NCoA2_SRC2)
- Cd18948 (bHLH-PAS_NCoA1_SRC1)
- Cd00130 (PAS)
- Pfam14598 (PAS_11)
- Pfam16665 (NCOA_u2)
- Pfam08832 (SRC-1)
- Pfam16279 (DUF4927)
- Cd18949 (bHLH-PAS_NCoA3_SRC3)
- Cl24752 (DUF4927 superfamily)
- Pfam08815 (Nuc_rec_co-act)
