# Supplementary material for: Comparative Genomics Provides Insights into the Evolutionary Origin and Structural Diversification of Steroid Receptor Coactivators (SRCs 1–3)
Source: Molecules. 2026 Aug 3;31(15):2698. doi: 10.3390/molecules31152698 (PMC13468300; doi:10.3390/molecules31152698)
Supplement: Supplementary file 1 [file molecules-31-02698-s001.zip › Figure S2.pdf]

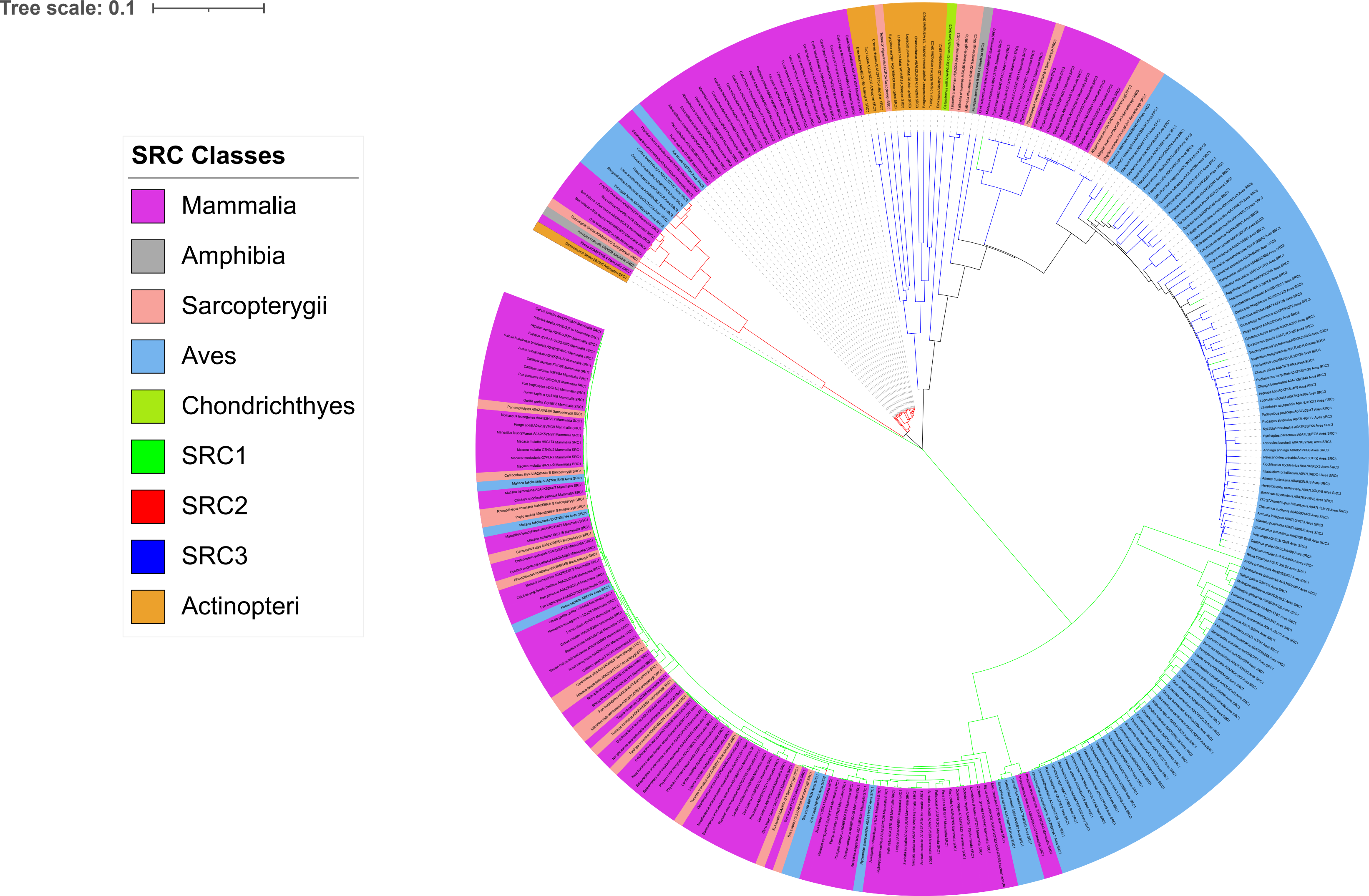
Tree scale: 0.1
SRC Classes
Mammalia
Amphibia
Sarcopterygii
Aves
Chondrichthyes
SRC1
SRC2
SRC3
Actinopteri
