# Supplementary material for: Comparative Genomics Provides Insights into the Evolutionary Origin and Structural Diversification of Steroid Receptor Coactivators (SRCs 1–3)
Source: Molecules. 2026 Aug 3;31(15):2698. doi: 10.3390/molecules31152698 (PMC13468300; doi:10.3390/molecules31152698)
Supplement: Supplementary file 1 [file molecules-31-02698-s001.zip › Supplementary dataset S1.pdf]

Supplementary Dataset 1: Protein sequences of steroid receptor coactivator (SRC) family members (SRC-1, SRC-2, and SRC-3) used for phylogenetic tree construction. For each SRC sequence, the species name, UniProt protein accession ID, phylum, and SRC subtype are provided. A unique numerical identifier preceding each species name is included solely for reference purposes and to facilitate tracking of individual SRC sequences throughout the dataset.

```
>1Aptenodytes forsteri|A0A087RA15|Aves|SRC1
MSGLGDSSTDPANPDSRKRKGSPCDTAQSNEKRRREQENKYLEELAELLSANIGDIDTLSVKPDKCKILKKTVD
QIQQMKRLEQEKAADDEVQKSDISSSSQGVIEKESLGPLLLEALDGFFFVVNREGRIVFVSENVTSYLGYNQEE
LMNTSVYSILHVGDHAEFVKNLLPKSLVNGVPWPQEATRRNSHTFSCRMLIRPPDEPGAENQEARQRYEVMQCF
TVSQPKSFKEEGEDFQSCLICIARRLPRPAAVTPSESFVTKQDTTGKIISIDTSSLRAAGRTGWEDLVRKCIYA
FFQPQGREPSYAKQLFQEVMTRGTAFSPSYRFTLSDGTVLSAHTKCKLCYPSSPEMQPFIMGIHVIDRDHGMLS
PQENTNSAMSLPRVSPSLNPSISPGQGMALPPSIPPSNGSMLASNVNQQPGAGLHNSNSSTSQTSFGCSPGNQI
GANVALSQGQAGPQNSNHTLNLNSSPMNSPGITPPQFMSPRHRVSPGLVPRPRVPSNPFSPTMPTMHSPVGMAN
SGSGGGSGTGGTRPFPSAPINSMQGLTEGPSTPVGYSTPPPVLRQLSSQSSPGRLAMQPMKAESKDSKEIASIL
SEMIQSDGGSGDGKPLDSGMLHAGDRLSEGDSKCSQATSHKLVQLLATTAEQQLRHADADTSCKDSLACAGTAG
TLASGNPPSSTCPSSHSSLTERHKILHRLLQEGSPSDITTLAMEHEKKENAPNPATTPTAQSQLPGTPDIKLEP
DALKKKDVKDHQLLRYLLDKDEKELAAAPALSLDDVKVKVEKTEQMEPCNTAPVPLAKPPATEEVKLETQGQFA
AELEQLDQLLPSLEKAAQLPGLCGPERNESGVAGVTVKPEMLAAPLQPSTAARAPTGVRRTSMNSGQGVQAGRT
PFEFCDPLEPAQLRPVPCPASNGSSPHARGPSQQGRGTVTAPNPFPPDTGMPELEMVPDHQFGQPGMGEQIPWT
DNSMAAMNRGALNKPEDQCITSQLDELLCPPTTPEGRNDEKALLEQLVSFLSGKDETELAELDRALGIDKLVQG
GGLEALTERFQPQQATPPLMMEQKPSMYPQPYSSASPTTNLPGAFPGMVRQKPSFGPMPVQVPPPRGAFPTSMA
MQPRQTLNRPPTAPNQLRLQLQQRLQGQQQLIHQNRQAILNQFAANSPVGINMRAGMQQQITPQPPLNAQMLAQ
RQRELYSQQHRQRQLMQQRAILMRQQSFGNNLPPSAGLPVQIGAARLPQAPPQQFPYPPNYGTTPGNPPTSTSP
FSPLASNPEAALANRSSMVNRGMMGGVGGQFGAGMTPQMQQNIFQYSGSGMGQQSEPAFAPSLSPSSPLMSPQL
PPSQSPMLQPAPPTPGYQSPDMKTWQQGAMGNSNVFSQAGQSQPAPAQQGMYNNMSITVSMAGGNTNVQNMNPM
AGQMQMNSLQMPGMNSMCSEQVNDPALRPAGLYCNQLSSTDLLKTEADGAQ
>2Nipponia nippon|A0A091V8Z5|Aves|SRC1
MSGLGDSSTDPANPDSRKRKGSPCDTAQSNEKRRREQENKYLEELAELLSANIGDIDTLSVKPDKCKILKKTVD
QIQQMKRLEQEKAADDEVQKSDISSSSQGVIEKESLGPLLLEALDGFFFVVNREGRIVFVSENVTSYLGYNQEE
LMNTSVYSILHVGDHAEFVKNLLPKSLVNGVPWPQETTRRNSHTFSCRMLIRPPDEPGAENQEARQRYEVMQCF
TVSQPKSFKEEGEDFQSCLICIARRLPRPAAVTPSESFVTKQDTTGKIISIDTSSLRAAGRTGWEDLVRKCIYA
FFQPQGREPSYAKQLFQEVMTRGTAFSPSYRFTLSDGTVLSAHTKCKLCYPSSPEMQPFIMGIHIIDRDHGMLS
PQENTNSAMSLPRVSPSLNPSISPGQSMALPPSIPPSNGSMLAANVNQQPGTGLHNSNSSTSQTSFGCSPGNQI
GANVALSQGQAGPQNSNHTLNLNSSPMNSPGITPPQFMSPRHRVSPGLVPRPRVPSNPFSPTMPTMHSPVGMAN
SGSGGGGGTGGTRPFPNAPINSMQGLTEGPSTPVGYSTPPPVLRQLSSQSSPGRLAMQPMKAESKDSKEIASIL
SEMIQSDGGSGDGKPLDSGMLHASDRLSEGDSKCSQATSHKLVQLLATTAEQQLRHADADTSCKDSLACAGTAG
TLASGNPPSSTCPSSHSSLTERHKILHRLLQEGSPSDITTLAMEHEKKENAPNPATTPTAQSQLPGTPDIKLEP
DTLKKKDVKDHQLLRYLLDKDEKELAAAPALSLDDVKVKVEKTEQMEPCNTAPVPLAKPPAAEEVKLETQGQFA
AELEQLDQLLPSLEKAAQLPGLCGPERNESSVASVAVKPEMLAAPLQPSTAARAPTGLNRTSMNSGQGVQAGRT
PFEFCDPLEPAQMRPVPCPASNGSSPHARGPSQQGRGTVTAPNPFPPDTGRPELEMGVPDHQFGQPGMGEQIPW
TDNSMSAMNRGALNKPEDQCITSQLDELLCPPTTPEGRNDEKALLEQLVSFLSGKDETELAELDRALGIDKLVQ
GGGLEALTERFQPQQATPPLMMEQKPGMYPQPYSSASPTTNLPGAFPGMVRQKPSFGPMPVQVPPPRGAFPTSM
AMQPRQTLNRPPTAPNQLRLQLQQRLQGQQQLIHQNRQAILNQFAANSPVGINVRAGMQQQITPQPPLNAQMLA
QRQRELYSQQHRQRQLMQQRAILMRQQSFGNNLPPSAGLPVQIGAARLPQAPPQQFPYPPNYGTTPGNPPTSTS
PFSPLASNPEAALANRSSMVNRGMMGGVGGQFGAGMTPQMQQNIFQYSGSGMGQQSEPAFAPSLSPSSPLMSPQ
LPPSQSPMLQPAPPTPGYQSPDMKTWQQGAMGNSNVFSQAGQSQPAPAQQGMYNNMSITVSMAGGNTNVQNMNP
MAGQMQMNSLQMPGMNSMCSEQVNDPALRPAGLYCNQLSSTDLLKTEADGAQQVQQVQVFADVQCTVNLVGDPY
LNPPAPLGTPKNAAGPPPPPGQQKSLLQQLLTE
```

>3Charadrius vociferus|A0A0A0A5N7|Aves|SRC1
MSGLGDSSTDPANPDSRKRKGSPCDTAQSNEKRRREQENKYLEELAELLSANIGDIDTLSVKPDKCKILKKTVD
QIQQMKRLEQEKTADDEVQKSDISSSSQGVIEKESLGPLLLEALDGFFFVVNREGRIVFVSENVTSYLGYNQEE
LMNTSVYSILHVGDHAEFVKNLLPKSLVNGVPWPQEVTRRNSHTFSCRMLIRPPDEPGAENQEARQRYEVMQCF
TVSQPKSFKEEGEDFQSCLICIARRLPRPAAVAPSESFVTKQDTTGKIISIDTSSLRAAGRTGWEDLVRKCIYA
FFQPQGREPSYAKQLFQEVMTRGTAFSPSYRFTLSDGTVLSAHTKCKLCYPSSPEMQPFIMGIHVIDRDHGMLS
PQENTNSAMSLPRVSPSLNPSISPGQGMTLPPSIPPSNGSMLATNVNQQPGAGLHNSTSSTTPTSPMNSPGITP
PQFMSPRHRVSPGLVPRPRVPSNPFSPTMPTMHSPVGMANSAGGGGTGGTRPFPSAPMNSMQGLTEGPSTPVGY
STPPPVLRQLSSQSSPGRLAMQPMKAESKDSKEIASILSEMIQSDGGSGDGKPLDSGMLHAGDRLSEGDSKCSQ
ATSHKLVQLLATTAEQQLRHADADTSCKDSLACAGTAGTLASGNPPSSTCPSSHSSLTERHKILHRLLQEGSPS
DITTLAMEHEKKENAPNPATTPTAQSQLPGTPDIKLEPDALKKKDVKDHQLLRYLLDKDEKELAAAPALSLDDV
KVKVEKTEQMEPCNTAPVPLAKPPAAEEVKLETQGQFAAELEQLDQLLPSLEKAAQLPGLCGPERSESGVAGVT
VKPEMLAAPLQPSTAARAPAGLNRTSMNSGQGVQAGRTPFEFCDPLEPAQLRPVPCPASNGSSPHARGPSQQGR
GTVTAPNPFPPDTGMYGRGHPLLSTFSSMAAMNRGALNKPEDQCITSQLDELLCPPTTPEGRNDEKALLEQLVS
FLSGKDETELAELDRALGIDKLVAGGREPLTKRSQPQQATPPLMMEQKPGMYPQPYSSASPTTNLPGAFPGMVR
QKPSFGPMPVQVPPPRGAFPTNMAMQPRQTLNRPPTAPNQLRLQLQQRLQGQQQLIHQNRQAILNQFAANSPVG
INMRAGMQQQITPQPPLNAQMLAQRQRELYSQQHRQRQLMQQRAILMRQQSFGNNLPPSAGLPVQIGAARLPQA
PPQQFPYPPNYGTTPGNPPTSTSPFSPLASNPEAALANRSSMVNRGMMGGVGGQFGAGMTPQMQQNIFQYSGSG
MGQQNEPAFAPSLSPSSPLMSPQLPPSQSPMLQPAPPTPGYQSPDMKTWQQGAMGNSNVFSQAGQSQPAPSQQG
MYNNMSITVSMAGGNTNVQNMNPMAGQMQMNSLQMPGMNSMCSEQVNDPALRPAGLYCNQLSSTDLLKTEADGA
Q

>4Chlorocebus sabaeus|A0A0D9R7Z5|Mammalia|SRC1
MSGLGDSSSDPANPDSHKRKGSPCDTLASSTEKRRREQENKYLEELAELLSANISDIDSLSVKPDKCKILKKTV
DQIQLMKRMEQEKSTTDDDVQKSDISSSSQGVIEKESLGPLLLEALDGFFFVVNCEGRIVFVSENVTSYLGYNQ
EELMNTSVYSILHVGDHAEFVKNLLPKSLVNGVPWPQEATRRNSHTFNCRMLIHPPDEPGTENQEACQRYEVMQ
CFTVSQPKSIQEDGEDFQSCLICIARRLPRPPAITGVESFMTKQDTTGKIISIDTSSLRAAGRTGWEDLVRKCI
YAFFQPQGREPSYARQLFQEVMTRGTASSPSYRFILNDGTMLSAHTKCKLCYPQSPDMQPFIMGIHIIDREHSG
LSPQDDTNSGMSIPRVNPSVNPSISPAHGVARSSTLPPSNSNMVSTRINRQQSSDLHSGSHSNSSNSQGSFGCS
PGSQIVANVALNQGQTSSQSSNPSLNLNNSPMEGTGISLAQFMSPRRQVTSGLTTRPRMPNNSFPPNISTLSSP
VGMTSSACNNNTNRSYSNIPVTSLQGMNEGPNNSVGFSANSPVLRQMSSQNSPSRLNIQPAKAESKDNKEIASI
LNEMIQSDNSSSDGKPLDSGLLHNNDRLSDGDSKYSQTSHKLVQLLTTTAEQQLRHADIDTSCKDVLSCTGTSN
SASANSSGGSCPSSHSSLTERHKILHRLLQEGSPSDITTLSVEPDKKDSASTSVSVTGQVQGNSSIKLELDASK
KKESKDHQLLRYLLDKDEKDLRSTPNLSLDDVKVKVEKKEQMDPCNTNPTPMTKPTPEEIKLEAQSQFTADLDQ
FDQLLPTLEKAAQLPGLCETDRMDGAVTSVTIKSEILPASLQSATARPTSRLNRLPELELEAIDNQFGQPGTGD
QIPWTNNTVTAINQNKSEDQCISSQLDELLCPPTTVEGRNDEKALLEQLVSFLSGKDETELAELDRALGIDKLV
QGGGLDVLSERFPPQQATPPLMMEERPNLYSQPYSSPSPTANLPSPFQGMVRQKPSLGTMPVQVTPPRGAFSPG
MGMQPRQTLNRPPAAPNQLRLQLQQRLQGQQQLIHQNRQAILNQFAATAPVGINMRSGMQQQITPQPPLNAQML
AQRQRELYSQQHRQRQLIQQQRAMLMRQQSFGNNLPPSSGLPVQMGNPRLPQGAPQQFPYPPNYGTNPGTPPAS
TSPFSQLAANPEASLANRNSMVSRGMTGNMGGQFGTGINPQIQQNVFQYPGAGMVPQGEANFAPSLSPGSSMVP
MPIPPPQSSLLQQTPPASGYQSPDMKAWQQGAIGNNVFSQAVQNQPTPAQPGVYNNMSITVSMAGGNTNVQNMN
PMMAQMQMSSLQMPGMNTVCPEQINDPALRHTGLYCNQLSSTDLLKTEADGTQDKKTEEFFSVVTTD

>5Sus scrofa|A0A287AVZ1|Sarcopterygii|SRC1

MSGLGDSSSDPANPDSHKRKGSPCDTLASSTEKRRREQENKYLEELAELLSANISDIDSLSVKP
DKCKILKKTVDQIQLMKRMEQEKSTTDDEVQKSDISSSSQGVIEKESLGPLLLEALDGFFFVVN
CEGRIVFVSENVTSYLGYNQEELMNTSVYSILHVGDHAEFVKNLLPKSLVNGVPWPQEATRRNS
HTFNCRMLIHPPDEPGTENQEACQRYEVMQCFTVSQPKSIQEDGEDFQSCLICIARRLPRPPAI
MGVESFMTKQDTTGKIISIDTSSLRAAGRTGWEDLVRKCIYAFFQPQGREPSYARQLFQEVMTR
GTASSPSYRFILNDGTMLSAHTKCKLCYPQSPDMQPFIMGIHIIDREHSGLSPQDDTNSGMSIP
RVNPPVNPSISPAHGVARSSSLPPSNSNMVSTRVNRQQSSDLHSSSHSNSSNSQGSFGCSPGNQ

IVAGVALNQGQASSQSTNPPLNLNNSPMEGTGISLAQFMSPRRQVSSGLATRPRMPNNSFPPNI
PTLNSPVSMTSTACNNSNRSYSNIPVTSLQSMNEGPNNSVGFSAGSPVLRQMSSQNSPSRLNIQ
PAKAESKDNKEIASILNEMIQSDNSSNDGKPLDSGLLHNNDRLSDGDNKYSQTSHKLVQLLTTT
AEQQLRHADIDTSCKEVLSCTGTSSSASANSSSGSCPSSHSSLTERHKILHRLLQEGSPSDITT
LSVEPDKKDSASTSVSVTGQVPGNSGIKLELDASKKKESKDHQLLRYLLDKDEKDLRSTPNLSL
DDVKVKVEKKEQMDPCNTNPTPMTKPPPEEIKLESQSQFTADLDQFDQLLPTLEKAAQLPGLCE
TERMDGAVTSVTIKSEILPATLQSTTARPTSRLNRLPELELEAIDNQFGQPGTGDQIPWANNTV
TAVNQNKPEDQCISSQLDELLCPPTTVEGRNDEKALLEQLVSFLSGKDETELAELDRALGIDKL
VQGGGLDVLSERFPPQQATPPLMMEERPNLYSQPYSSPSPTANLSGPFQGMVRQKPSLGTMPVQ
VTPPRGAFSPGMGMQPRQTLNRPPAAPNQLRLQLQQRLQGQQQLIHQNRQAILNQFAANAPVGI
NMRSGMQQQITPQPPLNAQMLAQRQRELYSQQHRQRQLIQQQRAMLMRQQSFGNNLPPSSGLPV
PMGNPRLPQGAPQQFPYPPNYGTNPGTPPASTSPFSQLAGNPEATLGNRNSMVNRGMTGNMGGQ
FGTGINPQMQQNVFQYPGSGMVPQGEANFAPSLSPGSSMVPMPIPPPQSSLLQQTPPASGYQSP
DMKAWQQGAMGNNNVFSQAVQNQPTPAQPGVYNNMSITVSMAGGNTNVQNMNPMMGQMQMSSLQ
MPGMNTVCPEQINDPALRHTGLYCNQLSSTDLLKTEADGTQVQQVQVFADVQCTVNLVGGDPYL
NQPGPLGTQKPTAGPQTPQAQQKSLLQQLLTE

>6Sus scrofa|A0A287AWE8|Sarcopterygii|SRC1
MSGLGDSSSDPANPDSHKRKGSPCDTLASSTEKRRREQENKYLEELAELLSANISDIDSL
SVKPDKCKILKKTVDQIQLMKRMEQEKSTTDDEVQKSDISSSSQGVIEKESLGPLLLEAL
DGFFFVVNCEGRIVFVSENVTSYLGYNQEELMNTSVYSILHVGDHAEFVKNLLPKSLVNG
VPWPQEATRRNSHTFNCRMLIHPPDEPGTENQEACQRYEVMQCFTVSQPKSIQEDGEDFQ
SCLICIARRLPRPPAIMGVESFMTKQDTTGKIISIDTSSLRAAGRTGWEDLVRKCIYAFF
QPQGREPSYARQLFQEVMTRGTASSPSYRFILNDGTMLSAHTKCKLCYPQSPDMQPFIMG
IHIIDREHSGLSPQDDTNSGMSIPRVNPPVNPSISPAHGVARSSSLPPSNSNMVSTRVNR
QQSSDLHSSSHSNSSNSQGSFGCSPGNQIVAGVALNQGQASSQSTNPPLNLNNSPMEGTG
ISLAQFMSPRRQVSSGLATRPRMPNNSFPPNIPTLNSPVSMTSTACNNSNRSYSNIPVTS
LQSMNEGPNNSVGFSAGSPVLRQMSSQNSPSRLNIQPAKAESKDNKEIASILNEMIQSDN
SSNDGKPLDSGLLHNNDRLSDGDNKYSQTSHKLVQLLTTTAEQQLRHADIDTSCKEVLSC
TGTSSSASANSSSGSCPSSHSSLTERHKILHRLLQEGSPSDITTLSVEPDKKDSASTSVS
VTGQVPGNSGIKLELDASKKKESKDHQLLRYLLDKDEKDLRSTPNLSLDDVKVKVEKKEQ
MDPCNTNPTPMTKPPPEEIKLESQSQFTADLDQFDQLLPTLEKAAQLPGLCETERMDGAV
TSVTIKSEILPATLQSTTARPTSRLNRLPELELEAIDNQFGQPGTGDQIPWANNTVTAVN
QNKPEDQCISSQLDELLCPPTTVEGRNDEKALLEQLVSFLSGKDETELAELDRALGIDKL
VQGGGLDVLSERFPPQQATPPLMMEERPNLYSQPYSSPSPTANLSGPFQGMVRQKPSLGT
MPVQVTPPRGAFSPGMGMQPRQTLNRPPAAPNQLRLQLQQRLQGQQQPPLNAQMLAQRQR
ELYSQQHRQRQLIQQQRAMLMRQQSFGNNLPPSSGLPVPMGNPRLPQGAPQQFPYPPNYG
TNPGTPPASTSPFSQLAGNPEATLGNRNSMVNRGMTGNMGGQFGTGINPQMQQNVFQYPG
SGMVPQGEANFAPSLSPGSSMVPMPIPPPQSSLLQQTPPASGYQSPDMKAWQQGAMGNNN
VFSQAVQNQPTPAQPGVYNNMSITVSMAGGNTNVQNMNPMMGQMQMSSLQMPGMNTVCPE
QINDPALRHTGLYCNQLSSTDLLKTEADGTQQVQQVQVFADVQCTVNLVGGDPYLNQPGP
LGTQKPTAGPQTPQAQQKSLLQQLLTE
>7Ictidomys tridecemlineatus|A0A287DDP9|Sarcopterygii|SRC1
MSGLGDSSSDPANPDSHKRKGSPCDTLASSTEKRRREQENKYLEELAELLSANISDIDSLSVKPDKCKILKKTV
DQIQLMKRMEQEKSTTDDDVQKSDISSSSQGVIEKESLGPLLLEALDGFFFVVNCEGRIVFVSENVTSYLGYNQ
EELMNTSVYSILHVGDHAEFVKNLLPKSLVNGVPWPQEATRRNSHTFNCRMLIHPPDEPGTENQEACQRYEVMQ
CFTVSQPKSIQEDGEDFQSCLICIARRLPRPPAITGVESFMTKQDTTGKIISIDTSSLRAAGRTGWEDLVRKCI
YAFFQPQGREPSYARQLFQEVMTRGTASSPSYRFILNDGTMLSAHTKCKLCYPQSPDMQPFIMGIHIIDREHSG
LSPQDDTNSGMSIPRVNPSVNPSISPAHGVARSSTLPPSNSSMVSTRVNRHQSSDLHSSSSHSNSSNNQGSFGC

SPGNQIVANVALNQGQASSQSSNPSLNLNNSPMEGTGISLAQFMSPRRQVTSGLATRARMPNNSFPPNIPTLNS
PVGMTSSACNNSNRSYSNIPVTSLQGMNEGPNNSVGFSASSPVLRQMSSQNSPSRLNIQPAKAESKDNKEIASI
LNEMIQSDNSASDSKPLDSGLLHNNDRLSDGDSKYSQTSHKLVQLLTTTAEQQLRHADIDTSCKEVLSCTGTSS
SASANSSGGTCPSSHSSLTERHKILHRLLQEGSPSDITTLSVEPDKKDSASTSVSVTGQAQGNSNIKLELDTSK
KKESKDHQLLRYLLDKDEKDLRSTPNLSLDDVKVKVEKKDQMDPCNTNPTPMTKPAPEEIKLESQSQFTADLEQ
FDQLLPTLEKAAQLPGLCETDRMDATVTSVTVKSEILPASLQSTTARPTSRLNRLPELELEAIDNQFGQPGTGD
QIPWANNAVTAINQNKAEDQCISSQLDELLCPPTTVEGRNDEKALLEQLVSFLSGKDETELAELDRALGIDKLV
QGGGLDVLSERFPPQQATPPLMMEERPNLYSQPYSSPSPTANLPSPFQGMVRQKPSLGTMPVQVTPPRGAFSPG
MGMQPRQTLNRPPAAPNQLRLQLQQRLQGQQQLLHQNRHAILNQFAATAPVGINMRSGMQQQITPQPPLNAQML
AQRQRELYSQQHRQRQLIQQQRAMLMRQQSFGNNLPPSSGLPVQMGNPRLPQGAPQQFPYPPNYGTNPGTPPAS
TSPFSQLAANPDAALATRNSMVNRGMAGNMGGQFGTGINPQMQQNVFQYPGSGMVPQGEANFAPSLSPGSSMVP
MPIPPPQSSLLQQTPPASGYQSPDMKAWQQGAIGNNNVFNQAVQNQPTPAQPGVYNNMSITVSMAGGNTNVQNM
NPMMGQMQMSSLQMPGMNAVCPEQINDPALRHTGLYCNQLSSTDLLKTEADGTQQVQQVQVFADVQCTVNLVGG
DPYLNQPGPLGTQKPTSGPQTPQAQQKSLLQQLLTE
>8Nomascus leucogenys|A0A2I3HVL7|Mammalia|SRC1
MSGLGDSSSDPANPDSHKRKGSPCDTLASSTEKRRREQENKYLEELAELLSANISDIDSLSVKPDKCKILKKTV
DQIQLMKRMEQEKSTTDDDVQKSDISSSSQGVIEKESLGPLLLEALDGFFFVVNCEGRIVFVSENVTSYLGYNQ
EELMNTSVYSILHVGDHAEFVKNLLPKSLVNGVPWPQEATRRNSHTFNCRMLIHPPDEPGTENQEACQRYEVMQ
CFTVSQPKSIQEDGEDFQSCLICIARRLPRPPAITGVESFMTKQDTTGKIISIDTSSLRAAGRTGWEDLVRKCI
YAFFQPQGREPSYARQLFQEVMTRGTASSPSYRFILNDGTMLSAHTKCKLCYPQSPDMQPFIMGIHIIDREHSG
LSPQDDTNSGMSIPRVNPSVNPSISPAHGVARSSTLPPSNSNMVSTRINRQQSSDLHNSSHSNSSNSQGSFGCS
PGSQIVASVALNQGQASSQSSNPSLNLNNSPMEGTGISLAQFMSPRRQVTSGLATRPRMPNNSFPPNISTLSSP
VGMTSSACNNNNRSYSNIPVTSLQGMNEGPNNSVGFSASSPVLRQMSSQNSPSRLNIQPAKAESKDNKEIASIL
NEMIQSDNSSSDGKPLDSGLLHNNDRLSDGDSKYSQTSHKLVQLLTTTAEQQLRHADIDTSCKDVLSCTGTSSS
ASANSSGGSCPSSHSSLTERHKILHRLLQEGSPSDITTLSVEPDKKDSASTSVSVTGQVQGNSSIKLELDASKK
KESKDHQLLRYLLDKDEKDLRSTPNLSLDDVKVKVEKKEQMDPCNTNPTPMTKPTPEEIKLEAQSQFTADLDQF
DQLLPTLEKAAQLPGLCETDRMDGAVTSVTIKSEILPASLQSATARPTSRLNRLPELELEAIDNQFGQPGTGDQ
IPWTNNTVTAINQSKSEDQCISSQLDELLCPPTTVEGRNDEKALLEQLVSFLSGKDETELAELDRALGIDKLVQ
GGGLDVLSERFPPQQATPPLMMEERPNLYSQPYSSPSPTANLPSPFQGMVRQKPSLGTMPVQVTPPRGAFSPGM
GMQPRQTLNRPPAAPNQLRLQLQQRLQGQQQLIHQNRQAILNQFAATAPVGINMRSGMQQQITPQPPLNAQMLA
QRQRELYSQQHRQRQLIQQQRAMLMRQQSFGNNLPPSSGLPVQMGNPRLPQGAPQQFPYPPNYGTNPGTPPAST
SPFSQLTANPEASLANRNSMVSRGMTGNMGGQFGTGINPQMQQNVFQYPGAGMVPQGEANFAPSLSPGSSMVPM
PIPPPQSSLLQQTPPASGYQSPDMKAWQQGAIGNNNVFSQAVQNQPTPAQPGVYNNMSITVSMAGGNTNVQNMN
PMMAQMQMSSLQMPGMNTVCPEQINDPALRHTGLYCNQLSSTDLLKTEADGTQQVQQVQVFADVQCTVNLVGGD
PYLNQPGPLGTQKPTSGPQTPQAQQKSLLQQLLTE
>9Papio anubis|A0A2I3N6H6|Sarcopterygii|SRC1
MSGLGDSSSDPANPDSHKRKGSPCDTLASSTEKRRREQENKYLEELAELLSANISDIDSLSVKPDKCKILKKTV
DQIQLMKRMEQEKSTTDDDVQKSDISSSSQGVIEKESLGPLLLEALDGFFFVVNCEGRIVFVSENVTSYLGYNQ
EELMNTSVYSILHVGDHAEFVKNLLPKSLVNGVPWPQEATRRNSHTFNCRMLIHPPDEPGTENQEACQRYEVMQ
CFTVSQPKSIQEDGEDFQSCLICIARRLPRPPAITGVESFMTKQDTTGKIISIDTSSLRAAGRTGWEDLVRKCI
YAFFQPQGREPSYARQLFQEVMTRGTASSPSYRFILNDGTMLSAHTKCKLCYPQSPDMQPFIMGIHIIDREHSG
LSPQDDTNSGMSIPRVNPSVNPSISPAHGVARSSTLPPSNSNMVSTRINRQQSSDLHSGSHSNSSNSQGSFGCS
PGSQIVANVALNQGQASSQSSNPSLNLNNSPMEGTGISLAQFMSPRRQVTSGLTTRPRMPNNSFPPNISTLSSP
VGMTSSACNNNTNRSYSNIPVTSLQGMNEGPNNSVGFSANSPVLRQMSSQNSPSRLNIQPAKAESKDNKEIASI
LNEMIQSDNSSSDGKPLDSGLLHNNDRLSDGDSKYSQTSHKLVQLLTTTAEQQLRHADIDTSCKDVLSCTGTSN
SASANSSGGSCPSSHSSLTERHKILHRLLQEGSPSDITTLSVEPDKKDSASTSVSVTGQVQGNSSIKLELDASK
KKESKDHQLLRYLLDKDEKDLRSTPNLSLDDVKVKVEKKEQMDPCNTNPTPMTKPTPEEIKLEAQSQFTADLDQ
FDQLLPTLEKAAQLPGLCETDRMDGAVTSVTIKSEILPASLQSATARPTSRLNRLPELELEAIDNQFGQPGTGD
QIPWTNNTVTAINQNKSEDQCISSQLDELLCPPTTVEGRNDEKALLEQLVSFLSGKDETELAELDRALGIDKLV
QGGGLDVLSERFPPQQATPPLMMEERPNLYSQPYSSPSPTANLPSPFQGMVRQKPSLGTMPVQVTPPRGAFSPG
MGMQPRQTLNRPPAAPNQLRLQLQQRLQGQQQLIHQNRQAILNQFAATAPVGINMRSGMQQQITPQPPLNAQML
AQRQRELYSQQHRQRQLIQQQRAMLMRQQSFGNNLPPSSGLPVQMGNPRLPQGAPQQFPYPPNYGTNPGTPPAS
TSPFSQLAANPEASLANRNSMVSRGMTGNMGGQFGTGINPQIQQNVFQYPGAGMVPQGEASFAPSLSPGSSMVP

MPIPPPQSSLLQQTPPASGYQSPDMKAWQQGAIGNNVFSQAVQNQPTPAQPGVYNNMSITVSMAGGNTNVQNMN
PMMAQMQMSSLQMPGMNTVCPEQINDPALRHTGLYCNQLSSTDLLKTEADGTQDKKTEEFFSVVTTD
>10Pan troglodytes|A0A2J8NLB6|Sarcopterygii|SRC1
MSGLGDSSSDPANPDSHKRKGSPCDTLASSTEKRRREQENKYLEELAELLSANISDIDSLSVKPDKCKILKKTV
DQIQLMKRMEQEKSTTDDDVQKSDISSSSQGVIEKESLGPLLLEALDGFFFVVNCEGRIVFVSENVTSYLGYNQ
EELMNTSVYSILHVGDHAEFVKNLLPKSLVNGVPWPQEATRRNSHTFNCRMLIHPPDEPGTENQEACQRYEVMQ
CFTVSQPKSIQEDGEDFQSCLICIARRLPRPPAITGVESFMTKQDTTGKIISIDTSSLRAAGRTGWEDLVRKCI
YAFFQPQGREPSYARQLFQEVMTRGTASSPSYRFILNDGTMLSAHTKCKLCYPQSPDMQPFIMGIHIIDREHSG
LSPQDDTNSGMSIPRVNPSVNPSISPAHGVARSSTLPPSNSNMVSTRINRQQSSDLHSSSHSNSSSNSQGSFGCS
PGSQIVANVALNQGQASSQSSNPSLNLNNSPMEGTGISLAQFMSPRRQVTSGLATRPRMPNNSFPPNISTLSSP
VGMTSSACNNNNRSYSNIPVTSLQGMNEGPNNSVGFSASSPVLRQMSSQNSPSRLNIQPAKAESKDNKEIASIL
NEMIQSDNSSSDGKPLDSGLLHNNDRLSDGDSKYSQTSHKLVQLLTTTAEQQLRHADIDTSCKDVLSCTGTSNS
ASANSSGGSCPSSHSSLTERHKILHRLLQEGSPSDITTLSVEPDKKDSASTSVSVTGQVQGNSSIKLELDASKK
KESKDHQLLRYLLDKDEKDLRSTPNLSLDDVKVKVEKKEQMDPCNTNPTPMTKPTPEEIKLEAQSQFTADLDQF
DQLLPTLEKAAQLPGLCETDRMDGAVTSVTIKSEILPASLQSATARPTSRLNRLPELELEAIDNQFGQPGTGDQ
IPWTNNTVTAINQSKSEDQCISSQLDELLCPPTTVEGRNDEKALLEQLVSFLSGKDETELAELDRALGIDKLVQ
GGGLDVLSERFPPQQATPPLIMEERPNLYSQPYSSPSPTANLPSPFQGMVRQKPSLGTMPVQVTPPRGAFSPGM
GMQPRQTLNRPPAAPNQLRLQLQQRLQGQQQLIHQNRQAILNQFAATAPVGINMRSGMQQQITPQPPLNAQMLA
QRQRELYSQQHRQRQLIQQQRAMLMRQQSFGNNLPPSSGLPVQMGNPRLPQGAPQQFPYPPNYGTNPGTPPAST
SPFSQLAANPEASLANRNSMVSRGMTGNMGGQFGTGINPQMQQNVFQYPGAGMVPQGEANFAPSLSPGSSMVPM
PIPPPQSSLLQQTPPASGYQSPDMKAWQQGAIGNNNVFSQAVQNQPTPAQPGVYNNMSITVSMAGGNTNVQNMN
PMMAQMQMSSLQMPGMNTVCPEQINDPALRHTGLYCNQLSSTDLLKTEADGTQVQQVQVFADVQCTVNLVGGDP
YLNQPGPLGTQKPTSGPQTPQAQQKSLLQQLLTE

>11Pan troglodytes|A0A2J8NLF0|Sarcopterygii|SRC1
MFLSRPGFGPLPGIQPKGPRKGSKHVDEAIGRGVEQAGVLVEKVTFFLRRRKLPSGTQDLTISVLRIHYEQRSL
PGVKFFNMSGLGDSSSDPANPDSHKRKGSPCDTLASSTEKRRREQENKYLEELAELLSANISDIDSLSVKPDKC
KILKKTVDQIQLMKRMEQEKSTTDDDVQKSDISSSSQGVIEKESLGPLLLEALDGFFFVVNCEGRIVFVSENVT
SYLGYNQEELMNTSVYSILHVGDHAEFVKNLLPKSLVNGVPWPQEATRRNSHTFNCRMLIHPPDEPGTENQEAC
QRYEVMQCFTVSQPKSIQEDGEDFQSCLICIARRLPRPPAITGVESFMTKQDTTGKIISIDTSSLRAAGRTGWE
DLVRKCIYAFFQPQGREPSYARQLFQEVMTRGTASSPSYRFILNDGTMLSAHTKCKLCYPQSPDMQPFIMGIHI
IDREHSGLSPQDDTNSGMSIPRVNPSVNPSISPAHGVARSSTLPPSNSNMVSTRINRQQSSDLHSSSHSNSSNS
QGSFGCSPGSQIVANVALNQGQASSQSSNPSLNLNNSPMEGTGISLAQFMSPRRQVTSGLATRPRMPNNSFPPN
ISTLSSPVGMTSSACNNNNRSYSNIPVTSLQGMNEGPNNSVGFSASSPVLRQMSSQNSPSRLNIQPAKAESKDN
KEIASILNEMIQSDNSSSDGKPLDSGLLHNNDRLSDGDSKYSQTSHKLVQLLTTTAEQQLRHADIDTSCKDVLS
CTGTSNSASANSSGGSCPSSHSSLTERHKILHRLLQEGSPSDITTLSVEPDKKDSASTSVSVTGQVQGNSSIKL
ELDASKKKESKDHQLLRYLLDKDEKDLRSTPNLSLDDVKVKVEKKEQMDPCNTNPTPMTKPTPEEIKLEAQSQF
TADLDQFDQLLPTLEKAAQLPGLCETDRMDGAVTSVTIKSEILPASLQSATARPTSRLNRLPELELEAIDNQFG
QPGTGDQIPWTNNTVTAINQSKSEDQCISSQLDELLCPPTTVEGRNDEKALLEQLVSFLSGKDETELAELDRAL
GIDKLVQGGGLDVLSERFPPQQATPPLIMEERPNLYSQPYSSPSPTANLPSPFQGMVRQKPSLGTMPVQVTPPR
GAFSPGMGMQPRQTLNRPPAAPNQLRLQLQQRLQGQQQLIHQNRQAILNQFAATAPVGINMRSGMQQQITPQPP
LNAQMLAQRQRELYSQQHRQRQLIQQQRAMLMRQQSFGNNLPPSSGLPVQMGNPRLPQGAPQQFPYPPNYGTNP
GTPPASTSPFSQLAANPEASLANRNSMVSRGMTGNMGGQFGTGINPQMQQNVFQYPGAGMVPQGEANFAPSLSP
GSSMVPMPIPPPQSSLLQQTPPASGYQSPDMKAWQQGAIGNNNVFSQAVQNQPTPAQPGVYNNMSITVSMAGGN
TNVQNMNPMMAQMQMSSLQMPGMNTVCPEQINDPALRHTGLYCNQLSSTDLLKTEADGTQVSATGSGVC
>12Pongo abelii|A0A2J8VMG9|Mammalia|SRC1
MSGLGDSSSDPANPDSHKRKGSPCDTLASSTEKRRREQENKYLEELAELLSANISDIDSLSVKPDKCKILKKTV
DQIQLMKRMEQEKSTTDDDVQKSDISSSSQGVIEKESLGPLLLEALDGFFFVVNCEGRIVFVSENVTSYLGYNQ
EELMNTSVYSILHVGDHAEFVKNLLPKSLVNGVPWPQEATRRNSHTFNCRMLIHPPDEPGTENQEAGQRYEVMQ
CFTVSQPKSIQEDGEDFQSCLICIARRLPRPPAITGVESFMTKQDTTGKIISIDTSSLRAAGRTGWEDLVRKCI
YAFFQPQGREPSYARQLFQEVMTRGTASSPSYRFILNDGTMLSAHTKCKLCYPQSPDMQPFIMGIHIIDREHSG
LSPQDDTNSGMSIPRVNPSVNPSISPAHGVARSSTLPPSNSNMVSTRINRQQSSDLHSSSHSNSSSNSQGSFGCS
PGSQIVANVALNQGQASSQSSNPSLNLNNSPMEGTGISLAQFMSPRRQVTSGLATRPRMPNNSFPPNISTLSSP
VGMTSSTCNNNNRSYSNIPVTSLQGMNEGPNNSVGFSASSPVLRQMSSQNSPSRLNIQPAKAESKDNKEIASIL

NEMIQSDNSSSDGKPLDSGLLHNNDRLSDGDSKYSQTSHKLVQLLTTTAEQQLRHADIDTSCKDVLSCTGTSNS
ASANSSGGSCPSSHSSLTERHKILHRLLQEGSPSDITTLSVEPDKKDSASTSVSVTGQVQGNSSIKLELDASKK
KESKDHQLLRYLLDKDEKDLRSTPNLSLDDVKVKVEKKEQMDPCNTNPTPMTKPTPEEIKLEAQSQFTADLDQF
DQLLPTLEKAAQLPGLCETDRMDGAVTSVTIKSEILPASLQSATARPTSRLNRLPELELEAIDNQFGQPGTGDQ
IPWTNNTVTAINQSKSEDQCISSQLDELLCPPTTVEGRNDEKALLEQLVSFLSGKDETELAELDRALGIDKLVQ
GGGLDVLSERFPPQQATPPLMMEERPNLYSQPYSSPSPTANLPSPFQGMVRQKPSLGTMPVQVTPPRGAFSAGM
GMQPRQTLNRPPAAPNQLRLQLQQRLQGQQQLIHQNRQAILNQFAATAPVGINMRSGMQQQITPQPPLNAQMLA
QRQRELYSQQHRQRQLIQQQRAMLMRQQSFGNNLPPSSGLPVQMGNPRLPQGAPQQFPYPPNYGTNPGTPPAST
SPFSQLAANPEASLASRNSMVSRGMTGNMGGQFGTGINPQMQQNVFQYPGAGMVPQGEANFAPSLSPGSSMVPM
PIPPPQSSLLQQTPPASGYQSPDMKAWQQGAIGNNNVFSQAVQNQPTPAQPGVYNNMSITVSMAGGNTNVQNMN
PMMAQMQMSSLQMPGMNTVCPEQINDPALRHTGLYCNQLSSTDLLKTEADGTQQVQQVQVFADVQCTVNLVGGD
PYLNQPGPLGTQKPTSGPQTPQAQQKSLLQQLLTE
>13Aotus nancymaae|A0A2K5CLJ9|Mammalia|SRC1
MSGLGDSSSDPANPDSHKRKGSPCDTLASSTEKRRREQENKYLEELAELLSANISDIDSLSVKPDKCKILKKTV
DQIQLMKRMEQEKSTTDDDVQKSDISSSSQGVIEKESLGPLLLEALDGFFFVVNCEGRIVFVSENVTSYLGYNQ
EELMNTSVYSILHVGDHAEFVKNLLPKSLVNGVPWPQEATRRNSHTFNCRMLIHPPDEPGTENQEACQRYEVMQ
CFTVSQPKSIQEDGEDFQSCLICIARRLPRPPAITGVESFMTKQDTTGKIISIDTSSLRAAGRTGWEDLVRKCI
YAFFQPQGREPSYARQLFQEVMTRGTASSPSYRFILNDGTMLSAHTKCKLCYPQSPDMQPFIMGIHIIDREHSG
LSPQDDTNSGMSIPRVNPSVNPNISPAHGVARSSTLPPSNSNMVSTRINRQQSSDLHSSSHSNSSNSQGSFGCS
PGSQIVANVALNQGQASSQSSNPSLNLNNSPMEGTGISLAQFISPRRQVPSGLATRPRMPNNSFPPNISTLSSP
VGMTSSACNNNNRSYSNIPVTSLQGMNEGPNNSVGFSASSPVLRQMSSQNSPSRLNIQPAKAESKDNKEIASIL
NEMIQSDNSSSDGKPLDSGLLHNSDRLSDGDSKYSQTSHKLVQLLTTTAEQQLRHADIDTSCKDVLSCTGTSNS
ASANSSGGSCPSSHSSLTERHKILHRLLQEGSPSDITTLSVEPDKKDSASTSVSVTGQVQGNSSVKLELDASKK
KESKDHQLLRYLLDKDEKDLRSTPNLSLDDVKVKVEKKEQMDPCNTNPTPMTKPAPEEIKLEAQSQFTADLDQF
DQLLPTLEKAAQLPGLCETDRMDGAVTSVTIKSEILPASLQSATARPTSRLNRLPELELEAIDNQFGQPGTGDQ
IPWTNNTVTAVNQNKSEDQCISSQLDELLCPPTTVEGRNDEKALLEQLVSFLSGKDETELAELDRALGIDKLVQ
GGGLDVLSERFPPQQATPPLMMEERPSLYSQPYSSPSPTANLPSPFQGMVRQKPSLGTMPVQVTPPRGAFSPGM
GMQPRQTLNRPPAAPNQLRLQLQQRLQGQQQLIHQNRQAILNQFAATAPVGINMRSGMQQQITPQPPLNAQMLA
QRQRELYSQQHRQRQLIQQQRAMLMRQQSFGNNLPPSSGLPVQMGNPRLPQGAPQQFPYPPNYGTNPGTPPAST
SPFSQLAANPEASLANRSSMVSRGMTGNMGGQFGTGINPQMQQNVFQYPGAGMVPQGEANFAPSLSPGSSMVPM
PIPPPQSSLLQQTPPASGYQSPDMKAWQQGAIGNNNVFSQAVQNQPTPAQPGVYNNMSITVSMAGGNTNVQNMN
PIMGQMQMSSLQMPGMNTVCPEQINDPALRHTGLYCNQLSSTDLLKTEADGTQQVQQVQVFADVQCTVNLVGGD
PYLNQPGPLGTQKPTSGPQTPQAQQKSLLQQLLTE

>14Aotus nancymaae|A0A2K5CLN4|Mammalia|SRC1
MSGLGDSSSDPANPDSHKRKGSPCDTLASSTEKRRREQENKYLEELAELLSANISDIDSLSVKPDKCKILKKTV
DQIQLMKRMEQEKSTTDDDVQKSDISSSSQGVIEKESLGPLLLEALDGFFFVVNCEGRIVFVSENVTSYLGYNQ
EELMNTSVYSILHVGDHAEFVKNLLPKSLVNGVPWPQEATRRNSHTFNCRMLIHPPDEPGTENQEACQRYEVMQ
CFTVSQPKSIQEDGEDFQSCLICIARRLPRPPAITGVESFMTKQDTTGKIISIDTSSLRAAGRTGWEDLVRKCI
YAFFQPQGREPSYARQLFQEVMTRGTASSPSYRFILNDGTMLSAHTKCKLCYPQSPDMQPFIMGIHIIDREHSG
LSPQDDTNSGMSIPRVNPSVNPNISPAHGVARSSTLPPSNSNMVSTRINRQQSSDLHSSSHSNSSNSQGSFGCS
PGSQIVANVALNQGQASSQSSNPSLNLNNSPMEGTGISLAQFISPRRQVPSGLATRPRMPNNSFPPNISTLSSP
VGMTSSACNNNNRSYSNIPVTSLQGMNEGPNNSVGFSASSPVLRQMSSQNSPSRLNIQPAKAESKDNKEIASIL
NEMIQSDNSSSDGKPLDSGLLHNSDRLSDGDSKYSQTSHKLVQLLTTTAEQQLRHADIDTSCKDVLSCTGTSNS
ASANSSGGSCPSSHSSLTERHKILHRLLQEGSPSDITTLSVEPDKKDSASTSVSVTGQVQGNSSVKLELDASKK
KESKDHQLLRYLLDKDEKDLRSTPNLSLDDVKVKVEKKEQMDPCNTNPTPMTKPAPEEIKLEAQSQFTADLDQF
DQLLPTLEKAAQLPGLCETDRMDGAVTSVTIKSEILPASLQSATARPTSRLNRLPELELEAIDNQFGQPGTGDQ
IPWTNNTVTAVNQNKSEDQCISSQLDELLCPPTTVEGRNDEKALLEQLVSFLSGKDETELAELDRALGIDKLVQ
GGGLDVLSERFPPQQATPPLMMEERPSLYSQPYSSPSPTANLPSPFQGMVRQKPSLGTMPVQVTPPRGAFSPGM
GMQPRQTLNRPPAAPNQLRLQLQQRLQGQQQLIHQNRQAILNQFAATAPVGINMRSGMQQQITPQPPLNAQMLA
QRQRELYSQQHRQRQLIQQQRAMLMRQQSFGNNLPPSSGLPVQMGNPRLPQGAPQQFPYPPNYGTNPGTPPAST
SPFSQLAANPEASLANRSSMVSRGMTGNMGGQFGTGINPQMQQNVFQYPGAGMVPQGEANFAPSLSPGSSMVPM
PIPPPQSSLLQQTPPASGYQSPDMKAWQQGAIGNNNVFSQAVQNQPTPAQPGVYNNMSITVSMAGGNTNVQNMN
PIMGQMQMSSLQMPGMNTVCPEQINDPALRHTGLYCNQLSSTDLLKTEADGTQDKKTEEFFSVVTTD

>15Colobus angolensis palliatus|Mammalia|SRC1
MSGLGDSSSDPANPDSHKRKGSPCDTLASSTEKRRREQENKYLEELAELLSANISDIDSLSVKPDKCKILKKTV
DQIQLMKRMEQEKSTTDDDVQKSDISSSSQGVIEKESLGPLLLEALDGFFFVVNCEGRIVFVSENVTSYLGYNQ
EELMNTSVYSILHVGDHAEFVKNLLPKSLVNGVPWPQEATRRNSHTFNCRMLIHPPDEPGTENQEACQRYEVMQ
CFTVSQPKSIQEDGEDFQSCLICIARRLPRPPAITGVESFMTKQDTTGKIISIDTSSLRAAGRTGWEDLVRKCI
YAFFQPQGREPSYARQLFQEVMTRGTASSPSYRFILNDGTMLSAHTKCKLCYPQSPDMQPFIMGIHIIDREHSG
LSPQDDTNSGMSIPRVNPSVNPSISPAHGVARSSTLPPSNSNMVSTRINRQQSSDLHSGSHSNSSNSQGSFGCS
PGSQIVANVALNQGQASSQSSNPSLNLNNSPMEGTGISLAQFMSPRRQVTSGLTTRPRMPNNSFPPNISTLSSP
IGMTSSACNNNTNRSYSNIPVTSLQGMNEGPNNSVGFSASSPVLRQMSSQNSPSRLNIQPAKAESKDNKEIASI
LNEMIQSDNSSSDSKPLDSGLLHNNDRLSDGDSKYSQTSHKLVQLLTTTAEQQLRHADIDTSCKDVLSCTGTSN
SASANSSGGSCPSSHSSLTERHKILHRLLQEGSPSDITTLSVEPDKKDSASTSVSVTGQVQGNSSIKLELDASK
KKESKDHQLLRYLLDKDEKDLRSTPNLSLDDVKVKVEKKEQMDPCNTNPTPMTKPTPEEIKLEAQSQFTADLDQ
FDQLLPTLEKAAQLPGLCETDRMDGAVTSVTIKSEILPASLQSATARPTSRLNRLPELELEAIDNQFGQPGTGD
QIPWTNNTVTAINQNKSEDQCISSQLDELLCPPTTVEGRNDEKALLEQLVSFLSGKDETELAELDRALGIDKLV
QGGGLDVLSERFPPQQATPPLMMEERPNLYSQPYSSPSPTANLPSPFQGIVRQKPSLGTMPVQVTPPRGAFSPG
MGMQPRQTLNRPPAAPNQLRLQLQQRLQGQQQLIHQNRQAILNQFAATAPVGINMRSGMQQQITPQPPLNAQML
AQRQRELYSQQHRQRQLIQQQRAMLMRQQSFGNNLPPSSGLPVQMGNPRLPQGAPQQFPYPPNYGTNPGTPPAS
TSPFSQLAANPEASLANRNSMVSRGMTGNMGGQFGTGINPQIQQNVFQYPGAGMVPQGEANFAPSLSPGSSMVP
MPIPPPQSSLLQQTPPASGYQSPDMKAWQQGAIGNNVFSQAVQNQPTPAQPGVYNNMSITVSMAGGNTNVQNMN
PMMAQMQMSSLQMPGMNTVCPEQINDPALRHTGLYCNQLSSTDLLKTEADGTQQVQQVQVFADVQCTVNLVGGD
PYLNQPGPLGTQKPTSGPQTPQAQQKSLLQQLLTE

>16Colobus angolensis palliatus|A0A2K5IHR6|Mammalia|SRC1
MSGLGDSSSDPANPDSHKRKGSPCDTLASSTEKRRREQENKYLEELAELLSANISDIDSLSVKPDKCKILKKTV
DQIQLMKRMEQEKSTTDDDVQKSDISSSSQGVIEKESLGPLLLEALDGFFFVVNCEGRIVFVSENVTSYLGYNQ
EELMNTSVYSILHVGDHAEFVKNLLPKSLVNGVPWPQEATRRNSHTFNCRMLIHPPDEPGTENQEACQRYEVMQ
CFTVSQPKSIQEDGEDFQSCLICIARRLPRPPAITGVESFMTKQDTTGKIISIDTSSLRAAGRTGWEDLVRKCI
YAFFQPQGREPSYARQLFQEVMTRGTASSPSYRFILNDGTMLSAHTKCKLCYPQSPDMQPFIMGIHIIDREHSG
LSPQDDTNSGMSIPRVNPSVNPSISPAHGVARSSTLPPSNSNMVSTRINRQQSSDLHSGSHSNSSNSQGSFGCS
PGSQIVANVALNQGQASSQSSNPSLNLNNSPMEGTGISLAQFMSPRRQVTSGLTTRPRMPNNSFPPNISTLSSP
IGMTSSACNNNTNRSYSNIPVTSLQGMNEGPNNSVGFSASSPVLRQMSSQNSPSRLNIQPAKAESKDNKEIASI
LNEMIQSDNSSSDSKPLDSGLLHNNDRLSDGDSKYSQTSHKLVQLLTTTAEQQLRHADIDTSCKDVLSCTGTSN
SASANSSGGSCPSSHSSLTERHKILHRLLQEGSPSDITTLSVEPDKKDSASTSVSVTGQVQGNSSIKLELDASK
KKESKDHQLLRYLLDKDEKDLRSTPNLSLDDVKVKVEKKEQMDPCNTNPTPMTKPTPEEIKLEAQSQFTADLDQ
FDQLLPTLEKAAQLPGLCETDRMDGAVTSVTIKSEILPASLQSATARPTSRLNRLPELELEAIDNQFGQPGTGD
QIPWTNNTVTAINQNKSEDQCISSQLDELLCPPTTVEGRNDEKALLEQLVSFLSGKDETELAELDRALGIDKLV
QGGGLDVLSERFPPQQATPPLMMEERPNLYSQPYSSPSPTANLPSPFQGIVRQKPSLGTMPVQVTPPRGAFSPG
MGMQPRQTLNRPPAAPNQLRLQLQQRLQGQQQLIHQNRQAILNQFAATAPVGINMRSGMQQQITPQPPLNAQML
AQRQRELYSQQHRQRQLIQQQRAMLMRQQSFGNNLPPSSGLPVQMGNPRLPQGAPQQFPYPPNYGTNPGTPPAS
TSPFSQLAANPEASLANRNSMVSRGMTGNMGGQFGTGINPQIQQNVFQYPGAGMVPQGEANFAPSLSPGSSMVP
MPIPPPQSSLLQQTPPASGYQSPDMKAWQQGAIGNNVFSQAVQNQPTPAQPGVYNNMSITVSMAGGNTNVQNMN
PMMAQMQMSSLQMPGMNTVCPEQINDPALRHTGLYCNQLSSTDLLKTEADGTQDKVITNDGH
>17Colobus angolensis palliatus|A0A2K5IIB9|Mammalia|SRC1
MSGLGDSSSDPANPDSHKRKGSPCDTLASSTEKRRREQENKYLEELAELLSANISDIDSLSVKPDKCKILKKTV
DQIQLMKRMEQEKSTTDDDVQKSDISSSSQGVIEKESLGPLLLEALDGFFFVVNCEGRIVFVSENVTSYLGYNQ
EELMNTSVYSILHVGDHAEFVKNLLPKSLVNGVPWPQEATRRNSHTFNCRMLIHPPDEPGTENQEACQRYEVMQ
CFTVSQPKSIQEDGEDFQSCLICIARRLPRPPAITGVESFMTKQDTTGKIISIDTSSLRAAGRTGWEDLVRKCI
YAFFQPQGREPSYARQLFQEVMTRGTASSPSYRFILNDGTMLSAHTKCKLCYPQSPDMQPFIMGIHIIDREHSG
LSPQDDTNSGMSIPRVNPSVNPSISPAHGVARSSTLPPSNSNMVSTRINRQQSSDLHSGSHSNSSNSQGSFGCS
PGSQIVANVALNQGQASSQSSNPSLNLNNSPMEGTGISLAQFMSPRRQVTSGLTTRPRMPNNSFPPNISTLSSP
IGMTSSACNNNTNRSYSNIPVTSLQGMNEGPNNSVGFSASSPVLRQMSSQNSPSRLNIQPAKAESKDNKEIASI
LNEMIQSDNSSSDSKPLDSGLLHNNDRLSDGDSKYSQTSHKLVQLLTTTAEQQLRHADIDTSCKDVLSCTGTSN
SASANSSGGSCPSSHSSLTERHKILHRLLQEGSPSDITTLSVEPDKKDSASTSVSVTGQVQGNSSIKLELDASK
KKESKDHQLLRYLLDKDEKDLRSTPNLSLDDVKVKVEKKEQMDPCNTNPTPMTKPTPEEIKLEAQSQFTADLDQ

FDQLLPTLEKAAQLPGLCETDRMDGAVTSVTIKSEILPASLQSATARPTSRLNRLPELELEAIDNQFGQPGTGD
QIPWTNNTVTAINQNKSEDQCISSQLDELLCPPTTVEGRNDEKALLEQLVSFLSGKDETELAELDRALGIDKLV
QGGGLDVLSERFPPQQATPPLMMEERPNLYSQPYSSPSPTANLPSPFQGIVRQKPSLGTMPVQVTPPRGAFSPG
MGMQPRQTLNRPPAAPNQLRLQLQQRLQGQQQLIHQNRQAILNQFAATAPVGINMRSGMQQQITPQPPLNAQML
AQRQRELYSQQHRQRQLIQQQRAMLMRQQSFGNNLPPSSGLPVQMGNPRLPQGAPQQFPYPPNYGTNPGTPPAS
TSPFSQLAANPEASLANRNSMVSRGMTGNMGGQFGTGINPQIQQNVFQYPGAGMVPQGEANFAPSLSPGSSMVP
MPIPPPQSSLLQQTPPASGYQSPDMKAWQQGAIGNNVFSQAVQNQPTPAQPGVYNNMSITVSMAGGNTNVQNMN
PMMAQMQMSSLQMPGMNTVCPEQINDPALRHTGLYCNQLSSTDLLKTEADGTQDKKTEEFFSVVTTD

>18Cercocebus atys|A0A2K5MIE6|Sarcopterygii|SRC1
MSGLGDSSSDPANPDSHKRKGSPCDTLASSTEKRRREQENKYLEELAELLSANISDIDSLSVKPDKCKILKKTV
DQIQLMKRMEQEKSTTDDDVQKSDISSSSQGVIEKESLGPLLLEALDGFFFVVNCEGRIVFVSENVTSYLGYNQ
EELMNTSVYSILHVGDHAEFVKNLLPKSLVNGVPWPQEATRRNSHTFNCRMLIHPPDEPGTENQEACQRYEVMQ
CFTVSQPKSIQEDGEDFQSCLICIARRLPRPPAITGVESFMTKQDTTGKIISIDTSSLRAAGRTGWEDLVRKCI
YAFFQPQGREPSYARQLFQEVMTRGTASSPSYRFILNDGTMLSAHTKCKLCYPQSPDMQPFIMGIHIIDREHSG
LSPQDDTNSGMSIPRVNPSVNPSISPAHGVARSSTLPPSNSNMVSTRINRQQSSDLHSGSHSNSSNSQGSFGCS
PGSQIVANVALNQGQASSQSSNPSLNLNNSPMEGTGISLAQFMSPRRQVTSGLTTRPRMPNNSFPPNISTLSSP
VGMTSSACNNNTNRSYSNIPVTSLQGMNEGPNNSVGFSANSPVLRQMSSQNSPSRLNIQPAKAESKDNKEIASI
LNEMIQSDNSSSDGKPLDSGLLHNNDRLSDGDSKYSQTSHKLVQLLTTTAEQQLRHADIDTSCKDVLSCTGTSN
SASANSSGGSCPSSHSSLTERHKILHRLLQEGSPSDITTLSVEPDKKDSASTSVSVTGQVQGNSSIKLELDASK
KKESKDHQLLRYLLDKDEKDLRSTPNLSLDDVKVKVEKKEQMDPCNTNPTPMTKPTPEEIKLEAQSQFTADLDQ
FDQLLPTLEKAAQLPGLCETDRMDGAVTSVTIKSEILPASHQSATARPTSRLNRLPELELEAIDNQFGQPGTGD
QIPWTNNTVTAINQNKSEDQCISSQLDELLCPPTTVEGRNDEKALLEQLVSFLSGKDETELAELDRALGIDKLV
QGGGLDVLSERFPPQQATPPLMMEERPNLYSQPYSSPSPTANLPSPFQGMVRQKPSLGTMPVQVTPPRGAFSPG
MGMQPRQTLNRPPAAPNQLRLQLQQRLQGQQQLIHQNRQAILNQFAATAPVGINMRSGMQQQITPQPPLNAQML
AQRQRELYSQQHRQRQLIQQQRAMLMRQQSFGNNLPPSSGLPVQMGNPRLPQGAPQQFPYPPNYGTNPGTPPAS
TSPFSQLAANPEASLANRNSMVSRGMTGNMGGQFGTGINPQIQQNVFQYPGAGMVPQGEASFAPSLSPGSSMVP
MPIPPPQSSLLQQTPPASGYQSPDMKAWQQGAIGNNVFSQAVQNQPTPAQPGVYNNMSITVSMAGGNTNVQNMN
PMMAQMQMSSLQMPGMNTVCPEQINDPALRHTGLYCNQLSSTDLLKTEADGTQQVQQVQVFADVQCTVNLVGGD
PYLNQPGPLGTQKPTSGPQTPQAQQKSLLQQLLTE
>19Cercocebus atys|A0A2K5MIR3|Sarcopterygii|SRC1
MSGLGDSSSDPANPDSHKRKGSPCDTLASSTEKRRREQENKYLEELAELLSANISDIDSLSVKPDKCKILKKTV
DQIQLMKRMEQEKSTTDDDVQKSDISSSSQGVIEKESLGPLLLEALDGFFFVVNCEGRIVFVSENVTSYLGYNQ
EELMNTSVYSILHVGDHAEFVKNLLPKSLVNGVPWPQEATRRNSHTFNCRMLIHPPDEPGTENQEACQRYEVMQ
CFTVSQPKSIQEDGEDFQSCLICIARRLPRPPAITGVESFMTKQDTTGKIISIDTSSLRAAGRTGWEDLVRKCI
YAFFQPQGREPSYARQLFQEVMTRGTASSPSYRFILNDGTMLSAHTKCKLCYPQSPDMQPFIMGIHIIDREHSG
LSPQDDTNSGMSIPRVNPSVNPSISPAHGVARSSTLPPSNSNMVSTRINRQQSSDLHSGSHSNSSNSQGSFGCS
PGSQIVANVALNQGQASSQSSNPSLNLNNSPMEGTGISLAQFMSPRRQVTSGLTTRPRMPNNSFPPNISTLSSP
VGMTSSACNNNTNRSYSNIPVTSLQGMNEGPNNSVGFSANSPVLRQMSSQNSPSRLNIQPAKAESKDNKEIASI
LNEMIQSDNSSSDGKPLDSGLLHNNDRLSDGDSKYSQTSHKLVQLLTTTAEQQLRHADIDTSCKDVLSCTGTSN
SASANSSGGSCPSSHSSLTERHKILHRLLQEGSPSDITTLSVEPDKKDSASTSVSVTGQVQGNSSIKLELDASK
KKESKDHQLLRYLLDKDEKDLRSTPNLSLDDVKVKVEKKEQMDPCNTNPTPMTKPTPEEIKLEAQSQFTADLDQ
FDQLLPTLEKAAQLPGLCETDRMDGAVTSVTIKSEILPASHQSATARPTSRLNRLPELELEAIDNQFGQPGTGD
QIPWTNNTVTAINQNKSEDQCISSQLDELLCPPTTVEGRNDEKALLEQLVSFLSGKDETELAELDRALGIDKLV
QGGGLDVLSERFPPQQATPPLMMEERPNLYSQPYSSPSPTANLPSPFQGMVRQKPSLGTMPVQVTPPRGAFSPG
MGMQPRQTLNRPPAAPNQLRLQLQQRLQGQQQLIHQNRQAILNQFAATAPVGINMRSGMQQQITPQPPLNAQML
AQRQRELYSQQHRQRQLIQQQRAMLMRQQSFGNNLPPSSGLPVQMGNPRLPQGAPQQFPYPPNYGTNPGTPPAS
TSPFSQLAANPEASLANRNSMVSRGMTGNMGGQFGTGINPQIQQNVFQYPGAGMVPQGEASFAPSLSPGSSMVP
MPIPPPQSSLLQQTPPASGYQSPDMKAWQQGAIGNNVFSQAVQNQPTPAQPGVYNNMSITVSMAGGNTNVQNMN
PMMAQMQMSSLQMPGMNTVCPEQINDPALRHTGLYCNQLSSTDLLKTEADGTQDKKTEEFFSVVTTD
>20Cercocebus atys|A0A2K5MIR9|Sarcopterygii|SRC1
MSGLGDSSSDPANPDSHKRKGSPCDTLASSTEKRRREQENKYLEELAELLSANISDIDSLSVKPDKCKILKKTV
DQIQLMKRMEQEKSTTDDDVQKSDISSSSQGVIEKESLGPLLLEALDGFFFVVNCEGRIVFVSENVTSYLGYNQ
EELMNTSVYSILHVGDHAEFVKNLLPKSLVNGVPWPQEATRRNSHTFNCRMLIHPPDEPGTENQEACQRYEVMQ

CFTVSQPKSIQEDGEDFQSCLICIARRLPRPPAITGVESFMTKQDTTGKIISIDTSSLRAAGRTGWEDLVRKCI
YAFFQPQGREPSYARQLFQEVMTRGTASSPSYRFILNDGTMLSAHTKCKLCYPQSPDMQPFIMGIHIIDREHSG
LSPQDDTNSGMSIPRVNPSVNPSISPAHGVARSSTLPPSNSNMVSTRINRQQSSDLHSGSHSNSSNSQGSFGCS
PGSQIVANVALNQGQASSQSSNPSLNLNNSPMEGTGISLAQFMSPRRQVTSGLTTRPRMPNNSFPPNISTLSSP
VGMTSSACNNNTNRSYSNIPVTSLQGMNEGPNNSVGFSANSPVLRQMSSQNSPSRLNIQPAKAESKDNKEIASI
LNEMIQSDNSSSDGKPLDSGLLHNNDRLSDGDSKYSQTSHKLVQLLTTTAEQQLRHADIDTSCKDVLSCTGTSN
SASANSSGGSCPSSHSSLTERHKILHRLLQEGSPSDITTLSVEPDKKDSASTSVSVTGQVQGNSSIKLELDASK
KKESKDHQLLRYLLDKDEKDLRSTPNLSLDDVKVKVEKKEQMDPCNTNPTPMTKPTPEEIKLEAQSQFTADLDQ
FDQLLPTLEKAAQLPGLCETDRMDGAVTSVTIKSEILPASHQSATARPTSRLNRLPELELEAIDNQFGQPGTGD
QIPWTNNTVTAINQNKSEDQCISSQLDELLCPPTTVEGRNDEKALLEQLVSFLSGKDETELAELDRALGIDKLV
QGGGLDVLSERFPPQQATPPLMMEERPNLYSQPYSSPSPTANLPSPFQGMVRQKPSLGTMPVQVTPPRGAFSPG
MGMQPRQTLNRPPAAPNQLRLQLQQRLQGQQQLIHQNRQAILNQFAATAPVGINMRSGMQQQITPQPPLNAQML
AQRQRELYSQQHRQRQLIQQQRAMLMRQQSFGNNLPPSSGLPVQMGNPRLPQGAPQQFPYPPNYGTNPGTPPAS
TSPFSQLAANPEASLANRNSMVSRGMTGNMGGQFGTGINPQIQQNVFQYPGAGMVPQGEASFAPSLSPGSSMVP
MPIPPPQSSLLQQTPPASGYQSPDMKAWQQGAIGNNVFSQAVQNQPTPAQPGVYNNMSITVSMAGGNTNVQNMN
PMMAQMQMSSLQMPGMNTVCPEQINDPALRHTGLYCNQLSSTDLLKTEADGTQQVQQVQVFADVQCTVNLSLAS
YLDEGFWRRTKTTSSKKKMKLRLRRSVPALPRKKGLWGFACARPPKAVTCLEISSPPHPNPRQLVCTISFSSNS
VSVGFKRVFCLHICTPSPFFKDGFKPRCLQERGRN
>21Cebus imitator|A0A2K5Q806|Mammalia|SRC1
MSGLGDSSSDPANPDSHKRKGSPCDTLASSTEKRRREQENKYLEELAELLSANISDIDSLSVKPDKCKILKKTV
DQIQLMKRMEQEKSTTDDDVQKSDISSSSQGVIEKESLGPLLLEALDGFFFVVNCEGRIVFVSENVTSYLGYNQ
EELMNTSVYSILHVGDHAEFVKNLLPKSLVNGVPWPQEATRRNSHTFNCRMLIHPPDEPGTENQEACQRYEVMQ
CFTVSQPKSIQEDGEDFQSCLICIARRLPRPPAITGVESFMTKQDTTGKIISIDTSSLRAAGRTGWEDLVRKCI
YAFFQPQGREPSYARQLFQEVMTRGTASSPSYRFILNDGTMLSAHTKCKLCYPQSPDMQPFIMGIHIIDREHSG
LSPQDDTNSGMSIPRVNPSVNPNISPAHGVARSSTLPPSNSNMVSTRINRQQSSDLHSSSHSNSSNSQGSFGCS
PGSQIVANVALNQGQASSQSSNPSLNLNNSPMEGTGISLAQFMSPRRQVTSGLATRPRMPSNSFPPNISTLSSP
VGMTSSACNNNNRSYSNIPVTSLQGMNEGPNNSVGFSASSPVLRQMSSQNSPSRLNIQPAKAESKDNKEIASIL
NEMIQSDNSSSDGKPLDSGLLHNSDRLSDGDNKYSQTSHKLVQLLTTTAEQQLRHADIDTSCKDVLSCTGTSNS
ASANSSGGSCPSSHSSLTERHKILHRLLQEGSPSDITTLSVEPDKKDSASTSVSVTGQVQGNSSIKLELDASKK
KESKDHQLLRYLLDKDEKDLRSTPNLSLDDVKVKVEKKEQMDPCNTNPTPMTKPAPEEIKLEAQSQFTADLDQF
DQLLPTLEKAAQLPGLCETDRMDGAVTSVTIKSEILPASLQSATARPTSRLNRLPELELEAIDNQFGQPGTGDQ
IPWTNNTVTAINQNKSEDQCISSQLDELLCPPTTVEGRNDEKALLEQLVSFLSGKDETELAELDRALGIDKLVQ
GGGLDVLSERFPPQQATPPLMMEERPSLYSQSYSSPSPTANLPSPFQGMVRQKPSLGTMPVQVTPPRGAFSPGM
GMQPRQTLNRPPAAPNQLRLQLQQRLQGQQQLIHQNRQAILNQFAATAPVGINMRSGMQQQITPQPPLNAQMLA
QRQRELYSQQHRQRQLIQQQRAMLMRQQSFGNNLPPSSGLPVQMGNPRLPQGAPQQFPYPPNYGTNPGTPPAST
SPFSQLAANPEASLANRSSMVSRGMTGNMGGQFGTGINPQMQQNVFQYPGAGMVPQGEANFAPSLSPGSSMVPM
PIPPPQSSLLQQTPPASGYQSPDMKAWQQGAIGNNNVFSQAVQNQPTPAQPGVYNNMSITVSMAGGNTNVQNMN
PMMGQMQMSSLQMPGMNTVCPEQINDPALRHTGLYCNQLSSTDLLKTEADGTQDKKTEEFFSVVTTD
>22Cebus imitator|A0A2K5Q826|Mammalia|SRC1
MSGLGDSSSDPANPDSHKRKGSPCDTLASSTEKRRREQENKYLEELAELLSANISDIDSLSVKPDKCKILKKTV
DQIQLMKRMEQEKSTTDDDVQKSDISSSSQGVIEKESLGPLLLEALDGFFFVVNCEGRIVFVSENVTSYLGYNQ
EELMNTSVYSILHVGDHAEFVKNLLPKSLVNGVPWPQEATRRNSHTFNCRMLIHPPDEPGTENQEACQRYEVMQ
CFTVSQPKSIQEDGEDFQSCLICIARRLPRPPAITGVESFMTKQDTTGKIISIDTSSLRAAGRTGWEDLVRKCI
YAFFQPQGREPSYARQLFQEVMTRGTASSPSYRFILNDGTMLSAHTKCKLCYPQSPDMQPFIMGIHIIDREHSG
LSPQDDTNSGMSIPRVNPSVNPNISPAHGVARSSTLPPSNSNMVSTRINRQQSSDLHSSSHSNSSNSQGSFGCS
PGSQIVANVALNQGQASSQSSNPSLNLNNSPMEGTGISLAQFMSPRRQVTSGLATRPRMPSNSFPPNISTLSSP
VGMTSSACNNNNRSYSNIPVTSLQGMNEGPNNSVGFSASSPVLRQMSSQNSPSRLNIQPAKAESKDNKEIASIL
NEMIQSDNSSSDGKPLDSGLLHNSDRLSDGDNKYSQTSHKLVQLLTTTAEQQLRHADIDTSCKDVLSCTGTSNS
ASANSSGGSCPSSHSSLTERHKILHRLLQEGSPSDITTLSVEPDKKDSASTSVSVTGQVQGNSSIKLELDASKK
KESKDHQLLRYLLDKDEKDLRSTPNLSLDDVKVKVEKKEQMDPCNTNPTPMTKPAPEEIKLEAQSQFTADLDQF
DQLLPTLEKAAQLPGLCETDRMDGAVTSVTIKSEILPASLQSATARPTSRLNRLPELELEAIDNQFGQPGTGDQ
IPWTNNTVTAINQNKSEDQCISSQLDELLCPPTTVEGRNDEKALLEQLVSFLSGKDETELAELDRALGIDKLVQ
GGGLDVLSERFPPQQATPPLMMEERPSLYSQSYSSPSPTANLPSPFQGMVRQKPSLGTMPVQVTPPRGAFSPGM
GMQPRQTLNRPPAAPNQLRLQLQQRLQGQQQLIHQNRQAILNQFAATAPVGINMRSGMQQQITPQPPLNAQMLA

QRQRELYSQQHRQRQLIQQQRAMLMRQQSFGNNLPPSSGLPVQMGNPRLPQGAPQQFPYPPNYGTNPGTPPAST
SPFSQLAANPEASLANRSSMVSRGMTGNMGGQFGTGINPQMQQNVFQYPGAGMVPQGEANFAPSLSPGSSMVPM
PIPPPQSSLLQQTPPASGYQSPDMKAWQQGAIGNNNVFSQAVQNQPTPAQPGVYNNMSITVSMAGGNTNVQNMN
PMMGQMQMSSLQMPGMNTVCPEQINDPALRHTGLYCNQLSSTDLLKTEADGTQQVQQVQVFADVQCTVNLVGGD
PYLNQPGPLGTQKPTSGPQTPQAQQKSLLQQLLTE

>23Macaca fascicularis|A0A2K5WTX9|Sarcopterygii|SRC1
MSGLGDSSSDPANPDSHKRKGSPCDTLASSTEKRRREQENKYLEELAELLSANISDIDSLSVKPDKCKILKKTV
DQIQLMKRMEQEKSTTDDDVQKSDISSSSQGVIEKESLGPLLLEALDGFFFVVNCEGRIVFVSENVTSYLGYNQ
EELMNTSVYSILHVGDHAEFVKNLLPKSLVNGVPWPQEATRRNSHTFNCRMLIHPPDEPGTENQEACQRYEVMQ
CFTVSQPKSIQEDGEDFQSCLICIARRLPRPPAITGVESFMTKQDTTGKIISIDTSSLRAAGRTGWEDLVRKCI
YAFFQPQGREPSYARQLFQEVMTRGTASSPSYRFILNDGTMLSAHTKCKLCYPQSPDMQPFIMGIHIIDREHSG
LSPQDDTNSGMSIPRVNPSVNPSISPAHGVARSSTLPPSNSNMVSTRINRQQSSDLHSGSHSNSSNSQGSFGCS
PGSQIVANVALNQGQASSQSSNPSLNLNNSPMEGTGISLAQFMSPRRQVTSGLTTRPRMPNNSFPPNISTLSSP
VGMTSSACNNNTNRSYSNIPVTSLQGMNEGPNNSVGFSANSPVLRQMSSQNSPSRLNIQPAKAESKDNKEIASI
LNEMIQSDNSSSDGKPLDSGLLHNNDRLSDGDSKYSQTSHKLVQLLTTTAEQQLRHADIDTSCKDVLSCTGTSN
SASANSSGGSCPSSHSSLTERHKILHRLLQEGSPSDITTLSVEPDKKDSASTSVSVTGQVQGNSSIKLELDASK
KKESKDHQLLRYLLDKDEKDLRSTPNLSLDDVKVKVEKKEQMDPCNTNPTPMTKPTPEEIKLEAQSQFTADLDQ
FDQLLPTLEKAAQLPGLCETDRMDGAVTSVTIKSEILPASLQSATARPTSRLNRLPELELEAIDNQFGQPGTGD
QIPWTNNTVTAINQNKSEDQCISSQLDELLCPPTTVEGRNDEKALLEQLVSFLSGKDETELAELDRALGIDKLV
QGGGLDVLSERFPPQQATPPLMMEERPNLYSQPYSSPSPTANLPSPFQGMVRQKPSLGTMPVQVTPPRGAFSPG
MGMQPRQTLNRPPAAPNQLRLQLQQRLQGQQQLIHQNRQAILNQFAATAPVGINMRSGMQQQITPQPPLNAQML
AQRQRELYSQQHRQRQLIQQQRAMLMRQQSFGNNLPPSSGLPVQMGNPRLPQGAPQQFPYPPNYGTNPGTPPAS
TSPFSQLAANPEASLANRNSMVSRGMTGNMGGQFGTGINPQIQQNVFQYPGAGMVPQGEASFAPSLSPGSSMVP
MPIPPPQSSLLQQTPPASGYQSPDMKAWQQGAIGNNVFSQAVQNQPTPAQPGVYNNMSITVSMAGGNTNVQNMN
PMMAQMQMSSLQMPGMNTVCPEQINDPALRHTGLYCNQLSSTDLLKTEADGTQQVQQVQVFADVQCTVNLSLAS
YLDEGFWRRTKTTSSKTKMKLRLRHSVPALPRKKGLWGFACARPPKAVTCLEISSPPHPNPRQLVCTISFSSNS
VSVGFKRVFCLHICTPSPFFKDGFKPRCLQERGRNEYIHRGIQKIQFGGKCNNF
>24Mandrillus leucophaeus|A0A2K5YNS7|Mammalia|SRC1
MSGLGDSSSDPANPDSHKRKGSPCDTLASSTEKRRREQENKYLEELAELLSANISDIDSLSVKPDKCKILKKTV
DQIQLMKRMEQEKSTTDDDVQKSDISSSSQGVIEKESLGPLLLEALDGFFFVVNCEGRIVFVSENVTSYLGYNQ
EELMNTSVYSILHVGDHAEFVKNLLPKSLVNGVPWPQEATRRNSHTFNCRMLIHPPDEPGTENQEACQRYEVMQ
CFTVSQPKSIQEDGEDFQSCLICIARRLPRPPAITGVESFMTKQDTTGKIISIDTSSLRAAGRTGWEDLVRKCI
YAFFQPQGREPSYARQLFQEVMTRGTASSPSYRFILNDGTMLSAHTKCKLCYPQSPDMQPFIMGIHIIDREHSG
LSPQDDTNSGMSIPRVNPSVNPSISPAHGVARSSTLPPSNSNMVSTRINRQQSSDLHSGSHSNSSNSQGSFGCS
PGSQIVANVALNQGQASSQSSNPSLNLNNSPMEGTGISLAQFMSPRRQVTSGLTTRPRMPNNSFPPNISALSSP
VGMTSSACNNNTNRSYSNIPVTSLQGMNEGPNNSVGFSANSPVLRQMSSQNSPSRLNIQPAKAESKDNKEIASI
LNEMIQSDNSSSDGKPLDSGLLHNNDRLSDGDSKYSQTSHKLVQLLTTTAEQQLRHADIDTSCKDVLSCTGTSN
SASANSSGGSCPSSHSSLTERHKILHRLLQEGSPSDITTLSVEPDKKDSASTSVSVTGQVQGNSSIKLELDASK
KKESKDHQLLRYLLDKDEKDLRSTPNLSLDDVKVKVEKKEQMDPCNTNPTPMTKPTPEEIKLEAQSQFTADLDQ
FDQLLPTLEKAAQLPGLCETDRMDGAVTSVTIKSEILPASLQSATARPTSRLNRLPELELEAIDNQFGQPGTGD
QIPWTNNTVTAINQNKSEDQCISSQLDELLCPPTTVEGRNDEKALLEQLVSFLSGKDETELAELDRALGIDKLV
QGGGLDVLSERFPPQQATPPLMMEERPNLYSQPYSSPSPTANLPSPFQGMVRQKPSLGTMPVQVTPPRGAFSPG
MGMQPRQTLNRPPAAPNQLRLQLQQRLQGQQQLIHQNRQAILNQFAATAPVGINMRSGMQQQITPQPPLNAQML
AQRQRELYSQQHRQRQLIQQQRAMLMRQQSFGNNLPPSSGLPVQMGNPRLPQGAPQQFPYPPNYGTNPGTPPAS
TSPFSQLAANPEASLANRNSMVSRGMTGNMGGQFGTGINPQIQQNVFQYPGAGMVPQGEASFAPSLSPGSSMVP
MPIPPPQSSLLQQTPPASGYQSPDMKAWQQGAIGNNVFSQAVQNQPTPAQPGVYNNMSITVSMAGGNTNVQNMN
PMMAQMQMSSLQMPGMNTVCPEQINDPALRHTGLYCNQLSSTDLLKTEADGTQQVQQVQVFADVQCTVNLVGGD
PYLNQPGPLGTQKPTSGPQTPQAQQKSLLQQLLTE

>25Mandrillus leucophaeus|A0A2K5YNU2|Mammalia|SRC1
MSGLGDSSSDPANPDSHKRKGSPCDTLASSTEKRRREQENKYLEELAELLSANISDIDSLSVKPDKCKILKKTV
DQIQLMKRMEQEKSTTDDDVQKSDISSSSQGVIEKESLGPLLLEALDGFFFVVNCEGRIVFVSENVTSYLGYNQ
EELMNTSVYSILHVGDHAEFVKNLLPKSLVNGVPWPQEATRRNSHTFNCRMLIHPPDEPGTENQEACQRYEVMQ

CFTVSQPKSIQEDGEDFQSCLICIARRLPRPPAITGVESFMTKQDTTGKIISIDTSSLRAAGRTGWEDLVRKCI
YAFFQPQGREPSYARQLFQEVMTRGTASSPSYRFILNDGTMLSAHTKCKLCYPQSPDMQPFIMGIHIIDREHSG
LSPQDDTNSGMSIPRVNPSVNPSISPAHGVARSSTLPPSNSNMVSTRINRQQSSDLHSGSHSNSSNSQGSFGCS
PGSQIVANVALNQGQASSQSSNPSLNLNNSPMEGTGISLAQFMSPRRQVTSGLTTRPRMPNNSFPPNISALSSP
VGMTSSACNNNTNRSYSNIPVTSLQGMNEGPNNSVGFSANSPVLRQMSSQNSPSRLNIQPAKAESKDNKEIASI
LNEMIQSDNSSSDGKPLDSGLLHNNDRLSDGDSKYSQTSHKLVQLLTTTAEQQLRHADIDTSCKDVLSCTGTSN
SASANSSGGSCPSSHSSLTERHKILHRLLQEGSPSDITTLSVEPDKKDSASTSVSVTGQVQGNSSIKLELDASK
KKESKDHQLLRYLLDKDEKDLRSTPNLSLDDVKVKVEKKEQMDPCNTNPTPMTKPTPEEIKLEAQSQFTADLDQ
FDQLLPTLEKAAQLPGLCETDRMDGAVTSVTIKSEILPASLQSATARPTSRLNRLPELELEAIDNQFGQPGTGD
QIPWTNNTVTAINQNKSEDQCISSQLDELLCPPTTVEGRNDEKALLEQLVSFLSGKDETELAELDRALGIDKLV
QGGGLDVLSERFPPQQATPPLMMEERPNLYSQPYSSPSPTANLPSPFQGMVRQKPSLGTMPVQVTPPRGAFSPG
MGMQPRQTLNRPPAAPNQLRLQLQQRLQGQQQLIHQNRQAILNQFAATAPVGINMRSGMQQQITPQPPLNAQML
AQRQRELYSQQHRQRQLIQQQRAMLMRQQSFGNNLPPSSGLPVQMGNPRLPQGAPQQFPYPPNYGTNPGTPPAS
TSPFSQLAANPEASLANRNSMVSRGMTGNMGGQFGTGINPQIQQNVFQYPGAGMVPQGEASFAPSLSPGSSMVP
MPIPPPQSSLLQQTPPASGYQSPDMKAWQQGAIGNNVFSQAVQNQPTPAQPGVYNNMSITVSMAGGNTNVQNMN
PMMAQMQMSSLQMPGMNTVCPEQINDPALRHTGLYCNQLSSTDLLKTEADGTQDKKTEEFFSVVTTD

>26Macaca nemestrina|A0A2K6D6P9|Mammalia|SRC1
MSGLGDSSSDPANPDSHKRKGSPCDTLASSTEKRRREQENKYLEELAELLSANISDIDSLSVKPDKCKILKKTV
DQIQLMKRMEQEKSTTDDDVQKSDISSSSQGVIEKESLGPLLLEALDGFFFVVNCEGRIVFVSENVTSYLGYNQ
EELMNTSVYSILHVGDHAEFVKNLLPKSLVNGVPWPQEATRRNSHTFNCRMLIHPPDEPGTENQEACQRYEVMQ
CFTVSQPKSIQEDGEDFQSCLICIARRLPRPPAITGVESFMTKQDTTGKIISIDTSSLRAAGRTGWEDLVRKCI
YAFFQPQGREPSYARQLFQEVMTRGTASSPSYRFILNDGTMLSAHTKCKLCYPQSPDMQPFIMGIHIIDREHSG
LSPQDDTNSGMSIPRVNPSVNPSISPAHGVARSSTLPPSNSNMVSTRINRQQSSDLHSGSHSSSSNSQGSFGCS
PGSQIVANVALNQGQASSQSSNPSLNLNNSPMEGTGISLAQFMSPRRQVTSGLTTRPRMPNNSFPPNISTLSSP
VGMTSSACNNNTNRSYSNIPVTSLQGMNEGPNNSVGFSANSPVLRQMSSQNSPSRLNIQPAKAESKDNKEIASI
LNEMIQSDNSSSDGKPLDSGLLHNNDRLSDGDSKYSQTSHKLVQLLTTTAEQQLRHADIDTSCKDVLSCTGTSN
SASANSSGGSCPSSHSSLTERHKILHRLLQEGSPSDITTLSVEPDKKDSASTSVSVTGQVQGNSSIKLELDASK
KKESKDHQLLRYLLDKDEKDLRSTPNLSLDDVKVKVEKKEQMDPCNTNPTPMTKPTPEEIKLEAQSQFTADLDQ
FDQLLPTLEKAAQLPGLCETDRMDGAVTSVTIKSEILPASLQSATARPTSRLNRLPELELEAIDNQFGQPGTGD
QIPWTNNTVTAINQNKSEDQCISSQLDELLCPPTTVEGRNDEKALLEQLVSFLSGKDETELAELDRALGIDKLV
QGGGLDVLSERFPPQQATPPLMMEERPNLYSQPYSSPSPTANLPSPFQGMVRQKPSLGTMPVQVTPPRGAFSPS
MGMQPRQTLNRPPAAPNQLRLQLQQRLQGQQQLIHQNRQAILNQFAATAPVGINMRSGMQQQITPQPPLNAQML
AQRQRELYSQQHRQRQLIQQQRAMLMRQQSFGNNFPPSSGLPVQMGNPRLPQGAPQQFPYPPNYGTNPGTPPAS
TSPFSQLAANPEASLANRNSMVSRGMTGNMGGQFGTGINPQIQQNVFQYPGAGMVPQGEASFAPSLSPGSSMVP
MPIPPPQSSLLQQTPPASGYQSPDMKAWQQGAIGNNVFSQAVQNQPTPAQPGVYNNMSITVSMAGGNTNVQNMN
PMMAQMQMSSLQMPGMNTVCPEQINDPALRHTGLYCNQLSSTDLLKTEADGTQDKKTEEFFSVVTTD

>27Macaca nemestrina|A0A2K6D6R7|Mammalia|SRC1
MSGLGDSSSDPANPDSHKRKGSPCDTLASSTEKRRREQENKYLEELAELLSANISDIDSLSVKPDKCKILKKTV
DQIQLMKRMEQEKSTTDDDVQKSDISSSSQGVIEKESLGPLLLEALDGFFFVVNCEGRIVFVSENVTSYLGYNQ
EELMNTSVYSILHVGDHAEFVKNLLPKSLVNGVPWPQEATRRNSHTFNCRMLIHPPDEPGTENQEACQRYEVMQ
CFTVSQPKSIQEDGEDFQSCLICIARRLPRPPAITGVESFMTKQDTTGKIISIDTSSLRAAGRTGWEDLVRKCI
YAFFQPQGREPSYARQLFQEVMTRGTASSPSYRFILNDGTMLSAHTKCKLCYPQSPDMQPFIMGIHIIDREHSG
LSPQDDTNSGMSIPRVNPSVNPSISPAHGVARSSTLPPSNSNMVSTRINRQQSSDLHSGSHSSSSNSQGSFGCS
PGSQIVANVALNQGQASSQSSNPSLNLNNSPMEGTGISLAQFMSPRRQVTSGLTTRPRMPNNSFPPNISTLSSP
VGMTSSACNNNTNRSYSNIPVTSLQGMNEGPNNSVGFSANSPVLRQMSSQNSPSRLNIQPAKAESKDNKEIASI
LNEMIQSDNSSSDGKPLDSGLLHNNDRLSDGDSKYSQTSHKLVQLLTTTAEQQLRHADIDTSCKDVLSCTGTSN
SASANSSGGSCPSSHSSLTERHKILHRLLQEGSPSDITTLSVEPDKKDSASTSVSVTGQVQGNSSIKLELDASK
KKESKDHQLLRYLLDKDEKDLRSTPNLSLDDVKVKVEKKEQMDPCNTNPTPMTKPTPEEIKLEAQSQFTADLDQ
FDQLLPTLEKAAQLPGLCETDRMDGAVTSVTIKSEILPASLQSATARPTSRLNRLPELELEAIDNQFGQPGTGD
QIPWTNNTVTAINQNKSEDQCISSQLDELLCPPTTVEGRNDEKALLEQLVSFLSGKDETELAELDRALGIDKLV
QGGGLDVLSERFPPQQATPPLMMEERPNLYSQPYSSPSPTANLPSPFQGMVRQKPSLGTMPVQVTPPRGAFSPS
MGMQPRQTLNRPPAAPNQLRLQLQQRLQGQQQLIHQNRQAILNQFAATAPVGINMRSGMQQQITPQPPLNAQML

AQRQRELYSQQHRQRQLIQQQRAMLMRQQSFGNNFPPSSGLPVQMGNPRLPQGAPQQFPYPPNYGTNPGTPPAS
TSPFSQLAANPEASLANRNSMVSRGMTGNMGGQFGTGINPQIQQNVFQYPGAGMVPQGEASFAPSLSPGSSMVP
MPIPPPQSSLLQQTPPASGYQSPDMKAWQQGAIGNNVFSQAVQNQPTPAQPGVYNNMSITVSMAGGNTNVQNMN
PMMAQMQMSSLQMPGMNTVCPEQINDPALRHTGLYCNQLSSTDLLKTEADGTQQVQQVQVFADVQCTVNLVGGD
PYLNQPGPLGTQKPTSGPQTPQAQQKSLLQQLLTE
>28Rhinopithecus bieti|A0A2K6LH38|Mammalia|SRC1
MSGLGDSSSDPANPDSHKRKGSPCDTLASSTEKRRREQENKYLEELAELLSANISDIDSLSVKPDKCKILKKTV
DQIQLMKRMEQGKKQTNKKPMAVCFKRESLGPLLLEALDGFFFVVNCEGRIVFVSENVTSYLGYNQEELMNTSV
YSILHVGDHAEFVKNLLPKSLVNGVPWPQEATRRNSHTFNCRMLIHPPDEPGTENQEACQRYEVMQCFTVSQPK
SIQEDGEDFQSCLICIARRLPRPPAITGVESFMTKQDTTGKIISIDTSSLRAAGRTGWEDLVRKCIYAFFQPQG
REPSYARQLFQEVMTRGTASSPSYRFILNDGTMLSAHTKCKLCYPQSPDMQPFIMGIHIIDREHSGLSPQDDTN
SGMSIPRVNPSVNPSISSAHGVARSSTLPPSNSNMVSTRINRQQSSDLHSGSHSNSSNSQGSFGCSPGSQIVAN
VALNQGQASSQSSNPSLNLNNSPMEGTGISLAQFMSPRRQVTSGLTTRPRMPNNSFPPNISTLSSPIGMTSSAC
NNNTNRSYSNIPVTSLQGMNEGPNNSVGFSANSPVLRQMSSQNSPSRLNIQPAKAESKDNKEIASILNEMIQSD
NSSSDSKPLDSGLLHNNDRLSDGDSKYSQTSHKLVQLLTTTAEQQLRHADIDTSCKDVLSCTGTSNSASANSSG
GSCPSSHSSLTERHKILHRLLQEGSPSDITTLSVEPDKKDSASTSVSVTGQVQGNSSIKLELDASKKKESKDHQ
LLRYLLDKDEKDLRSTPNLSLDDVKVKVEKKEQMDPCNTNPTPMTKPTPEEIKLEAQSQFTADLDQFDQLLPTL
EKAAQLPGLCETDRMDGVVTSVTIKSEILPASLQSATARPTSRLNRLPELELEAIDNQFGQPGTGDQSPWTNNT
VTAINQNKSEDQCISSQLDELLCPPTTVEGRNDEKALLEQLVSFLSGKDETELAELDRALGIDKLVQGGGLDVL
SERFPPQQATPPLMMEERPNLYSQPYSSPSPTANLPSPFQGIVRQKPSLGTMPVQVTPPRGAFSPGMGMQPRQT
LNRPPAAPNQLRLQLQQRLQGQQQLIHQNRQAILNQFAATAPVGINMRSGMQQQITPQPPLNAQMLAQRQRELY
SQQHRQRQLIQQQRAMLMRQQSFGNNLPPSSGLPVQMGNPRLPQGAPQQFPYPPNYGTNPGTPPASTSPFSQLA
ANPEASLASRNSMVSRGMTGNMGGQFGTGINPQIQQNVFQYPGAGMVPQGEANFAPSLSPGSSMVPMPIPPPQS
SLLQQTPPASGYQSPDMKAWQQGAIGNNVFSQAVQNQPTPAQPGVYNNMSITVSMAGGNTNVQNMNPMMAQMQM
SSLQMPGMNTVCPEQINDPTLRHTGLYCNQLSSTDLLKTEADGTQDKKTEEFFSVVTTD
>29Rhinopithecus bieti|A0A2K6LH71|Mammalia|SRC1
MSGLGDSSSDPANPDSHKRKGSPCDTLASSTEKRRREQENKYLEELAELLSANISDIDSLSVKPDKCKILKKTV
DQIQLMKRMEQGKKQTNKKPMAVCFKRESLGPLLLEALDGFFFVVNCEGRIVFVSENVTSYLGYNQEELMNTSV
YSILHVGDHAEFVKNLLPKSLVNGVPWPQEATRRNSHTFNCRMLIHPPDEPGTENQEACQRYEVMQCFTVSQPK
SIQEDGEDFQSCLICIARRLPRPPAITGVESFMTKQDTTGKIISIDTSSLRAAGRTGWEDLVRKCIYAFFQPQG
REPSYARQLFQEVMTRGTASSPSYRFILNDGTMLSAHTKCKLCYPQSPDMQPFIMGIHIIDREHSGLSPQDDTN
SGMSIPRVNPSVNPSISSAHGVARSSTLPPSNSNMVSTRINRQQSSDLHSGSHSNSSNSQGSFGCSPGSQIVAN
VALNQGQASSQSSNPSLNLNNSPMEGTGISLAQFMSPRRQVTSGLTTRPRMPNNSFPPNISTLSSPIGMTSSAC
NNNTNRSYSNIPVTSLQGMNEGPNNSVGFSANSPVLRQMSSQNSPSRLNIQPAKAESKDNKEIASILNEMIQSD
NSSSDSKPLDSGLLHNNDRLSDGDSKYSQTSHKLVQLLTTTAEQQLRHADIDTSCKDVLSCTGTSNSASANSSG
GSCPSSHSSLTERHKILHRLLQEGSPSDITTLSVEPDKKDSASTSVSVTGQVQGNSSIKLELDASKKKESKDHQ
LLRYLLDKDEKDLRSTPNLSLDDVKVKVEKKEQMDPCNTNPTPMTKPTPEEIKLEAQSQFTADLDQFDQLLPTL
EKAAQLPGLCETDRMDGVVTSVTIKSEILPASLQSATARPTSRLNRLPELELEAIDNQFGQPGTGDQSPWTNNT
VTAINQNKSEDQCISSQLDELLCPPTTVEGRNDEKALLEQLVSFLSGKDETELAELDRALGIDKLVQGGGLDVL
SERFPPQQATPPLMMEERPNLYSQPYSSPSPTANLPSPFQGIVRQKPSLGTMPVQVTPPRGAFSPGMGMQPRQT
LNRPPAAPNQLRLQLQQRLQGQQQLIHQNRQAILNQFAATAPVGINMRSGMQQQITPQPPLNAQMLAQRQRELY
SQQHRQRQLIQQQRAMLMRQQSFGNNLPPSSGLPVQMGNPRLPQGAPQQFPYPPNYGTNPGTPPASTSPFSQLA
ANPEASLASRNSMVSRGMTGNMGGQFGTGINPQIQQNVFQYPGAGMVPQGEANFAPSLSPGSSMVPMPIPPPQS
SLLQQTPPASGYQSPDMKAWQQGAIGNNVFSQAVQNQPTPAQPGVYNNMSITVSMAGGNTNVQNMNPMMAQMQM
SSLQMPGMNTVCPEQINDPTLRHTGLYCNQLSSTDLLKTEADGTQQVQQVQVFADVQCTVNLVGGDPYLNQPGP
LGTQKPTSGPQTPQAQQKSLLQQLLTE
>30Rhinopithecus roxellana|A0A2K6R4I8|Sarcopterygii|SRC1
MSGLGDSSSDPANPDSHKRKGSPCDTLASSTEKRRREQENKYLEELAELLSANISDIDSLSVKPDKCKILKKTV
DQIQLMKRMEQEKSTTDDDVQKSDISSSSQGVIEKESLGPLLLEALDGFFFVVNCEGRIVFVSENVTSYLGYNQ
EELMNTSVYSILHVGDHAEFVKNLLPKSLVNGVPWPQEATRRNSHTFNCRMLIHPPDEPGTENQETCQRYEVMQ
CFTVSQPKSIQEDGEDFQSCLICIARRLPRPPAITGVESFMTKQDTTGKIISIDTSSLRAAGRTGWEDLVRKCI
YAFFQPQGREPSYARQLFQEVMTRGTASSPSYRFILNDGTMLSAHTKCKLCYPQSPDMQPFIMGIHIIDREHSG
LSPQDDTNSGMSIPRVNPSVNPSISSAHGVARSSTLPPSNSNMVSTRINRQQSSDLHSGSHSNSSNSQGSFGCS
PGSQIVANVALNQGQASSQSSNPSLNLNNSPMEGTGISLAQFMSPRRQVTSGLTTRPRMPNNSFPPNISTLSSP

IGMTSSACNNNTNRSYSNIPVTSLQGMNEGPNNSVGFSASSPVLRQMSSQNSPSRLNIQPAKAESKDNKEIASI
LNEMIQSDNSSSDSKPLDSGLLHNNDRLSDGDSKYSQTSHKLVQLLTTTAEQQLRHADIDTSCKDVLSCTGTSN
SASANSSGGSCPSSHSSLTERHKILHRLLQEGSPSDITTLSVEPDKKDSASTSVSVTGQVQGNSSIKLELDASK
KKESKDHQLLRYLLDKDEKDLRSTPNLSLDDVKVKVEKKEQMDPCNTNPTPMTKPTPEEIKLEAQSQFTADLDQ
FDQLLPTLEKAAQLPGLCETDRMDGAVTSVTIKSEILPASLQSATARPTSRLNRLPELELEAIDNQFGQPGTGD
QSPWTNNTVTAINQNKSEDQCISSQLDELLCPPTTVEGRNDEKALLEQLVSFLSGKDETELAELDRALGIDKLV
QGGGLDVLSERFPPQQATPPLMMEERPNLYSQPYSSPSPTANLPSPFQGIVRQKPSLGTMPVQVTPPRGAFSPG
MGMQPRQTLNRPPAAPNQLRLQLQQRLQGQQQLIHQNRQAILNQFAATAPVGINMRSGMQQQITPQPPLNAQML
AQRQRELYSQQHRQRQLIQQQRAMLMRQQSFGNNLPPSSGLPVQMGNPRLPQGAPQQFPYPPNYGTNPGTPPAS
TSPFSQLAANPEASLASRNSMVSRGMTGNMGGQFGTGINPQIQQNVFQYPGAGMVPQGEANFAPSLSPGSSMVP
MPIPPPQSSLLQQTPPASGYQSPDMKAWQQGAIGNNVFSQAVQNQPTPAQPGVYNNMSITVSMAGGNTNVQNMN
PMMAQMQMSSLQMPGMNTVCPEQINDPTLRHTGLYCNQLSSTDLLKTEADGTQDKKTEEFFSVVTTD
>31Rhinopithecus roxellana|A0A2K6R4L5|Sarcopterygii|SRC1
MSGLGDSSSDPANPDSHKRKGSPCDTLASSTEKRRREQENKYLEELAELLSANISDIDSLSVKPDKCKILKKTV
DQIQLMKRMEQEKSTTDDDVQKSDISSSSQGVIEKESLGPLLLEALDGFFFVVNCEGRIVFVSENVTSYLGYNQ
EELMNTSVYSILHVGDHAEFVKNLLPKSLVNGVPWPQEATRRNSHTFNCRMLIHPPDEPGTENQETCQRYEVMQ
CFTVSQPKSIQEDGEDFQSCLICIARRLPRPPAITGVESFMTKQDTTGKIISIDTSSLRAAGRTGWEDLVRKCI
YAFFQPQGREPSYARQLFQEVMTRGTASSPSYRFILNDGTMLSAHTKCKLCYPQSPDMQPFIMGIHIIDREHSG
LSPQDDTNSGMSIPRVNPSVNPSISSAHGVARSSTLPPSNSNMVSTRINRQQSSDLHSGSHSNSSNSQGSFGCS
PGSQIVANVALNQGQASSQSSNPSLNLNNSPMEGTGISLAQFMSPRRQVTSGLTTRPRMPNNSFPPNISTLSSP
IGMTSSACNNNTNRSYSNIPVTSLQGMNEGPNNSVGFSASSPVLRQMSSQNSPSRLNIQPAKAESKDNKEIASI
LNEMIQSDNSSSDSKPLDSGLLHNNDRLSDGDSKYSQTSHKLVQLLTTTAEQQLRHADIDTSCKDVLSCTGTSN
SASANSSGGSCPSSHSSLTERHKILHRLLQEGSPSDITTLSVEPDKKDSASTSVSVTGQVQGNSSIKLELDASK
KKESKDHQLLRYLLDKDEKDLRSTPNLSLDDVKVKVEKKEQMDPCNTNPTPMTKPTPEEIKLEAQSQFTADLDQ
FDQLLPTLEKAAQLPGLCETDRMDGAVTSVTIKSEILPASLQSATARPTSRLNRLPELELEAIDNQFGQPGTGD
QSPWTNNTVTAINQNKSEDQCISSQLDELLCPPTTVEGRNDEKALLEQLVSFLSGKDETELAELDRALGIDKLV
QGGGLDVLSERFPPQQATPPLMMEERPNLYSQPYSSPSPTANLPSPFQGIVRQKPSLGTMPVQVTPPRGAFSPG
MGMQPRQTLNRPPAAPNQLRLQLQQRLQGQQQLIHQNRQAILNQFAATAPVGINMRSGMQQQITPQPPLNAQML
AQRQRELYSQQHRQRQLIQQQRAMLMRQQSFGNNLPPSSGLPVQMGNPRLPQGAPQQFPYPPNYGTNPGTPPAS
TSPFSQLAANPEASLASRNSMVSRGMTGNMGGQFGTGINPQIQQNVFQYPGAGMVPQGEANFAPSLSPGSSMVP
MPIPPPQSSLLQQTPPASGYQSPDMKAWQQGAIGNNVFSQAVQNQPTPAQPGVYNNMSITVSMAGGNTNVQNMN
PMMAQMQMSSLQMPGMNTVCPEQINDPTLRHTGLYCNQLSSTDLLKTEADGTQQVQQVQVFADVQCTVNLVGGD
PYLNQPGPLGTQKPTSGPQTPQAQQKSLLQQLLTE
>32Saimiri boliviensis boliviensis|A0A2K6UBP2|Mammalia|SRC1
MSGLGDSSSDPANPDSHKRKGSPCDTLASSTEKRRREQENKYLEELAELLSANISDIDSLSVKPDKCKILKKTV
DQIQLMKRMEQEKSTTDDDVQKSDISSSSQGVIEKESLGPLLLEALDGFFFVVNCEGRIVFVSENVTSYLGYNQ
EELMNTSVYSILHVGDHAEFVKNLLPKSLVNGVPWPQEATRRNSHTFNCRMLIHPPDEPGTENQEACQRYEVMQ
CFTVSQPKSIQEDGEDFQSCLICIARRLPRPPAITGVESFMTKQDTTGKIISIDTSSLRAAGRTGWEDLVRKCI
YAFFQPQGREPSYARQLFQEVMTRGTASSPSYRFILNDGTMLSAHTKCKLCYPQSPDMQPFIMGIHIIDREHSG
LSPQDDTNSGMSIPRVNPSVNPNISPAHGVARSSALPPSNSNMVSTRINRQQSSDLHSSSHSNSSNSQGSFGCS
PGSQIVANVALNQGQASSQSSNPSLNLNNSPMEGTGISLAQFMSPRRQVTSGLATRPRMPNNSFPPNISTLSSP
VGMTSSACNNNNRSYSNIPVTSLQGMNEGPNNSVGFSASSPVLRQMSSQNSPSRLNIQPAKAESKDNKEIASIL
NEMIQSDNSSSDGKPLDSGLLHNSDRLSDGDNKYSQTSHKLVQLLTTTAEQQLRHADIDTSCKDVLSCTGTSNS
ASANSSGGSCPSSHSSLTERHKILHRLLQEGSPSDITTLSVEPDKKDSASTSVSVTGQVQGNSSIKLELDASKK
KESKDHQLLRYLLDKDEKDLRSTPNLSLDDVKVKVEKKEQMDPCNTNPTPMTKPPPEEIKLEAQSQFTADLDQF
DQLLPTLEKAAQLPGLCETDRMDGAVTSVTIKSEILPASLQSATARPTSRLNRLPELELEAIDNQFGQPGTGDQ
IPWTNNAVTAINQNKSEDQCISSQLDELLCPPTTVEGRNDEKALLEQLVSFLSGKDETELAELDRALGIDKLVQ
GGGLDVLSERFPPQQATPPLMMEERPSLYSQPYSSPSPTANLPSPFQGMVRQKPSLGTMPVQVTPPRGAFSPGM
GMQPRQTLNRPPAAPNQLRLQLQQRLQGQQQLIHQNRQAILNQFAATAPVGINMRSGMQQQITPQPPLNAQMLA
QRQRELYSQQHRQRQLIQQQRAMLMRQQSFGNSLPPSSGLPVQMGNPRLPQGAPQQFPYPPNYGTNPGTPPAST
SPFSQLAANPEASLANRSSMVSRGMTGNMGGQFGTGINPQMQQNVFQYPGAGMVPQGEANFAPSLSPGSSMVPM
PIPPPQSSLLQQTPPASGYQSPDMKAWQQGAIGNNNVFSQAVQNQPTPAQPGVYNNMSITVSMAGGNTNVQNMN
PMMGQMQMSSLQMPGMNTVCPEQINDPALRHTGLYCNQLSSTDLLKTEADGTQQVQQVQVFADVQCTVNLVGGD
PYLNQPGPLGTQKPTSGPQTPQAQQKSLLQQLLTE

>33Saimiri boliviensis boliviensis|A0A2K6UBR7|Mammalia|SRC1
MSGLGDSSSDPANPDSHKRKGSPCDTLASSTEKRRREQENKYLEELAELLSANISDIDSLSVKPDKCKILKKTV
DQIQLMKRMEQEKSTTDDDVQKSDISSSSQGVIEKESLGPLLLEALDGFFFVVNCEGRIVFVSENVTSYLGYNQ
EELMNTSVYSILHVGDHAEFVKNLLPKSLVNGVPWPQEATRRNSHTFNCRMLIHPPDEPGTENQEACQRYEVMQ
CFTVSQPKSIQEDGEDFQSCLICIARRLPRPPAITGVESFMTKQDTTGKIISIDTSSLRAAGRTGWEDLVRKCI
YAFFQPQGREPSYARQLFQEVMTRGTASSPSYRFILNDGTMLSAHTKCKLCYPQSPDMQPFIMGIHIIDREHSG
LSPQDDTNSGMSIPRVNPSVNPNISPAHGVARSSALPPSNSNMVSTRINRQQSSDLHSSSHSNSSNSQGSFGCS
PGSQIVANVALNQGQASSQSSNPSLNLNNSPMEGTGISLAQFMSPRRQVTSGLATRPRMPNNSFPPNISTLSSP
VGMTSSACNNNNRSYSNIPVTSLQGMNEGPNNSVGFSASSPVLRQMSSQNSPSRLNIQPAKAESKDNKEIASIL
NEMIQSDNSSSDGKPLDSGLLHNSDRLSDGDNKYSQTSHKLVQLLTTTAEQQLRHADIDTSCKDVLSCTGTSNS
ASANSSGGSCPSSHSSLTERHKILHRLLQEGSPSDITTLSVEPDKKDSASTSVSVTGQVQGNSSIKLELDASKK
KESKDHQLLRYLLDKDEKDLRSTPNLSLDDVKVKVEKKEQMDPCNTNPTPMTKPPPEEIKLEAQSQFTADLDQF
DQLLPTLEKAAQLPGLCETDRMDGAVTSVTIKSEILPASLQSATARPTSRLNRLPELELEAIDNQFGQPGTGDQ
IPWTNNAVTAINQNKSEDQCISSQLDELLCPPTTVEGRNDEKALLEQLVSFLSGKDETELAELDRALGIDKLVQ
GGGLDVLSERFPPQQATPPLMMEERPSLYSQPYSSPSPTANLPSPFQGMVRQKPSLGTMPVQVTPPRGAFSPGM
GMQPRQTLNRPPAAPNQLRLQLQQRLQGQQQLIHQNRQAILNQFAATAPVGINMRSGMQQQITPQPPLNAQMLA
QRQRELYSQQHRQRQLIQQQRAMLMRQQSFGNSLPPSSGLPVQMGNPRLPQGAPQQFPYPPNYGTNPGTPPAST
SPFSQLAANPEASLANRSSMVSRGMTGNMGGQFGTGINPQMQQNVFQYPGAGMVPQGEANFAPSLSPGSSMVPM
PIPPPQSSLLQQTPPASGYQSPDMKAWQQGAIGNNNVFSQAVQNQPTPAQPGVYNNMSITVSMAGGNTNVQNMN
PMMGQMQMSSLQMPGMNTVCPEQINDPALRHTGLYCNQLSSTDLLKTEADGTQDKKTEEFFSVVTTD

>34Pan paniscus|A0A2R9CAU3|Mammalia|SRC1
MSGLGDSSSDPANPDSHKRKGSPCDTLASSTEKRRREQENKYLEELAELLSANISDIDSLSVKPDKCKILKKTV
DQIQLMKRMEQEKSTTDDDVQKSDISSSSQGVIEKESLGPLLLEALDGFFFVVNCEGRIVFVSENVTSYLGYNQ
EELMNTSVYSILHVGDHAEFVKNLLPKSLVNGVPWPQEATRRNSHTFNCRMLIHPPDEPGTENQEACQRYEVMQ
CFTVSQPKSIQEDGEDFQSCLICIARRLPRPPAITGVESFMTKQDTTGKIISIDTSSLRAAGRTGWEDLVRKCI
YAFFQPQGREPSYARQLFQEVMTRGTASSPSYRFILNDGTMLSAHTKCKLCYPQSPDMQPFIMGIHIIDREHSG
LSPQDDTNSGMSIPRVNPSVNPSISPAHGVARSSTLPPSNSNMVSTRINRQQSSDLHSSSHSNSSNSQGSFGCS
PGSQIVANVALNQGQASSQSSNPSLNLNNSPMEGTGISLAQFMSPRRQVTSGLATRPRMPNNSFPPNISTLSSP
VGMTSSACNNNNRSYSNIPVTSLQGMNEGPNNSVGFSASSPVLRQMSSQNSPSRLNIQPAKAESKDNKEIASIL
NEMIQSDNSSSDGKPLDSGLLHNNDRLSDGDSKYSQTSHKLVQLLTTTAEQQLRHADIDTSCKDVLSCTGTSNS
ASANSSGGSCPSSHSSLTERHKILHRLLQEGSPSDITTLSVEPDKKDSASTSVSVTGQVQGNSSIKLELDASKK
KESKDHQLLRYLLDKDEKDLRSTPNLSLDDVKVKVEKKEQMDPCNTNPTPMTKPTPEEIKLEAQSQFTADLDQF
DQLLPTLEKAAQLPGLCETDRMDGAVTSVTIKSEILPASLQSATARPTSRLNRLPELELEAIDNQFGQPGTGDQ
IPWTNNTVTAINQSKSEDQCISSQLDELLCPPTTVEGRNDEKALLEQLVSFLSGKDETELAELDRALGIDKLVQ
GGGLDVLSERFPPQQATPPLIMEERPNLYSQPYSSPSPTANLPSPFQGMVRQKPSLGTMPVQVTPPRGAFSPGM
GMQPRQTLNRPPAAPNQLRLQLQQRLQGQQQLIHQNRQAILNQFAATAPVGINMRSGMQQQITPQPPLNAQMLA
QRQRELYSQQHRQRQLIQQQRAMLMRQQSFGNNLPPSSGLPVQMGNPRLPQGAPQQFPYPPNYGTNPGTPPAST
SPFSQLAANPEASLANRNSMVSRGMTGNMGGQFGTGINPQMQQNVFQYPGAGMVPQGEANFAPSLSPGSSMVPM
PIPPPQSSLLQQTPPASGYQSPDMKAWQQGAIGNNNVFSQAVQNQPTPAQPGVYNNMSITVSMAGGNTNVQNMN
PMMAQMQMSSLQMPGMNTVCPEQINDPALRHTGLYCNQLSSTDLLKTEADGTQQVQQVQVFADVQCTVNLVGGD
PYLNQPGPLGTQKPTSGPQTPQAQQKSLLQQLLTE
>35Pan paniscus|A0A2R9CDJ4|Mammalia|SRC1
MSGLGDSSSDPANPDSHKRKGSPCDTLASSTEKRRREQENKYLEELAELLSANISDIDSLSVKPDKCKILKKTV
DQIQLMKRMEQEKSTTDDDVQKSDISSSSQGVIEKESLGPLLLEALDGFFFVVNCEGRIVFVSENVTSYLGYNQ
EELMNTSVYSILHVGDHAEFVKNLLPKSLVNGVPWPQEATRRNSHTFNCRMLIHPPDEPGTENQEACQRYEVMQ
CFTVSQPKSIQEDGEDFQSCLICIARRLPRPPAITGVESFMTKQDTTGKIISIDTSSLRAAGRTGWEDLVRKCI
YAFFQPQGREPSYARQLFQEVMTRGTASSPSYRFILNDGTMLSAHTKCKLCYPQSPDMQPFIMGIHIIDREHSG
LSPQDDTNSGMSIPRVNPSVNPSISPAHGVARSSTLPPSNSNMVSTRINRQQSSDLHSSSHSNSSNSQGSFGCS
PGSQIVANVALNQGQASSQSSNPSLNLNNSPMEGTGISLAQFMSPRRQVTSGLATRPRMPNNSFPPNISTLSSP
VGMTSSACNNNNRSYSNIPVTSLQGMNEGPNNSVGFSASSPVLRQMSSQNSPSRLNIQPAKAESKDNKEIASIL
NEMIQSDNSSSDGKPLDSGLLHNNDRLSDGDSKYSQTSHKLVQLLTTTAEQQLRHADIDTSCKDVLSCTGTSNS
ASANSSGGSCPSSHSSLTERHKILHRLLQEGSPSDITTLSVEPDKKDSASTSVSVTGQVQGNSSIKLELDASKK
KESKDHQLLRYLLDKDEKDLRSTPNLSLDDVKVKVEKKEQMDPCNTNPTPMTKPTPEEIKLEAQSQFTADLDQF

DQLLPTLEKAAQLPGLCETDRMDGAVTSVTIKSEILPASLQSATARPTSRLNRLPELELEAIDNQFGQPGTGDQ
IPWTNNTVTAINQSKSEDQCISSQLDELLCPPTTVEGRNDEKALLEQLVSFLSGKDETELAELDRALGIDKLVQ
GGGLDVLSERFPPQQATPPLIMEERPNLYSQPYSSPSPTANLPSPFQGMVRQKPSLGTMPVQVTPPRGAFSPGM
GMQPRQTLNRPPAAPNQLRLQLQQRLQGQQQLIHQNRQAILNQFAATAPVGINMRSGMQQQITPQPPLNAQMLA
QRQRELYSQQHRQRQLIQQQRAMLMRQQSFGNNLPPSSGLPVQMGNPRLPQGAPQQFPYPPNYGTNPGTPPAST
SPFSQLAANPEASLANRNSMVSRGMTGNMGGQFGTGINPQMQQNVFQYPGAGMVPQGEANFAPSLSPGSSMVPM
PIPPPQSSLLQQTPPASGYQSPDMKAWQQGAIGNNNVFSQAVQNQPTPAQPGVYNNMSITVSMAGGNTNVQNMN
PMMAQMQMSSLQMPGMNTVCPEQINDPALRHTGLYCNQLSSTDLLKTEADGTQDKKTEEFFSVVTTD
>36Leptonychotes weddellii|A0A2U3YCD8|Mammalia|SRC1
MSGLGDSSSDPANPDSHKRKGSPCDTLASSTEKRRREQENKYLEELAELLSANISDIDSLSVKPDKCKILKKTV
DQIQLMKRMEQEKSTTDDEVQKSDISSSSQGVIEKESLGPLLLEALDGFFFVVNCEGRIVFVSENVTSYLGYNQ
EELMNTSVYSILHVGDHAEFVKNLLPKSLVNGVPWPQEATRRNSHTFNCRMLIHPPDEPGTENQEACQRYEVMQ
CFTVSQPKSIQEDGEDFQSCLICIARRLPRPPTITGVESFMTKQDTTGKIISIDTSSLRAAGRTGWEDLVRKCI
YAFFQPQGREPSYARQLFQEVMTRGTASSPSYRFILNDGTMLSAHTKCKLCYPQSPDMQPFIMGIHIIDREHSG
LSPQDDTNSGMSIPRVNPSVNSSISPAHGVARSSTLPPSSNNMVSPRVNRQQSSDLHSSSHSNSSNSQGSFGCS
PGNQIVASVALNQGQASSQSTNPSLNLNNSPMEGTGISLAQFMSPRRQVSSGLAPRPRMPNNSFPPNIPTLSSP
VVMTGTACNNNNRSYSNIPVTSLQSMNEGPNNSVGFSASSPVLRQMSSQNSPSRLNIQPAKAESKDNKEIASIL
NEMIQSDNSSSDGKPLDSGLLHNNDRLSDGDNKYSQTSHKLVQLLTTTAEQQLRHADIDTSCKEVLSCSGTSGS
ASANSSSGSCPSSHSSLTERHKILHRLLQEGSPSDITTLSVEPDKKDSSSTSVSVTGQGQGSSSVKLELDASKK
KESKDHQLLRYLLDKDEKDLRSTPNLSLDDVKVKVEKKEQMDPCNTNPTPMTKPAPEEIKLESQSQFTADLDQF
DQLLPTLEKAAQLPGLCEPERMDGAVTNVTIKSETLPTSLQSTTARPTSRLNRLPELELEAIDNQFGQPGTGDQ
IPWANNTVTAVNQNKPEDQCISSQLDELLCPPTTVEGRNDEKALLEQLVSFLSGKDETELAELDRALGIDKLVQ
GGGLDVLSERFPPQQATPPLMMEERPNLYSQPYSSPSPTANLSSPFQGMVRQKPSLGTMPVQVTPPRGAFSPGM
GMPPRQTLNRPPAAPNQLRLQLQQRLQGQQQLIHQNRQAILNQFAANAPVGINMRSGMQQQITPQPPLNAQMLA
QRQRELYSQQHRQRQLIQQQRAMLMRQQSFGNNLPPSSGLPVQMGNPRLPQGAPQQFPYPPNYGTNPGTPPAST
SPFSQLAANPESTLANRNSMVNRGMAGNMGGQFGTGINPQMQQNVFQYPGSGMVPQGEANFAPSLSPGSSMVPM
PIPPPQSSLLQPTPPSSGYQSPDMKAWQQGAMGNNNVFSQAVQNQPTPAQPGVYNNMSITVSMAGGNTNVQNIN
PMMGQMQMSSLQMPGMNTVCPEQINDPALRHTGLYCNQLSSTDLLKTETDGTQQVQQVQVFADVQCTVNLVGGD
PYLNQPGPLGTQKPTAGPQTPQAQQKSLLQQLLTE
>37Tursiops truncatus|A0A2U4BZR5|Sarcopterygii|SRC1
MSGLGDSSSDPANPDSHKRKGSPCDTLASSTEKRRREQENKYLEELAELLSANISDIDSLSVKPDKCKILKKTV
DQIQLMKRMEQEKSTTDDEVQKSDISSSSQGVIEKESLGPLLLEALDGFFFVVNCEGRIVFVSENVTSYLGYNQ
EELMNTSVYSILHVGDHAEFVKNLLPKSLVNGVPWPQEATRRNSHTFNCRMLIHPPDEPGTENQEACQRYEVMQ
CFTVSQPKSIQEDGEDFQSCLICIARRLPRPPAITGVESFMTKQDTTGKIISIDTSSLRAAGRTGWEDLVRKCI
YAFFQPQGREPSYARQLFQEVMTRGTASSPSYRFILNDGTMLSAHTKCKLCYPQSPDMQPFIMGIHIIDREHSG
LSPQDDTNPGMSIPRVNPSVNPSISPAHGAARSATLPPSNNNVVSTRVNRQQSSDLHSSSHSNSSNSQGSFGCS
PGNQIVASVALNQGQASSQSTNPSLNLNNSPMEGTGISLAQFMSPRRQVSSGLATRPRMPNNSFPPNIPTLSSP
VSMTSTACNNSNRSYSNIPVTPLQSMNEGPNNSVGFSASSPVLRQMSSQNSPNRLNIQPAKAESKDNKEIASIL
NEMIQSDNSSNDGKPLDSGLLHNSDRLSDGDNKYSQTSHKLVQLLTTTAEQQLRHADIDTSCKEVLSCTGTPSS
ASASSSSGSCPSSHSSLTERHKILHRLLQEGSPSDITTLSVEPDKKDSASTSVAVTGQVQGNSSIKLELDASKK
KESKDHQLLRYLLDKDEKDLRSTPNLSLDDVKVKVEKKEQMDPCNTNPAPMTKPAPEEIKLESQSQFTADLDQF
DQLLPTLEKAAQLPGLCETERMDGAVTTVAIKSEILPAALQTTTARPTSRLNRLPELELEAIDNQFGQPGSGDQ
VPWTNNTVTAVNQNKPEDQCISSQLDELLCPPTTVEGRNDEKALLEQLVSFLSGKDETELAELDRALGIDKLVQ
GGGLDVLSERFPPQQATPPLMMEERPNLYSQPYSSPSPTANLSGPFQGMVRQKPSLGTMPVQVTPPRGAFSPGM
GMQTRQTLNRPPAAPNQLRLQLQQRLQGQQQLIHQNRQAILNQFAANAPVGISMRSGMQQQITPQPPLNAQMLA
QRQRELYSQQHRQRQLIQQQRAMLMRQQSFGNNLPPSSGLPVQMGNPRLPQGAPQQFPYPPNYGTNPGTPPAST
SPFSQLAANPEATLANRNSMVNRGMTGNMGGQFGTGISPQMQQNVFQYPGSGMLPQGEANFSPSLSPGSSMVPM
PIPPPQSSLLQQTPPTSGYQSPDMKAWQQGAMGNNNVFSQAVQNQPTPAQPGVYNNMSITVSMAGGNTNVQNMN
PMMGQMQMSSLQMPGMNTVCPEQINDPALRHTGLYCNQLSSTDLLKTEADGTQDKKTEEFFSVVTTD
>38Tursiops truncatus|A0A2U4BZR9|Sarcopterygii|SRC1
MSGLGDSSSDPANPDSHKRKGSPCDTLASSTEKRRREQENKYLEELAELLSANISDIDSLSVKPDKCKILKKTV
DQIQLMKRMEQEKSTTDDEVQKSDISSSSQGVIEKESLGPLLLEALDGFFFVVNCEGRIVFVSENVTSYLGYNQ
EELMNTSVYSILHVGDHAEFVKNLLPKSLVNGVPWPQEATRRNSHTFNCRMLIHPPDEPGTENQEACQRYEVMQ
CFTVSQPKSIQEDGEDFQSCLICIARRLPRPPAITGVESFMTKQDTTGKIISIDTSSLRAAGRTGWEDLVRKCI

YAFFQPQGREPSYARQLFQEVMTRGTASSPSYRFILNDGTMLSAHTKCKLCYPQSPDMQPFIMGIHIIDREHSG
LSPQDDTNPGMSIPRVNPSVNPSISPAHGAARSATLPPSNNNVVSTRVNRQQSSDLHSSSHSNSSNSQGSFGCS
PGNQIVASVALNQGQASSQSTNPSLNLNNSPMEGTGISLAQFMSPRRQVSSGLATRPRMPNNSFPPNIPTLSSP
VSMTSTACNNSNRSYSNIPVTPLQSMNEGPNNSVGFSASSPVLRQMSSQNSPNRLNIQPAKAESKDNKEIASIL
NEMIQSDNSSNDGKPLDSGLLHNSDRLSDGDNKYSQTSHKLVQLLTTTAEQQLRHADIDTSCKEVLSCTGTPSS
ASASSSSGSCPSSHSSLTERHKILHRLLQEGSPSDITTLSVEPDKKDSASTSVAVTGQVQGNSSIKLELDASKK
KESKDHQLLRYLLDKDEKDLRSTPNLSLDDVKVKVEKKEQMDPCNTNPAPMTKPAPEEIKLESQSQFTADLDQF
DQLLPTLEKAAQLPGLCETERMDGAVTTVAIKSEILPAALQTTTARPTSRLNRLPELELEAIDNQFGQPGSGDQ
VPWTNNTVTAVNQNKPEDQCISSQLDELLCPPTTVEGRNDEKALLEQLVSFLSGKDETELAELDRALGIDKLVQ
GGGLDVLSERFPPQQATPPLMMEERPNLYSQPYSSPSPTANLSGPFQGMVRQKPSLGTMPVQVTPPRGAFSPGM
GMQTRQTLNRPPAAPNQLRLQLQQRLQGQQQLIHQNRQAILNQFAANAPVGISMRSGMQQQITPQPPLNAQMLA
QRQRELYSQQHRQRQLIQQQRAMLMRQQSFGNNLPPSSGLPVQMGNPRLPQGAPQQFPYPPNYGTNPGTPPAST
SPFSQLAANPEATLANRNSMVNRGMTGNMGGQFGTGISPQMQQNVFQYPGSGMLPQGEANFSPSLSPGSSMVPM
PIPPPQSSLLQQTPPTSGYQSPDMKAWQQGAMGNNNVFSQAVQNQPTPAQPGVYNNMSITVSMAGGNTNVQNMN
PMMGQMQMSSLQMPGMNTVCPEQINDPALRHTGLYCNQLSSTDLLKTEADGTQVQQVQVFADVQCTVNLVGGDP
YLNQPGPLGTQKPTAGPQTPQAQQKSLLQQLLTE
>39Tursiops truncatus|A0A2U4BZW4|Sarcopterygii|SRC1
MSGLGDSSSDPANPDSHKRKGSPCDTLASSTEKRRREQENKYLEELAELLSANISDIDSLSVKPDKCKILKKTV
DQIQLMKRMEQEKSTTDDEVQKSDISSSSQGVIEKESLGPLLLEALDGFFFVVNCEGRIVFVSENVTSYLGYNQ
EELMNTSVYSILHVGDHAEFVKNLLPKSLVNGVPWPQEATRRNSHTFNCRMLIHPPDEPGTENQEACQRYEVMQ
CFTVSQPKSIQEDGEDFQSCLICIARRLPRPPAITGVESFMTKQDTTGKIISIDTSSLRAAGRTGWEDLVRKCI
YAFFQPQGREPSYARQLFQEVMTRGTASSPSYRFILNDGTMLSAHTKCKLCYPQSPDMQPFIMGIHIIDREHSG
LSPQDDTNPGMSIPRVNPSVNPSISPAHGAARSATLPPSNNNVVSTRVNRQQSSDLHSSSHSNSSNSQGSFGCS
PGNQIVASVALNQGQASSQSTNPSLNLNNSPMEGTGISLAQFMSPRRQVSSGLATRPRMPNNSFPPNIPTLSSP
VSMTSTACNNSNRSYSNIPVTPLQSMNEGPNNSVGFSASSPVLRQMSSQNSPNRLNIQPAKAESKDNKEIASIL
NEMIQSDNSSNDGKPLDSGLLHNSDRLSDGDNKYSQTSHKLVQLLTTTAEQQLRHADIDTSCKEVLSCTGTPSS
ASASSSSGSCPSSHSSLTERHKILHRLLQEGSPSDITTLSVEPDKKDSASTSVAVTGQVQGNSSIKLELDASKK
KESKDHQLLRYLLDKDEKDLRSTPNLSLDDVKVKVEKKEQMDPCNTNPAPMTKPAPEEIKLESQSQFTADLDQF
DQLLPTLEKAAQLPGLCETERMDGAVTTVAIKSEILPAALQTTTARPTSRLNRLPELELEAIDNQFGQPGSGDQ
VPWTNNTVTAVNQNKPEDQCISSQLDELLCPPTTVEGRNDEKALLEQLVSFLSGKDETELAELDRALGIDKLVQ
GGGLDVLSERFPPQQATPPLMMEERPNLYSQPYSSPSPTANLSGPFQGMVRQKPSLGTMPVQVTPPRGAFSPGM
GMQTRQTLNRPPAAPNQLRLQLQQRLQGQQQLIHQNRQAILNQFAANAPVGISMRSGMQQQITPQPPLNAQMLA
QRQRELYSQQHRQRQLIQQQRAMLMRQQSFGNNLPPSSGLPVQMGNPRLPQGAPQQFPYPPNYGTNPGTPPAST
SPFSQLAANPEATLANRNSMVNRGMTGNMGGQFGTGISPQMQQNVFQYPGSGMLPQGEANFSPSLSPGSSMVPM
PIPPPQSSLLQQTPPTSGYQSPDMKAWQQGAMGNNNVFSQAVQNQPTPAQPGVYNNMSITVSMAGGNTNVQNMN
PMMGQMQMSSLQMPGMNTVCPEQINDPALRHTGLYCNQLSSTDLLKTEADGTQQVQQVQVFADVQCTVNLVGGD
PYLNQPGPLGTQKPTAGPQTPQAQQKSLLQQLLTE
>40Delphinapterus leucas|A0A2Y9N5N9|Mammalia|SRC1
MSGLGDSSSDPANPDSHKRKGSPCDTLASSTEKRRREQENKYLEELAELLSANISDIDSLSVKPDKCKILKKTV
DQIQLMKRMEQEKSTTDDEVQKSDISSSSQGVIEKESLGPLLLEALDGFFFVVNCEGRIVFVSENVTSYLGYNQ
EELMNTSVYSILHVGDHAEFVKNLLPKSLVNGVPWPQEATRRNSHTFNCRMLIHPPDEPGTENQEACQRYEVMQ
CFTVSQPKSIQEDGEDFQSCLICIARRLPRPPAITGVESFMTKQDTTGKIISIDTSSLRAAGRTGWEDLVRKCI
YAFFQPQGREPSYARQLFQEVMTRGTASSPSYRFILNDGTMLSAHTKCKLCYPQSPDMQPFIMGIHIIDREHSG
LSPQDDTNPGMSIPRVNPSVNPSISPAHGAARSATLPPSNNNVVSTRVNRQQSSDLHSSSHSNSSNSQGSFGCS
PGNQIVASVALNQGQASSQSTNPSLNLNNSPMEGTGISLAQFMSPRRQVSSGLATRPRMPNNSFPPNIPTLSSP
VSMTSTACNNSNRSYSNIPVTPLQSMNEGPNNSVGFSASSPVLRQMSSQNSPNRLNIQPAKAESKDNKEIASIL
NEMIQSDNSSNDGKPLDSGLLHNSDRLSDGDNKYSQTSHKLVQLLTTTAEQQLRHADIDTSCKEVLSCTGTPSS
ASASSSSGSCPSSHSSLTERHKILHRLLQEGSPSDITTLSVEPDKKDSASTSVAVTGQVQGNSSIKLELDASKK
KESKDHQLLRYLLDKDEKDLRSTPNLSLDDVKVKVEKKEQMDPCNTNPAPMTKPAPEEIKLESQSQFTADLDQF
DQLLPTLEKAAQLPGLCETERMDGAVTTVAIKSEILPAALQTTTARPTSRLNRLPELELEAIDNQFGQPGSGDQ
VPWTNNTVTAVNQNKPEDQCISSQLDELLCPPTTVEGRNDEKALLEQLVSFLSGKDETELAELDRALGIDKLVQ
GGGLDVLSERFPPQQATPPLMMEERPNLYSQPYSSPSPTANLSGPFQGMVRQKPSLGTMPVQVTPPRGAFSPGM
GMQTRQTLNRPPAAPNQLRLQLQQRLQGQQQLIHQNRQAILNQFAANAPVGISMRSGMQQQITPQPPLNAQMLA
QRQRELYSQQHRQRQLIQQQRAMLMRQQSFGNNLPPSSGLPVQMGNPRLPQGAPQQFPYPPNYGTNPGTPPAST

SPFSQLAANPEATLANRNSMVNRGMTGNMGGQFGTGISPQMQQNVFQYPGSGMVPQGEANFSPSLSPGSSMVPM
PIPPPQSSLLQQTPPTSGYQSPDMKAWQQGAMGNNNVFSQAVQNQPTPAQPGVYNNMSITVSMAGGNTNVQNMN
PMMGQMQMSSLQMPGMNTVCPEQINDPALRHTGLYCNQLSSTDLLKTEADGTQQVQQVQVFADVQCTVNLVGGD
PYLNQPGPLGTQKPTAGPQTPQAQQKSLLQQLLTE

>41Delphinapterus leucas|A0A2Y9N6X8|Mammalia|SRC1
MSGLGDSSSDPANPDSHKRKGSPCDTLASSTEKRRREQENKYLEELAELLSANISDIDSLSVKPDKCKILKKTV
DQIQLMKRMEQEKSTTDDEVQKSDISSSSQGVIEKESLGPLLLEALDGFFFVVNCEGRIVFVSENVTSYLGYNQ
EELMNTSVYSILHVGDHAEFVKNLLPKSLVNGVPWPQEATRRNSHTFNCRMLIHPPDEPGTENQEACQRYEVMQ
CFTVSQPKSIQEDGEDFQSCLICIARRLPRPPAITGVESFMTKQDTTGKIISIDTSSLRAAGRTGWEDLVRKCI
YAFFQPQGREPSYARQLFQEVMTRGTASSPSYRFILNDGTMLSAHTKCKLCYPQSPDMQPFIMGIHIIDREHSG
LSPQDDTNPGMSIPRVNPSVNPSISPAHGAARSATLPPSNNNVVSTRVNRQQSSDLHSSSHSNSSNSQGSFGCS
PGNQIVASVALNQGQASSQSTNPSLNLNNSPMEGTGISLAQFMSPRRQVSSGLATRPRMPNNSFPPNIPTLSSP
VSMTSTACNNSNRSYSNIPVTPLQSMNEGPNNSVGFSASSPVLRQMSSQNSPNRLNIQPAKAESKDNKEIASIL
NEMIQSDNSSNDGKPLDSGLLHNSDRLSDGDNKYSQTSHKLVQLLTTTAEQQLRHADIDTSCKEVLSCTGTPSS
ASASSSSGSCPSSHSSLTERHKILHRLLQEGSPSDITTLSVEPDKKDSASTSVAVTGQVQGNSSIKLELDASKK
KESKDHQLLRYLLDKDEKDLRSTPNLSLDDVKVKVEKKEQMDPCNTNPAPMTKPAPEEIKLESQSQFTADLDQF
DQLLPTLEKAAQLPGLCETERMDGAVTTVAIKSEILPAALQTTTARPTSRLNRLPELELEAIDNQFGQPGSGDQ
VPWTNNTVTAVNQNKPEDQCISSQLDELLCPPTTVEGRNDEKALLEQLVSFLSGKDETELAELDRALGIDKLVQ
GGGLDVLSERFPPQQATPPLMMEERPNLYSQPYSSPSPTANLSGPFQGMVRQKPSLGTMPVQVTPPRGAFSPGM
GMQTRQTLNRPPAAPNQLRLQLQQRLQGQQQLIHQNRQAILNQFAANAPVGISMRSGMQQQITPQPPLNAQMLA
QRQRELYSQQHRQRQLIQQQRAMLMRQQSFGNNLPPSSGLPVQMGNPRLPQGAPQQFPYPPNYGTNPGTPPAST
SPFSQLAANPEATLANRNSMVNRGMTGNMGGQFGTGISPQMQQNVFQYPGSGMVPQGEANFSPSLSPGSSMVPM
PIPPPQSSLLQQTPPTSGYQSPDMKAWQQGAMGNNNVFSQAVQNQPTPAQPGVYNNMSITVSMAGGNTNVQNMN
PMMGQMQMSSLQMPGMNTVCPEQINDPALRHTGLYCNQLSSTDLLKTEADGTQVQQVQVFADVQCTVNLVGGDP
YLNQPGPLGTQKPTAGPQTPQAQQKSLLQQLLTE
>42Delphinapterus leucas|A0A2Y9NAR9|Mammalia|SRC1
MSGLGDSSSDPANPDSHKRKGSPCDTLASSTEKRRREQENKYLEELAELLSANISDIDSLSVKPDKCKILKKTV
DQIQLMKRMEQEKSTTDDEVQKSDISSSSQGVIEKESLGPLLLEALDGFFFVVNCEGRIVFVSENVTSYLGYNQ
EELMNTSVYSILHVGDHAEFVKNLLPKSLVNGVPWPQEATRRNSHTFNCRMLIHPPDEPGTENQEACQRYEVMQ
CFTVSQPKSIQEDGEDFQSCLICIARRLPRPPAITGVESFMTKQDTTGKIISIDTSSLRAAGRTGWEDLVRKCI
YAFFQPQGREPSYARQLFQEVMTRGTASSPSYRFILNDGTMLSAHTKCKLCYPQSPDMQPFIMGIHIIDREHSG
LSPQDDTNPGMSIPRVNPSVNPSISPAHGAARSATLPPSNNNVVSTRVNRQQSSDLHSSSHSNSSNSQGSFGCS
PGNQIVASVALNQGQASSQSTNPSLNLNNSPMEGTGISLAQFMSPRRQVSSGLATRPRMPNNSFPPNIPTLSSP
VSMTSTACNNSNRSYSNIPVTPLQSMNEGPNNSVGFSASSPVLRQMSSQNSPNRLNIQPAKAESKDNKEIASIL
NEMIQSDNSSNDGKPLDSGLLHNSDRLSDGDNKYSQTSHKLVQLLTTTAEQQLRHADIDTSCKEVLSCTGTPSS
ASASSSSGSCPSSHSSLTERHKILHRLLQEGSPSDITTLSVEPDKKDSASTSVAVTGQVQGNSSIKLELDASKK
KESKDHQLLRYLLDKDEKDLRSTPNLSLDDVKVKVEKKEQMDPCNTNPAPMTKPAPEEIKLESQSQFTADLDQF
DQLLPTLEKAAQLPGLCETERMDGAVTTVAIKSEILPAALQTTTARPTSRLNRLPELELEAIDNQFGQPGSGDQ
VPWTNNTVTAVNQNKPEDQCISSQLDELLCPPTTVEGRNDEKALLEQLVSFLSGKDETELAELDRALGIDKLVQ
GGGLDVLSERFPPQQATPPLMMEERPNLYSQPYSSPSPTANLSGPFQGMVRQKPSLGTMPVQVTPPRGAFSPGM
GMQTRQTLNRPPAAPNQLRLQLQQRLQGQQQLIHQNRQAILNQFAANAPVGISMRSGMQQQITPQPPLNAQMLA
QRQRELYSQQHRQRQLIQQQRAMLMRQQSFGNNLPPSSGLPVQMGNPRLPQGAPQQFPYPPNYGTNPGTPPAST
SPFSQLAANPEATLANRNSMVNRGMTGNMGGQFGTGISPQMQQNVFQYPGSGMVPQGEANFSPSLSPGSSMVPM
PIPPPQSSLLQQTPPTSGYQSPDMKAWQQGAMGNNNVFSQAVQNQPTPAQPGVYNNMSITVSMAGGNTNVQNMN
PMMGQMQMSSLQMPGMNTVCPEQINDPALRHTGLYCNQLSSTDLLKTEADGTQDKKTEEFFSVVTTD
>43Physeter macrocephalus|A0A2Y9S762|Mammalia|SRC1
MSGLGDSSSDPANPDSHKRKGSPCDTLASSTEKRRREQENKYLEELAELLSANISDIDSL
SVKPDKCKILKKTVDQIQLMKRMEQEKSATDDEVQKSDISSSSQGVIEKESLGPLLLEAL
DGFFFVVNCEGRIVFVSENVTSYLGYNQEELMNTSVYSILHVGDHAEFVKNLLPKSLVNG
VPWPQEATRRNSHTFNCRMLIHPPDEPGTENQEACQRYEVMQCFTVSQPKSIQEDGEDFQ
SCLICIARRLPRPPAITGVESFMTKQDTTGKIISIDTSSLRAAGRTGWEDLVRKCIYAFF
QPQGREPSYARQLFQEVMTRGTASSPSYRFILNDGTMLSAHTKCKLCYPQSPDMQPFIMG
IHIIDREHSGLSPQDDTNPGMSIPRVNPSVNPSISPAHGAARSATLPPSNNNVVSTRVNR

QQSSDLHSSSHSNSSNSQGSFGCSPGNQIVASVALNQGQASSQSTNPSLNLSNSPMEGTG
ISLAQFMSPRRQVSSGLATRPRMPNNSFPPNIPTLSSPVSMTSTACNNSNRSYSNIPVTP
LQSMNEGPNNSVGFSASSPVLRQMSSQNSPNRLNIQPAKAESKDNKEIASILNEMIQSDN
SSNDGKPLDSGLLHNSDRLSDGDNKYSQTSHKLVQLLTTTAEQQLRHADIDTSCKEVLSC
TGTPSSASASSSSGSCPSSHSSLTERHKILHRLLQEGSPSDITTLSVEPDKKDSASTSVA
VTGQVQGNSSIKLELDASKKKESKDHQLLRYLLDKDEKDLRSTPNLSLDDVKVKVEKKEQ
MDPCNTNPAPMTKPAPEEIKLESQSQFTADLDQFDQLLPTLEKAAQLPGLCETERMDGAV
TTVAIKSEILPAALQTTTARPTSRLNRLPELELEAIDNQFGQPGSGDQVPWTNNTVTAVN
QNKPEDQCISSQLDELLCPPTTVEGRNDEKALLEQLVSFLSGKDETELAELDRALGIDKL
VQGGGLDVLSERFPPQQATPPLMMEERPNLYSQPYSSPSPTANLSSPFQGMVRQKPSLGT
MPVQVTPPRGAFSPGMGMQTRQTLNRPPAAPNQLRLQLQQRLQGQQQLIHQNRQAILNQF
AANAPVGINMRSGMQQQITPQPPLNAQMLAQRQRELYSQQHRQRQLIQQQRAMLMRQQSF
GNNLPPSSGLPVQMGNPRLPQGAPQQFPYPPNYGTNPGTPPASTSPFSQLAANPEATLAN
RNSMVNRGMTGNMGGQFGTGISPQMQQNVFQYPGSGMVPQGEANFSPSLSPGSSMVPMPI
PPPQSSLLQQTPPASGYQSPDMKAWQQGAMGNNNVFSQAVQNQPAPAQPGVYNNMSITVS
MAGGNTNVQNMNPMMGQMQMSSLQMPGMNTVCPEQINDPALRHTGLYCNQLSSTDLLKTE
ADGTQDKKTEEFFSVVTTD
>44Physeter macrocephalus|A0A2Y9SDL3|Mammalia|SRC1
MSGLGDSSSDPANPDSHKRKGSPCDTLASSTEKRRREQENKYLEELAELLSANISDIDSL
SVKPDKCKILKKTVDQIQLMKRMEQEKSATDDEVQKSDISSSSQGVIEKESLGPLLLEAL
DGFFFVVNCEGRIVFVSENVTSYLGYNQEELMNTSVYSILHVGDHAEFVKNLLPKSLVNG
VPWPQEATRRNSHTFNCRMLIHPPDEPGTENQEACQRYEVMQCFTVSQPKSIQEDGEDFQ
SCLICIARRLPRPPAITGVESFMTKQDTTGKIISIDTSSLRAAGRTGWEDLVRKCIYAFF
QPQGREPSYARQLFQEVMTRGTASSPSYRFILNDGTMLSAHTKCKLCYPQSPDMQPFIMG
IHIIDREHSGLSPQDDTNPGMSIPRVNPSVNPSISPAHGAARSATLPPSNNNVVSTRVNR
QQSSDLHSSSHSNSSNSQGSFGCSPGNQIVASVALNQGQASSQSTNPSLNLSNSPMEGTG
ISLAQFMSPRRQVSSGLATRPRMPNNSFPPNIPTLSSPVSMTSTACNNSNRSYSNIPVTP
LQSMNEGPNNSVGFSASSPVLRQMSSQNSPNRLNIQPAKAESKDNKEIASILNEMIQSDN
SSNDGKPLDSGLLHNSDRLSDGDNKYSQTSHKLVQLLTTTAEQQLRHADIDTSCKEVLSC
TGTPSSASASSSSGSCPSSHSSLTERHKILHRLLQEGSPSDITTLSVEPDKKDSASTSVA
VTGQVQGNSSIKLELDASKKKESKDHQLLRYLLDKDEKDLRSTPNLSLDDVKVKVEKKEQ
MDPCNTNPAPMTKPAPEEIKLESQSQFTADLDQFDQLLPTLEKAAQLPGLCETERMDGAV
TTVAIKSEILPAALQTTTARPTSRLNRLPELELEAIDNQFGQPGSGDQVPWTNNTVTAVN
QNKPEDQCISSQLDELLCPPTTVEGRNDEKALLEQLVSFLSGKDETELAELDRALGIDKL
VQGGGLDVLSERFPPQQATPPLMMEERPNLYSQPYSSPSPTANLSSPFQGMVRQKPSLGT
MPVQVTPPRGAFSPGMGMQTRQTLNRPPAAPNQLRLQLQQRLQGQQQLIHQNRQAILNQF
AANAPVGINMRSGMQQQITPQPPLNAQMLAQRQRELYSQQHRQRQLIQQQRAMLMRQQSF
GNNLPPSSGLPVQMGNPRLPQGAPQQFPYPPNYGTNPGTPPASTSPFSQLAANPEATLAN
RNSMVNRGMTGNMGGQFGTGISPQMQQNVFQYPGSGMVPQGEANFSPSLSPGSSMVPMPI
PPPQSSLLQQTPPASGYQSPDMKAWQQGAMGNNNVFSQAVQNQPAPAQPGVYNNMSITVS
MAGGNTNVQNMNPMMGQMQMSSLQMPGMNTVCPEQINDPALRHTGLYCNQLSSTDLLKTE
ADGTQVQQVQVFADVQCTVNLVGGDPYLNQPGPLGTQKPTAGPQTPQAQQKSLLQQLLTE

>45Physeter macrocephalus|A0A2Y9SF15|Mammalia|SRC1
MSGLGDSSSDPANPDSHKRKGSPCDTLASSTEKRRREQENKYLEELAELLSANISDIDSLSVKPDKCKILKKTV
DQIQLMKRMEQEKSATDDEVQKSDISSSSQGVIEKESLGPLLLEALDGFFFVVNCEGRIVFVSENVTSYLGYNQ
EELMNTSVYSILHVGDHAEFVKNLLPKSLVNGVPWPQEATRRNSHTFNCRMLIHPPDEPGTENQEACQRYEVMQ
CFTVSQPKSIQEDGEDFQSCLICIARRLPRPPAITGVESFMTKQDTTGKIISIDTSSLRAAGRTGWEDLVRKCI
YAFFQPQGREPSYARQLFQEVMTRGTASSPSYRFILNDGTMLSAHTKCKLCYPQSPDMQPFIMGIHIIDREHSG
LSPQDDTNPGMSIPRVNPSVNPSISPAHGAARSATLPPSNNNVVSTRVNRQQSSDLHSSSHSNSSNSQGSFGCS
PGNQIVASVALNQGQASSQSTNPSLNLSNSPMEGTGISLAQFMSPRRQVSSGLATRPRMPNNSFPPNIPTLSSP
VSMTSTACNNSNRSYSNIPVTPLQSMNEGPNNSVGFSASSPVLRQMSSQNSPNRLNIQPAKAESKDNKEIASIL
NEMIQSDNSSNDGKPLDSGLLHNSDRLSDGDNKYSQTSHKLVQLLTTTAEQQLRHADIDTSCKEVLSCTGTPSS
ASASSSSGSCPSSHSSLTERHKILHRLLQEGSPSDITTLSVEPDKKDSASTSVAVTGQVQGNSSIKLELDASKK

KESKDHQLLRYLLDKDEKDLRSTPNLSLDDVKVKVEKKEQMDPCNTNPAPMTKPAPEEIKLESQSQFTADLDQF
DQLLPTLEKAAQLPGLCETERMDGAVTTVAIKSEILPAALQTTTARPTSRLNRLPELELEAIDNQFGQPGSGDQ
VPWTNNTVTAVNQNKPEDQCISSQLDELLCPPTTVEGRNDEKALLEQLVSFLSGKDETELAELDRALGIDKLVQ
GGGLDVLSERFPPQQATPPLMMEERPNLYSQPYSSPSPTANLSSPFQGMVRQKPSLGTMPVQVTPPRGAFSPGM
GMQTRQTLNRPPAAPNQLRLQLQQRLQGQQQLIHQNRQAILNQFAANAPVGINMRSGMQQQITPQPPLNAQMLA
QRQRELYSQQHRQRQLIQQQRAMLMRQQSFGNNLPPSSGLPVQMGNPRLPQGAPQQFPYPPNYGTNPGTPPAST
SPFSQLAANPEATLANRNSMVNRGMTGNMGGQFGTGISPQMQQNVFQYPGSGMVPQGEANFSPSLSPGSSMVPM
PIPPPQSSLLQQTPPASGYQSPDMKAWQQGAMGNNNVFSQAVQNQPAPAQPGVYNNMSITVSMAGGNTNVQNMN
PMMGQMQMSSLQMPGMNTVCPEQINDPALRHTGLYCNQLSSTDLLKTEADGTQQVQQVQVFADVQCTVNLVGGD
PYLNQPGPLGTQKPTAGPQTPQAQQKSLLQQLLTE

>46Felis catus|A0A337S0K3|Mammalia|SRC1
MSGLGDSSSDPANPDSHKRKGSPCDTLASSTEKRRREQENKYLEELAELLSANISDIDSLSVKPDKCKILKKTV
DQIQLMKRMEQEKSTTDDEVQKSDISSSSQGVIEKESLGPLLLEALDGFFFVVNCEGRIVFVSENVTSYLGYNQ
EELMNTSVYSILHVGDHAEFVKNLLPKSLVNGVPWPQEATRRNSHTFNCRMLIHPPDEPGTENQEACQRYEVMQ
CFTVSQPKSIQEDGEDFQSCLICIARRLPRPPAITGVESFMTKQDTTGKIISIDTSSLRAAGRTGWEDLVRKCI
YAFFQPQGREPSYARQLFQEVMTRGTASSPSYRFILNDGTMLSAHTKCKLCYPQSPDMQPFIMGIHIIDREHSG
LSPQDDTNSGMSISRVNPSVNSSISPAHGVARSSTLPPSSNNMVSPRVNRQQSSDLHSSSHSNSSNSQGSFGCS
PGNQIVASVALNQGQASSQSTNPSLNLNNSPMEGTGISLAQFMSPRRQVSSGLATRPRMPNNSFPPNIPTLSSP
VGMTGTTCNNSNRSYSNIPVTSLQSMNEGPNNSVGFSASSPVLRQMSSQNSPSRLNIQPTKAESKDNKEIASIL
NEMIQSDNSSSDGKPLDSGLLHNNDRLSDGDNKYSQTSHKLVQLLTTTAEQQLRHADIDTSCKEVLSCSGTSGS
ASANSSSGSCPSSHSSLTERHKILHRLLQEGSPSDITTLSVEPDKKDSASTSVSVTGQVQGNSSVKLELDASKK
KESKDHQLLRYLLDKDEKDIRSTPNLSLDDVKVKVEKKEQMDPCNTNPTPMTKPAPEEIKLESQSQFTADLDQF
DQLLPTLEKAAQLPGLCEPERMDGAVTNVTIKSETLPASLQSTTARPTSRLNRLPELELEAIDNQFGQPGTGDQ
IPWANNTVTTVNQNKPEDQCISSQLDELLCPPTTVEGRNDEKALLEQLVSFLSGKDETELAELDRALGIDKLVQ
GGGLDVLSERFPPQQATPPLMMDERPNLYSQPYSSPSPTANLSSPFQGMVRQKPSLGTMPVQVTPPRGAFSPSM
GMQPRQTLNRPPAAPNQLRLQLQQRLQGQQQLIHQNRQAILNQFAANTPVGINMRSGMQQQITPQPPLNAQMLA
QRQRELYSQQHRQRQLIQQQRAMLMRQQSFGNNLPPSSGLPVQMGNPRLPQGAPQQFPYPPNYGTNPGTPPAST
SPFSQLAANPESTLANRNSMVNRGMTGNMGGQFGTGINPQMQQNVFQYPGSGMVPQGEASFAPSLSPGSSMVPM
PIPPPQSSLLQQTPPTSGYQSPDMKAWQQGAMGNNNVFSQAVQSQPTPAQPGVYNNMSITVSMAGGNTNVQNMN
PMMGQMQMSSLQMPGMNTVCPEQINDPALRHTGLYCNQLSSTDLLKTETDGTQQVQQVQSLASYLDEGFWRRTK
TTSSKGKMKPRPCWLSPDPDEEEGPKGPVGLCLCPSTKGCHVCLNQQPSPSRPQAACVYNQLLLATLYLLASRE
YFASHMYPFSFF
>47Felis catus|A0A337SGE9|Mammalia|SRC1
MSGLGDSSSDPANPDSHKRKGSPCDTLASSTEKRRREQENKYLEELAELLSANISDIDSLSVKPDKCKILKKTV
DQIQLMKRMEQEKSTTDDEVQKSDISSSSQGVIEKESLGPLLLEALDGFFFVVNCEGRIVFVSENVTSYLGYNQ
EELMNTSVYSILHVGDHAEFVKNLLPKSLVNGVPWPQEATRRNSHTFNCRMLIHPPDEPGTENQEACQRYEVMQ
CFTVSQPKSIQEDGEDFQSCLICIARRLPRPPAITGVESFMTKQDTTGKIISIDTSSLRAAGRTGWEDLVRKCI
YAFFQPQGREPSYARQLFQEVMTRGTASSPSYRFILNDGTMLSAHTKCKLCYPQSPDMQPFIMGIHIIDREHSG
LSPQDDTNSGMSISRVNPSVNSSISPAHGVARSSTLPPSSNNMVSPRVNRQQSSDLHSSSHSNSSNSQGSFGCS
PGNQIVASVALNQGQASSQSTNPSLNLNNSPMEGTGISLAQFMSPRRQVSSGLATRPRMPNNSFPPNIPTLSSP
VGMTGTTCNNSNRSYSNIPVTSLQSMNEGPNNSVGFSASSPVLRQMSSQNSPSRLNIQPTKAESKDNKEIASIL
NEMIQSDNSSSDGKPLDSGLLHNNDRLSDGDNKYSQTSHKLVQLLTTTAEQQLRHADIDTSCKEVLSCSGTSGS
ASANSSSGSCPSSHSSLTERHKILHRLLQEGSPSDITTLSVEPDKKDSASTSVSVTGQVQGNSSVKLELDASKK
KESKDHQLLRYLLDKDEKDIRSTPNLSLDDVKVKVEKKEQMDPCNTNPTPMTKPAPEEIKLESQSQFTADLDQF
DQLLPTLEKAAQLPGLCEPERMDGAVTNVTIKSETLPASLQSTTARPTSRLNRLPELELEAIDNQFGQPGTGDQ
IPWANNTVTTVNQNKPEDQCISSQLDELLCPPTTVEGRNDEKALLEQLVSFLSGKDETELAELDRALGIDKLVQ
GGGLDVLSERFPPQQATPPLMMDERPNLYSQPYSSPSPTANLSSPFQGMVRQKPSLGTMPVQVTPPRGAFSPSM
GMQPRQTLNRPPAAPNQLRLQLQQRLQGQQQPPLNAQMLAQRQRELYSQQHRQRQLIQQQRAMLMRQQSFGNNL
PPSSGLPVQMGNPRLPQGAPQQFPYPPNYGTNPGTPPASTSPFSQLAANPESTLANRNSMVNRGMTGNMGGQFG
TGINPQMQQNVFQYPGSGMVPQGEASFAPSLSPGSSMVPMPIPPPQSSLLQQTPPTSGYQSPDMKAWQQGAMGN
NNVFSQAVQSQPTPAQPGVYNNMSITVSMAGGNTNVQNMNPMMGQMQMSSLQMPGMNTVCPEQINDPALRHTGL
YCNQLSSTDLLKTETDGTQQVQQVQVFADVQCTVNLVGGDPYLNQPGPLGTQKPTAGPQTPQAQQKSLLQQLLT
E

>48Lipotes vexillifer|A0A340X1V7|Mammalia|SRC1
MSGLGDSSSDPANPDSHKRKGSPCDTLASSTEKRRREQENKYLEELAELLSANISDIDSLSVKPDKCKILKKTV
DQIQLMKRMEQEKSTTDDEVQKSDISSSSQGVIEKESLGPLLLEALDGFFFVVNCEGRIVFVSENVTSYLGYNQ
EELMNTSVYSILHVGDHAEFVKNLLPKSLVNGVPWPQEATRRNSHTFNCRMLIHPPDEPGTENQEACQRYEVMQ
CFTVSQPKSIQEDGEDFQSCLICIARRLPRPPAITGVESFMTKQDTTGKIISIDTSSLRAAGRTGWEDLVRKCI
YAFFQPQGREPSYARQLFQEVMTRGTASSPSYRFILNDGTMLSAHTKCKLCYPQSPDMQPFIMGIHIIDREHSG
LSPQDDTNPGMSIPRVNPSVNPSISPAHGAARSATLPPSNNVVSTRVNRQQSSDLHSSSHSNSSNSQGSFGCSP
GNQIVASVALNQGQASSQSTNPSLNLNNSPMEGTGISLAQFMSPRRQVGSGLAARPRMPNNSFPPNIPTLSSPV
SMTSTACNNSNRSYSNIPVTPLQSMNEGPNNSIGFSASSPVLRQMSSQNSPNRLNIQPAKAESKDNKEIASILN
EMIQSDNSSNDGKPLDSGLLHNSDRLSDGDNKYSQTSHKLVQLLTTTAEQQLRHADIDTSCKEVLSCTGTPSSA
SASSSSGSCPSSHSSLTERHKILHRLLQEGSPSDITTLSVEPDKKDSASTSVAVTGQVQGNSSIKLELDASKKK
ESKDHQLLRYLLDKDEKDLRSTPNLSLDDVKVKVEKKEQMDPCNTNPAPMTKPAPEEIKLESQSQFTADLDQFD
QLLPTLEKAAQLPGLCETERMDGAVTTVAIKSEILPAALQTTTARPTSRLNRLPELELEAIDNQFGQPGSGDQV
PWTNNTVTAVNQNKPEDQCISSQLDELLCPPTTVEGRNDEKALLEQLVSFLSGKDETELAELDRALGIDKLVQG
GGLDVLSERFPPQQATPPLMMEERPNLYSQPYSSPSPTANLSSPFQGMVRQKPSLGTMPVQVTPPRGAFSPGMG
MQTRQTLNRPPAAPNQLRLQLQQRLQGQQQLIHQNRQAILNQFAANAPVGISMRSGMQQQITPQPPLNAQMLAQ
RQRELYSQQHRQRQLIQQQRAMLMRQQSFGNNLPPSSGLPVQMGNPRLPQGAPQQFPYPPNYGTNPGTPPASTS
PFSQLAANPEATLANRNSMVNRGMTGNMGGQFGTGISPQMQQNVFQYPGSGMVPQGEANFSPSLSPGSSMVPMP
IPPPQSSLLQQTPPTSGYQSPDMKAWQQGAMGNNNVFSQAVQNQPTPAQPGVYNNMSITVSMAGGNTNVQNMNP
MMGQMQMSSLQMPGMNTVCPEQINDPALRHTGLYCNQLSSTDLLKTEADGTQQVQQVQVFADVQCTVNLVGGDP
YLNQPGPLGTQKPTAGPQTPQAQQKSLLQQLLTE
>49Lipotes vexillifer|A0A340X8L1|Mammalia|SRC1
MSGLGDSSSDPANPDSHKRKGSPCDTLASSTEKRRREQENKYLEELAELLSANISDIDSLSVKPDKCKILKKTV
DQIQLMKRMEQEKSTTDDEVQKSDISSSSQGVIEKESLGPLLLEALDGFFFVVNCEGRIVFVSENVTSYLGYNQ
EELMNTSVYSILHVGDHAEFVKNLLPKSLVNGVPWPQEATRRNSHTFNCRMLIHPPDEPGTENQEACQRYEVMQ
CFTVSQPKSIQEDGEDFQSCLICIARRLPRPPAITGVESFMTKQDTTGKIISIDTSSLRAAGRTGWEDLVRKCI
YAFFQPQGREPSYARQLFQEVMTRGTASSPSYRFILNDGTMLSAHTKCKLCYPQSPDMQPFIMGIHIIDREHSG
LSPQDDTNPGMSIPRVNPSVNPSISPAHGAARSATLPPSNNVVSTRVNRQQSSDLHSSSHSNSSNSQGSFGCSP
GNQIVASVALNQGQASSQSTNPSLNLNNSPMEGTGISLAQFMSPRRQVGSGLAARPRMPNNSFPPNIPTLSSPV
SMTSTACNNSNRSYSNIPVTPLQSMNEGPNNSIGFSASSPVLRQMSSQNSPNRLNIQPAKAESKDNKEIASILN
EMIQSDNSSNDGKPLDSGLLHNSDRLSDGDNKYSQTSHKLVQLLTTTAEQQLRHADIDTSCKEVLSCTGTPSSA
SASSSSGSCPSSHSSLTERHKILHRLLQEGSPSDITTLSVEPDKKDSASTSVAVTGQVQGNSSIKLELDASKKK
ESKDHQLLRYLLDKDEKDLRSTPNLSLDDVKVKVEKKEQMDPCNTNPAPMTKPAPEEIKLESQSQFTADLDQFD
QLLPTLEKAAQLPGLCETERMDGAVTTVAIKSEILPAALQTTTARPTSRLNRLPELELEAIDNQFGQPGSGDQV
PWTNNTVTAVNQNKPEDQCISSQLDELLCPPTTVEGRNDEKALLEQLVSFLSGKDETELAELDRALGIDKLVQG
GGLDVLSERFPPQQATPPLMMEERPNLYSQPYSSPSPTANLSSPFQGMVRQKPSLGTMPVQVTPPRGAFSPGMG
MQTRQTLNRPPAAPNQLRLQLQQRLQGQQQLIHQNRQAILNQFAANAPVGISMRSGMQQQITPQPPLNAQMLAQ
RQRELYSQQHRQRQLIQQQRAMLMRQQSFGNNLPPSSGLPVQMGNPRLPQGAPQQFPYPPNYGTNPGTPPASTS
PFSQLAANPEATLANRNSMVNRGMTGNMGGQFGTGISPQMQQNVFQYPGSGMVPQGEANFSPSLSPGSSMVPMP
IPPPQSSLLQQTPPTSGYQSPDMKAWQQGAMGNNNVFSQAVQNQPTPAQPGVYNNMSITVSMAGGNTNVQNMNP
MMGQMQMSSLQMPGMNTVCPEQINDPALRHTGLYCNQLSSTDLLKTEADGTQVQQVQVFADVQCTVNLVGGDPY
LNQPGPLGTQKPTAGPQTPQAQQKSLLQQLLTE

>50Lipotes vexillifer|A0A340X968|Mammalia|SRC1
MSGLGDSSSDPANPDSHKRKGSPCDTLASSTEKRRREQENKYLEELAELLSANISDIDSLSVKPDKCKILKKTV
DQIQLMKRMEQEKSTTDDEVQKSDISSSSQGVIEKESLGPLLLEALDGFFFVVNCEGRIVFVSENVTSYLGYNQ
EELMNTSVYSILHVGDHAEFVKNLLPKSLVNGVPWPQEATRRNSHTFNCRMLIHPPDEPGTENQEACQRYEVMQ
CFTVSQPKSIQEDGEDFQSCLICIARRLPRPPAITGVESFMTKQDTTGKIISIDTSSLRAAGRTGWEDLVRKCI
YAFFQPQGREPSYARQLFQEVMTRGTASSPSYRFILNDGTMLSAHTKCKLCYPQSPDMQPFIMGIHIIDREHSG
LSPQDDTNPGMSIPRVNPSVNPSISPAHGAARSATLPPSNNVVSTRVNRQQSSDLHSSSHSNSSNSQGSFGCSP
GNQIVASVALNQGQASSQSTNPSLNLNNSPMEGTGISLAQFMSPRRQVGSGLAARPRMPNNSFPPNIPTLSSPV
SMTSTACNNSNRSYSNIPVTPLQSMNEGPNNSIGFSASSPVLRQMSSQNSPNRLNIQPAKAESKDNKEIASILN
EMIQSDNSSNDGKPLDSGLLHNSDRLSDGDNKYSQTSHKLVQLLTTTAEQQLRHADIDTSCKEVLSCTGTPSSA
SASSSSGSCPSSHSSLTERHKILHRLLQEGSPSDITTLSVEPDKKDSASTSVAVTGQVQGNSSIKLELDASKKK

ESKDHQLLRYLLDKDEKDLRSTPNLSLDDVKVKVEKKEQMDPCNTNPAPMTKPAPEEIKLESQSQFTADLDQFD
QLLPTLEKAAQLPGLCETERMDGAVTTVAIKSEILPAALQTTTARPTSRLNRLPELELEAIDNQFGQPGSGDQV
PWTNNTVTAVNQNKPEDQCISSQLDELLCPPTTVEGRNDEKALLEQLVSFLSGKDETELAELDRALGIDKLVQG
GGLDVLSERFPPQQATPPLMMEERPNLYSQPYSSPSPTANLSSPFQGMVRQKPSLGTMPVQVTPPRGAFSPGMG
MQTRQTLNRPPAAPNQLRLQLQQRLQGQQQLIHQNRQAILNQFAANAPVGISMRSGMQQQITPQPPLNAQMLAQ
RQRELYSQQHRQRQLIQQQRAMLMRQQSFGNNLPPSSGLPVQMGNPRLPQGAPQQFPYPPNYGTNPGTPPASTS
PFSQLAANPEATLANRNSMVNRGMTGNMGGQFGTGISPQMQQNVFQYPGSGMVPQGEANFSPSLSPGSSMVPMP
IPPPQSSLLQQTPPTSGYQSPDMKAWQQGAMGNNNVFSQAVQNQPTPAQPGVYNNMSITVSMAGGNTNVQNMNP
MMGQMQMSSLQMPGMNTVCPEQINDPALRHTGLYCNQLSSTDLLKTEADGTQDKKTEEFFSVVTTD
>51Neophocaena asiaeorientalis asiaeorientalis|A0A341CZR1|Mammalia|SRC1
MSGLGDSSSDPANPDSHKRKGSPCDTLASSTEKRRREQENKYLEELAELLSANISDIDSLSVKPDKCKILKKTV
DQIQLMKRMEQEKSTTDDEVQKSDISSSSQGVIEKESLGPLLLEALDGFFFVVNCEGRIVFVSENVTSYLGYNQ
EELMNTSVYSILHVGDHAEFVKNLLPKSLVNGVPWPQEATRRNSHTFNCRMLIHPPDEPGTENQEACQRYEVMQ
CFTVSQPKSIQEDGEDFQSCLICIARRLPRPPAITGVESFMTKQDTTGKIISIDTSSLRAAGRTGWEDLVRKCI
YAFFQPQGREPSYARQLFQEVMTRGTASSPSYRFILNDGTMLSAHTKCKLCYPQSPDMQPFIMGIHIIDREHSG
LSPQDDTNPGMSIPRVNPSVNPSISPAHGAARSATLPPSNNNVVSTRVNRQQSSDLHSSSHSNSSNSQGSFGCS
PGNQIVASVALNQGQANSQSTNPSLNLNNSPMEGTGISLAQFMSPRRQVTSGLATRPRMPNNSFPPNIPTLSSP
VSMTSTACNNSNRSYSNIPVTPLQSMNEGPNNSVGFSASSPVLRQMSSQNSPNRLNIQPAKAESKDNKEIASIL
NEMIQSDNSSNDGKPLDSGLLHNSDRLSDGDNKYSQTSHKLVQLLTTTAEQQLRHADIDTSCKEVLSCTGTPSS
ASASSSSGSCPSSHSSLTERHKILHRLLQEGSPSDITTLSVEPDKKDSASTSVAVTGQVQGNSSIKLELDASKK
KESKDHQLLRYLLDKDEKDLRSTPNLSLDDVKVKVEKKEQMDPCNTNPAPMTKPAPEEIKLESQSQFTADLDQF
DQLLPTLEKAAQLPGLCETERMDGAVTTVAIKSEILPAALQTTTARPTSRLNRLPELELEAIDNQFGQPGSGDQ
VPWTNNTVTAVNQNKPEDQCISSQLDELLCPPTTVEGRNDEKALLEQLVSFLSGKDETELAELDRALGIDKLVQ
GGGLDVLSERFPPQQATPPLMMEERPNLYSQPYSSPSPTANLSGPFQGMVRQKPSLGTMPVQVTPPRGAFSPGM
GMQTRQTLNRPPAAPNQLRLQLQQRLQGQQQLIHQNRQAILNQFAANAPVGISMRSGMQQQITPQPPLNAQMLA
QRQRELYSQQHRQRQLIQQQRAMLMRQQSFGNNLPPSSGLPVQMGNPRLPQGAPQQFPYPPNYGTNPGTPPAST
SPFSQLAANPEATLANRNSMVNRGMTGNMGGQFGTGISPQMQQNVFQYPGSGMVPQGEANFSPSLSPGSSMVPM
PIPPPQSSLLQQTPPTSGYQSPDMKAWQQGAMGNNNVFSQAVQNQPTPAQPGVYNNMSITVSMAGGNTNVQNMN
PMMGQMQMSSLQMPGMNTVCPEQINDPALRHTGLYCNQLSSTDLLKTEADGTQQVQQVQVFADVQCTVNLVGGD
PYLNQPGPLGTQKPTAGPQTPQAQQKSLLQQLLTE
>52Neophocaena asiaeorientalis asiaeorientalis|A0A341CZX1|Mammalia|SRC1
MSGLGDSSSDPANPDSHKRKGSPCDTLASSTEKRRREQENKYLEELAELLSANISDIDSLSVKPDKCKILKKTV
DQIQLMKRMEQEKSTTDDEVQKSDISSSSQGVIEKESLGPLLLEALDGFFFVVNCEGRIVFVSENVTSYLGYNQ
EELMNTSVYSILHVGDHAEFVKNLLPKSLVNGVPWPQEATRRNSHTFNCRMLIHPPDEPGTENQEACQRYEVMQ
CFTVSQPKSIQEDGEDFQSCLICIARRLPRPPAITGVESFMTKQDTTGKIISIDTSSLRAAGRTGWEDLVRKCI
YAFFQPQGREPSYARQLFQEVMTRGTASSPSYRFILNDGTMLSAHTKCKLCYPQSPDMQPFIMGIHIIDREHSG
LSPQDDTNPGMSIPRVNPSVNPSISPAHGAARSATLPPSNNNVVSTRVNRQQSSDLHSSSHSNSSNSQGSFGCS
PGNQIVASVALNQGQANSQSTNPSLNLNNSPMEGTGISLAQFMSPRRQVTSGLATRPRMPNNSFPPNIPTLSSP
VSMTSTACNNSNRSYSNIPVTPLQSMNEGPNNSVGFSASSPVLRQMSSQNSPNRLNIQPAKAESKDNKEIASIL
NEMIQSDNSSNDGKPLDSGLLHNSDRLSDGDNKYSQTSHKLVQLLTTTAEQQLRHADIDTSCKEVLSCTGTPSS
ASASSSSGSCPSSHSSLTERHKILHRLLQEGSPSDITTLSVEPDKKDSASTSVAVTGQVQGNSSIKLELDASKK
KESKDHQLLRYLLDKDEKDLRSTPNLSLDDVKVKVEKKEQMDPCNTNPAPMTKPAPEEIKLESQSQFTADLDQF
DQLLPTLEKAAQLPGLCETERMDGAVTTVAIKSEILPAALQTTTARPTSRLNRLPELELEAIDNQFGQPGSGDQ
VPWTNNTVTAVNQNKPEDQCISSQLDELLCPPTTVEGRNDEKALLEQLVSFLSGKDETELAELDRALGIDKLVQ
GGGLDVLSERFPPQQATPPLMMEERPNLYSQPYSSPSPTANLSGPFQGMVRQKPSLGTMPVQVTPPRGAFSPGM
GMQTRQTLNRPPAAPNQLRLQLQQRLQGQQQLIHQNRQAILNQFAANAPVGISMRSGMQQQITPQPPLNAQMLA
QRQRELYSQQHRQRQLIQQQRAMLMRQQSFGNNLPPSSGLPVQMGNPRLPQGAPQQFPYPPNYGTNPGTPPAST
SPFSQLAANPEATLANRNSMVNRGMTGNMGGQFGTGISPQMQQNVFQYPGSGMVPQGEANFSPSLSPGSSMVPM
PIPPPQSSLLQQTPPTSGYQSPDMKAWQQGAMGNNNVFSQAVQNQPTPAQPGVYNNMSITVSMAGGNTNVQNMN
PMMGQMQMSSLQMPGMNTVCPEQINDPALRHTGLYCNQLSSTDLLKTEADGTQDKKTEEFFSVVTTD
>53Neophocaena asiaeorientalis asiaeorientalis|A0A341D0Q2|Mammalia|SRC1
MSGLGDSSSDPANPDSHKRKGSPCDTLASSTEKRRREQENKYLEELAELLSANISDIDSLSVKPDKCKILKKTV
DQIQLMKRMEQEKSTTDDEVQKSDISSSSQGVIEKESLGPLLLEALDGFFFVVNCEGRIVFVSENVTSYLGYNQ
EELMNTSVYSILHVGDHAEFVKNLLPKSLVNGVPWPQEATRRNSHTFNCRMLIHPPDEPGTENQEACQRYEVMQ

CFTVSQPKSIQEDGEDFQSCLICIARRLPRPPAITGVESFMTKQDTTGKIISIDTSSLRAAGRTGWEDLVRKCI
YAFFQPQGREPSYARQLFQEVMTRGTASSPSYRFILNDGTMLSAHTKCKLCYPQSPDMQPFIMGIHIIDREHSG
LSPQDDTNPGMSIPRVNPSVNPSISPAHGAARSATLPPSNNNVVSTRVNRQQSSDLHSSSHSNSSNSQGSFGCS
PGNQIVASVALNQGQANSQSTNPSLNLNNSPMEGTGISLAQFMSPRRQVTSGLATRPRMPNNSFPPNIPTLSSP
VSMTSTACNNSNRSYSNIPVTPLQSMNEGPNNSVGFSASSPVLRQMSSQNSPNRLNIQPAKAESKDNKEIASIL
NEMIQSDNSSNDGKPLDSGLLHNSDRLSDGDNKYSQTSHKLVQLLTTTAEQQLRHADIDTSCKEVLSCTGTPSS
ASASSSSGSCPSSHSSLTERHKILHRLLQEGSPSDITTLSVEPDKKDSASTSVAVTGQVQGNSSIKLELDASKK
KESKDHQLLRYLLDKDEKDLRSTPNLSLDDVKVKVEKKEQMDPCNTNPAPMTKPAPEEIKLESQSQFTADLDQF
DQLLPTLEKAAQLPGLCETERMDGAVTTVAIKSEILPAALQTTTARPTSRLNRLPELELEAIDNQFGQPGSGDQ
VPWTNNTVTAVNQNKPEDQCISSQLDELLCPPTTVEGRNDEKALLEQLVSFLSGKDETELAELDRALGIDKLVQ
GGGLDVLSERFPPQQATPPLMMEERPNLYSQPYSSPSPTANLSGPFQGMVRQKPSLGTMPVQVTPPRGAFSPGM
GMQTRQTLNRPPAAPNQLRLQLQQRLQGQQQLIHQNRQAILNQFAANAPVGISMRSGMQQQITPQPPLNAQMLA
QRQRELYSQQHRQRQLIQQQRAMLMRQQSFGNNLPPSSGLPVQMGNPRLPQGAPQQFPYPPNYGTNPGTPPAST
SPFSQLAANPEATLANRNSMVNRGMTGNMGGQFGTGISPQMQQNVFQYPGSGMVPQGEANFSPSLSPGSSMVPM
PIPPPQSSLLQQTPPTSGYQSPDMKAWQQGAMGNNNVFSQAVQNQPTPAQPGVYNNMSITVSMAGGNTNVQNMN
PMMGQMQMSSLQMPGMNTVCPEQINDPALRHTGLYCNQLSSTDLLKTEADGTQVQQVQVFADVQCTVNLVGGDP
YLNQPGPLGTQKPTAGPQTPQAQQKSLLQQLLTE
>54Balaenoptera acutorostrata scammoni|A0A384AV83|Mammalia|SRC1
MSGLGDSSSDPANPDSHKRKGSPCDTLASSTEKRRREQENKYLEELAELLSANISDIDSLSVKPDKCKILKKTV
DQIQLMKRMEQEKSTTDDEVQKSDISSSSQGVIEKESLGPLLLEALDGFFFVVNCEGRIVFVSENVTSYLGYNQ
EELMNTSVYSILHVGDHAEFVKNLLPKSLVNGVPWPQEATRRNSHTFNCRMLIHPPDEPGTENQEACQRYEVMQ
CFTVSQPKSIQEDGEDFQSCLICIARRLPRPPAITGVESFMTKQDTTGKIISIDTSSLRAAGRTGWEDLVRKCI
YAFFQPQGREPSYARQLFQEVMTRGTASSPSYRFILNDGTMLSAHTKCKLCYPQSPDMQPFIMGIHIIDREHSG
LSPQDDTNPGMSIPRVNPSVNPSISPAHGAARSATLPPSNNNVVSTRVNRQQSSDLHSSSHSNSSNSQGSFGCS
PGNQIVASVALNQGQASSQSTNPSLNLNNSPMEGTGISLAQFMSPRRQVSSGLATRPRMPNNSFPPNIPTLSSP
VSMTSTACNNSNRSYSNIPVTPLQSMNEGPNNSVGFSASSPVLRQMSSQNSPNRLNIQPAKAESKDNKEIASIL
NEMIQSDNSSNDGKPLDSGLLHNSDRLSDGDNKYSQTSHKLVQLLTTTAEQQLRHADIDTSCKEVLSCTGTPSS
ASASSSTGSCPSSHSSLTERHKILHRLLQEGSPSDITTLSVEPDKKDSASTSVAVTGQVQGNSSIKLELDASKK
KESKDHQLLRYLLDKDEKDLRSTPNLSLDDVKVKVEKKEQMDPCNTNPAPMTKPAPEEIKLESQSQFTADLDQF
DQLLPTLEKAAQLPGLCETERMDGAVTTVAIKSEILPAALQTTTARPTSRLNRLPELELEAIDNQFGQPGSGDQ
VSWTNNTVTAVNQNKPEDQCISSQLDELLCPPTTVEGRNDEKALLEQLVSFLSGKDETELAELDRALGIDKLVQ
GGGLDVLSERFPPQQATPPLMMEERPNLYSQPYSSPSPTANLSSPFQGMVRQKPSLGTMPVQVTPPRGAFSPGM
GMQTRQTLNRPPAAPNQLRLQLQQRLQGQQQLIHQNRQAILNQFAANAPVGINMRSGMQQQITPQPPLNAQMLA
QRQRELYSQQHRQRQLIQQQRAMLMRQQSFGNNLPPSSGLPVQMGNPRLPQGAPQQFPYPPSYGTNPGTPPAST
SPFSQLAANPEATLANRNSMVNRGMTGNMGGQFGTGISPQMQQNVFQYPGSGMVPQGEANFSPSLSPGSSMVPM
PIPPPQSSLLQQTPPTSGYQSPDMKAWQQGAMGNSNVFSQAVQNQPTPAQPGVYNNMSITVSMAGGNTNVQNMN
PMMGQMQMSSLQMPGMNTVCPEQINDPALRHTGLYCNQLSSTDLLKTEADGTQVQQVQVFADVQCTVNLVGGDP
YLNQPGPLGTQKPTAGPQTPQAQQKSLLQQLLTE
>55Balaenoptera acutorostrata scammoni|A0A384AV84|Mammalia|SRC1
MSGLGDSSSDPANPDSHKRKGSPCDTLASSTEKRRREQENKYLEELAELLSANISDIDSLSVKPDKCKILKKTV
DQIQLMKRMEQEKSTTDDEVQKSDISSSSQGVIEKESLGPLLLEALDGFFFVVNCEGRIVFVSENVTSYLGYNQ
EELMNTSVYSILHVGDHAEFVKNLLPKSLVNGVPWPQEATRRNSHTFNCRMLIHPPDEPGTENQEACQRYEVMQ
CFTVSQPKSIQEDGEDFQSCLICIARRLPRPPAITGVESFMTKQDTTGKIISIDTSSLRAAGRTGWEDLVRKCI
YAFFQPQGREPSYARQLFQEVMTRGTASSPSYRFILNDGTMLSAHTKCKLCYPQSPDMQPFIMGIHIIDREHSG
LSPQDDTNPGMSIPRVNPSVNPSISPAHGAARSATLPPSNNNVVSTRVNRQQSSDLHSSSHSNSSNSQGSFGCS
PGNQIVASVALNQGQASSQSTNPSLNLNNSPMEGTGISLAQFMSPRRQVSSGLATRPRMPNNSFPPNIPTLSSP
VSMTSTACNNSNRSYSNIPVTPLQSMNEGPNNSVGFSASSPVLRQMSSQNSPNRLNIQPAKAESKDNKEIASIL
NEMIQSDNSSNDGKPLDSGLLHNSDRLSDGDNKYSQTSHKLVQLLTTTAEQQLRHADIDTSCKEVLSCTGTPSS
ASASSSTGSCPSSHSSLTERHKILHRLLQEGSPSDITTLSVEPDKKDSASTSVAVTGQVQGNSSIKLELDASKK
KESKDHQLLRYLLDKDEKDLRSTPNLSLDDVKVKVEKKEQMDPCNTNPAPMTKPAPEEIKLESQSQFTADLDQF
DQLLPTLEKAAQLPGLCETERMDGAVTTVAIKSEILPAALQTTTARPTSRLNRLPELELEAIDNQFGQPGSGDQ
VSWTNNTVTAVNQNKPEDQCISSQLDELLCPPTTVEGRNDEKALLEQLVSFLSGKDETELAELDRALGIDKLVQ
GGGLDVLSERFPPQQATPPLMMEERPNLYSQPYSSPSPTANLSSPFQGMVRQKPSLGTMPVQVTPPRGAFSPGM
GMQTRQTLNRPPAAPNQLRLQLQQRLQGQQQLIHQNRQAILNQFAANAPVGINMRSGMQQQITPQPPLNAQMLA

QRQRELYSQQHRQRQLIQQQRAMLMRQQSFGNNLPPSSGLPVQMGNPRLPQGAPQQFPYPPSYGTNPGTPPAST
SPFSQLAANPEATLANRNSMVNRGMTGNMGGQFGTGISPQMQQNVFQYPGSGMVPQGEANFSPSLSPGSSMVPM
PIPPPQSSLLQQTPPTSGYQSPDMKAWQQGAMGNSNVFSQAVQNQPTPAQPGVYNNMSITVSMAGGNTNVQNMN
PMMGQMQMSSLQMPGMNTVCPEQINDPALRHTGLYCNQLSSTDLLKTEADGTQQVQQVQVFADVQCTVNLVGGD
PYLNQPGPLGTQKPTAGPQTPQAQQKSLLQQLLTE

>56Balaenoptera acutorostrata scammoni|A0A384AW22|Mammalia|SRC1
MSGLGDSSSDPANPDSHKRKGSPCDTLASSTEKRRREQENKYLEELAELLSANISDIDSLSVKPDKCKILKKTV
DQIQLMKRMEQEKSTTDDEVQKSDISSSSQGVIEKESLGPLLLEALDGFFFVVNCEGRIVFVSENVTSYLGYNQ
EELMNTSVYSILHVGDHAEFVKNLLPKSLVNGVPWPQEATRRNSHTFNCRMLIHPPDEPGTENQEACQRYEVMQ
CFTVSQPKSIQEDGEDFQSCLICIARRLPRPPAITGVESFMTKQDTTGKIISIDTSSLRAAGRTGWEDLVRKCI
YAFFQPQGREPSYARQLFQEVMTRGTASSPSYRFILNDGTMLSAHTKCKLCYPQSPDMQPFIMGIHIIDREHSG
LSPQDDTNPGMSIPRVNPSVNPSISPAHGAARSATLPPSNNNVVSTRVNRQQSSDLHSSSHSNSSNSQGSFGCS
PGNQIVASVALNQGQASSQSTNPSLNLNNSPMEGTGISLAQFMSPRRQVSSGLATRPRMPNNSFPPNIPTLSSP
VSMTSTACNNSNRSYSNIPVTPLQSMNEGPNNSVGFSASSPVLRQMSSQNSPNRLNIQPAKAESKDNKEIASIL
NEMIQSDNSSNDGKPLDSGLLHNSDRLSDGDNKYSQTSHKLVQLLTTTAEQQLRHADIDTSCKEVLSCTGTPSS
ASASSSTGSCPSSHSSLTERHKILHRLLQEGSPSDITTLSVEPDKKDSASTSVAVTGQVQGNSSIKLELDASKK
KESKDHQLLRYLLDKDEKDLRSTPNLSLDDVKVKVEKKEQMDPCNTNPAPMTKPAPEEIKLESQSQFTADLDQF
DQLLPTLEKAAQLPGLCETERMDGAVTTVAIKSEILPAALQTTTARPTSRLNRLPELELEAIDNQFGQPGSGDQ
VSWTNNTVTAVNQNKPEDQCISSQLDELLCPPTTVEGRNDEKALLEQLVSFLSGKDETELAELDRALGIDKLVQ
GGGLDVLSERFPPQQATPPLMMEERPNLYSQPYSSPSPTANLSSPFQGMVRQKPSLGTMPVQVTPPRGAFSPGM
GMQTRQTLNRPPAAPNQLRLQLQQRLQGQQQLIHQNRQAILNQFAANAPVGINMRSGMQQQITPQPPLNAQMLA
QRQRELYSQQHRQRQLIQQQRAMLMRQQSFGNNLPPSSGLPVQMGNPRLPQGAPQQFPYPPSYGTNPGTPPAST
SPFSQLAANPEATLANRNSMVNRGMTGNMGGQFGTGISPQMQQNVFQYPGSGMVPQGEANFSPSLSPGSSMVPM
PIPPPQSSLLQQTPPTSGYQSPDMKAWQQGAMGNSNVFSQAVQNQPTPAQPGVYNNMSITVSMAGGNTNVQNMN
PMMGQMQMSSLQMPGMNTVCPEQINDPALRHTGLYCNQLSSTDLLKTEADGTQDKKTEEFFSVVTTD
>57Gulo gulo|A0A3P4S765|Mammalia|SRC1
MSGLGDSSSDPANPDSHKRKGSPCDTLASSTEKRRREQENKYLEELAELLSANISDIDSL
SVKPDKCKILKKTVDQIQLMKRMEQEKSTTDDEVQKSDISSSSQGVIEKESLGPLLLEAL
DGFFFVVNCEGRIVFVSENVTSYLGYNQEELMNTSVYSILHVGDHAEFVKNLLPKSLVNG
VPWPQEATRRNSHTFNCRMLIHPPDEPGTENQEACQRYEVMQCFTVSQPKSIQEDGEDFQ
SCLICIARRLPRPPAITGVESFMTKQDTTGKIISIDTSSLRAAGRTGWEDLVRKCIYAFF
QPQGREPSYARQLFQEVMTRGTASSPSYRFILNDGTMLSAHTKCKLCYPQSPDMQPFIMG
IHIIDREHSGLSPQDDTNSGISIPRVNPSVNSSISPAHGVARSSTLPPSSNNMVSPRVNR
QQSSDLHSSGHSNSSNSQGSFGCSPGNQIVASVALNQGQASSQSTNPSLNLNNSPMEGTG
ISLAQFMSPRRQVSSGLAPRPRMPNNSFPPNIPTLSSPVGMTGTACNNSNRSYSNIPVTS
LQSMNEGPNNSVGFSASSPVLRQMSSQNSPSRLNIQPAKAESKDNKEIASILNEMIQSDN
SSSDGKPLDSGLLHNNDRLSDGDNKYSQTSHKLVQLLTTTAEQQLRHADIDTSCKEVLSC
SGSSSSASANSSSGSCPSSHSSLTERHKILHRLLQEGSPSDITTLSVEPDKKDSASTSVS
VTGQVQGSSSVKLELDASKKKESKDHQLLRYLLDKDEKDLRSTPNLSLDDVKVKVEKKEQ
MDPCNTNPTPMTKPAPEEIKLESQSQFTADLDQFDQLLPTLEKAAQLPGLCEPERMDGAV
TNVTIKSETLPTSLQSTTARPTSRLNRLPELELEAIDNQFGQPGTGDQIPWANNTVTPVN
QNKPEDQCISSQLDELLCPPTTVEGRNDEKALLEQLVSFLSGKDETELAELDRALGIDKL
VQGGGLDVLSERFPPQQATPPLMMEERPNLYSQPYSSPSPTANLSTPFQGMVRQKPSLGT
MPVQVTPPRGAFSPGMGMQPRQPLNRPPAAPNQLRLQLQQRLQGQQQLIHQNRQAILNQF
AANAPVGINMRSGMQQQITPQPPLNAQMLAQRQRELYSQQHRQRQLIQQQRAMLMRQQSF
GNNLPPSSGLPVQMGNPRLPQGAPQQFPYPPNYGTNPGTPPASTSPFSQLAANPESALAN
RNSMVNRGMTGNMGGQFGTGINPQMQQNVFQYPGSGMVPQGEANFAPSLSPGSSMVPMPI
PPPQSSLLQPTPPSSGYQSPDMKAWQQGAMGNNNVFSQAVQNQPTPAQPGVYNNMSITVS
MAGGNTNVQNMNPMMGQMQMSSLQMPGMNAVCPEQINDPALRHTGLYCNQLSSTDLLKTE
TDGTQDKKTEEFFSVVTTD
>58Equus caballus|A0A3Q2LK05|A0A3Q2LK05_HORSE Nuclear receptor coactivator
OS= OX=9796 GN=NCOA1 PE=3 SV=2

MPHIPFLNSSGRMRIKMPILKCTEKRRREQENKYLEELAELLSANISDIDSLSVKPDKCKILKKTVDQIQLMKR
MEQEKSTTDDEVQKSDISSSSQGVIEKESLGPLLLEALDGFFFVVNCEGRIVFVSENVTSYLGYNQEELMNTSV
YSILHVGDHAEFVKNLLPKSLVNGVPWPQEATRRNSHTFNCRMLIHPPDEPGTENQEACQRYEVMQCFTVSQPK
SIQEDGEDFQSCLICIARRLPRPPAVTGVESFMTKQDTTGKIISIDTSSLRAAGRTGWEDLVRKCIYAFFQPQG
REPSYARQLFQEVMTRGTASSPSYRFILNDGTMLSAHTKCKLCYPQSPDMQPFIMGIHIIDREHSGLSPQDDTS
SGMSIPRVNPSVTPSISPAHGVARTSTLPPSNSNMVSARVNRQQSSDLHSSSHSNSSNSQGNFGCSPGNPIVAS
VALNQGQANSQSTNPSLNLSNSSMEGTGISLAQFMSPRRQVSSGLATRPRMPNNSFPPNIPTLSSPVGMTSTTC
NSSNRSYSNIPVTSLQSMNEGPNNSVGFSATSPVLRQMSSQNSPSRLNIQPAKTESKDNKEIASILNEMIQSDN
SSSDGKPLDSGLLHNNDRLSDGDNKYSQTSHKLVQLLTTTAEQQLRHADIDTSCKEVLSCTGTSSSASANSSSG
SCPSSHSSLTERHKILHRLLQEGSPSDITTLSVEPDKKDSASTSVSVTGQVQGNSSIKVELDASKKKESKDHQL
LRYLLDKDEKDLRSTPNLSLDDVKVKVEKKEQMDPCNTNPTPMTKPAPEEIKLEAQSQFTADLDQFDQLLPTLE
KAAQLPGLCETERMDGAVTSVPIKSEILPASLQSTTARPTTSRLNRLPELELEAIDNQFGQAGTGDQIPWANNT
VTTANQNKPEDQCISSQLDELLCPPTTVEGRNDEKALLEQLVSFLSGKDETELAELDRALGIDKLVQGGGLDVL
SERFPPQQATPPLMMEERANIYSQPYSSPSPTANLSSPFQGMVRQKPSLGTMPVQVTPPRGAFSPGMGMQPRQT
LNRPPAAPNQLRLQLQQRLQGQQQLIHQNRQAILNQFAANTPVGINMRSGMQQQITPQPPLNAQMLAQRQRELY
SQQHRQRQLIQQQRAMLMRQQSFGNNLPPSSGLPVQMGNTRLPQGAPQQFPYPPNYGTNPGTPPASTSPFSQLA
ANPEATLANRNSMVNRGMTGNMGGQFGTGINPQMQQNVFQYPGSGMVPQGEANFAPSLSPGSSMVPMPIPPPQS
SLLQQTPPASGYQSPDMKAWQQGAMGNNNVFSQAVQNQPTPAQPGVYNNMSITVSMAGGNTNVQNMNPMMGQMQ
MSSLQMPGMNTVCPEQINDPALRHTGLYCNQLSSTDLLKTEADGTQDKKTEEFFSVVTTD
>59Suricata suricatta|A0A673UU48|Mammalia|SRC1
MSGLGDSSSDPANPDSHKRKGSPCDTLASSTEKRRREQENKYLEELAELLSANISDIDSLSVKPDKCKILKKTV
DQIQLMKRMEQEKSTTDDEVQKSDISSSSQGVIEKESLGPLLLEALDGFFFVVNCEGRIVFVSENVTSYLGYNQ
EELMNTSVYSILHVGDHAEFVKNLLPKSLVNGVPWPQEATRRNSHTFNCRMLIHPPDEPGTENQEACQRYEVMQ
CFTVSQPKSIQEDGEDFQSCLICIARRLPRPPAMTGVESFMTKQDTTGKIISIDTSSLRAAGRTGWEDLVRKCI
YAFFQPQGREPSYARQLFQEVMTRGTASSPSYRFILNDGTMLSAHTKCKLCYPQSPDMQPFIMGIHIIDREHSG
LSPQEDTNSGMSISRVNPSVNSSISPAHGVARSSTLPPSNNNMVSPRVNRQQSSDLHSSSHSNSSNSQGSFGCS
PGNQIVASVALNQGQASSQSTNPSLNLNNSPMEGTGISLAQFMSPRRQVSSGLATRPRMPNNSFPPNIPTLSSP
VGMTGTACNNSNRSYSNIPVTSLQSMNEGPNNSVGFSASSPVLRQMSSQNSPSRLNIQPAKAESKDNKEIASIL
NEMIQSDNSSSDGKPLDSGLLHNDRLSDGDNKYSQTSHKLVQLLTTTAEQQLRHAEIDTSCKEVLSCSGASGSA
SANSSSGSCPSSHSSLTERHKILHRLLQEGSPSDITTLSVEPDKKDSASTSVSVTGQVQGNSSVKLELDASKKK
ESKDHQLLRYLLDKDEKDLRSTPNLSLDDVKVKVEKKEQMDPCNTNPAPMAKPAPEEIKLESQSQFTADLDQFD
QLLPTLEKAAQLPGVCEPERMDGAVTNVTIKSEALPASLQSTTARPAPRLSRLPELELEAIDNQFGQPGTGDQI
PWGNNPVTTVNQNKPEDQCISSQLDELLCPPTTVEGRNDEKALLEQLVSFLSGKDETELAELDRALGIDKLVQG
GGLDVLSERFPPQQATPPLMMDERPNIYSQPYSSPSPTANLSSPFQGMVRQKPSLGTMPVQVTPPRGAFSPGMG
MQPRQTLSRPPAAPNQLRLQLQQRLQGQQQLIHQNRQAILNQFAANTPVGINMRSGMQQQITPQPPLNAQMLAQ
RQRELYSQQHRQRQLIQQQRAMLMRQQSFGNNLPPSSGLPVQMGNPRLPQGAPQQFPYPPNYGTNPGTPPASTS
PFSQLAANPESTLANRNSMVNRGMTGNMGGQFGTGINPQMQQNVFQYPGSGMVPQGEANFAPSLSPGSSMVPMP
IPPPQSSLLQQTPPTSGYQSPDMKAWQQGAMGNNNVFSQAVQSQPTPAQPGLYNNMSITVSMAGGNTNVQNMNP
MMGQMQMSSLQMPGMNTVCPEQINDPALRHTGLYCNQLSSTDLLKTETDGTQQVQQVQVFADVQCTVNLVGGDP
YLNQPGPLGTQKPTPGPQTPQAQQKSLLQQLLTE
>60Suricata suricatta|A0A673UWX3|Mammalia|SRC1
MSGLGDSSSDPANPDSHKRKGSPCDTLASSTEKRRREQENKYLEELAELLSANISDIDSLSVKPDKCKILKKTV
DQIQLMKRMEQEKSTTDDEVQKSDISSSSQGVIEKESLGPLLLEALDGFFFVVNCEGRIVFVSENVTSYLGYNQ
EELMNTSVYSILHVGDHAEFVKNLLPKSLVNGVPWPQEATRRNSHTFNCRMLIHPPDEPGTENQEACQRYEVMQ
CFTVSQPKSIQEDGEDFQSCLICIARRLPRPPAMTGVESFMTKQDTTGKIISIDTSSLRAAGRTGWEDLVRKCI
YAFFQPQGREPSYARQLFQEVMTRGTASSPSYRFILNDGTMLSAHTKCKLCYPQSPDMQPFIMGIHIIDREHSG
LSPQEDTNSGMSISRVNPSVNSSISPAHGVARSSTLPPSNNNMVSPRVNRQQSSDLHSSSHSNSSNSQGSFGCS
PGNQIVASVALNQGQASSQSTNPSLNLNNSPMEGTGISLAQFMSPRRQVSSGLATRPRMPNNSFPPNIPTLSSP
VGMTGTACNNSNRSYSNIPVTSLQSMNEGPNNSVGFSASSPVLRQMSSQNSPSRLNIQPAKAESKDNKEIASIL
NEMIQSDNSSSDGKPLDSGLLHNDRLSDGDNKYSQTSHKLVQLLTTTAEQQLRHAEIDTSCKEVLSCSGASGSA
SANSSSGSCPSSHSSLTERHKILHRLLQEGSPSDITTLSVEPDKKDSASTSVSVTGQVQGNSSVKLELDASKKK
ESKDHQLLRYLLDKDEKDLRSTPNLSLDDVKVKVEKKEQMDPCNTNPAPMAKPAPEEIKLESQSQFTADLDQFD
QLLPTLEKAAQLPGVCEPERMDGAVTNVTIKSEALPASLQSTTARPAPRLSRLPELELEAIDNQFGQPGTGDQI
PWGNNPVTTVNQNKPEDQCISSQLDELLCPPTTVEGRNDEKALLEQLVSFLSGKDETELAELDRALGIDKLVQG

GGLDVLSERFPPQQATPPLMMDERPNIYSQPYSSPSPTANLSSPFQGMVRQKPSLGTMPVQVTPPRGAFSPGMG
MQPRQTLSRPPAAPNQLRLQLQQRLQGQQQLIHQNRQAILNQFAANTPVGINMRSGMQQQITPQPPLNAQMLAQ
RQRELYSQQHRQRQLIQQQRAMLMRQQSFGNNLPPSSGLPVQMGNPRLPQGAPQQFPYPPNYGTNPGTPPASTS
PFSQLAANPESTLANRNSMVNRGMTGNMGGQFGTGINPQMQQNVFQYPGSGMVPQGEANFAPSLSPGSSMVPMP
IPPPQSSLLQQTPPTSGYQSPDMKAWQQGAMGNNNVFSQAVQSQPTPAQPGLYNNMSITVSMAGGNTNVQNMNP
MMGQMQMSSLQMPGMNTVCPEQQVQQVQVFADVQCTVNLVGGDPYLNQPGPLGTQKPTPGPQTPQAQQKSLLQQ
LLTE
>61Suricata suricatta|A0A673V5D4|Mammalia|SRC1
MSGLGDSSSDPANPDSHKRKGSPCDTLASSTEKRRREQENKYLEELAELLSANISDIDSLSVKPDKCKILKKTV
DQIQLMKRMEQEKSTTDDEVQKSDISSSSQGVIEKESLGPLLLEALDGFFFVVNCEGRIVFVSENVTSYLGYNQ
EELMNTSVYSILHVGDHAEFVKNLLPKSLVNGVPWPQEATRRNSHTFNCRMLIHPPDEPGTENQEACQRYEVMQ
CFTVSQPKSIQEDGEDFQSCLICIARRLPRPPAMTGVESFMTKQDTTGKIISIDTSSLRAAGRTGWEDLVRKCI
YAFFQPQGREPSYARQLFQEVMTRGTASSPSYRFILNDGTMLSAHTKCKLCYPQSPDMQPFIMGIHIIDREHSG
LSPQEDTNSGMSISRVNPSVNSSISPAHGVARSSTLPPSNNNMVSPRVNRQQSSDLHSSSHSNSSNSQGSFGCS
PGNQIVASVALNQGQASSQSTNPSLNLNNSPMEGTGISLAQFMSPRRQVSSGLATRPRMPNNSFPPNIPTLSSP
VGMTGTACNNSNRSYSNIPVTSLQSMNEGPNNSVGFSASSPVLRQMSSQNSPSRLNIQPAKAESKDNKEIASIL
NEMIQSDNSSSDGKPLDSGLLHNDRLSDGDNKYSQTSHKLVQLLTTTAEQQLRHAEIDTSCKEVLSCSGASGSA
SANSSSGSCPSSHSSLTERHKILHRLLQEGSPSDITTLSVEPDKKDSASTSVSVTGQVQGNSSVKLELDASKKK
ESKDHQLLRYLLDKDEKDLRSTPNLSLDDVKVKVEKKEQMDPCNTNPAPMAKPAPEEIKLESQSQFTADLDQFD
QLLPTLEKAAQLPGVCEPERMDGAVTNVTIKSEALPASLQSTTARPAPRLSRLPELELEAIDNQFGQPGTGDQI
PWGNNPVTTVNQNKPEDQCISSQLDELLCPPTTVEGRNDEKALLEQLVSFLSGKDETELAELDRALGIDKLVQG
GGLDVLSERFPPQQATPPLMMDERPNIYSQPYSSPSPTANLSSPFQGMVRQKPSLGTMPVQVTPPRGAFSPGMG
MQPRQTLSRPPAAPNQLRLQLQQRLQGQQQPPLNAQMLAQRQRELYSQQHRQRQLIQQQRAMLMRQQSFGNNLP
PSSGLPVQMGNPRLPQGAPQQFPYPPNYGTNPGTPPASTSPFSQLAANPESTLANRNSMVNRGMTGNMGGQFGT
GINPQMQQNVFQYPGSGMVPQGEANFAPSLSPGSSMVPMPIPPPQSSLLQQTPPTSGYQSPDMKAWQQGAMGNN
NVFSQAVQSQPTPAQPGLYNNMSITVSMAGGNTNVQNMNPMMGQMQMSSLQMPGMNTVCPEQINDPALRHTGLY
CNQLSSTDLLKTETDGTQQVQQVQVFADVQCTVNLVGGDPYLNQPGPLGTQKPTPGPQTPQAQQKSLLQQLLTE
>62Suricata suricatta|A0A673V6B0|Mammalia|SRC1
MSGLGDSSSDPANPDSHKRKGSPCDTLASSTEKRRREQENKYLEELAELLSANISDIDSLSVKPDKCKILKKTV
DQIQLMKRMEQEKSTTDDEVQKSDISSSSQGVIEKESLGPLLLEALDGFFFVVNCEGRIVFVSENVTSYLGYNQ
EELMNTSVYSILHVGDHAEFVKNLLPKSLVNGVPWPQEATRRNSHTFNCRMLIHPPDEPGTENQEACQRYEVMQ
CFTVSQPKSIQEDGEDFQSCLICIARRLPRPPAMTGVESFMTKQDTTGKIISIDTSSLRAAGRTGWEDLVRKCI
YAFFQPQGREPSYARQLFQEVMTRGTASSPSYRFILNDGTMLSAHTKCKLCYPQSPDMQPFIMGIHIIDREHSG
LSPQEDTNSGMSISRVNPSVNSSISPAHGVARSSTLPPSNNNMVSPRVNRQQSSDLHSSSHSNSSNSQGSFGCS
PGNQIVASVALNQGQASSQSTNPSLNLNNSPMEGTGISLAQFMSPRRQVSSGLATRPRMPNNSFPPNIPTLSSP
VGMTGTACNNSNRSYSNIPVTSLQSMNEGPNNSVGFSASSPVLRQMSSQNSPSRLNIQPAKAESKDNKEIASIL
NEMIQSDNSSSDGKPLDSGLLHNDRLSDGDNKYSQTSHKLVQLLTTTAEQQLRHAEIDTSCKEVLSCSGASGSA
SANSSSGSCPSSHSSLTERHKILHRLLQEGSPSDITTLSVEPDKKDSASTSVSVTGQVQGNSSVKLELDASKKK
ESKDHQLLRYLLDKDEKDLRSTPNLSLDDVKVKVEKKEQMDPCNTNPAPMAKPAPEEIKLESQSQFTADLDQFD
QLLPTLEKAAQLPGVCEPERMDGAVTNVTIKSEALPASLQSTTARPAPRLSRLPELELEAIDNQFGQPGTGDQI
PWGNNPVTTVNQNKPEDQCISSQLDELLCPPTTVEGRNDEKALLEQLVSFLSGKDETELAELDRALGIDKLVQG
GGLDVLSERFPPQQATPPLMMDERPNIYSQPYSSPSPTANLSSPFQGMVRQKPSLGTMPVQVTPPRGAFSPGMG
MQPRQTLSRPPAAPNQLRLQLQQRLQGQQQLIHQNRQAILNQFAANTPVGINMRSGMQQQITPQPPLNAQMLAQ
RQRELYSQQHRQRQLIQQQRAMLMRQQSFGNNLPPSSGLPVQMGNPRLPQGAPQQFPYPPNYGTNPGTPPASTS
PFSQLAANPESTLANRNSMVNRGMTGNMGGQFGTGINPQMQQNVFQYPGSGMVPQGEANFAPSLSPGSSMVPMP
IPPPQSSLLQQTPPTSGYQSPDMKAWQQGAMGNNNVFSQAVQSQPTPAQPGLYNNMSITVSMAGGNTNVQNMNP
MMGQMQMSSLQMPGMNTVCPEQINDPALRHTGLYCNQLSSTDLLKTETDGTQDKKTEEFFSVVTTD
>63Pan troglodytes|A0A6D2Y8C8|Mammalia|SRC1
MSGLGDSSSDPANPDSHKRKGSPCDTLASSTEKRRREQENKYLEELAELLSANISDIDSLSVKPDKCKILKKTV
DQIQLMKRMEQEKSTTDDDVQKSDISSSSQGVIEKESLGPLLLEALDGFFFVVNCEGRIVFVSENVTSYLGYNQ
EELMNTSVYSILHVGDHAEFVKNLLPKSLVNGVPWPQEATRRNSHTFNCRMLIHPPDEPGTENQEACQRYEVMQ
CFTVSQPKSIQEDGEDFQSCLICIARRLPRPPAITGVESFMTKQDTTGKIISIDTSSLRAAGRTGWEDLVRKCI
YAFFQPQGREPSYARQLFQEVMTRGTASSPSYRFILNDGTMLSAHTKCKLCYPQSPDMQPFIMGIHIIDREHSG
LSPQDDTNSGMSIPRVNPSVNPSISPAHGVARSSTLPPSNSNMVSTRINRQQSSDLHSSSHSNSSNSQGSFGCS

PGSQIVANVALNQGQASSQSSNPSLNLNNSPMEGTGISLAQFMSPRRQVTSGLATRPRMPNNSFPPNISTLSSP
VGMTSSACNNNNRSYSNIPVTSLQGMNEGPNNSVGFSASSPVLRQMSSQNSPSRLNIQPAKAESKDNKEIASIL
NEMIQSDNSSSDGKPLDSGLLHNNDRLSDGDSKYSQTSHKLVQLLTTTAEQQLRHADIDTSCKDVLSCTGTSNS
ASANSSGGSCPSSHSSLTERHKILHRLLQEGSPSDITTLSVEPDKKDSASTSVSVTGQVQGNSSIKLELDASKK
KESKDHQLLRYLLDKDEKDLRSTPNLSLDDVKVKVEKKEQMDPCNTNPTPMTKPTPEEIKLEAQSQFTADLDQF
DQLLPTLEKAAQLPGLCETDRMDGAVTSVTIKSEILPASLQSATARPTSRLNRLPELELEAIDNQFGQPGTGDQ
IPWTNNTVTAINQSKSEDQCISSQLDELLCPPTTVEGRNDEKALLEQLVSFLSGKDETELAELDRALGIDKLVQ
GGGLDVLSERFPPQQATPPLIMEERPNLYSQPYSSPSPTANLPSPFQGMVRQKPSLGTMPVQVTPPRGAFSPGM
GMQPRQTLNRPPAAPNQLRLQLQQRLQGQQQLIHQNRQAILNQFAATAPVGINMRSGMQQQITPQPPLNAQMLA
QRQRELYSQQHRQRQLIQQQRAMLMRQQSFGNNLPPSSGLPVQMGNPRLPQGAPQQFPYPPNYGTNPGTPPAST
SPFSQLAANPEASLANRNSMVSRGMTGNMGGQFGTGINPQMQQNVFQYPGAGMVPQGEANFAPSLSPGSSMVPM
PIPPPQSSLLQQTPPASGYQSPDMKAWQQGAIGNNNVFSQAVQNQPTPAQPGVYNNMSITVSMAGGNTNVQNMN
PMMAQMQMSSLQMPGMNTVCPEQINDPALRHTGLYCNQLSSTDLLKTEADGTQDKKTEEFFSVVTTD
>64Sapajus apella|A0A6J3J5R5|Mammalia|SRC1
MSGLGDSSSDPANPDSHKRKGSPCDTLASSTEKRRREQENKYLEELAELLSANISDIDSLSVKPDKCKILKKTV
DQIQLMKRMEQEKSTTDDDVQKSDISSSSQGVIEKESLGPLLLEALDGFFFVVNCEGRIVFVSENVTSYLGYNQ
EELMNTSVYSILHVGDHAEFVKNLLPKSLVNGVPWPQEATRRNSHTFNCRMLIHPPDEPGTENQEACQRYEVMQ
CFTVSQPKSIQEDGEDFQSCLICIARRLPRPPAITGVESFMTKQDTTGKIISIDTSSLRAAGRTGWEDLVRKCI
YAFFQPQGREPSYARQLFQEVMTRGTASSPSYRFILNDGTMLSAHTKCKLCYPQSPDMQPFIMGIHIIDREHSG
LSPQDDTNSGMSIPRVNPSVNPNISPAHGVARSSTLPPSNSNMVSTRINRQQSSDLHSSSHSNSSNSQGSFGCS
PGSQIVANVALNQGQASSQSSNPSLNLNNSPMEGTGISLAQFMSPRRQVTSGLATRPRMPSNSFPPNISTLSSP
VGMTSSACNNNNRSYSNIPVTSLQGMNEGPNNSVGFSASSPVLRQMSSQNSPSRLNIQPAKAESKDNKEIASIL
NEMIQSDNSSSDGKPLDSGLLHNSDRLSDGDSKYSQTSHKLVQLLTTTAEQQLRHADIDTSCKDVLSCTGTSNS
ASANTSGGSCPSSHSSLTERHKILHRLLQEGSPSDITTLSVEPDKKDSASTSVSVTGQVQGNSSIKLELDASKK
KESKDHQLLRYLLDKDEKDLRSTPNLSLDDVKVKVEKKEQMDPCNTNPTPMTKPAPEEIKLEAQSQFTADLDQF
DQLLPTLEKAAQLPGLCETDRMDGAVTSVTIKSEILPASLQSATARPTSRLNRLPELELEAIDNQFGQPGTGDQ
IPWTNNTVTAINQNKSEDQCISSQLDELLCPPTTVEGRNDEKALLEQLVSFLSGKDETELAELDRALGIDKLVQ
GGGLDVLSERFPPQQATPPLMMEERPSLYSQSYSSPSPTANLPSPFQGMVRQKPSLGTMPVQVTPPRGAFSPGM
GMQPRQTLNRPPAAPNQLRLQLQQRLQGQQQLIHQNRQAILNQFAATAPVGINMRSGMQQQITPQPPLNAQMLA
QRQRELYSQQHRQRQLIQQQRAMLMRQQSFGNNLPPSSGLPVQMGNPRLPQGAPQQFPYPPNYGTNPGTPPAST
SPFSQLAANPEASLANRSSMVSRGMTGNMGGQFGTGINPQMQQNVFQYPGAGMVPQGEANFAPSLSPGSSMVPM
PIPPPQSSLLQQTPPASGYQSPDMKAWQQGAIGNNNVFSQAVQNQPTPAQPGVYNNMSITVSMAGGNTNVQNMN
PMMGQMQMSSLQMPGMNTVCPEQINDPALRHTGLYCNQLSSTDLLKTEADGTQVQQVQVFADVQCTVNLVGGDP
YLNQPGPLGTQKPTSGPQTPQAQQKSLLQQLLTE

>65Sapajus apella|A0A6J3J6N0|Mammalia|SRC1
MSGLGDSSSDPANPDSHKRKGSPCDTLASSTEKRRREQENKYLEELAELLSANISDIDSLSVKPDKCKILKKTV
DQIQLMKRMEQEKSTTDDDVQKSDISSSSQGVIEKESLGPLLLEALDGFFFVVNCEGRIVFVSENVTSYLGYNQ
EELMNTSVYSILHVGDHAEFVKNLLPKSLVNGVPWPQEATRRNSHTFNCRMLIHPPDEPGTENQEACQRYEVMQ
CFTVSQPKSIQEDGEDFQSCLICIARRLPRPPAITGVESFMTKQDTTGKIISIDTSSLRAAGRTGWEDLVRKCI
YAFFQPQGREPSYARQLFQEVMTRGTASSPSYRFILNDGTMLSAHTKCKLCYPQSPDMQPFIMGIHIIDREHSG
LSPQDDTNSGMSIPRVNPSVNPNISPAHGVARSSTLPPSNSNMVSTRINRQQSSDLHSSSHSNSSNSQGSFGCS
PGSQIVANVALNQGQASSQSSNPSLNLNNSPMEGTGISLAQFMSPRRQVTSGLATRPRMPSNSFPPNISTLSSP
VGMTSSACNNNNRSYSNIPVTSLQGMNEGPNNSVGFSASSPVLRQMSSQNSPSRLNIQPAKAESKDNKEIASIL
NEMIQSDNSSSDGKPLDSGLLHNSDRLSDGDSKYSQTSHKLVQLLTTTAEQQLRHADIDTSCKDVLSCTGTSNS
ASANTSGGSCPSSHSSLTERHKILHRLLQEGSPSDITTLSVEPDKKDSASTSVSVTGQVQGNSSIKLELDASKK
KESKDHQLLRYLLDKDEKDLRSTPNLSLDDVKVKVEKKEQMDPCNTNPTPMTKPAPEEIKLEAQSQFTADLDQF
DQLLPTLEKAAQLPGLCETDRMDGAVTSVTIKSEILPASLQSATARPTSRLNRLPELELEAIDNQFGQPGTGDQ
IPWTNNTVTAINQNKSEDQCISSQLDELLCPPTTVEGRNDEKALLEQLVSFLSGKDETELAELDRALGIDKLVQ
GGGLDVLSERFPPQQATPPLMMEERPSLYSQSYSSPSPTANLPSPFQGMVRQKPSLGTMPVQVTPPRGAFSPGM
GMQPRQTLNRPPAAPNQLRLQLQQRLQGQQQLIHQNRQAILNQFAATAPVGINMRSGMQQQITPQPPLNAQMLA
QRQRELYSQQHRQRQLIQQQRAMLMRQQSFGNNLPPSSGLPVQMGNPRLPQGAPQQFPYPPNYGTNPGTPPAST
SPFSQLAANPEASLANRSSMVSRGMTGNMGGQFGTGINPQMQQNVFQYPGAGMVPQGEANFAPSLSPGSSMVPM
PIPPPQSSLLQQTPPASGYQSPDMKAWQQGAIGNNNVFSQAVQNQPTPAQPGVYNNMSITVSMAGGNTNVQNMN

PMMGQMQMSSLQMPGMNTVCPEQQVQQVQVFADVQCTVNLVGGDPYLNQPGPLGTQKPTSGPQTPQAQQKSLLQ
QLLTE
>66Sapajus apella|A0A6J3J719|Mammalia|SRC1
MSGLGDSSSDPANPDSHKRKGSPCDTLASSTEKRRREQENKYLEELAELLSANISDIDSLSVKPDKCKILKKTV
DQIQLMKRMEQEKSTTDDDVQKSDISSSSQGVIEKESLGPLLLEALDGFFFVVNCEGRIVFVSENVTSYLGYNQ
EELMNTSVYSILHVGDHAEFVKNLLPKSLVNGVPWPQEATRRNSHTFNCRMLIHPPDEPGTENQEACQRYEVMQ
CFTVSQPKSIQEDGEDFQSCLICIARRLPRPPAITGVESFMTKQDTTGKIISIDTSSLRAAGRTGWEDLVRKCI
YAFFQPQGREPSYARQLFQEVMTRGTASSPSYRFILNDGTMLSAHTKCKLCYPQSPDMQPFIMGIHIIDREHSG
LSPQDDTNSGMSIPRVNPSVNPNISPAHGVARSSTLPPSNSNMVSTRINRQQSSDLHSSSHSNSSNSQGSFGCS
PGSQIVANVALNQGQASSQSSNPSLNLNNSPMEGTGISLAQFMSPRRQVTSGLATRPRMPSNSFPPNISTLSSP
VGMTSSACNNNNRSYSNIPVTSLQGMNEGPNNSVGFSASSPVLRQMSSQNSPSRLNIQPAKAESKDNKEIASIL
NEMIQSDNSSSDGKPLDSGLLHNSDRLSDGDSKYSQTSHKLVQLLTTTAEQQLRHADIDTSCKDVLSCTGTSNS
ASANTSGGSCPSSHSSLTERHKILHRLLQEGSPSDITTLSVEPDKKDSASTSVSVTGQVQGNSSIKLELDASKK
KESKDHQLLRYLLDKDEKDLRSTPNLSLDDVKVKVEKKEQMDPCNTNPTPMTKPAPEEIKLEAQSQFTADLDQF
DQLLPTLEKAAQLPGLCETDRMDGAVTSVTIKSEILPASLQSATARPTSRLNRLPELELEAIDNQFGQPGTGDQ
IPWTNNTVTAINQNKSEDQCISSQLDELLCPPTTVEGRNDEKALLEQLVSFLSGKDETELAELDRALGIDKLVQ
GGGLDVLSERFPPQQATPPLMMEERPSLYSQSYSSPSPTANLPSPFQGMVRQKPSLGTMPVQVTPPRGAFSPGM
GMQPRQTLNRPPAAPNQLRLQLQQRLQGQQQLIHQNRQAILNQFAATAPVGINMRSGMQQQITPQPPLNAQMLA
QRQRELYSQQHRQRQLIQQQRAMLMRQQSFGNNLPPSSGLPVQMGNPRLPQGAPQQFPYPPNYGTNPGTPPAST
SPFSQLAANPEASLANRSSMVSRGMTGNMGGQFGTGINPQMQQNVFQYPGAGMVPQGEANFAPSLSPGSSMVPM
PIPPPQSSLLQQTPPASGYQSPDMKAWQQGAIGNNNVFSQAVQNQPTPAQPGVYNNMSITVSMAGGNTNVQNMN
PMMGQMQMSSLQMPGMNTVCPEQINDPALRHTGLYCNQLSSTDLLKTEADGTQQVQQVQVFADVQCTVNLVGGD
PYLNQPGPLGTQKPTSGPQTPQAQQKSLLQQLLTE
>67Sapajus apella|A0A6J3J7U6|Mammalia|SRC1
MSGLGDSSSDPANPDSHKRKGSPCDTLASSTEKRRREQENKYLEELAELLSANISDIDSLSVKPDKCKILKKTV
DQIQLMKRMEQEKSTTDDDVQKSDISSSSQGVIEKESLGPLLLEALDGFFFVVNCEGRIVFVSENVTSYLGYNQ
EELMNTSVYSILHVGDHAEFVKNLLPKSLVNGVPWPQEATRRNSHTFNCRMLIHPPDEPGTENQEACQRYEVMQ
CFTVSQPKSIQEDGEDFQSCLICIARRLPRPPAITGVESFMTKQDTTGKIISIDTSSLRAAGRTGWEDLVRKCI
YAFFQPQGREPSYARQLFQEVMTRGTASSPSYRFILNDGTMLSAHTKCKLCYPQSPDMQPFIMGIHIIDREHSG
LSPQDDTNSGMSIPRVNPSVNPNISPAHGVARSSTLPPSNSNMVSTRINRQQSSDLHSSSHSNSSNSQGSFGCS
PGSQIVANVALNQGQASSQSSNPSLNLNNSPMEGTGISLAQFMSPRRQVTSGLATRPRMPSNSFPPNISTLSSP
VGMTSSACNNNNRSYSNIPVTSLQGMNEGPNNSVGFSASSPVLRQMSSQNSPSRLNIQPAKAESKDNKEIASIL
NEMIQSDNSSSDGKPLDSGLLHNSDRLSDGDSKYSQTSHKLVQLLTTTAEQQLRHADIDTSCKDVLSCTGTSNS
ASANTSGGSCPSSHSSLTERHKILHRLLQEGSPSDITTLSVEPDKKDSASTSVSVTGQVQGNSSIKLELDASKK
KESKDHQLLRYLLDKDEKDLRSTPNLSLDDVKVKVEKKEQMDPCNTNPTPMTKPAPEEIKLEAQSQFTADLDQF
DQLLPTLEKAAQLPGLCETDRMDGAVTSVTIKSEILPASLQSATARPTSRLNRLPELELEAIDNQFGQPGTGDQ
IPWTNNTVTAINQNKSEDQCISSQLDELLCPPTTVEGRNDEKALLEQLVSFLSGKDETELAELDRALGIDKLVQ
GGGLDVLSERFPPQQATPPLMMEERPSLYSQSYSSPSPTANLPSPFQGMVRQKPSLGTMPVQVTPPRGAFSPGM
GMQPRQTLNRPPAAPNQLRLQLQQRLQGQQQLIHQNRQAILNQFAATAPVGINMRSGMQQQITPQPPLNAQMLA
QRQRELYSQQHRQRQLIQQQRAMLMRQQSFGNNLPPSSGLPVQMGNPRLPQGAPQQFPYPPNYGTNPGTPPAST
SPFSQLAANPEASLANRSSMVSRGMTGNMGGQFGTGINPQMQQNVFQYPGAGMVPQGEANFAPSLSPGSSMVPM
PIPPPQSSLLQQTPPASGYQSPDMKAWQQGAIGNNNVFSQAVQNQPTPAQPGVYNNMSITVSMAGGNTNVQNMN
PMMGQMQMSSLQMPGMNTVCPEQINDPALRHTGLYCNQLSSTDLLKTEADGTQDKKTEEFFSVVTTD

>68Octodon degus|A0A6P3ELZ7|Mammalia|SRC1
MSGLGDSSSDPANPDSHKRKGSPCDTLASSTEKRRREQENKYLEELAELLSANISDIDSLSVKPDKCKILKKTV
DQIQLMKRMEQEKSTTDDDVQKSDISSSSQGVIEKESLGPLLLEALDGFFFVVNCEGRIVFVSENVTSYLGYNQ
EELMNTSVYSILHVGDHAEFVKNLLPKSLVNGVPWPQEAARRNSHTFNCRMLIHPPDEPGTENQEACQRYEVMQ
CFTVSQPKSIQEDGEDFQSCLICIARRLPRPPAITGVESFMTKQDTTGKIISIDTSSLRAAGRTGWEDLVRKCI
YAFFQPQGREPSYARQLFQEVMTRGTASSPSYRFILNDGTMLSAHTKCKLCYPQSPDMQPFIMGIHIIDREHSG
LSPQDDTNSGMTVPRVNPSVNPSISPAHGVARPSTLPPSNSNMVSTRVNRQQSSDLHSSSSHTNSSNNQGSFGC
SPGNQIVANVALNQGQASSQSSNPSLSLNNSAIEGTGISLTQFMSPRRQVASGLAARPRMPNNSFPPNIPTLNS
PVGMTSGASNNSNRPYSNIPVTSLQGMNEGPSNSVGFSANSPVLRQMSSQNSPSRLNLQPAKAESKDNKEIASI
LNEMIQSDNSSSDGKPLDSGLLHNNDRLSDGDSKYAQTSHKLVQLLTTTAEQQLRHADIDASCKDVLSCTGTSN

SASANSSGGTCPSSHSSLTERHKILHRLLQEGSPSDITTLSVEPDKKDNASTSVSVTGQAQGNSSIKLELDASK
KKESKDHQLLRYLLDKDEKDLRSTPNLSLDDVKVKVEKKEQMDPCNTNPTPVTKPAPEEIKLESQSQFTADLDQ
FDQLLPTLEKAAQLPGLCETERMEGAATSATIKSEILPASLQSTTARPTSRLNRLPELELETIDNQFGQPGTGD
QIPWANSAVTAVNQTKQEDQCISSQLDELLCPPTTVEGRNDEKALLEQLVSFLSGKDETELAELDRALGIDKLV
QGGGLDVLSERFPPQQATPPLMMEERPNLYPQPYSSPSPTANLPSPFQGMVRQKPSLGTMPVQVTPPRGTFSPG
IGMQPRQTLNRPPAAPNQLRLQLQQRLQGQQQLIHQNRQAILNQFAANAPVGINMRSGMQQQITPQPPLNAQML
AQRQRELYSQQHRQRQLIQQQRAMLMRQQSFGNNLPPSSGLPVQMGNPRLPQGAPQQFPYPPNYGTNPGTPPAS
TSPFSQLAANPEASLATRNSMVSRGIAGNMGGQFGTAINPQMQQNVFQYPGSGMVPQSEANFAPSLSPGSSMVP
MPIPPPQSSLLQQTPPTSGYQSPDMKAWQQGAMGNNNVFSQAVQNQPTPAPPGVYNNMSITVSMAGGNTNVQNM
NPMMGQMQMSSLQMPGMNTVCPEQINDPALRHTGLYCNQLSSTDLLKTEADGTQVQQVQVFADVQCTVNLVGGD
PYLNQPGPLGSQKPTPGPQTPQAQQKSLLQQLLTE
>69Octodon degus|A0A6P3F1F3|Mammalia|SRC1
MSGLGDSSSDPANPDSHKRKGSPCDTLASSTEKRRREQENKYLEELAELLSANISDIDSLSVKPDKCKILKKTV
DQIQLMKRMEQEKSTTDDDVQKSDISSSSQGVIEKESLGPLLLEALDGFFFVVNCEGRIVFVSENVTSYLGYNQ
EELMNTSVYSILHVGDHAEFVKNLLPKSLVNGVPWPQEAARRNSHTFNCRMLIHPPDEPGTENQEACQRYEVMQ
CFTVSQPKSIQEDGEDFQSCLICIARRLPRPPAITGVESFMTKQDTTGKIISIDTSSLRAAGRTGWEDLVRKCI
YAFFQPQGREPSYARQLFQEVMTRGTASSPSYRFILNDGTMLSAHTKCKLCYPQSPDMQPFIMGIHIIDREHSG
LSPQDDTNSGMTVPRVNPSVNPSISPAHGVARPSTLPPSNSNMVSTRVNRQQSSDLHSSSSHTNSSNNQGSFGC
SPGNQIVANVALNQGQASSQSSNPSLSLNNSAIEGTGISLTQFMSPRRQVASGLAARPRMPNNSFPPNIPTLNS
PVGMTSGASNNSNRPYSNIPVTSLQGMNEGPSNSVGFSANSPVLRQMSSQNSPSRLNLQPAKAESKDNKEIASI
LNEMIQSDNSSSDGKPLDSGLLHNNDRLSDGDSKYAQTSHKLVQLLTTTAEQQLRHADIDASCKDVLSCTGTSN
SASANSSGGTCPSSHSSLTERHKILHRLLQEGSPSDITTLSVEPDKKDNASTSVSVTGQAQGNSSIKLELDASK
KKESKDHQLLRYLLDKDEKDLRSTPNLSLDDVKVKVEKKEQMDPCNTNPTPVTKPAPEEIKLESQSQFTADLDQ
FDQLLPTLEKAAQLPGLCETERMEGAATSATIKSEILPASLQSTTARPTSRLNRLPELELETIDNQFGQPGTGD
QIPWANSAVTAVNQTKQEDQCISSQLDELLCPPTTVEGRNDEKALLEQLVSFLSGKDETELAELDRALGIDKLV
QGGGLDVLSERFPPQQATPPLMMEERPNLYPQPYSSPSPTANLPSPFQGMVRQKPSLGTMPVQVTPPRGTFSPG
IGMQPRQTLNRPPAAPNQLRLQLQQRLQGQQQLIHQNRQAILNQFAANAPVGINMRSGMQQQITPQPPLNAQML
AQRQRELYSQQHRQRQLIQQQRAMLMRQQSFGNNLPPSSGLPVQMGNPRLPQGAPQQFPYPPNYGTNPGTPPAS
TSPFSQLAANPEASLATRNSMVSRGIAGNMGGQFGTAINPQMQQNVFQYPGSGMVPQSEANFAPSLSPGSSMVP
MPIPPPQSSLLQQTPPTSGYQSPDMKAWQQGAMGNNNVFSQAVQNQPTPAPPGVYNNMSITVSMAGGNTNVQNM
NPMMGQMQMSSLQMPGMNTVCPEQINDPALRHTGLYCNQLSSTDLLKTEADGTQQVQQVQVFADVQCTVNLVGG
DPYLNQPGPLGSQKPTPGPQTPQAQQKSLLQQLLTE
>70Bison bison bison|A0A6P3H6F2|Mammalia|SRC1
MSGLGDSSSDPANPDSHKRKGSPCDTLASSTEKRRREQENKYLEELAELLSANISDIDSL
SVKPDKCKILKKTVDQIQLMKRMEQGRKINRKTLWLCSLLLRGLIQQTGDTGILGLQRGE
PPRDCMAPSLLGMDTRTDWLILPQPKMGGFRRWGNGVVQREGHVDTDTGIGPQRKKSAPC
TVHTLHTAASQDFSTLLFNVNKNPVNPFFFFSLQALDGFFFVVNCEGRIVFVSENVTSYL
GYNQEELMNTSVYSILHVGDHAEFVKNLLPKSLVNGVPWPQEATRRNSHTFNCRMLIHRP
DEPGPENQEACQRYEVMQCFTVSQPKSIQEDGEDFQSCLICIARRLPRPPAITGVESFMT
KQDTTGKIISIDTSSLRAAGRTGWEDLVRKCIYAFFQPQGREPSYARQLFQEVMTRGTAS
SPSYRFILNDGTMLSAHTKCKLCYPQSPDMQPFIMGIHIIDREHSGLSPQDDTNPGMSVP
RVNPSVNPSISPAHSVARSSTLPPSNSNMVPTRVNRQQSSDLHSSSHSNSSNSQGSFGCS
PGNQIVASVALNQGQASSQSTNPPLNLNNSPMEGTGISLAQFMSPRRQVSSGLATRPRMP
NNSFPPNIPTLSSPVSMTSTACNNSNRSYSNIPVTPLQSMNEGPNNSVGFSASSPVLRQM
SSQNSPSRLNIQPAKAESKDNKEIASILNEMIQSDNSSNDSKPLDSGLLHNNDRLSDGDN
KYSQTSHKLVQLLTTTAEQQLRHADIDTSCKEVLSCTGTSTSASTNSSSGSCPSSHSSLT
ERHKILHRLLQEGSPSDITTLSVEPDKKDSASTSXSVTGQVQGNSSVKLELDASKKKESK
DHQLLRYLLDKDEKDLRSTPNLSLDDVKVKVEKKEQMDPCNTNPAPMTKPAPEEIKLESQ
SQFTADLDQFDQLLPTLEKAPQLPGLCETERMDGAVSNVAIKSEILPSALQSTSARPTSR
LNRLPELELEAIDNQFGQPGTGDQIPWANNTVTAVNQNKPEDQCISSQLDELLCPPTTVE
GRNDEKALLEQLVSFLSGKDETELAELDRALGIDKLVQGGGLDVLSERFPPQQATPPLMM
EERPNLYSQPYSSPSPTANLSSPFQGMVRQKPSLGTMPVQVTPPRGAFSPGMGMQPRQTL
NRPPAAPNQLRLQLQQRLQGQQQLIHQNRQAILNQFAANAPVGINMRSGMQQQITPQPPL
NAQMLAQRQRELYSQQHRQRQLIQQQRAMLMRQQSFGNSLPPSSGLPVQMGNPRLPQGAP

QQFPYPPNYGTNPGTPPASTSPFSQLAANPEAPLANRSSMVNRGMTGNMGGQFGTGINPQ
MQQNVFQYPGSGMVPQGEANFAPSLSPGSSMVPMPIPPPQSSLLQQTPPTSGYQSPDMKA
WQQGAMGNNNVFSQAIQNQPTPAQPGVYNNMSITVSMAGGNTNVQNMNPMMGQMQMSSLQ
MPGMSTVCPEQINDPALRHTGLYCNQLSSTDLLKTEADGTQVQQVQVFADVQCTVNLVGG
DPYLNQPGPLGTQKPTAGPQTPQAQQKSLLQQLLTE
>71Pteropus vampyrus|A0A6P3PYQ4|Mammalia|SRC1
MSGLGDSSSDPANPDSHKRKGSPCDTLASSTEKRRREQENKYLEELAELLSANISDIDSLSVKPDKCKILKKTV
DQIQLMKRMEQEKSATDDEVQKSDISSSSQGVIEKESLGPLLLEALDGFFFVVNCEGRIVFVSENVTSYLGYNQ
EELMNTSVYSILHVGDHAEFVKNLLPKSLVNGVPWPQEATRRNSHTFNCRMLIHPPDEPGIENQEACQRYEVMQ
CFTVSQPKSIQEDGEDFQSCLICIARRLPRPPAITGVESFMTKQDTTGKIISIDTSSLRAAGRTGWEDLVRKCI
YAFFQPQGREPSYARQLFQEVMTRGTASSPSYRFILNDGTMLSAHTKCKLCYPQNADMQPFIMGIHIIDREHSG
LSPQDDTNSGMSVPRVNPSVNPSISPAHGVARSSTLPPSNSNIVSTRVNRQQSSDLHSSSHSNSSNSQGSFGCS
PGNQIVASVALNQGQASSQSTNPSLNLNNSPIEGTGISLAQFMSPRRQVSSGLATRPRMPSNSFPPNIPTLSSP
VGMTSTACNNSNRSYSNIPVTPLQSMNEGPNNSVGFSASSPVLRQISSQNSPSRLNIQPAKAESKDNKEIASIL
NEMIQSDNSSSDGKPLDSGLLHNNDRLSDGDNKYSQTSHKLVQLLTTTAEQQLRHADIDTSCKEVLSCAGTSSS
ASANSSGGSCPSSHSSLTERHKILHRLLQEGSPSDITTLSVEPDKKDSASASVSVTGQAQGNSSVKLELDASKK
KESKDHQLLRYLLDKDEKDLRSTPNLSLDDVKVKVEKKEQMDPCNTNPTPMTKPAPEEIKLESQNQFTADLDQF
DQLLPTLEKAAQLPGLCETERMDGAVTSVTVKSEILPPSLQSTTARPTSRLNRLPELELEAIDNQFGQPGTGDQ
IPWANNAVTAVNQNKPEDQCISSQLDELLCPPTTVEGRNDEKALLEQLVSFLSGKDETELAELDRALGIDKLVQ
GGGLDVLSERFPPQQATPPLMMEERPNLYSQPYSSPSPTANLTSPFQGMVRQKPSLGTMPVQVTPPRGAFSPGM
GMQPRQTLNRPPAAPNQLRLQLQQRLQGQQQLIHQNRQAILNQFAANAPVGINMRSGMQQQITPQPPLNAQMLA
QRQRELYSQQHRQRQLIQQQRAMLMRQQSFGNNLPPSSGLPVQMGNPRLPQGASQQFPYPPNYGTNPGTPPAST
SPFSQLAANPEAALASRNSMVNRGMTGNLGGQFGTGINPQMQQTVFQYPGSGMVPQGEANFAPSLSPGNSMVPM
PIPPPQTSLLQQTPPASGYQSPDMKAWQQGAMGNNNVFGQAVQNQPAPAQPGVYNNMSITVSMAGGNTNVQNMN
PMMGQMQMSSLQMPGMNTVCPEQINDSALRHTGLYCNQLSSTDLLKTEADGTQQVQQVQVFADVQCTVNLVGGD
PYLNQPGPLGTQKPTAGPQTPQAQQKSLLQQLLTE

>72Pteropus vampyrus|A0A6P3Q968|Mammalia|SRC1
MSGLGDSSSDPANPDSHKRKGSPCDTLASSTEKRRREQENKYLEELAELLSANISDIDSLSVKPDKCKILKKTV
DQIQLMKRMEQEKSATDDEVQKSDISSSSQGVIEKESLGPLLLEALDGFFFVVNCEGRIVFVSENVTSYLGYNQ
EELMNTSVYSILHVGDHAEFVKNLLPKSLVNGVPWPQEATRRNSHTFNCRMLIHPPDEPGIENQEACQRYEVMQ
CFTVSQPKSIQEDGEDFQSCLICIARRLPRPPAITGVESFMTKQDTTGKIISIDTSSLRAAGRTGWEDLVRKCI
YAFFQPQGREPSYARQLFQEVMTRGTASSPSYRFILNDGTMLSAHTKCKLCYPQNADMQPFIMGIHIIDREHSG
LSPQDDTNSGMSVPRVNPSVNPSISPAHGVARSSTLPPSNSNIVSTRVNRQQSSDLHSSSHSNSSNSQGSFGCS
PGNQIVASVALNQGQASSQSTNPSLNLNNSPIEGTGISLAQFMSPRRQVSSGLATRPRMPSNSFPPNIPTLSSP
VGMTSTACNNSNRSYSNIPVTPLQSMNEGPNNSVGFSASSPVLRQISSQNSPSRLNIQPAKAESKDNKEIASIL
NEMIQSDNSSSDGKPLDSGLLHNNDRLSDGDNKYSQTSHKLVQLLTTTAEQQLRHADIDTSCKEVLSCAGTSSS
ASANSSGGSCPSSHSSLTERHKILHRLLQEGSPSDITTLSVEPDKKDSASASVSVTGQAQGNSSVKLELDASKK
KESKDHQLLRYLLDKDEKDLRSTPNLSLDDVKVKVEKKEQMDPCNTNPTPMTKPAPEEIKLESQNQFTADLDQF
DQLLPTLEKAAQLPGLCETERMDGAVTSVTVKSEILPPSLQSTTARPTSRLNRLPELELEAIDNQFGQPGTGDQ
IPWANNAVTAVNQNKPEDQCISSQLDELLCPPTTVEGRNDEKALLEQLVSFLSGKDETELAELDRALGIDKLVQ
GGGLDVLSERFPPQQATPPLMMEERPNLYSQPYSSPSPTANLTSPFQGMVRQKPSLGTMPVQVTPPRGAFSPGM
GMQPRQTLNRPPAAPNQLRLQLQQRLQGQQQLIHQNRQAILNQFAANAPVGINMRSGMQQQITPQPPLNAQMLA
QRQRELYSQQHRQRQLIQQQRAMLMRQQSFGNNLPPSSGLPVQMGNPRLPQGASQQFPYPPNYGTNPGTPPAST
SPFSQLAANPEAALASRNSMVNRGMTGNLGGQFGTGINPQMQQTVFQYPGSGMVPQGEANFAPSLSPGNSMVPM
PIPPPQTSLLQQTPPASGYQSPDMKAWQQGAMGNNNVFGQAVQNQPAPAQPGVYNNMSITVSMAGGNTNVQNMN
PMMGQMQMSSLQMPGMNTVCPEQINDSALRHTGLYCNQLSSTDLLKTEADGTQDKKTEEFFSVVTTD
>73Leopard|A0A6P4WER0|Mammalia|SRC1
MSGLGDSSSDPANPDSHKRKGSPCDTLASSTEKRRREQENKYLEELAELLSANISDIDSL
SVKPDKCKILKKTVDQIQLMKRMEQEKSTTDDEVQKSDISSSSQGVIEKESLGPLLLEAL
DGFFFVVNCEGRIVFVSENVTSYLGYNQEELMNTSVYSILHVGDHAEFVKNLLPKSLVNG
VPWPQEATRRNSHTFNCRMLIHPPDEPGTENQEACQRYEVMQCFTVSQPKSIQEDGEDFQ
SCLICIARRLPRPPAITGVESFMTKQDTTGKIISIDTSSLRAAGRTGWEDLVRKCIYAFF
QPQGREPSYARQLFQEVMTRGTASSPSYRFILNDGTMLSAHTKCKLCYPQSPDMQPFIMG

IHIIDREHSGLSPQDDTNSGMSISRVNPSVNSSISPAHGVARSSTLPPSSNNMVSPRVNR
QQSSDLHSSSHSNSSNSQGSFGCSPGNQIVASVALNQGQASSQSTNPSLNLNNSPMEGTG
ISLAQFMSPRRQVSSGLATRPRMPNNSFPPNIPTLSSPVGMTGTTCNNSNRSYSNIPVTS
LQSMNEGPNNSVGFSASSPVLRQMSSQNSPSRLNIQPTKAESKDNKEIASILNEMIQSDN
SSSDGKPLDSGLLHNNDRLSDGDNKYSQTSHKLVQLLTTTAEQQLRHADIDTSCKEVLSC
SGTSGSASGNSSSGSCPSSHSSLTERHKILHRLLQEGSPSDITTLSVEPDKKDSASTSVS
VTGQVQGNSSVKLELDASKKKESKDHQLLRYLLDKDEKDIRSTPNLSLDDVKVKVEKKEQ
MDPCNTNPTPMTKPAPEEIKLESQSQFTADLDQFDQLLPTLEKAAQLPGLCEPERMDGAV
TNVTIKSETLPASLQSTTARPTSRLNRLPELELEAIDNQFGQPGTGDQIPWANNTVTTVN
QNKPEDQCISSQLDELLCPPTTVEGRNDEKALLEQLVSFLSGKDETELAELDRALGIDKL
VQGGGLDVLSERFPPQQATPPLMMDERPNLYSQPYSSPSPTANLSSPFQGMVRQKPSLGT
MPVQVTPPRGAFSPSMGMQPRQTLNRPPAAPNQLRLQLQQRLQGQQQLIHQNRQAILNQF
AANTPVGINMRSGMQQQITPQPPLNAQMLAQRQRELYSQQHRQRQLIQQQRAMLMRQQSF
GNNLPPSSGLPVQMGNPRLPQGAPQQFPYPPNYGTNPGTPPASTSPFSQLAANPESTLAN
RNSMVNRGMTGNMGGQFGTGINPQMQQNVFQYPGSGMVPQGEASFAPSLSPGSSMVPMPI
PPPQSSLLQQTPPTSGYQSPDMKAWQQGAMGNNNVFSQAVQSQPTPAQPGVYNNMSITVS
MAGGNTNVQNMNPMMGQMQMSSLQMPGMNTVCPEQINDPALRHTGLYCNQLSSTDLLKTE
TDGTQQVQQVQVFADVQCTVNLVGGDPYLNQPGPLGTQKPTAGPQTPQAQQKSLLQQLLT
E
>74Bos indicus|A0A6P5CKJ8|Mammalia|SRC1
MSGLGDSSSDPANPDSHKRKGSPCDTLASSTEKRRREQENKYLEELAELLSANISDIDSLSVKPDKCKILKKTV
DQIQLMKRMEQEKSTTDDEVQKSDISSSSQGVIEKESLGPLLLEALDGFFFVVNCEGRIVFVSENVTSYLGYNQ
EELMNTSVYSILHVGDHAEFVKNLLPKSLVNGVPWPQEATRRNSHTFNCRMLIHRPDEPGPENQEACQRYEVMQ
CFTVSQPKSIQEDGEDFQSCLICIARRLPRPPAITGVESFMTKQDTTGKIISIDTSSLRAAGRTGWEDLVRKCI
YAFFQPQGREPSYARQLFQEVMTRGTASSPSYRFILNDGTMLSAHTKCKLCYPQSPDMQPFIMGIHIIDREHSG
LSPQDDTNPGMSVPRVNPSVNPSISPAHGVARSSTLPPSNSNMVPTRVNRQQSSDLHSSSHSNSSNSQGSFGCS
PGNQIVASVALNQGQASSQSTNPPLNLNNSPMEGTGISLAQFMSPRRQVSSGLATRPRMPNNSFPPNIPTLSSP
VSMTSTACNNSNRSYSNIPVTPLQSMNEGPNNSVGFSASSPVLRQMSSQNSPSRLNIQPAKAESKDNKEIASIL
NEMIQSDNSSNDSKPLDSGLLHNNDRLSDGDNKYSQTSHKLVQLLTTTAEQQLRHADIDTSCKEVLSCTGTSTS
ASTNSSSGSCPSSHSSLTERHKILHRLLQEGSPSDITTLSVEPDKKDSASTSVSVTGQVQGNSSVKLELDASKK
KESKDHQLLRYLLDKDEKDLRSTPNLSLDDVKVKVEKKEQMDPCNTNPASMTKPAPEEIKLESQSQFTADLDQF
DQLLPTLEKAPQLPGLCETERMDGAVSNVAIKSEILPSALQSTSARPTSRLNRLPELELEAIDNQFGQPGTGDQ
IPWANNTVTAVNQNKPEDQCISSQLDELLCPPTTVEGRNDEKALLEQLVSFLSGKDETELAELDRALGIDKLVQ
GGGLDVLSERFPPQQATPPLMMEERPNLYSQPYSSPSPTANLSSPFQGMVRQKPSLGTMPVQVTPPRGAFSPGM
GMQPRQTLNRPPAAPNQLRLQLQQRLQGQQQLIHQNRQAILNQFAANAPVGINMRSGMQQQITPQPPLNAQMLA
QRQRELYSQQHRQRQLIQQQRAMLMRQQSFGNSLPPSSGLPVQMGNPRLPQGAPQQFPYPPNYGTNPGTPPAST
SPFSQLAANPEAPLANRSSMVNRGMTGNMGGQFGTGINPQMQQNVFQYPGSGMVPQGEANFAPSLSPGSSMVPM
PIPPPQSSLLQQTPPTSGYQSPDMKAWQQGAMGNNNVFSQAIQNQPTPAQPGVYNNMSITVSMAGGNTNVQNMN
PMMGQMQMSSLQMPGMSTVCPEQINDPALRHTGLYCNQLSSTDLLKTEADGTQDKKTEEFFSVVTTD
>75Bos indicus|A0A6P5CLT2|Mammalia|SRC1
MSGLGDSSSDPANPDSHKRKGSPCDTLASSTEKRRREQENKYLEELAELLSANISDIDSLSVKPDKCKILKKTV
DQIQLMKRMEQEKSTTDDEVQKSDISSSSQGVIEKESLGPLLLEALDGFFFVVNCEGRIVFVSENVTSYLGYNQ
EELMNTSVYSILHVGDHAEFVKNLLPKSLVNGVPWPQEATRRNSHTFNCRMLIHRPDEPGPENQEACQRYEVMQ
CFTVSQPKSIQEDGEDFQSCLICIARRLPRPPAITGVESFMTKQDTTGKIISIDTSSLRAAGRTGWEDLVRKCI
YAFFQPQGREPSYARQLFQEVMTRGTASSPSYRFILNDGTMLSAHTKCKLCYPQSPDMQPFIMGIHIIDREHSG
LSPQDDTNPGMSVPRVNPSVNPSISPAHGVARSSTLPPSNSNMVPTRVNRQQSSDLHSSSHSNSSNSQGSFGCS
PGNQIVASVALNQGQASSQSTNPPLNLNNSPMEGTGISLAQFMSPRRQVSSGLATRPRMPNNSFPPNIPTLSSP
VSMTSTACNNSNRSYSNIPVTPLQSMNEGPNNSVGFSASSPVLRQMSSQNSPSRLNIQPAKAESKDNKEIASIL
NEMIQSDNSSNDSKPLDSGLLHNNDRLSDGDNKYSQTSHKLVQLLTTTAEQQLRHADIDTSCKEVLSCTGTSTS
ASTNSSSGSCPSSHSSLTERHKILHRLLQEGSPSDITTLSVEPDKKDSASTSVSVTGQVQGNSSVKLELDASKK
KESKDHQLLRYLLDKDEKDLRSTPNLSLDDVKVKVEKKEQMDPCNTNPASMTKPAPEEIKLESQSQFTADLDQF
DQLLPTLEKAPQLPGLCETERMDGAVSNVAIKSEILPSALQSTSARPTSRLNRLPELELEAIDNQFGQPGTGDQ
IPWANNTVTAVNQNKPEDQCISSQLDELLCPPTTVEGRNDEKALLEQLVSFLSGKDETELAELDRALGIDKLVQ
GGGLDVLSERFPPQQATPPLMMEERPNLYSQPYSSPSPTANLSSPFQGMVRQKPSLGTMPVQVTPPRGAFSPGM

GMQPRQTLNRPPAAPNQLRLQLQQRLQGQQQLIHQNRQAILNQFAANAPVGINMRSGMQQQITPQPPLNAQMLA
QRQRELYSQQHRQRQLIQQQRAMLMRQQSFGNSLPPSSGLPVQMGNPRLPQGAPQQFPYPPNYGTNPGTPPAST
SPFSQLAANPEAPLANRSSMVNRGMTGNMGGQFGTGINPQMQQNVFQYPGSGMVPQGEANFAPSLSPGSSMVPM
PIPPPQSSLLQQTPPTSGYQSPDMKAWQQGAMGNNNVFSQAIQNQPTPAQPGVYNNMSITVSMAGGNTNVQNMN
PMMGQMQMSSLQMPGMSTVCPEQINDPALRHTGLYCNQLSSTDLLKTEADGTQQVQQVQVFADVQCTVNLVGGD
PYLNQPGPLGTQKPTAGPQTPQAQQKSLLQQLLTE
>76Bos indicus|A0A6P5CMP1|Mammalia|SRC1
MSGLGDSSSDPANPDSHKRKGSPCDTLASSTEKRRREQENKYLEELAELLSANISDIDSLSVKPDKCKILKKTV
DQIQLMKRMEQEKSTTDDEVQKSDISSSSQGVIEKESLGPLLLEALDGFFFVVNCEGRIVFVSENVTSYLGYNQ
EELMNTSVYSILHVGDHAEFVKNLLPKSLVNGVPWPQEATRRNSHTFNCRMLIHRPDEPGPENQEACQRYEVMQ
CFTVSQPKSIQEDGEDFQSCLICIARRLPRPPAITGVESFMTKQDTTGKIISIDTSSLRAAGRTGWEDLVRKCI
YAFFQPQGREPSYARQLFQEVMTRGTASSPSYRFILNDGTMLSAHTKCKLCYPQSPDMQPFIMGIHIIDREHSG
LSPQDDTNPGMSVPRVNPSVNPSISPAHGVARSSTLPPSNSNMVPTRVNRQQSSDLHSSSHSNSSNSQGSFGCS
PGNQIVASVALNQGQASSQSTNPPLNLNNSPMEGTGISLAQFMSPRRQVSSGLATRPRMPNNSFPPNIPTLSSP
VSMTSTACNNSNRSYSNIPVTPLQSMNEGPNNSVGFSASSPVLRQMSSQNSPSRLNIQPAKAESKDNKEIASIL
NEMIQSDNSSNDSKPLDSGLLHNNDRLSDGDNKYSQTSHKLVQLLTTTAEQQLRHADIDTSCKEVLSCTGTSTS
ASTNSSSGSCPSSHSSLTERHKILHRLLQEGSPSDITTLSVEPDKKDSASTSVSVTGQVQGNSSVKLELDASKK
KESKDHQLLRYLLDKDEKDLRSTPNLSLDDVKVKVEKKEQMDPCNTNPASMTKPAPEEIKLESQSQFTADLDQF
DQLLPTLEKAPQLPGLCETERMDGAVSNVAIKSEILPSALQSTSARPTSRLNRLPELELEAIDNQFGQPGTGDQ
IPWANNTVTAVNQNKPEDQCISSQLDELLCPPTTVEGRNDEKALLEQLVSFLSGKDETELAELDRALGIDKLVQ
GGGLDVLSERFPPQQATPPLMMEERPNLYSQPYSSPSPTANLSSPFQGMVRQKPSLGTMPVQVTPPRGAFSPGM
GMQPRQTLNRPPAAPNQLRLQLQQRLQGQQQLIHQNRQAILNQFAANAPVGINMRSGMQQQITPQPPLNAQMLA
QRQRELYSQQHRQRQLIQQQRAMLMRQQSFGNSLPPSSGLPVQMGNPRLPQGAPQQFPYPPNYGTNPGTPPAST
SPFSQLAANPEAPLANRSSMVNRGMTGNMGGQFGTGINPQMQQNVFQYPGSGMVPQGEANFAPSLSPGSSMVPM
PIPPPQSSLLQQTPPTSGYQSPDMKAWQQGAMGNNNVFSQAIQNQPTPAQPGVYNNMSITVSMAGGNTNVQNMN
PMMGQMQMSSLQMPGMSTVCPEQINDPALRHTGLYCNQLSSTDLLKTEADGTQVQQVQVFADVQCTVNLVGGDP
YLNQPGPLGTQKPTAGPQTPQAQQKSLLQQLLTE
>77Phascolarctos cinereus|A0A6P5K2Y3|Mammalia|SRC1
MSGLGDSSTDPSNPDSHKRKGSPCDTLASSTEKRRREQENKYLEELAELLSANIGDIDSLSVKPDKCKILKKTV
DQIQQMKRMEQEKTATDDDVQKSDISSSSQGVIEKESLGPLLLEALDGFFFVVNREGRIVFVSENVTSYLGYNQ
EELMNTSVYSILHVGDHAEFVKNLLPKSLVNGVPWPQEATRRNSHTFNCRMLIRPPDEPGTENQEACQRYEVMQ
CFTVSQPKSIQEEGEDFQSCLICIARRLPRPAAITGSESFMTKQDTTGKIISIDTSSLRAAGRTGWEDLVRKCI
YAFFQPQGREPSYARQLFQEVMTRGTAFSPSYRFILNDGTMLSAHTKCKLCYPQSPDMQPFIMGIHIIDREHNV
LSPQDNTNSGMPLPRINPSVNPSISPAQGMARSSTLPPSNSSSMVTPRVNHQPNSDLHSSSSNNNSNTSQGSFG
CSPGNQVVASVALNQGQAGSQGSNHSLSLNNSPMDSTGITLAQFMSPRRQVSSGLATRPRMPSNSFSPNIPSLS
SPVGMVGSACGNPNRTYSNIPATSLQGINEGPSNSIGFSATSPVLRQLSSQNSPSRINMLPTKVESKDSKEITS
ILNEMVQSDNSSSDVKPLDSGLLHSSDKLSEGDNKYSQTSHKLVQLLATTAEQQLRHTDADTSGKDALSCSGTS
TSTANNSSSSGNTCPSSHSSLTERHKILHRLLQEGSPSDITTLSVEHDKKDNGSSSVSNQVPGNSNIKLELEAS
KKKESKDHQLLRYLLDKDEKDLRAAPTLSLDDVKVKVEKKEQMDPCNTTPTPLTKAAPEEIKLESQNQFPADLD
QFDPLLPTLEKASQLPGLCESERLESGGTGVTIKSEILPTALQSTPARPPPRLNRLPELELSGLDHQFGQPGID
DQVPWANNTITAMNQGPLNKPEDQCITSQLDELLCPPTTIEGRNDEKALLDQLVSFLSGKDETELAELDRALGI
DKLVQGGGLEVLTERFQPQQSTPPLMIEERSSLYSQPYSSASPTANLPSPFQGIVRQKPAMGTMPVQVPPPRGA
FPTNMGMQPRQTLTRPPAAPNQLRLQLQQRLQGQQQPPLNAQMLAQRQRELYSQQHRQRQLIQQQRALLMRQQS
FGNNLPSSSGLPVQMGTSRLPQGTPQQFPYPSSYGTNPGTPPTSTSPFSQLTSNPEASLASRNNMVNRGMTGNV
GGQFGTGINPQMQQSVFQYPGSGLGQQGEPNFTPSLSPGSPMMSLQIPPSQSPMLQQTQPSSGYQSPDMKTWQQ
GAIGSNNMFNQNQPTPAQPGVYNNMSITVSMAGGNTNVQNMSPLMGQMQMSSLQMPGMNTVCSEQINDPALRHT
GLYCNQLSSADILKTESDGSAQQVQQVQVFADVQCTVNLVGGDPYLNQPGPLGSQKPTSGPQNPQAQQKSLLQQ
LLTE
>78Phascolarctos cinereus|A0A6P5K3M9|Mammalia|SRC1
MSGLGDSSTDPSNPDSHKRKGSPCDTLASSTEKRRREQENKYLEELAELLSANIGDIDSLSVKPDKCKILKKTV
DQIQQMKRMEQEKTATDDDVQKSDISSSSQGVIEKESLGPLLLEALDGFFFVVNREGRIVFVSENVTSYLGYNQ
EELMNTSVYSILHVGDHAEFVKNLLPKSLVNGVPWPQEATRRNSHTFNCRMLIRPPDEPGTENQEACQRYEVMQ
CFTVSQPKSIQEEGEDFQSCLICIARRLPRPAAITGSESFMTKQDTTGKIISIDTSSLRAAGRTGWEDLVRKCI
YAFFQPQGREPSYARQLFQEVMTRGTAFSPSYRFILNDGTMLSAHTKCKLCYPQSPDMQPFIMGIHIIDREHNV

LSPQDNTNSGMPLPRINPSVNPSISPAQGMARSSTLPPSNSSSMVTPRVNHQPNSDLHSSSSNNNSNTSQGSFG
CSPGNQVVASVALNQGQAGSQGSNHSLSLNNSPMDSTGITLAQFMSPRRQVSSGLATRPRMPSNSFSPNIPSLS
SPVGMVGSACGNPNRTYSNIPATSLQGINEGPSNSIGFSATSPVLRQLSSQNSPSRINMLPTKVESKDSKEITS
ILNEMVQSDNSSSDVKPLDSGLLHSSDKLSEGDNKYSQTSHKLVQLLATTAEQQLRHTDADTSGKDALSCSGTS
TSTANNSSSSGNTCPSSHSSLTERHKILHRLLQEGSPSDITTLSVEHDKKDNGSSSVSNQVPGNSNIKLELEAS
KKKESKDHQLLRYLLDKDEKDLRAAPTLSLDDVKVKVEKKEQMDPCNTTPTPLTKAAPEEIKLESQNQFPADLD
QFDPLLPTLEKASQLPGLCESERLESGGTGVTIKSEILPTALQSTPARPPPRLNRLPELELSGLDHQFGQPGID
DQVPWANNTITAMNQGPLNKPEDQCITSQLDELLCPPTTIEGRNDEKALLDQLVSFLSGKDETELAELDRALGI
DKLVQGGGLEVLTERFQPQQSTPPLMIEERSSLYSQPYSSASPTANLPSPFQGIVRQKPAMGTMPVQVPPPRGA
FPTNMGMQPRQTLTRPPAAPNQLRLQLQQRLQGQQQLIHQNRQAILNQFAASAPIGINMRSGMQQQITPQPPLN
AQMLAQRQRELYSQQHRQRQLIQQQRALLMRQQSFGNNLPSSSGLPVQMGTSRLPQGTPQQFPYPSSYGTNPGT
PPTSTSPFSQLTSNPEASLASRNNMVNRGMTGNVGGQFGTGINPQMQQSVFQYPGSGLGQQGEPNFTPSLSPGS
PMMSLQIPPSQSPMLQQTQPSSGYQSPDMKTWQQGAIGSNNMFNQNQPTPAQPGVYNNMSITVSMAGGNTNVQN
MSPLMGQMQMSSLQMPGMNTVCSEQINDPALRHTGLYCNQLSSADILKTESDGSAQQVQQVQVFADVQCTVNLV
GGDPYLNQPGPLGSQKPTSGPQNPQAQQKSLLQQLLTE
>79Pteropus vampyrus|A0A6P6CAE6|Mammalia|SRC1
MSGLGDSSSDPANPDSHKRKGSPCDTLASSTEKRRREQENKYLEELAELLSANISDIDSLSVKPDKCKILKKTV
DQIQLMKRMEQEKSATDDEVQKSDISSSSQGVIEKESLGPLLLEALDGFFFVVNCEGRIVFVSENVTSYLGYNQ
EELMNTSVYSILHVGDHAEFVKNLLPKSLVNGVPWPQEATRRNSHTFNCRMLIHPPDEPGIENQEACQRYEVMQ
CFTVSQPKSIQEDGEDFQSCLICIARRLPRPPAITGVESFMTKQDTTGKIISIDTSSLRAAGRTGWEDLVRKCI
YAFFQPQGREPSYARQLFQEVMTRGTASSPSYRFILNDGTMLSAHTKCKLCYPQNADMQPFIMGIHIIDREHSG
LSPQDDTNSGMSVPRVNPSVNPSISPAHGVARSSTLPPSNSNIVSTRVNRQQSSDLHSSSHSNSSNSQGSFGCS
PGNQIVASVALNQGQASSQSTNPSLNLNNSPIEGTGISLAQFMSPRRQVSSGLATRPRMPSNSFPPNIPTLSSP
VGMTSTACNNSNRSYSNIPVTPLQSMNEGPNNSVGFSASSPVLRQISSQNSPSRLNIQPAKAESKDNKEIASIL
NEMIQSDNSSSDGKPLDSGLLHNNDRLSDGDNKYSQTSHKLVQLLTTTAEQQLRHADIDTSCKEVLSCAGTSSS
ASANSSGGSCPSSHSSLTERHKILHRLLQEGSPSDITTLSVEPDKKDSASASVSVTGQAQGNSSVKLELDASKK
KESKDHQLLRYLLDKDEKDLRSTPNLSLDDVKVKVEKKEQMDPCNTNPTPMTKPAPEEIKLESQNQFTADLDQF
DQLLPTLEKAAQLPGLCETERMDGAVTSVTVKSEILPPSLQSTTARPTSRLNRLPELELEAIDNQFGQPGTGDQ
IPWANNAVTAVNQNKPEDQCISSQLDELLCPPTTVEGRNDEKALLEQLVSFLSGKDETELAELDRALGIDKLVQ
GGGLDVLSERFPPQQATPPLMMEERPNLYSQPYSSPSPTANLTSPFQGMVRQKPSLGTMPVQVTPPRGAFSPGM
GMQPRQTLNRPPAAPNQLRLQLQQRLQGQQQLIHQNRQAILNQFAANAPVGINMRSGMQQQITPQPPLNAQMLA
QRQRELYSQQHRQRQLIQQQRAMLMRQQSFGNNLPPSSGLPVQMGNPRLPQGASQQFPYPPNYGTNPGTPPAST
SPFSQLAANPEAALASRNSMVNRGMTGNLGGQFGTGINPQMQQTVFQYPGSGMVPQGEANFAPSLSPGNSMVPM
PIPPPQTSLLQQTPPASGYQSPDMKAWQQGAMGNNNVFGQAVQNQPAPAQPGVYNNMSITVSMAGGNTNVQNMN
PMMGQMQMSSLQMPGMNTVCPEQINDSALRHTGLYCNQLSSTDLLKTEADGTQVQQVQVFADVQCTVNLVGGDP
YLNQPGPLGTQKPTAGPQTPQAQQKSLLQQLLTE
>80Rousettus aegyptiacus|A0A7J8FKM1|Mammalia|SRC1
MSGLGDSSSDPANPDSHKRKGSPCDTLASSTEKRRREQENKYLEELAELLSANISDIDSLSVKPDKCKILKKTV
DQIQLMKRMEQEKSTTDDEVQKSDISSSSQGVIEKESLGPLLLEALDGFFFVVNCEGRIVFVSENVTSYLGYNQ
EELMNTSVYSILHVGDHAEFVKNLLPKSLVNGVPWPQEATRRNSHTFNCRMLIHPPDEPGIENQEACQRYEVMQ
CFTVSQPKSIQEDGEDFQSCLICIARRLPRPPAITGVESFMTKQDTTGKIISIDTSSLRAAGRTGWEDLVRKCI
YAFFQPQGREPSYARQLFQEVMTRGTASSPSYRFILNDGTMLSAHTKCKLCCPQSADMQPFIMGIHIIDREHSG
LSPQDDTNSGMSVPRVNPSVNPSISPAHGVARSSTLPPSNSNVVSTRVNRQQSSDLHSSSHSNSSNSQGSFGCS
PGNQIVASVALNQGQASSQSTNPSLNLNNSPMEGTGISLAQFMSPRRQVSSGLATRPRMPSNSFPPTLSSPVGM
TSTACNNSNRSYSNIPVTPLQSMNEAPNNSVGFSASSPVLRQISSQNSPSRLNIQPAKAESKDNKEIASILNEM
IQSDNNSSDGKPLDSGLLHNNDRLSDGDNKYAQTSHKLVQLLTTTAEQQLRHADIDTSCKEVLSCAGTSSSASA
NSSGGSCPSSHSSLTERHKILHRLLQEGSPSDLTTLSVEPDKKDSASASVSVTGQAQGNSSVKLELDASKKKES
KDHQLLRYLLDKDEKDLRSTPNLSLDDVKVKVEKKEQMDPCNTNPTPMTKPAPEEIKLESQNQFTADLDQFDQL
LPTLEKAAQLPGLCETERMDGAVTSVTVKSEILPPSLQPTTARPTSRLNRLPELELEAIDNQFGQPGTGDQIPW
ANNAVTAVNQNKPEDQCISSQLDELLCPPTTVEGRNDEKALLEQLVSFLSGKDETELAELDRALGIDKLVQGGG
LDVLSERFPPQQATPPLMMEERPNLYSQPYSSPSPTANLTSPFQGMVRQKPSLGTMPVQVTPPRGAFSPGMGMQ
PRQTLNRPPAAPNQLRLQLQQRLQGQQQLIHQNRQAILNQFAANAPVGINMRSGMQQQITPQPPLNAQMLAQRQ
RELYSQQHRQRQLIQQQRAMLMRQQSFGNNLPPSSGLPVQMGNPRLPQGASQQFPYPPNYGTNPGTPPASTSPF
SQLAANPEAALASRNSMVNRGMTGNLGGQFGTGINPQMQQTVFQYPGSGMVPQGEANFAPSLSPGNSMVPMPIP

PPQTSLLQQTPPASGYQSPDMKAWQQGAMGNNNVFGQAVQNQPAPAQPGMYNNMSITVSMAGGNTNVQNMNPMM
GQMQMSSLQMPGMNAVCPEQINDSALRHTGLYCNQLSSTDLLKTEADGTQDKKTEEFFSVVTTD
>81Molossus molossus|A0A7J8HI04|Mammalia|SRC1
MSGLGENSLDPLVPDSRKRKLPCDIPGQGLTCSGEKWRREQESKYIEELAELISANLSDIDNFNVKPDKCAILK
ETVRQIRQIKEQGKAISNDDDVQKADVSSTGQGVIDKDSLGPLLLQALDGFLFVVNRDGNIVFVSENVTQYLQY
KQEDLVNTSVYNILHEEDRKDFLKNLPKSTVNGVSWTNETQRQKSHTFNCRMLMKTPHDIVEDMNTSPEMRQRY
ETMQCFALSQPRAMMEEGEDLQSCMICVARRITTGERAFPSNPESFITRHDLSGKLVNIDTNSLRSSMRPGFED
IIRRCIQRFFSLNDAQSWSQKRHYQEVIFISNRIFPPIAYIHGHAETPMYRFSLADGTLVTAQTKSKLFRNPVT
NDRHGFVSTHFLQREQNGYRPNPNTVGPGIRPPAAGCSNSVGGMSMSPNQGLQMLNSRTYGLADPSTTGQMSGA
RYGASSNIASMNPGPAMQSPSSYQNNYGLNMSSPPHGSPGLGSNQQNIMISPRNRGSPKMASHQFSPVAGVHSP
MGSSGSTGSHSFSSSSLSALQAISEGVGTSLLSTLSSPGPKLDNSPNMNITQPSKGNSQDSKSPLGLYCDQNPV
ESSMCQSNSRDHLSDKDSKESNVEGSENQRSSQESKGHKKLLQLLTCSSDDRGHSSLTNSPLDSSCKDSSISVT
SPSGVSSSTSGGVSSTSNMHGSLLQEKHRILHKLLQNGNSPAEVAKITAEATGKDTSSTTFCVEGTVKQEQLSP
KKKENNALLRYLLDRDDPSDTLSKEVQPKVEGVDNKMSQCSSSSVPSSSQEKDAKIKTEANEEGSGDLDNLDAI
LGDLTSSDFYNNSISTNGSNLGTKQQMFQGTNSLGLRGTQSVQSIRPPYSRAVSLDSPVSVGSSPPVKNISAFP
MLPKQPMLSGNPRMMDSQENYGSNMRDWGLPNSKTSRMEPLSSNSMGRPGVDYNASLPRPTMGGSMPTLPLRSS
SIPGVRPMLQQQQQQQQQQQQQMLQMRPGEIPMGMGVSPYGPAVPSNQSGSWPDSMLSMEQSPHGTQNRPLLRN
SLDDLLGPPSNLEGQNDERALLDQLHTLLSNTDATGLEEIDRALGIPELVNQGQTLEPKQDVFQGQEAAVMMDQ
KAALYGQTYPAQGPPMQGGFNLQGQSPSFNSMMSQMNQQGNFPLQGMHPRANIMRPRTNTPKQLRMQLQQRLQG
QQFLNQSRQALEMKMENPTAGGAAMMRPMMQPQQGFLNAQMVAQRSRELLSHHFRQQRVAAMMMQQQQQQQQQ
QQPPQPQAFSPPPNVTASPSMDGVLAGPAMPQAPPQQFPYPPNYGMGQQPDPAFGRVSSPPNAMMSSRMGPSQN
PLMQHPQTAPMYQPSEMKGWPSGNLARNSSFPQQQFVHQGPPAAYSMVHMNGSSGPMGQMNMNSMPMSGMPMGP
DQKYC
>82Bucorvus abyssinicus|A0A7K4Z0Q0|Aves|SRC1
MSGLGDSSTDPANPDSRKRKGSPCDTAQSNEKRRREQENKYLEELAELLSANIGDIDTLSVKPDKCKILKKTVD
QIQQMKRLEQEKAADDEVQKSDISSSSQGVIEKESLGPLLLEALDGFFFVVNREGRIVFVSENVTSYLGYNQEE
LMNTSVYSILHVGDHAEFVKNLLPKSLVNGVPWPQEATRRNSHTFSCRMLIRPPDEPGAENQEARQRYEVMQCF
TVSQPKSFKEEGEDFQSCLICIARRLPRPAAVTPSESFVTKQDTTGKIISIDTSSLRAAGRTGWEDLVRKCIYA
FFQPQGREPSYAKQLFQEVMTRGTAFSPSYRFTLSDGTVLSAHTKCKLCYPSSPEMQPFIMGIHVIDRDHGMLS
PQENTNSAMSLPRVSPSLNPSISPVQGMALPPSIPPSNGSMLATNVNQQPGASLHNSNSSTSQTSFGCSPGNQI
GANVALSQGQAGPQNSNHTLNLSSSPMNSPGITPPQFMSPRHRVSPGLVPRPRVPGNPFSPTMPTMHSPVGMAN
SGSGGGCGTGGSRPFPSAPINSMQGLTEGPSTPVGYSTPPPVLRQLSSQSSPGRLAMQPMKAESKDSKEIASIL
SEMIQSDGASGDSKPLDSGMLHAGDRLSEGDSKCSQATSHKLVQLLATTAEQQLRHADADTSCKDSLACAGTTG
TLASSNPPSSTCPSSHSSLTERHKILHRLLQEGSPSDITTLGMEHEKKENTSNTTPTAQSQLPVTPDIKVEPDA
LKKKDVKDHQLLRYLLDKDEKELAAAPALSLDDVKVKVEKTEQMEPCNTAPVPLTKPPAAEEVKPETQGQFAAE
LEQLDQLLPSLEKAAQLPGLCGPERNESGVASVAVKPDMLTAPLQPSATARAPTGLNPMSSGQGVQAGRTPFEF
CDPLEPAQMRPVPCPVSNGSSPHARGSSQQGRGTVTTPNPFPPDTGMPELEMGVPDHQFGQPGMAEQMPWSENS
MAAMNRSTLNKPEDQCITSQLDELLCPPTTPEGRNDEKALLEQLVSFLSGKDETELAELDRALGIDKLVQGGGL
EALTDRFQPQQATPPLMMEQKPGMYPQPYSSASPTANLPGAFPGMVRQKPSFGPMPVQVPPPRGAFPTNMAMQP
RQTLNRPPTAPNQLRLQLQQRLQGQQQLIHQNRQAILNQFTANSPVSINMRAGMQQQITPQPPLNAQMLAQRQR
ELYSQQHRQRQLMQQRAILMRQQSFGNNLPPSTGLPVQIGAARLPQAPPQQFPYPPNYGTTPGNPPTSTSPFSP
LASNPEAALANRSSMVNRGMMGGVAGQFSAGMTPQMQQNIFQYSGSGMGQQSEPAFAPSLSPSSPLMSPQLPPS
QSPMLQPAPPTPGYQSPDMKTWQQGAMGNSNVFSQAGQNQPAPAQQGMYNNMSITVSMAGGNTNVQNMNPMAGQ
MQMNSLQMPGMNSMCSEQVNDPALRPAGLYCNQLSSTDLLKTEADGAQVQQVQVFADVQCTVNLVGDPYLNPPA
PLGTPKNAAGPPPPPGQQKSLLQQLLTE
>83Chunga burmeisteri|A0A7K5GYB0|Aves|SRC1
MSGLGDSSTDPANPDSRKRKGSPCDTAQSNEKRRREQENKYLEELAELLSANIGDIDTLSVKPDKCKILKKTVD
QIQQMKRLEQEKAADDEVQKSDISSSSQGVIEKESLGPLLLEALDGFFFVVNREGRIVFVSENVTSYLGYNQEE
LMNTSVYSILHVGDHAEFVKNLLPKSLVNGVPWPQETTRRNSHTFSCRMLIRPPDEPGAENQEARQRYEVMQCF
TVSQPKSFKEEGEDFQSCLICIARRLPRPAAVTPSESFVTKQDTTGKIISIDTSSLRAAGRTGWEDLVRKCIYA
FFQPQGREPSYARQLFQEVMTRGTAFSPSYRFTLSDGTVLSAHTKCKLCYPSSPEMQPFIMGIHVIDRDHGMLS
PQENTNSTMSLPRVSPSLNPSISPGQGMALPPSIPPSNGSMLATNVNQQPGAGLHNSNSSTSQTSFGCSPGNQI
GANVALSQGQAGPQNSNHTLNLNSSPMNSPGITPPQFMSPRHRVSPGLVPRPRVPSNPFSPTMPTMHSPVGMAN
SGSGGSCGTGGTRPFPSAPINSMQGLTEGPSTPVGYSTPPPVLRQLSSQSSPGRLAMQPMKAESKDSKEIASIL

SEMIQSDSGSGDGKPLDSGMLHTGDRLSEGDSKCSQATSHKLVQLLATTAEQQLRHADADTSCKDSLACAGTAG
TLASSNPPSSTCPSSHSSLTERHKILHRLLQEGSPSDITTLAMEHEKKENVPNPTTTPTAQSQLPGTPDVKLEP
DALKKKDVKDHQLLRYLLDKDEKELAAAPALSLDDVKVKVEKTEQMEPCNTAPVPLAKPPATEEAKLETQGQFA
AELEQLDQLLPSLEKAAQLPGLCGPERSESGVAGVTVKPEMLATPLQPSTAARAPTGLNPMNSGQGVQAGRTPF
EFCDPLEPAQLRPVPCPASNGSSPHARGPSQQGRGTVTAPNPFPPDTGMPELEMGVPDHQFGQPGMGEQIPWTD
NSMAAMNRGTLNKPEDQCITSQLDELLCPPTTPEGRNDEKALLEQLVSFLSGKDETELAELDRALGIDKLVQGG
GLEALTERFQPQQATPPLMMEQKPGMYPQPYSSASPTTNLPGAFPGMVRQKPSFGPMPVQVPPPRGAFPTNMAM
QPRQTLNRPPTAPNQLRLQLQQRLQGQQQLIHQNRQAILNQFAANSPVGINMRAGMQQQITPQPPLNAQMLAQR
QRELYSQQHRQRQLMQQRAILMRQQSFGNNLPPSAGLPVQIGAARLPQAPPQQFPYPPNYGTTPGNPPTSTSPF
SPLASNPEAALANRSSMVNRGMMGSVGGQFGAGMTPQMQQNIFQYSGSGMGQQSEPAFAPSLSPSSPLMSPQLP
PSQSPMLQPAPPTPGYQSPDMKTWQQGAMGNSNVFSQTGQSQPAPAQQGMYNNMSITVSMAGGNTNVQNMNPMA
GQMQMNSLQMPGMNSMCSEQVNEPALRPAGLYCNQLPSTDLLKTEADGAQ
>84Chroicocephalus maculipennis|A0A7K5NNA7|Aves|SRC1
MSGLGDSSTDPANSDSRKRKGSPCDTAQSNEKRRREQENKYLEELAELLSANIGDIDTLSVKPDKCKILKKTVD
QIQQMKRLEQEKAADDEVQKSDISSSSQGVIEKESLGPLLLEALDGFFFVVNREGRIVFVSENVTGYLGYNQEE
LMNTSVYSILHVGDHAEFVKNLLPKSLVNGVPWPQEATRRNSHTFSCRMLIRPPDEPGAENQEARQRYEVMQCF
TVSQPKSFKEEGEDFQSCLICIARRLPRPAAVTPSESFVTKQDTTGKIISIDTSSLRAAGRTGWEDLVRKCIYA
FFQPQGREPSYAKQLFQEVMTRGTAFSPSYRFTLSDGTVLSAHTKCKLCYPSSPEMQPFIMGIHVIDRDHGMLS
PQENTNSAMSLPRVSPSLNPSISPGQGMALPPSIPPSNGSMLATNVNQQPGAGLHNSNSSTSQTSFGCSPGNQI
GANVALSQGQAGPQNSNHTLNLNSSPMNSPGITPPQFMSPRHRVSPGLVPRPRVPSNPFSPTMPTMHSPVGMAN
SGSGGGGGTGGARPFPSAPINSMQGLTEGPSTPVGYSTPPPVLRQLSSQSSPGRLAMQPMKAESKDSKEIASIL
SEMIQSDGGSGDGKPLDSGMLHAGDRLSEGDSKCSQATSHKLVQLLATTAEQQLRHADADTSCKDSLACAGTAG
TLASSNPPSSTCPSSHSSLTERHKILHRLLQEGSPSDITTLAMEHEKKENAPNPATTPTAQSQLPGTPDIKLEP
DALKKKDVKDHQLLRYLLDKDEKELAAAPALSLDDVKVKVEKTEQMEPCNTAPVPLAKPPAAEEVKLETQGQFA
AELEQLDQLLPSLEKAAQMPGLCGPERSESGVAGVTVKPEMLAAPLQPSTAARAPTGLNPMPSGQGVQAGRPPF
EFCDPLEPAQLRPVPCPASNGSSPHARGPSQQGRGTVTAPNPFPPDTGMPELEMGVPDHQFGQPGMGEQIPWTD
NSMAAMNRGALNKPEDQCITSQLDELLCPPTTPEGRNDEKALLEQLVSFLSGKDETELAELDRALGIDKLVQGG
GLEALTERFQPQQATPPLMMDQKPGMYPQPYSSASPTTNLPGAFPGMVRQKPSFGPMPVQVPPPRGAFPTNMAM
QPRQTLNRPPTAPNQLRLQLQQRLQGQQQLIHQNRQAILNQFAANSPVGINMRAGMQQQITPQPPLNAQMLAQR
QRELYSQQHRQRQLMQQRAILMRQQSFGNNLPPSAGLPVQIGAARLPQAPPQQFPYPPNYGTNPGNPPTSTSPF
SPLASNPEAALANRSSMVNRGMMGGVGGQFGAGMTPQMQQNIFQYSGSGMGQQSEPAFAPSLSPSSPLMSPQLP
PSQSPMLQPAPPTPGYQSPDMKTWQQGAMGNSNVFSQAGQSQPAPAQQGMYNNMSITVSMAGGNTNVQNMNPMA
GQMQMNSLQMPGMNSMCSEQVNDPALRPAGLYCNQLSSTDLLKTEADGAQQVQQVQVFADVQCTVNLVGDPYLN
PPAPLGTPKNAAGPPPPPGQQKSLLQQLLTE
>85Dromas ardeola|A0A7K5XHT5|Aves|SRC1
MSGLGDSSTDPANPDSRKRKGSPCDTAQSNEKRRREQENKYLEELAELLSANIGDIDTLSVKPD
KCKILKKTVDQIQQMKRLEQEKAADDEVQKSDISSSSQGVIEKESLGPLLLEALDGFFFVVNRE
GRIVFVSENVTGYLGYNQEELMNTSVYSILHVGDHAEFVKNLLPKSLVNGVPWPQEATRRNSHT
FSCRMLIRPPDEPGAENQEARQRYEVMQCFTVSQPKSFKEEGEDFQSCLICIARRLPRPAAVTP
SESFVTKQDTTGKIISIDTSSLRAAGRTGWEDLVRKCIYAFFQPQGREPSYAKQLFQEVMTRGT
AFSPSYRFTLSDGTVLSAHTKCKLCYPTSPEMQPFIMGIHIIDRDHGMLSPQENTNSAMSLPRV
SPSLNPSISPGQGMALPPSIPPSNGSMLATNVNQQPGAGLHNSNSSTSQTSFGCSPGNQIGANV
ALSQGQAGPQNSNHTLNLNSSPMNSPGITPPQFMSPRHRVSPGLVPRPRVPSNPFSPTMPTMHS
PVGMANSGSGGGGGTGGARPFPSAPINSMQGLTEGPSTPVGYSTPPPVLRQLSSQSSPGRLAMQ
PMKAESKDSKEIASILSEMIQSDGGSGDGKPLDSGMLHAGDRLSEGDSKCSQATSHKLVQLLAT
TAEQQLRHADADTSCKDSLACAGTAGTLASSNPPSSTCPSSHSSLTERHKILHRLLQEGSPSDI
TTLAMEHEKKENAPNPATTPTAQSQLPGTPDIKLEPDALKKKDVKDHQLLRYLLDKDEKELTAA
PALTLDDVKVKVEKTEQMEPCNTAPVPLAKPPAAEEVKLETQGQFAAELEQLDQLLPSLEKAAQ
MPGLCGPERSDSSVAGVAVKPEMLAAPLQPSTAARAPTGLNPMTSGQGVQAGRPPFEFCDPLEP

AQLRPVPCPASNGSSPHARGPSQQGRGTVTAPNPFPPDTGMPELEMGVPDHQFGQPGMGEQIPW
TDNSMAAMNRGALNKPEDQCITSQLDELLCPPTTPEGRNDEKALLEQLVSFLSGKDETELAELD
RALGIDKLVQGGGLEALTERFQPQQATPPLMMEQKPGMYPQPYSSASPTTNLPGAFPGMVRQKP
SFGPMPVQVPPPRGAFPTNMAMQPRQTLNRPPTAPNQLRLQLQQRLQGQQQLIHQNRQAILNQF
AANSPVGINMRAGMQQQITPQPPLNAQMLAQRQRELYSQQHRQRQLMQQRAILMRQQSFGNNLP
PSAGLPVQIGAARLPQAPPQQFPYPPNYGTTPGNPPTSTSPFSPLASNPEAALANRSSMVNRGM
MGGVGGQFGAGMTPQMQQNIFQYSGSGMGQQSEPAFAPSLSPSSPLMSPQLPPSQSPMLQPAPP
TPGYQSPDMKTWQQGAMGNSNVFSQAGQSQPAPAQQGMYNNMSITVSMAGGNTNVQNMNPMAGQ
MQMNSLQMPGMNSMCSEQVNDPALRPAGLYCNQLSSTDLLKTEADGAQ

>86Upupa epops|A0A7K6AXE2|Aves|SRC1
MSGLGDSSTDPGNPDSRKRKGSPCDTAQSNEKRRREQENKYLEELAELLSANIGDIDTLSVKPDKCKILKKTVD
QIQQMKRLEQEKAADDEVQKSDISSSSQGVIEKESLGPLLLEALDGFFFVVNREGRIVFVSENVTSYLGYNQEE
LMNTSVYSILHVGDHAEFVKNLLPKSLVNGVPWPQEATRRNSHTFSCRMLIRPPDEPGTENQEARQRYEVMQCF
TVSQPKSFKEEGEDFQSCLICIARRLPRPAAVTPSESFVTKQDTTGKIISIDTSSLRAAGRTGWEDLVRKCIYA
FFQPQGREPSYAKQLFQEVMTRGTAFSPSYRFTLSDGTVLSAHTKCKLCYPSSPEMQPFIMGIHVIDRDHGMLS
PQENTNSTMSLPRVSPSLNTSISPGQGMALPPSIPPSNGSMLASNVNQQPGAGLHNSNSSTSQTNFGCSPGNQI
GANVALSQGQSGPQNSNHTLNLNSSPMNSPGITPPQFMSPRHRVSPGLVPRPRGPSNPFSPTMPTMHSPVGMAN
NGSSGNCGTGGTRPFPNAPINSMQGLTEGPSTPVGYSTPPPVLRQISSQSSPGRLAMQPMKTESKDSKEIASIL
SEMIQSDGGSGDSKPLDSAMLHSSDRLSEGDTKCPQATSHKLVQLLATTAEQQLRHVDADTSCKDSLACAGTAG
ALASSNPPSSSCPSSHSSLTERHKILHRLLQEGSPSDITTLGMDHEKKENTPNPTTTPAAQSQLPGTPDIKLEP
DALKKKDVKDHQLLRYLLDKDEKELAAAPALSLDDVKVKVEKTEQMEPCNTAPVPLAKPPATEEVKLETQGQFA
AELEQLDQLLPSLEKAAQLPGLCGPDRNESGVAGVTVKPEMLAAPMQPSTAARAPTGLNPMSSGQGVQAGRAPF
EFCDPLEPGQLRPGPCPTSNGSSPHARGPSQQGRGTVTAPNPFPTDTGMPELEMGVPDHQFGQPGMGEQIPWTD
NSMAAMNRGAMSKPEDQCITSQLDELLCPPTTPEGRNDEKALLEQLVNFLSGKDETELAELDRALGIDKLVQGG
GLEALTERFQPQQATPPLMMEQKPGMYPQPYSSASPTANLPGAFPGMVRQKPSFGPMPVQVPPPRGAFPTNMAM
QPRQTLNRPPTAPNQLRLQLQQRLQGQQQLIHQNRQAILNQFTANSPVGINMRAGMQQQITPQPPLNAQMLAQR
QRELYSQQHRQRQLMQQRAILMRQQSFGNNLPPSTGLPVQIGAARLPQAPPQQFPYPPNYGTTPGNPPTSTSPF
SPLASNPEAALANRGSMVNRGMMGSVGGQFGAGMTPQMQQNIFQYSGSGMGQQSEPAFAPSLSPSSPLMSPQLP
PSQSPMLQPAPPTPGYQSPDMKTWQQGAMGNSNVFSQAGQNQPAPAQQGMYNNMSITVSMAGGNTNVQNMNPMA
GQMQMNSLQMPGMNSMCSEQVNDSALRPTGLYCNQLSSTDLLKTEADGAQQVQQVQVFADVQCTVNLVGDPYLN
PPAPLGTPKNAAGPPPPPGQQKSLLQQLLTE
>87Cochlearius cochlearius|A0A7K8Q017|Aves|SRC1
MSGLGDSSTDPANPDSRKRKGSPCDTAQSNEKRRREQENKYLEELAELLSANIGDIDTLSVKPDKCKILKKTVD
QIQQMKRLEQEKAADDEVQKSDISSSSQGVIEKESLGPLLLEALDGFFFVVNREGRIVFVSENVTSYLGYNQEE
LMNTSVYSILHVGDHAEFVKNLLPKSLVNGVPWPQEATRRNSHTFSCRMLIRPPDEPGAENQEARQRYEVMQCF
TVSQPKSFKEEGEDFQSCLICIARRLPRPAAVAPSESFVTKQDTTGKIISIDTSSLRAAGRTGWEDLVRKCIYA
FFQPQGREPSYAKQLFQEVMTRGTAFSPSYRFTLSDGTVLSAHTKCKLCYPSSPEMQPFIMGIHVIDRDHGMLS
PQENTNSAMSLPRVSPSLNPSISPGQGMALPPSIPPSNGSMLATNVNQQPGAGLHNSNSSTSQTSFGCSPGNQI
GANVALSQGQAGPQNSNHTLNLNSSPMNSPGITPPQFMSPRHRVSPGLVPRPRVPSNPFSPTMPTMHSPVGMAN
SGSGGGGGTAGTRPFPSAPINSMQGLTEGPSTPVGYSTPPPVLRQLSSQSSPGRLAMQPMKAESKDSKEIASIL
SEMIQSDGGSGDSKPLDSGMLHAGDRLSEGDSKCSQATSHKLVQLLATTAEQQLRHADADTSCKDSLACAGTAG
PLASGNPPSSTCPSSHSSLTERHKILHRLLQEGSPSDITTLAMEHEKKENTPNPATTPTAQSQLPGTPDIKLEP
DALKKKDVKDHQLLRYLLDKDEKELAAAPALSLDDVKVKVEKTEQMEPCNTAPVPLAKPPAAEEVKLETQGQFA
AELEQLDQLLPSLEKAAQLPGLCGPERNESGVAGVTVKPEMLAAPLQPSAAARAPTGLNPMNSGQGVQAGRTPF
EFCDPLEPAQLRPVPCPASNGSSPHARGPSQQGRGTVTAPNPFPPDAGMPELEMGVPDHQFDQPGMGEQIPWTD
NSMSAMNRGALNKPEDQCITSQLDELLCPPTTPEGRNDEKALLEQLVSFLSGKDETELAELDRALGIDKLVQGG
GLEALTERFQPQQATPPLMMEQKPGMYPQPYSSASPTTNLPGAFPGMVRQKPSFGPMPVQVPPPRGAFPTSMAM
QPRQTLNRPPTAPNQLRLQLQQRLQGQQQLIHQNRQAILNQFAASSPGGINMRAGMQQQITPQPPLNAQMLAQR
QRELYSQQHRQRQLMQQRAILMRQQSFGNNLPPSAGLPVQIGAARLPQAPPQQFPYPPNYGTTPGNPPTSTSPF
SPLASNPEAALANRSSMVNRGMMGGVGGQFGAGMTPQMQQNIFQYSGSGMGQQSEPAFAPSLSPSSPLMSPQLP

PSQSPMLQPAPPTPGYQSPDMKTWQQGAMGNSSVFSQAGQSQPAPAQQGMYNNMSITVSMAGGNTNVQNMNPMA
GQMQMNSLQMPGMNSMCSEQVNDPALRPAGLYCNQLSSTDLLKTEADGAQQVQQVQVFADVQCTVNLVGDPYLN
PPAPLGTPKNAAGPPPPPGQQKSLLQQLLTE
>88Oceanites oceanicus|A0A7K8UC72|Aves|SRC1
MSGLGDSSTDPANPDSRKRKGSPCDTAQSNEKRRREQENKYLEELAELLSANIGDIDTLSVKPDKCKILKKTVD
QIQQMKRLEQEKAADDEVQKSDISSSSQGVIEKESLGPLLLEALDGFFFVVNREGRIVFVSENVTSYLGYNQEE
LMNTSVYSILHVGDHAEFVKNLLPKSLVNGVPWPQEATRRNSHTFSCRMLIRPPDEPGAENQEARQRYEVMQCF
TVSQPKSFKEEGEDFQSCLICIARRLPRPTAVTPSESFVTKQDTTGKIISIDTSSLRAAGRTGWEDLVRKCIYA
FFQPQGREPSYAKQLFQEVMTRGTAFSPSYRFTLSDGTVLSAHTKCKLCYPSSPEMQPFIMGIHVIDRDHGMIS
PQENTNSTMSLPRVSPSLNPSISPGQGMALPPSIPPSNGSMLATNVNQQPGAGLHSSNSSTSQTSFGCSPGNQI
GANVALSQGQAGPQNSNHTLNLNSSPMNSPGITPPQFMSPRHRVSPGLVPRPRMPSNPFSPTMPTMHSPVGMAN
SGSGGGSGTGSTRPFPSAPINSMQGLTEGPSTPVGYSTPPPVLRQLSSQSSPGRLAMQPMKTESKDSKEIASIL
SEMIQSDGGSGDGKPLDSGMLHTGDRLSEGDSKCSQATSHKLVQLLATTAEQQLRHADADTSCKDSLACAGTAG
TLASGNPPSSTCPSSHSSLTERHKILHRLLQEGSPSDITTLAMEHEKKENAPNPATTATAQSQLPGTPDIKLEP
DALKKKDVKDHQLLRYLLDKDEKELAAAPALSLDDVKVKVEKTEQMEPCNTAPPVPLAKPPATEEVKLETQGQF
AAELEQLDQLLPSLEKAAQLPGLCGPERNESGVAGVTVKPEMLATPLQPSTAARAPTGLNPMNSGQGVQAGRTP
FEFCDPLEPAQLRPVPCPTSNGSSPHARGPSQQGRGAVTAPNPFPPDTGMPELEMGVPDHQFGQPGMGEQIPWT
DNSMAAMNRGALNKPEDQCITSQLDELLCPPTTPEGRNDEKALLEQLVSFLSGKDETELAELDRALGIDKLVQG
GGLEALTERFQPQQATPPLMMEQKPGGMYPQPYSSASPTTNLPGAFPGMVRQKPSFGPMPVQVPPPRGAFPTSM
AMQPRQTLNRPPTAPNQLRLQLQQRLQGQQQLIHQNRQAILNQFAASSPVGINMRAGMQQQITPQPPLNAQMLA
QRQRELYSQQHRQRQLMQQRAILMRQQSFGNNLPPSAGLPVQIGAARLPQAPPQQFPYPPNYGTTPGNPPTSTS
PFSPLASNPEAALANRSSMVNRGMMGGVGGQFGAGMTPQMQQNIFQYSGSGMGQQSEPAFAPSLSPSSPLMSPQ
LPPSQSPMLQPAPPTPGYQSPDMKTWQQGAMGNSNVFSQAGQSQPAPAQQGMYNNMSITVSMAGGNTNVQNMNP
MAGQMQMNSLQMPGINSMCSEQVNDPALRPAGLYCNQLSSTDLLKTEADGAQQVQQVQVFADVQCTVNLVGDPY
LNPPAPLGTPKNAAGPPPPPGQQKSLLQQLLTE
>89Ciccaba nigrolineata|A0A7K8VRI6|Aves|SRC1
MSGLGDSSTDPANPDSRKRKGSPCDTAQSNEKRRREQENKYLEELAELLSANIGDIDTLSVKPDKCKILKKTVD
QIQQMKRLEQEKAADDEVQKSDISSSSQGVIEKESLGPLLLEALDGFFFVVNREGRIVFVSENVTSYLGYNQEE
LMNTSVYSILHVGDHAEFVKNLLPKSLVNGVPWPQEATRRNSHTFSCRMLIRPPDEPGAENQEARQRYEVMQCF
TVSQPKSFKEEGEDFQSCLICIARRLPRSAAVTPSESFVTKQDTTGKIISIDTSSLRAAGRTGWEDLVRKCIYA
FFQPQGREPSYAKQLFQEVMTRGTAFSPSYRFTLSDGTVLSAHTKCKLCYPSSPEMQPFIMGIHVIDRDHGMLS
PPENTNSAMSLPRVSPSLNPSISPGQGMALPPSIPPSNGSMLATNVNQQPGASLHNSNSSTSQTSFGCSPGNQI
GANVALSQGQAGPQNSNHTLNLNSSPMNSPGITPPQFMSPRHRVSPGLVPRPRVPSNPFSPPVPTMHSPVGMAN
SGGGGGCGTAATRTFPSAPLNSMQGLTEGPSTPVGYSTPPPALRQLSSQSSPGRLAMQPMKAESKDSKEIASIL
SEMIQSDGGSGDGKPLDSGMLHAGDRLAEGGDSKCSQATSHKLVQLLATTAEQQLRHADADTSCKDSLACAGTA
GTLASGNPPSSTCPSSHSSLTERHKILHRLLQEGSPSDITTLAMEHEKKENAPNAATTPTAQNQLPGTPDIKLE
PDALKKKDVKDHQLLRYLLDKDEKELAAAPALSLDDVKVKVEKTEQMEPCNTAPVPLAKPPATEEVKLETQGQF
AAELEQLDQLLPSLEKAAQLPGLCGPERSESGVAGVAVKPEMLAAPLQPSTAARAPTGLNPMNSGQSVQAGRTP
FEFCDPLEPAQLRPVLCPTSNGSSPHTRGPSQQGRGTVTAPNPFPPDTGMPELEMGVPDHQFGQPGMGEQIPWT
DNSMATMNRGALNKPEDQCITSQLDELLCPPTTPEGRNDEKALLEQLVSFLSGKDETELAELDRALGIDKLVQG
GGLEALTERFQPQQATPPLMMEQKPGMYPQPYSSASPTTNLPGAFPGMVRQKPSFGPMPVQVPPPRGAFPTNMA
MQPRQTLNRPPTAPNQLRLQLQQRLQGQQQLIHQNRQAILNQFAANSPVGINMRAGMQQQITPQPPLNAQMLAQ
RQRELYSQQHRQRQLMQQRAILMRQQSFGNNLPPSAGLPVQIGAARLPQAPPQXTTPGNPPTSTSPFSPLASNP
EAALANRSSMVNRGMMGGVGGQFGAGMTPQMQQNIFQYSGSGMGQQSEPAFAPSLSPSSPLMSPQLPPSQSPML
QPAPPTPGYQSPDMKTWQQGAMGNSNVFSQAGQSQPAPAQQGMYNNMSITVSMAGGNTNVQNMNPMAGQMQMNS
LQMPGMNSMCSEQVNDPALRPTGLYCNQLSSTDLLKTEADGAQQVQQVQVFADVQ
>90Eubucco bourcierii|A0A7K8X9Z8|Aves|SRC1
MSGLGDSSTDPANPDSRKRKGSPCDTAQSNEKRRREQENKYLEELAELLSANIGDIDTLSVKPDKCKILKKTVD
QIQQMKRLEQEKTADDEVQKSDISSSSQGVIEKESLGPLLLEALDGFFFVVNREGRIVFVSENVTSYLGYNQEE
LMNTSVYSILHVGDHAEFVKNLLPKSLVNGVPWPQEATRRNSHTFSCRMLIRPPDEPGAENQEARQRYEVMQCF
TVSQPKSFKEEGEDFQSCLICIARRLPRPAAVTPSESFVTKQDTTGKIISIDTSSLRAAGRTGWEDLVRKCIYA
FFQPQGREPSYAKQLFQEVMTRGTAFSPSYRFTLSDGTVLSAHTKCKLCYPSSPEMQPFIMGIHVIDRDHGMLS
PQENTNSTMSLPRVSPSLNPSISPGQGMALPPSIPPSNGSMLATNVNQQPGAGLHNSNSSTSQTSFGCSPGNQI
GANVALSQGQAGPQNSNHTLNLNSSPMNSPGITPPQFMSPRHRVSPGLVPRPRVPSNPFSPTMPTMHSPVGMAN

SGSGGSCGTGGTRPFPSAPINSMQGLTEGPSTPVGYSTPPPVLRQLSSQSSPGRLAMQPMKAESKDSKEIASIL
SEMIQSDGASGDAKPLDSGMLHTSDRLAEGETKCPQTTSHKLVQLLASTAEQQLRHADVDTSCKDSLACANTAG
PLTSGNPPSSSCPSSHSSLTERHKILHRLLQEGSPSDITTLGMEHEKKESAPNPTPTAQSQLPGTPDMKLEPEA
LKKKDAKDHQLLRYLLDKDEKELAAAPALSLDDVKVKVEKTEQMEPCNTAPVPLPKPPATEEVKLETQGQFAAE
LEQLDQLLPSLEKAAQLPGLCGPERNESGVAGVAVKPEMLAAPLQPSTAARAPAGLNPMSSGQGVQAARAPFEF
CDTLEPAQLRQVPCPGSNGSSPHARGPAQQGRGTVTAPNPFPPDTGMPELEMGVPDHQFGQPGMGEQIPWTDSS
MAAMNRGTLNKPEDQCITSQLDELLCPPTTPEGRNDEKALLEQLVSFLSGKDETELAELDRALGIDKLVQGGGL
EALTERFQPQQSTPPLMMEQKPGMYPQPYSSASPTTNLPGAFPGMVRQKPSFGAMPVQVPPPRGAFPTNMAMQP
RQTLSRPPTAPNQLRLQLQQRLQGQQQLIHQNRQAILNQFAANSPVGINMRAGMQQQITPQPPLNAQMLAQRQR
ELYSQQHRQRQLMQQRAILMRQQSFGSNLPPSSGLPVQIGAARLPQAPPQQFPYPPNYGTSPGNPPTSTSPFSP
LASNPEAALGNRSGMVNRGMMGGVAGQFGAGMAPQMQQNIFPYSGSGMGQQSEPSFAPALSPSSPLMSPQLPPS
QSPMLQPAPPTAGYQSPDMKSWQQGAMGSSNVFNQTGQGQVAPAQQGMYNNMSITVSMAGGNTNVQNMNPMAGP
MQMNSLQMPGMNSMCSEQVNDPALRPAGLYCNQLSSTDLLKTEADGAQQVQQVQVFADVQCTVNLVGDPSLTPP
APLGTPKNAAGPPPPPGQQKSLLQQLLTE
>91Psilopogon haemacephalus|A0A7K9BZ28|Aves|SRC1
MSGLGDSSTDPANPDSRKRKGSPCDTAQSNEKRRREQENKYLEELAELLSANIGDIDTLSVKPDKCKILKKTVD
QIQQMKRLEQEKTADDEVQKSDISSSSQGVIEKESLGPLLLEALDGFFFVVNREGRIVFVSENVTSYLGYNQEE
LMNTSVYSILHVGDHAEFVKNLLPKSLVNGVPWPQEATRRNSHTFSCRMLIRPPDEPGTENQEARQRYEVMQCF
TVSQPKSFKEEGEDFQSCLICIARRLPRPAAVTPSESFVTKQDTTGKIISIDTSSLRAAGRTGWEDLVRKCIYA
FFQPQGREPSYAKQLFQEVMTRGTAFSPSYRFTLSDGTVLSAHTKCKLCYPSSPEMQPFIMGIHVIDRDHGMLS
PQENTNSTMPLPRVSPSLNPSISPGQGMALPPSIPPSNGSMLATNVNQQPGAGLHNNNSSTSQTSFGCSPGNQI
GANVALSQGQAGPQNSNHTLNLNSSPMNSPGITPPQFMSPRHRVSPGLVARPRGPSNPFSPTMPTMHSPVGMAN
SGSGGSCGTGGTRAFPSTPMNSMQGLTEGPSTPVGYSTPPPVLRQLSSQSSPGRLAMQPMKAESKDSKEIASIL
SEMIQSDGGSGDSKPLDSGMLHGGDRLAEGETKCPQTTSHKLVQLLASTAEQQLRHADVDTSCKDSLVCAGTGA
PLASSNPPSGSCPSSHSSLTERHKILHRLLQEGSPSDITTLGMEHEKKESAPNPTPAAQSQLPSTPDMKLEPEA
LKKKDAKDHQLLRYLLDKDEKELAAAPALSLDDVKVKVEKTEQMEPCNTAPVPLPKPPATEEVKLETQGQFAAE
LEQLDQLLPSLEKAAQLPGLCGPERSESGVAGVAVKPEMLAAPLQPSTAARAPAGLNPMSSGQGVQAGRAPFEF
CDTLEQAQLRQVPCPGSNGSSPHARGPAQQGRGTVTAPNPFPPDTGMPELEMGVPDHQFGQPGMGEQIPWTDNS
MAAMNRGTLNKPEDQCITSQLDELLCPPTTPEGRNDEKALLEQLVSFLSGKDETELAELDRALGIDKLVQGGGL
EALTERFQPQQSTPPLMMEQKPGMYPQPYSSASPTANLPGAFPGMVRQKPSFGGMPVQVPPPRGAFPTNMAMQP
RQTLSRPPTAPNQLRLQLQQRLQGQQQLIHQNRQAILNQFAASSPVGINMRAGMQQQITPQPPLNAQMLAQRQR
ELYSQQHRQRQLMQQRAILMRQQSFGSNLPPSSGLPVQIGAARLPQAPPQQFPYPPNYGTSPGNPPTSTSPFSP
LASNPEAALGNRSSMVNRGMMGGVPGQFGAGMAPQMQQNIFPYSGSGMGQQSEPSFAPALSPSSPLMSPQLPPS
QSPMLQPAPPAAGYQSPDMKSWQQGSMASSSVFNQTGQGQVAPAQQGMYNNMSITVSMAGGNTNVQNMNPMAGP
MQMNSLQMPGMNSMCSEQVNDPALRPAGLYCNQLSSTDLLKTEADGAQQVQQVQVFADVQCTVNLVGDPYLNPP
APLGTPKNAAGPPPPPGQQKSLLQQLLTE
>92Hemiprocne comata|A0A7K9CYK3|Aves|SRC1
MSGLGDSSTDPANPDSRKRKGSPCDTAQSNEKRRREQENKYLEELAELLSANIGDIDTLSVKPDKCKILKKTVD
QIQQMKRLEQEKAADDEVQKSDISSSSQGVIEKESLGPLLLEALDGFFFVVNREGRIVFVSENVTSYLGYNQEE
LMNTSVYSILHVGDHAEFVKNLLPKSLVNGVPWPQEATRRNSHTFSCRMLIRPPDEPGAENQEARQRYEVMQCF
TVSQPKSFKEEGEDFQSCLICIARRLPRPAAVTPSESFVTKQDTTGKIISIDTSTLRAAGRTGWEDLVRKCIYA
FFQPQGREPSYAKQLFQEVMTRGTAFSPSYRFTLSDGTVLSAHTKCKLCYPSSPEMQPFIMGIHVIDRDHGMLS
PQENTNSAMSLPRVSPSLNPSISPGQGMALPPSIPPSNSSMLATNVNQQPSAGLHNSNSSTSQTSFGCSPGNQI
GANVALSQGQAGPQNSNHTLNLNSSPMNSPGITPPQFMSPRHRVSPGLVTRPRVPGNPFSPTMPTMHSPVGMAN
SGSGSGTGGTRPFPSASLNSMQGLSEGPSTPVGYSTPPPVLRQISSQNSPGRLAMQPMKAESKDSKEIASILSE
MIQSDGGSGDSKPLESGMLHAGDRLAEGDNKCSQATSHKLVQLLATTAEQQLRHADADTSCKDSLACAGTAGTL
ASSNPPSSTCPSSHSSLTERHKILHRLLQEGSPSDITTLAMEHEKKENAPNPTTTPTAQSQLPGNPDIKLEPDA
LKKKDVKDHQLLRYLLDKDEKELAAAPALSLDDVKVKVEKTEQMEPCNTAPVPLAKPPATEEVKLETQGQFAAE
LEQLDQLLPSLEKAAQLPGLCGPERNEGSVAGVAVKPEMLAAPLQPSAAARAPTGLNPMSSGQAVQAGRTPFEF
CDPLEPGQLRPVPCPTSNGSSPHARGPSQQGRGTVTAPNPFPPDTGMPELEMGVPDHQFGQPGMGEQIPWTDNT
MTAMNRGTLNKPEDQCITSQLDELLCPPTTPEGRNDEKALLEQLVSFLSGKDETELAELDRALGIDKLVQGGGL
EALTERFQPQQATPPLMMEQKPGMYPQPYSSASPTTNLPGAFPGMVRQKPSFGSMPVQVPPPRGAFPTTMAMQP
RQTLNRPPTAPNQLRLQLQQRLQGQQQLLHQNRQAILNQFAASSPVGINMRAGMQQQITPQPPLNAQMLAQRQR
ELYSQQHRQRQLMQQRAILMRQQSFGNNLPPSAGLPVQIGATRLPQAPPQQFPYPPNYGTTPGNPPTSTSPFSP

LTSNPEAALANRSSMVNRGMMGGVGGQFGAGMTPQMQQNIFQYSGSGMGQQSEPAFAPSLSPSSPLMSPQLPPS
QSPMLQPAPPTPGYQSPDMKTWQQGAMGNSNVFSQAGQSQPAPAQQGMYNNMSITVSMAGGNTNVQNMNPMAGQ
MQMNSLQMPGMNSMCSEQVNDPALRPAGLYCNQLSSTDLLKTEADGAQVSKKLLSTPQCTVNLVGDPSLTPPAP
LGTPKNAAGPPPPPGQQKSLLQQLLTE
>93Stercorarius parasiticus|A0A7K9FBS5|Aves|SRC1
MSGLGDSSTDPANPDSRKRKGSPCDTAQSNEKRRREQENKYLEELAELLSANIGDIDTLSVKPDKCKILKKTVD
QIQQMKRLEQEKAADDEVQKSDISSSSQGVIEKESLGPLLLEALDGFFFVVNREGRIVFVSENVTGYLGYNQEE
LMNTSVYSILHVGDHAEFVKNLLPKSLVNGVPWPQEATRRNSHTFSCRMLIRPPDEPGAENQEARQRYEVMQCF
TVSQPKSFKEEGEDFQSCLICIARRLPRPAAVTPSESFVTKQDTTGKIISIDTSSLRAAGRTGWEDLVRKCIYA
FFQPQGREPSYAKQLFQEVMTRGTAFSPSYRFTLSDGTVLSAHTKCKLCYPSSPEMQPFIMGIHVIDRDHGMLS
PQENTNSAMSLPRVSPSLNPSISPGQGMALPPSIPPSNGSMLATNVNQQPGAGLHNSNSSTSQTSFGCSPGNQI
GANVALSQGQAGPQNSNHTLNLSSSPMNSPGITPPQFMSPRHRVSPGLVPRPRVPSNPFSPTMPTMHSPVGMAN
SGSGGGGGTGGARPFPSAPINSMQGLTEGPSTPVGYSTPPPVLRQLSSQSSPGRLAMQPMKTESKDSKEIASIL
SEMIQSDGGSGDGKPLDSGMLHAGDRLSEGDSKCSQATSHKLVQLLATTAEQQLRHADADTSCKDSLACAGTAG
TLASSNPPSSTCPSSHSSLTERHKILHRLLQEGSPSDITTLAMEHEKKENAPNPTTTPTAQSQLPGTPDIKLEP
DALKKKDVKDHQLLRYLLDKDEKELAAAPALSLDDVKVKVEKTEQMEPCNTTPVPLAKPPAAEEVKLETQGQFA
AELEQLDQLLPSLEKAAQMPGLCGPERSESGVAGVTVKPEMLAAPLQPSTAARAPTGLNPMTSGQGVQAGRPPF
EFCDPLEPAQLRPVPCPASNGSSPHAARGPSQQGRGTVTAPNPFPPDTGMPELEMGVPDHQFGQPGMGEQIPWT
DNGMAAMNRGALNKPEDQCITSQLDELLCPPTTPEGRNDEKALLEQLVSFLSGKDETELAELDRALGIDKLVQG
GGLEALTERFQPQQATPPLMMEQKPGMYPQPYSSASPTTNLPGAFPGMVRQKPSFGPMPVQVPPPRGAFPTNMA
MQPRQTLNRPPTAPNQLRLQLQQRLQGQQQLIHQNRQAILNQFAANSPVGINMRAGMQQQITPQPPLNAQMLAQ
RQRELYSQQHRQRQLMQQRAILMRQQSFGNNLPPSAGLPVQIGAARLPQAPPQQFPYPPNYGTTPGNPPTSTSP
FSPLASNPEAALANRSSMVNRGMMGGVGGQFGTGMTPQMQQNIFQYSGSGMGQQSEPAFAPSLSPSSPLMSPQL
PPSQSPMLQPAPPTPGYQSPDMKTWQQGAMGNSNVFSQAGQSQPAPAQQGMYNNMSITVSMAGGNTNVQNMNPM
AGQMQMNSLQMPGMNSMCSEQVNDPALRPAGLYCNQLSSTDLLKTEADGAQQVQQVQVFADVQCTVNLVGDPYL
NPPAPLGTPKNAAGPPPPPGQQKSLLQQLLTE
>94Oceanodroma tethys|A0A7K9MMM5|Aves|SRC1
MSGLGDSSTDPANPDSRKRKGSPCDTAQSNEKRRREQENKYLEELAELLSANIGDIDTLSVKPDKCKILKKTVD
QIQQMKRLEQEKAADDEVQKSDISSSSQGVIEKESLGPLLLEALDGFFFVVNREGRIVFVSENVTSYLGYNQEE
LMNTSVYSILHVGDHAEFVKNLLPKSLVNGVPWPQEATRRNSHTFSCRMLIRPPDEPGAENQEARQRYEVMQCF
TVSQPKSFKEEGEDFQSCLICIARRLPRPAAVTPSESFVTKQDTTGKIISIDTSSLRAAGRTGWEDLVRKCIYA
FFQPQGREPSYAKQLFQEVMTRGTAFSPSYRFTLSDGTVLSAHTKCKLCYPSSPEMQPFIMGIHVIDRDHGMLS
PQENTNSAMSLPRVSPSLNPSISPGQGMALPPSIPPSNGSMLATNVNQQPGAGLHNSNSSTSQTSFGCSPGNQI
GANVALSQGQAGPQNSNHTLNLNSSPMNSPGITPPQFMSPRHRVSPGLVPRPRVPSNPFSPTMPTMHSPVGMAN
SGSGGGSGTGGTRPFPSAPINSMQGLTEGPSTPVGYSTPPPVLRQLSSQSSPGRLAMQPMKAESKDSKEIASIL
SEMIQSDGGSGDGKPLDSGMLHAGDRISEGDGKCSQATSHKLVQLLATTAEQQLRHADADTSCKDSLACAGTAG
TLASGNPPSSTCPSSHSSLTERHKILHRLLQEGSPSDITTLAMEHEKKENAPNPATTPTAQNQLPGTPDIKLEP
DALKKKDVKDHQLLRYLLDKDEKELAAAPALSLDDVKVKVEKTEQMEPCNTAPVPLAKPPATEEVKLETQGQFA
AELEQLDQLLPSLEKAAQLPGLCGPERNESGVAGVTVKPEMLAAPLQASTAARAPTGLNPMNSGQGVQAGRTPF
EFCDPLEPAQLRPVPCPTSNGSSPHARGPSQQGRGTVTAANPFPPDTGMPELEMGVPDHQFGQPGMGEQIPWTD
NSMAAMNRGALNKPEDQCITSQLDELLCPPTTPEGRNDEKALLEQLVSFLSGKDETELAELDRALGIDKLVQGG
GLEALTERFQPQQATPPLMMEQKPGMYPQPYSSASPTANLPGAFPGMVRQKPSFGPMPVQVPPPRGAFPTSMAM
QPRQTLNRPPTAPNQLRLQLQQRLQGQQQLIHQNRQAILNQFAANSPVGINMRAGMQQQITPQPPLNAQMLAQR
QRELYSQQHRQRQLMQQRAILMRQQSFGNNLPPSAGLPVQIGAARLPQAPPQQFPYPPNYGTTPGNPPTSTSPF
SPLASNPEAALANRSSMVNRGMMGSVGGQFGAGMTPQMQQNIFQYSGSGMGQQSEPAFAPSLSPSSPLMSPQLP
PSQSPMLQPAPPTPGYQSPDMKTWQQGAMGNSNVFSQGGQSQPAPAQQGMYNNMSITVSMAGGNTNVQNMNPMA
GQMQMNSLQMPGMNSMCSEQVNDPALRPAGLYCNQLSSTDLLKTEADGAQ
>95Odontophorus gujanensis|A0A7K9YMF7|Aves|SRC1
MSGLGDSSTDPANPDSRKRKGSPCDTAQSNEKRRREQENKYLEELAELLSANIGDIDTLSVKPDKCKILKKTVD
QIQQMKRLEQEKAADDEVQKSDISSSSQGVIEKESLGPLLLEALDGFFFVVNREGRIVFVSENVTSYLGYNQEE
LMNTSVYSILHVGDHTEFVKNLLPKSLVNGVPWPQEATRRNSHTFSCRMLIRPPDEPGTENQEARQRYEVMQCF
TVSQPKSFKEEGEDFQSCLICIARRLPRPAAVTPSESFVTKQDTTGKIISIDTSSLRAAGRTGWEDLVRKCIYA
FFQPQGREPSYAKQLFQEVMTRGTAFSPSYRFTLSDGTVLSAHTKCKLCYPSSPEVQPFIMGIHIIDRDHGMLS
PQENTNSAMALPRVSPSLNPSISPGQGMALPPSIPPSNGSMLATPGNQQPGASLHSSNSSTSQPSFGCSPGNQI

GANVALSQGQAGPPNSNHTLNLNSSPMNSPGITPPQFMSPRHRVSPGLVPRPRGPSNPFSPTMPTMHSPVGMAN
SGGGSNGGGGGSRSFPTAPINSLQGLSEGASTPVGYSTPPPVLRQLSSQSSPGRLGMQPMKAESKDSKEIASIL
SEMIQSDGGAGDGKPLDSGVLHPGERLSEGESKCSQATSHKLVQLLASTAEQQLRHADADTSCKDSLVCAGTTG
TLGAGNPPSSTCPSSHSSLTERHKILHRLLQEGSPSDITTLAMEHDKKDNVPNPATTPTAPSQLPGTPDIKLES
DAMKKKDVKDHQLLRYLLDKDEKELASAPALSLDDVKVKVEKTEQMEPCNTTPVPLAKPPAAEEVKLESQGQFA
AELEQLDQLLPSLEKAAQLPGLCGPERSESGVAVKPEMLAAPLQPSTAPRAPTGLNPISSGQAMQAARAPFEFC
DPLEPAQLRPVAFPATNGSSPRPRAPTEQGRGTVPGPNPFPPDTGMSELEMGVPDHQFGQPAMGEQIPWTDNSM
APMNRGTLSKPEDPCITSQLDELLCPPTTPEGRNDEKALLEQLVSFLSGKDETELAELDRALGIDKLVQGGGLE
ALTERFQPQQATPPLMMEQKPGMYPQPYSSASPTTSLPAAFPGMVRQKPSFGPMPVQVPPPRGAFPTTMAMQPR
QALSRPPTAPNQLRLQLQQRLQGQQQLIHQNRQAILNQFAANSPVGINMRAGMQQQMTPQPPLNAQMLAQRQRE
LYSQQHRQRQLMQQRAILMRQQSFGNNLPPSAGLPVQMGAARLPQAPPQQFPYPPNYGTSPGNPPTSTSPFSQL
ASSPEAALANRGGMVTRGMMGSVGGQFGAGMTPQMQQNIFQYSGSGMGQQNEPAFAPSLSPSSPLMSPQLPPSQ
SPMLQPAPPAPGYQSPDMKTWQQGAMGNSNVFSQTGQTQPAPAQQGMYNNMSITVSMAGGNTNVQNMNPMAGQM
QMNSMQMPGMNSMCSEQVNDPALRPTGLYCNQLSSTDLLKTEADGAQVSKKLLSTSQVQ
>96Spizaetus tyrannus|A0A7L0BY49|Aves|SRC1
MSGLGDSSTDPANPDSRKRKGSPCDTAQSNEKRRREQENKYLEELAELLSANIGDIDTLSVKPDKCKILKKTVD
QIQQMKRLEQEKAADDEVQKSDISSSSQGVIEKESLGPLLLEALDGFFFVVNREGRIVFVSENVTSYLGYNQEE
LMNTSVYSILHVGDHAEFVKNLLPKSLVNGVPWPQEATRRNSHTFSCRMLIRPPDEPGTENQEARQRYEVMQCF
TVSQPKSFKEEGEDFQSCLICIARRLPRPAAVTPSESFVTKQDTTGKIISIDTSSLRAAGRTGWEDLVRKCIYA
FFQPQGREPSYAKQLFQEVMTRGTAFSPSYRFTLSDGTVLSAHTKCKLCYPSSPEMQPFIMGIHVIDRDHGMLS
PQENTNSAMSLPRVSPSLNPSISPGQGMALPPSIPPSNGSMLATNVNQQPGAGLHNSNSSTSQTSFGCSPGNQI
GANVALSQGQAGPQNSNHTLNLNSSPMNSPGITPPQFMSPRHRVSPGLVPRPRVPSNPFSPTMPTMHSPVGMAN
SGSGGSCGTGGTRPFPSAPINSMQGLTEGPSTPVGYSTPPPVLRQLSSQSSPGRLAMQPMKAESKDSKEIASIL
SEMIQSDGGSGDGKPLDSGMLHASDRLSEGDSKCSQATSHKLVQLLATTAEQQLRHADADTSCKDSLVCAGTAG
TLASGNPPSSTCPSSHSSLTERHKILHRLLQEGSPSDITTLAMEHEKKENTPNPTTTPTAQSQLPGTPDIKLEP
DALKKKDVKDHQLLRYLLDKDEKELAAAPALTLDDVKVKVEKTEQMEPCNTAPVPLAKPPATEEVKLETQGQFA
AELEQLDQLLPSLEKAAQLPSLCGPERSESGVTSVAVKPEMLAAPLQPSTAARAPTGLNPMNSGQGVQAGRTPF
EFCDPLEPAQLRPVPCPASNGSSPHTRGPSQQGRGTVTAPNPFPPDTGMPELEMGVPDHQFGQPGMGEQIPWTD
NSMAAMNRGALNKPEDQCITSQLDELLCPPTTPEGRNDEKALLEQLVSFLSGKDETELAELDRALGIDKLVQGG
GLEALTERFQPQQATPPLMMEQKPGMYPQPYSSASPTTNLPGAFPGMVRQKPSFGPMPVQVPPPRGAFPTNMAM
QPRQTLNRPPTAPNQLRLQLQQRLQGQQQLIHQNRQAILNQFAATSPVGINMRAGMQQQITPQPPLNAQMLAQR
QRELYSQQHRQRQLMQQRAILMRQQSFGNNLPPSAGLPVQIGAARLPQAPPQQFPYPPNYGTTPGNPPTSTSPF
SPLASNPEAALANRSSMVNRGMMGGVGGQFGAGMTPQMQQNIFQYSGSGMGQQSEPAFAPSLSPSSPLMSPQLP
PSQSPMLQPAPPTPGYQSPDMKTWQQGAMGNSNVFSQAGQSQPAPAQQGMYNNMSITVSMAGGNTNVQNMNPMA
GQMQMNSLQMPGMNSMCSEQVNDPALRPAGLYCNQLSSTDLLKTEADGAQQVQQVQVFADVQCTVNLVGDPYLN
PPAPLGTPKNAAGPPPPPGQQKSLLQQLLTE
>97Corythaixoides concolor|A0A7L0FKI8|Aves|SRC1
MSGLGDSSTDPANPDSRKRKGSPCDTAQSNEKRRREQENKYLEELAELLSANIGDIDTLSVKPDKCKILKKTVD
QIQQMKRLEQEKAADDEVQKSDISSSSQGVIEKESLGPLLLEALDGFFFVVNREGRIVFVSENVTSYLGYNQEE
LMNTSVYSILHVGDHAEFVKNLLPKSLVNGVPWPQEATRRNSHTFSCRMLIRPPDEPGAENQEARQRYEVMQCF
TVSQPKSFKEEGEDFQSCLICIARRLPRPTAVTPSESFMTKQDTTGKIISIDTSSLRAAGRTGWEDLVRKCIYA
FFQPQGREPSYAKQLFQEVMTRGTAFSPSYRFTLSDGTVLSAHTKCKLCYPSSPEMQPFIMGIHVIDRDHGMLS
PQENTNSAMSLPRVSPSLNPSISPGQGMALPPSIPPSNGSMLATNVNQQPGAGHHNSNSSTSQTSFGCSPGNQI
GANVALSQGQGGPQNSNHALNLNSSPMNSPGITPPQFMSPRHRVSPGLVPRPRVPSNPFSPTMPTMHSPVGMTN
SGSGGGGGTAGTRPFPSAPINSMQGLTEGPSTPVGYSTPPPVLRQLSSQSSPGRLAMQPMKAESKDSKEIASIL
SEMIQSDSGSGDSKPLDSGVLHAGDRLSEGDSKCSQATSHKLVQLLATTAEQQLRHADADTSCKDSLACAGTAG
TLASSNPPSSTCPSSHSSLTERHKILHRLLQEGSPSDITTLGMEHEKKENPPNPTTTPTAQSQLPGAPDIKLEQ
EALKKKDVKDHQLLRYLLDKDEKELAAAPALSLDDVKVKVEKTEQMEPCNTAPVPLAKPPATEEVKLETQGQFA
AELEQLDQLLPSLDKAAQLPGLCPPERSESGVSGVAVKPEMLAAPLQPSAAARAPAGLNPMSSGQGVQAGRTPF
EFCDPLEPAQLRPVPCPTSNGSSPHARGPSQQGRGTVTAPNPFPPDTGMPELEMGVPDHQFGQPGMGEQIPWTD
NSMAAMNRGALNKPEDQCITSQLDELLCPPTTPEGRNDEKALLEQLVSFLSGKDETELAELDRALGIDKLVQGG
GLEALTERFQPQQATPPLMMEQKGGMYPQPYSSASPTASLPGAFPGMVRQKPSFGAMPVQVPPPRGAFPTTMAM
QPRQPLARPPTAPNQLRLQLQQRLQGQQQLIHQNRQAILNQFAASSPVGINMRAGMQQQITPQPPLNAQMLAQR
QRELYSQQHRQRQLMQQRAILMRQQSFGNNLPPSAGLPVQIGAARLPQAPPQQFPYPPNYGTSPGNPPTSTSPF

SPLASNPEATLANRSSMVNRGMMGGVGGQFGAGMTPQMQQNIFQYSGSGMGQQSEPTFAPSLSPSSPLMSPQLP
PSQSPMLQPAPPTPGYQSPDMKTWQQGAMGNSNVFSQAGQSQPAPAQQGMYNNMSITVSMAGGNTNVQNMNPMA
GQMQMNSLQMPGMNSMCSEQVNEPALRPAGLYCNQLSSTDLLKTEADGAQ
>98Herpetotheres cachinnans|A0A7L0GR87|Aves|SRC1
MSGLGDSSTDPANPDSRKRKGSPCDTAQSNEKRRREQENKYLEELAELLSANIGDIDTLSVKPDKCKILKKTVD
QIQQMKRLEQEKAADDEVQKSDISSSSQGVIEKESLGPLLLEALDGFFFVVNREGRIVFVSENVTGYLGYNQEE
LMNTSVYSILHVGDHAEFVKNLLPKSLVNGVPWPQEATRRNSHTFSCRMLIRPPDEPGAENQEARQRYEVMQCF
TVSQPKSFKEEGEDFQSCLICIARRLPRPAAVTPSESFVTKQDTTGKIISIDTSSLRAAGRTGWEDLVRKCIYA
FFQPQGREPSYARQLFQEVMTRGTAFSPSYRFTLSDGTVLSAHTKCKLCYPSSPEMQPFIMGIHVIDRDHGMLS
PQENTNSAMPLPRVSPSLNPSISPGQGMALPPSIPPSNGSMLATNVNQQPGASLHNSNSSTSQTSFGCSPGNQI
GANVALSQGQAGPQNSNHTLNLNSSPMNSPGITPPQFMSPRHRVSPGLVPRPRVPSNPFSPTMPTMHSPVGMAN
SGSGGGCGTGATRPFPSAPINSMQGLTEGPSTPVGYSTPPPVLRQLSSQSSPSRLAMQPMKAESKDSKEIASIL
SEMIQSDGSSGDGKPLDSGMLHASDRLSEGDSKCSQATSHKLVQLLATTAEQQLRHADADTSCKDSLACAGTTG
TLASGNPPSSTCPSSHSSLTERHKILHRLLQEGSPSDITTLAMEHEKKETTPNPATTPTAQSQLLGTPDIKLEP
DTLKKKDVKDHQLLRYLLDKDEKELAAAPALSLDDVKVKVEKTEQMEPCNTAPVPLAKPPAAEEVKLETQGQFA
AELEQLDQLLPSLEKAAQLPSLCGPERTESGVASVAVKPEMLAAPLQPSTAARAPTGLNPMNSGQSGQAGRTPF
EFCDPLEPAQLRPVPCPASNGSSPHTRGPSQQGRGTVTAPNPFPPDTGMPELEMGVPDHQFGQPGMGEQIPWTD
NSMAAMNRGALNKPEDQCITSQLDELLCPPTTPEGRNDEKALLEQLVSFLSGKDETELAELDRALGIDKLVQGG
GLEALTERFQPQQATPPLMMEQKPGMYPQPYSSASPTTNLPGAFPGMVRQKPSFGPMPVQVPPPRGAFPTNMAM
QPRQTLNRPPTAPNQLRLQLQQRLQGQQQLIHQNRQAILNQFAANSPVGINMRAGMQQQITPQPPLNAQMLAQR
QRELYSQQHRQRQLMQQRAILMRQQSFGNNLPPSAGLPVQIGAARLPQAPPQQFPYPPNYGTTPGNPPTSTSPF
SPLASNPEAALANRSSMVNRGMMGSVGGQFGAGMTPQMQQNIFQYSGSGMGQQNEPAFAPSLSPSSPLMSPQLP
PSQSPMLQPAPPTPGYQSPDMKTWQQGAMGNSNVFSQAGQSQPAPAQQGMYNNMSITVSMAGGNTNVQNMNPMA
GQMQMNSLQMPGMNSMCSEQVNDPALRPAGLYCNQLSSTDLLKTEADGAQQVQQVQVFADVQCTVNLVGDPYLN
PPAPLGTPKNAAGPPPPPGQQKSLLQQLLTE
>99Mesembrinibis cayennensis|A0A7L0P1N9|Aves|SRC1
MSGLGDSSTDPANPDSRKRKGSPCDTAQSNEKRRREQENKYLEELAELLSANIGDIDTLSVKPDKCKILKKTVD
QIQQMKRLEQEKAADDEVQKSDISSSSQGVIEKESLGPLLLEALDGFFFVVNREGRIVFVSENVTSYLGYNQEE
LMNTSVYSILHVGDHAEFVKNLLPKSLVNGVPWPQETTRRNSHTFSCRMLIRPPDEPGTENQEARQRYEVMQCF
TVSQPKSFKEEGEDFQSCLICIARRLPRPAAVTPSESFVTKQDTTGKIISIDTSSLRAAGRTGWEDLVRKCIYA
FFQPQGREPSYARQLFQEVMTRGTAFSPSYRFTLSDGTVLSAHTKCKLCYPSSPEMQPFIMGIHVIDRDHGMLS
PQENTNSAMSLPRVSPSLNPSISPGQGMALPPSIPPSNGSMLATNVNQQPGAGLHNNNSSTSQTSFGCSPGNQI
GANVALSQGQAGPQNSNHTLNLNSSPMNSPGITPPQFMSPRHRVSPGLVPRPRVPSNPFSPTMPTMHSPVGMAN
SGSGGGGGTGGTRPFPNAPINSMQGLTEGPSTPVGYSTPPPVLRQLSSQSSPGRLAMQPMKAESKDSKEIASIL
SEMIQSDGGSGDGKPLDSGMLHAGDRLSEGDSKCSQATSHKLVQLLATTAEQQLRHADADTSCKDSLACAGTAG
SLASSNPPSSTCPSSHSSLTERHKILHRLLQEGSPSDITTLAMEHEKKENTPNPATTPTAQSQLPGTPDIKLEP
DVLKKKDVKDHQLLRYLLDKDEKELAAAPALSLDDVKVKVEKTEQMEPCNTAPVPLAKPPAAEEEAKLETQGQFA
AELEQLDQLLPSLEKAAQLPGLCGPERNESGVAGVTVKPEMLAAPLQPSTAARAPTGLNPMNSGQGVQAGRTPF
EFCDPLEPAQMRPVPCPASNGSSPHARGPSQQGRGTVTAPNPFPPDAGMPELEMGVPDHQFGQPGMGEQIPWTD
NSMSAMNRGALNKPEDQCITSQLDELLCPPTTPEGRNDEKALLEQLVSFLSGKDETELAELDRALGIDKLVQGG
GLEALTERFQPQQATPPLMMEQKPGMYPQPYSSASPTTNLPGAFPGMVRQKPSFGPMPVQVPPPRGAFPTSMAM
QPRQTLNRPPTAPNQLRLQLQQRLQGQQQLIHQNRQAILNQFAASSPVGINVRAGMQQQITPQPPLNAQMLAQR
QRELYSQQHRQRQLMQQRAILMRQQSFGNNLPPSAGLPVQIGAARLPQAPPQQFPYPPNYGTTPGNPPTSTSPF
SPLASNPEAALANRSSMVNRGMMGGVGGQFGAGMTPQMQQNIFQYSGSGMGQQSEPAFAPSLSPSSPLMSPQLP
PSQSPMLQPAPPTPGYQSPDMKTWQQGAMGNSNVFSQAGQSQPAPAQQGMYNNMSITVSMAGGNTNVQNMNPMA
GQMQMNSLQMPGMNSMCSEQVNDPALRPTGLYCNQLSSTDLLKTEADGAQQVQQVQVFADVQCTVNLVGDPYLN
PPAPLGTPKNAAGPPPPPG
>100Glaucidium brasilianum|A0A7L0RXR6|Aves|SRC1
MSGLGDSSTDPANPDSRKRKGSPCDTAQSNEKRRREQENKYLEELAELLSANIGDIDTLSVKPDKCKILKKTVD
QIQQMKRLEQEKAADDEVQKSDISSSSQGVIEKESLGPLLLEALDGFFFVVNREGRIVFVSENVTSYLGYNQEE
LMNTSVYSILHVGDHAEFVKNLLPKSLVNGVPWPQEATRRNSHTFSCRMLIRPPDEPGAENQEARQRYEVMQCF
TVSQPKSFKEEGEDFQSCLICIARRLPRSAAVTPSESFVTKQDTTGKIISIDTSSLRAAGRTGWEDLVRKCIYA
FFQPQGREPSYAKQLFQEVMTRGTAFSPSYRFTLSDGTVLSAHTKCKLCYPSSPEMQPFIMGIHVIDRDHGMLS
PPENTNSAMSLPRVSPSLNPSISPGQGMALPPSIPPSNGSMLATNVNQQPGAGLHNSNSSTSQTSFGCSPGNQI

GANVALSQGQAGPQNSNHTLNLNSSPMNSPGITPPQFMSPRHRVSPGLVPRPRVPSNPFSPSVPTMHSPVGMAN
SGSGGGCGTAATRSFPSAPLNSMQGLTEGPSTPVGYSTPPPALRQLSSQSSPGRLAMQPMKAESKDSKEIASIL
SEMIQSDSGSGDGKPLDSGMLHAGDRLTEGGDSKCSQATSHKLVQLLATTAEQQLRHADADTSCKDSLACAGTT
GTLASGNPPSSTCPSSHSSLTERHKILHRLLQEGSPSDITTLAMEHEKKENTPNAATTPTAQNQLPGTPDIKLE
PDALKKKDVKDHQLLRYLLDKDEKELAAAPALSLDDVKVKVEKTEQMEPCNTAPVPLAKPPAAEEVKLETQGQF
AAELEQLDQLLPSLEKAAQLPGLCGPERSESGVAGVTVKPEMLAAPLQPSTAARAPTGLNPMNSGQSVQAGRAP
FEFCDPLEPTQLRPVPCPASNGSSPHTRGPAQQGRGTVPAPNPFPPDTGMPELEMGVPDHQFGQPGMGEQIPWT
DNSMATMNRGTLNKPEDQCITSQLDELLCPPTTPEGRNDEKALLEQLVSFLSGKDETELAELDRALGIDKLVQG
GGLEALTERFQPQQATPPLMMEQKPGMYPQPYSSASPTTNLPGAFPGMVRQKPSFGPMPVQVPPPRGAFPTNMA
MQPRQTLNRPPTAPNQLRLQLQQRLQGQQQLIHQNRQAILNQFAANSPVGINMRAGMQQQITPQPPLNAQMLAQ
RQRELYSQQHRQRQLMQQRAILMRQQSFGNNLPPSAGLPVQIGAARLPQAPPQQFPYPPNYGTTPGNPPTSTSP
FSPLASNPEAALANRSSMVNRGMMGGVGGQFGAGMTPQMQQNIFQYSGSGMGQQSEPAFAPSLSPSSPLMSPQL
PPSQSPMLQPAPPTPGYQSPDMKTWQQGAMGNSNVFSQAGQSQPAPAQQGMYNNMSITVSMAGGNTNVQNMNPM
AGQMQMNSLQMPGMNSMCSEQVNDPALRPAGLYCNQLSSTDLLKTEADGAQQVQQVQVFADVQCTVNLVGDPYL
NPPAPLGTPKNAAGPPPPPGQQKSLLQQLLTE
>101Indicator maculatus|A0A7L1GF91|Aves|SRC1
MSGLGDSSTDPANPDSRKRKGSPCDTAQSNEKRRREQENKYLEELAELLSANIGDIDTLSVKPDKCKILKKTVD
QIQQMKRLEQEKTADDEVQKSDISSSSQGVIEKESLGPLLLEALDGFFFVVNREGRIVFVSENVTSYLGYNQEE
LMNTSVYSILHVGDHAEFVKNLLPKSLVNGVPWPQEATRRNSHTFSCRMLIRPPDEPGAENQEARQRYEVMQCF
TVSQPKSFKEEGEDFQSCLICIARRLPRPAAVTPSESFVTKQDTTGKIISIDTSSLRAAGRTGWEDLVRKCIYA
FFQPQGREPSYAKQLFQEVMTRGTAFSPSYRFTLSDGTVLSAHTKCKLCYPSSPEMQPFIMGIHVIDRDHGMLS
PQENTNSTMSLPRVSPSLNPSISPGQGMTLPPSIPPSNGSMLATNVNQQPGASLHNSNSSTSQTSFGCSPGNQI
GANVALSQGQAGPQNSNHTLNLNSSPMNSPGITPPQFMSPRHRVSPGLVPRPRVPSNPFSPTMPTMHSPVGMAN
SGSGGSCGTGGTRPFPNTPMNSMQGLPEGPSTPVGYSTPPPVLRQLSSQSSPGRLAMQPMKAESKDSKEIASIL
SEMIQSDSGSGDSKPLDSGMLHPSDRLVEGDSKCPQTTSHKLVQLLATTAEQQLRHADVDTSCKDSLACASTAG
TLGSGNPPSSSCPSSHSSLTERHKILHRLLQEGSPSDITTLGMEHEKKESAPNPTPTAPSQLPGTPDVKLEPDA
LKKKDAKDHQLLRYLLDKDEKELAAAPALSLDDVKVKVEKTEQMEPCNTAPVPLAKPPATEEVKLETQGQFAAE
LEQLDQLLPSLEKAAQLPSLCGPERNESGVAGVAVKPEMLAAPLQPSTAARAPTGLNPMSSGQGVQAGRAPFEF
CDTLEPAQLRQVPCPASNGSSPHARGQAQQGRGTVTAPNPFPPDTGMPELEMGVPDHQFGQPGMGEQIPWTDNS
MAAMNRGTMNKPEDQCITSQLDELLCPPTTPEGRNDEKALLEQLVSFLSGKDETELAELDRALGIDKLVQGGGL
EALTERFQPQQATPPLMMEQKPGMYPQPYSSASPTTNLPGAFPGMVRQKPSFGPMPVQVPPPRGAFPTNMAMQP
RQTLNRPPTAPNQLRLQLQQRLQGQQQLIHQNRQAILNQFAASSPVGINMRAGMQQQITPQPPLNAQMLAQRQR
ELYSQQHRQRQLMQQRALLMRQQSFGSNLPPSTGLPVQIGAARLPQAPPQQFPYPPNYGTSPGNPPTSTSPFSP
LASNPEAALGNRSSMVNRGMMGGVAGQFGAGMAPQMQQNIFQYSGSGMGQQSEPAFAPSLSPSSPLMSPQLPPS
QSPMLQPAPPTAGYQSPDMKTWQQGAMANSNVFSQAGQSQVAPAQQGMYNNMSITVSMAGGNTNVQNMNPMAGQ
MQMNSLQMPGMNSMCSEQVNDPALRPAGLYCNQLSSTDLLKTEADGAQQVQQVQVFADVQCTVNLVGDPYLNPP
APLGTPKNA
>102Indicator maculatus|A0A7L1GTR3|Aves|SRC1
CMVKMSGLGENSLDSLTNESRKRKALPCDTPGASLTCSGEKRRREQESKYIEELAELISANLSDIDNFNVKPDK
CAILKETVRQIRQIKEQGKAVSNDDDVQKADVSSTGQGVIDKDSLGPLLLQALDGFLFVVNRDGNIVFVSENVT
QYLQYKQEDLVNTSVYGILHEEDRKDFLKNLPKSSVNGVAWSNETPRQKSHTFNCRMMVKTSHELLEDVGSHQD
IRQRYETMQCFALSQPRAMMEEGEDLQSCMICVARRITTAERAFPTNPESFITRHDLSGKLVNIDTNSLRSSMR
PGFEDIIRRCIQRFLCHNDGQSWSNKRHYHEAYTQGHAETPLYRFSLADGTIVTAQTKSKLFRNPVTNDRHGFV
STHFLQREQNGYRPNPNSVGQNIRTPVAGNTNSVGGLMNMSPGQGMPIQNRNYGMGDPSAVGQLGSTRYGGPGN
MAPVNSGPGMQSSAYQNNSYGLSISSPPHGSPGLSSNQQTLMISPRNRGSPKMNSHQFSPVTGMHSPMGSASNT
GNSTFSSSSLSALQAISEGVGTSLLSTLSSPGPKLDNSPNMSMGQQSKTSTQDSKSPSGLYCEQNQVESSICQS
NSRDLLNEKDSKEGNLEASESQRGPSESKGHKKLLQLLTCSSDERGHSTASNSPLDSNCKESSTSVTSPSGVSS
STSGGVSSSSNMHGSLLQEKHRILHKLLQNGNSPAEVAKITAEATGKDTYHDASNPAPCGEGPVKQEQLSPKKK
ENNALLRYLLDKDDVKDPLSKELKPKVENVDTKMGQCTSSTIPTSSHEKGMKIKTEPAEEMSGDLDNLDAILGD
LTSSEFYNNAMSANGNNLGTKQALFQGNALLAGMRSPQAMQTTRPPFNRAVSLDSPMGSSAPARSVNTFSMLQK
QNLMGGSPRMMENQENFGANLANAPNRNVAMNQHQSADWGLSNSKVNRLEQTNSSSMLRPGAEFSASLPRPTVG
GSMPGLPVRSNSLPGTRPMLQQQMIHMRPNEINIGMGGNPYGQPGPSTQPGSWPDSMLSMEQASRGSQTRQLVR
NSLDDLLCPAPSMEGQNDERALLDQLHTLLSNTDVTSLEEIDRALGIPDLVNQGQALEPKQDSFQGQESSVMID
QKASLYGQPYQGQGAAMPGGFSNIQGQQPSFNSVMNQMSQQSSFPLQSMHPRANVMRPRTNTPKQLRMQLQQRL

QGQQFMNQTRQTLEMKMENPVSGNTSVMRPVMQPQVGSQQQGFLNAQMVAQRNRELISHHFRQQRMAMMMQQQQ
QPQAFSPPPNVTASGSMDSALTGPPMAQVPPQQFPYPPNYGMSQQPDPAFSRVASPPNPMMSSRIGPSQNPMIQ
HPQTATMYQSPEMKGWPSGSMPRSSSFPQQQFSHQGNPAAYSMMHMNGSSGHIGQMNINTMPMSGMSMAPDQVS
C
>103Smutsornis africanus|A0A7L1JB03|Aves|SRC1
MSGLGDSSTDPANPDSRKRKGSPCDTAQSNEKRRREQENKYLEELAELLSANIGDIDTLSVKPDKCKILKKTVD
QIQQMKRLEQEKAADDEVQKSDISSSSQGVIEKESLGPLLLEALDGFFFVVNREGRIVFVSENVTGYLGYNQEE
LMNTSVYSILHVGDHAEFVKNLLPKSLVNGVPWPQEATRRNSHTFSCRMLIRPPDEPGAENQEARQRYEVMQCF
TVSQPKSFKEEGEDFQSCLICIARRLPRPAAVTPSESFVTKQDTTGKIISIDTSSLRAAGRTGWEDLVRKCIYA
FFQPQGREPSYAKQLFQEVMTRGTAFSPSYRFTLSDGTVLSAHTKCKLCYPSSPEMQPFIMGIHVIDRDHGMLS
PQENTNSTMSLPRVSPSLNPSISPGQGMALPPSIPPSNGSMLATNVNQQPGAGLHNSNSSTSQTSFGCSPGNQI
GANVALSQGQAGPQNSNHTLNLNSSPMNSPGITPPQFMSPRHRVSPGLVPRPRVPSNPFSPTMPTMHSPVGMAN
SGSGGGGGTGGARPFPSAPINSMQALTEGPSTPVGYSTPPPVLRQLSSQSSPGRLAMQPMKAESKDSKEIASIL
SEMIQSDGGSGDGKPLDSGMLHAGDRLSEGDSKCSQATSHKLVQLLATTAEQQLRHADADTSCKDSLACAGTAG
TLASSNPPSSTCPSSHSSLTERHKILHRLLQEGSPSDITTLAMEHEKKENAPNPTTTPTAQSQLPGTADIKLEP
DALKKKDVKDHQLLRYLLDKDEKELAAAPALSLDDVKVKVEKTEQMEPCNTAPVPLAKPPAVEEVKLETQGQFA
AELEQLDQLLPSLEKAAQMPGLCGAERSESGVAGVTVKPEMLAAPLQPSPAARAPTGLNPMTSGQGVQAGRPPF
EFCDPLEPAQLRPVPCPASNGSSPHARGPSQQGRGTVTAPNPFPPDTGMPELEMGVPDHQFGQPGMGEQIPWTD
NSMAAMNRGALNKPEDQCITSQLDELLCPPTTPEGRNDEKALLEQLVSFLSGKDETELAELDRALGIDKLVQGG
GLEALTERFQPQQATPPLMMEQKPGMYPQPYSSASPTTSLPGAFPGMVRQKPSFGPMPVQVPPPRGAFPTNMAM
QPRQTLNRPPTAPNQLRLQLQQRLQGQQQLIHQNRQAILNQFAASSPVGINMRAGMQQQITPQPPLNAQMLAQR
QRELYSQQHRQRQLMQQRAILMRQQSFGNNLPPSAGLPVQIGAARLPQAPPQQFPYPPNYGTTPGNPPTSTSPF
SPLTSNPEAALANRSSMVNRGMMGGVGGQFGAGMTPQMQQNIFQYSGSGMGQQSEPAFAPSLSPSSPLMSPQLP
PSQSPMLQPAPPTPGYQSPDMKTWQQGAMGNSSVFSQAGQSQPAPAQQGMYNNMSITVSMAGGNTNVQNMNPMA
GQMQMNSLQMPGMNSMCSEQVNEPALRPAGLYCNQLSSTDLLKTEADGAQQVQQVQVFADVQCTVNLVGDPYLN
PPAPLGTPKNAAGPPPPPGQQ

>104Rynchops niger|A0A7L1JWB3|Aves|SRC1
MSGLGDSSTDPANPDSRKRKGSPCDTAQSNEKRRREQENKYLEELAELLSANIGDIDTLSVKPDKCKILKKTVD
QIQQMKRLEQEKAADDEVQKSDISSSSQGVIEKESLGPLLLEALDGFFFVVNREGRIVFVSENVTGYLGYNQEE
LMNTSVYSILHVGDHAEFVKNLLPKSLVNGVPWPQEATRRNSHTFSCRMLIRPPDEPGTENQEARQRYEVMQCF
TVSQPKSFKEEGEDFQSCLICIARRLPRPAAVTPSESFVTKQDTTGKIISIDTSSLRAAGRTGWEDLVRKCIYA
FFQPQGREPSYAKQLFQEVMTRGTAFSPSYRFTLSDGTVLSAHTKCKLCYPSSPEMQPFIMGIHVIDRDHGMLS
PQENTNSAMSLPRVSPSLNPSISPGQGMALPPSIPPSNGSMLATNVNQQPGAGLHNSNSSTSQTSFGCSPGNQI
GANVALSQGQAGPQNSNHTLNLNSSPMNSPGITPPQFMSPRHRVSPGLVPRPRVPSNPFSPTMPTMHSPVGMAN
SGSGGSGGTGGARPFPSTPINSMQGLTEGPSTPVGYSTPPPVLRQLSSQSSPGRLAMQPMKAESKDSKEIASIL
SEMIQSDGGSGDGKPLDSGMLHAGDRLSEGDSKCSQATSHKLVQLLATTAEQQLRHADADTSCKDSLACAGTAG
TLASSNPPSSTCPSSHSSLTERHKILHRLLQEGSPSDITTLAMEHEKKENAPNPATTPTAQSQLPGTPDIKLEP
DALKKKDVKDHQLLRYLLDKDEKELAAAPALSLDDVKVKVEKTEQMEPCNTAPVPLAKPPVAEEVKLETQGQFA
AELEQLDQLLPSLEKAAQMPGLCGPERSESGVAGVTVKPEMLAAPLQPSTAARAPTGLNPMTSGQGVQAGRPPF
EFCDPLEPAQLRPVPCPASNGSSPHARGPSQQGRGTVTAPNPFPPDAGMPELEMGVPDHQFGQPGMGEQIPWTD
NSMAAMNRGALNKPEDQCITSQLDELLCPPTTPEGRNDEKALLEQLVSFLSGKDETELAELDRALGIDKLVQGG
GLEALTERFQPQQATPPLMMDQKPGMYPQPYSSASPTANLPGAFPGMVRQKPSFGPMPVQVPPPRGAFPTNMAM
QPRQTLNRPPTAPNQLRLQLQQRLQGQQQLIHQNRQAILNQFAANSPVGINMRAGMQQQITPQPPLNAQMLAQR
QRELYSQQHRQRQLMQQRAILMRQQSFGNNLPPSAGLPVQIGAARLPQAPPQQFPYPPNYGTTPGNPPTSTSPF
SPLASNPEAALANRSSMVNRGMMGGVGGQFGAGMTPQMQQNIFQYSGSGMGQQSEPAFAPSLSPSSPLMSPQLP
PSQSPMLQPAPPTPGYQSPDMKTWQQGAMGNSNVFSQAGQSQPAPAQQGMYNNMSITVSMAGGNTNVQNMNPMA
GQMQMNSLQMPGMNSMCSEQVNDPALRPAGLYCNQLSSTDLLKTEADGAQQVQQVQVFADVQCTVNLVGDPYLN
PPAPLGTPKNAAGP
>105Himantopus himantopus|A0A7L1LE48|Aves|SRC1
MSGLGDSSTDPANPDSRKRKGSPCDTAQSNEKRRREQENKYLEELAELLSANIGDIDTLSVKPDKCKILKKTVD
QIQQMKRLEQEKAADDEVQKSDISSSSQGVIEKESLGPLLLEALDGFFFVVNREGRIVFVSENVTSYLGYNQEE
LMNTSVYSILHVGDHAEFVKNLLPKSLVNGVPWPQEATRRNSHTFSCRMLIRPPDEPGAENQEARQRYEVMQCF
TVSQPKSFKEEGEDFQSCLICIARRLPRPAAVTPSESFVTKQDTTGKIISIDTSSLRAAGRTGWEDLVRKCIYA

FFQPQGREPSYAKQLFQEVMTRGTAFSPSYRFTLSDGTVLSAHTKCKLCYPSSPEMQPFIMGIHVIDRDHGMLS
PQENTNSAMSLPRVSPSLNPSISPGQGMALPPSIPPSNGSMLATNVNQQPGAGLHNSNSSTSQTSFGCSPGNQI
GANVALSQGQAGPQNSNHTLNLNSSPMNSPGITPPQFMSPRHRVSPGLVPRPRVPSNPFSPTMPTMHSPVGMAN
SGSGGGGGTGGTRPFPSAPINSMQGLTEGPSTPVGYSTPPPVLRQLSSQSSPGRLAMQPMKAESKDSKEIASIL
SEMIQSDGGSGDGKPLDSGMLHAGDRLSEGDSKCSQATSHKLVQLLATTAEQQLRHADADTSCKDSLACAGTAG
TLASSNPPSSTCPSSHSSLTERHKILHRLLQEGSPSDITTLAMEHEKKENAPNPATTPTAQSQLPGTPDIKLEP
DALKKKDVKDHQLLRYLLDKDEKELAAAPALSLDDVKVKVEKTEQMEPCNTAPVPLAKPPAAEEVKLETQGQFA
AELEQLDQLLPSLEKAAQLPGLCGPERSESGVAGVTVKPEMLAPPLQPSTAARAPTGLNPMNSGQGVQAGRTPF
EFCDPLEPAQLRPVPCPASNGSSPHARGPSQQGRGTVTAPNPFPPDTGMPELEMGVPDHQFGQPGMGEQIPWTD
NSMAAMNRGALNKPEDQCITSQLDELLCPPTTPEGRNDEKALLEQLVSFLSGKDETELAELDRALGIDKLVQGG
GLEALTERFQPQQATPPLMMEQKPGMYPQPYSSASPTTNLPGAFPGMVRQKPSFGPMPVQVPPPRGAFPTNMAM
QPRQTLNRPPTAPNQLRLQLQQRLQGQQQLIHQNRQAILNQFAASSPVGINMRAGMQQQITPQPPLNAQMLAQR
QRELYSQQHRQRQLMQQRAILMRQQSFGNNLPPSAGLPVQIGAARLPQAPPQQFPYPPNYGTTPGNPPTSTSPF
SPLASNPEAALANRSSMVNRGMMGGVGGQFGAGMTPQMQQNIFQYSGSGMGQQNEPAFAPSLSPSSPLMSPQLP
PSQSPMLQPAPPTPGYQSPDMKTWQQGAMGNSNVFSQAGQSQPAPAQQGMYNNMSITVSMAGGNTNVQNMNPMA
GQMQMNSLQMPGMNSMCSEQVNDPALRPAGLYCNQLSSTDLLKTEADGAQQVQQVQVFADVQCTVNLVGDPYLN
PPAPLGTPKNAA
>106Rhinopomastus cyanomelas|A0A7L1NJY1|Aves|SRC1
MSGLGDSSTDPANPDSRKRKGSPCDTAQSNEKRRREQENKYLEELAELLSANIGDIDTLSVKPDKCKILKKTVD
QIQQMKRLEQEKAADDEVQKSDISSSSQGVIEKESLGPLLLEALDGFFFVVNREGRIVFVSENVTSYLGYNQEE
LMNTSVYSILHVGDHAEFVKNLLPKSLVNGVPWPQEATRRNSHTFSCRMLIRPPDEPGTENQEARQRYEVMQCF
TVSQPKSFKEEGEDFQSCLICIARRLPRPAAVTPSESFVTKQDTTGKIISIDTSSLRAAGRTGWEDLVRKCIYA
FFQPQGREPSYAKQLFQEVMTRGTAFSPSYRFTLSDGTVLSAHTKCKLCYPSSPEMQPFIMGIHVIDRDHGMLS
PQENTNSTMPLPRVSPSLNPSISPGQGMTLPPSIPPSNGSMLASNVNQQPGAGLHNSNSSTSQTNFGCSPGNQI
GANVALNQGQAGPQNSNHTLNLNSSPMNSPGITPPQFMSPRHRVSPGLVPRPRGPSNPFSPTMPTMHSPVGMAN
NGSGGSCGSVGTRPFPSAPINSMQGLTEGPSTPVGYSTPPPVLRQLSSQSSPGRLAMQPMKAESKDSKEIASIL
SEMIQSDGASGDSKPLDSAMLHSSDRLAEGDTKCPQTTSHKLVQLLATTAEQQLRHVDADTSCKDSLACAGTAG
TLASSNPPSSTCPSSHSSLTERHKILHRLLQEGSPSDITTLGMDHEKKESAPNAAATPAAQSQLPGTPDIKLEP
DALKKKDVKDHQLLRYLLDKDEKELAAAPALSLDDVKVKVEKTEQMEPCNTAPVPLAKPPAPEEVKLETQGQFA
AELEQLDQLLPSLEKAAQLPGLCGPDRNESAVAGVAVKAEMLAAPMQPGAATRAPAGLNPMSSGQGVQAGRASF
EFCDPLEPGQLRPGPCPTSNGSSPHARGPSQQGRGAVPAPNPFPPDAAMPELEMAVPEHQFGQPGMGEQMPWAD
GGVAAMARGAIGKPEDQCITSQLDELLCPPTTPEGRNDEKALLEQLVSFLSGKDETELAELDRALGIDKLVQGG
GLEALTERFQPQQATPPLMMEQKPGMYPQPYSSASPTASLPGAFPGMVRQKPSFGPMPVQVPPPRGAFPTNMAM
QPRQTLSRPPTAPNQLRLQLQQRLQGQQQLLHQNRQAILNQFAASSPVGINMRAGMQQQITPQPPLNAQMLAQR
QRELYSQQHRQRQLMQQRAILMRQQSFGNNLPPSTGLPVQIGAARLPQAPPQQFPYPPNYGTSPGNPPTSTSPF
SPLASNPEAALANRSSMVNRGMMGGVGGQFGAGMTPQMQQNIFQYSGSGMGQQSEPAFAPSLSPSSPLMSPQLP
PSQSPMLQPAPPTPGYQSPDMKTWQQGAMGNSNVFSQAGQSQPAPAQQGMYNNMSITVSMAGGNTNVQNMNPMA
PQMQMNSLQMPGMNSMCSEQVNDPALRPTGLYCNQLSSTDLLKTEADGAQVQQVQVFADVQCTVNLVGDPYLNP
PAALGTPKNAAGPPPPPGQQKSLLQQLLTE
>107Sagittarius serpentarius|A0A7L2HEB4|Aves|SRC1
MSGLGDSSTDPANPDSRKRKGSPCDTAQSNEKRRREQENKYLEELAELLSANIGDIDTLSVKPDKCKILKKTVD
QIQQMKRLEQEKAADDEVQKSDISSSSQGVIEKESLGPLLLEALDGFFFVVNREGRIVFVSENVTSYLGYNQEE
LMNTSVYSILHVGDHAEFVKNLLPKSLVNGVPWPQEATRRNSHTFSCRMLIRPPDEPGAENQEARQRYEVMQCF
TVSQPKSFKEEGEDFQSCLICIARRLPRPAAVTPSESFVTKQDTTGKIISIDTSSLRAAGRTGWEDLVRKCIYA
FFQPQGREPSYAKQLFQEVMTRGTAFSPSYRFTLSDGTVLSAHTKCKLCYPSSPEMQPFIMGIHVIDRDHGMLS
PQENTNSAMSLPRVSPSLNPSISPGQGMALPPSIPPSNGSMLATNVNQQPGAGLHNSNSSTSQTSFGCSPGNQI
GANVALSQGQAGPQNSNHTLNLSSSPMNSPGITPPQFMSPRHRVSPGLVPRPRVPSNPFSPTMPTMHSPVGMAN
SGSGGGCGTGGTRPFPNAPINSMQGLTEGPSTPVGYSTPPPVLRQLSSQSSPGRLAMQPMKAESKDSKEIASIL
SEMIQSDGGSGDGKPLDSGMLHAGDRLSEGDSKCSQATSHKLVQLLATTAEQQLRHADADTSCKDSLACAGTAG
TLASGNPPSSTCPSSHSSLTERHKILHRLLQEGSPSDITTLAMEHEKKENAPNPATTPTAPSQLPGTPDIKLEP
DTLKKKDVKDHQLLRYLLDKDEKELAAAPALSLDDVKVKVEKTEQMEPCNTAPVPLAKPPATEEVKLETQGQFA
AELEQLDQLLPSLEKAAQLPGLCGPERSESGVTGVAVKPEMLAAPLQPSTAARAPTGLNPMNSGQGVQAGRTPF
EFCDPLEPAQLRPVPCPSSNSSSPHARGPSQQGRGTVTAPNPFPPDTGMPELEMGVPDHQFGQPGMGEQIPWTD
NSMAAMNRGALNKPEDQCITSQLDELLCPPTTPEGRNDEKALLEQLVSFLSGKDETELAELDRALGIDKLVQGG

GLEALTERFQPQQATPPLMMEQKPGMYPQPYSSASPTTNLPGAFPGMVRQKPSFGPMPVQVPPPRGAFPTNMAM
QPRQTLNRPPTAPNQLRLQLQQRLQGQQQLIHQNRQAILNQFAANSPVGINMRAGMQQQITPQPPLNAQMLAQR
QRELYSQQHRQRQLMQQRAILMRQQSFGNNLPPSAGLPVQIGAARLPQAPPQQFPYPPNYGTTPGNPPTSTSPF
SPLASSPEAALANRSSMVNRGMMGGVGGQFGTGMTPQMQQNIFQYSGSGMGQQSEPAFAPSLSPSSPLMSPQLP
PSQSPMLQPAPPTPGYQSPDMKTWQQGAMGNSNVFSQAGQSQPAPAQQGMYNNMSITVSMAGGNTNVQNMNPMA
GQMQMNSLQMPGMNSMCSEQVNDPALRPAGLYCNQLSSTDLLKTEADGAQ
>108Brachypteracias leptosomus|A0A7L2V0X2|Aves|SRC1
CMVKMSGLGENSLDSLTNESRKRKPLPCDTPGASLTCSGEKRRREQESKYIEELAELISANLSDIDNFNVKPDK
CAILKETVRQIRQIKEQGKAVSNDDDVQKADVSSTGQGVIDKDSLGPLLLQAALDGFLFVVNRDGNIVFVSENV
TQYLQYKQEDLVNTSVYGILHEEDRKDFLKNLPKSSVNGVAWTNETPRQKSHTFNCRMMVKTSHDLLEDVGSHQ
DIRQRYETMQCFALSQPRAMMEEGEDLQSCMICVARRITTAERVFPANPESFITRHDLSGKLVNIDTNSLRSSM
RPGFEDIIRRCIQRFLCHNDGQSWSNKRHYHEAYTQGHAETPLYRFSLADGTIVTAQTKSKLFRNPVTNDRHGF
VSTHFLQREQNGYRPNPNSVGQNIRTPVAGNTNSVGGLMNMSPGQGMPVQNRNYGMGDPSAVGQLSGTRYAGPG
NMGPVNSGPGMQSSAYQNNSYGLNISSPPHGSPGLTSNQQNLMISPRNRGSPKMSSHQFSPVTGMHSPMASTSN
TGNSTFSSSSLSALQAISEGVGTSLLSTLSSPGPKLDNSPNMSIAQQSKTSTQDSKSPSGLYCEQNQVESSICQ
SNSRDLLSEKDSKEGNLEASESQRGPSESKGHKKLLQLLTCSSDERGHSTASNSPLDSNCKESSTNVTSPSGVS
SSTSAGVSSSSNVHGSLLQEKHRILHKLLQNGNSPAEVAKITAEATGKDTYHDASNTAPCGEGTVKQEQLSPKK
KENNALLRYLLDKDDVKDPLSKELKPKVEGVENKMGQCSSSTIPTSSQEKEMKIKTEPAEEMSGDLDNLDAILG
DLTSSEFYNNAMSANGNNLGTKQALFQGNALLGMRSPQSVQSTRPPFSRAMSLDSPVGSGAPGRNVNAFSMLQK
QNLMGGSPRMIENQENFGANLGFAPNRNVALTQHQSGEWGLSSSKGSRLEPAGSAPLLRAGPEFSAALARPAGG
GSVPGLPVRSNSMPATRAVLQQQMIHMRPNEINIGMGGNPYGQPGPATQPGSWPDNMLSMEQASRASQNRQLVR
NSLDDLLCPAPNMEGQNDERALLDQLHTLLSNTDVTGLEEIDRALGIPDLVNQGQALEPKQDSFQGQESSVMID
QKASLYSQPYQGQGAAMPGGFSNIQGQQPSFNSVMNQMSQQSNFPLQSMHPRANVMRPRTNTPKQLRMQLQQRL
QGQQFMNQTRQALEMKMENPVSGNTSVMRPVMQPQVGSQQQGFLNAQMVAQRNRELISHHFRQQRMAMMMQQQQ
QPQAFSPPPNVTASGNMDSALTGPPMAQVPPQQFPYPPNYGISQQPDPAFSRVSSPPNPMMSSRIGPSQNPMMQ
HPQTASMYQSPEMKGWPSGSMARSSSFPQQQFSHQGSPAAYSMMHMNGSSGHIGQMNINTMPMSGMPMGPDQVS
W
>109Pelecanoides urinatrix|A0A7L3BZ27|Aves|SRC1
MSGLGDSSTDPANPDSRKRKGSPCDTAQSNEKRRREQENKYLEELAELLSANIGDIDTLSVKPDKCKILKKTVD
QIQQMKRLEQEKTADDEVQKSDISSSSQGVIEKESLGPLLLEALDGFFFVVNREGRIVFVSENVTSYLGYNQEE
LMNTSVYSILHVGDHAEFVKNLLPKSLVNGVPWPQEATRRNSHTFSCRMLIRPPDEPGAENQEARQRYEVMQCF
TVSQPKSFKEEGEDFQSCLICIARRLPRPAAVTPSESFVTKQDTTGKIISIDTSSLRAAGRTGWEDLVRKCIYA
FFQPQGREPSYAKQLFQEVMTRGTAFSPSYRFTLSDGTVLSAHTKCKLCYPSSPEMQPFIMGIHVIDRDHGMLS
PQENTNSTMSLPRVSPSLNPSISPGQGMALPPSIPPSNGSMLATNVNQQPGAGLHNSNSSTSQTSFGCSPGNQI
GANVALSQGQAGPQNSNHTLNLNSSPMNSPGITPPQFMSPRHRVSPGLVPRPRVPSNPFSPTMPTMHSPVGMAN
SGSGGSGGTGGTRPFPNAPINSMQGLSEGPSTPVGYSTPPPVLRQLSSQSSPGRLAMQPMKAESKDSKEIASIL
SEMIQSDGGSGDGKPLDSGMLHAGDRLSEGDSKCSQATSHKLVQLLATTAEQQLRHADADTSCKDSLACAGTAG
TLASSNPPNSTCPSSHSSLTERHKILHRLLQEGSPSDITTLAMEHEKKENAPNPTTTPTAQSQLPGTPDIKLEP
EALKKKDVKDHQLLRYLLDKDEKELAAAPALSLDDVKVKVEKTEQMEPCNTAPVPLAKPPATEEVKLETQGQFA
AELEQLDQLLPSLEKAAQLPGLCGPERNESGVAGVAVKPEMLAAPLQPSTAARAPTGLNPMNSGQGVQAGRTPF
EFCDPLEPAQLRPVPCPTSNGSSPHARGPSQQGRGTVTAPNPFPPDAGMPELEMGVPDHQFGQPGMGEQIPWTD
NSMAAMNRGALNKPEDQCITSQLDELLCPPTTPEGRNDEKALLEQLVSFLSGKDETELAELDRALGIDKLVQGG
GLEALTERFQPQQATPPLMMEQKPGMYPQPYSSASPTTNLPGAFPGMVRQKPSFGPMPVQVPPPRGAFPTSMAM
QPRQTLSRPPTAPNQLRLQLQQRLQGQQQLIHQNRQAILNQFAANSPVGINMRAGMQQQITPQPPLNAQMLAQR
QRELYSQQHRQRQLMQQRAILMRQQSFGNNLPPSAGLPVQIGAARLPQAPPQQFPYPPNYGTTPGNPPTSTSPF
SPLASNPEAALANRSSMVNRGMMGGVGGQFGAGMTPQMQQNIFQYSGSGMGQQSEPAFAPSLSPSSPLMSPQLP
PSQSPMLQPAPPTPGYQSPDMKSWPQGAMGNSNVFSQAGQSQPAPAQQGMYNNMSITVSMAGGNTNVQNMNPMA
GQMQMNSLQMPGMNSMCSEQVNDPALRPAGLYCNQLSSTDLLKTEADGAQQVQQVQVFADVQCTVNLVGDPYLN
PPAPLGTPKNAAGPPPPPGQQKSLLQQLLTE
>110Pardalotus punctatus|A0A7L3I5S1|Aves|SRC1
CMVKMSGLGDNSLDSLTNESRKRKPVPCDTPGVSLACSGEKRRREQESKYIEELAELISANLSDIDNFNVKPDK
CAILKETVRQIRQIKEQGKAVSNDDDVQKADVSSTGQGVIDKDSLGPLLLQAALDGFLFVVNRDGNIVFVSDNV
TQYLQYKQEDLVNTSVYGILHEEDRKDFLKNLPKSSVNGVAWTNETPRQKSHTFNCRMMVKSCHDLLEDVGGHQ
DIRQRYETMQCFALSQPRAMMEEGEDLQSCMICVARRITTAERVFPTNPESFITRHDLSGKLVNIDTNSLRSSM

RPGFEDTIRRCIQRFLCHNDGQSWSNKRHYHEAYTQGHAETPLYRFSLADGTIVTAQTKSKLFRNPVTNDRHGF
VSTHFLQREQNGYRPNPNSVGQNVRTPVTGNANSVGGFMNMSPGQAVPMQNRSYGMGDSNAVGQLTSTRYGGPG
NMGPVNSGPGMQSSAYQNNNYGLNISSPPHGSPSLTSNQQNLMVSPRNRGSPKMSSHQYSPVTGMHSPVGSASS
ASNSTFSSSSLSALQAISEGVGNSLLSTLSSPGPKLDNSPNVSITQQSKTSAQDSKSPSGLYCEQNQVESSICQ
SNSRDVLSEKDSKEGNLEASECQRGPSDSKGHKKLLQLLTCSSDERGHSTASNSPLDSNCKESSNSVTSPSGVS
SSTSGAVSSSSNIHGSLLQEKHRILHKLLQNGNSPAEVAKITAEATGKDTYHDASNTASCGEGTVKQEQLSPKK
KENNALLRYLLDKDDIKDPLSKELKPKVEGVDNKMGQCTSSTIPTSSQEKEMKIKMEPAEEANGDLDNLDAILG
DLTSSEFYNNAMSANGNNLGTKPALFQGNALLGVKSPQSVQTTRLPFNRALSLDSPVGSGASVRNVNAFSMLQK
QNLMGGSPRMIENQENFGANLGFTPNRNVAMNQHQSGDWGLPNSKVNRLESGSSASMLRPGPEFSTTLPRPLEG
GSVSGLPVRSNSIPGTRPALQQQMIHMRPNEINVGMGGNLYGQPGPSNQPGLWPDNMMSTEQASRGSQNRQLVR
GSLDDLLCSAPNMEGQNDERALLDQLHTLLSNTDVIGLEEIDRALGIPDLVNQGQALEHKQDSFQGQESSVMID
QKPLLYGQPYQGQGAVMPAGFGNMQGQQPSFNSVMNQMSQPSNFPLQSIHPRANAMRPRTNAPKQLRMQLQQRL
QGQQFMNQTRQTLEIKMENPVSGTNSVMRPVMQPQVGSQQQGFLNAQMVAQRNRELISHHFRQQRMAMMMQQQQ
QPQAFSPPPNVTASGSMDSALPGPPMAQVPSQQFPYPPNYGISQQPDAAFSRVPSPPNPMIPRIGPSQNPMLQH
PQSAAMYQSSEMKGWPSGSVPRNSSFPQQQFSHQGNPAAYSMMHMNGSSGHIGQMSINTVPMSGMPMGPDQVSC
>111Rissa tridactyla|A0A7L3SL24|Aves|SRC1
CMFKMSGLGENSLDSLTNESRKRKPLPCDTPGASLTCSGEKRRREQESKYIEELAELISANLSDIDNFNVKPDK
CAILKETVRQIRQIKEQGKAVSNDDDVQKADVSSTGQGVIDKDSLGPLLLQASALDGFLFVVNRDGNIVFVSEN
VTQYLQYKQEDLVNTSVYGILHEEDRKDFLKNLPKSSVNGVAWTNETPRQKSHTFNCRMMVKTSHDLLEDVGSH
QDTRQRYETMQCFALSQPRAMMEEGEDLQSCMICVARRITTAERVFPTNPESFITRHDLSGKLVNIDTNSLRSS
MRPGFEDIIRRCIQRFLCHNDGQSWSNKRHYHEAYTQGHAETPLYRFSLADGTIVTAQTKSKLFRNPVTNDRHG
FVSTHFLQREQNGYRPNPNSVGQNIRTPVAGNTNSVGGLMNMSPGQGVPMQNRNYGMGDPSAVGQLSSTRYGGP
GNMGPVNSGPGMQSSAYQNNSYGLNISSPPHGSPGLASNQQNLMISPRNRGSPKMSSHQFSPVTGMHSPMGSAS
NTGNSTFSSSSLSALQAISEGVGTSLLSTLSSPGPKLDNSPNMSIAQQSKTSTQDSKSPSGLYCEQNQVESSIC
QSNSRDLLSEKDSKEGNLEAPESQRGPSESKGHKKLLQLLTCSSDERGHSTASNSPLDSNCKESSTNVTSPSGV
SSSTSGGVSSSSSNMHGSLLQEKHRILHKLLQNGNSPAEVAKITAEATGKDTYHDASNTAPCGEGTVKQEQLSP
KKKENNALLRYLLDKDDGKDPLSKELKPKVESVDNKMGQCSSSTIPTSSQEKEMKIKTEPAEEMSGDLDNLDAI
LGDLTSSEFYNNAMSANGNNLGTKQALFQGNALLGVRSPQSVPSSRPPFTRAMSLDSPVGSSAPVRNVNAFSML
QKQNLMGGSPRMIESQENFGANLGFAPNRNVAMNQHQSGEWGLPNSKVNRLEPTSSASMLRPGPEFSASLPRSS
VGGSMSGLPVRSNSIPGTRPMLQQQMIHMRPNEINIGMGGNPYGQPGPSNQPGSWPDNMLSMEQASRGSQNRQL
VRNSLDDLLCPAPNVEGQNDERALLDQLHTLLSNTDVTGLEEIDRALGIPDLVNQGQALEPKQDSFQGQESSVM
IDQKPSLYGQPYQGQGAAMPGSFSNIQGQQPTFNSVMNQMSQQSNFPLQSMHPRANVMRPRTNTPKQLRMQLQQ
RLQGQQFMNQTRQALEMKMENPVSGNTSVMRPVMQPQVGSQQQGFLNAQMVAQRNRELISHHFRQQRMAMMMQQ
QQQPQAFSPPPNVTASGSMDSALTGPPMAQVPPQQFPYPPNYGISQQPDPAFSRVSSPPNPMMASRIGASQNPM
MQHPQTAAMYQSPEMKGWPSGSMARSSSFPQQQFSHQGNPAAYSMMHMNGSSGHIGQMNINTMPMSGMPMGPDQ
VRR
>112Rissa tridactyla|A0A7L3T852|Aves|SRC1
MSGLGDSSTDPANPDSRKRKGSPCDTAQSNEKRRREQENKYLEELAELLSANIGDIDTLSVKPDKCKILKKTVD
QIQQMKRLEQEKAADDEVQKSDISSSSQGVIEKESLGPLLLEALDGFFFVVNREGRIVFVSENVTGYLGYNQEE
LMNTSVYSILHVGDHAEFVKNLLPKSLVNGVPWPQEATRRNSHTFSCRMLIRPPDEPGAENQEARQRYEVMQCF
TVSQPKSFKEEGEDFQSCLICIARRLPRPAAVTPSESFVTKQDTTGKIISIDTSSLRAAGRTGWEDLVRKCIYA
FFQPQGREPSYAKQLFQEVMTRGTAFSPSYRFTLSDGTVLSAHTKCKLCYPSSPEMQPFIMGIHVIDRDHGMLS
PQENTNSAMSLPRVSPSLNPSISPGQGMALPPSIPPSNGSMLATNVNQQPGAGLHNSNSSTSQTSFGCSPGNQI
GANVALSQGQAGPQNSNHTLNLNSSPMNSPGITPPQFMSPRHRVSPGLVPRPRVPSNPFSPTMPTMHSPVGMAN
SGSGGGGGTGGARPFPSAPINSMQGLTEGPSTPVGYSTPPPVLRQLSSQSSPGRLAMQPMKAESKDSKEIASIL
SEMIQSDGGSGDGKPLDSGMLHAGDRLSEGDSKCSQATSHKLVQLLATTAEQQLRHADADTSCKDSLACAGTAG
TLASSNPPSSTCPSSHSSLTERHKILHRLLQEGSPSDITTLAMEHEKKENAPNPATTPTAQSQLPGTPDIKLEP
DALKKKDVKDHQLLRYLLDKDEKELAAAPALSLDDVKVKVEKTEQMEPCNTAPVPLAKPPAAEEVKLETQGQFA
AELEQLDQLLPSLEKAAQMPGLCGPERSESSVAGVTVKPEMLAAPLQPSTAARAPTGLNPMPSGQGVQAGRPPF
EFCDPLEPAQLRPVPCPASNGSSPHARGPSQQGRGTVTAPNPFPPDTGMPELEMGVPDHQFGQPGMGEQIPWTD
NSMAAMNRGALNKPEDQCITSQLDELLCPPTTPEGRNDEKALLEQLVSFLSGKDETELAELDRALGIDKLVQGG
GLEALTERFQPQQATPPLMMDQKPGMYPQPYSSASPTTNLPGAFPGMVRQKPSFGPMPVQVPPPRGAFPTNMAM
QPRQTLNRPPTAPNQLRLQLQQRLQGQQQLIHQNRQAILNQFAANSPVGINMRAGMQQQITPQPPLNAQMLAQR
QRELYSQQHRQRQLMQQRAILMRQQSFGNNLPPSAGLPVQIGAARLPQAPPQQFPYPPNYGTNPGNPPTSTSPF

SPLASNPEAALANRSSMVNRGMMGGVGGQFGAGMSPQMQQNIFQYSGSGMGQQSEPAFAPSLSPSSPLMSPQLP
PSQSPMLQPAPPTPGYQSPDMKTWQQGAMGNSNVFSQAGQSQPAPAQQGMYNNMSITVSMAGGNANVQNMNPMA
GQMQMNSLQMPGMNSMCSEQVNEPALRPAGLYCNQLSSTDLLKTEADGAQQVQQVQVFADVQCTVNLVGDPYLN
PPAPLGTPKNAAGPPPPPGQQKSLLQQLLTE
>113Circaetus pectoralis|A0A7L4AEA1|Aves|SRC1
MSGLGDSSTDPANPDSRKRKGSPCDTAQSNEKRRREQENKYLEELAELLSANIGDIDTLSVKPDKCKILKKTVD
QIQQMKRLEQEKAADDEVQKSDISSSSQGVIEKESLGPLLLEALDGFFFVVNREGRIVFVSENVTSYLGYNQEE
LMNTSVYSILHVGDHAEFVKNLLPKSLVNGVPWPQEATRRNSHTFSCRMLIRPPDEPGTENQEARQRYEVMQCF
TVSQPKSFKEEGEDFQSCLICIARRLPRPAAVTPSESFVTKQDTTGKIISIDTSSLRAAGRTGWEDLVRKCIYA
FFQPQGREPSYAKQLFQEVMTRGTAFSPSYRFTLSDGTVLSAHTKCKLCYPSSPEMQPFIMGIHVIDRDHGMLS
PQENTNSAMSLPRVSPSLNASISPGQGMALPPSIPPSNGSMLATNVNQQPGAGLHNSNSSTSQTSFGCSPGNQI
GANVALSQGQAGPQNSNHTLNLNSSPMNSPGITPPQFMSPRHRVSPGLVPRPRVPSNPFSPTMPTMHSPVGMAN
SGSGGSCGTGGTRPFPSAPINSMQGLTEGPSTPVGYSTPPPVLRQLSSQSSPGRLAMQPMKAESKDSKEIASIL
SEMIQSDGGSGDGKPLDSGMLHASDRLSEGDSKCSQATSHKLVQLLATTAEQQLRHADADTSCKDSLACVGTAG
TLASSNPPSSTCPSSHSSLTERHKILHRLLQEGSPSDITTLAMEHEKKENTPNATTAPTAQSQLPGTPDIKLEP
DALKKKDVKDHQLLRYLLDKDEKELAAAPALTLDDVKVKVEKTEQMEPCNTAPVPLAKPPATEEVKLETQGQFA
AELEQLDQLLPSLEKAAQLPGLCGPERSESSVTSVAVKPEMLAAPLQPSTAARAPTGLNPMNSGQGVQAGRTPF
EFCDPLEPAQLRPVPCPASNGGSPHTRGPSQQGRGTVTAPNPFPPDTGMPELEMGVPDHQFGQPGMGEQIPWTD
NSMAAMNRGALNKPEDQCITSQLDELLCPPTTPEGRNDEKALLEQLVSFLSGKDETELAELDRALGIDKLVQGG
GLEALTERFQPQQATPPLMMEQKPGMYPQPYSSASPTTNLPGAFPGMVRQKPSFGPMPVQVPPPRGAFPTNMAM
QPRQTLNRPPTAPNQLRLQLQQRLQGQQQLIHQNRQAILNQFAANSPVGINMRAGMQQQITPQPPLNAQMLAQR
QRELYSQQHRQRQLMQQRAILMRQQSFGNNLPPSAGLPVQIGAARLPQAPPQQFPYPPNYGTTPGNPPTSTSPF
SPLASNPEAALANRSSMVNRGMMGGVGGQFGAGMTPQMQQNIFQYSGSGMGQQSEPAFAPSLSPSSPLMSPQLP
PSQSPMLQPAPPTPGYQSPDMKTWQQGAMGNSNVFSQAGQSQPAPAQQGMYNNMSITVSMAGGNTNVQNMNPMA
GQMQMNSLQMPGMNSMCSEQVNDPALRPAGLYCNQLSSTDLLKTEADGAQQVQQVQVFADVQCTVNLVGDPYLN
PPAPLGTPKNAAGPPPPPGQQKSLLQQLLTE
>114Phaetusa simplex|A0A7L4ANA9|Aves|SRC1
CMFKMSGLGENSLDSLTNESRKRKPLPCDTPGASLTCSGEKRRREQESKYIEELAELISANLSDIDNFNVKPDK
CAILKETVRQIRQIKEQGKAVSNDDDVQKADVSSTGQGVIDKDSLGPLLLQASALDGFLFVVNRDGNIVFVSEN
VTQYLQYKQEDLVNTSVYGILHEEDRKDFLKNLPKSSVNGVAWTNETPRQKSHTFNCRMMVKTCHDLLEDVGSH
QDTRQRYETMQCFALSQPRAMMEEGEDLQSCMICVARRITTAERVFPTNPESFITRHDLSGKLVNIDTNSLRSS
MRPGFEDIIRRCIQRFLCHNDGQSWSNKRHYHEAYTQGHAETPLYRFSLADGTIVTAQTKSKLFRNPVTNDRHG
FVSTHFLQREQNGYRPNPNSVGQNIRTPVAGNTNSVGGLMNMSPGQGVPMQNRNYGMGDPSAVGQLSSTRYGGP
GNMGPVNSGPGMQSSAYQNNSYGLNISSPPHGSPGLTSNQQNLMISPRNRGSPKMSSHQFSPVTGVHSPMGSAS
NTGNSTFSSSSLSALQAISEGVGTSLLSTLSSPGPKLDNSPNMSIAQQSKMSTQDSKSPSGLYCEQNQVESSIC
QSNSRDLLSEKDSKEGNLEAPESQRGPSESKGHKKLLQLLTCSSDERGHSTASNSPLDSNCKESSTNVTSPSGV
SSSTSGGVSSSSSNMHGSLLQEKHRILHKLLQNGNSPAEVAKITAEATGKDTYHDASNTAPCGEGTVKQEQLSP
KKKENNALLRYLLDKDDVKDPLSKELKPKVESVDNKMGQCSSSTIPTSSQEKEMKIKMEPAEEMSGDLDNLDAI
LGDLTSSEFYNNAMSANGNNLGTKQALFQGNALLGVRSPQSVPTTRPPFTRAMSLDSPVGSSAPVRNVNAFSML
QKQNLMGGSPRMIESQENFGANLGFAPNRNVVMNQHQSGEWGLPNSKVNRLEPTSSASMLRPGPEFSASLPRSS
VGGSMSGLPVRSNSIPGTRPMLQQQMIHMRPNEINIGMGANPYGQPGPSNQPGSWPDNMLSMEQASRGSQSRQL
VRSSLDDLLCPAPNVEGQNDERALLDQLHTLLSNTDVTGLEEIDRALGIPDLVNQGQALEPKQDSFQGQESSVM
IDQKPSLYGQPYQGQGAAMPGSFSNIQGQQPTFNSVMNQMSQQSNFPLQSMHPRANVMRPRTNTPKQLRMQLQQ
RLQGQQFMNQTRQALEMKMENPVSGNTSVMRPVMQPQVGSQQQGFLNAQMVAQRNRELISHHFRQQRMAMMMQQ
QQQQPQAFSPPPNVTASGSMDSALTGPPMAQVPPQQFPYPPNYGISQQPDPAFSRVSSPPNPMMSSRIGASQNP
MMQHPQTAAMYQSPEMKGWPSGSMARSSSFPQQQFSHQGNPAAYSLMHMNGSSGHIGQMNINTMPMSGMPMGPD
QVSR
>115Sarcophilus harrisii|A0A7N4NS31|Aves|SRC1
MSGLGDSSTDPSNPDSHKRKGSPCDTLASSTEKRRREQENKYLEELAELLSANIGDIDSLSVKPDKCKILKKTV
DQIQQMKRMEQEKTATDDDVQKSDISSSSQGVIEKESLGPLLLEALDGFFFVVNREGRIVFVSENVTSYLGYNQ
EELMNTSVYSILHVGDHAEFVKNLLPKSLVNGVPWPQEATRRNSHTFNCRMLIRPPDEPGTENQEACQRYEVMQ
CFTVSQPKSIQEEGEDFQSCLICIARRLPRPAAITGSESFMTKQDTTGKIISIDTSSLRAAGRTGWEDLVRKCI
YAFFQPQGREPSYARQLFQEVMTRGTAFSPSYRFILNDGTMLSAHTKCKLCYPQSPDMQPFIMGIHIIDREHSV
LSPQDNTNSGMSLPRINPSVNPSISPAQGMARSSTLPPSNNNLVTPRVNHQPSSDLHSSSSNSNSNTNQGSFGC

SSGNQVVASVALNQGQAGSQGSNHSLSLNNSPMDSTGITLAQFMSPRRQVSSGLATRPRMPSNSFSPNIPSLSS
PIGMVGSACGNPNRTYSNIPATSLQGINEGPSNSIGFSATSPVLRQLSSQNSPSRMNMLPTKAESKDSKEITSI
LNEMVQSDNSSNDVKPLDSGLLHNSDKLSEGENKYSQTSHKLVQLLATTAEQQLRHTDADTSGKDALSCSGTST
STANNSSSSGNTCPSSHSSLTERHKILHRLLQEGSPSDITTLSVEHDKKDNGSSSVSNQVPGNSNIKLELEASK
KKESKDHQLLRYLLDKDEKDLRAAPTLSLDDVKVKVEKKEQMDPCNTTPTPLTKPAPEEIKLESQNQFPADLEQ
FDPLLPTLEKASQLPSLCESERIEAGGTGVTVKSEILPTALQSTPARPPPRLNRLPELELGGLDHQFGQPGIDD
QVPWANNTLTAMNQGPLNKSEDQCITSQLDELLCPPTTIEGRNDEKALLDQLVSFLSGTDETELAELDRALGID
KLVQGGGLEVLTERFQPQQSTPPLMMEERSSLYSQPYSSASPTANLPGPFQGMIRQKPSMGTMPVQVPPPRGAF
PTNMSMQPRQTLNRPPAAPNQLRLQLQQRLQGQQQLIHQNRQAILNQFAASAPVGINMRSGIQQQITPQPPLNA
QMLAQRQRELYSQQHRQRQLIQQQRALLMRQQSFGNNLPPSSGLPVQMGTTRLPQGAPQQFPYPSSYGTNPGTP
PASTSPFSQLTSNPEASLASRNNMVNRGMTGNVGGQFGTGINPQMQQSVFQYPGSGLGQQGEPNFTPSLSPGSP
MMSLQIPPSQSPMLQQAQPSSGYQSPDMKTWQQGAIGNNNMFSQNQPTPAQPGVYNNMSITVSMAGGNTNVQNM
NPLMGQMQMSSLQMPGMNTVCSEQINDPALRHTGLYCNQLSSADILKTESDGSAQQVQQVQVFADVQCTVNLVG
GDPYLNQPGPLGSQKPTSGPQNPQAQQKSLLQQLLTE
>116Sarcophilus harrisii|A0A7N4P165|Aves|SRC1
MSGLGDSSTDPSNPDSHKRKGSPCDTLASSTEKRRREQENKYLEELAELLSANIGDIDSLSVKPDKCKILKKTV
DQIQQMKRMEQEKTATDDDVQKSDISSSSQGVIEKESLGPLLLEALDGFFFVVNREGRIVFVSENVTSYLGYNQ
EELMNTSVYSILHVGDHAEFVKNLLPKSLVNGVPWPQEATRRNSHTFNCRMLIRPPDEPGTENQEACQRYEVMQ
CFTVSQPKSIQEEGEDFQSCLICIARRLPRPAAITGSESFMTKQDTTGKIISIDTSSLRAAGRTGWEDLVRKCI
YAFFQPQGREPSYARQLFQEVMTRGTAFSPSYRFILNDGTMLSAHTKCKLCYPQSPDMQPFIMGIHIIDREHSV
LSPQDNTNSGMSLPRINPSVNPSISPAQGMARSSTLPPSNNNLVTPRVNHQPSSDLHSSSSNSNSNTNQGSFGC
SSGNQVVASVALNQGQAGSQGSNHSLSLNNSPMDSTGITLAQFMSPRRQVSSGLATRPRMPSNSFSPNIPSLSS
PIGMVGSACGNPNRTYSNIPATSLQGINEGPSNSIGFSATSPVLRQLSSQNSPSRMNMLPTKAESKDSKEITSI
LNEMVQSDNSSNDVKPLDSGLLHNSDKLSEGENKYSQTSHKLVQLLATTAEQQLRHTDADTSGKDALSCSGTST
STANNSSSSGNTCPSSHSSLTERHKILHRLLQEGSPSDITTLSVEHDKKDNGSSSVSNQVPGNSNIKLELEASK
KKESKDHQLLRYLLDKDEKDLRAAPTLSLDDVKVKVEKKEQMDPCNTTPTPLTKPAPEEIKLESQNQFPADLEQ
FDPLLPTLEKASQLPSLCESERIEAGGTGVTVKSEILPTALQSTPARPPPRLNRLPELELGGLDHQFGQPGIDD
QVPWANNTLTAMNQGPLNKSEDQCITSQLDELLCPPTTIEGRNDEKALLDQLVSFLSGTDETELAELDRALGID
KLVQGGGLEVLTERFQPQQSTPPLMMEERSSLYSQPYSSASPTANLPGPFQGMIRQKPSMGTMPVQVPPPRGAF
PTNMSMQPRQTLNRPPAAPNQLRLQLQQRLQGQQQPPLNAQMLAQRQRELYSQQHRQRQLIQQQRALLMRQQSF
GNNLPPSSGLPVQMGTTRLPQGAPQQFPYPSSYGTNPGTPPASTSPFSQLTSNPEASLASRNNMVNRGMTGNVG
GQFGTGINPQMQQSVFQYPGSGLGQQGEPNFTPSLSPGSPMMSLQIPPSQSPMLQQAQPSSGYQSPDMKTWQQG
AIGNNNMFSQNQPTPAQPGVYNNMSITVSMAGGNTNVQNMNPLMGQMQMSSLQMPGMNTVCSEQINDPALRHTG
LYCNQLSSADILKTESDGSAQDKKTEEFFSVVTTD
>117Sarcophilus harrisii|A0A7N4V5S3|Aves|SRC1
MSGLGDSSTDPSNPDSHKRKGSPCDTLASSTEKRRREQENKYLEELAELLSANIGDIDSLSVKPDKCKILKKTV
DQIQQMKRMEQEKTATDDDVQKSDISSSSQGVIEKESLGPLLLEALDGFFFVVNREGRIVFVSENVTSYLGYNQ
EELMNTSVYSILHVGDHAEFVKNLLPKSLVNGVPWPQEATRRNSHTFNCRMLIRPPDEPGTENQEACQRYEVMQ
CFTVSQPKSIQEEGEDFQSCLICIARRLPRPAAITGSESFMTKQDTTGKIISIDTSSLRAAGRTGWEDLVRKCI
YAFFQPQGREPSYARQLFQEVMTRGTAFSPSYRFILNDGTMLSAHTKCKLCYPQSPDMQPFIMGIHIIDREHSV
LSPQDNTNSGMSLPRINPSVNPSISPAQGMARSSTLPPSNNNLVTPRVNHQPSSDLHSSSSNSNSNTNQGSFGC
SSGNQVVASVALNQGQAGSQGSNHSLSLNNSPMDSTGITLAQFMSPRRQVSSGLATRPRMPSNSFSPNIPSLSS
PIGMVGSACGNPNRTYSNIPATSLQGINEGPSNSIGFSATSPVLRQLSSQNSPSRMNMLPTKAESKDSKEITSI
LNEMVQSDNSSNDVKPLDSGLLHNSDKLSEGENKYSQTSHKLVQLLATTAEQQLRHTDADTSGKDALSCSGTST
STANNSSSSGNTCPSSHSSLTERHKILHRLLQEGSPSDITTLSVEHDKKDNGSSSVSNQVPGNSNIKLELEASK
KKESKDHQLLRYLLDKDEKDLRAAPTLSLDDVKVKVEKKEQMDPCNTTPTPLTKPAPEEIKLESQNQFPADLEQ
FDPLLPTLEKASQLPSLCESERIEAGGTGVTVKSEILPTALQSTPARPPPRLNRLPELELGGLDHQFGQPGIDD
QVPWANNTLTAMNQGPLNKSEDQCITSQLDELLCPPTTIEGRNDEKALLDQLVSFLSGTDETELAELDRALGID
KLVQGGGLEVLTERFQPQQSTPPLMMEERSSLYSQPYSSASPTANLPGPFQGMIRQKPSMGTMPVQVPPPRGAF
PTNMSMQPRQTLNRPPAAPNQLRLQLQQRLQGQQQLIHQNRQAILNQFAASAPVGINMRSGIQQQITPQPPLNA
QMLAQRQRELYSQQHRQRQLIQQQRALLMRQQSFGNNLPPSSGLPVQMGTTRLPQGAPQQFPYPSSYGTNPGTP
PASTSPFSQLTSNPEASLASRNNMVNRGMTGNVGGQFGTGINPQMQQSVFQYPGSGLGQQGEPNFTPSLSPGSP
MMSLQIPPSQSPMLQQAQPSSGYQSPDMKTWQQGAIGNNNMFSQNQPTPAQPGVYNNMSITVSMAGGNTNVQNM
NPLMGQMQMSSLQMPGMNTVCSEQINDPALRHTGLYCNQLSSADILKTESDGSAQDKKTEEFFSVVTTD

>118Macaca fascicularis|A0A7N9DBY9|Aves|SRC1
MSGLGDSSSDPANPDSHKRKGSPCDTLASSTEKRRREQENKYLEELAELLSANISDIDSLSVKPDKCKILKKTV
DQIQLMKRMEQEKSTTDDDVQKSDISSSSQGVIEKESLGPLLLEALDGFFFVVNCEGRIVFVSENVTSYLGYNQ
EELMNTSVYSILHVGDHAEFVKNLLPKSLVNGVPWPQEATRRNSHTFNCRMLIHPPDEPGTENQEACQRYEVMQ
CFTVSQPKSIQEDGEDFQSCLICIARRLPRPPAITGVESFMTKQDTTGKIISIDTSSLRAAGRTGWEDLVRKCI
YAFFQPQGREPSYARQLFQEVMTRGTASSPSYRFILNDGTMLSAHTKCKLCYPQSPDMQPFIMGIHIIDREHSG
LSPQDDTNSGMSIPRVNPSVNPSISPAHGVARSSTLPPSNSNMVSTRINRQQSSDLHSGSHSNSSNSQGSFGCS
PGSQIVANVALNQGQASSQSSNPSLNLNNSPMEGTGISLAQFMSPRRQVTSGLTTRPRMPNNSFPPNISTLSSP
VGMTSSACNNNTNRSYSNIPVTSLQGMNEGPNNSVGFSANSPVLRQMSSQNSPSRLNIQPAKAESKDNKEIASI
LNEMIQSDNSSSDGKPLDSGLLHNNDRLSDGDSKYSQTSHKLVQLLTTTAEQQLRHADIDTSCKDVLSCTGTSN
SASANSSGGSCPSSHSSLTERHKILHRLLQEGSPSDITTLSVEPDKKDSASTSVSVTGQVQGNSSIKLELDASK
KKESKDHQLLRYLLDKDEKDLRSTPNLSLDDVKVKVEKKEQMDPCNTNPTPMTKPTPEEIKLEAQSQFTADLDQ
FDQLLPTLEKAAQLPGLCETDRMDGAVTSVTIKSEILPASLQSATARPTSRLNRLPELELEAIDNQFGQPGTGD
QIPWTNNTVTAINQNKSEDQCISSQLDELLCPPTTVEGRNDEKALLEQLVSFLSGKDETELAELDRALGIDKLV
QGGGLDVLSERFPPQQATPPLMMEERPNLYSQPYSSPSPTANLPSPFQGMVRQKPSLGTMPVQVTPPRGAFSPG
MGMQPRQTLNRPPAAPNQLRLQLQQRLQGQQQLIHQNRQAILNQFAATAPVGINMRSGMQQQITPQPPLNAQML
AQRQRELYSQQHRQRQLIQQQRAMLMRQQSFGNNLPPSSGLPVQMGNPRLPQGAPQQFPYPPNYGTNPGTPPAS
TSPFSQLAANPEASLANRNSMVSRGMTGNMGGQFGTGINPQIQQNVFQYPGAGMVPQGEASFAPSLSPGSSMVP
MPIPPPQSSLLQQTPPASGYQSPDMKAWQQGAIGNNVFSQAVQNQPTPAQPGVYNNMSITVSMAGGNTNVQNMN
PMMAQMQMSSLQMPGMNTVCPEQQVQQVQVFADVQCTVNLVGGDPYLNQPGPLGTQKPTSGPQTPQAQQKSLLQ
QLLTE
>119Macaca fascicularis|A0A7N9IFH4|Aves|SRC1
MSGLGDSSSDPANPDSHKRKGSPCDTLASSTEKRRREQENKYLEELAELLSANISDIDSLSVKPDKCKILKKTV
DQIQLMKRMEQEKSTTDDDVQKSDISSSSQGVIEKESLGPLLLEALDGFFFVVNCEGRIVFVSENVTSYLGYNQ
EELMNTSVYSILHVGDHAEFVKNLLPKSLVNGVPWPQEATRRNSHTFNCRMLIHPPDEPGTENQEACQRYEVMQ
CFTVSQPKSIQEDGEDFQSCLICIARRLPRPPAITGVESFMTKQDTTGKIISIDTSSLRAAGRTGWEDLVRKCI
YAFFQPQGREPSYARQLFQEVMTRGTASSPSYRFILNDGTMLSAHTKCKLCYPQSPDMQPFIMGIHIIDREHSG
LSPQDDTNSGMSIPRVNPSVNPSISPAHGVARSSTLPPSNSNMVSTRINRQQSSDLHSGSHSNSSNSQGSFGCS
PGSQIVANVALNQGQASSQSSNPSLNLNNSPMEGTGISLAQFMSPRRQVTSGLTTRPRMPNNSFPPNISTLSSP
VGMTSSACNNNTNRSYSNIPVTSLQGMNEGPNNSVGFSANSPVLRQMSSQNSPSRLNIQPAKAESKDNKEIASI
LNEMIQSDNSSSDGKPLDSGLLHNNDRLSDGDSKYSQTSHKLVQLLTTTAEQQLRHADIDTSCKDVLSCTGTSN
SASANSSGGSCPSSHSSLTERHKILHRLLQEGSPSDITTLSVEPDKKDSASTSVSVTGQVQGNSSIKLELDASK
KKESKDHQLLRYLLDKDEKDLRSTPNLSLDDVKVKVEKKEQMDPCNTNPTPMTKPTPEEIKLEAQSQFTADLDQ
FDQLLPTLEKAAQLPGLCETDRMDGAVTSVTIKSEILPASLQSATARPTSRLNRLPELELEAIDNQFGQPGTGD
QIPWTNNTVTAINQNKSEDQCISSQLDELLCPPTTVEGRNDEKALLEQLVSFLSGKDETELAELDRALGIDKLV
QGGGLDVLSERFPPQQATPPLMMEERPNLYSQPYSSPSPTANLPSPFQGMVRQKPSLGTMPVQVTPPRGAFSPG
MGMQPRQTLNRPPAAPNQLRLQLQQRLQGQQQLIHQNRQAILNQFAATAPVGINMRSGMQQQITPQPPLNAQML
AQRQRELYSQQHRQRQLIQQQRAMLMRQQSFGNNLPPSSGLPVQMGNPRLPQGAPQQFPYPPNYGTNPGTPPAS
TSPFSQLAANPEASLANRNSMVSRGMTGNMGGQFGTGINPQIQQNVFQYPGAGMVPQGEASFAPSLSPGSSMVP
MPIPPPQSSLLQQTPPASGYQSPDMKAWQQGAIGNNVFSQAVQNQPTPAQPGVYNNMSITVSMAGGNTNVQNMN
PMMAQMQMSSLQMPGMNTVCPEQINDPALRHTGLYCNQLSSTDLLKTEADGTQDKKTEEFFSVVTTD
>120Anolis carolinensis|A0A803SPC1|Aves|SRC1
MSGLGNNSMDPANPDSQKRKGSPCDTLAQSNEKRRREQENKYLEELADLLSANIGEIDSLSVKPDKCKILKKTV
DQIQQMKRLEQEKTADDDVQKSDISSSSQGVIEKESLGPLLLEALDGFFFVVNREGRIVFVSENVTSYLGYNQD
ELMNTSVYSILHVGDHAEFVKNLLPKSLVNGVPWPQESTRRNSHTFNCRMLTRPPDESAAENQEARQRYEVMQC
FTVSQPKLIKEEGEDLQSCLICIARRLPRSTVPTTSESFMTKQDTTGKIISIDTSSLRAAGRTGWEDLVRKCIY
AFFQPQGREPSYAKQLFQEVMTRGTAFSPSYRFTLSDGTILSAHTKCRLCCPQSPEMQPFIMGIHIIEREHGPL
SPQDNTNSGMPVPRVNPALNPNISPGQGMPLSTPLPPTNNGNMLPSNPGHPPGPGLHANSGGAAATGPAGFGCS
PGSQMGPNVALSQGQAAPQNSSHAMNLSSSPMNSPGVTPPQFMSPRHRVSPGLVHRPRVPSNPFSPTMPTTHSP
VGMMGGCGVASAVGNRPYPGTPLNSMQGLREGPSPPVGYSTPSPVLRQLSNQNSPSRMSMPPMKTEPKDSKEMG
SIMCGMAHSDSVSGDSKPLDSSLLHGGDRLSEGDSKCLQATSQKLVQLLATTAQEQLRHADADTSCKEPLSCTG
TSGTPSSGNPVGSACPSSHSSLTERHKILHRLLQEGSPSDINSLAMEHDKKDGVAGGNTAAVAAQNQGPGTPDM
RLEADAMKKKESKDHQLLRYLLDKDEKELASNPTLSLDDVKVKENPEQLESCSITPKQEDVKLQPQGQFASDLD
QILPSLEKAAQLPPGLCGSETAESGGISIKSEILTPSLQPTTTARTPSGMSSVNGGQGMQAGRSPYEICDPLEP

VQPRTAPFPPMNGSPHPGLPPELARGAMSGPTPYQPDTGLPELELGVTDHQFGQPTMGEQLPWADNAMTSINQG
PISKPDDHCITSQLDELLCPPTTPEGRNDEKALLEQLVSFLSGKDENELAELDRALGIDKLVQGGGLEALTERF
QPQQAAPAPPPMAMDQKPSLYPQPYPSASPNANLAGPFQGMVRQKQSFGPMPVQVPPPRGGFPTNMGMQPRQAL
SRPPTAPNQLRLQLQQRLQGQQQLIHQNRQAILNQFAGNPPGSMGMRPSMQQQIAGQPPLNAQMLAQRQRELYS
QQHRQRQLMQQRAMLMRQQQSFGSSLPPSAGVPVPMGAGRLPQAPPQQFPFPPNYGTNPGNPPASTSPFSQLAA
SNPEAALASRNNMVNRGMMGSVGGQFGTNMSPQMQQNIFQYSGSGMGQQGEAAFAPSLSPGSPLMSPQIPPSQS
PMLQSAPPTPGYQSPDMKTWQQGAMGNNSVFGQAGQSQPSPAQQGMYNNMSITVSMAGGNANVQNMNPMAGQMP
MSSLQMPGMNQLCPEQVNDPSLRPSGLYCNQLSSDLLKTEADGAQGKKTEEFFFSVVTTG
>121Meleagris gallopavo|A0A803XKQ0|Aves|SRC1
MSGLGDSSTDPANPDSRKRKGSPCDTAQSNEKRRREQENKYLEELAELLSANIGDIDTLSVKPDKCKILKKTVD
QIQQMKRLEQEKAADDEVQKSDISSSSQGVIEKESLGPLLLEALDGFFFVVNREGRIVFVSENVTSYLGYNQEE
LMNTSVYSILHVGDHTEFVKNLLPKSLVNGVPWPQEATRRNSHTFSCRMLIRPPDEPGTENQEARQRYEVMQCF
TVSQPKTFKEEGEDFQSCLICIARRLPRPAAVTPSESFVTKQDTTGKIISIDTSSLRAAGRTGWEDLVRKCIYA
FFQPQGREPSYAKQLFQEVMTRGTAFSPSYRFTLSDGTVLSAHTKCKLCYPSSPEVQPFIMGIHIIDRDHGMLP
SQENTNSAMALPRVSPSLNPNISPGQGMALPPSIPPSNGSMLATPGNQQPGAGLHSSNSSTSQTSFGCSPGNQI
GANVALSQGQAGPPNSNHNLNLNSSPMNSPGITPPQFMSPRHRVSPGLVPRPRGPSNPFSPTMPTMHSPVGMAN
SGGAATAVAVAGLSEGASTPVGYSTPPPVLRQLGSQSSPGRLGMQPMKAESKDSKEIASILSEMIQSDGGAGDG
KPLDSGVLHAGERLTEGESKCSQSTSHKLVQLLASTAEQQLRHADADTSCKDSLACAGTTSALGASNPPSSTCP
SSHSSLTERHKILHRLLQEGSPSDITTLAMEHDKKDNVPNPATTPTAPSQLPGTPDIKLESDAMKKKDVKDHQL
LRYLLDKDEKELAAAPALSLDDVKVKVEKTEQMEPCNTTPVPLAKPPAAEEVKLESQGQFAAELEQLDQLLPSL
EKAAQLPGLCGPERSESGVAVKPEMLAAPLQPSTAPRAPAGLNRMSELEMGVPDHQFGQPGMGEQIPWTDNSMA
PMNRSTLSKPEDPCITSQLDELLCPPTTPEGRNDEKALLEQLVSFLSGKDETELAELDRALGIDKLVQGGGLEA
LTERFQPQQATPPLMMEQKPGMYPQPYSSASPTTNLPAAFPGMVRQKPSFGPMPVQVPPPRGAFPTTMAMQPRQ
TLSRPPTAPNQLRLQLQQRLQGQQQLIHQNRQAILNQFAANSPVGINMRAGMQQQMTPQPPLNAQMLAQRQREL
YSQQHRQRQLMQQRAILMRQQSFGNNLPPSAGLPVQMGAARLPQAPPQQFPYPPNYGTSPGNPPTSTSPFSQLA
SSPEAALANRGGMVTRGMMGSVGGQFGAGMTPQMQQNIFQYSGSGMGQQNEPAFAPSLSPSSPLMSPQLPPSQS
PMLQPAPPAPGYQSPDMKTWQQGAMGNSNVFSQTGQTQPAPAQQGMYNNMSITVSMAGGNTNVQNMNPMAGQMQ
MNSLQMPGMNSMCSEQVNDPALRPTGLYCNQLSSTDLLKTEADGAQQVQQVQVFADVQCTVNLVGDPYLNPPAP
LGAPKAAAVPPTPQGQQKSLLQQLLTE
>122Meleagris gallopavo|A0A803YEQ2|Aves|SRC1
MSGLGDSSTDPANPDSRKRKGSPCDTAQSNEKRRREQENKYLEELAELLSANIGDIDTLSVKPDKCKILKKTVD
QIQQMKRLEQEKAADDEVQKSDISSSSQGVIEKESLGPLLLEALDGFFFVVNREGRIVFVSENVTSYLGYNQEE
LMNTSVYSILHVGDHTEFVKNLLPKSLVNGVPWPQEATRRNSHTFSCRMLIRPPDEPGTENQEARQRYEVMQCF
TVSQPKTFKEEGEDFQSCLICIARRLPRPAAVTPSESFVTKQDTTGKIISIDTSSLRAAGRTGWEDLVRKCIYA
FFQPQGREPSYAKQLFQEVMTRGTAFSPSYRFTLSDGTVLSAHTKCKLCYPSSPEVQPFIMGIHIIDRDHGMLP
SQENTNSAMALPRVSPSLNPNISPGQGMALPPSIPPSNGSMLATPGNQQPGAGLHSSNSSTSQTSFGCSPGNQI
GANVALSQGQAGPPNSNHNLNLNSSPMNSPGITPPQFMSPRHRVSPGLVPRPRGPSNPFSPTMPTMHSPVGMAN
SGGAATAVAVAGLSEGASTPVGYSTPPPVLRQLGSQSSPGRLGMQPMKAESKDSKEIASILSEMIQSDGGAGDG
KPLDSGVLHAGERLTEGESKCSQSTSHKLVQLLASTAEQQLRHADADTSCKDSLACAGTTSALGASNPPSSTCP
SSHSSLTERHKILHRLLQEGSPSDITTLAMEHDKKDNVPNPATTPTAPSQLPGTPDIKLESDAMKKKDVKDHQL
LRYLLDKDEKELAAAPALSLDDVKVKVEKTEQMEPCNTTPVPLAKPPAAEEVKLESQGQFAAELEQLDQLLPSL
EKAAQLPGLCGPERSESGVAVKPEMLAAPLQPSTAPRAPAGLNRMSELEMGVPDHQFGQPGMGEQIPWTDNSMA
PMNRSTLSKPEDPCITSQLDELLCPPTTPEGRNDEKALLEQLVSFLSGKDETELAELDRALGIDKLVQGGGLEA
LTERFQPQQATPPLMMEQKPGMYPQPYSSASPTTNLPAAFPGMVRQKPSFGPMPVQVPPPRGAFPTTMAMQPRQ
TLSRPPTAPNQLRLQLQQRLQGQQQLIHQNRQAILNQFAANSPVGINMRAGMQQQMTPQPPLNAQMLAQRQREL
YSQQHRQRQLMQQRAILMRQQSFGNNLPPSAGLPVQMGAARLPQAPPQQFPYPPNYGTSPGNPPTSTSPFSQLA
SSPEAALANRGGMVTRGMMGSVGGQFGAGMTPQMQQNIFQYSGSGMGQQNEPAFAPSLSPSSPLMSPQLPPSQS
PMLQPAPPAPGYQSPDMKTWQQGAMGNSNVFSQTGQTQPAPAQQGMYNNMSITVSMAGGNTNVQNMNPMAGQMQ
MNSLQMPGMNSMCSEQVNDPALRPTGLYCNQLSSTDLLKTEADGAQEKKTEEFFSVVAPD
>123Nyctereutes procyonoides|A0A811YFZ7|Aves|SRC1
MSGLGDSSSDPANPDSHKRKGSPCDTLASSTEKRRREQENKYLEELAELLSANISDIDSLSVKPDKCKILKKTV
DQIQLMKRMEQEKSTTDDEVQKSDISSSSQGVIEKESLGPLLLEALDGFFFVVNCEGRIVFVSENVTSYLGYNQ
EELMNTSVYSILHVGDHAEFVKNLLPKSLVNGVPWPQEATRRNSHTFNCRMLIHPPDEPGTENQEACQRYEVMQ
CFTVSQPKSIQEDGEDFQSCLICIARRLPRPPAITGVESFMTKQDTTGKIISIDTSSLRAAGRTGWEDLVRKCI

YAFFQPQGREPSYARQLFQEVMTRGTASSPSYRFILNDGTMLSAHTKCKLCYPQSPDMQPFIMGIHIIDREHSG
LSPQDDTNSGMSIPRVNPSVNSSISPAHGVARSSTLPPSSNNMVSPRINRQQSSDLHSSSHSNSSNSQGSFGCS
PGNQIVASVALNQGQASSQSTNPSLNLNNSPMEGTGISLAQFMSPRRQVSSGLAPRPRMPNNSFPPNIPTLSSP
VSMTGTACNNSNRSYSNIPVTSLQSMNEGPNNSVGFSASSPVLRQMSSQNSPSRLNIQPAKAESKDNKEIASIL
NEMIQSDNSSTDGKPLDSGLLHNNDRLSEGDNKYSQTSHKLVQLLTTTAEQQLRHADIDTSCKEVLSCSGTSNS
ASANSSSGSCPSSHSSLTERHKILHRLLQEGSPSDITTLSVEPDKKDGASTSVSVTGQVQGNSSVKLELDASKK
KESKDHQLLRYLLDKDEKDLRSTPNLSLDDVKVKVEKKEQMDPCTTNPTPMTKPAPEEIKLESQSQFTADLDQF
DQLLPTLEKAAQLPGLCEPERMDGAVTNVTIKSETLPASLQSTTARPTSRLNRLPELELEAIDNQFGQPGTGDQ
IPWANNTVTAVNQNKPEDQCISSQLDELLCPPTTVEGRNDEKALLEQLVSFLSGKDETELAELDRALGIDKLVQ
GGGLDVLSERFPPQQATPPLMMEERPNLYSQPYSSPSPTANLSSPFQGMVRQKPSLGTMPVQVTPPRGAFSPGM
GMQPRQTLNRPPAAPNQLRLQLQQRLQGQQQLIHQNRQAILNQFAANAPVGINMRSGMQQQITPQPPLNAQMLA
QRQRELYSQQHRQRQLIQQQRAMLMRQQSFGNNLPPSSGLPVQMGNPRLPQGAPQQFPYPPSYGTNPGTPPAST
SPFSQLAANPESTLANRNSMVNRGMAGNMGGQFGTGINPQMQQNVFQYPGSGMVPQGEANFAPSLSPGSSMVPM
PIPPPQSSLLQPTPPSSGYQSPDMKAWQPGAMGNNVFSQAVQNQPTPAQPGVYNNMSITVSMAGGNTNVQNMNP
MMGQMQMSSLQMPGMNTVCPEQINDPALRHTGLYCNQLSSTDLLKTETDGTQQVQQVQVFADVQCTVNLVGGDP
YLNQPGPLGTQKPTAGPQTPQAQQKSLLQQLLTE
>124Sula dactylatra|A0A851AU85|Aves|SRC1
MSGLGDSSTDPANPDSRKRKGSPCDTAQSNEKRRREQENKYLEELAELLSANIGDIDTLSVKPDKCKILKKTVD
QIQQMKRLEQEKAADDEVQKSDISSSSQGVIEKESLGPLLLEALDGFFFVVNREGRIVFVSENVTSYLGYNQEE
LMNTSVYSILHVGDHAEFVKNLLPKSLVNGVPWPQEATRRNSHTFSCRMLIRPPDEPGAENQEARQRYEVMQCF
TVSQPKSFKEEGEDFQSCLICIARRLPRPAAVTPSESFVTKQDTTGKIISIDTSSLRAAGRTGWEDLVRKCIYA
FFQPQGREPSYAKQLFQEVMTRGTAFSPSYRFTLSDGTVLSAHTKCKLCYPSSPEMQPFIMGIHVIDRDHGMLS
PQENTNSAMSLPRVSPSLNPSISPGQGMALPPSIPPSNGSMLATNVNQQPGASLHNSNSSTSQTSFGCSPGNQI
GANVALSQGQAGPQNSNHTLNLNSSPMNSPGITPPQFMSPRHRVSPGLVPRPRVPSNPFSPTMPTMHSPVGMAN
SGTGGGGGTGGTRPFPSAPINSMQGLTEGPSTPVGYSTPPPVLRQLSSQSSPGRLAMQPMKAESKDSKEIASIL
SEMIQSDGGSGDGKPLDSGMLHASDRLSEGDSKCSQATSHKLVQLLATTAEQQLRHADADTSCKDSLACAGTAG
TLASSNPPSSTCPSSHSSLTERHKILHRLLQEGSPSDITTLAMEHEKKENAPNPATTPTAQSQLPGTPDIKLEP
DTLKKKDVKDHQLLRYLLDKDEKELAAAPALSLDDVKVKVEKTEQMEPCNTAPVPLAKPPAAEEVKLETQGQFA
AELEQLDQLLPSLEKAAQLPSLCGPERNESGVAGVAVKPEMLAAPLQPGTAARAPTGLNPMNSGQGVQAGRTPF
EFCDPLEPAQLRPVPCPTSNGSSPHARGPSQQGRGTVTAPNPFPPDTGMPELEMGVPDHQFGQPGMGEQIPWTD
NSMSAMNRGTLNKPEDQCITSQLDELLCPPTTPEGRNDEKALLEQLVSFLSGKDETELAELDRALGIDKLVQGG
GLEALTERFQPQQATPPLMMEQKAGMYPQPYSSASPTTNLPGAFPGMVRQKPSFGPMPVQVPPPRGAFPTSMAM
QPRQTLNRPPTAPNQLRLQLQQRLQGQQQLIHQNRQAILNQFAASSPVGINMRAGMQQQITPQPPLNAQMLAQR
QRELYSQQHRQRQLMQQRAILMRQQSFGNNLPPSAGLPVQIGAARLPQAPPQQFPYPPNYGTTPGNPPTSTSPF
SPLASNPEAALANRSSMVNRGMMGGVGGQFGTGMTPQMQQNIFQYSGSGMGQQSEPTFAPSLSPSSPLMSPQLP
PSQSPMLQPAPPTPGYQSPDMKTWQQGAMGNSNVFSQAGQSQPAPAQQGMYNNMSITVSMAGGNTNVQNMNPMA
GQMQMNSLQMPGMNSMCSEQVNDPALRPAGLYCNQLSSTDLLKTEADGAQQVQQVQVFADVQCTVNLVGDPYLN
PPAPLGTPKNAAGPPPPPGQQKSLLQQLL
>125Anhinga anhinga|A0A851Q4K2|Aves|SRC1
MSGLGDSSTDPANPDSRKRKGSPCDTAQSNEKRRREQENKYLEELAELLSANIGDIDTLSVKPDKCKILKKTVD
QIQQMKRLEQEKAADDEVQKSDISSSSQGVIEKESLGPLLLEALDGFFFVVNREGRIVFVSENVTSYLGYNQEE
LMNTSVYSILHVGDHAEFVKNLLPKSLVNGVPWPQEATRRNSHTFSCRMLIRPPDEPGAENQEARQRYEVMQCF
TVSQPKSFKEEGEDFQSCLICIARRLPRPAAVTPSESFVTKQDTTGKIISIDTSSLRAAGRTGWEDLVRKCIYA
FFQPQGREPSYAKQLFQEVMTRGTAFSPSYRFTLSDGTVLSAHTKCKLCYPSSPEMQPFIMGIHVIDRDHGMLS
PQENTNSAMSLPRVSPSLNPSISPGQGMALPPTIPPSNGSMLATNVNQQPGAGLHNSNSSTSQTSFGCSPGNQI
GANVALSQGQAGPQNSNHTLNLNSSPMNSPGITPPQFMSPRHRVSPGLVPRPRVPSNPFSPTMPTMHSPVGMAN
SGTGGGGGTGGTRPFPSAPINSMQGLTEGPSTPVGYSTPPPVLRQLSSQSSPGRLAMQPMKAESKDSKEIASIL
SEMIQSDGGSGDGKPLDSGMLHASDRLSEGDSKCSQATSHKLVQLLATTAEQQLRHADADTSCKDSLACAGTAG
TLASSNPPSSTCPSSHSSLTERHKILHRLLQEGSPSDITTLAMEHEKKENAPNPATTPTAQSQLPGTPDIKLEP
DALKKKDVKDHQLLRYLLDKDEKELAAAPALSLDDVKVKVEKTEQMEPCNTAPVPLAKPPATEEVKLETQGQFA
AELEQLDQLLPSLEKAAQLPSLCGPERNESSVASVAVKPEMLAAPLQPGTAARAPGGLNPMNSGQGVQAGRTPF
EFCDPLEPAQLRPVPCPTSNGSSPHTRGPSQQGRGTVTAPNPFPPDTGMPELEMGVPDHQFGQPGMGEQIPWTD
NSMSAMNRGALNKPEDQCITSQLDELLCPPTTPEGRNDEKALLEQLVSFLSGKDETELAELDRALGIDKLVQGG
GLEALTERFQPQQATPPLMMEQKPGMYPQPYSSASPTTNLPGAFPGMVRQKPSFGPMPVQVPPPRGAFPTSMAM

QPRQTLNRPPTAPNQLRLQLQQRLQGQQQLIHQNRQAILNQFAANSPVGINMRAGMQQQITPQPPLNAQMLAQR
QRELYSQQHRQRQLMQQRAILMRQQSFGNNLPPSAGLPVQIGAARLPQAPPQQFPYPPNYGTTPGNPPTSTSPF
SPLASNPEAALANRSSMVNRGMMGGVGGQFGTGMTPQMQQNIFQYSGSGMGQQSEPTFAPSLSPSSPLMSPQLP
PSQSPMLQPAPPTPGYQSPDMKTWQQGAMGNTNVFSQAGQSQPAPAQQGMYNNMSITVSMAGGNTNVQNMNPMA
GQMQMNSLQMPGMNSMCSEQVNDPALRPAGLYCNQLSSTDLLKTEADGAQQVQQVQVFADVQCTVNLVGDPYLN
PPAPLGTPKNAA
>126Elachura formosa|A0A851TYF3|Aves|SRC1
CMVKMSGLGENSLDSLTNESRKRKPLPCDTLGTSLVCSGEKRRREQESKYIEELAELISANLSDIDNFNVKPDK
CVILKETVRQIRQIKEQGKAVSNDDDVQKADVSSTGQGVIDKDSLGPLLLQAALDGFLFVVHRDGNIVFVSENV
THYLQYKQEDLVNTSVYGILHEEDRKDFLKNLPKSSANGVAWTNETPRQKSHTFNCRMMVKSSHDLFEDVGGYQ
DIRQRYETMQCFALSQPKAMMEEGEDLQSCMICVARRITTAERVFPGNPESFITRHDLSGKLINIDTNSLRSSM
RPGFEDTIRRCIQKFLYHNDGQSWSNKRHYHEAYTQGHAETPLYRFSLADGTIVTAQTKSKLFRNPVTNDRHGF
VSTHFLQREQNGYRPNPNSVGQNIRTPVTGNTNSVNGLMNMSPGQAMPMQNRSYGMGDPNAVGQLTSTRYGSPG
SMGPVNSGLGMQSSAYQCNGYGLNISSPPHGSPGLTSNQQNLMVSPRNRASPKMSSHQYSPVTGVNSPMGSASN
TSNSTFSNSSLSALQAISEGVGNSLLSTLSSPGTKLDNSPNISIAQQNKTSTQDSKSPSGLYCEQSQVDSSICQ
SNNRDLLSEKDSKEGNLEASESQRGPSESKGHKKLLQLLTCSSDERGHSTASNSPLDSNCKESSASITSPSGVS
SSTSGGVSSSSNMHGSLLQEKHRILHKLLQNGNSPAEVAKITAEATGKDTYHDAGNTASCGEGTVKQEQLSPKK
KENNALLRYLLDKDDIKDPLSKELKPKVEGVDNKMGQCTSSTVPTSSQEKDMKIKMEPAEEMNGDLDNLDAILG
DLTSSEFYNNAMSANGNSPGTKKALFQGNALMGVKSPQSVQTTCPPFNRALSLDSPMGSGASVRNVNVFSMLQK
PNLMGESPRMVENQENFGANLGFTPKRNVVLSQHQSGDWGLPNSKMNRLESASSGSVLRSGPELSTALLRPPEG
GSVSGLPVRSNSIPGTRPMLQQQMIHMRPNEINIDIGGNAYGQPGPSNQPGLWPDNMMSMEQVSRGSQNRQLVR
NSLDDLLCSAPNSESQNDERALLDQLHTLLSNTDVTGLEEIDRALGIPDLVNQGQSLDPKQDSFQGQESSVMVD
QKPLLYGQPYQGQGAAMPGGFNNIQAQQPSFNSVMNQMSQPSNFPLQSMHPRANVMRPRTSTPKQLRMQLQQRL
QGQQFMNQTRQTLEIKMENPVSGTNAVMRPVMQPQVGSQQQGFLNAQMVAQRNRELISHHFRQQRMAMMMQQQQ
QPQAFSSPPNVTASGSMDSPLTGPPMAQVPSQQFPYPPNYGISQQPDPSFSRVSSPPNPMISRIGAGQNPMLQH
PQSATVYQSSEMKGWPSGSMPRNSSFFQQQISHQGSPAAYSMMHMNGSSGHIGQMSINTMSMSGLPMGPDPVSC
>127Eolophus roseicapilla|A0A851XTB1|Aves|SRC1
MSGLGDSSTDPANPDSRKRKGSPCDTAQSNEKRRREQENKYLEELAELLSANIGDIDTLSVKPDKCKILKKTVD
QIQQMKRLEQGKATTPALACQKPSDLFPLQALDGFFFVVNREGRIVFVSENVTSYLGYNQEELMNTSVYSILHV
GDHAEFVKNLLPKSLVNGVPWPQETTRRSSHTFSCRMLVRPPDDTGVENQEARQRYEVMQCFTVSQPKSFKEEG
EDFQSCLICIARRLPRPAAVAPSESFVTKQDTTGKIISIDTSSLRAAGRTGWEDLVRKCIYAFFQPQGREPSYA
KQLFQEVITRGTAFSPSYRFTLSDGTVLSAHTKCKLCYPSSPEMQPFIMGIHVIDRDHGMLSPQENTNSAMSLP
RVSPSLNPSISPGQGMALPPSIPPSNGSMLATNVNQQPGAGLHNSNSSTSQTSFGCSPGNQIGANVALSQGQAG
PQNSNHTLNLNSSPMNSPGITPPQFMSPRHRVSPGLVPRPRVPSNPFSPTMPTMHSPVGMANSGSGGSCGTGGN
RPFPSAPINSMQGLTEGPSPPVGYSTPPPVLRQLSSQSSPGRLAMQPMKAESKDSKEIASILSEMIQSDSGSGD
GKPLDSGVLHTGDRLSEGDSKCSQATSHKLVQLLATTAEQQLRHADADTSCKDSLVCAGTGAALASSNPPSSTC
PSSHSSLTERHKILHRLLQEGSPSDLTSLAMEHEKKESTSNPTTTPTAHNQLPGNPDIKLEPDTLKKKDVKDHQ
LLRYLLDKDEKELAAAPALSLDDVKVKVEKTEQMEPCNTAPVPLAKPPAAEEVKLETQGQFAAELEQLDQLLPS
LEKAAQLPGLCGPERNESSVAGVAVKPEMLAAPLQPSTAARAPPGLNPINSGQSVQPGRTPFEFCDPMEPAQMR
PVPCPASNGTSPHTRGPAQQGRGTVTAPNPFPADTGMPELEMGVPDHQFGQPGMGEQMPWTDNSMAAMNRAALN
KPEDQCITSQLDELLCPPTTPEGRNDEKALLEQLVSFLSGKDETELAELDRALGIDKLVQGGGLEALTERFQPQ
QATPPLMMEQKPGMYPQPYSSASPTTNLPGAFPGMVRQKPSFGPMPVQVPPPRGAFPTNMAMQPRQTLNRPPTA
PNQLRLQLQQRLQGQQQLIHQNRQAILNQFAANSPVGINMRAGMQQQITPQPPLNAQMLAQRQRELYSQQHRQR
QLMQQRAILMRQQSFGNNLPPSAGLPVQIGAARLPQAPPQQFPYPPNYGTTPGNPPTSTSPFSPLASNPEAAAL
ANRSSMVNRGMMGGVGGQFGAGMTPQMQQNIFQYSGSGMGQQSEPGFAPSLSPSSPLMSPQLPPSQSPMLQPAP
PTPGYQSPDMKTWQQGAMGNSNVFSQAGQSQPAPAQQGMYNNMSITVSMAGGNTNVQNMNPMAGQMQMNSLQMP
GMNSMCSEQVNDPALRPTGLYCNQLSSTDLLKTEADGAQQVQQVQVFADVQCTVNLVGDPYLNPPASLGTPKNA
AGPPPPPGQQKSLLQQLLTE

>128Ramphastos sulfuratus|A0A852CHI7|Aves|SRC1
MSGLGDSSTDPANPDSRKRKGSPCDTAQSNEKRRREQENKYLEELAELLSANIGDIDTLSVKPDKCKILKKTVD
QIQQMKRLEQEKTADDEVQKSDISSSSQGVIEKESLGPLLLEALDGFFFVVNREGRIVFVSENVTSYLGYNQEE
LMNTSVYSILHVGDHAEFVKNLLPKSLVNGVPWPQEATRRNSHTFSCRMLIRPPDEPGAENQEARQRYEVMQCF
TVSQPKSFKEEGEDFQSCLICIARRLPRPAAVTPSESFVTKQDTTGKIISIDTSSLRAAGRTGWEDLVRKCIYA

FFQPQGREPSYAKQLFQEVMTRGTAFSPSYRFTLSDGTVLSAHTKCKLCYPSSPEMQPFIMGIHVIDRDHGMLS
PQENTNSTMSLPRVSPSLNPSISPGQGMALPPSIPPSNGSMLATNVNQQPGAGLHNSNSSTSQTSFGCSPGNQI
GASVALSQGQAGPQNSNHTLNLNSSPMNSPGITPPQFMSPRHRVSPGLVPRPRVPSNPFSPTMPTMHSPVGMAN
SGSGGGCGTGGTRPFPSAPINSMQGLTEGPSTPVGYSTPPPVLRQLSSQSSPGRLAMQPIKAESKDSKEIASIL
SEMIQSDGASGDGKPLDSGMLHTSDRLAEGETKCPQTTSHKLVQLLASTAEQQLRHADVDTSCKDSLACASTTG
PLASGNPPSSSCPSSHSSLTERHKILHRLLQEGSPSDITTLGMEHEKKESAPNPTPTAQSQLPGTPDMKLEPEA
LKKKDAKDHQLLRYLLDKDEKELAAAPALSLDDVKVKVEKTEQMEPCNTAPVPLPKPPATEEVKLETQGQFAAE
LEQLDQLLPSLEKAAQLPGLCGPERNESGVAGVSVKPEMLAAPLQPSAAARAPTGLNPMSSGQGVQAARAPFEF
CDTLEPAQLRQVPCPGSNGSSPHARGPAQQGRGTVTAPNPFPPDTGMPELEMGVPDHQFGQPGMGEQIPWTDSS
MAAMNRGTLNKPEDQCITSQLDELLCPPTTPEGRNDEKALLEQLVSFLSGKDETELAELDRALGIDKLVQGGGL
EALTERFQPQQSTPPLMMEQKPGMYPQPYSSASPTTNLPGAFPGMVRQKPSFGAMPVQVPPPRGAFPTNMAMQP
RQTLSRPPTAPNQLRLQLQQRLQGQQQLIHQNRQAILNQFAANSPVGINMRAGMQQQITPQPPLNAQMLAQRQR
ELYSQQHRQRQLMQQRAILMRQQSFGSNLPPSSGLPVQIGAARLPQAPPQQFPYPPNYGTSPGNPPTSTSPFSP
LASNPEAALGNRSGMVNRGMMGSVAGQFGAGMAPQMQQNIFPYSGSGMGQQSEPSFAPALSPSSPLMSPQLPPS
QSPMLQPAPPTAGYQSPDMKSWQQGAMGSSNVFNQAGQGQVAPAQQGMYNNMSITVSMAGGNTNVQNMNPMAGP
MQMNSLQMPGMNSMCSEQVNDPALRPAGLYCNQLSSTDLLKTEADGAQQVQQVQVFADVQCTVNLVGDPYLNPP
APLGTPKNAAGPPPPPGQQKSLLQQLLTE
>129Larus smithsonianus|A0A852GFG5|Aves|SRC1
MSGLGDSSTDPANPDSRKRKGSPCDTAQSNEKRRREQENKYLEELAELLSANIGDIDTLSVKPDKCKILKKTVD
QIQQMKRLEQEKAADDEVQKSDISSSSQGVIEKESLGPLLLEALDGFFFVVNREGRIVFVSENVTGYLGYNQEE
LMNTSVYSILHVGDHAEFVKNLLPKSLVNGVPWPQEATRRNSHTFSCRMLIRPPDEPGAENQEARQRYEVMQCF
TVSQPKSFKEEGEDFQSCLICIARRLPRPAAVTPSESFVTKQDTTGKIISIDTSSLRAAGRTGWEDLVRKCIYA
FFQPQGREPSYAKQLFQEVMTRGTAFSPSYRFTLSDGTVLSAHTKCKLCYPSSPEMQPFIMGIHVIDRDHGMLS
PQENTNSAMSLPRVSPSLNPSISPGQGMALPPSIPPSNGSMLATNVNQQPGAGLHNSNSSTSQTSFGCSPGNQI
GANVALSQGQAGPQNSNHTLNLNSSPMNSPGITPPQFMSPRHRVSPGLVPRPRVPSNPFSPTMPTMHSPVGMAN
SGSGGGGGTGGARPFPSAPINSMQGLTEGPSTPVGYSTPPPVLRQLSSQSSPGRLAMQPMKAESKDSKEIASIL
SEMIQSDGGSGDGKPLDSGMLHAGDRLSEGDSKCSQATSHKLVQLLATTAEQQLRHADADTSCKDSLACAGTAG
TLASSNPPSSTCPSSHSSLTERHKILHRLLQEGSPSDITTLAMEHEKKENAPNPATTPTAQSQLPGTPDIKLEP
DALKKKDVKDHQLLRYLLDKDEKELAAAPALSLDDVKVKVEKTEQMEPCNTAPVPLAKPPAAEEVKLETQGQFA
AELEQLDQLLPSLEKAAQMPGLCGPERSESGMAGVTVKPEMLAAPLQPSTAARAPTGLNPMPSGQGVQAGRPPF
EFCDPLEPAQLRPVPCPASNGSSPHARGPSQQGRGTVTAPNPFPPDTGMPELEMGVPDHQFGQPGMGEQIPWTD
NSMAAMNRGALNKPEDQCITSQLDELLCPPTTPEGRNDEKALLEQLVSFLSGKDETELAELDRALGIDKLVQGG
GLEALTERFQPQQATPPLMMDQKPGMYPQPYSSASPTTNLPGAFPGMVRQKPSFGPMPVQVPPPRGAFPTNMAM
QPRQTLNRPPTAPNQLRLQLQQRLQGQQQLIHQNRQAILNQFAANSPVGINMRAGMQQQITPQPPLNAQMLAQR
QRELYSQQHRQRQLMQQRAILMRQQSFGNNLPPSAGLPVQIGAARLPQAPPQQFPYPPNYGTNPGNPPTSTSPF
SPLASNPEAALANRSSMVNRGMMGGVGGQFGAGMTSQMQQNIFQYSGSGMGQQSEPAFAPSLSPSSPLMSPQLP
PSQSPMLQPAPPTPGYQSPDMKTWQQGAMGNSNVFSQAGQSQPAPAQQGMYNNMSITVSMAGGNTNVQNMNPMA
GQMQMNSLQMPGMNSMCSEQVNDPALRPAGLYCNQLSSTDLLKTEADGAQQVQQVQVFADVQCTVNLVGDPYLN
PPAPLGTPKNAAGPPPPPGQQKSLLQQLLTE

>130Pteruthius melanotis|A0A852MXW4|Aves|SRC1
CMAKMSGLGENSLDSLTNESRKRKPLPCDTPGASLVCSGEKRRREQESKYIEELAELISANLSDIDNFNVKPDK
CAILKETVRQIRQIKEQGKAVSNDDDVQKADVSSTGQGVIDKDSLGPLLLQAALDGFLFVVNRDGNIVFVSENV
TQYLQYKQEDLVNTSVYGILHEEDRKDFLKNLPKSSVNGVAWTNETPRQKSHTFNCRMVVKSCRDLLEDVGGYQ
DIRQRYETMQCFALSQPRAMMEEGEDLQSCMICVARRIATAERAFPTNPESFITRHDLSGKLVNIDTNSLRSSM
RPGFEDIIRRCIQRFLCHNDGQSWSNKRHYHEAYTQGHAETPLYRFSLADGTIVTAQTKSKLFRNPVTNDRHGF
VSTHFLQREQSGYRPNPNSVGQNIRTPVPGNTNSVGGLMNMSPGQAMPMQNRNYGMGDPNAVGQLTSTRYGGPG
NMGPVNSGPGVQSSAYQNTSYGLNISSPPHGSPGLTSNQQNLMVSPRNRGSPKMSSHQYSPVTGMHSPMGSASN
TSNSAFSSSSLSALQAISEGVGNSLLSTLSSPGPKLDNSPNISITQQSKTSTQDSKSPSGLYCEQNQVESSICQ
SNSRDLLSEKGSKEGNLEASENQRGPSESKGHKKLLQLLTCSSDERGHSTPSNSPLDSNCKESSTGVTSPSGVS
SSTSGGVSSSSNMHGSLLQEKHRILHKLLQNGNSPAEVAKITAEATGKDTYHDASNTASCGEGTVKQEQLSPKK
KENNALLRYLLDKDDIKDPLSKELKPKVESVDSKMGPCTSSTIPTSSQEKEMKIKMEPAEEMNGDLENLDAILG
DLTSSEFYNNAMSANGNNLGTKQALFQGNVLLGVKSPQSVQTTRPPFNRALSLDSPVGSGASVRNVNAFSVLQK
QNLMGGSPRMIENQENFGANLGFAPNRNVMNQHQSGDWGLPNSKMNRLESASSASVLRPGPEFSTTLPRPPEGG

SVSGLPGRSNSIPGTRPMLQQQMIHMRPNEINIDMGGNPYGQPGPSNQPGLWPDNMMSMEQASRGSQNRQLVRS
SLDDLLCSTPNTEGQNDERALLDQLHTLLSNTDVTGLEEIDRALGIPDLVNQGQALEPKQDSFQGQESSVMIDQ
KPLLYGQPYQGQGAAMPGGFSSIQGQQPSFNSVMNQMSQPSNFPLQSTHPRANVMRPRTNTPKQLRMQLQQSLQ
GQQFMNQTRQTLEIKMENPVSGTNSVMRPVMQPQVGSQQQGFLNAQMVAQRNRELISHHFRQQRMAMMMQQQQQ
PQAFSPPPNVTASGSMDSALTGPPMAQVPSQQFPYPPNYGISQQPDPAFSRVPSPSNPMISSRIGPSQNPMLQH
PQSATMYQSSEMKGWPSGSMPRNSSFPQQQFTHQGNPAAYSMMHMNGSSGHIGQMSINTMPMSGMSMGPDQVSC
>131Atrichornis clamosus|A0A852NMI0|Aves|SRC1
CMVKMSGLGENSLDSLTNESRKRKPLPCDTPGASLACSGEKRRREQESKYIEELAELISANLSDIDNFNVKPDK
CAILKETVRQIRQIKEQGKAVSNDDDVQKADVSSTGQGVIDKDSLGPLLLQAALDGFLFVVNRDGNIVFVSENV
TQYLQYKQEDLVNTSVYGILHEEDRKDFLKNLPKSSVNGVAWTNETPRQKSHTFNCRMMVKSSHDLLEDVGGHQ
DVRQRHETMQCFALSQPRAMMEEGEDLQSCMICVARRITTAERVFPTNPESFITRHDLSGKLVNIDTNSLRSSM
RPGFEDIIRRCIQRFLCHNDGQSWSNKHHYHEAYTQGHAETPLYRFSLADGTIVTAQTKSKVFRNPVTNDRHGF
VSTHFLQREQNGYRPNPNSVGQNVRTPVTGNTVSVGGLMNIPPGQSMPMQNRNYGMGDPNAVGQLTSTRYGGPG
NMGPVNSGPGMQSSAYQNSSYGLNISSPPHGSPGLTSNQQNLMISPRNRGSPKMSSHQYSPVTGMHSPMGSASN
TSNSTFSSSSLSALQAISEGVGNSLLSTLSSPGPKLDNSPNISITQQSKTSTQDCKSPGLYCEQSQAESSICQS
NSRDLLSEKDSKEGNVEASESQRGPSESKGHKKLLQLLTCSSDERGHSTASNSPLDSNCKESSTSVTSPSGVSS
STSGGVSSSSNVHGSLLQEKHRILHKLLQNGNSPAEVAKITAEATGKDTYHDASNTASCGEGTVKQEQLSPKKK
ENNALLRYLLDKDDIKDPLSKELKPKVESVDNKMGQCTSSTIPTSSQEKQMKIKMEPSGEMNGDLDNLDAILGD
LTSSEFYNNPMSANGNNLGTKQALFQGNALLGVKSPQSVQTTRPPFSRALSLDSPVGSGASVRNVSAFSMLQKQ
NLMGGSARIIENQENFGADTGFTPNRNVVMNHHQSGDWGLPNSKVNRLESTSSASMLRPGPEFSTTLPRPTGGS
SMSGLPVRSNSIPGTRPMLQQQMIHMRPNEINIGMGGNPYGQSGPSNQPGLWPDNMLSMEQASRGSQNRQLVRN
SLDDLLCSAPNMEGQTDERALLDQLHTLLSNTDVTGLEEIDRALGIPDLVNQGQALEPKQDSLQGQESSVMIDQ
KSLLYGQPYQGQGAAMPGSFSNIQGQQPSFNSVMNHMSQPSNFPLQSMHPRANVMRPRTNTPKQLRMQLQQRLQ
GQQFMNQTRQTLDMKMENPVSGNTSVMRPVMQPQGSQQQGFLNAQMVAQRNRELISHHFRQQRMAMMMQQQQQP
QAFSPPPNVTASGSMDSALTGSAVAQVPPQQFPYPPNYGISQQPDPAFSRVSSPPNTMISRIGPSQNPMLQHPQ
SPTVYQSSEMKGWPSGSTPRNSSFPQQQFSHQGSPSAYSMMHMNGSSGHIGQMSINTMPMSGMPMGPDQVSC
>132Homo sapiens|A8K1V4|Aves|SRC1
MSGLGDSSSDPANPDSHKRKGSPCDTLASSTEKRRREQENKYLEELAELLSANISDIDSLSVKPDKCKILKKTV
DQIQLMKRMEQEKSTTDDDVQKSDISSSSQGVIEKESLGPLLLEALDGFFFVVNCEGRIVFVSENVTSYLGYNQ
EELMNTSVYSILHVGDHAEFVKNLLPKSLVNGVPWPQEATRRNSHTFNCRMLIHPPDEPGTENQEACQRYEVMQ
CFTVSQPKSIQEDGEDFQSCLICIARRLPRPPAITGVESFMTKQDTTGKIISIDTSSLRAAGRTGWEDLVRKCI
YAFFQPQGREPSYARQLFQEVMTRGTASSPSYRFILNDGTMLSAHTKCKLCYPQSPDMQPFIMGIHIIDREHSG
LSPQDDTNSGMSIPRVNPSVNPSISPAHGVARSSTLPPSNSNMVSTRINRQQSSDLHSSSHSNSSNSQGSFGCS
PGSQIVANVALNQGQASSQSSNPSLNLNNSPMEGTGISLAQFMSPRRQVTSGLATRPRMPNNSFPPNISTLSSP
VGMTSSACNNNNRSYSNIPVTSLQGMNEGPNNSVGFSASSPVLRQMSSQNSPSRLNIQPAKAESKDNKEIASIL
NEMIQSDNSSSDGKPLDSGLLHNNDRLSDGDSKYSQTSHKLVQLLTTTAEQQLRHADIDTSCKDVLSCTGTSNS
ASANSSGGSCPSSHSSLTERHKILHRLLQEGSPSDITTLSVEPDKKDSASTSVSVTGQVQGNSSIKLELDASKK
KESKDHQLLRYLLDKDEKDLRSTPNLSLDDVKVKVEKKEQMDPCNTNPTPMTKPTPEEIKLEAQSQFTADLDQF
DQLLPTLEKAAQLPGLCETDRMDGAVTSVTIKSEILPASLQSATARPTSRLNRLPELELEAIDNQFGQPGTGDQ
IPWTNNTVTAINQSKSEDQCISSQLDELLCPPTTVEGRNDEKALLEQLVSFLSGKDETELAELDRALGIDKLVQ
GGGLDVLSERFPPQQATPPLIMEERPNLYSQPYSSPSPTANLPSPFQGMVRQKPSLGTMPVQVTPPRGAFSPGM
GMQPRQTLNRPPAAPNQLRLQLQQRLQGQQQLIHQNRQAILNQFAATAPVGINMRSGMQQQITPQPPLNAQMLA
QRQRELYSQQHRQRQLIQQQRAMLMRQQSFGNNLPPSSGLPVQMGNPRLPQGAPQQFPYPPNYGTNPGTPPAST
SPFSQLAANPEASLANRNSMVSRGMTGNIGGQFGTGINPQMQQNVFQYPGAGMVPQGEANFAPSLSPGSSMVPM
PIPPPQSSLLQQTPPASGYQSPDMKAWQQGAIGNNNVFSQAVQNQPTPAQPGVYNNMSITVSMAGGNTNVQNMN
PMMAQMQMSSLQMPGVNTVCPEQINDPALRHTGLYCNQLSSTDLLKTEADGTQDKKTEEFFSVVTTD
>133Sus scrofa|B0FRD4|Aves|SRC1
MSGLGDSSSDPANPDSHKRKGSPCDTLASSTEKRRREQENKYLEELAELLSANISDIDSLSVKPDKCKILKKTV
DQIQLMKRMEQEKSTTDDEVQKSDISSSSQGVIEKESLGPLLLEALDGFFFVVNCEGRIVFVSENVTSYLGYNQ
EELMNTSVYSILHVGDHAEFVKNLLPKSLVNGVPWPQEATRRNSHTFNCRMLIHPPDEPGTENQEACQRYEVMQ
CFTVSQPKSIQEDGEDFQSCLICIARRLPRPPAIMGVESFMTKQDTTGKIISIDTSSLRAAGRTGWEEFMRKCI
YAFFQPQGREPSYARQLFQEVMTRGTASSPSYRFILNDGTMLSAHTKCKLCYPQSPDMQPFIMGIHIIDREHSG
LSPQDDTNSGMSIPRVNPPVNPSISPAHGVARSSSLPPSNSNMVSTRVNRQQSSDLHSSSHSNSSNSQGSFGCS
PGNQIVAGVALNQGQASSQSTNPPLNLNNSPMEGTGISLAQFMSPRRQVSSGLATRPRMPNNSFPPNIPTLNSP

VSMTSTACNNSNRSYSNIPVTSLQSMNEGPNNSVGFSAGSPVLRQMSSQNSPSRLNIQPAKAESKDNKETASIL
NEMIQSDNSSNDGKPLDSGLLHNNDRLSDGDNKYSQTSHKLVQLLTTTAEQQLRHADIDTSCKEVLSCTGTSSS
ASANSSSGSCPSSHSSLTERHKILHRLLQEGSPSDITTLSVEPDKKDSASTSVSVTGQVPGNSGIKLELDASKK
KESKDHQLLRYLLDKDEKDLRSTPNLSLDDVKVKVEKKEQMDPCNTNPTPMTKPPPEEIKLESQSQFTADLDQF
DQLLPTLEKAAQLPGLCETERMDGAVTSVTIKSEILPATLQSTTARPTSRLNRLPELELEAIDNQFGQPGTGDQ
IPWANNTVTAVNQNKPEDQCISSQLDELLCPPTTVEGRNDEKALLEQLVSFLSGKDETELAELDRALGIDKLVQ
GGGLDVLSERFPPQQATPPLMMEERPNLYSQPYSSPSPTANLSGPFQGMVRQKPSLGTMPVQVTPPRGAFSPGM
GMQPRQTLNRPPAAPNQLRLQLQQRLQGQQQLIHQNRQAILNQFAANAPVGINMRSGMQQQITPQPPLNAQMLA
QRQRELYSQQHRRRQLIQQQRAMLMRQQSFGNNLPPSSGLPVPMGNPRLPQGAPQQFPYPPNYGTNPGTPPAST
SPFSQLAENPEATLGNRNSMVNRGMTGNMGGQFGTGINPQMQQNVFQYPGSGMVPQGEANFAPSLSPGSSMVPM
PIPPPQSSLLQQTPPASGYQSPDMKAWQQGAMGNNNVFSQAVQNQPTPAQPGVYNNMSITVSMAGGNTNVQNMN
PMMGQMQMSSLQMPGMNTVCPEQINDPALRHTGLYCNQLSSTDLLKTEADGTQQVQQVQVFADVQCTVNLVGGD
PYLNQPGPLGTQKPTAGPQTPQAQQKSLLQQLLTE
>134Dicentrarchus labrax|E6ZIN5|Actinopteri|SRC1
MSGVGENSSDPARAESRKRKECSAELLGPSPKRSNEKRNREHENKYIEELAELIFANFNDIDNFNVKPDKCAIL
KETVKQIRQIKEQEKAAAANEEEVQKADVSSTGQSVIDKDALGPMMLEALDGFFFVVNMEGNIVFVSENVTQYL
RYNQEELMNTSVYSVLHVGDHAEFIKNLLPKSLVNGVPWSSEGPRRNSHTFNCRMLVNPHSESQDEPHEHEPQQ
QKYETMQCFAVSEPKSIKEEGDDFQSCLICVARRVPMKERPMLPAHESFTTRQDLQGKITSLDTSLLRASMKPG
WEDLVRRCIQRFHLQNDGEMSFAKRHQQEVLRHGQAFSPIYRFSLSDGTIVSAHTKSKLVRSPATNEPQLYMSL
HILQREQTVCGMGQDMGPGSQATGMAPKPMNSSTPSMTPPTPGSLPGSGPQGQDTTISSNSTPFSPPGPAGPRE
PAAMGGHMQGYRYGCPPPHNHTGGMQPPQQGPNWRMNSPSRASPSPAGGPHPPQQNSMLSPRHRGSPGGVGSPR
VAGGLHSPSPVGMCGGGPGSAGSSSTTSTHGNSYTSSSLSALQALSECHGVGHGHGPPHAHTLGSPDRKIGSPA
GVTQGSGVNTHMMSKLGGGSGTTGGTGDSFGPHTEVGQGHPQGQTENNLAEPKEEREGPSEGHDAHNRLHDNKG
HTKLLQLLTTKPEPLETPLSPGAGGEGKDQAGTGVRGGHTGGNTHATSLKEKHKILHQLLQNSTSPVDLAKLTA
EATGKELEQAQGAGSTASGAEIAPKQEPLSPKKKDNALLRYLLDKDDTVLKDKVPKLEPGEVKVEGGKHPLVKA
EKQDAGYDRAEQSSELDDILNDLQNAQPQLFCEPRPVSLPSPMDKQNIINDILQMSETNPAMAAQQQHRGIGTG
MGPTNFGGVRLSQPGRGLPVRSVSLDMNVSPQQSSLQYPIRATSPYTLIQQQQQQGIVGSHGMMANQAGMVSTG
LMAPGGPRPSMQQQEGWVGSASAPPLGASGTGQQGAMPGRMGPNSAPMRPNSQPGPRQMLQSPMMANAQPDMDI
GIAGHHFSQQQAPPNQTAPWPDSMMPIDQTAFVNTTRPVHPAAQDDLLCSTGLGSGEGSAVDEGALMSQLYSAL
KDFDGLEEIDRALGIPALVEQNQSLEPEQFPQDPSMMMEQKPPMYTQQFPSHMAQRGYPGAAMQEQGFHPMAGQ
IGPRPGYPMMRMQARPGLRPAGVVPNQPNTLRLQLQHRLQSQQNRQPMMTQMSSVSNVNLPLRASAPNQGTINA
QMLAQRQRELLSNHLRQRQQQQQQAQQVQQQRSMAMRAQGLNLPPNMAAGGMSNPRIPQANPQQFPYPPSYGTS
TGLASPPPPTSPFSSPLSPSLPSLPPNPQLHLHGTSSSSSTSSSSSQMMMGNTNMMGGQYGAVLSPQMQHSAFQ
FPNSGMSQQADGGFGGPGTPQSPLLSPRMAHTQSPMMQQGQGNAAFQGSPDMNGWPQGNMGGNSMFSQQQASPQ
FTQQNNSNMYNGNGMNLNNVSMAPNMATSSMSSMGQMAGQMSVTSMASGPSPGLPSMGQEQVNTRPILTQ
>135Sus scrofa|F1SDJ9|Mammalia|SRC1
MSGLGDSSSDPANPDSHKRKGSPCDTLASSTEKRRREQENKYLEELAELLSANISDIDSL
SVKPDKCKILKKTVDQIQLMKRMEQEKSTTDDEVQKSDISSSSQGVIEKESLGPLLLEAL
DGFFFVVNCEGRIVFVSENVTSYLGYNQEELMNTSVYSILHVGDHAEFVKNLLPKSLVNG
VPWPQEATRRNSHTFNCRMLIHPPDEPGTENQEACQRYEVMQCFTVSQPKSIQEDGEDFQ
SCLICIARRLPRPPAIMGVESFMTKQDTTGKIISIDTSSLRAAGRTGWEDLVRKCIYAFF
QPQGREPSYARQLFQEVMTRGTASSPSYRFILNDGTMLSAHTKCKLCYPQSPDMQPFIMG
IHIIDREHSGLSPQDDTNSGMSIPRVNPPVNPSISPAHGVARSSSLPPSNSNMVSTRVNR
QQSSDLHSSSHSNSSNSQGSFGCSPGNQIVAGVALNQGQASSQSTNPPLNLNNSPMEGTG
ISLAQFMSPRRQVSSGLATRPRMPNNSFPPNIPTLNSPVSMTSTACNNSNRSYSNIPVTS
LQSMNEGPNNSVGFSAGSPVLRQMSSQNSPSRLNIQPAKAESKDNKEIASILNEMIQSDN
SSNDGKPLDSGLLHNNDRLSDGDNKYSQTSHKLVQLLTTTAEQQLRHADIDTSCKEVLSC
TGTSSSASANSSSGSCPSSHSSLTERHKILHRLLQEGSPSDITTLSVEPDKKDSASTSVS
VTGQVPGNSGIKLELDASKKKESKDHQLLRYLLDKDEKDLRSTPNLSLDDVKVKVEKKEQ
MDPCNTNPTPMTKPPPEEIKLESQSQFTADLDQFDQLLPTLEKAAQLPGLCETERMDGAV
TSVTIKSEILPATLQSTTARPTSRLNRLPELELEAIDNQFGQPGTGDQIPWANNTVTAVN
QNKPEDQCISSQLDELLCPPTTVEGRNDEKALLEQLVSFLSGKDETELAELDRALGIDKL
VQGGGLDVLSERFPPQQATPPLMMEERPNLYSQPYSSPSPTANLSGPFQGMVRQKPSLGT
MPVQVTPPRGAFSPGMGMQPRQTLNRPPAAPNQLRLQLQQRLQGQQQLIHQNRQAILNQF

AANAPVGINMRSGMQQQITPQPPLNAQMLAQRQRELYSQQHRQRQLIQQQRAMLMRQQSF
GNNLPPSSGLPVPMGNPRLPQGAPQQFPYPPNYGTNPGTPPASTSPFSQLAGNPEATLGN
RNSMVNRGMTGNMGGQFGTGINPQMQQNVFQYPGSGMVPQGEANFAPSLSPGSSMVPMPI
PPPQSSLLQQTPPASGYQSPDMKAWQQGAMGNNNVFSQAVQNQPTPAQPGVYNNMSITVS
MAGGNTNVQNMNPMMGQMQMSSLQMPGMNTVCPEQQVQQVQVFADVQCTVNLVGGDPYLN
QPGPLGTQKPTAGPQTPQAQQKSLLQQLLTE
>136Sus scrofa|F1SDK1|Mammalia|SRC1
MSGLGDSSSDPANPDSHKRKGSPCDTLASSTEKRRREQENKYLEELAELLSANISDIDSL
SVKPDKCKILKKTVDQIQLMKRMEQEKSTTDDEVQKSDISSSSQGVIEKESLGPLLLEAL
DGFFFVVNCEGRIVFVSENVTSYLGYNQEELMNTSVYSILHVGDHAEFVKNLLPKSLVNG
VPWPQEATRRNSHTFNCRMLIHPPDEPGTENQEACQRYEVMQCFTVSQPKSIQEDGEDFQ
SCLICIARRLPRPPAIMGVESFMTKQDTTGKIISIDTSSLRAAGRTGWEDLVRKCIYAFF
QPQGREPSYARQLFQEVMTRGTASSPSYRFILNDGTMLSAHTKCKLCYPQSPDMQPFIMG
IHIIDREHSGLSPQDDTNSGMSIPRVNPPVNPSISPAHGVARSSSLPPSNSNMVSTRVNR
QQSSDLHSSSHSNSSNSQGSFGCSPGNQIVAGVALNQGQASSQSTNPPLNLNNSPMEGTG
ISLAQFMSPRRQVSSGLATRPRMPNNSFPPNIPTLNSPVSMTSTACNNSNRSYSNIPVTS
LQSMNEGPNNSVGFSAGSPVLRQMSSQNSPSRLNIQPAKAESKDNKEIASILNEMIQSDN
SSNDGKPLDSGLLHNNDRLSDGDNKYSQTSHKLVQLLTTTAEQQLRHADIDTSCKEVLSC
TGTSSSASANSSSGSCPSSHSSLTERHKILHRLLQEGSPSDITTLSVEPDKKDSASTSVS
VTGQVPGNSGIKLELDASKKKESKDHQLLRYLLDKDEKDLRSTPNLSLDDVKVKVEKKEQ
MDPCNTNPTPMTKPPPEEIKLESQSQFTADLDQFDQLLPTLEKAAQLPGLCETERMDGAV
TSVTIKSEILPATLQSTTARPTSRLNRLPELELEAIDNQFGQPGTGDQIPWANNTVTAVN
QNKPEDQCISSQLDELLCPPTTVEGRNDEKALLEQLVSFLSGKDETELAELDRALGIDKL
VQGGGLDVLSERFPPQQATPPLMMEERPNLYSQPYSSPSPTANLSGPFQGMVRQKPSLGT
MPVQVTPPRGAFSPGMGMQPRQTLNRPPAAPNQLRLQLQQRLQGQQQLIHQNRQAILNQF
AANAPVGINMRSGMQQQITPQPPLNAQMLAQRQRELYSQQHRQRQLIQQQRAMLMRQQSF
GNNLPPSSGLPVPMGNPRLPQGAPQQFPYPPNYGTNPGTPPASTSPFSQLAGNPEATLGN
RNSMVNRGMTGNMGGQFGTGINPQMQQNVFQYPGSGMVPQGEANFAPSLSPGSSMVPMPI
PPPQSSLLQQTPPASGYQSPDMKAWQQGAMGNNNVFSQAVQNQPTPAQPGVYNNMSITVS
MAGGNTNVQNMNPMMGQMQMSSLQMPGMNTVCPEQINDPALRHTGLYCNQLSSTDLLKTE
ADGTQDKKTEEFFSVVTTD
>137Callithrix jacchus|F7IG69|Mammalia|SRC1
MSGLGDSSSDPANPDSHKRKGSPCDTLASSTEKRRREQENKYLEELAELLSANISDIDSLSVKPDKCKILKKTV
DQIQLMKRMEQEKSTTDDDVQKSDISSSSQGVIEKESLGPLLLEALDGFFFVVNCEGRIVFVSENVTSYLGYNQ
EELMNTSVYSILHVGDHAEFVKNLLPKSLVNGVPWPQEATRRNSHTFNCRMLIHPPDEPGTENQEACQRYEVMQ
CFTVSQPKSIQEDGEDFQSCLICIARRLPRPPAITGVESFMTKQDTTGKIISIDTSSLRAAGRTGWEDLVRKCI
YAFFQPQGREPSYARQLFQEVMTRGTASSPSYRFILNDGTMLSAHTKCKLCYPQSPDMQPFIMGIHIIDREHSG
LSPQDDTNSGMSIPRVNPSVNPNISPAHGVARSSTLPPSSSNMVSTRINRQQSSDLHSSSHSNSSNSQGSFGCS
PGSQIVATVALNQGQASSQSSNPSLNLNNSPMEGTGISLAQFMSPRRQVTSGLATRPRMPNNSFPPNISTLSSP
VGMTSSACNNNNRSYSNIPVTSLQGMNEGPNNSVGFSASSPVLRQISSQNSPNRLNIQPAKAESKDNKEIASIL
NEMIQSDNSSSDGKPLDSGLLHNSDRLSDGDSKYSQTSHKLVQLLTTTAEQQLRHADIDTSCKDVLSCTGTSNS
ASANSSGGSCPSSHSSLTERHKILHRLLQEGSPSDITTLSVEPDKKDSASTSVSVTGQVQGNSSIKLELDASKK
KESKDHQLLRYLLDKDEKDLRSTPNLSLDDVKVKVEKKEQMDPCNTNPTPMTKPAPEEIKLEAQSQFTADLDQF
DQLLPTLEKAAQLPGLCETDRMDGAVTSVTIKSEILPASLQSATARPTSRLNRLPELELEAIDNQFGQPGTGDQ
IPWTNNTVTAINQNKSEDQCISSQLDELLCPPTTVEGRNDEKALLEQLVSFLSGKDETELAELDRALGIDKLVQ
GGGLDVLSERFPPQQATPPLMMEERPSLYSQPYSSPSPTANLPSPFQGMVRQKPSLGTMPVQVTPPRGAFSPGM
GMQPRQTLNRPPAAPNQLRLQLQQRLQGQQQLIHQNRQAILNQFAATAPVGINMRSGMQQQITPQPPLNAQMLA
QRQRELYSQQHRQRQLIQQQRAMLMRQQSFGNNLTPSSGLPVQMGNPRLPQGAPQQFPYPPNYGTNPGTPPAST
SPFSQLAANPEASLANRSSMVSRGMTGNMGGQFGTGINPQMQQNVFQYPGAGMVPQGEANFAPSLSPGSSMVPM
PIPPPQSSLLQQTPPASGYQSPDMKAWQQGAIGNNNVFSQAVQNQPTPAQPGVYNNMSITVSMAGGNTNVQNMN
PMMGQMQMSSLQMPGMNTVCPEQISDPALRHTGLYCNQLSSTDLLKTEADGTQDKKTEEFFSVVTTD
>138Callithrix jacchus|F7IG96|Mammalia|SRC1
MSGLGDSSSDPANPDSHKRKGSPCDTLASSTEKRRREQENKYLEELAELLSANISDIDSLSVKPDKCKILKKTV
DQIQLMKRMEQEKSTTDDDVQKSDISSSSQGVIEKESLGPLLLEALDGFFFVVNCEGRIVFVSENVTSYLGYNQ

EELMNTSVYSILHVGDHAEFVKNLLPKSLVNGVPWPQEATRRNSHTFNCRMLIHPPDEPGTENQEACQRYEVMQ
CFTVSQPKSIQEDGEDFQSCLICIARRLPRPPAITGVESFMTKQDTTGKIISIDTSSLRAAGRTGWEDLVRKCI
YAFFQPQGREPSYARQLFQEVMTRGTASSPSYRFILNDGTMLSAHTKCKLCYPQSPDMQPFIMGIHIIDREHSG
LSPQDDTNSGMSIPRVNPSVNPNISPAHGVARSSTLPPSSSNMVSTRINRQQSSDLHSSSHSNSSNSQGSFGCS
PGSQIVATVALNQGQASSQSSNPSLNLNNSPMEGTGISLAQFMSPRRQVTSGLATRPRMPNNSFPPNISTLSSP
VGMTSSACNNNNRSYSNIPVTSLQGMNEGPNNSVGFSASSPVLRQISSQNSPNRLNIQPAKAESKDNKEIASIL
NEMIQSDNSSSDGKPLDSGLLHNSDRLSDGDSKYSQTSHKLVQLLTTTAEQQLRHADIDTSCKDVLSCTGTSNS
ASANSSGGSCPSSHSSLTERHKILHRLLQEGSPSDITTLSVEPDKKDSASTSVSVTGQVQGNSSIKLELDASKK
KESKDHQLLRYLLDKDEKDLRSTPNLSLDDVKVKVEKKEQMDPCNTNPTPMTKPAPEEIKLEAQSQFTADLDQF
DQLLPTLEKAAQLPGLCETDRMDGAVTSVTIKSEILPASLQSATARPTSRLNRLPELELEAIDNQFGQPGTGDQ
IPWTNNTVTAINQNKSEDQCISSQLDELLCPPTTVEGRNDEKALLEQLVSFLSGKDETELAELDRALGIDKLVQ
GGGLDVLSERFPPQQATPPLMMEERPSLYSQPYSSPSPTANLPSPFQGMVRQKPSLGTMPVQVTPPRGAFSPGM
GMQPRQTLNRPPAAPNQLRLQLQQRLQGQQQLIHQNRQAILNQFAATAPVGINMRSGMQQQITPQPPLNAQMLA
QRQRELYSQQHRQRQLIQQQRAMLMRQQSFGNNLTPSSGLPVQMGNPRLPQGAPQQFPYPPNYGTNPGTPPAST
SPFSQLAANPEASLANRSSMVSRGMTGNMGGQFGTGINPQMQQNVFQYPGAGMVPQGEANFAPSLSPGSSMVPM
PIPPPQSSLLQQTPPASGYQSPDMKAWQQGAIGNNNVFSQAVQNQPTPAQPGVYNNMSITVSMAGGNTNVQNMN
PMMGQMQMSSLQMPGMNTVCPEQISDPALRHTGLYCNQLSSTDLLKTEADGTQQVQQVQVFADVQCTVNLVGGD
PYLNQPGPLGTQKPTSGPQTPQAQQKSLLQQLLTE
>139Ailuropoda melanoleuca|G1L7I1|Mammalia|SRC1
MMCLLKQRGVKFFNMSGLGDSSSDPANPDSHKRKGSPCDTLASSTEKRRREQENKYLEELAELLSANISDIDSL
SVKPDKCKILKKTVDQIQLMKRMEQEKSTTDDEVQKSDISSSSQGVIEKESLGPLLLEALDGFFFVVNCEGRIV
FVSENVTSYLGYNQEELMNTSVYSILHVGDHAEFVKNLLPKSLVNGVPWPQEATRRNSHTFNCRMLIHPPDEPG
TENQEACQRYEVMQCFTVSQPKSIQEDGEDFQSCLICIARRLPRPPAITGVESFMTKQDTTGKIISIDTSSLRA
AGRTGWEDLVRKCIYAFFQPQGREPSYARQLFQEVMTRGTASSPSYRFILNDGTMLSAHTKCKLCYPQSPDMQP
FIMGIHIIDREHSGLSPQDDTNSGMSIPRVNPSVNSSISPAHGVARSSTLPPSSNNMVSPRVNRQQSSDLHSSS
HSNSSNSQGSFGCSPGNQIVASVALNQGQASSQSTNPSLNLNNSPMEGTGISLAQFMSPRRQVSSGLAPRPRMP
NNSFPPNIPTLSSPVGMTGTACNNSNRSYSNIPVTSLQSMNEGPNNSVGFSSSSPVLRQMSSQNSPSRLNIQPA
KAESKDNKEIASILNEMIQSDNSSSDGKPLDSGLLHNNDRLSDGDNKYSQTSHKLVQLLTTTAEQQLRHADIDT
SCKEVLSCSGTSSSASANSSSGSCPSSHSSLTERHKILHRLLQEGSPSDITTLSVEPDKKDSASTSVSVTGQVQ
GNSSVKLELEASKKKESKDHQLLRYLLDKDEKDVRSTPNLSLDDVKVKVEKKEQMDPCNTNPTPVTKPAPEEIK
LESQSQFTADLDQFDQLLPTLEKAAQLPGLCEPERMDGAVTNVTIKSETLPASLQSTTARPTSRMNRLPELELE
AIDNQFGQPGTGDQIPWANNTVTAVNQNKPEDQCISSQLDELLCPPTTVEGRNDEKALLEQLVSFLSGKDETEL
AELDRALGIDKLVQGGGLDVLSERFPPQQATPPLMMEERPNLYSQPYSSPSPTANLSSPFQGMVRQKPSLGTLP
VQVTPPRGAFSPGMGMQPRQTLNRPPAAPNQLRLQLQQRLQGQQQLIHQNRQAILNQFAANAPVGINMRSGMQQ
QITPQPPLNAQMLAQRQRELYSQQHRQRQLIQQQRAMLMRQQSFGNNLPPSSGLPVQMGNPRLPQGAPQQFPYP
PNYGTNPGTPPASTSPFSQLAANPESTLANRNSMVNRGMTGNMGGQFGTGINPQMQQNVFQYPGSGMVPQGETN
FAPSLSPGSSMVPMPIPPPQSSLLQPTPPSSGYQSPDMKAWQQGAMGNNNVFSQAVQNQPTPAQPGVYNNMSIT
VSMAGGNTNVQNMNPMMGQMQMSSLQMPGMNTVCPEQINDPALRHTGLYCNQLSSTDLLKTETDGTQQVQQVQV
FADVQCTVNLVGGDPYLNQPGPLGTQKPTAGPQTPQAQQKSLLQQLLTE
>140Nomascus leucogenys|G1QJQ6|Mammalia|SRC1
MSGLGDSSSDPANPDSHKRKGSPCDTLASSTEKRRREQENKYLEELAELLSANISDIDSLSVKPDKCKILKKTV
DQIQLMKRMEQEKSTTDDDVQKSDISSSSQGVIEKESLGPLLLEALDGFFFVVNCEGRIVFVSENVTSYLGYNQ
EELMNTSVYSILHVGDHAEFVKNLLPKSLVNGVPWPQEATRRNSHTFNCRMLIHPPDEPGTENQEACQRYEVMQ
CFTVSQPKSIQEDGEDFQSCLICIARRLPRPPAITGVESFMTKQDTTGKIISIDTSSLRAAGRTGWEDLVRKCI
YAFFQPQGREPSYARQLFQEVMTRGTASSPSYRFILNDGTMLSAHTKCKLCYPQSPDMQPFIMGIHIIDREHSG
LSPQDDTNSGMSIPRVNPSVNPSISPAHGVARSSTLPPSNSNMVSTRINRQQSSDLHNSSHSNSSNSQGSFGCS
PGSQIVASVALNQGQASSQSSNPSLNLNNSPMEGTGISLAQFMSPRRQVTSGLATRPRMPNNSFPPNISTLSSP
VGMTSSACNNNNRSYSNIPVTSLQGMNEGPNNSVGFSASSPVLRQMSSQNSPSRLNIQPAKAESKDNKEIASIL
NEMIQSDNSSSDGKPLDSGLLHNNDRLSDGDSKYSQTSHKLVQLLTTTAEQQLRHADIDTSCKDVLSCTGTSSS
ASANSSGGSCPSSHSSLTERHKILHRLLQEGSPSDITTLSVEPDKKDSASTSVSVTGQVQGNSSIKLELDASKK
KESKDHQLLRYLLDKDEKDLRSTPNLSLDDVKVKVEKKEQMDPCNTNPTPMTKPTPEEIKLEAQSQFTADLDQF
DQLLPTLEKAAQLPGLCETDRMDGAVTSVTIKSEILPASLQSATARPTSRLNRLPELELEAIDNQFGQPGTGDQ
IPWTNNTVTAINQSKSEDQCISSQLDELLCPPTTVEGRNDEKALLEQLVSFLSGKDETELAELDRALGIDKLVQ
GGGLDVLSERFPPQQATPPLMMEERPNLYSQPYSSPSPTANLPSPFQGMVRQKPSLGTMPVQVTPPRGAFSPGM

GMQPRQTLNRPPAAPNQLRLQLQQRLQGQQQLIHQNRQAILNQFAATAPVGINMRSGMQQQITPQPPLNAQMLA
QRQRELYSQQHRQRQLIQQQRAMLMRQQSFGNNLPPSSGLPVQMGNPRLPQGAPQQFPYPPNYGTNPGTPPAST
SPFSQLTANPEASLANRNSMVSRGMTGNMGGQFGTGINPQMQQNVFQYPGAGMVPQGEANFAPSLSPGSSMVPM
PIPPPQSSLLQQTPPASGYQSPDMKAWQQGAIGNNNVFSQAVQNQPTPAQPGVYNNMSITVSMAGGNTNVQNMN
PMMAQMQMSSLQMPGMNTVCPEQINDPALRHTGLYCNQLSSTDLLKTEADGTQDKKTEEFFSVVTTD
>141Gorilla gorilla|G3R6P2|Mammalia|SRC1
MSGLGDSSSDPANPDSHKRKGSPCDTLASSTEKRRREQENKYLEELAELLSANISDIDSLSVKPDKCKILKKTV
DQIQLMKRMEQEKSTTDDDVQKSDISSSSQGVIEKESLGPLLLEALDGFFFVVNCEGRIVFVSENVTSYLGYNQ
EELMNTSVYSILHVGDHAEFVKNLLPKSLVNGVPWPQEATRRNSHTFNCRMLIHPPDEPGTENQEACQRYEVMQ
CFTVSQPKSIQEDGEDFQSCLICIARRLPRPPAITGVESFMTKQDTTGKIISIDTSSLRAAGRTGWEDLVRKCI
YAFFQPQGREPSYARQLFQEVMTRGTASSPSYRFILNDGTMLSAHTKCKLCYPQSPDMQPFIMGIHIIDREHSG
LSPQDDTNSGMSIPRVNPSVNPSISPAHGVARSSTLPPSNSNMVSTRINRQQSSDLHSSSHSNSSNSQGSFGCS
PGSQIVANVALNQGQASSQSSNPSLNLNNSPMEGTGISLAQFMSPRRQVTSGLATRPRMPNNSFPPNISTLSSP
VGMTSSACNNNNRSYSNIPVTSLQGMNEGPNNSVGFSASSPVLRQMSSQNSPSRLNIQPAKAESKDNKEIASIL
NEMIQSDNSSSDGKPLDSGLLHNNDRLSDGDSKYSQTSHKLVQLLTTTAEQQLRHADIDTSCKDVLSCTGTSNS
ASANSSGGSCPSSHSSLTERHKILHRLLQEGSPSDITTLSVEPDKKDSASTSVSVTGQVQGNSSIKLELDASKK
KESKDHQLLRYLLDKDEKDLRSTPNLSLDDVKVKVEKKEQMDPCNTNPTPMTKPTPEEIKLEAQSQFTADLDQF
DQLLPTLEKAAQLPGLCETDRMDGAVTSVTIKSEILPASLQSATARPTSRLNRLPELELEAIDNQFGQPGTGDQ
IPWTNNTVTAINQSKSEDQCISSQLDELLCPPTTVEGRNDEKALLEQLVSFLSGKDETELAELDRALGIDKLVQ
GGGLDVLSERFPPQQATPPLIMEERPNLYSQPYSSPSPTTNLPSPFQGMVRQKPSLGTMPVQVTPPRGAFSPGM
GMQPRQTLNRPPAAPNQLRLQLQQRLQGQQQLIHQNRQAILNQFAATAPVGISMRSGMQQQITPQPPLNAQMLA
QRQRELYSQQHRQRQLIQQQRAMLMRQQSFGNNLPPSSGLPVQMGNPRLPQGAPQQFPYPPNYGTNPGTPPAST
SPFSQLAANPEASLANRNSMVSRGMTGNMGGQFGTGINPQMQQNVFQYPGAGMVPQGEANFAPSLSPGSSMVPM
PIPPPQSSLLQQTPPASGYQSPDMKAWQQGAIGNNNVFSQAVQNQPTPAQPGVYNNMSITVSMAGGNTNVQNMN
PMMAQMQMSSLQMPGMNTVCPEQINDPALRHTGLYCNQLSSTDLLKTEADGTQQVQQVQVFADVQCTVNLVGGD
PYLNQPGPLGTQKPTSGPQTPQAQQKSLLQQLLTE
>142Gorilla gorilla gorilla|G3RV45|Mammalia|SRC1
MSGLGDSSSDPANPDSHKRKGSPCDTLASSTEKRRREQENKYLEELAELLSANISDIDSLSVKPDKCKILKKTV
DQIQLMKRMEQEKSTTDDDVQKSDISSSSQGVIEKESLGPLLLEALDGFFFVVNCEGRIVFVSENVTSYLGYNQ
EELMNTSVYSILHVGDHAEFVKNLLPKSLVNGVPWPQEATRRNSHTFNCRMLIHPPDEPGTENQEACQRYEVMQ
CFTVSQPKSIQEDGEDFQSCLICIARRLPRPPAITGVESFMTKQDTTGKIISIDTSSLRAAGRTGWEDLVRKCI
YAFFQPQGREPSYARQLFQEVMTRGTASSPSYRFILNDGTMLSAHTKCKLCYPQSPDMQPFIMGIHIIDREHSG
LSPQDDTNSGMSIPRVNPSVNPSISPAHGVARSSTLPPSNSNMVSTRINRQQSSDLHSSSHSNSSNSQGSFGCS
PGSQIVANVALNQGQASSQSSNPSLNLNNSPMEGTGISLAQFMSPRRQVTSGLATRPRMPNNSFPPNISTLSSP
VGMTSSACNNNNRSYSNIPVTSLQGMNEGPNNSVGFSASSPVLRQMSSQNSPSRLNIQPAKAESKDNKEIASIL
NEMIQSDNSSSDGKPLDSGLLHNNDRLSDGDSKYSQTSHKLVQLLTTTAEQQLRHADIDTSCKDVLSCTGTSNS
ASANSSGGSCPSSHSSLTERHKILHRLLQEGSPSDITTLSVEPDKKDSASTSVSVTGQVQGNSSIKLELDASKK
KESKDHQLLRYLLDKDEKDLRSTPNLSLDDVKVKVEKKEQMDPCNTNPTPMTKPTPEEIKLEAQSQFTADLDQF
DQLLPTLEKAAQLPGLCETDRMDGAVTSVTIKSEILPASLQSATARPTSRLNRLPELELEAIDNQFGQPGTGDQ
IPWTNNTVTAINQSKSEDQCISSQLDELLCPPTTVEGRNDEKALLEQLVSFLSGKDETELAELDRALGIDKLVQ
GGGLDVLSERFPPQQATPPLIMEERPNLYSQPYSSPSPTTNLPSPFQGMVRQKPSLGTMPVQVTPPRGAFSPGM
GMQPRQTLNRPPAAPNQLRLQLQQRLQGQQQLIHQNRQAILNQFAATAPVGISMRSGMQQQITPQPPLNAQMLA
QRQRELYSQQHRQRQLIQQQRAMLMRQQSFGNNLPPSSGLPVQMGNPRLPQGAPQQFPYPPNYGTNPGTPPAST
SPFSQLAANPEASLANRNSMVSRGMTGNMGGQFGTGINPQMQQNVFQYPGAGMVPQGEANFAPSLSPGSSMVPM
PIPPPQSSLLQQTPPASGYQSPDMKAWQQGAIGNNNVFSQAVQNQPTPAQPGVYNNMSITVSMAGGNTNVQNMN
PMMAQMQMSSLQMPGMNTVCPEQINDPALRHTGLYCNQLSSTDLLKTEADGTQDKKTEEFFSVVTTD
>143Loxodonta africana|G3TD92|Mammalia|SRC1
MSGLGDSSSDPANPDSHKRKGSPCDTLASSTEKRRREQENKYLEELAELLSANISDIDSLSVKPDKCKILKKTV
DQIQLMKRMEQEKSTTDDDVQKSDISSSSQGVIEKESLGPLLLEALDGFFFVVNCEGRIVFVSENVTSYLGYNQ
EELMNTSVYSILHVGDHAEFVKNLLPKSLVNGVPWPQEATRRNSHTFNCRMLIHPPDEPGTENQEACQRYEVMQ
CFTVSQPKSIQEDGEDFQSCLICIARRLPRPPAVTGVESFMTKQDTTGKIISIDTSSLRAAGRTGWEDLVRKCI
YAFFQPQGREPSYARQLFQEETVMTRGTASSPSYRFILNDGTMLSAHTKCKLCYPQTPDMQPFIMGIHIIDREH
SGLSPQDDSNSGMSIPRVNPTVNPSISPAHSVARSSTLPPSNSNMAPTRVNRQQSSDLHSSSHSNSSNSQGSFG
CPPGNQIATSVALNQGQAGSQSSNPSLNLNNSSMEGTGITLAQFMSPRRQVSSGLAARPRMPNNSFSPNIPTLS

SPVGMTNSACNNSNRSYSNIPVTSLQGMNEGPNNSVGFSASSPVLRQMSSQNSPSRLNIQPPKAESKDNKEITS
ILNEMIQSDNSSSDGKPLDSGLLHNNDRLSDGDNKYSQTSHKLVQLLTTTAEQQLRHADIDTSCKDVLSCTGTS
NSASGNSSGGSCPSSHSSLTERHKILHRLLQEGSPSDITTLSVEPDKKDSASTSVSVTGQVQGSSTIKLELDAS
KKKESKDHQLLRYLLDKDEKDLRSTPNLSLDDVKVKVEKKEQMDPCNTNPTPMTKPAPEEVKLESQNQFTADLD
QFDQLLPTLEKAAQLPGLCEADRMDGAVTSVPIKSEILPTSLQSTTARPTTRLNRLPELELEAIDNQFGQQGTG
DQMPWANNTVAAVNQNKPEDQCISSQLDELLCPPTTVEGRNDEKALLEQLVSFLSGKDETELAELDRALGIDKL
VQGGGLDVLSERFPPQQATPPLMMEERPNLYSQPYSSPSPTANLPSPFQGMVRQKPSLGTVPVQVTPPRGAFSP
GMGMQPRQTLNRPPAAPNQLRLQLQQRLQGQQQLIHQNRQAILNQFAATAPVGINMRSGMQQQITPQPPLNAQM
LAQRQRELYSQQHRQRQLIQQQRAMLMRQQSFGNSLPPSSGLPVQMGNPRLPQGAPQQFPYPPNYGTNPGTPPA
STSPFSQLAANPEASLANRSSMVNRGMTGNMGGQFGTGISPQMQQNVFQYPGSGMVSQGEANFAPSLSPGSSMV
PMPIPPPQSSLLQQTPPASGYQSPDMKAWQQGAMGNTNSVFSQAVQSQPTPAQPGVYNNMSITVSMAGGNTNVQ
NMNPMMGQMQMSSLQMQGMNTVCPEQMNDPALRHTGLYCNQLSSTDLLKTETDGTQQVQQVQVFADVQCTVNLV
GGDPYLNQPGPLGTQKPTSGPQTPQAQQKSLLQQLLTE

>144Macaca mulatta|G7N9J2|Mammalia|SRC1
MSGLGDSSSDPANPDSHKRKGSPCDTLASSTEKRRREQENKYLEELAELLSANISDIDSLSVKPDKCKILKKTV
DQIQLMKRMEQEKSTTDDDVQKSDISSSSQGVIEKESLGPLLLEALDGFFFVVNCEGRIVFVSENVTSYLGYNQ
EELMNTSVYSILHVGDHAEFVKNLLPKSLVNGVPWPQEATRRNSHTFNCRMLIHPPDEPGTENQEACQRYEVMQ
CFTVSQPKSIQEDGEDFQSCLICIARRLPRPPAITGVESFMTKQDTTGKIISIDTSSLRAAGRTGWEDLVRKCI
YAFFQPQGREPSYARQLFQEVMTRGTASSPSYRFILNDGTMLSAHTKCKLCYPQSPDMQPFIMGIHIIDREHSG
LSPQDDTNSGMSIPRVNPSVNPSISPAHGVARSSTLPPSNSNMVSTRINRQQSSDLHSGSHSNSSNSQGSFGCS
PGSQIVANVALNQGQASSQSSNPSLNLNNSPMEGTGISLAQFMSPRRQVTSGLTTRPRMPNNSFPPNISTLSSP
VGMTSSACNNNTNRSYSNIPVTSLQGMNEGPNNSVGFSANSPVLRQMSSQNSPSRLNIQPAKAESKDNKEIASI
LNEMIQSDNSSSDGKPLDSGLLHNNDRLSDGDSKYSQTSHKLVQLLTTTAEQQLRHADIDTSCKDVLSCTGTSN
SASANSSGGSCPSSHSSLTERHKILHRLLQEGSPSDITTLSVEPDKKDSASTSVSVTGQVQGNSSIKLELDASK
KKESKDHQLLRYLLDKDEKDLRSTPNLSLDDVKVKVEKKEQMDPCNTNPTPMTKPTPEEIKLEAQSQFTADLDQ
FDQLLPTLEKAAQLPGLCETDRMDGAVTSVTIKSEILPASLQSATARPTSRLNRLPELELEAIDNQFGQPGTGD
QIPWTNNTVTAINQNKSEDQCISSQLDELLCPPTTVEGRNDEKALLEQLVSFLSGKDETELAELDRALGIDKLV
QGGGLDVLSERFPPQQATPPLMMEERPNLYSQPYSSPSPTANLPSPFQGMVRQKPSLGTMPVQVTPPRGAFSPG
MGMQPRQTLNRPPAAPNQLRLQLQQRLQGQQQLIHQNRQAILNQFAATAPVGINMRSGMQQQITPQPPLNAQML
AQRQRELYSQQHRQRQLIQQQRAMLMRQQSFGNNLPPSSGLPVQMGNPRLPQGAPQQFPYPPNYGTNPGTPPAS
TSPFSQLAANPEASLANRNSMVSRGMTGNMGGQFGTGINPQIQQNVFQYPGAGMVPQGEASFAPSLSPGSSMVP
MPIPPPQSSLLQQTPPASGYQSPDMKAWQQGAIGNNSVFSQAVQNQSTPAQPGVYNNMSITVSMAGGNTNVQNM
NPMMAQMQMSSLQMPGMNTVCPEQINDPALRHTGLYCNQLSSTDLLKTEADGTQQVQQVQVFADVQCTVNLVGG
DPYLNQPGPLGTQKPTSGPQTPQAQQKSLLQQLLTE
>145Macaca fascicularis|G7PLR7|Mammalia|SRC1
MSGLGDSSSDPANPDSHKRKGSPCDTLASSTEKRRREQENKYLEELAELLSANISDIDSLSVKPDKCKILKKTV
DQIQLMKRMEQEKSTTDDDVQKSDISSSSQGVIEKESLGPLLLEALDGFFFVVNCEGRIVFVSENVTSYLGYNQ
EELMNTSVYSILHVGDHAEFVKNLLPKSLVNGVPWPQEATRRNSHTFNCRMLIHPPDEPGTENQEACQRYEVMQ
CFTVSQPKSIQEDGEDFQSCLICIARRLPRPPAITGVESFMTKQDTTGKIISIDTSSLRAAGRTGWEDLVRKCI
YAFFQPQGREPSYARQLFQEVMTRGTASSPSYRFILNDGTMLSAHTKCKLCYPQSPDMQPFIMGIHIIDREHSG
LSPQDDTNSGMSIPRVNPSVNPSISPAHGVARSSTLPPSNSNMVSTRINRQQSSDLHSGSHSNSSNSQGSFGCS
PGSQIVANVALNQGQASSQSSNPSLNLNNSPMEGTGISLAQFMSPRRQVTSGLTTRPRMPNNSFPPNISTLSSP
VGMTSSACNNNTNRSYSNIPVTSLQGMNEGPNNSVGFSANSPVLRQMSSQNSPSRLNIQPAKAESKDNKEIASI
LNEMIQSDNSSSDGKPLDSGLLHNNDRLSDGDSKYSQTSHKLVQLLTTTAEQQLRHADIDTSCKDVLSCTGTSN
SASANSSGGSCPSSHSSLTERHKILHRLLQEGSPSDITTLSVEPDKKDSASTSVSVTGQVQGNSSIKLELDASK
KKESKDHQLLRYLLDKDEKDLRSTPNLSLDDVKVKVEKKEQMDPCNTNPTPMTKPTPEEIKLEAQSQFTADLDQ
FDQLLPTLEKAAQLPGLCETDRMDGAVTSVTIKSEILPASLQSATARPTSRLNRLPELELEAIDNQFGQPGTGD
QIPWTNNTVTAINQNKSEDQCISSQLDELLCPPTTVEGRNDEKALLEQLVSFLSGKDETELAELDRALGIDKLV
QGGGLDVLSERFPPQQATPPLMMEERPNLYSQPYSSPSPTANLPSPFQGMVRQKPSLGTMPVQVTPPRGAFSPG
MGMQPRQTLNRPPAAPNQLRLQLQQRLQGQQQLIHQNRQAILNQFAATAPVGINMRSGMQQQITPQPPLNAQML
AQRQRELYSQQHRQRQLIQQQRAMLMRQQSFGNNLPPSSGLPVQMGNPRLPQGAPQQFPYPPNYGTNPGTPPAS
TSPFSQLAANPEASLANRNSMVSRGMTGNMGGQFGTGINPQIQQNVFQYPGAGMVPQGEASFAPSLSPGSSMVP
MPIPPPQSSLLQQTPPASGYQSPDMKAWQQGAIGNNSVFSQAVQNQPTPAQPGVYNNMSITVSMAGGNTNVQNM

NPMMAQMQMSSLQMPGMNTVCPEQINDPALRHTGLYCNQLSSTDLLKTEADGTQQVQQVQVFADVQCTVNLVGG
DPYLNQPGPLGTQKPTSGPQTPQAQQKSLLQQLLTE
>146Otolemur garnettii|H0WH45|Mammalia|SRC1
MSGLGDSSSDPANPDSHKRKGSPCDTLASSTEKRRREQENKYLEELAELLSANISDIDSLSVKPDKCKILKKTV
DQIQLMKRMEQEKSTTDDDVQKSDISSSSQGVIEKESLGPLLLEALDGFFFVVNCEGRIVFVSENVTSYLGYNQ
EELMNTSVYSILHVGDHAEFVKNLLPKSLVNGVPWPQEATRRNSHTFNCRMLIHPPDEPGTENQEACQRYEVMQ
CFTVSQPKSIQEDGEEYMCQVLCDLPPQPPPHPPSVKKLSSLQTKWNTYSKIISIDTSSLRAAGRTGWEDLVRK
CIYAFFQPQGREPSYARQLFQEVMTRGTASSPSYRFILNDGTMLSAHTKCKLCYPQSPDMQPFIMGIHIIDREH
SGLSPQDDTNSGMTIPRVNPSVNPNISPAHGVARPATLPPSNNSMVSTRVNRQQSSDLHSSAHSNSGNSQGNFG
CSPGNQIVANVALNQGQASSQSSNPSLNLSNSPMEGTGMSLAQFMSPRRQVTSGLAARPRMPNSSFPPNISTLS
SPVGMTSSACNTSNRSYSNIPVTSLQGMNEGPNNSVGFSASSPVLRQMSSQNSPGRLNIQPAKAESKDNKEIAS
ILNEMIQSDNSSSDGKPLDSGLLHNNDRLSDGDSKYSQTSHKLVQLLTTTAEQQLRHADIDTSCKDVLSCTGTS
NSASANSSGGSCPSSHSSLTERHKILHRLLQEGSPSDITTLSVEPDKKDSASTSVSVTGQVQGNSSIKLELDAS
KKKESKDHQLLRYLLDKDEKDLRSTPNLSLDDVKVKVEKKEQMDPCNTNPTPMTKPAPEEIKLESQSQFSADLD
QFDQLLPTLEKAAQLPGLCETDRMDGAVTNVAIKSEILPTSLQSTTARPTSRLNRLPELELETIDNQFGQPGTG
DQIPWTNNTVTAVNQNKPEDQCISSQLDELLCPPTTVEGRNDEKALLEQLVSFLSGKDETELAELDRALGIDKL
VQGGGLDVLSERFPPQQATPPLMMEERPNLYSQPYSSPSPTANLPSPFQGMVRQKPSLGTMPVQVTPPRGAFSP
GMGMQPRQTLNRPPAAPNQLRLQLQQRLQGQQQLIHQNRQAILNQFAATAPVGINMRSGMQQQITPQPPLNAQM
LAQRQRELYSQQHRQRQLIQQQRAMLMRQQSFGNNLPPSSGLPVQMGNPRLPQGAPQQFPYPPNYGTNPGTPPA
STSPFSQLAGNPEASLANRNSMVNRGMTGNMGGQFGTGINPQMQQNVFQYPGSGMVPQGEANFAPSLSPGSSMV
PMPIPPPQSSLLQQTPPASGYQSPDMKAWQQGAIGNINSVFSQAVQNQPTPAQPGVYNNMSITVSMAGGNTNVQ
NMNPMMGQMQMSSLQMPGMNTVCPEQISDPALRHTGLYCNQLSSTDLLKTEADGTQQVQQVQVFADVQCTVNLV
GGDPYLNQPGPLGTQKPTSGPQTPQAQQKSLLQQLLTE
>147Pongo abelii|H2P6T7|Mammalia|SRC1
MSGLGDSSSDPANPDSHKRKGSPCDTLASSTEKRRREQENKYLEELAELLSANISDIDSL
SVKPDKCKILKKTVDQIQLMKRMEQEKSTTDDDVQKSDISSSSQGVIEKESLGPLLLEAL
DGFFFVVNCEGRIVFVSENVTSYLGYNQEELMNTSVYSILHVGDHAEFVKNLLPKSLVNG
VPWPQEATRRNSHTFNCRMLIHPPDEPGTENQEAGQRYEVMQCFTVSQPKSIQEDGEDFQ
SCLICIARRLPRPPAITGVESFMTKQDTTGKIISIDTSSLRAAGRTGWEDLVRKCIYAFF
QPQGREPSYARQLFQEVMTRGTASSPSYRFILNDGTMLSAHTKCKLCYPQSPDMQPFIMG
IHIIDREHSGLSPQDDTNSGMSIPRVNPSVNPSISPAHGVARSSTLPPSNSNMVSTRINR
QQSSDLHSSSHSNSSNSQGSFGCSPGSQIVANVALNQGQASSQSSNPSLNLNNSPMEGTG
ISLAQFMSPRRQVTSGLATRPRMPNNSFPPNISTLSSPVGMTSSTCNNNNRSYSNIPVTS
LQGMNEGPNNSVGFSASSPVLRQMSSQNSPSRLNIQPAKAESKDNKEIASILNEMIQSDN
SSSDGKPLDSGLLHNNDRLSDGDSKYSQTSHKLVQLLTTTAEQQLRHADIDTSCKDVLSC
TGTSNSASANSSGGSCPSSHSSLTERHKILHRLLQEGSPSDITTLSVEPDKKDSASTSVS
VTGQVQGNSSIKLELDASKKKESKDHQLLRYLLDKDEKDLRSTPNLSLDDVKVKVEKKEQ
MDPCNTNPTPMTKPTPEEIKLEAQSQFTADLDQFDQLLPTLEKAAQLPGLCETDRMDGAV
TSVTIKSEILPASLQSATARPTSRLNRLPELELEAIDNQFGQPGTGDQIPWTNNTVTAIN
QSKSEDQCISSQLDELLCPPTTVEGRNDEKALLEQLVSFLSGKDETELAELDRALGIDKL
VQGGGLDVLSERFPPQQATPPLMMEERPNLYSQPYSSPSPTANLPSPFQGMVRQKPSLGT
MPVQVTPPRGAFSAGMGMQPRQTLNRPPAAPNQLRLQLQQRLQGQQQLIHQNRQAILNQF
AATAPVGINMRSGMQQQITPQPPLNAQMLAQRQRELYSQQHRQRQLIQQQRAMLMRQQSF
GNNLPPSSGLPVQMGNPRLPQGAPQQFPYPPNYGTNPGTPPASTSPFSQLAANPEASLAS
RNSMVSRGMTGNMGGQFGTGINPQMQQNVFQYPGAGMVPQGEANFAPSLSPGSSMVPMPI
PPPQSSLLQQTPPASGYQSPDMKAWQQGAIGNNNVFSQAVQNQPTPAQPGVYNNMSITVS
MAGGNTNVQNMNPMMAQMQMSSLQMPGMNTVCPEQINDPALRHTGLYCNQLSSTDLLKTE
ADGTQDKKTEEFFSVVTTD
>148Pan troglodytes|H2QHJ2|Mammalia|SRC1
MSGLGDSSSDPANPDSHKRKGSPCDTLASSTEKRRREQENKYLEELAELLSANISDIDSLSVKPDKCKILKKTV
DQIQLMKRMEQEKSTTDDDVQKSDISSSSQGVIEKESLGPLLLEALDGFFFVVNCEGRIVFVSENVTSYLGYNQ
EELMNTSVYSILHVGDHAEFVKNLLPKSLVNGVPWPQEATRRNSHTFNCRMLIHPPDEPGTENQEACQRYEVMQ
CFTVSQPKSIQEDGEDFQSCLICIARRLPRPPAITGVESFMTKQDTTGKIISIDTSSLRAAGRTGWEDLVRKCI
YAFFQPQGREPSYARQLFQEVMTRGTASSPSYRFILNDGTMLSAHTKCKLCYPQSPDMQPFIMGIHIIDREHSG

LSPQDDTNSGMSIPRVNPSVNPSISPAHGVARSSTLPPSNSNMVSTRINRQQSSDLHSSSHSNSSNSQGSFGCS
PGSQIVANVALNQGQASSQSSNPSLNLNNSPMEGTGISLAQFMSPRRQVTSGLATRPRMPNNSFPPNISTLSSP
VGMTSSACNNNNRSYSNIPVTSLQGMNEGPNNSVGFSASSPVLRQMSSQNSPSRLNIQPAKAESKDNKEIASIL
NEMIQSDNSSSDGKPLDSGLLHNNDRLSDGDSKYSQTSHKLVQLLTTTAEQQLRHADIDTSCKDVLSCTGTSNS
ASANSSGGSCPSSHSSLTERHKILHRLLQEGSPSDITTLSVEPDKKDSASTSVSVTGQVQGNSSIKLELDASKK
KESKDHQLLRYLLDKDEKDLRSTPNLSLDDVKVKVEKKEQMDPCNTNPTPMTKPTPEEIKLEAQSQFTADLDQF
DQLLPTLEKAAQLPGLCETDRMDGAVTSVTIKSEILPASLQSATARPTSRLNRLPELELEAIDNQFGQPGTGDQ
IPWTNNTVTAINQSKSEDQCISSQLDELLCPPTTVEGRNDEKALLEQLVSFLSGKDETELAELDRALGIDKLVQ
GGGLDVLSERFPPQQATPPLIMEERPNLYSQPYSSPSPTANLPSPFQGMVRQKPSLGTMPVQVTPPRGAFSPGM
GMQPRQTLNRPPAAPNQLRLQLQQRLQGQQQLIHQNRQAILNQFAATAPVGINMRSGMQQQITPQPPLNAQMLA
QRQRELYSQQHRQRQLIQQQRAMLMRQQSFGNNLPPSSGLPVQMGNPRLPQGAPQQFPYPPNYGTNPGTPPAST
SPFSQLAANPEASLANRNSMVSRGMTGNMGGQFGTGINPQMQQNVFQYPGAGMVPQGEANFAPSLSPGSSMVPM
PIPPPQSSLLQQTPPASGYQSPDMKAWQQGAIGNNNVFSQAVQNQPTPAQPGVYNNMSITVSMAGGNTNVQNMN
PMMAQMQMSSLQMPGMNTVCPEQINDPALRHTGLYCNQLSSTDLLKTEADGTQQVQQVQVFADVQCTVNLVGGD
PYLNQPGPLGTQKPTSGPQTPQAQQKSLLQQLLTE
>149Macaca mulatta|H9G174|Mammalia|SRC1
MSGLGDSSSDPANPDSHKRKGSPCDTLASSTEKRRREQENKYLEELAELLSANISDIDSLSVKPDKCKILKKTV
DQIQLMKRMEQEKSTTDDDVQKSDISSSSQGVIEKESLGPLLLEALDGFFFVVNCEGRIVFVSENVTSYLGYNQ
EELMNTSVYSILHVGDHAEFVKNLLPKSLVNGVPWPQEATRRNSHTFNCRMLIHPPDEPGTENQEACQRYEVMQ
CFTVSQPKSIQEDGEDFQSCLICIARRLPRPPAITGVESFMTKQDTTGKIISIDTSSLRAAGRTGWEDLVRKCI
YAFFQPQGREPSYARQLFQEVMTRGTASSPSYRFILNDGTMLSAHTKCKLCYPQSPDMQPFIMGIHIIDREHSG
LSPQDDTNSGMSIPRVNPSVNPSISPAHGVARSSTLPPSNSNMVSTRINRQQSSDLHSGSHSNSSNSQGSFGCS
PGSQIVANVALNQGQASSQSSNPSLNLNNSPMEGTGISLAQFMSPRRQVTSGLTTRPRMPNNSFPPNISTLSSP
VGMTSSACNNNTNRSYSNIPVTSLQGMNEGPNNSVGFSANSPVLRQMSSQNSPSRLNIQPAKAESKDNKEIASI
LNEMIQSDNSSSDGKPLDSGLLHNNDRLSDGDSKYSQTSHKLVQLLTTTAEQQLRHADIDTSCKDVLSCTGTSN
SASANSSGGSCPSSHSSLTERHKILHRLLQEGSPSDITTLSVEPDKKDSASTSVSVTGQVQGNSSIKLELDASK
KKESKDHQLLRYLLDKDEKDLRSTPNLSLDDVKVKVEKKEQMDPCNTNPTPMTKPTPEEIKLEAQSQFTADLDQ
FDQLLPTLEKAAQLPGLCETDRMDGAVTSVTIKSEILPASLQSATARPTSRLNRLPELELEAIDNQFGQPGTGD
QIPWTNNTVTAINQNKSEDQCISSQLDELLCPPTTVEGRNDEKALLEQLVSFLSGKDETELAELDRALGIDKLV
QGGGLDVLSERFPPQQATPPLMMEERPNLYSQPYSSPSPTANLPSPFQGMVRQKPSLGTMPVQVTPPRGAFSPG
MGMQPRQTLNRPPAAPNQLRLQLQQRLQGQQQLIHQNRQAILNQFAATAPVGINMRSGMQQQITPQPPLNAQML
AQRQRELYSQQHRQRQLIQQQRAMLMRQQSFGNNLPPSSGLPVQMGNPRLPQGAPQQFPYPPNYGTNPGTPPAS
TSPFSQLAANPEASLANRNSMVSRGMTGNMGGQFGTGINPQIQQNVFQYPGAGMVPQGEASFAPSLSPGSSMVP
MPIPPPQSSLLQQTPPASGYQSPDMKAWQQGAIGNNVFSQAVQNQSTPAQPGVYNNMSITVSMAGGNTNVQNMN
PMMAQMQMSSLQMPGMNTVCPEQINDPALRHTGLYCNQLSSTDLLKTEADGTQQVQQVQVFADVQCTVNLVGGD
PYLNQPGPLGTQKPTSGPQTPQAQQKSLLQQLLTE

>150Macaca mulatta|H9G175|Mammalia|SRC1
MSGLGDSSSDPANPDSHKRKGSPCDTLASSTEKRRREQENKYLEELAELLSANISDIDSLSVKPDKCKILKKTV
DQIQLMKRMEQEKSTTDDDVQKSDISSSSQGVIEKESLGPLLLEALDGFFFVVNCEGRIVFVSENVTSYLGYNQ
EELMNTSVYSILHVGDHAEFVKNLLPKSLVNGVPWPQEATRRNSHTFNCRMLIHPPDEPGTENQEACQRYEVMQ
CFTVSQPKSIQEDGEDFQSCLICIARRLPRPPAITGVESFMTKQDTTGKIISIDTSSLRAAGRTGWEDLVRKCI
YAFFQPQGREPSYARQLFQEVMTRGTASSPSYRFILNDGTMLSAHTKCKLCYPQSPDMQPFIMGIHIIDREHSG
LSPQDDTNSGMSIPRVNPSVNPSISPAHGVARSSTLPPSNSNMVSTRINRQQSSDLHSGSHSNSSNSQGSFGCS
PGSQIVANVALNQGQASSQSSNPSLNLNNSPMEGTGISLAQFMSPRRQVTSGLTTRPRMPNNSFPPNISTLSSP
VGMTSSACNNNTNRSYSNIPVTSLQGMNEGPNNSVGFSANSPVLRQMSSQNSPSRLNIQPAKAESKDNKEIASI
LNEMIQSDNSSSDGKPLDSGLLHNNDRLSDGDSKYSQTSHKLVQLLTTTAEQQLRHADIDTSCKDVLSCTGTSN
SASANSSGGSCPSSHSSLTERHKILHRLLQEGSPSDITTLSVEPDKKDSASTSVSVTGQVQGNSSIKLELDASK
KKESKDHQLLRYLLDKDEKDLRSTPNLSLDDVKVKVEKKEQMDPCNTNPTPMTKPTPEEIKLEAQSQFTADLDQ
FDQLLPTLEKAAQLPGLCETDRMDGAVTSVTIKSEILPASLQSATARPTSRLNRLPELELEAIDNQFGQPGTGD
QIPWTNNTVTAINQNKSEDQCISSQLDELLCPPTTVEGRNDEKALLEQLVSFLSGKDETELAELDRALGIDKLV
QGGGLDVLSERFPPQQATPPLMMEERPNLYSQPYSSPSPTANLPSPFQGMVRQKPSLGTMPVQVTPPRGAFSPG
MGMQPRQTLNRPPAAPNQLRLQLQQRLQGQQQLIHQNRQAILNQFAATAPVGINMRSGMQQQITPQPPLNAQML
AQRQRELYSQQHRQRQLIQQQRAMLMRQQSFGNNLPPSSGLPVQMGNPRLPQGAPQQFPYPPNYGTNPGTPPAS

TSPFSQLAANPEASLANRNSMVSRGMTGNMGGQFGTGINPQIQQNVFQYPGAGMVPQGEASFAPSLSPGSSMVP
MPIPPPQSSLLQQTPPASGYQSPDMKAWQQGAIGNNVFSQAVQNQSTPAQPGVYNNMSITVSMAGGNTNVQNMN
PMMAQMQMSSLQMPGMNTVCPEQINDPALRHTGLYCNQLSSTDLLKTEADGTQDKKTEEFFSVVTTD
>151Macaca mulatta|H9ZER0|Mammalia|SRC1
MSGLGDSSSDPANPDSHKRKGSPCDTLASSTEKRRREQENKYLEELAELLSANISDIDSLSVKPDKCKILKKTV
DQIQLMKRMEQEKSTTDDDVQKSDISSSSQGVIEKESLGPLLLEALDGFFFVVNCEGRIVFVSENVTSYLGYNQ
EELMNTSVYSILHVGDHAEFVKNLLPKSLVNGVPWPQEATRRNSHTFNCRMLIHPPDEPGTENQEACQRYEVMQ
CFTVSQPKSIQEDGEDFQSCLICIARRLPRPPAITGVESFMTKQDTTGKIISIDTSSLRAAGRTGWEDLVRKCI
YAFFQPQGREPSYARQLFQEVMTRGTASSPSYRFILNDGTMLSAHTKCKLCYPQSPDMQPFIMGIHIIDREHSG
LSPQDDTNSGMSIPRVNPSVNPSISPAHGVARSSTLPPSNSNMVSTRINRQQSSDLHSGSHSNSSNSQGSFGCS
PGSQIVANVALNQGQASSQSSNPSLNLNNSPMEGTGISLAQFMSPRRQVTSGLTTRPRMPNNSFPPNISTLSSP
VGMTSSACNNNTNRSYSNIPVTSLQGMNEGPNNSVGFSANSPVLRQMSSQNSPSRLNIQPAKAESKDNKEIASI
LNEMIQSDNSSSDGKPLDSGLLHNNDRLSDGDSKYSQTSHKLVQLLTTTAEQQLRHADIDTSCKDVLSCTGTSN
SASANSSGGSCPSSHSSLTERHKILHRLLQEGSPSDITTLSVEPDKKDSASTSVSVTGQVQGNSSIKLELDASK
KKESKDHQLLRYLLDKDEKDLRSTPNLSLDDVKVKVEKKEQMDPCNTNPTPMTKPTPEEIKLEAQSQFTADLDQ
FDQLLPTLEKAAQLPGLCETDRMDGAVTSVTIKSEILPASLQSATARPTSRLNRLPELELEAIDNQFGQPGTGD
QIPWTNNTVTAINQNKSEDQCISSQLDELLCPPTTVEGRNDEKALLEQLVSFLSGKDETELAELDRALGIDKLV
QGGGLDVLSERFPPQQATPPLMMEERPNLYSQPYSSPSPTANLPSPFQGMVRQKPSLGTMPVQVTPPRGAFSPG
MGMQPRQTLNRPPAAPNQLRLQLQQRLQGQQQLIHQNRQAILNQFAATAPVGINMRSGMQQQITPQPPLNAQML
AQRQRELYSQQHRQRQLIQQQRAMLMRQQSFGNNLPPSSGLPVQMGNPRLPQGAPQQFPYPPNYGTNPGTPPAS
TSPFSQLAANPEASLANRNSMVSRGMTGNMGGQFGTGINPQIQQNVFQYPGAGMVPQGEASFAPSLSPGSSMVP
MPIPPPQSSLLQQTPPASGYQSPDMKAWQQGAIGNNVFSQAVQNQSTPAQPGVYNNMSITVSMAGGNTNVQNMN
PMMAQMQMSSLQMPGMNTVCPEQINDPALRHTGLYCNQLSSTDLLKTEADGTQVQQVQVFADVQCTVNLVGGDP
YLNQPGPLGTQKPTSGPQTPQAQQKSLLQQLLTE
>152Pteropus alecto|L5KRG5|Mammalia|SRC1
MSGLGDSSSDPANPDSHKRKGSPCDTLASSTEKRRREQENKYLEELAELLSANISDIDSLSVKPDKCKILKKTV
DQIQLMKRMEQEKSATDDEVQKSDISSSSQGVIEKESLGPLLLEALDGFFFVVNCEGRIVFVSENVTSYLGYNQ
EELMNTSVYSILHVGDHAEFVKNLLPKSLVNGVPWPQEATRRNSHTFNCRMLIHPPDEPGIENQEACQRYEVMQ
CFTVSQPKSIQEDGEDFQSCLICIARRLPRPPAITGVESFMTKQDTTGKIISIDTSSLRAAGRTGWEDLVRKCI
YAFFQPQGREPSYARQLFQEVMTRGTASSPSYRFILNDGTMLSAHTKCKLCYPQNADMQPFIMGIHIIDREHSG
LSPQDDTNSGMSVPRVNPSVNPSISPAHGVARSSTLPPSNSNIVSTRVNRQQSSDLHSSSHSNSSNSQGSFGCS
PGNQIVASVALNQGQASSQSTNPSLNLNNSPMEGTGISLAQFMSPRRQVSSGLATRPRMPSNSFPPNIPTLSSP
VGMTSTACNNSNRSYSNIPVTPLQSMNEGPNNSVGFSASSPVLRQISSQNSPSRLNIQPAKAESKDNKEIASIL
NEMIQSDNSSSDGKPLDSGLLHNNDRLSDGDNKYSQTSHKLVQLLTTTAEQQLRHADIDTSCKEVLSCAGTSSS
ASANSSGGSCPSSHSSLTERHKILHRLLQEGSPSDITTLSVEPDKKDSASASVSVTGQAQGNSSVKLELDASKK
KESKDHQLLRYLLDKDEKDLRSTPNLSLDDVKVKVEKKEQMDPCNTNPTPMTKPAPEEIKLESQNQFTADLDQF
DQLLPTLEKAAQLPGLCETERMDGAVTSVTVKSEILPPSLQSTTARPTSRLNRLPELELEAIDNQFGQPGTGDQ
IPWANNAVTAVNQNKPEDQCISSQLDELLCPPTTVEGRNDEKALLEQLVSFLSGKDETELAELDRALGIDKLVQ
GGGLDVLSERFPPQQATPPLMMEERPNLYSQPYSSPSPTANLTSPFQGMVRQKPSLGTMPVQVTPPRGAFSPGM
GMQPRQTLNRPPAAPNQLRLQLQQRLQGQQQLIHQNRQAILNQFAANAPVGINMRSGMQQQITPQPPLNAQMLA
QRQRELYSQQHRQRQLIQQQRAMLMRQQSFGNNLPPSSGLPVQMGNPRLPQGASQQFPYPPNYGTNPGTPPAST
SPFSQLAANPEAALASRNSMVNRGMTGNLGGQFGTGINPQMQQTVFQYPGSGMVPQGEANFAPSLSPGNSMVPM
PIPPPQTSLLQQTPPASGYQSPDMKAWQQGAMGNNNVFGQAVQNQPAPAQPGVYNNMSITVSMAGGNTNVQNMN
PMMGQMQMSSLQMPGMNTVCPEQINDSALRHTGLYCNQLSSTDLLKTEADGTQQVQQVQVFADVQCTVNLVGGD
PYLNQPGPLGTQKPTAGPQTPQAQQKSLLQQLLTE
>153Tupaia chinensis|L9KW68|Mammalia|SRC1
MSGLGDSSSDPANPDSHKRKGSPCDTLASSTEKRRREQENKYLEELAELLSANISDIDSLSVKPDKCKILKKTV
DQIQLMKRMEQEKSTTDDDVQKSDISSSSQGVIEKESLGPLLLEALDGFFFVVNCEGRIVFVSENVTSYLGYNQ
EELMNTSVYSILHVGDHAEFVKNLLPKSLVNGVPWPQEATRRNSHTFNCRMLIHPPDEPGTESQEACQRYEVMQ
CFTVSQPKSIQEDGEDFQSCLICIARRLPRPPAITGVESFMTKQDTTGKIISIDTSSLRAAGRTGWEDLVRKCI
YAFFQPQGREPSYARQLFQEVMTRGTASSPSYRFILNDGTMLSAHTKCKLCYPQSPDMQPFIMGIHIIDREHSG
LSPQDDTNSGMSIPRVNPSVNPSISPAHGVARSSTLPPSNSNMVSTRVTRQQSSDLHGSNHSNSSNSQGSFGCS
PGNQIVANVALNQGQAGSQSSNPPLNLNNSPMEGTGISLAQFMSPRRQVTSGLATRPRMPNNSFPPNIPTLSSP
VGMTGSSCSNSNRPYSNIPVTSLQGMNEGPNNSVGFSASSPALRQVSSQNSPSRLNIQPAKAESKDNKEIASIL

NEMIQSDNSSSDGKPLDSGLLHNNDRLSDGDSKYSQTSHKLVQLLTTTAEQQLRHADIDTSCKDVLSCTGTSSS
ASANSSGGSCPSSHSSLTERHKILHRLLQEGSPSDITTLSVEPDKKDSASTSVSVAGPGQGNSSIKLELDASKK
KESKDHQLLRYLLDKDEKDLRSTPNLSLDDVKVKVEKKEQMDPCNTNPTPMTKSAPEEIKLESQSQFTADLDQF
DQLLPTLEKAAQLPGLCETDRMDGAVTNVTIKSEILPASLQSTTARPTSRLNRLPELELEAIDNQFGQPGTGDQ
IPWANNSVTAVNQNKPEDPCISSQLDELLCPPTTVEGRNDEKALLEQLVSFLSGKDETELAELDRALGIDKLVQ
GGGLDVLSERFPPQQATPPLMMEERPNLYSQPYSSPSPTANLPSPFQGMVRQKPSLGTMPVQVTPPRGAFSPSM
GMQPRQTLNRPPAAPNQLRLQLQQRLQGQQQLIHQNRQAILNQFAATAPVGINMRSGMQQQITPQPPLNAQMLA
QRQRELYSQQHRQRLIQQQRAMLMRQQSFGNNLPPSSGLPVHMGNPRLPQGAPQQFPYPPNYGTNPGTPPAST
SPFSQLAANPEASLASRNSMVNRGMTGNMGGQFGTGITPQMQQNVFQYPGSGMVPQGEANFAPSLSPGSSMVPM
PIPPPQSSLLQQTPPTSGYQSPDMKAWQQGAMGNNNVFSQAVQNQPAPAQPGVYNNMSITVSMAGGNTNVQNMN
PMMGQMQMSSLQMPGMNAVCPEQINDSALRHTGLYCNQLSSTDLLKTEADGTQQVQQVQVFADVQCTVNLVGGD
PYLNQPGPLGTQKPTSGPQTPQAQQKSLLQQLLTE

>154Felis catus|M3X7H7|Mammalia|SRC1
MSGLGDSSSDPANPDSHKRKGSPCDTLASSTEKRRREQENKYLEELAELLSANISDIDSLSVKPDKCKILKKTV
DQIQLMKRMEQEKSTTDDEVQKSDISSSSQGVIEKESLGPLLLEALDGFFFVVNCEGRIVFVSENVTSYLGYNQ
EELMNTSVYSILHVGDHAEFVKNLLPKSLVNGVPWPQEATRRNSHTFNCRMLIHPPDEPGTENQEACQRYEVMQ
CFTVSQPKSIQEDGEDFQSCLICIARRLPRPPAITGVESFMTKQDTTGKIISIDTSSLRAAGRTGWEDLVRKCI
YAFFQPQGREPSYARQLFQEVMTRGTASSPSYRFILNDGTMLSAHTKCKLCYPQSPDMQPFIMGIHIIDREHSG
LSPQDDTNSGMSISRVNPSVNSSISPAHGVARSSTLPPSSNNMVSPRVNRQQSSDLHSSSHSNSSNSQGSFGCS
PGNQIVASVALNQGQASSQSTNPSLNLNNSPMEGTGISLAQFMSPRRQVSSGLATRPRMPNNSFPPNIPTLSSP
VGMTGTTCNNSNRSYSNIPVTSLQSMNEGPNNSVGFSASSPVLRQMSSQNSPSRLNIQPTKAESKDNKEIASIL
NEMIQSDNSSSDGKPLDSGLLHNNDRLSDGDNKYSQTSHKLVQLLTTTAEQQLRHADIDTSCKEVLSCSGTSGS
ASANSSSGSCPSSHSSLTERHKILHRLLQEGSPSDITTLSVEPDKKDSASTSVSVTGQVQGNSSVKLELDASKK
KESKDHQLLRYLLDKDEKDIRSTPNLSLDDVKVKVEKKEQMDPCNTNPTPMTKPAPEEIKLESQSQFTADLDQF
DQLLPTLEKAAQLPGLCEPERMDGAVTNVTIKSETLPASLQSTTARPTSRLNRLPELELEAIDNQFGQPGTGDQ
IPWANNTVTTVNQNKPEDQCISSQLDELLCPPTTVEGRNDEKALLEQLVSFLSGKDETELAELDRALGIDKLVQ
GGGLDVLSERFPPQQATPPLMMDERPNLYSQPYSSPSPTANLSSPFQGMVRQKPSLGTMPVQVTPPRGAFSPSM
GMQPRQTLNRPPAAPNQLRLQLQQRLQGQQQLIHQNRQAILNQFAANTPVGINMRSGMQQQITPQPPLNAQMLA
QRQRELYSQQHRQRLIQQQRAMLMRQQSFGNNLPPSSGLPVQMGNPRLPQGAPQQFPYPPNYGTNPGTPPAST
SPFSQLAANPESTLANRNSMVNRGMTGNMGGQFGTGINPQMQQNVFQYPGSGMVPQGEASFAPSLSPGSSMVPM
PIPPPQSSLLQQTPPTSGYQSPDMKAWQQGAMGNNNVFSQAVQSQPTPAQPGVYNNMSITVSMAGGNTNVQNMN
PMMGQMQMSSLQMPGMNTVCPEQINDPALRHTGLYCNQLSSTDLLKTETDGTQDKKTEEFFSVVTTD
>155Homo sapiens|Q15788|Mammalia|SRC1
MSGLGDSSSDPANPDSHKRKGSPCDTLASSTEKRRREQENKYLEELAELLSANISDIDSLSVKPDKCKILKKTV
DQIQLMKRMEQEKSTTDDDVQKSDISSSSQGVIEKESLGPLLLEALDGFFFVVNCEGRIVFVSENVTSYLGYNQ
EELMNTSVYSILHVGDHAEFVKNLLPKSLVNGVPWPQEATRRNSHTFNCRMLIHPPDEPGTENQEACQRYEVMQ
CFTVSQPKSIQEDGEDFQSCLICIARRLPRPPAITGVESFMTKQDTTGKIISIDTSSLRAAGRTGWEDLVRKCI
YAFFQPQGREPSYARQLFQEVMTRGTASSPSYRFILNDGTMLSAHTKCKLCYPQSPDMQPFIMGIHIIDREHSG
LSPQDDTNSGMSIPRVNPSVNPSISPAHGVARSSTLPPSNSNMVSTRINRQQSSDLHSSSHSNSSNSQGSFGCS
PGSQIVANVALNQGQASSQSSNPSLNLNNSPMEGTGISLAQFMSPRRQVTSGLATRPRMPNNSFPPNISTLSSP
VGMTSSACNNNNRSYSNIPVTSLQGMNEGPNNSVGFSASSPVLRQMSSQNSPSRLNIQPAKAESKDNKEIASIL
NEMIQSDNSSSDGKPLDSGLLHNNDRLSDGDSKYSQTSHKLVQLLTTTAEQQLRHADIDTSCKDVLSCTGTSNS
ASANSSGGSCPSSHSSLTERHKILHRLLQEGSPSDITTLSVEPDKKDSASTSVSVTGQVQGNSSIKLELDASKK
KESKDHQLLRYLLDKDEKDLRSTPNLSLDDVKVKVEKKEQMDPCNTNPTPMTKPTPEEIKLEAQSQFTADLDQF
DQLLPTLEKAAQLPGLCETDRMDGAVTSVTIKSEILPASLQSATARPTSRLNRLPELELEAIDNQFGQPGTGDQ
IPWTNNTVTAINQSKSEDQCISSQLDELLCPPTTVEGRNDEKALLEQLVSFLSGKDETELAELDRALGIDKLVQ
GGGLDVLSERFPPQQATPPLIMEERPNLYSQPYSSPSPTANLPSPFQGMVRQKPSLGTMPVQVTPPRGAFSPGM
GMQPRQTLNRPPAAPNQLRLQLQQRLQGQQQLIHQNRQAILNQFAATAPVGINMRSGMQQQITPQPPLNAQMLA
QRQRELYSQQHRQRLIQQQRAMLMRQQSFGNNLPPSSGLPVQMGNPRLPQGAPQQFPYPPNYGTNPGTPPAST
SPFSQLAANPEASLANRNSMVSRGMTGNIGGQFGTGINPQMQQNVFQYPGAGMVPQGEANFAPSLSPGSSMVPM
PIPPPQSSLLQQTPPASGYQSPDMKAWQQGAIGNNNVFSQAVQNQPTPAQPGVYNNMSITVSMAGGNTNVQNMN
PMMAQMQMSSLQMPGMNTVCPEQINDPALRHTGLYCNQLSSTDLLKTEADGTQQVQQVQVFADVQCTVNLVGGD
PYLNQPGPLGTQKPTSGPQTPQAQQKSLLQQLLTE

>156Mus musculus|P70365|Mammalia|SRC1
MSGLGDSSSDPANPDSHKRKGSPCDTLASSTEKRRREQENKYLEELAELLSANISDIDSLSVKPDKCKILKKTV
DQIQLMKRMEQEKSTTDDDVQKSDISSSSQGVIEKESLGPLLLEALDGFFFVVNCEGRIVFVSENVTSYLGYNQ
EELMNTSVYSILHVGDHAEFVKNLLPKSLVNGVPWPQEATRRNSHTFNCRMLIHPPEDPGTENQEACQRYEVMQ
CFTVSQPKSIQEDGEDFQSCLICIARRLPRPPAITGVESFMTKQDTTGKIISIDTSSLRAAGRTGWEDLVRKCI
YAFFQPQGREPSYARQLFQEVMTRGTASSPSYRFILNDGTMLSAHTKCKLCYPQSPDMQPFIMGIHIIDREHSG
LSPQDDSNSGMSIPRINPSVNPGISPAHGVTRSSTLPPSNNNMVSARVNRQQSSDLNSSSSHTNSSNNQGNFGC
SPGNQIVANVALNQGQAGSQSSNPSLNLNNSPMEGTGIALSQFMSPRRQANSGLATRARMSNNSFPPNIPTLSS
PVGITSGACNNNNRSYSNIPVTSLQGMNEGPNNSVGFSAGSPVLRQMSSQNSPSRLSMQPAKAESKDSKEIASI
LNEMIQSDNSDNSANEGKPLDSGLLHNNDRLSEGDSKYSQTSHKLVQLLTTTAEQQLRHADIDTSCKDVLSCTG
TSSSASSNPSGGTCPSSHSSLTERHKILHRLLQEGSPSDITTLSVEPEKKDSVPASTAVSVSGQSQGSASIKLE
LDAAKKKESKDHQLLRYLLDKDEKDLRSTPNLCLDDVKVKVEKKEQMDPCNTNPTPMTKPAPEEVKLESQSQFT
ADLDQFDQLLPTLEKAAQLPSLCETDRMDGAVTGVSIKAEVLPASLQPTTARAAPRLSRLPELELEAIDNQFGQ
PGAGDQIPWANNTLTTINQNKPEDQCISSQLDELLCPPTTVEGRNDEKALLEQLVSFLSGKDETELAELDRALG
IDKLVQGGGLDVLSERFPPQQATPPLMMEDRPTLYSQPYSSPSPTAGLSGPFQGMVRQKPSLGAMPVQVTPPRG
TFSPNMGMQPRQTLNRPPAAPNQLRLQLQQRLQGQQQLMHQNRQAILNQFAANAPVGMNMRSGMQQQITPQPPL
NAQMLAQRQRELYSQQHRQRQIIQQQRAMLMRHQSFGNNIPPSSGLPVQMGTPRLPQGAPQQFPYPPNYGTNPG
TPPASTSPFSQLAANPEASLATRSSMVNRGMAGNMGGQFGAGISPQMQQNVFQYPGPGLVPQGEATFAPSLSPG
SSMVPMPVPPPQSSLLQQTPPTSGYQSPDMKAWQQGTMGNNNVFSQAVQSQPAPAQPGVYNNMSITVSMAGGNA
NIQNMNPMMGQMQMSSLQMPGMNTVCSEQMNDPALRHTGLYCNQLSSTDLLKTDADGNQQVQQVQVFADVQCTV
NLVGGDPYLNQPGPLGTQKPTSGPQTPQAQQKSLLQQLLTE
>157Gallus gallus|Q5F393|Aves|SRC1
MSGLGDSSTDPANPDSRKRKGSPCDTAQSNEKRRREQENKYLEELAELLSANIGDIDTLSVKPDKCKILKKTVD
QIQQMKRLEQEKAADDEVQKSDISSSSQGVIEKESLGPLLLEALDGFFFVVNREGRIVFVSENVTSYLGYNQEE
LMNTSVYSILHVGDHTEFVKNLLPKSLVNGVPWPQEATRRNSHTFSCRMLIRPPDEPGTENQEARQRYEVMQCF
TVSQPKSFKEEGEDFQSCLICIARRLPRPAAVTPSESFVTKQDTTGKIISIDTSSLRAAGRTGWEDLVRKCIYA
FFQPQGREPSYAKQLFQEVMTRGTAFSPSYRFTLSDGTVLSAHTKCKLCYPSSPEVQPFIMGIHIIDRDHGMLS
PQENTNSAMALPRVSPSLNPNISPGQGMALPPSIPPSNGSMLATPGNQQPGAGLHSSNSSTSQTSFGCSPGNQI
GANVALSQGQAGPPNSNHNLNLSSSPMNSPGITPPQFMSPRHRVSPGLVPRPRGPSNPFSPTMPTMHSPVGMAN
SGGGGNGGGGGGGSRSFPTAPINSLQGLSEGASTPVGYSTPPPVLRQLGSQSSPGRLGMQPMKAESKDSKEIAS
ILSEMIQSDGSAGDGKPLDSGVLHASERLTEGESKCSQATSHKLVQLLASTAEQQLRHADADTSCKDSLACAGT
TGTLGASNPPSSTCPSSHSSLTERHKILHRLLQEGSPSDITTLAMEHDKKDNVPNPATTPTAPSQLPGTQDIKL
ESDAMKKKDVKDHQLLRYLLDKDEKELAAAPALSLDDVKVKVEKTEQMEPCNTTPVPLAKPPAAEEVKLESQGQ
FAAELEQLDQLLPSLEKAAQLPGLCGPERSESGVAVKSEMLAAPLQPSTAPRAPTGLNPLSSGQGMQAARAPFE
FCDPLEPAQLRPVAFPTTNGSSPRPRAPAEQGRGTVPGPNPFPPDTGMSELEMGVPDHQFGQPGMGEQMPWTDN
SMAPMNRGTLSKPEDPCITSQLDELLCPPTTPEGRNDEKALLEQLVSFLSGKDETELAELDRALGIDKLVQGGG
LEALTERFQPQQATPPLMMEQKPGMYPQPYSSASPTTNLPAAFPGMVRQKPSFGPMPVQVPPPRGAFPTTMAMQ
PRQALSRPPTAPNQLRLQLQQRLQGQQQLIHQNRQAILNQFAANSPVGINMRAGMQQQMTPQPPLNAQMLAQRQ
RELYSQQHRQRQLMQQRAILMRQQSFGNNLPPSAGLPVQMGAARLPQAPPQQFPYPPNYGTSPGNPPTSTSPFS
QLASSPEAALANRGGMVTRGMMGSVGGQFGTGMTPQMQQNIFQYSGSGMGQQNEPTFAPSLSPSSPLMSPQLPP
SQSPMLQPAPPAPGYQSPDMKTWQQGAMGNSNVFSQTGQTQPAPAQQGMYNNMSITVSMAGGNTNVQNMNPMAG
QMQMNSLQMPGMNSMCSEQVNDPALRPTGLYCNQLSSTDLLKTEADGAQQVQQVQVFADVQCTVNLVGDPYLNP
PAPLGAPKAAAVPPTPQGQQKSLLQQLLTE

>158Callithrix jacchus|U3FP54|Mammalia|SRC1
MSGLGDSSSDPANPDSHKRKGSPCDTLASSTEKRRREQENKYLEELAELLSANISDIDSLSVKPDKCKILKKTV
DQIQLMKRMEQEKSTTDDDVQKSDISSSSQGVIEKESLGPLLLEALDGFFFVVNCEGRIVFVSENVTSYLGYNQ
EELMNTSVYSILHVGDHAEFVKNLLPKSLVNGVPWPQEATRRNSHTFNCRMLIHPPDEPGTENQEACQRYEVMQ
CFTVSQPKSIQEDGEDFQSCLICIARRLPRPPAITGVESFMTKQDTTGKIISIDTSSLRAAGRTGWEDLVRKCI
YAFFQPQGREPSYARQLFQEVMTRGTASSPSYRFILNDGTMLSAHTKCKLCYPQSPDMQPFIMGIHIIDREHSG
LSPQDDTNSGMSIPRVNPSVNPNISPAHGVARSSTLPPSSSNMVSTRINRQQSSDLHSSSHSNSSNSQGSFGCS
PGSQIVATVALNQGQASSQSSNPSLNLNNSPMEGTGISLAQFMSPRRQVTSGLATRPRMPNNSFPPNISTLSSP
VGMTSSACNNNNRSYSNIPVTSLQGMNEGPNNSVGFSASSPVLRQISSQNSPNRLNIQPAKAESKDNKEIASIL
NEMIQSDNSSSDGKPLDSGLLHNSDRLSDGDSKYSQTSHKLVQLLTTTAEQQLRHADIDTSCKDVLSCTGTSNS

ASANSSGGSCPSSHSSLTERHKILHRLLQEGSPSDITTLSVEPDKKDSASTSVSVTGQVQGNSSIKLELDASKK
KESKDHQLLRYLLDKDEKDLRSTPNLSLDDVKVKVEKKEQMDPCNTNPTPMTKPAPEEIKLEAQSQFTADLDQF
DQLLPTLEKAAQLPGLCETDRMDGAVTSVTIKSEILPASLQSATARPTSRLNRLPELELEAIDNQFGQPGTGDQ
IPWTNNTVTAINQNKSEDQCISSQLDELLCPPTTVEGRNDEKALLEQLVSFLSGKDETELAELDRALGIDKLVQ
GGGLDVLSERFPPQQATPPLMMEERPSLYSQPYSSPSPTANLPSPFQGMVRQKPSLGTMPVQVTPPRGAFSPGM
GMQPRQTLNRPPAAPNQLRLQLQQRLQGQQQLIHQNRQAILNQFAATAPVGINMRSGMQQQITPQPPLNAQMLA
QRQRELYSQQHRQRQLIQQQRAMLMRQQSFGNNLTPSSGLPVQMGNPRLPQGAPQQFPYPPNYGTNPGTPPAST
SPFSQLAANPEASLANRSSMVSRGMTGNMGGQFGTGINPQMQQNVFQYPGAGMVPQGEANFAPSLSPGSSMVPM
PIPPPQSSLLQQTPPASGYQSPDMKAWQQGAIGNNNVFSQAVQNQPTPAQPGVYNNMSITVSMAGGNTNVQNMN
PMMGQMQMSSLQMPGMNTVCPEQISDPALRHTGLYCNQLSSTDLLKTEADGTQVQQVQVFADVQCTVNLVGGDP
YLNQPGPLGTQKPTSGPQTPQAQQKSLLQQLLTE
>159Grus americana|A0A850TPR3|Aves|SRC1
MSGLGDSSTDPANPDSRKRKGSPCDTAQSNEKRRREQENKYLEELAELLSANIGDIDTLSVKPDKCKILKKTVD
QIQQMKRLEQEKAADDEVQKSDISSSSQGVIEKESLGPLLLEALDGFFFVVNREGRIVFVSENVTSYLGYNQEE
LMNTSVYSILHVGDHAEFVKNLLPKSLVNGVPWPQEATRRNSHTFSCRMLIRPPDEPGAENQEARQRYEVMQCF
TVSQPKSFKEEGEDFQSCLICIARRLPRPAAVTPSESFVTKQDTTGKIISIDTSSLRAAGRTGWEDLVRKCIYA
FFQPQGREPSYAKQLFQEVMTRGTAFSPSYRFTLSDGTVLSAHTKCKLCYPSSPEMQPFIMGIHVIDRDHGMLS
PQENTNSTMSLPRVSPSLNPSISPGQGMALPPSIPPSNGSMLATNVNQQPGAGLHNSNSSTSQTSFGCSPGNQI
GANVALSQGQAGPQNSNHTLNLNSSPMNSPGITPPQFMSPRHRVSPGLVPRPRVPSNPFSPTMPTMHSPVGMAN
SGGGGGGGTAGTRPFPNAPINSMQGLTEGPSTPVGYSTPPPVLRQLSSQSSPGRLAMQPMKAESKDSKEIASIL
SEMIQSDGGSGDGKPLDSGMLHAGDRLSEGDTKCSQATSHKLVQLLATTAEQQLRHADADTSCKDSLACAGTAG
TLASGNPASSTCPSSHSSLTERHKILHRLLQEGSPSDITTLAMEHEKKENAPNPATTPTAQSQLPGNPDIKLEP
DALKKKDVKDHQLLRYLLDKDEKELAAAPALSLDDVKVKVEKTEQMEPCNTAPVPLAKPPAAEEVKLETQGQFA
AELEQLDQLLPSLEKAAQLPSLCGPERSESGVAGVAVKPEMLAAPLQPSTAARAPAGLNPMNSGQGVQPGRTPF
EFCDPLEPTQLRPVPCPTSNGSSPHARGPSQQGRGAVTAPNPFPPDTGMPELEMGVPDHQFGQPGMGEQIPWTD
NSMAAMNRGALNKPEDQCITSQLDELLCPPTTPEGRNDEKALLEQLVSFLSGKDETELAELDRALGIDKLVQGG
GLEALTERFQPQQATPPLMMEQKPGMYPQPYSSASPTANLPGAFPGMVRQKPSFGPMPVQVPPPRGAFPTNMAM
QPRQTLNRPPTAPNQLRLQLQQRLQGQQQLIHQNRQAILNQFAANSPVGINMRAGMQQQITPQPPLNAQMLAQR
QRELYSQQHRQRQLMQQRAILMRQQSFGNNLPPSAGLPVQIGAARLPQAPPQQFPYPPNYGTTPGNPPTSTSPF
SPLASNPEAALANRGSMVNRGMMGGVGGQFGAGMTPQMQQNIFQYSGSGMGQQNEPAFAPSLSPSSPLMSPQLP
PSQSPMLQPAPPTPGYQSPDMKTWQQGAMGNSNVFSQAGQSQPAPAQQGMYNNMSITVSMAGGNTNVQNMNPMA
GQMQMNSLQMPGMNSMCSEQVNDPALRPAGLYCNQLSSTDLLKTEADGAQQVQQVQVCADVQCTVNLVGDPYLN
PPASLGNPKNAAGQP

>160Jacana jacana|A0A7L2Z564|Aves|SRC1
SNSNEKRRREQENKYLEELAELLSANIGDIDTLSVKPDKCKILKKTVDQIQQMKRLEQEKAADDEVQKSDISSS
SQGVIEKESLGPLLLEALDGFFFVVNREGRIVFVSENVTSYLGYNQEELMNTSVYSILHVGDHAEFVKNLLPKS
LVNGVPWPQEATRRNSHTFSCRMLIRPPDEPGAENQEARQRYEVMQCFTVSQPKSFKEEGEDFQSCLICIARRL
PRPAAVTPSESFVTKQDTTGKIISIDTSSLRAAGRTGWEDLVRKCIYAFFQPQGREPSYAKQLFQEVMTRGTAF
SPSYRFTLSDGTVLSAHTKCKLCYPNSPEMQPFIMGIHVIDRDHGMLSPQENTNSAMSLPRVSPSLNPSISPGQ
GMALPTSIPPSNGSMLATNVNQQPGTGLHNSNSSTSQTSFGCSPGNQIGANVALSQGQAGPQNSNHTLNLNSSP
MNSPGITPPQFMSPRHRVSPGLVPRPRVPSNPFSPTMPTMHSPVGMANSGGGGGGGGTGGTRPFPNAPINSMQ
GLSEGPSTPVGYSTPPPVLRQLSSQSSPGRLAMQPMKAESKDSKEIASILSEMIQSDGGSGDGKPLDSGMLHTS
DRLSEGDSKCSQATSHKLVQLLATTAEQQLRHADADTSCKDSLACAGTGGTLASTNPPSSTCPSSHSSLTERHK
ILHRLLQEGSPSDITTLAMEHEKKETAPSAAPTAQSQLPGTPDIKLEPEALKKKDVKDHQLLRYLLDKDEKELA
AAPALSLDDVKVKVEKTEQMEACNTAPVPLAKPPAAEEVKLETQGQFAAELEQLDQLLPSLEKAAQLPGLCAPE
RNESGVTGVTVKPEMLAAPLQPSTAARAPTGLNPMTSGQSVQAGRPPFEFCDPLEPAQLRPVPCPASNGSSPHT
RGASQQGRGTVTAPNPFPPDTGMPELEMGVPDHQFGQPGMGEQIPWTDNGMAAMNRGALNKPEDQCITSQLDEL
LCPPTTPEGRNDEKALLEQLVSFLSGKDETELAELDRALGIDKLVQGGGLEALTERFQPQQATPPLMMEQKPGM
YPQPYSSASPTTNLPSAFPGMVRQKPSFGPVPVQVPPPRGAFPTNMAMQPRQTLNRPPTAPNQLRLQLQQRLQG
QQQLIHQNRQAILNQFAASSPVGINMRAGMQQQITPQPPLNAQMLAQRQRELYSQQHRQRQLMQQRAILMRQQS
FGNNLPPPSAGLPVQIGAARLPQAPPQQFPYPPNYGTTPGNPPTSTSPFSPLASNPEAALANRGGLVNRGMMGG
VGGQFGAGMTPQMQQNIFQYSGSGMGQQSEPAFAPSLSPSSPLMSPQLPPSQSPMLQPAPPTPGYQSPDMKTWQ

QGAMGNSNVFSQAGQNQAAPAQQGMYNNMSITVSMAGGNTNVQNMNPMAGQMQMNSLQMPGMNSMCSEQVNDPA
LRPTGLYCNQLSSTDLLKTEADGAQ
>161Cercocebus atys|A0A2K5N442|Mammalia|SRC2
MSGMGENTSDPSRAETRKRKECPDQLGPSPKRNTEKRNREQENKYIEELAELIFANFNDIDNFNFKPDKCAILK
ETVKQIRQIKEQEKAAAANIDEVQKSDVSSTGQGVIDKDALGPMMLEALDGFFFVVNLEGNVVFVSENVTQYLR
YNQEELMNKSVYSILHVGDHTEFVKNLLPKSIVNGGSWSGEPPRRNSHTFNCRMLVKPLPDSEEEGHDNQEAHQ
KYETMQCFAVSQPKSIKEEGEDLQSCLICVARRVPMKERPVLPSSESFTTRQDLQGKITSLDTSTMRAAMKPGW
EDLVRRCIQKFHAQHEGESVSYAKRHHHEVLRQGLAFSQIYRFSLSDGTLVAAQTKSKLIRSQTTNEPQLVISL
HMLHREQNVCVMNPDLTGQTMGKPLNPISSSSPAHQALCSGNPGQDMTLSSNINFPINGPKEQMGMPMGRFGGS
GGMNHVSGMQATTPQGSNYALKMNSPSQSSPGMNPGQPTSMLSPRHRMSPGVAGSPRIPPSQFSPAGSLHSPVG
VCSSTGNSHSYTNSSLNALQALSEGHGVSLGSSLASPDLKMGNLQNSPVNMNPPPLSKMGSLDSKDCFGLYGEP
SEGTTGQAESSCHPGEQKETNDPNLPPTVSGERADGQSRLHDSKGQTKLLQLLTTKSDQMEPSPLASSLSDTNK
DSTGSLPGSGSTHGTSLKEKHKILHRLLQDSSSPVDLAKLTAEATGKDLSQESSSTAPGSEVTIKQEPVSPKKK
ENALLRYLLDKDDTKDIGLPEITPKLERLDSKTDPASNTKLIAMKTEKEEMSFEPGDQPGSELDNLEEILDDLQ
NSQLPQLFPDTRPGAPAGSVDKQAIINDLMQLTAENSPVTPVGAQKTALRISQSTFNNPRPGQLGRLLPNQNLP
LDITLQSPTGAGPFPPIRNSSPYSVIPQPGMMGNQGMIGNQGNLGNNSTGMIGSSASRPTMPSGEWAPQSSAVR
VTCAATTSAMNRPVQGGMIRNPAASIPMRPSSQPGQRQTLQSQVMNIGPSELEMNMGGPQYSQQQAPPNQTAPW
PESILPIDQASFASQNRQPFGSSPDDLLCPHPAAESPSDEGALLDQLYLALRNFDGLEEIDRALGIPELVSQSQ
AVDPEQFSSQDSNIMLEQKAPVFPQQYASQAQMAQGSYSPMQDPNFHTMGQRPSYATLRMQPRPGLRPTGLVQN
QPNQLRLQLQHRLQAQQNRQPLMNQISNVSNVNLTLRPGVPTQAPINAQMLAQRQREILNQHLRQRQMHQQQQV
QQRTLMMRGQGLNMTPSMVAPSGMPATMSNPRIPQANAQQFPFPPNYGISQQPDPGFTGATTPQSPLMSPRMAH
TQSPMMQQSQANPAYQAPSDMNGWAQGSMGGNSMFSQQSPPHFGQQANTSMYSNNMNISVSMATNTGGMSSMNQ
MTGQISMTSVTSVPTSGLSSMGPEQVNDPALRGGNLFPNQLPGMDMIKQEGDTTRIYCQPVD
>162Cercocebus atys|A0A2K5N484|Mammalia|SRC2
MSGMGENTSDPSRAETRKRKECPDQLGPSPKRNTEKRNREQENKYIEELAELIFANFNDIDNFNFKPDKCAILK
ETVKQIRQIKEQEKAAAANIDEVQKSDVSSTGQGVIDKDALGPMMLEALDGFFFVVNLEGNVVFVSENVTQYLR
YNQEELMNKSVYSILHVGDHTEFVKNLLPKSIVNGGSWSGEPPRRNSHTFNCRMLVKPLPDSEEEGHDNQEAHQ
KYETMQCFAVSQPKSIKEEGEDLQSCLICVARRVPMKERPVLPSSESFTTRQDLQGKITSLDTSTMRAAMKPGW
EDLVRRCIQKFHAQHEGESVSYAKRHHHEVLRQGLAFSQIYRFSLSDGTLVAAQTKSKLIRSQTTNEPQLVISL
HMLHREQNVCVMNPDLTGQTMGKPLNPISSSSPAHQALCSGNPGQDMTLSSNINFPINGPKEQMGMPMGRFGGS
GGMNHVSGMQATTPQGSNYALKMNSPSQSSPGMNPGQPTSMLSPRHRMSPGVAGSPRIPPSQFSPAGSLHSPVG
VCSSTGNSHSYTNSSLNALQALSEGHGVSLGSSLASPDLKMGNLQNSPVNMNPPPLSKMGSLDSKDCFGLYGEP
SEGTTGQAESSCHPGEQKETNDPNLPPTVSGERADGQSRLHDSKGQTKLLQLLTTKSDQMEPSPLASSLSDTNK
DSTGSLPGSGSTHGTSLKEKHKILHRLLQDSSSPVDLAKLTAEATGKDLSQESSSTAPGSEVTIKQEPVSPKKK
ENALLRYLLDKDDTKDIGLPEITPKLERLDSKTDPASNTKLIAMKTEKEEMSFEPGDQPGSELDNLEEILDDLQ
NSQLPQLFPDTRPGAPAGSVDKQAIINDLMQLTAENSPVTPVGAQKTALRISQSRMIGSSASRPTMPSGEWAPQ
SSAVRVTCAATTSAMNRPVQGGMIRNPAASIPMRPSSQPGQRQTLQSQVMNIGPSELEMNMGGPQYSQQQAPPN
QTAPWPESILPIDQASFASQNRQPFGSSPDDLLCPHPAAESPSDEGALLDQLYLALRNFDGLEEIDRALGIPEL
VSQSQAVDPEQFSSQDSNIMLEQKAPVFPQQYASQAQMAQGSYSPMQDPNFHTMGQRPSYATLRMQPRPGLRPT
GLVQNQPNQLRLQLQHRLQAQQNRQPLMNQISNVSNVNLTLRPGVPTQAPINAQMLAQRQREILNQHLRQRQMH
QQQQVQQRTLMMRGQGLNMTPSMVAPSGMPATMSNPRIPQANAQQFPFPPNYGISQQPDPGFTGATTPQSPLMS
PRMAHTQSPMMQQSQANPAYQAPSDMNGWAQGSMGGNSMFSQQSPPHFGQQANTSMYSNNMNISVSMATNTGGM
SSMNQMTGQISMTSVTSVPTSGLSSMGPEQVNDPALRGGNLFPNQLPGMDMIKQEGDTTRIYCQPVD
>163Mandrillus leucophaeus|A0A2K5YY53|Mammalia|SRC2
MSGMGENTSDPSRAETRKRKECPDQLGPSPKRNTEKRNREQENKYIEELAELIFANFNDIDNFNFKPDKCAILK
ETVKQIRQIKEQEKAAAANIDEVQKSDVSSTGQGVIDKDALGPMMLEALDGFFFVVNLEGNVVFVSENVTQYLR
YNQEELMNKSVYSILHVGDHTEFVKNLLPKSIVNGGSWSGEPPRRNSHTFNCRMLVKPLPDSEEEGHDNQEAHQ
KYETMQCFAVSQPKSIKEEGEDLQSCLICVARRVPMKERPVLPSSESFTTRQDLQGKITSLDTSTMRAAMKPGW
EDLVRRCIQKFHAQHEGESVSYAKRHHHEVLRQGLAFSQIYRFSLSDGTLVAAQTKSKLIRSQTTNEPQLVISL
HMLHREQNVCVMNPDLTGQTMGKPLNPISSSSPAHQALCSGNPGQDMTLSSNINFPINGPKEQMGMPMGRFGGS
GGMNHVSGMQATTPQGSNYALKMNSPSQSSPGMNPGQPTSMLSPRHRMSPGVAGSPRIPPSQFSPAGSLHSPVG
VCSSTGNSHSYTNSSLNALQALSEGHGVSLGSSLASPDLKMGNLQNSPVNMNPPPLSKMGSLDSKDCFGLYGEP
SEGTTGQAESSCHPGEQKETNDPNLPPTVSGERADGQSRLHDSKGQTKLLQLLTTKSDQMEPSPLASSLSDTNK
DSTGSLPGSGSTHGTSLKEKHKILHRLLQDSSSPVDLAKLTAEATGKDLSQESSSTAPGSEVTIKQEPVSPKKK

ENALLRYLLDKDDTKDIGLPEITPKLERLDSKTDPASNTKLIAMKTEKEEMSFEPGDQPGSELDNLEEILDDLQ
NSQLPQLFPDTRPGAPAGSVDKQAIINDLMQLTAENSPVTPVGAQKTALRISQSTFNNPRPGQLGRLLPNQNLP
LDITLQSPTGAGPFPPIRNSSPYSVIPQPGMMGNQGMIGNQGNLGNNSTGMIGSSASRPTMPSGEWAPQSSAVR
VTCAATTSAMNRPVQGGMIRNPAASIPMRPSSQPGQRQTLQSQVMNIGPSELEMNMGGPQYSQQQAPPNQTAPW
PESILPIDQASFASQNRQPFGSSPDDLLCPHPAAESPSDEGALLDQLYLALRNFDGLEEIDRALGIPELVSQSQ
AVDPEQFSSQDSNIMLEQKAPVFPQQYASQAQMAQGSYSPMQDPNFHTMGQRPSYATLRMQPRPGLRPTGLVQN
QPNQLRLQLQHRLQAQQNRQPLMNQISNVSNVNLTLRPGVPTQAPINAQMLAQRQREILNQHLRQRQMHQQQQV
QQRTLMMRGQGLNMTPSMVAPSGMPATMSNPRIPQANAQQFPFPPNYGISQQPDPGFTGATTPQSPLMSPRMAH
TQSPMMQQSQANPAYQAPSDMNGWAQGSMGGNSMFSQQSPPHFGQQANTSMYSNNMNISVSMATNTGGMSSMNQ
MTGQISMTSVTSVPTSGLSSMGPEQVNDPALRGGNLFPNQLPGMDMIKQEGDTTRIYCQPVD
>164Mandrillus leucophaeus|A0A2K5YY59|Mammalia|SRC2
MSGMGENTSDPSRAETRKRKECPDQLGPSPKRNTEKRNREQENKYIEELAELIFANFNDIDNFNFKPDKCAILK
ETVKQIRQIKEQEKAAAANIDEVQKSDVSSTGQGVIDKDALGPMMLEALDGFFFVVNLEGNVVFVSENVTQYLR
YNQEELMNKSVYSILHVGDHTEFVKNLLPKSIVNGGSWSGEPPRRNSHTFNCRMLVKPLPDSEEEGHDNQEAHQ
KYETMQCFAVSQPKSIKEEGEDLQSCLICVARRVPMKERPVLPSSESFTTRQDLQGKITSLDTSTMRAAMKPGW
EDLVRRCIQKFHAQHEGESVSYAKRHHHEVLRQGLAFSQIYRFSLSDGTLVAAQTKSKLIRSQTTNEPQLVISL
HMLHREQNVCVMNPDLTGQTMGKPLNPISSSSPAHQALCSGNPGQDMTLSSNINFPINGPKEQMGMPMGRFGGS
GGMNHVSGMQATTPQGSNYALKMNSPSQSSPGMNPGQPTSMLSPRHRMSPGVAGSPRIPPSQFSPAGSLHSPVG
VCSSTGNSHSYTNSSLNALQALSEGHGVSLGSSLASPDLKMGNLQNSPVNMNPPPLSKMGSLDSKDCFGLYGEP
SEGTTGQAESSCHPGEQKETNDPNLPPTVSGERADGQSRLHDSKGQTKLLQLLTTKSDQMEPSPLASSLSDTNK
DSTGSLPGSGSTHGTSLKEKHKILHRLLQDSSSPVDLAKLTAEATGKDLSQESSSTAPGSEVTIKQEPVSPKKK
ENALLRYLLDKDDTKDIGLPEITPKLERLDSKTDPASNTKLIAMKTEKEEMSFEPGDQPGSELDNLEEILDDLQ
NSQLPQLFPDTRPGAPAGSVDKQAIINDLMQLTAENSPVTPVGAQKTALRISQSRMIGSSASRPTMPSGEWAPQ
SSAVRVTCAATTSAMNRPVQGGMIRNPAASIPMRPSSQPGQRQTLQSQVMNIGPSELEMNMGGPQYSQQQAPPN
QTAPWPESILPIDQASFASQNRQPFGSSPDDLLCPHPAAESPSDEGALLDQLYLALRNFDGLEEIDRALGIPEL
VSQSQAVDPEQFSSQDSNIMLEQKAPVFPQQYASQAQMAQGSYSPMQDPNFHTMGQRPSYATLRMQPRPGLRPT
GLVQNQPNQLRLQLQHRLQAQQNRQPLMNQISNVSNVNLTLRPGVPTQAPINAQMLAQRQREILNQHLRQRQMH
QQQQVQQRTLMMRGQGLNMTPSMVAPSGMPATMSNPRIPQANAQQFPFPPNYGISQQPDPGFTGATTPQSPLMS
PRMAHTQSPMMQQSQANPAYQAPSDMNGWAQGSMGGNSMFSQQSPPHFGQQANTSMYSNNMNISVSMATNTGGM
SSMNQMTGQISMTSVTSVPTSGLSSMGPEQVNDPALRGGNLFPNQLPGMDMIKQEGDTTRIYCQPVD
>165Rhinopithecus bieti|A0A2K6MV24|Mammalia|SRC2
MSGMGENTSDPSRAETRKRKECPDQLGPSPKRNTEKRNREQENKYIEELAELIFANFNDIDNFNFKPDKCAILK
ETVKQIRQIKEQEKAAAANIDEVQKSDVSSTGQGVIDKDALGPMMLEALDGFFFVVNLEGNVVFVSENVTQYLR
YNQEELMNKSVYSILHVGDHTEFVKNLLPKSIVNGGSWSGEPPRRNSHTFNCRMLVKPLPDSEEEGHDNQEAHQ
KYETMQCFAVSQPKSIKEEGEDLQSCLICVARRVPMKERPVLPSSESFTTRQDLQGKITSLDTSTMRAAMKPGW
EDLVRRCIQKFHAQHEGESVSYAKRHHHEVLRQGLAFSQIYRFSLSDGTLVAAQTKSKLIRSQTTNEPQLVISL
HMLHREQNVCVMNPDLTGQTMGKPLNPISSSSPAHQALCSGNPGQDMTLSSNINFPINGPKEQMGMPMGRFGGS
GGMNHVSGMQATTPQGSNYALKMNSPSQSSPGMNPGQPTSMLSPRHRMSPGVAGSPRIPPSQFSPAGSLHSPVG
VCSSTGNSHSYTNSSLNALQALSEGHGVSLGSSLASPDLKMGNLQNSPVNMNPPPLSKMGSLDSKDCFGLYGEP
SEGTTGQAESSCHPGEQKETNDPNLPPTVSSERADGQSRLHDSKGQTKLLQLLTTKSDQMEPSPLASSLSDTNK
DSTGSLPGSGSTHGTSLKEKHKILHRLLQDSSSPVDLAKLTAEATGKDLSQESSSTAPGSEVTIKQEPVSPKKK
ENALLRYLLDKDDTKDIGLPEITPKLERLDSKTDPASNTKLIAMKTEKEEMSFEPGDQPGSELDNLEEILDDLQ
NSQLPQLFPDTRPGAPAGSVDKQAIINDLMQLTAENSPVTPVGAQKTALRISQSTFNNPRPGQLGRLLPNQNLP
LDITLQSPTGAGPFPPIRNSSPYSVIPQPGMMGNQGMIGNQGNLGNNSTGMIGSSASRPTMPSGEWAPQSSAVR
VTCAATTSAMNRPVQGGMIRNPAASIPMRPSSQPGQRQTLQSQVMNIGPSELEMNIGGPQYSQQQAPPNQTAPW
PESILPIDQASFASQNRQPFGSSPDDLLCPHPAAESPSDEGALLDQLYLALRNFDGLEEIDRALGIPELVSQSQ
AVDPEQFSSQDSNIMLEQKAPVFPQQYASQAQMAQGSYSPMQDPNFHTMGQRPSYATLRMQPRPGLRPTGLVQN
QPNQLRLQLQHRLQAQQNRQPLMNQISNVSNVNLTLRPGVPTQAPINAQMLAQRQREILNQHLRQRQMHQQQQV
QQRTLMMRGQGLNMTPSMVAPSGMPATMSNPRIPQANAQQFPFPPNYGISQQPDPGFTGATTPQSPLMSPRMAH
TQSPMMQQSQANPAYQAPSDMNGWAQGSMGGNSMFSQQSPPHFGQQANTSMYSNNMNISVSMATNTGGMSSMNQ
MTGQISMTSVTSVPTSGLSSMGPEQVNDPALRGGNLFPNQLPGMDMIKQEGDTTRMLTRYCKKGTLIHC
>166Rhinopithecus bieti|A0A2K6MV37|Mammalia|SRC2
MSGMGENTSDPSRAETRKRKECPDQLGPSPKRNTEKRNREQENKYIEELAELIFANFNDIDNFNFKPDKCAILK
ETVKQIRQIKEQEKAAAANIDEVQKSDVSSTGQGVIDKDALGPMMLEALDGFFFVVNLEGNVVFVSENVTQYLR

YNQEELMNKSVYSILHVGDHTEFVKNLLPKSIVNGGSWSGEPPRRNSHTFNCRMLVKPLPDSEEEGHDNQEAHQ
KYETMQCFAVSQPKSIKEEGEDLQSCLICVARRVPMKERPVLPSSESFTTRQDLQGKITSLDTSTMRAAMKPGW
EDLVRRCIQKFHAQHEGESVSYAKRHHHEVLRQGLAFSQIYRFSLSDGTLVAAQTKSKLIRSQTTNEPQLVISL
HMLHREQNVCVMNPDLTGQTMGKPLNPISSSSPAHQALCSGNPGQDMTLSSNINFPINGPKEQMGMPMGRFGGS
GGMNHVSGMQATTPQGSNYALKMNSPSQSSPGMNPGQPTSMLSPRHRMSPGVAGSPRIPPSQFSPAGSLHSPVG
VCSSTGNSHSYTNSSLNALQALSEGHGVSLGSSLASPDLKMGNLQNSPVNMNPPPLSKMGSLDSKDCFGLYGEP
SEGTTGQAESSCHPGEQKETNDPNLPPTVSSERADGQSRLHDSKGQTKLLQLLTTKSDQMEPSPLASSLSDTNK
DSTGSLPGSGSTHGTSLKEKHKILHRLLQDSSSPVDLAKLTAEATGKDLSQESSSTAPGSEVTIKQEPVSPKKK
ENALLRYLLDKDDTKDIGLPEITPKLERLDSKTDPASNTKLIAMKTEKEEMSFEPGDQPGSELDNLEEILDDLQ
NSQLPQLFPDTRPGAPAGSVDKQAIINDLMQLTAENSPVTPVGAQKTALRISQSTFNNPRPGQLGRLLPNQNLP
LDITLQSPTGAGPFPPIRNSSPYSVIPQPGMMGNQGMIGNQGNLGNNSTGMIGSSASRPTMPSGEWAPQSSAVR
VTCAATTSAMNRPVQGGMIRNPAASIPMRPSSQPGQRQTLQSQVMNIGPSELEMNIGGPQYSQQQAPPNQTAPW
PESILPIDQASFASQNRQPFGSSPDDLLCPHPAAESPSDEGALLDQLYLALRNFDGLEEIDRALGIPELVSQSQ
AVDPEQFSSQDSNIMLEQKAPVFPQQYASQAQMAQGSYSPMQDPNFHTMGQRPSYATLRMQPRPGLRPTGLVQN
QPNQLRLQLQHRLQAQQNRQPLMNQISNVSNVNLTLRPGVPTQAPINAQMLAQRQREILNQHLRQRQMHQQQQV
QQRTLMMRGQGLNMTPSMVAPSGMPATMSNPRIPQANAQQFPFPPNYGISQQPDPGFTGATTPQSPLMSPRMAH
TQSPMMQQSQANPAYQAPSDMNGWAQGSMGGNSMFSQQSPPHFGQQANTSMYSNNMNISVSMATNTGGMSSMNQ
MTGQISMTSVTSVPTSGLSSMGPEQVNDPALRGGNLFPNQLPGMDMIKQEGDTTRKYC
>167Rhinopithecus roxellana|A0A2K6QWW9|Mammalia|SRC2
MSGMGENTSDPSRAETRKRKECPDQLGPSPKRNTEKRNREQENKYIEELAELIFANFNDIDNFNFKPDKCAILK
ETVKQIRQIKEQEKAAAANIDEVQKSDVSSTGQGVIDKDALGPMMLEALDGFFFVVNLEGNVVFVSENVTQYLR
YNQEELMNKSVYSILHVGDHTEFVKNLLPKSIVNGGSWSGEPPRRNSHTFNCRMLVKPLPDSEEEGHDNQEAHQ
KYETMQCFAVSQPKSIKEEGEDLQSCLICVARRVPMKERPVLPSSESFTTRQDLQGKITSLDTSTMRAAMKPGW
EDLVRRCIQKFHAQHEGESVSYAKRHHHEVLRQGLAFSQIYRFSLSDGTLVAAQTKSKLIRSQTTNEPQLVISL
HMLHREQNVCVMNPDLTGQTMGKPLNPISSSSPAHQALCSGNPGQDMTLSSNINFPINGPKEQMGMPMGRFGGS
GGMNHVSGMQATTPQGSNYALKMNSPSQSSPGMNPGQPTSMLSPRHRMSPGVAGSPRIPPSQFSPAGSLHSPVG
VCSSTGNSHSYTNSSLNALQALSEGHGVSLGSSLASPDLKMGNLQNSPVNMNPPPLSKMGSLDSKDCFGLYGEP
SEGTTGQAESSCHPGEQKETNDPNLPPTVSSERADGQSRLHDSKGQTKLLQLLTTKSDQMEPSPLASSLSDTNK
DSTGSLPGSGSTHGTSLKEKHKILHRLLQDSSSPVDLAKLTAEATGKDLSQESSSTAPGSEVTIKQEPVSPKKK
ENALLRYLLDKDDTKDIGLPEITPKLERLDSKTDPASNTKLIAMKTEKEEMSFEPGDQPGSELDNLEEILDDLQ
NSQLPQLFPDTRPGAPAGSVDKQAIINDLMQLTAENSPVTPVGAQKTALRISQSTFNNPRPGQLGRLLPNQNLP
LDITLQSPTGAGPFPPIRNSSPYSVIPQPGMMGNQGMIGNQGNLGNNSTGMIGSSASRPTMPSGEWAPQSSAVR
VTCAATTSAMNRPVQGGMIRNPAASIPMRPSSQPGQRQTLQSQVMNIGPSELEMNIGGPQYSQQQAPPNQTAPW
PESILPIDQASFASQNRQPFGSSPDDLLCPHPAAESPSDEGALLDQLYLALRNFDGLEEIDRALGIPELVSQSQ
AVDPEQFSSQDSNIMLEQKAPVFPQQYASQAQMAQGSYSPMQDPNFHTMGQRPSYATLRMQPRPGLRPTGLVQN
QPNQLRLQLQHRLQAQQNRQPLMNQISNVSNVNLTLRPGVPTQAPINAQMLAQRQREILNQHLRQRQMHQQQQV
QQRTLMMRGQGLNMTPSMVAPSGMPATMSNPRIPQANAQQFPFPPNYGISQQPDPGFTGATTPQSPLMSPRMAH
TQSPMMQQSQANPAYQAPSDMNGWAQGSMGGNSMFSQQSPPHFGQQANTSMYSNNMNISVSMATNTGGMSSMNQ
MTGQISMTSVTSVPTSGLSSMGPEQVNDPALRGGNLFPNQLPGMDMIKQEGDTTRMLTRYCKKGTLIHC
>168Rhinopithecus roxellana|A0A2K6QWY5|Mammalia|SRC2
MSGMGENTSDPSRAETRKRKECPDQLGPSPKRNTEKRNREQENKYIEELAELIFANFNDIDNFNFKPDKCAILK
ETVKQIRQIKEQEKAAAANIDEVQKSDVSSTGQGVIDKDALGPMMLEALDGFFFVVNLEGNVVFVSENVTQYLR
YNQEELMNKSVYSILHVGDHTEFVKNLLPKSIVNGGSWSGEPPRRNSHTFNCRMLVKPLPDSEEEGHDNQEAHQ
KYETMQCFAVSQPKSIKEEGEDLQSCLICVARRVPMKERPVLPSSESFTTRQDLQGKITSLDTSTMRAAMKPGW
EDLVRRCIQKFHAQHEGESVSYAKRHHHEVLRQGLAFSQIYRFSLSDGTLVAAQTKSKLIRSQTTNEPQLVISL
HMLHREQNVCVMNPDLTGQTMGKPLNPISSSSPAHQALCSGNPGQDMTLSSNINFPINGPKEQMGMPMGRFGGS
GGMNHVSGMQATTPQGSNYALKMNSPSQSSPGMNPGQPTSMLSPRHRMSPGVAGSPRIPPSQFSPAGSLHSPVG
VCSSTGNSHSYTNSSLNALQALSEGHGVSLGSSLASPDLKMGNLQNSPVNMNPPPLSKMGSLDSKDCFGLYGEP
SEGTTGQAESSCHPGEQKETNDPNLPPTVSSERADGQSRLHDSKGQTKLLQLLTTKSDQMEPSPLASSLSDTNK
DSTGSLPGSGSTHGTSLKEKHKILHRLLQDSSSPVDLAKLTAEATGKDLSQESSSTAPGSEVTIKQEPVSPKKK
ENALLRYLLDKDDTKDIGLPEITPKLERLDSKTDPASNTKLIAMKTEKEEMSFEPGDQPGSELDNLEEILDDLQ
NSQLPQLFPDTRPGAPAGSVDKQAIINDLMQLTAENSPVTPVGAQKTALRISQSTFNNPRPGQLGRLLPNQNLP
LDITLQSPTGAGPFPPIRNSSPYSVIPQPGMMGNQGMIGNQGNLGNNSTGMIGSSASRPTMPSGEWAPQSSAVR
VTCAATTSAMNRPVQGGMIRNPAASIPMRPSSQPGQRQTLQSQVMNIGPSELEMNIGGPQYSQQQAPPNQTAPW

PESILPIDQASFASQNRQPFGSSPDDLLCPHPAAESPSDEGALLDQLYLALRNFDGLEEIDRALGIPELVSQSQ
AVDPEQFSSQDSNIMLEQKAPVFPQQYASQAQMAQGSYSPMQDPNFHTMGQRPSYATLRMQPRPGLRPTGLVQN
QPNQLRLQLQHRLQAQQNRQPLMNQISNVSNVNLTLRPGVPTQAPINAQMLAQRQREILNQHLRQRQMHQQQQV
QQRTLMMRGQGLNMTPSMVAPSGMPATMSNPRIPQANAQQFPFPPNYGISQQPDPGFTGATTPQSPLMSPRMAH
TQSPMMQQSQANPAYQAPSDMNGWAQGSMGGNSMFSQQSPPHFGQQANTSMYSNNMNISVSMATNTGGMSSMNQ
MTGQISMTSVTSVPTSGLSSMGPEQVNDPALRGGNLFPNQLPGMDMIKQEGDTTRKYC
>169Ursus maritimus|A0A384DPQ8|Mammalia|SRC2
MSGMGENTSDPSRAETRKRKECPDQLGPSPKRSTEKRNREQENKYIEELAELIFANFNDI
DNFNFKPDKCAILKETVKQIRQIKEQEKAAAANVDEVQKSDVSSTGQGVIDKDALGPMML
EALDGFFFVVNLEGNVVFVSENVTQYLRYNQEELMNKSVYSILHVGDHTEFVKNLLPKSI
VNGGSWSGEPPRRNSHTFNCRMLVKPLPESEEEGHDNQEAHQKYETMQCFAVSQPKSIKE
EGEDLQSCLICVARRVPMKERPVLPSSESFTTRQDLQGKITSLDTSTMRAAMKPGWEDLV
RRCIQKFHAQHEGESVSYAKRHHHEVLRQGLAFSQIYRFSLSDGTLVAAQTKSKLIRSQT
TNEPQLVISLHMLHREQNVCVMNPDLTGQAMGKPLNPISSSSPAHQAMCSGNPGQDMTLG
SNINFPINGPKEQMGMPMGRFGGSGGMNHVSSMQATTPQGSNYALKMNSPSQSSPGMNPG
QPNSMLSPRHRMSPGVAGSPRIPPSQFSPAGSLHSPVGVCSSTGNSHSYTNSSLNALQAL
SEGHGVSLGSSLASPDLKMGNLQNSPVNMNPPPLSKMGSLDSKDCFGLYGEPSEGTTGQA
ESSCHPGEQKETNDSNMPQAVSAERADGQNRLHDSKGQTKLLQLLTTKSDQMEPSPLSSS
LSDTNKDSTASLPGSGSTHGTSLKEKHKILHRLLQDSSSPVDLAKLTAEATGKELSQEAN
STAPGSEVTIKQEPVSPKKKENALLRYLLDKDDTKDIGLPEITPKLERLDSKTDPASNTK
LIAMKTEKEEMSFEPSEQPGSELDNLEEILDDLQNSQLPQLFPDTRPGAPAGSVDKQAII
NDLMQLTAENSPVTPVGAQKTALRISQSTFNNPRPGQLGRLLPNQNLPLDITLQSPTGAG
PFPPIRNSSPYSVIPQPGMMGNQGMIGNQGNLGNSSTGMIGSNASRPTMPSGEWAPQSSA
VRVTCAATTSAMNRPIQGGMYFTLYYIAEISLTTSCKVFGFLSSPFLGPSELEMNMGGPQ
YSQQQAPPNQTAPWPESILPIDQASFASQNRQPFGNSPDDLLCPHPAAESPSDEGALLDQ
LYLALRNFDGLEEIDRALGIPELVSQSQAVDPEQFSSQDSNIMLEQKAAVFPQQYASQAQ
MAQGSYPPMQDPNFHTMGQRPSYATLRMQPRPGLRPAGLVQNQPNQLRLQLQHRLQAQQN
RQPLMNQISNVSNVNLTLRPGVPTQAPINAQMLAQRQREILNQHLRQRQMHQQQQVQQRT
LMMRGQGLNMTSSMVAPGGLPATLSNPRMPQANAQQFPFPPNYGISQQPDPGFTGATTPQ
SPLMSPRMAHTQSPMMQQSQANPAYQASSDMNGWAQGNMGGNSMFSQQSPPHFGQQANTS
MYNNNMNINVSMATNTGGMSNMNQMTGQISMTSVTSVPTSGLSSMGPEQVNDPALRGGNL
FPNQLPGMEMIKQEGDASRKYC
>170Callorhinus ursinus|A0A3Q7NXT7|Mammalia|SRC2
MSGMGENTSDPSRAETRKRKECPDQLGPSPKRSTEKRNREQENKYIEELAELIFANFNDIDNFNFKPDKCAILK
ETVKQIRQIKEQEKAAAANVDEVQKSDVSSTGQGVIDKDALGPMMLEALDGFFFVVNLEGNVVFVSENVTQYLR
YNQEELMNKSVYSILHVGDHTEFVKNLLPKSIVNGGSWSGEPPRRNSHTFNCRMLVKPLPDSEEEGHDNQDAHQ
KYETMQCFAVSQPKSIKEEGEDLQSCLICVARRVPMKERPVLPSSESFTTRQDLQGKITSLDTSTMRAAMKPGW
EDLVRRCIQKFHAQHEGESVSYAKRHHHEVLRQGLAFSQIYRFSLSDGTLVAAQTKSKLIRSQTTNEPQLVISL
HMLHREQNVCVMNPDLTGQAMGKPLNPISSSSPAHQAMCSGNPGQDMTLGSNINFPINGPKEQMGMPMGRFGGS
GGMNHVSSMQATTPQGSNYALKMNSPSQSSPGMNPGQPNSMLSPRHRMSPGVAGSPRIPPSQFSPAGSLHSPVG
VCSSTGNSHSYTNSSLNALQALSEGHGVSLGSSLASPDLKLGNLQNSPVNMNPPPLSKMGSLDSKDCFGLYGEP
AEGTTGQAESTCRPGEQKEADDSSMPQAVSGERADAQNRLHDSKGQTKLLQLLTTKSDQMEPSPLPSSLSDTNK
DSTASLPGPGSTHGTSLKEKHKILHRLLQDSSSPVDLAKLTAEATGKELSQEANSTASGSEVTIKQEPVSPKKK
ENALLRYLLDKDDTKDIGLPEMTPKLERLDSKTDPASNTKLIAMKTEKEEMSFEPSEQPGSELDNLEEILDDLQ
NSQLPQLFPDTRPGAPAGSVDKQAIINDLMQLTAENSPVTPVGAQKTALRISQSRMMGGNASRPTMPSGEWAPQ
SSAVRVTCAAPTSAMNRPIQGGMIRNPTASIPMRPNSQPGQRQMLQSQVMNLGPSELEMNMGGPQYSQQQAPPN
QTAPWPESILPIDQASFASQNRQPFGSSPDDLLCPHPAAESPSDEGALLDQLYLALRNFDGLEEIDRALGIPEL
VSQSQAVDPEQFSSQDSNIMLEQKAAVFPQQYASQAQMAQGSYPPMQDPSFHTMGQRPSYATLRMQPRPGLRPT
GLVQNQPNQLRLQLQHRLQAQQNRQPLMNQISNVSNVNLTLRPGVPTQAPINAQMLAQRQREILNQHLRQRQMH
QQQQVQQRTLMMRGQGLNMTSSMVAPGSLPATLSNPRMPQANAQQFPFPPNYGISQQPDPGFTGATTPQSPLMS
PRMAHTQSPMMQQSQANPAYQASSDMNGWAQGNMGGNSMFSQQSPPHFGQQANTSMYNNNMNINVSMATNTGGM
SNMNQMTGQISMTSVTSVPTSGLSSMGPEQVNDPALRGGNLFPNQLPGMDMIKQEGDASRKYC

>171Callorhinus ursinus|A0A3Q7NZ27|Mammalia|SRC2

MSGMGENTSDPSRAETRKRKECPDQLGPSPKRSTEKRNREQENKYIEELAELIFANFNDIDNFNFKPDKCAILK
ETVKQIRQIKEQEKAAAANVDEVQKSDVSSTGQGVIDKDALGPMMLEALDGFFFVVNLEGNVVFVSENVTQYLR
YNQEELMNKSVYSILHVGDHTEFVKNLLPKSIVNGGSWSGEPPRRNSHTFNCRMLVKPLPDSEEEGHDNQDAHQ
KYETMQCFAVSQPKSIKEEGEDLQSCLICVARRVPMKERPVLPSSESFTTRQDLQGKITSLDTSTMRAAMKPGW
EDLVRRCIQKFHAQHEGESVSYAKRHHHEVLRQGLAFSQIYRFSLSDGTLVAAQTKSKLIRSQTTNEPQLVISL
HMLHREQNVCVMNPDLTGQAMGKPLNPISSSSPAHQAMCSGNPGQDMTLGSNINFPINGPKEQMGMPMGRFGGS
GGMNHVSSMQATTPQGSNYALKMNSPSQSSPGMNPGQPNSMLSPRHRMSPGVAGSPRIPPSQFSPAGSLHSPVG
VCSSTGNSHSYTNSSLNALQALSEGHGVSLGSSLASPDLKLGNLQNSPVNMNPPPLSKMGSLDSKDCFGLYGEP
AEGTTGQAESTCRPGEQKEADDSSMPQAVSGERADAQNRLHDSKGQTKLLQLLTTKSDQMEPSPLPSSLSDTNK
DSTASLPGPGSTHGTSLKEKHKILHRLLQDSSSPVDLAKLTAEATGKELSQEANSTASGSEVTIKQEPVSPKKK
ENALLRYLLDKDDTKDIGLPEMTPKLERLDSKTDPASNTKLIAMKTEKEEMSFEPSEQPGSELDNLEEILDDLQ
NSQLPQLFPDTRPGAPAGSVDKQAIINDLMQLTAENSPVTPVGAQKTALRISQSTFNNPRPGQLGRLLPNQNLP
LDITLQSPTGAGPFPPIRNSSPYSVIPQPGMMGNQGMIGNQGNLGNSSTGMMGGNASRPTMPSGEWAPQSSAVR
VTCAAPTSAMNRPIQGGMIRNPTASIPMRPNSQPGQRQMLQSQVMNLGPSELEMNMGGPQYSQQQAPPNQTAPW
PESILPIDQASFASQNRQPFGSSPDDLLCPHPAAESPSDEGALLDQLYLALRNFDGLEEIDRALGIPELVSQSQ
AVDPEQFSSQDSNIMLEQKAAVFPQQYASQAQMAQGSYPPMQDPSFHTMGQRPSYATLRMQPRPGLRPTGLVQN
QPNQLRLQLQHRLQAQQNRQPLMNQISNVSNVNLTLRPGVPTQAPINAQMLAQRQREILNQHLRQRQMHQQQQV
QQRTLMMRGQGLNMTSSMVAPGSLPATLSNPRMPQANAQQFPFPPNYGISQQPDPGFTGATTPQSPLMSPRMAH
TQSPMMQQSQANPAYQASSDMNGWAQGNMGGNSMFSQQSPPHFGQQANTSMYNNNMNINVSMATNTGGMSNMNQ
MTGQISMTSVTSVPTSGLSSMGPEQVNDPALRGGNLFPNQLPGMDMIKQEGDASRKYC
>172Canis lupus familiaris|A0A5F4DEU8|Mammalia|SRC2
MSGMGENTSDPSRAETRKRKECPDQLGPSPKRSTEKRNREQENKYIEELAELIFANFNDI
DNFNFKPDKCAILKETVKQIRQIKEQEKAAAANIDEVQKSDVSSTGQGVIDKDALGPMML
EALDGFFFVVNLEGNVVFVSENVTQYLRYNQEELMNKSVYGILHVGDHTEFVKNLLPKSI
VNGGSWSGEPPRRNSHTFNCRMLVKPLPDSEEEGHDNQEAHQKYETMQCFAVSQPKSIKE
EGEDLQSCLICVARRVPMKERPVLPSSESFTTRQDLQGKITSLDTSTMRAAMKPGWEDLV
RRCIQKFHAQHEGESVSYAKRHHHEVLRQGLAFSQIYRFSLSDGTLVAAQTKSKLIRSQT
TNEPQLVISLHMLHREQNVCVMNPDLTGQAMGKPLNPISSSSPAHQAMCSGNPGQDMTLS
SNINFPINGPKEQMGMPMGRFGGSGGMNHVSSMQATTPQGSNYALKMNSPSQSSPGMNPG
QPNSMLSPRHRMSPGVAGSPRIPPSQFSPAGSLHSPVGVCSSTGNSHSYTNSSLNALQAL
SEGHGVSLGSSLASPDLKMGNLQNSPVNMNPPPLSKMGSLDSKDCFGLYGEPSEGTTGPA
ESSCHPGEQKETNDSNMPPAVSSERADGQNRLHDSKGQTKLLQLLTTKSDQMEPSPLSSS
LSDANKDSTAGLPGSGSTHGTSLKEKHKILHRLLQDSSSPVDLAKLTAEATGKELSQEAS
STAPGSEVTIKQEPVSPKKKENALLRYLLDKDDTKDIGLPEITPKLERLDSKTDPASNTK
LIAMKTEKEEMSFEPSDQPGSELDNLEEILDDLQNSQLPQLFPDTRPGAPAGSVDKQAII
NDLMQLTAENSPVTPVGAQKTALRISQSTFNNPRPGQLGRLLPNQNLPLDITLQSPTGAG
PFPPIRNNSPYSVIPQPGMMGNQGMIGNQGNLGNSSTGMIGSNASRPTMPSGEWAPQSSA
VRVTCAAATTSAMNRPIQGGMIRNPTASIPMRPNSQPGQRQMLQSQVMNIGPSELEMNMG
GPQYSQQQAPPNQTAPWPESILPIDQASFANQNRQPFGSSPDDLLCPHPAAESPSDEGAL
LDQLYLALRNFDGLEEIDRALGIPELVSQSQAVDPEQFSSQDSNIMLEQKAAVFPQQYAS
QAQMAQGSYPPMQDPNFHTMGQRPSYATLRMQPRPGLRPTGLVQNQPNQLRLQLQHRLQA
QQAPINAQMLAQRQREILNQHLRQRQMHQQQQVQQRTLMMRGQGLNMTSSMVAPGGLPAT
LSNPRMPQANAQQFPFPPNYGISQQPDPGFTGATTPQSPLMSPRMAHTQSPMMQQSQANP
AYQASSDMNGWAQGNMGGNSMFSQQSPPHFGQQANTSMYNNNMNINVSMATNTGGMNNMN
QMTGQISMTSVTSVPTSGLSSMGPEQVNDPALRGGNLFPNQLPGMDMIKQEGDASRIISR
TRPEDKVHVASAKIKPQPVNDCTRKWPPKDLSPQTIQVSAHVFKPHRFGQPRWLSGLAPP
SAQGLILETRDQVPRRASCMEPASPSACVSASLSLSLSPSHE
>173Canis lupus familiaris|A0A5F4DGH2|Mammalia|SRC2
MSGMGENTSDPSRAETRKRKECPDQLGPSPKRSTEKRNREQENKYIEELAELIFANFNDI
DNFNFKPDKCAILKETVKQIRQIKEQEKAAAANIDEVQKSDVSSTGQGVIDKDALGPMML
EALDGFFFVVNLEGNVVFVSENVTQYLRYNQEELMNKSVYGILHVGDHTEFVKNLLPKSI
VNGGSWSGEPPRRNSHTFNCRMLVKPLPDSEEEGHDNQEAHQKYETMQCFAVSQPKSIKE
EGEDLQSCLICVARRVPMKERPVLPSSESFTTRQDLQGKITSLDTSTMRAAMKPGWEDLV
RRCIQKFHAQHEGESVSYAKRHHHEVLRQGLAFSQIYRFSLSDGTLVAAQTKSKLIRSQT

TNEPQLVISLHMLHREQNVCVMNPDLTGQAMGKPLNPISSSSPAHQAMCSGNPGQDMTLS
SNINFPINGPKEQMGMPMGRFGGSGGMNHVSSMQATTPQGSNYALKMNSPSQSSPGMNPG
QPNSMLSPRHRMSPGVAGSPRIPPSQFSPAGSLHSPVGVCSSTGNSHSYTNSSLNALQAL
SEGHGVSLGSSLASPDLKMGNLQNSPVNMNPPPLSKMGSLDSKDCFGLYGEPSEGTTGPA
ESSCHPGEQKETNDSNMPPAVSSERADGQNRLHDSKGQTKLLQLLTTKSDQMEPSPLSSS
LSDANKDSTAGLPGSGSTHGTSLKEKHKILHRLLQDSSSPVDLAKLTAEATGKELSQEAS
STAPGSEVTIKQEPVSPKKKENALLRYLLDKDDTKDIGLPEITPKLERLDSKTDPASNTK
LIAMKTEKEEMSFEPSDQPGSELDNLEEILDDLQNSQLPQLFPDTRPGAPAGSVDKQAII
NDLMQLTAENSPVTPVGAQKTALRISQSTFNNPRPGQLGRLLPNQNLPLDITLQSPTGAG
PFPPIRNNSPYSVIPQPGMMGNQGMIGNQGNLGNSSTGMIGSNASRPTMPSGEWAPQSSA
VRVTCAAATTSAMNRPIQGGMIRNPTASIPMRPNSQPGQRQMLQSQVMNIGPSELEMNMG
GPQYSQQQAPPNQTAPWPESILPIDQASFANQNRQPFGSSPDDLLCPHPAAESPSDEGAL
LDQLYLALRNFDGLEEIDRALGIPELVSQSQAVDPEQFSSQDSNIMLEQKAAVFPQQYAS
QAQMAQGSYPPMQDPNFHTMGQRPSYATLRMQPRPGLRPTGLVQNQPNQLRLQLQHRLQA
QQNRQPLMNQISNVSNVNLTLRPGVPTQAPINAQMLAQRQREILNQHLRQRQMHQQQQVQ
QRTLMMRGQGLNMTSSMVAPGGLPATLSNPRMPQANAQQFPFPPNYGISQQPDPGFTGAT
TPQSPLMSPRMAHTQSPMMQQSQANPAYQASSDMNGWAQGNMGGNSMFSQQSPPHFGQQA
NTSMYNNNMNINVSMATNTGGMNNMNQMTGQISMTSVTSVPTSGLSSMGPEQVNDPALRG
GNLFPNQLPGMDMIKQEGDASRIISRTRPEDKVHVASAKIKPQPVNDCTRKWPPKDLSPQ
TIQVSAHVFKPHRFGQPRWLSGLAPPSAQGLILETRDQVPRRASCMEPASPSACVSASLS
LSLSPSHE
>174Canis lupus familiaris|A0A5F4DJ58|Mammalia|SRC2
MSGMGENTSDPSRAETRKRKECPDQLGPSPKRSTEKRNREQENKYIEELAELIFANFNDI
DNFNFKPDKCAILKETVKQIRQIKEQEKAAAANIDEVQKSDVSSTGQGVIDKDALGPMML
EALDGFFFVVNLEGNVVFVSENVTQYLRYNQEELMNKSVYGILHVGDHTEFVKNLLPKSI
VNGGSWSGEPPRRNSHTFNCRMLVKPLPDSEEEGHDNQEAHQKYETMQCFAVSQPKSIKE
EGEDLQSCLICVARRVPMKERPVLPSSESFTTRQDLQGKITSLDTSTMRAAMKPGWEDLV
RRCIQKFHAQHEGESVSYAKRHHHEVLRQGLAFSQIYRFSLSDGTLVAAQTKSKLIRSQT
TNEPQLVISLHMLHREQNVCVMNPDLTGQAMGKPLNPISSSSPAHQAMCSGNPGQDMTLS
SNINFPINGPKEQMGMPMGRFGGSGGMNHVSSMQATTPQGSNYALKMNSPSQSSPGMNPG
QPNSMLSPRHRMSPGVAGSPRIPPSQFSPAGSLHSPVGVCSSTGNSHSYTNSSLNALQAL
SEGHGVSLGSSLASPDLKMGNLQNSPVNMNPPPLSKMGSLDSKDCFGLYGEPSEGTTGPA
ESSCHPGEQKETNDSNMPPAVSSERADGQNRLHDSKGQTKLLQLLTTKSDQMEPSPLSSS
LSDANKDSTAGLPGSGSTHGTSLKEKHKILHRLLQDSSSPVDLAKLTAEATGKELSQEAS
STAPGSEVTIKQEPVSPKKKENALLRYLLDKDDTKDIGLPEITPKLERLDSKTDPASNTK
LIAMKTEKEEMSFEPSDQPGSELDNLEEILDDLQNSQLPQLFPDTRPGAPAGSVDKQAII
NDLMQLTAENSPVTPVGAQKTALRISQSTFNNPRPGQLGRLLPNQNLPLDITLQSPTGAG
PFPPIRNNSPYSVIPQPGMMGNQGMIGNQGNLGNSSTGMIGSNASRPTMPSGEWAPQSSA
VRVTCAAATTSAMNRPIQGGMIRNPTASIPMRPNSQPGQRQMLQSQVMNIGPSELEMNMG
GPQYSQQQAPPNQTAPWPESILPIDQASFANQNRQPFGSSPDDLLCPHPAAESPSDEGAL
LDQLYLALRNFDGLEEIDRALGIPELVSQSQAVDPEQFSSQDSNIMLEQKAAVFPQQYAS
QAQMAQGSYPPMQDPNFHTMGQRPSYATLRMQPRPGLRPTGLVQNQPNQLRLQLQHRLQA
QQNRQPLMNQISNVSNVNLTLRPGVPTQAPINAQMLAQRQREILNQHLRQRQMHQQQQVQ
QRTLMMRGQGLNMTSSMVAPGGLPATLSNPRMPQANAQQFPFPPNYGISQQPDPGFTGAT
TPQSPLMSPRMAHTQSPMMQQSQANPAYQASSDMNGWAQGNMGGNSMFSQQSPPHFGQQA
NTSMYNNNMNINVSMATNTGGMNNMNQMTGQISMTSVTSVPTSGLSSMGPEQKYC
>175Sheep|A0A6P3YKL4|Mammalia|SRC2
MSGMGENTPDPSRAETRKRKECPDQLGPSPKRSTEKRNREQENKYIEELAELIFANFNDI
DNFNFKPDKCAILKETVKQIRQIKEQEKAAAANIDEVQKSDVSSTGQGVIDKDALGPMML
EALDGFFFVVNLEGNVVFVSENVTQYLRYNQEELMNKSVYSVLHVGDHTEFVKNLLPKSI
VNGGAWSGEPPRRNSHTFNCRMLVKPLPESEEEGHDNQEAHQKYETMQCFAVSQPKSIKE
EGEDLQSCLICVARRVPMKERPALPSSESFTTRQDLQGKITSLDTSTMRAAMKPGWEDLV
RRCIQKFHTQHEGESVSYAKRHHHEVLRQGLAFSQIYRFSLSDGTLVAAQTKSKLIRSQT
TNEPQLVISLHVLHREQNVCVMNPDLTGQAMGKPLNPISSSSPAHPAMCSGNPGQDMTLS

SNINFPMNGPKEQMGMPMGRFSGSGGMNHVSSMQATTPQGSNYALKMNSPSQSSPGLNPG
QPSSMLSPRHRMSPGVAGSPRIPPSQFSPAGSLHSPVGVCSSTGNSHSYTNSSLNALQAL
SEGHGVSLGSSLASPDLKMGNLQNSPVNMNPPQLSKMGSLDSKDCFGLYGEPPEGPTGPA
ESSCHPGEPKDPSEAGLPAAGSNERPDGQSRLLDNKGQTKLLQLLTTKSDQIEPSPLPSS
LSDTNKDSTGSLPGPGSTHGTSLKEKHKILHRLLQDSSSPVDLAKLTAEATGKELSQEAS
STAPGSEATIKQEPVSPKKKENALLRYLLDKDDTKDIGLPEITPKLERLDSKTDPASNTK
LIAMKTEKEEMSFEPSDQPGSELDNLEEILDDLQNSQLPQLFPDTRPGAPAGSVDKQAII
NDLMQLTAESSPVTLVGAQKTAMRIPQSTFNNPRPGQLGRLLPNQNFPLDITLQSPTGAG
PFPPIRNSSPYSVIPQPGMMGNQGMIGNQGNLGNSSTGMLGSNAARPTMPSGEWAPQSPA
VRVTCAATSNAMSRPIQGGMIRNPTASIPMRPNSQPGQRQMLQPQVMNMGPSELEMNMGG
PQYSQQQAPPNQTAPWPESILPIDQAPFASQNRQPFGSSPDDLLCPHPAAESPSDEGALL
DQLYLALRNFDGLEEIDRALGIPELVSQSQGVDPEQFSSQDSSLMLEQKTPVFPQQYASQ
AQVAQGSYTPMQDPSFHTMGQRPSYATLRMQPRPGLRPTGLVQSQPNQLRLQLQHRLQAQ
QNRQPLMNQISNVSNVNLTLRPGVPTQAPINAQMLAQRQREILNQHLRQRQMHHQQQQVQ
QRTLMMRGQGLNMTPSVVAPSGVPATMSNPRLPQANAQQFPFPPNYGISQQPDPGFTGAA
APQSPLISPRLAHTQSPMLQQSQANPAYQASSDMNGWAQGSMGANSMFSQQSPPHFAQQA
NTSMYNNNMNINVSMATNTGGMSNMNQMTGQISMTSVTSVPTSGLSSMGPEQKYC
>176Ovis aries|A0A6P3YN88|Mammalia|SRC2
MSGMGENTPDPSRAETRKRKECPDQLGPSPKRSTEKRNREQENKYIEELAELIFANFNDI
DNFNFKPDKCAILKETVKQIRQIKEQEKAAAANIDEVQKSDVSSTGQGVIDKDALGPMML
EALDGFFFVVNLEGNVVFVSENVTQYLRYNQEELMNKSVYSVLHVGDHTEFVKNLLPKSI
VNGGAWSGEPPRRNSHTFNCRMLVKPLPESEEEGHDNQEAHQKYETMQCFAVSQPKSIKE
EGEDLQSCLICVARRVPMKERPALPSSESFTTRQDLQGKITSLDTSTMRAAMKPGWEDLV
RRCIQKFHTQHEGESVSYAKRHHHEVLRQGLAFSQIYRFSLSDGTLVAAQTKSKLIRSQT
TNEPQLVISLHVLHREQNVCVMNPDLTGQAMGKPLNPISSSSPAHPAMCSGNPGQDMTLS
SNINFPMNGPKEQMGMPMGRFSGSGGMNHVSSMQATTPQGSNYALKMNSPSQSSPGLNPG
QPSSMLSPRHRMSPGVAGSPRIPPSQFSPAGSLHSPVGVCSSTGNSHSYTNSSLNALQAL
SEGHGVSLGSSLASPDLKMGNLQNSPVNMNPPQLSKMGSLDSKDCFGLYGEPPEGPTGPA
ESSCHPGEPKDPSEAGLPAAGSNERPDGQSRLLDNKGQTKLLQLLTTKSDQIEPSPLPSS
LSDTNKDSTGSLPGPGSTHGTSLKEKHKILHRLLQDSSSPVDLAKLTAEATGKELSQEAS
STAPGSEATIKQEPVSPKKKENALLRYLLDKDDTKDIGLPEITPKLERLDSKTDPASNTK
LIAMKTEKEEMSFEPSDQPGSELDNLEEILDDLQNSQLPQLFPDTRPGAPAGSVDKQAII
NDLMQLTAESSPVTLVGAQKTAMRIPQSTFNNPRPGQLGRLLPNQNFPLDITLQSPTGAG
PFPPIRNSSPYSVIPQPGMMGNQGMIGNQGNLGNSSTGMLGSNAARPTMPSGEWAPQSPA
VRVTCAATSNAMSRPIQGGMIRNPTASIPMRPNSQPGQRQMLQPQVMNMGPSELEMNMGG
PQYSQQQAPPNQTAPWPESILPIDQAPFASQNRQPFGSSPDDLLCPHPAAESPSDEGALL
DQLYLALRNFDGLEEIDRALGIPELVSQSQGVDPEQFSSQDSSLMLEQKTPVFPQQYASQ
AQVAQGSYTPMQDPSFHTMGQRPSYATLRMQPRPGLRPTGLVQSQPNQLRLQLQHRLQAQ
QNRQPLMNQISNVSNVNLTLRPGVPTQAPINAQMLAQRQREILNQHLRQRQMHHQQQQVQ
QRTLMMRGQGLNMTPSVVAPSGVPATMSNPRLPQANAQQFPFPPNYGISQQPDPGFTGAA
APQSPLISPRLAHTQSPMLQQSQANPAYQASSDMNGWAQGSMGANSMFSQQSPPHFAQQA
NTSMYNNNMNINVSMATNTGGMSNMNQMTGQISMTSVTSVPTSGLSSMGPEQVNDPALRG
GGLFPSQLPGMDMIKQEGDASRKYC
>177Panthera pardus|A0A6P4WN98|Mammalia|SRC2
MSGMGENTSDPSRAETRKRKECPDQLGPSPKRSTEKRNREQENKYIEELAELIFANFNDI
DNFNFKPDKCAILKETVKQIRQIKEQEKAAAANIDEVQKSDVSSTGQGVIDKDALGPMML
EALDGFFFVVNLEGNVVFVSENVTQYLRYNQEELMNKSVYSILHVGDHTEFVKNLLPKSI
VNGGSWSGEPPRRNSHTFNCRMLVKPLPDSEEEGHDNQEAHQKYETMQCFAVSQPKSIKE
EGEDLQSCLICVARRVPMKERPGLPSSESFTTRQDLQGKITSLDTSTMRAAMKPGWEDLV
RRCIQKFHAQHEGESVSYAKRHHHEVLRQGLAFSQIYRFSLSDGTLVAAQTKSKLIRSQT
TNEPQLVISLHMLHREQNVCVMNPDLTGQAMGKPLNPISSSSPAHQAMCSGNPGQDMTLS
SNINFPINGPKEQMGMPMGRFGGSGGMNHVSSMQATTPQGSNYALKMNSPSQSSPGMNPG
QPNSMLSPRHRMSPGVAGSPRIPPSQFSPAGSLHSPVGVCSSTGNSHSYTNSSLNALQAL
SEGHGVSLGSSLASPDLKMGNLQNSPVNMNPPPLSKMGSLDSKDCFGLYGEPSEGTTGQA

ESSCHPGEQKEANDPSMPQAVSGERSDGQSRLHDGKGQTKLLQLLTTKSDQMEPSPLSSS
LSDTNKDSAGSLPGSGSTHGTSLKEKHKILHRLLQDSSSPVDLAKLTAEATGKELSQEAG
NTAPGSEVTIKQEPVSPKKKENALLRYLLDKDDTKDIGLPEITPKLERLDSKTDPASNTK
LIAMKTEKEEMSFEPSDQPGSELDNLEEILDDLQNSQLPQLFPDTRPGAPAGSVDKQAII
NDLMQLTAENSPVTPVGAQKTALRISQSRMIGSNASRPTMPSGEWAPQSSAVRVTCAATT
GALNRPIQGGMIRNPTASIPMRPNSQPGQRQMLQSQVMNIGPSELEMNMGGPQYSQQQAP
PNQTAPWPESILPIDQASFASQNRQPFGSSPDDLLCPHPAAESPSDEGALLDQLYLALRN
FDGLEEIDRALGIPELVSQSQAVDPEQFSSQESNIMLEQKTPVFPQQYASQAQMAQGSYN
PMQDPNFHTMGQRPSYATLRMQPRPGLRPTGLVQNQPNQLRLQLQHRLQAQQNRQPLMNQ
ISNVSNVNLTLRPGVPTQAPINAQMLAQRQREILNQHLRQRQMHQQQQVQQRTLMMRGQG
LNMTSSMVAPGGLPATLSNPRIPQANAQQFPFPPNYGISQQPDPGFTGATTPQSPLMSPR
MAHTQSPMMQQSQANPAYQASSDMNGWAQGNMGGNSMFSQQSPPHFGQQANTSMYNNNMN
INVSMATNTGGMSNMNQMTGQISMTSVTSVPTSGLSSMGPEQVNDPALRGGNLFPNQLPG
MDMIKQEGDASRKYC
>178Leopard|A0A6P4WWM1|Mammalia|SRC2
MSGMGENTSDPSRAETRKRKECPDQLGPSPKRSTEKRNREQENKYIEELAELIFANFNDI
DNFNFKPDKCAILKETVKQIRQIKEQEKAAAANIDEVQKSDVSSTGQGVIDKDALGPMML
EALDGFFFVVNLEGNVVFVSENVTQYLRYNQEELMNKSVYSILHVGDHTEFVKNLLPKSI
VNGGSWSGEPPRRNSHTFNCRMLVKPLPDSEEEGHDNQEAHQKYETMQCFAVSQPKSIKE
EGEDLQSCLICVARRVPMKERPGLPSSESFTTRQDLQGKITSLDTSTMRAAMKPGWEDLV
RRCIQKFHAQHEGESVSYAKRHHHEVLRQGLAFSQIYRFSLSDGTLVAAQTKSKLIRSQT
TNEPQLVISLHMLHREQNVCVMNPDLTGQAMGKPLNPISSSSPAHQAMCSGNPGQDMTLS
SNINFPINGPKEQMGMPMGRFGGSGGMNHVSSMQATTPQGSNYALKMNSPSQSSPGMNPG
QPNSMLSPRHRMSPGVAGSPRIPPSQFSPAGSLHSPVGVCSSTGNSHSYTNSSLNALQAL
SEGHGVSLGSSLASPDLKMGNLQNSPVNMNPPPLSKMGSLDSKDCFGLYGEPSEGTTGQA
ESSCHPGEQKEANDPSMPQAVSGERSDGQSRLHDGKGQTKLLQLLTTKSDQMEPSPLSSS
LSDTNKDSAGSLPGSGSTHGTSLKEKHKILHRLLQDSSSPVDLAKLTAEATGKELSQEAG
NTAPGSEVTIKQEPVSPKKKENALLRYLLDKDDTKDIGLPEITPKLERLDSKTDPASNTK
LIAMKTEKEEMSFEPSDQPGSELDNLEEILDDLQNSQLPQLFPDTRPGAPAGSVDKQAII
NDLMQLTAENSPVTPVGAQKTALRISQSTFNNPRPGQLGRLLPNQNLPLDITLQSPTGAG
PFPPIRNNSPYSVIPQPGMMGNQGMIGNQGNLGNSSTGMIGSNASRPTMPSGEWAPQSSA
VRVTCAATTGALNRPIQGGMIRNPTASIPMRPNSQPGQRQMLQSQVMNIGPSELEMNMGG
PQYSQQQAPPNQTAPWPESILPIDQASFASQNRQPFGSSPDDLLCPHPAAESPSDEGALL
DQLYLALRNFDGLEEIDRALGIPELVSQSQAVDPEQFSSQESNIMLEQKTPVFPQQYASQ
AQMAQGSYNPMQDPNFHTMGQRPSYATLRMQPRPGLRPTGLVQNQPNQLRLQLQHRLQAQ
QNRQPLMNQISNVSNVNLTLRPGVPTQAPINAQMLAQRQREILNQHLRQRQMHQQQQVQQ
RTLMMRGQGLNMTSSMVAPGGLPATLSNPRIPQANAQQFPFPPNYGISQQPDPGFTGATT
PQSPLMSPRMAHTQSPMMQQSQANPAYQASSDMNGWAQGNMGGNSMFSQQSPPHFGQQAN
TSMYNNNMNINVSMATNTGGMSNMNQMTGQISMTSVTSVPTSGLSSMGPEQVNDPALRGG
NLFPNQLPGMDMIKQEGDASRKYC
>179Panthera pardus|A0A6P4WWN0|Mammalia|SRC2
MSGMGENTSDPSRAETRKRKECPDQLGPSPKRSTEKRNREQENKYIEELAELIFANFNDI
DNFNFKPDKCAILKETVKQIRQIKEQEKAAAANIDEVQKSDVSSTGQGVIDKDALGPMML
EALDGFFFVVNLEGNVVFVSENVTQYLRYNQEELMNKSVYSILHVGDHTEFVKNLLPKSI
VNGGSWSGEPPRRNSHTFNCRMLVKPLPDSEEEGHDNQEAHQKYETMQCFAVSQPKSIKE
EGEDLQSCLICVARRVPMKERPGLPSSESFTTRQDLQGKITSLDTSTMRAAMKPGWEDLV
RRCIQKFHAQHEGESVSYAKRHHHEVLRQGLAFSQIYRFSLSDGTLVAAQTKSKLIRSQT
TNEPQLVISLHMLHREQNVCVMNPDLTGQAMGKPLNPISSSSPAHQAMCSGNPGQDMTLS
SNINFPINGPKEQMGMPMGRFGGSGGMNHVSSMQATTPQGSNYALKMNSPSQSSPGMNPG
QPNSMLSPRHRMSPGVAGSPRIPPSQFSPAGSLHSPVGVCSSTGNSHSYTNSSLNALQAL
SEGHGVSLGSSLASPDLKMGNLQNSPVNMNPPPLSKMGSLDSKDCFGLYGEPSEGTTGQA
ESSCHPGEQKEANDPSMPQAVSGERSDGQSRLHDGKGQTKLLQLLTTKSDQMEPSPLSSS
LSDTNKDSAGSLPGSGSTHGTSLKEKHKILHRLLQDSSSPVDLAKLTAEATGKELSQEAG
NTAPGSEVTIKQEPVSPKKKENALLRYLLDKDDTKDIGLPEITPKLERLDSKTDPASNTK

LIAMKTEKEEMSFEPSDQPGSELDNLEEILDDLQNSQLPQLFPDTRPGAPAGSVDKQAII
NDLMQLTAENSPVTPVGAQKTALRISQSTFNNPRPGQLGRLLPNQNLPLDITLQSPTGAG
PFPPIRNNSPYSVIPQPGMMGNQGMIGNQGNLGNSSTGMIGSNASRPTMPSGEWAPQSSA
VRVTCAATTGALNRPIQGGMIRNPTASIPMRPNSQPGQRQMLQSQVMNIGPSELEMNMGG
PQYSQQQAPPNQTAPWPESILPIDQASFASQNRQPFGSSPDDLLCPHPAAESPSDEGALL
DQLYLALRNFDGLEEIDRALGIPELVSQSQAVDPEQFSSQESNIMLEQKTPVFPQQYASQ
AQMAQGSYNPMQDPNFHTMGQRPSYATLRMQPRPGLRPTGLVQNQPNQLRLQLQHRLQAQ
QNRQPLMNQISNVSNVNLTLRPGVPTQAPINAQMLAQRQREILNQHLRQRQMHQQQQVQQ
RTLMMRGQGLNMTSSMVAPGGLPATLSNPRIPQANAQQFPFPPNYGISQQPDPGFTGATT
PQSPLMSPRMAHTQSPMMQQSQANPAYQASSDMNGWAQGNMGGNSMFSQQSPPHFGQQAN
TSMYNNNMNINVSMATNTGGMSNMNQMTGQISMTSVTSVPTSGLSSMGPEQKYC
>180Puma concolor|A0A6P6HRX6|Mammalia|SRC2
MSGMGENTSDPSRAETRKRKECPDQLGPSPKRSTEKRNREQENKYIEELAELIFANFNDIDNFNFKPDKCAILK
ETVKQIRQIKEQEKAAAANIDEVQKSDVSSTGQGVIDKDALGPMMLEALDGFFFVVNLEGNVVFVSENVTQYLR
YNQEELMNKSVYSILHVGDHTEFVKNLLPKSIVNGGSWSGEPPRRNSHTFNCRMLVKPLPDSEEEGHDNQEAHQ
KYETMQCFAVSQPKSIKEEGEDLQSCLICVARRVPMKERPGLPSSESFTTRQDLQGKITSLDTSTMRAAMKPGW
EDLVRRCIQKFHAQHEGESVSYAKRHHHEVLRQGLAFSQIYRFSLSDGTLVAAQTKSKLIRSQTTNEPQLVISL
HMLHREQNVCVMNPDLTGQAMGKPLNPISSSSPAHQAMCSGNPGQDMTLSSNINFPINGPKEQMGMPMGRFGGS
GGMNHVSSMQATTPQGSNYALKMNSPSQSSPGMNPGQPNSMLSPRHRMSPGVAGSPRIPPSQFSPAGSLHSPVG
VCSSTGNSHSYTNSSLNALQALSEGHGVSLGSSLASPDLKMGNLQNSPVNMNPPPLSKMGSLDSKDCFGLYGEP
SEGTTGQAESSCHPGEQKEANDPSMPQAVSGERSDGQSRLHDGKGQTKLLQLLTTKSDQMEPSPLSSSLSDTNK
DSTGSLPGSGSTHGTSLKEKHKILHRLLQDSSSPVDLAKLTAEATGKELSQEAGNTAPGSEVTIKQEPVSPKKK
ENALLRYLLDKDDTKDIGLPEITPKLERLDSKTDPASNTKLIAMKTEKEEMSFEPSDQPGSELDNLEEILDDLQ
NSQLPQLFPDTRPGAPAGSVDKQAIINDLMQLTAENSPVTPVGAQKTALRISQSTFNNPRPGQLGRLLPNQNLP
LDITLQSPTGAGPFPPIRNNSPYSVIPQPGMMGNQGMIGNQGNLGNSSTGMIGSNASRPTMPSGEWAPQSSAVR
VTCAATTGALNRPIQGGMIRNPTASIPMRPNSQPGQRQMLQSQVMNIGPSELEMNMGGPQYSQQQAPPNQTAPW
PESILPIDQASFASQNRQPFGSSPDDLLCPHPAAESPSDEGALLDQLYLALRNFDGLEEIDRALGIPELVSQSQ
AVDPEQFSSQESNIMLEQKTPVFPQQYASQAQMAQGSYNPMQDPNFHTMGQRPSYATLRMQPRPGLRPTGLVQN
QPNQLRLQLQHRLQAQQNRQPLMNQISNVSNVNLTLRPGVPTQAPINAQMLAQRQREILNQHLRQRQMHQQQQV
QQRTLMMRGQGLNMTSSMVAPGGLPATLSNPRIPQANAQQFPFPPNYGISQQPDPGFTGATTPQSPLMSPRMAH
TQSPMMQQSQANPAYQASSDMNGWAQGNMGGNSMFSQQSPPHFGQQANTSMYNNNMNINVSMATNTGGMSNMNQ
MTGQISMTSVTSVPTSGLSSMGPEQVNDPALRGGNLFPNQLPGMDMIKQEGDASRKYC
>181Ovis aries|A0A6P7EF47|Mammalia|SRC2
MSGMGENTPDPSRAETRKRKECPDQLGPSPKRSTEKRNREQENKYIEELAELIFANFNDI
DNFNFKPDKCAILKETVKQIRQIKEQEKAAAANIDEVQKSDVSSTGQGVIDKDALGPMML
EALDGFFFVVNLEGNVVFVSENVTQYLRYNQEELMNKSVYSVLHVGDHTEFVKNLLPKSI
VNGGAWSGEPPRRNSHTFNCRMLVKPLPESEEEGHDNQEAHQKYETMQCFAVSQPKSIKE
EGEDLQSCLICVARRVPMKERPALPSSESFTTRQDLQGKITSLDTSTMRAAMKPGWEDLV
RRCIQKFHTQHEGESVSYAKRHHHEVLRQGLAFSQIYRFSLSDGTLVAAQTKSKLIRSQT
TNEPQLVISLHVLHREQNVCVMNPDLTGQAMGKPLNPISSSSPAHPAMCSGNPGQDMTLS
SNINFPMNGPKEQMGMPMGRFSGSGGMNHVSSMQATTPQGSNYALKMNSPSQSSPGLNPG
QPSSMLSPRHRMSPGVAGSPRIPPSQFSPAGSLHSPVGVCSSTGNSHSYTNSSLNALQAL
SEGHGVSLGSSLASPDLKMGNLQNSPVNMNPPQLSKMGSLDSKDCFGLYGEPPEGPTGPA
ESSCHPGEPKDPSEAGLPAAGSNERPDGQSRLLDNKGQTKLLQLLTTKSDQIEPSPLPSS
LSDTNKDSTGSLPGPGSTHGTSLKEKHKILHRLLQDSSSPVDLAKLTAEATGKELSQEAS
STAPGSEATIKQEPVSPKKKENALLRYLLDKDDTKDIGLPEITPKLERLDSKTDPASNTK
LIAMKTEKEEMSFEPSDQPGSELDNLEEILDDLQNSQLPQLFPDTRPGAPAGSVDKQAII
NDLMQLTAESSPVTLVGAQKTAMRIPQSTFNNPRPGQLGRLLPNQNFPLDITLQSPTGAG
PFPPIRNSSPYSVIPQPGMMGNQGMIGNQGNLGNSSTGMLGSNAARPTMPSGEWAPQSPA
VRVTCAATSNAMSRPIQGGMIRNPTASIPMRPNSQPGQRQMLQPQVMNMGPSELEMNMGG
PQYSQQQAPPNQTAPWPESILPIDQAPFASQNRQPFGSSPDDLLCPHPAAESPSDEGALL
DQLYLALRNFDGLEEIDRALGIPELVSQSQGVDPEQFSSQDSSLMLEQKTPVFPQQYASQ
AQVAQGSYTPMQDPSFHTMGQRPSYATLRMQPRPGLRPTGLVQSQPNQLRLQLQHRLQAQ
QNRQPLMNQISNVSNVNLTLRPGVPTQAPINAQMLAQRQREILNQHLRQRQMHHQQQQVQ

QRTLMMRGQGLNMTPSVVAPSGVPATMSNPRLPQANAQQFPFPPNYGISQQPDPGFTGAA
APQSPLISPRLAHTQSPMLQQSQANPAYQASSDMNGWAQGSMGANSMFSQQSPPHFAQQA
NTSMYNNNMNINVSMATNTGGMSNMNQMTGQISMTSVTSVPTSGLSSMGPEQVNDPALRG
GGLFPSQLPGMDMIKQEGDASRVRNNREINPWL
>182Rhegmatorhina hoffmannsi|A0A7K7H7X5|Aves|SRC2
MSGMGENTSDPSRAESRKRKDPDQLGPSPKRSTEKRNREQENKYIEELAELIFANFNDID
NFNFKPDKCAILKETVKQIRQIKEQEKAAAANEDEVQKADVSSTGQSVIDKDALGPMMLE
ALDGFFFVVNPEGNVVFVSENVTQYLRYNQEELMNTSIYSILHVGDHNEFVKILLPKSLV
NGGSWSGDTPRRNSHTFNCRMLVKPLADSEEGHDNQEAHQKYETMQCFAVSQPNSFKEDG
EDLQSCLICVARRAPMKERPLLPSSESFTTRQDLQGKITSLDTSTMRAAMKPGWEDLVRR
CIQRFHSQHEGEISFAKRHHQEVLRQGLAYSPIYRFSLSDGTIASAQTKSKLIRSQTTNE
PQLVISLHVLHREQNVCGMNQDLTGQGMGKILNPISSSSPAHQAMCSGNPGQDMTISSNI
NFAINGPKEQMGMPAGRFGGSGGMNHVSSIQTSTPQGSNYALKMNSPSQSSPGMNPGQPN
SMLSPRHRVSPGVAGSPRIPPSQFSPAGSLHSPVGVCSSTGNSHNYTNSSLNALQALSEG
HGVSLGSSLASPDLKMGNLQNSPVNMNPPQLSKMGSLDSKDCFGMYGEQSEGTTGQAENN
CHSGEQKDNSDSNMPPVVSGERPDGQSKLHDGKSQTKLLQLLTTKSDQMEPSPLSSTMGD
ISKDSTGGGLPGSGSTHGTSLKEKHKILHRLLQDSSSPVDLAKLTAEATGKELNQESSST
APGSEVTVKQEPASPKKNNNALLRYLLDKDDTKDIGLPDIPPKLERLDSKTDPSGSTKLI
TMRTEKEEISFEPNEQPGSELDNLEEILDDLQNSQLPQLFPDSRPGAPAGSVDKQAIIND
LMQLTSDSSPGTAVATQKPAMRISQSTFNNPRAGQLGRLLPNQNLPLDITLQNPTGAGTF
PPIRTSSPYSVIPQPGLMGRNEGMIGSQGALGNSSTGMIGGNAPRPAMTSGDWGSQAAAV
RVTCAPTASAMNRPVQAGMIRNPTASIPLRPSNQPGPRQMLSSQVMNMGPSELEMNIGGP
QYSQQQAPPNQTAPWPDSILPIEQATFGNQNRQPFGSSPDDLLCNHPSSESPSDEGALLD
QLYMALRNFDGLEEIDRALGIPELVSQSQAVDPETFPSQESSILMEQKAPVFSQQYAAQA
QMAQGSFAPMQDPSFHTMGQRPGYTALRMQPRPGLRPTGMVQNQPNQLRLQLQHRLQAQQ
NRQPLINQISSVSNMNLSLRPGVPTQGPINAQMLAQRQREILSQHLRQRQMQQQQQVQQ
RTLMMRGQGLSMTPSMVATGGIPATMSNPRIPQANAQQFPFPPNYGISQQPDPGFTGATT
PQSPLMSPRLAHTQSPMIQQSQANPAYQSSTEMNGWAQGNMGGNSMFSQQSPPHFGQQAS
TSMYNNNMNISVSMATNTSGMNNMNQMTGQISMTSATSVPTSGLSSMGPEQVNDPAMRGG
NLFPNQLPGMDMIKQDADAAR
>183Certhia brachydactyla|A0A7L1W1X7|Aves|SRC2
MSGMGENTSDPSRAESRKRKDPDQVGPSPKRSTEKRNREQENKYIEELAELIFANFNDIDNFNFKPDKCAILKE
TVKQIRQIKEQEKAAAANEDEVQKADVSSTGQSVIDKDALGPMMLEALDGFFFVVNPEGNVVFVSENVTQYLRY
NQEELMNTSIYSILHVGDHNEFVKILLPKSLVNGGSWSGDTPRRNSHTFNCRMLVKPLADSEEGHDNQEAHQKY
ETMQCFAVSQPNSFKEDGEDLQSCLICVARRAPMKERPLLPSSESFTTRQDLQGKITSLDTSTMRAAMKPGWED
LVRRCIQRFHSQHEGEISFAKRHHQEVLRQGLAFSPVYRFSLSDGTIVSAQTKSKLIRSQTTSEPQLVISLHML
HREQNVCGMNQDLTGQGMGKILNPISSSSPAHQAMCSGNPGQDMTISSNINFAINGPKEQVGMPAGRFGGSGGM
NHVSSIQASTPQGSNYALKMNSPSQSSPGMNPGQPNSMLSPRHRVSPGVAGSPRIPPSQFSPAGSLHSPVGVCS
STGNSHNYTNSSLNALQALSEGHGVSLGSSLASPDLKMGNLQNSPVNMNPPQLSKMGSLDSKDCFGIYGEQSEG
TTGQAESSCHSGEQKDNSESSMPPVVSGERPDGQNKLHDGKSQTKLLQLLTTKSDQMEPSPLSSTMGDISKDST
GGLPGSGSAHGTSLKEKHKILHRLLQDSSSPVDLAKLTAEATGKELNQESSSTAPGSEVTVKQEPASPKKNNNA
LLRYLLDKDDTKDIGLPDIPPKLERLDSKTDPSGSTKLIAMRTEKEEISFEPNEQPGSELDNLEEILDDLQNSQ
LPQLFPDSRPGVPAGSVDKQAIINDLMQLTSDSSPGAAVATQKPAMRISQSTFNNPRAGQLGRLLPNQNLPLDI
TLQNPTGAGTFPPIRTSSPYSVIPQPGLMGRNEGMIGSQGTLGNSSTGMIGGNAPRPAMASGDWGSQAPAVRVT
CAPTAGTMNRPVQAGMIRNPTASIPLRPSNQPGPRQMLPSQVMNMGPSELEMNIGGPQYSQQQAPPNQTAPWPD
SILPIEQATFGNQNRQPFGSSPDDLLCNHPSSESPSDEGALLDQLYMALRNFDGLEEIDRALGIPELVSQSQAV
DPETFPSQESSMLLEQKAPVFSQQYAAQAQMAQGSYAPMQDPSFHAMGQRPGYTALRMQPRPGLRPAGIVQNQP
NQLRLQLQHRLQAQQNRQPLINQISSVSNMNLSLRTGVPTQGPINAQMLAQRQREILSQHLRQRQMQQQQQVQ
QRTLMMRGQGLSMTPSMVATGGIPATMSNPRIPQANAQQFPFPPNYGISQQPDPGFTGATTPQSPLMSPRLAHT
QSPMIQQSQANPAYQSSTEMNGWAQGNMGGNSMFSQQSPPHFGQQASTSMYNNNMNISVSMATNTSGMNNMNQM
TGQISMTSATSVPTSGLSSMGPEQVNDPAMRGGNLFPNQLPGMDMIKQDGDAAR
>184Rissa tridactyla|A0A7L3T2J2|Aves|SRC2
MSGMGENTSDPSRAESRKRKDPDQLGPSPKRSTEKRNREQENKYIEELAELIFANFNDIDNFNFKPDKCAILKE
TVKQIRQIKEQEKAAAANEDEVQKADVSSTGQSVIDKDALGPMMLEALDGFFFVVNPEGNVVFVSENVTQYLRY

NQEELMNTSIYSILHVGDHNEFVKILLPKSLVNGGSWSGDTPRRNSHTFNCRMLVKPLADSEEGHDNQEAHQKY
ETMQCFAVSQPNSFKEDGEDLQSCLICVARRAPMKERPLLPSSESFTTRQDLQGKITSLDTSTMRAAMKPGWED
LVRRCIQRFHSQHEGEISFAKRHHQEVLRQGLAFSPVYRFSLSDGTIVSAQTKSKLIRSQTTSEPQLVISLHML
HREQNVCGMNQDLTGQGMGKILNPISSSSPAHQAMCSGNPGQDMTISSNINFAINGPKEQMGMPAGRFGGSGGM
NHVSSIQASTPQGSNYALKMNSPSQSSPGMNPGQPNSMLSPRHRVSPGVAGSPRIPPSQFSPAGSLHSPVGVCS
STGNSHNYTNSSLNALQALSEGHGVSLGSSLASPDLKMGNLQNSPVNMNPPQLSKMGSLDSKDCFGLYGEQSEG
TTGQAESSCHSGEQKDNSDSNMPPVVSGERPDGQNKLHDGKSQTKLLQLLTTKSDQMEPSPLSSTMGDVSKDST
GGLPGSGSAHGTSLKEKHKILHRLLQDSSSPVDLAKLTAEATGKELNQESSSTAPGSEVTVKQEPASPKKNNNA
LLRYLLDKDDTKDIGLPDIPPKLERLDSKTDPSGSTKLIAMRTEKEEINFEPNEQPGSELDNLEEILDDLQNSQ
LPQLFPESRPGAPAGSVDKQAIINDLMQLTSDSSPGAAVATQKPAMRISQSTFNNPRAGQLGRLLPNQNLPLDI
TLQNPTGAGTFPPIRTSSPYSVIPQPGLMGRNEGMIGNQGTLGNSSTGMIGGNAPRPAMTSGDWGSQAPAVRVT
CAPTASAMNRPVQAGMIRNPTASIPLRPSNQPGPRQMLPSQVMNMGPSELEMNIGGPQYSQQQAPPNQTAPWPD
SILPIDQATFGNQNRQPFGSSPDDLLCNHPSSESPSDEGALLDQLYMALRNFDGLEEIDRALGIPELVSQSQAV
DPETFPSQESGILMEQKAPVFSQQYAAQAQMAQGSYAPMQDPSFHPMGQRPGYAALRMQPRPGLRPAGIVQNQP
NQLRLQLQHRLQAQQNRQPLINQISSVSNMNLSLRPGVPTQGPINAQMLAQRQREILSQHLRQRQMQQQQQVQQ
RTLMMRGQGLSMTPSMVATGGIPATMSNPRIPQANAQQFPFPPNYGISQQPDPGFTGATTPQSPLMSPRLAHTQ
SPMIQQSQANPAYQSSTEMNGWAQGNMGGNSMFSQQSPPHFGQQASTSMYNNNMNISVSMATNTSGMNNNMNQMT
GQISMTSATSVPTSGLSSMGPEQVNDPAMRGGNLFPNQLPGMDMIKQDGDATR
>185Canis lupus familiaris|E2QY25|Mammalia|SRC2
MSGMGENTSDPSRAETRKRKECPDQLGPSPKRSTEKRNREQENKYIEELAELIFANFNDI
DNFNFKPDKCAILKETVKQIRQIKEQEKAAAANIDEVQKSDVSSTGQGVIDKDALGPMML
EALDGFFFVVNLEGNVVFVSENVTQYLRYNQEELMNKSVYGILHVGDHTEFVKNLLPKSI
VNGGSWSGEPPRRNSHTFNCRMLVKPLPDSEEEGHDNQEAHQKYETMQCFAVSQPKSIKE
EGEDLQSCLICVARRVPMKERPVLPSSESFTTRQDLQGKITSLDTSTMRAAMKPGWEDLV
RRCIQKFHAQHEGESVSYAKRHHHEVLRQGLAFSQIYRFSLSDGTLVAAQTKSKLIRSQT
TNEPQLVISLHMLHREQNVCVMNPDLTGQAMGKPLNPISSSSPAHQAMCSGNPGQDMTLS
SNINFPINGPKEQMGMPMGRFGGSGGMNHVSSMQATTPQGSNYALKMNSPSQSSPGMNPG
QPNSMLSPRHRMSPGVAGSPRIPPSQFSPAGSLHSPVGVCSSTGNSHSYTNSSLNALQAL
SEGHGVSLGSSLASPDLKMGNLQNSPVNMNPPPLSKMGSLDSKDCFGLYGEPSEGTTGPA
ESSCHPGEQKETNDSNMPPAVSSERADGQNRLHDSKGQTKLLQLLTTKSDQMEPSPLSSS
LSDANKDSTAGLPGSGSTHGTSLKEKHKILHRLLQDSSSPVDLAKLTAEATGKELSQEAS
STAPGSEVTIKQEPVSPKKKENALLRYLLDKDDTKDIGLPEITPKLERLDSKTDPASNTK
LIAMKTEKEEMSFEPSDQPGSELDNLEEILDDLQNSQLPQLFPDTRPGAPAGSVDKQAII
NDLMQLTAENSPVTPVGAQKTALRISQSTFNNPRPGQLGRLLPNQNLPLDITLQSPTGAG
PFPPIRNNSPYSVIPQPGMMGNQGMIGNQGNLGNSSTGMIGSNASRPTMPSGEWAPQSSA
VRVTCAAATTSAMNRPIQGGMIRNPTASIPMRPNSQPGQRQMLQSQVMNIGPSELEMNMG
GPQYSQQQAPPNQTAPWPESILPIDQASFANQNRQPFGSSPDDLLCPHPAAESPSDEGAL
LDQLYLALRNFDGLEEIDRALGIPELVSQSQAVDPEQFSSQDSNIMLEQKAAVFPQQYAS
QAQMAQGSYPPMQDPNFHTMGQRPSYATLRMQPRPGLRPTGLVQNQPNQLRLQLQHRLQA
QQNRQPLMNQISNVSNVNLTLRPGVPTQAPINAQMLAQRQREILNQHLRQRQMHQQQQVQ
QRTLMMRGQGLNMTSSMVAPGGLPATLSNPRMPQANAQQFPFPPNYGISQQPDPGFTGAT
TPQSPLMSPRMAHTQSPMMQQSQANPAYQASSDMNGWAQGNMGGNSMFSQQSPPHFGQQA
NTSMYNNNMNINVSMATNTGGMNNMNQMTGQISMTSVTSVPTSGLSSMGPEQVNDPALRG
GNLFPNQLPGMDMIKQEGDASRILKGKEIFVRTVGQADSKHSATY
>186Felis catus|A0A2I2V024|Mammalia|SRC2
MSGMGENTSDPSRAETRKRKECPDQLGPSPKRSTEKRNREQENKYIEELAELIFANFNDIDNFNFKPDKCAILK
ETVKQIRQIKEQEKAAAANIDEVQKSDVSSTGQGVIDKDALGPMMLEALDGFFFVVNLEGNVVFVSENVTQYLR
YNQEELMNKSVYSILHVGDHTEFVKNLLPKSIVNGGSWSGEPPRRNSHTFNCRMLVKPLPDSEEEGHDNQEAHQ
KYETMQCFAVSQPKSIKEEGEDLQSCLICVARRVPMKERPGLPSSESFTTRQDLQGKITSLDTSTMRAAMKPGW
EDLVRRCIQKFHAQHEGESVSYAKRHHHEVLRQGLAFSQIYRFSLSDGTLVAAQTKSKLIRSQTTNEPQLVISL
HMLHREQNVCVMNPDLTGQAMGKPLNPISSSSPAHQAMCSGNPGQDMTLSSNINFPINGPKEQMGMPMGRFGGS
GGMNHVSSMQATTPQGSNYALKMNSPSQSSPGMNPGQPNSMLSPRHRMSPGVAGSPRIPPSQFSPAGSLHSPVG
VCSSTGNSHSYTNSSLNALQALSEGHGVSLGSSLASPDLKMGNLQNSPVNMNPPPLSKMGSLDSKDCFGLYGEP
SEGTTGQAESSCHPGEQKEANDPSMPQAVSGERSDGQSRLHDGKGQTKLLQLLTTKSDQMEPSPLSSSLSDTNK

DSTGSLPGSGSTHGTSLKEKHKILHRLLQDSSSPVDLAKLTAEATGKELSQEAGNTAPGSEVTIKQEPVSPKKK
ENALLRYLLDKDDTKDIGLPEITPKLERLDSKTDPASNTKLIAMKTEKEEMSFEPSDQPGSELDNLEEILDDLQ
NSQLPQLFPDTRPGAPAGSVDKQAIINDLMQLTAENSPVTPVGAQKTALRISQSTFNNPRPGQLGRLLPNQNLP
LDITLQSPTGAGPFPPIRNNSPYSVIPQPGMMGNQGMIGNQGNLGNSSTGMIGSNASRPTMPSGEWAPQSSAVR
VTCAATTGALNRPIQGGMIRNPTASIPMRPNSQPGQRQMLQSQVMNIGPSELEMNMGGPQYSQQQAPPNQTAPW
PESILPIDQASFASQNRQPFGSSPDDLLCPHPAAESPSDEGALLDQLYLALRNFDGLEEIDRALGIPELVSQSQ
AVDPEQFSSQESNIMLEQKTPVFPQQYASQAQMAQGSYNPMQDPNFHTMGQRPSYATLRMQPRPGLRPTGLVQN
QPNQLRLQLQHRLQAQQNRQPLMNQISNVSNVNLTLRPGVPTQAPINAQMLAQRQREILNQHLRQRQMHQQQQV
QQRTLMMRGQGLNMTSSMVAPGGLPATLSNPRIPQANAQQFPFPPNYGISQQPDPGFTGATTPQSPLMSPRMAH
TQSPMMQQSQANPAYQASSDMNGWAQGNMGGNSMFSQQSPPHFGQQANTSMYNNNMNINVSMATNTGGMSNMNQ
MTGQISMTSVTSVPTSGLSSMGPEQVNDPALRGGNLFPNQLPGMDMIKQEGDASRKYC

>187Pan troglodytes|A0A2I3RZ80|Mammalia|SRC2
MSGMGENTSDPSRAETRKRKECPDQLGPSPKRNTEKRNREQENKYIEELAELIFANFNDIDNFNFKPDKCAILK
ETVKQIRQIKEQEKAAAANIDEVQKSDVSSTGQGVIDKDALGPMMLEALDGFFFVVNLEGNVVFVSENVTQYLR
YNQEELMNKSVYSILHVGDHTEFVKNLLPKSIVNGGSWSGEPPRRNSHTFNCRMLVKPLPDSEEEGHDNQEAHQ
KYETMQCFAVSQPKSIKEEGEDLQSCLICVARRVPMKERPVLPSSESFTTRQDLQGKITSLDTSTMRAAMKPGW
EDLVRRCIQKFHAQHEGESVSYAKRHHHEVLRQGLAFSQIYRFSLSDGTLVAAQTKSKLIRSQTTNEPQLVISL
HMLHREQNVCVMNPDLTGQTMGKPLNPISSNSPAHQALCSGNPGQDMTLSSNINFPINGPKEQMGMPMGRFGGS
GGMNHVSGMQATTPQGSNYALKMNSPSQSSPGMNPGQPTSMLSPRHRMSPGVAGSPRIPPSQFSPAGSLHSPVG
VCSSTGNSHSYTNSSLNALQALSEGHGVSLGSSLASPDLKMGNLQNSPVNMNPPPLSKMGSLDSKDCFGLYGEP
SEGTTGQAESSCHPGEQKETNDPNLPPAVSSERADGQSRLHDSKGQTKLLQLLTTKSDQMEPSPLASSLSDTNK
DSTGSLPGSGSTHGTSLKEKHKILHRLLQDSSSPVDLAKLTAEATGKDLSQESSSTAPGSEVTIKQEPVSPKKK
ENALLRYLLDKDDTKDIGLPEITPKLERLDSKTDPASNTKLIAMKTEKEEMSFEPGDQPGSELDNLEEILDDLQ
NSQLPQLFPDTRPGAPAGSVDKQAIINDLMQLTAENSPVTPVGAQKTALRISQSTFNNPRPGQLGRLLPNQNLP
LDITLQSPTGAGPFPPIRNSSPYSVIPQPGMMGNQGMIGNQGNLGNSSTGMIGNSASRPTMPSGEWAPQSSAVR
VTCAATTSAMNRPVQGGMIRNPAASIPMRPSSQPGQRQTLQSQVMNIGPSELEMNMGGPQYSQQQAPPNQTAPW
PESILPIDQASFASQNRQPFGSSPDDLLCPHPAAESPSDEGALLDQLYLALRNFDGLEEIDRALGIPELVSQSQ
AVDPEQFSSQDSNIMLEQKAPVFPQQYASQAQMAQGSYSPMQDPNFHTMGQRPSYATLRMQPRPGLRPTGLVQN
QPNQLRLQLQHRLQAQQNRQPLMNQISNVSNVNLTLRPGVPTQAPINAQMLAQRQREILNQHLRQRQMHQQQQV
QQRTLMMRGQGLNMTPSMVAPSGMPATMSNPRIPQANAQQFPFPPNYGISQQPDPGFTGATTPQSPLMSPRMAH
TQSPMMQQSQANPAYQAPSDINGWAQGNMGGNSMFSQQSPPHFGQQANTSMYSNNMNINVSMATNTGGMSSMNQ
MTGQISMTSVTSVPTSGLSSMGPEQVNDPALRGGNLFPNQLPGMDMIKQEGDTTRKYC
>188Canis lupus familiaris|A0A5F4BNW2|Mammalia|SRC2
MSGMGENTSDPSRAETRKRKECPDQLGPSPKRSTEKRNREQENKYIEELAELIFANFNDI
DNFNFKPDKCAILKETVKQIRQIKEQEKAAAANIDEVQKSDVSSTGQGVIDKDALGPMML
EALDGFFFVVNLEGNVVFVSENVTQYLRYNQEELMNKSVYGILHVGDHTEFVKNLLPKSI
VNGGSWSGEPPRRNSHTFNCRMLVKPLPDSEEEGHDNQEAHQKYETMQCFAVSQPKSIKE
EGEDLQSCLICVARRVPMKERPVLPSSESFTTRQDLQGKITSLDTSTMRAAMKPGWEDLV
RRCIQKFHAQHEGESVSYAKRHHHEVLRQGLAFSQIYRFSLSDGTLVAAQTKSKLIRSQT
TNEPQLVISLHMLHREQNVCVMNPDLTGQAMGKPLNPISSSSPAHQAMCSGNPGQDMTLS
SNINFPINGPKEQMGMPMGRFGGSGGMNHVSSMQATTPQGSNYALKMNSPSQSSPGMNPG
QPNSMLSPRHRMSPGVAGSPRIPPSQFSPAGSLHSPVGVCSSTGNSHSYTNSSLNALQAL
SEGHGVSLGSSLASPDLKMGNLQNSPVNMNPPPLSKMGSLDSKDCFGLYGEPSEGTTGPA
ESSCHPGEQKETNDSNMPPAVSSERADGQNRLHDSKGQTKLLQLLTTKSDQMEPSPLSSS
LSDANKDSTAGLPGSGSTHGTSLKEKHKILHRLLQDSSSPVDLAKLTAEATGKELSQEAS
STAPGSEVTIKQEPVSPKKKENALLRYLLDKDDTKDIGLPEITPKLERLDSKTDPASNTK
LIAMKTEKEEMSFEPSDQPGSELDNLEEILDDLQNSQLPQLFPDTRPGAPAGSVDKQAII
NDLMQLTAENSPVTPVGAQKTALRISQSRMIGSNASRPTMPSGEWAPQSSAVRVTCAAAT
TSAMNRPIQGGMIRNPTASIPMRPNSQPGQRQMLQSQVMNIGPSELEMNMGGPQYSQQQA
PPNQTAPWPESILPIDQASFANQNRQPFGSSPDDLLCPHPAAESPSDEGALLDQLYLALR
NFDGLEEIDRALGIPELVSQSQAVDPEQFSSQDSNIMLEQKAAVFPQQYASQAQMAQGSY
PPMQDPNFHTMGQRPSYATLRMQPRPGLRPTGLVQNQPNQLRLQLQHRLQAQQNRQPLMN
QISNVSNVNLTLRPGVPTQAPINAQMLAQRQREILNQHLRQRQMHQQQQVQQRTLMMRGQ

GLNMTSSMVAPGGLPATLSNPRMPQANAQQFPFPPNYGISQQPDPGFTGATTPQSPLMSP
RMAHTQSPMMQQSQANPAYQASSDMNGWAQGNMGGNSMFSQQSPPHFGQQANTSMYNNNM
NINVSMATNTGGMNNMNQMTGQISMTSVTSVPTSGLSSMGPEQKYC
>189Canis lupus familiaris|A0A5F4C4H2|Mammalia|SRC2
MYLLFSPKRSTEKRNREQENKYIEELAELIFANFNDIDNFNFKPDKCAILKETVKQIRQI
KEQEKAAAANIDEVQKSDVSSTGQGVIDKDALGPMMLEALDGFFFVVNLEGNVVFVSENV
TQYLRYNQEELMNKSVYGILHVGDHTEFVKNLLPKSIVNGGSWSGEPPRRNSHTFNCRML
VKPLPDSEEEGHDNQEAHQKYETMQCFAVSQPKSIKEEGEDLQSCLICVARRVPMKERPV
LPSSESFTTRQDLQGKITSLDTSTMRAAMKPGWEDLVRRCIQKFHAQHEGESVSYAKRHH
HEVLRQGLAFSQIYRFSLSDGTLVAAQTKSKLIRSQTTNEPQLVISLHMLHREQNVCVMN
PDLTGQAMGKPLNPISSSSPAHQAMCSGNPGQDMTLSSNINFPINGPKEQMGMPMGRFGG
SGGMNHVSSMQATTPQGSNYALKMNSPSQSSPGMNPGQPNSMLSPRHRMSPGVAGSPRIP
PSQFSPAGSLHSPVGVCSSTGNSHSYTNSSLNALQALSEGHGVSLGSSLASPDLKMGNLQ
NSPVNMNPPPLSKMGSLDSKDCFGLYGEPSEGTTGPAESSCHPGEQKETNDSNMPPAVSS
ERADGQNRLHDSKGQTKLLQLLTTKSDQMEPSPLSSSLSDANKDSTAGLPGSGSTHGTSL
KEKHKILHRLLQDSSSPVDLAKLTAEATGKELSQEASSTAPGSEVTIKQEPVSPKKKENA
LLRYLLDKDDTKDIGLPEITPKLERLDSKTDPASNTKLIAMKTEKEEMSFEPSDQPGSEL
DNLEEILDDLQNSQLPQLFPDTRPGAPAGSVDKQAIINDLMQLTAENSPVTPVGAQKTAL
RISQSTFNNPRPGQLGRLLPNQNLPLDITLQSPTGAGPFPPIRNNSPYSVIPQPGMMGNQ
GMIGNQGNLGNSSTGMIGSNASRPTMPSGEWAPQSSAVRVTCAAATTSAMNRPIQGGMIR
NPTASIPMRPNSQPGQRQMLQSQVMNIGPSELEMNMGGPQYSQQQAPPNQTAPWPESILP
IDQASFANQNRQPFGSSPDDLLCPHPAAESPSDEGALLDQLYLALRNFDGLEEIDRALGI
PELVSQSQAVDPEQFSSQDSNIMLEQKAAVFPQQYASQAQMAQGSYPPMQDPNFHTMGQR
PSYATLRMQPRPGLRPTGLVQNQPNQLRLQLQHRLQAQQNRQPLMNQISNVSNVNLTLRP
GVPTQAPINAQMLAQRQREILNQHLRQRQMHQQQQVQQRTLMMRGQGLNMTSSMVAPGGL
PATLSNPRMPQANAQQFPFPPNYGISQQPDPGFTGATTPQSPLMSPRMAHTQSPMMQQSQ
ANPAYQASSDMNGWAQGNMGGNSMFSQQSPPHFGQQANTSMYNNNMNINVSMATNTGGMN
NMNQMTGQISMTSVTSVPTSGLSSMGPEQVNDPALRGGNLFPNQLPGMDMIKQEGDASRK
YC
>190Corvus moneduloides|A0A851XH76|Aves|SRC2
MSGMGENTSDPSRAESRKRKDPDQVGPSPKRSTEKRNREQENKYIEELAELIFANFNDID
NFNFKPDKCAILKETVKQIRQIKEQEKAAAANEDEVQKADVSSTGQSVIDKDALGPMMLE
ALDGFFFVVNPEGNVVFVSENVTQYLRYNQEELMNTSIYSILHVGDHNEFVKILLPKSLV
NGGSWSGDTPRRNSHTFNCRMLVKPLADSEEGHDNQEAHQKYETMQCFAVSQPNSFKEDG
EDLQSCLICVARRAPMKERPLLPSSESFTTRQDLQGKITSLDTSTMRAAMKPGWEDLVRR
CIQRFHSQHEGEISFAKRHHQEVLRQGLAFSPVYRFSLSDGTIVSAQTKSKLIRSQTTSE
PQLVISLHMLHREQNVCGMNQDLTGQGMGKILNPISSSSPAHQAMCSGNPGQDMTISSNI
NFAINGPKEQVGMPAGRFGGSGGMNHVSSIQASTPQGSNYALKMNSPSQSSPGMNPGQPN
SMLSPRHRVSPGVAGSPRIPPSQFSPAGSLHSPVGVCSSTGNSHNYTNSSLNALQALSEG
HGVSLGSSLASPDLKMGNLQNSPVNMNPPQLSKMGSLDSKDCFGIYGEQSEGTTGQAESS
CHSGEQKDNSDSNMPPVVSGERPDGQNKLHDGKSQTKLLQLLTTKSDQMEPSPLSSTMGD
ISKDSAGGLPGSGSAHGTSLKEKHKILHRLLQDSSSPVDLAKLTAEATGKELNQESSSTA
PGSEVTVKQEPASPKKNNNALLRYLLDKDDTKDIGLPDIPPKLERLDSKTDPSGSTKLIA
MRTEKEEISFEPNEQPGSELDNLEEILDDLHNSQLPQLFPDSRPGVPAGSVDKQAIINDL
MQLTSDSSPGAAVATQKPAMRISQSTFNNPRAGQLGRLLPNQNLPLDITLQNPTGAGTFP
PIRTSSPYSVIPQPGLMGRNEGMIGSQGTLGNSSTGMMGGNAPRPAMASGDWGSQAPAVR
VTCAPTAGAMNRPVQAGMIRNPTASIPLRPSNQPGPRQMLPSQVMNMGPSELEMNIGGPQ
YSQQQAPPNQTAPWPDSILPIEQATFGSQNRQPFGSSPDDLLCNHPSSESPSDEGALLDQ
LYMALRNFDGLEEIDRALGIPELVSQSQAVDPETFPSQESSMLMEQKAPVFSQQYAAQAQ
MAQGSYAPMQDPGFHGMGQRPGYTALRMQPRPGLRPAGIVQNQPNQLRLQLQHRLQAQQN
RQPLINQISSVSNMNLSLRTGVPTQGPINAQMLAQRQREILSQHLRQRQMQQQQQVQQRT
LMMRGQGLSMTPSMVATGGIPATMSNPRIPQANAQQFPFPPNYGISQQPDPGFTGATTPQ
SPLMSPRLAHTQSPMIQQSQANPAYQSSTEMNGWAQGNMGGNSMFSQQSPPHFGQQASTS
MYNNNMNINVSMATNTSGMNNMNQMTGQISMTSATSVPTSGLSSMGPEQVNDPAMRGGGN

LFPNQLPGMDMIKQDGDAAR

>191Larus smithsonianus|A0A852GZL3|Aves|SRC2
MSGMGENTSDPSRAESRKRKDPDQLGPSPKRSTEKRNREQENKYIEELAELIFANFNDIDNFNFKPDKCAILKE
TVKQIRQIKEQEKAAAANEDEVQKADVSSTGQSVIDKDALGPMMLEALDGFFFVVNPEGNVVFVSENVTQYLRY
NQEELMNTSIYSILHVGDHNEFVKILLPKSLVNGGSWSGDTPRRNSHTFNCRMLVKPLADSEEGHDNQEAHQKY
ETMQCFAVSQPNSFKEDGEDLQSCLICVARRAPMKERPLLPSSESFTTRQDLQGKITSLDTSTMRAAMKPGWED
LVRRCIQRFHSQHEGEISFAKRHHQEVLRQGLAFSPVYRFSLSDGTIVSAQTKSKLIRSQTTSEPQLVISLHML
HREQNVCGMNQDLTGQGMGKILNPISSSSPAHQAMCSGNPGQDMTISSNINFAINGPKEQMGMPAGRFGGSGGM
NHVSSIQASTPQGSNYALKMNSPSQSSPGMNPGQPNSMLSPRHRVSPGVAGSPRIPPSQFSPAGSLHSPVGVCS
STGNSHNYTNSSLNALQALSEGHGVSLGSSLASPDLKMGNLQNSPVNMNPPQLSKMGSLDSKDCFGLYGEQSEG
TTGQAESSCHSGEQKDNSDSNMPPVVSGERPDGQNKLHDGKSQTKLLQLLTTKSDQMEPSPLSSTMGDVSKDST
GGLPGSGSAHGTSLKEKHKILHRLLQDSSSPVDLAKLTAEATGKELNQESSSTAPGSEVTVKQEPASPKKNNNA
LLRYLLDKDDTKDIGLPDIPPKLERLDSKTDPSGSTKLIAMRTEKEEINFEPNEQPGSELDNLEEILDDLQNSQ
LPQLFPESRPGAPAGSVDKQAIINDLMQLTSDSSPGAAVATQKPAMRISQSTFNNPRAGQLGRLLPNQNLPLDI
TLQNPTGAGTFPPIRTSSPYSVIPQPGLMGRNEGMIGNQGTLGNSSTGMIGGNAPRPAMTSGDWGSQAPAVRVT
CAPTASAMNRPVQAGMIRNPTASIPLRPSNQPGPRQMLPSQVMNMGPSELEMNIGGPQYSQQQAPPNQTAPWPD
SILPIDQATFGNQNRQPFGSSPDDLLCNHPSSESPSDEGALLDQLYMALRNFDGLEEIDRALGIPELVSQSQAV
DPETFPSQESGILMEQKAPVFSQQYAAQAQMAQGSYAPMQDPSFHPMGQRPGYAALRMQPRPGLRPAGIVQNQP
NQLRLQLQHRLQAQQNRQPLINQISSVSNMNLSLRPGVPTQGPINAQMLAQRQREILSQHLRQRQMQQQQQVQQ
RTLMMRGQGLSMTPSMVATGGIPATMSNPRIPQANAQQFPFPPNYGISQQPDPGFTGATTPQSPLMSPRLAHTQ
SPMIQQSQANPAYQSSTEMNGWAQGNMGGNSMFSQQSPPHFGQQASTSMYNNNMNISVSMATNTSGMNNMNQMT
GQISMTSATSVPTSGLSSMGPEQVNDPAMRGGNLFPNQLPGMDMIKQDGDATR
>192Homo sapiens|Q15596|Mammalia|SRC2
MSGMGENTSDPSRAETRKRKECPDQLGPSPKRNTEKRNREQENKYIEELAELIFANFNDIDNFNFKPDKCAILK
ETVKQIRQIKEQEKAAAANIDEVQKSDVSSTGQGVIDKDALGPMMLEALDGFFFVVNLEGNVVFVSENVTQYLR
YNQEELMNKSVYSILHVGDHTEFVKNLLPKSIVNGGSWSGEPPRRNSHTFNCRMLVKPLPDSEEEGHDNQEAHQ
KYETMQCFAVSQPKSIKEEGEDLQSCLICVARRVPMKERPVLPSSESFTTRQDLQGKITSLDTSTMRAAMKPGW
EDLVRRCIQKFHAQHEGESVSYAKRHHHEVLRQGLAFSQIYRFSLSDGTLVAAQTKSKLIRSQTTNEPQLVISL
HMLHREQNVCVMNPDLTGQTMGKPLNPISSNSPAHQALCSGNPGQDMTLSSNINFPINGPKEQMGMPMGRFGGS
GGMNHVSGMQATTPQGSNYALKMNSPSQSSPGMNPGQPTSMLSPRHRMSPGVAGSPRIPPSQFSPAGSLHSPVG
VCSSTGNSHSYTNSSLNALQALSEGHGVSLGSSLASPDLKMGNLQNSPVNMNPPPLSKMGSLDSKDCFGLYGEP
SEGTTGQAESSCHPGEQKETNDPNLPPAVSSERADGQSRLHDSKGQTKLLQLLTTKSDQMEPSPLASSLSDTNK
DSTGSLPGSGSTHGTSLKEKHKILHRLLQDSSSPVDLAKLTAEATGKDLSQESSSTAPGSEVTIKQEPVSPKKK
ENALLRYLLDKDDTKDIGLPEITPKLERLDSKTDPASNTKLIAMKTEKEEMSFEPGDQPGSELDNLEEILDDLQ
NSQLPQLFPDTRPGAPAGSVDKQAIINDLMQLTAENSPVTPVGAQKTALRISQSTFNNPRPGQLGRLLPNQNLP
LDITLQSPTGAGPFPPIRNSSPYSVIPQPGMMGNQGMIGNQGNLGNSSTGMIGNSASRPTMPSGEWAPQSSAVR
VTCAATTSAMNRPVQGGMIRNPAASIPMRPSSQPGQRQTLQSQVMNIGPSELEMNMGGPQYSQQQAPPNQTAPW
PESILPIDQASFASQNRQPFGSSPDDLLCPHPAAESPSDEGALLDQLYLALRNFDGLEEIDRALGIPELVSQSQ
AVDPEQFSSQDSNIMLEQKAPVFPQQYASQAQMAQGSYSPMQDPNFHTMGQRPSYATLRMQPRPGLRPTGLVQN
QPNQLRLQLQHRLQAQQNRQPLMNQISNVSNVNLTLRPGVPTQAPINAQMLAQRQREILNQHLRQRQMHQQQQV
QQRTLMMRGQGLNMTPSMVAPSGMPATMSNPRIPQANAQQFPFPPNYGISQQPDPGFTGATTPQSPLMSPRMAH
TQSPMMQQSQANPAYQAPSDINGWAQGNMGGNSMFSQQSPPHFGQQANTSMYSNNMNINVSMATNTGGMSSMNQ
MTGQISMTSVTSVPTSGLSSMGPEQVNDPALRGGNLFPNQLPGMDMIKQEGDTTRKYC

>193Eurypyga helias|A0A093LVM6|Aves|SRC2
KMSGMGENTSDPSRAESRKRKDPDQLGPSPKRSTEKRNREQENKYIEELAELIFANFNDIDNFNFKPDKCAILK
ETVKQIRQIKEQEKAAAANEDEVQKADVSSTGQSVIDKDALGPMMLEALDGFFFVVNPEGNVVFVSENVTQYLR
YNQEELMNTSIYSILHVGDHNEFVKILLPKSLVNGGSWSGDTPRRNSHTFNCRMLVKPLADSEEGHDNQEAHQK
YETMQCFAVSQPNSFKEDGEDLQSCLICVARRAPMKERPLLPSSESFTTRQDLQGKITSLDTSTMRAAMKPGWE
DLVRRCIQRFHSQHEGEISFAKRHHQEVLRQGLAFSPVYRFSLSDGTIVSAQTKSKLIRSQTTSEPQLVISLHM
LHREQNVCGMNQDLTGQGMGKILNPISSSSPAHQAMCSGNPGQDMTISSNINFAINGPKEQMGMPAGRFGGSGG
MNHVSSIQASTPQGSNYALKMNSPSQSSPGMNPGQPNSMLSPRHRVSPGVAGSPRIPPSQFSPAGSLHSPVGVC
SSTGNSHNYTNSSLNALQALSEGHGVSLGSSLASPDLKMGNLQNSPVSMNPPQLSKMGSLDSKDCFGLYGEQSE

GTTGQAESSCHSGEQKDNGDSNMPPVVSGERPDGQNKLHDGKSQTKLLQLLTTKSDQMEPSPLSSTMGDVNKDS
TGGLPGSGSAHGTSLKEKHKILHRLLQDSSSPVDLAKLTAEATGKELNQESGSTAPGSEVTVKQEPASPKKNNN
ALLRYLLDKDDTKDIGLPDIPPKLERLDNKTDPSGSTKLIPMRTEKEEISFEPNEQPGSELDNLEEILDDLQNS
QLPQLFPDSRPGAPAGSVDKQAIINDLMQLTSDSSPGAAVATQKPAMRISQSSGGTFPPIRTSSPYSVIPQPGL
MGRNEGMIGSQGTLGNSSTGMIAGNAPRPAMTSGDWGSQAPAVRVTCAPTASAMNRPVQAGMIRNPTASIPLRP
SNQPGPRQMLPSQVMNMGPSELEMNIAGPQYSQQQAPPNQTAPWPDSILPIDQSTFGSQNRQPFGSSPDDLLCN
HPSSESPSDEGALLDQLYMALRNFDGLEEIDRALGIPELVSQSQAVDPETFSSQESSILMEQKAPVFSQQYAAQ
AQMAQGSYAPMQDPSFHPMGQRPGYAALRMQPRPGLRPAGIVQNQPNQLRLQLQHRLQAQQVHPLPCPASRGPT
DCQLEPNRPDCSSGDASTGQVVGLLTSGGGFPCRAPSMRRSQRHATGYTRLLKKMQQQQQQQVQQRTLMMRGQG
LSMTPSMVAAGGIPATMSNPRIPQANAQQFPFPPNYGISQQPDPGFTGATTPQSPLMSPRLAHTQSPMIQQSQA
NPAYQSSTEMNGWAQGNMGGN
>194Physeter macrocephalus|A0A2Y9FRB3|Mammalia|SRC2
MSGMGENTSDPSRAETRKRKECPDQLGPSPKRSTEKRNREQENKYIEELAELIFANFNDIDNFNFKPDKCAILK
ETVKQIRQIKEQEKAAATNIDEVQKSDVSSTGQGVIDKDALGPMMLEALDGFFFVVNLEGNVVFVSENVTQYLR
YNQEELMNKSVYSILHVGDHTEFVKNLLPKSIVNGGAWSGEPPRRNSHTFNCRMLVKPLPDSEEEGHENQEAHQ
KYETMQCFAVSQPKSIKEEGEDLQSCLICVARRVPMKERPVLPSSESFTTRQDLQGKITSLDTSTMRAAMKPGW
EDLVRRCIQKFHAQHEGESVSYAKRHHHEVLRQGLAFSQIYRFSLSDGTLVAAQTKSKLIRSQTTNEPQLVISL
HMLHREQNVCVMNPDLTGQAMGKPLNPISSGSPAHQAMCSGNPGQDMTLSSNINFPMNGPKEQVGMPMGRFGGS
GGMNHVSSMQAATPQGSNYALKMNSPSQSSPGLNPGQPSSMLSPRHRMSPGVAGSPRIPPSQFSPAGSLHSPVG
VCSSTGNSHSYTNSSLNALQALSEGHGVSLGSSLASPDLKMGNLQNSPVHMNPPPLSKMGSLDSKDCFGLYGEP
SEGPAGPAESSCHPGQQKEASEAGLPQAGSGERPDGQSRLHDGKGQTKLLQLLTTKSDQMEPSPLPGALSDTHK
DSTGSLPGSGSTHGTSLKEKHKILHRLLQDSSSPVDLAKLTAEATGKELNQEASSTAPGSEVTIKQEPVSPKKK
ENALLRYLLDKDDTKDIGLPEITPKLERLDSKTDPASNTKLITMKTEKEEMSFEPSDQPGSELDNLEEILDDLQ
NSQLPQLFPDTRPGAPAGSVDKQAIINDLMQLTAESSPVTPVGAQKTALRISQSTFNNPRPGQLGRLLPNQNLP
LDITLQSPTGAGPFPPIRNNSPYSVIPQPGMMGNQGMIGNQGNLGNSSTGMIGSNAARPTLPSGEWAPQSSAVR
VTCAATTSAMNRPIQGGMIRNPTASIPMRPNSQPGQRQMLQPQVMNIGPSELEMNIGGPQYSQQQAPPNQTAPW
PESILPIEQAPFASQNRQPFGNSPDDLLCPHPAAESPSDEGALLDQLYLALRNFDGLEEIDRALGIPELVSQSQ
AVDPEQFPSQDSNILLEQKAPVFPQQYASQAQMAQGSYTAMQDPSFHTMGQRPSYATLRVQPRPGLRPTGLAQT
QPNQLRLQLQHRLQAQQNRQPLMNQISNVSNVNLTLRPGVPTQAPINAQMLAQRQREILNQHLRQRQMHQQQQV
QQRTLMMRGQGLNMTPSVAAPSGVPATMSSPRVPQANAQQFPFPPNYGISQQPDPGFAGATAPQSPLMSPRLAH
TQSPMLQQSQANPAYQASSDMNGWAQGSMGANSMFSQQSPPHFGQQANTSMYNNNMNISVSMATNTGGMGNMNQ
MTGQISMTSVTSVPTSGLSSMGPEQVNDPALRGGSLFPSQLPGMDMIKQEGDAPRKYC

>195Balaenoptera acutorostrata|A0A384ACK4|Mammalia|SRC2
MSGMGENTSDPSRAETRKRKECPDQLGPSPKRSTEKRNREQENKYIEELAELIFANFNDIDNFNFKPDKCAILK
ETVKQIRQIKEQEKAAAANIDEVQKSDVSSTGQGVIDKDALGPMMLEALDGFFFVVNLEGNVVFVSENVTQYLR
YNQEELMNKSVYSILHVGDHTEFVKNLLPKSIVNGGAWSGEPPRRNSHTFNCRMLVKPLPDSEEEGHENQEVHQ
KYETMQCFAVSQPKSIKEEGEDLQSCLICVARRVPMKERPVLPSSESFTTRQDLQGKITSLDTSTMRAAMRPGW
EDLVRRCIQKFHAQHEGESASHAKRHHHEVLRQGLAFSQIYRFSLSDGTLVAAQTKSKLIRSQTTNEPQLVISL
HMLHREQNVCVMNPDLTGQAMGKPLNPISSSSPAHQAMCSGNPGQDMTLSSNINFPMNGPKEQMGMPMGRFGGS
GGMNHVSSMPAATPQGSNYALKMNSPSQGSPGLNPGQPSSMLSPRHRMSPGVAGSPRIPPSQFSPAGSLHSPVG
VCSSTGNSHSYTNSSLNALQALSEGHGVSLGSSLASPDLKMGNLQNSPVNMNPPPLSKMGSLDSKDCFGLYGEP
SEGPAGPAESSCHPGEQKDAGEAGGGERPDGQGRLHDGKGQTKLLQLLTTKSDQTEPSPLPGALSDTSKDSTGS
LPGSGSTHGTSLKEKHKILHRLLQDSSSPVDLAKLTAEATGKELSQEASSTAPGSEVTIKQEPVSPKKKENALL
RYLLDKDDTKDIGLPEITPKLERLDSKTDPASNTKLITMKTEKEEMSFEPGDQPGSELDNLEEILDDLQNSQLP
QLFPDTRPGAPAGSVDKQAIINDLMQLTAESSPVTPAGAQKTALRISQSTFNNPRPGQLGRLLPNQNLPLDITL
QSPTGAGPFPPIRNSSPYSVIPQPGMMGNQGMIGNQGNLGNSSTGMIGSNAARPTLPSGEWAPQSAAVRVTCAA
ATSAMNRPIQGGMIRNPTASIPMRPNSQPGQRQMLQPQVMNIGPSELEMNIGGPQYSQQQAPPNQTAPWPESIL
PIEQAPFASQNRQPFGNSPDDLLCPHPAAESPSDEGALLDQLYLALRNFDGLEEIDRALGIPELVSQSQAVDPE
QFPSQDSSIMLEQKAPVFPQQYAPQAQMAQGSYTPMQDPSFHPMGQRPSYATLRMQPRPGLRPTGLVQSQPNQL
RLQLQHRLQAQQNRQPLMNQISNVSNVNLTLRPGVPTQAPINAQMLAQRQREILNQHLRQRQMHQQQQVQQRTL
MMRGQGLNMAPSVVAPSGVPATMSSPRVPQANAQQFPFPPNYGISQQPDPGFAGATAPQSPLMSPRLAHTQSPM
LQQSQANPAYQASSDMNGWAQGSMGANSMFSQQSPPHFGQQANTSMYNNNMNINVSMATNTGGMSNMNQMTGQI
SMTSVTSVPTSGLSSMGPEQVNDPALRGGSLFPSQLPGMDMIKQEGDAPRKYC

>196Ursus maritimus|A0A452UWS9|Mammalia|SRC2
MSGMGENTSDPSRAETRKRKECPDQLGPSPKRSTEKRNREQENKYIEELAELIFANFNDIDNFNFKPDKCAILK
ETVKQIRQIKEQEKAAAANVDEVQKSDVSSTGQGVIDKDALGPMMLEALDGFFFVVNLEGNVVFVSENVTQYLR
YNQEELMNKSVYSILHVGDHTEFVKNLLPKSIVNGGSWSGEPPRRNSHTFNCRMLVKPLPESEEEGHDNQEAHQ
KYETMQCFAVSQPKSIKEEGEDLQSCLICVARRVPMKERPVLPSSESFTTRQDLQGKITSLDTSTMRAAMKPGW
EDLVRRCIQKFHAQHEGESVSYAKRHHHEVLRQGLAFSQIYRFSLSDGTLVAAQTKSKLIRSQTTNEPQLVISL
HMLHREQNVCVMNPDLTGQAMGKPLNPISSSSPAHQAMCSGNPGQDMTLGSNINFPINGPKEQMGMPMGRFGGS
GGMNHVSSMQATTPQGSNYALKMNSPSQSSPGMNPGQPNSMLSPRHRMSPGVAGSPRIPPSQFSPAGSLHSPVG
VCSSTGNSHSYTNSSLNALQALSEGHGVSLGSSLASPDLKMGNLQNSPVNMNPPPLSKMGSLDSKDCFGLYGEP
SEGTTGQAESSCHPGEQKETNDSNMPQAVSAERADGQNRLHDSKGQTKLLQLLTTKSDQMEPSPLSSSLSDTNK
DSTASLPGSGSTHGTSLKEKHKILHRLLQDSSSPVDLAKLTAEATGKELSQEANSTAPGSEVTIKQEPVSPKKK
ENALLRYLLDKDDTKDIGLPEITPKLERLDSKTDPASNTKLIAMKTEKEEMSFEPSEQPGSELDNLEEILDDLQ
NSQLPQLFPDTRPGAPAGSVDKQAIINDLMQLTAENSPVTPVGAQKTALRISQSTFNNPRPGQLGRLLPNQNLP
LDITLQSPTGAGPFPPIRNSSPYSVIPQPGMMGNQGMIGNQGNLGNSSTGMIGSNASRPTMPSGEWAPQSSAVR
VTCAATTSAMNRPIQGGPSELEMNMGGPQYSQQQAPPNQTAPWPESILPIDQASFASQNRQPFGNSPDDLLCPH
PAAESPSDEGALLDQLYLALRNFDGLEEIDRALGIPELVSQSQAVDPEQFSSQDSNIMLEQKAAVFPQQYASQA
QMAQGSYPPMQDPNFHTMGQRPSYATLRMQPRPGLRPAGLVQNQPNQLRLQLQHRLQAQQNRQPLMNQISNVSN
VNLTLRPGVPTQAPINAQMLAQRQREILNQHLRQRQMHQQQQVQQRTLMMRGQGLNMTSSMVAPGGLPATLSNP
RMPQANAQQFPFPPNYGISQQPDPGFTGATTPQSPLMSPRMAHTQSPMMQQSQANPAYQASSDMNGWAQGNMGG
NSMFSQQSPPHFGQQANTSMYNNNMNINVSMATNTGGMSNMNQMTGQISMTSVTSVPTSGLSSMGPEQKYC
>197Bos indicus x Bos taurus|A0A4W2CJC9|Mammalia|SRC2
MSGMGENTPDPSRAETRKRKECPDQLGPSPKRSTEKRNREQENKYIEELAELIFANFNDIDNFNFKPDKCAILK
ETVKQIRQIKEQEKAAAANIDEVQKSDVSSTGQGVIDKDALGPMMLEALDGFFFVVNLEGNVVFVSENVTQYLR
YNQEELMNKSVYSILHVGDHTEFVKNLLPKSIVNGGAWSGEPPRRNSHTFNCRMLVKPLPESEEEGHDNQEAHQ
KYETMQCFAVSQPKSIKEEGEDLQSCLICVARRVPMKERPALPSSESFTTRQDLQGKITSLDTSTMRAAMKPGW
EDLVRRCIQKFHAQHEGESVSYAKRHHHEVLRQGLAFSQIYRFSLSDGTLVAAQTKSKLIRSQTTNEPQLVISL
HVLHREQNVCVMNPDLTGQAMGKPLNPISSSSPAHPAMCSGNPGQDMTLSSNINFPMNGPKEQMGMPMGRFGGS
GGMNHVSSMQATTPQGSNYALKMNSPSQSSPGLNPGQPSSMLSPRHRMSPGVAGSPRIPPSQFSPAGSLHSPVG
VCSSTGNSHSYTNSSLNALQALSEGHGVSLGSSLASPDLKMGNLQNSPVNMNPPQLSKMGSLDSKDCFGLYGEP
PEGPAGPAESSCHPGEPKDPSEAGLPPAGSNERPDGQSRLLDNKGQTKLLQLLTTKSDQIEPSPLPSSLSDTNK
DSTGSLPGPGSTHGTSLKEKHKILHRLLQDSSSPVDLAKLTAEATGKELSQEASSTAPGSEATIKQEPVSPKKK
ENALLRYLLDKDDTKDIGLPEITPKLERLDSKTDPASNTKLIALKTEKEEMSFEPSDQPGSELDNLEEILDDLQ
NSQLPQLFPDTRPGAPAGSVDKQAIINDLMQLTAESSPVTPVGAQKTAMRIPQSTFNNPRPGQLGRLLPNQNFP
LDITLQSPTGAGPFPPIRNSSPYPVIPQPGMMGNQGMIGNQGNLGNSSTGMIGSNAARPTMPSGEWAPQSPAVR
VTCAATSNAMNRPIQGGMIRNPTASIPMRPNSQPGQRQMLQPQVMNMGPSELEMNMGGPQYSQQQAPPNQTAPW
PESILPIDQAPFASQNRQPFGSSPDDLLCPHPAAESPSDEGALLDQLYMALRNFDGLEEIDRALGIPELVSQAP
INAQMLAQRQREILNQHLRQRQMHHQQQQVQQRTLMMRGQGLNMTPSVVAPSGVPAAMSNPRIPQANAQQFAFP
PNYGISQQPDPGFTGAAAPQSPLISPRLAHTQSPMLQQSQANPAYQASSDMNGWAQGSMGANSMFSQQSPPHFA
QQANTSMYNNNMNINVSMATNTGGMNNMNQMTGQISMTSVTSVPTSGLSSMGPEQVNDPALRGGGLFPSQLPGM
DMIKQEGDASRKYC
>198Bos indicus x Bos taurus|A0A4W2GB79|Mammalia|SRC2
MSGMGENTPDPSRAETRKRKECPDQLGPSPKRSTEKRNREQENKYIEELAELIFANFNDIDNFNFKPDKCAILK
ETVKQIRQIKEQEKAAAANIDEVQKSDVSSTGQGVIDKDALGPMMLEALDGFFFVVNLEGNVVFVSENVTQYLR
YNQEELMNKSVYSILHVGDHTEFVKNLLPKSIVNGGAWSGEPPRRNSHTFNCRMLVKPLPESEEEGHDNQEAHQ
KYETMQCFAVSQPKSIKEEGEDLQSCLICVARRVPMKERPALPSSESFTTRQDLQGKITSLDTSTMRAAMKPGW
EDLVRRCIQKFHAQHEGESVSYAKRHHHEVLRQGLAFSQIYRFSLSDGTLVAAQTKSKLIRSQTTNEPQLVISL
HVLHREQNVCVMNPDLTGQAMGKPLNPISSSSPAHPAMCSGNPGQDMTLSSNINFPMNGPKEQMGMPMGRFGGS
GGMNHVSSMQATTPQGSNYALKMNSPSQSSPGLNPGQPSSMLSPRHRMSPGVAGSPRIPPSQFSPAGSLHSPVG
VCSSTGNSHSYTNSSLNALQALSEGHGVSLGSSLASPDLKMGNLQNSPVNMNPPQLSKMGSLDSKDCFGLYGEP
PEGPAGPAESSCHPGEQKDPSEAGLPPAGSNERPDGQSRLLDNKGQTKLLQLLTTKSDQIEPSPLPSSLSDTNK
DSTGSLPGPGSTHGTSLKEKHKILHRLLQDSSSPVDLAKLTAEATGKELSQEASSTAPGSEATIKQEPVSPKKK
ENALLRYLLDKDDTKDIGLPEITPKLERLDSKTDPASNTKLIALKTEKEEMSFEPSDQPGSELDNLEEILDDLQ
NSQLPQLFPDTRPGAPAGSVDKQAIINDLMQLTAESSPVTPVGAQKTAMRIPQSTFNNPRPGQLGRLLPNQNFP

LDITLQSPTGAGPFPPIRNSSPYPVIPQPGMMGNQGMIGNQGNLGNSSTGMIGSNAARPTMPSGEWAPQSPAVR
VTCAATSNAMNRPIQGGMIRNPTASIPMRPNSQPGQRQMLQPQVMNMGPSELEMNMGGPQYSQQQAPPNQTAPW
PESILPIDQAPFASQNRQPFGSSPDDLLCPHPAAESPSDEGALLDQLYMALRNFDGLEEIDRALGIPELVSQAP
INAQMLAQRQREILNQHLRQRQMHHQQQQVQQRTLMMRGQGLNMTPSVVAPSGVPAAMSNPRIPQANAQQFPFP
PNYGISQQPDPGFTGAAAPQSPLISPRLAHTQSPMLQQSQANPAYQASSDMNGWAQGSMGANSMFSQQSPPHFA
QQANTSMYNNNMNINVSMATNTGGMNNNMNQMTGQISMTSVTSVPTSGLSSMGPEQVNDPALRGGGLFPSQLPGM
DMIKQEGDASRKYC
>199Thamnophis sirtalis|A0A6I9XX79|Sarcopterygii|SRC2
MSGIGENSSDPARAELRKRKECPDQLGPSPKRSTEKRNREQENKYIEELAELIFANFNDIDNFNFKPDKCAILK
ETVKQIRQIKEQEKAAAANEDEVQKADVSSTGQSVIDKDALGPMMLEALDGFFFVVNPEGNVVFVSENVTQYLR
YNQEELMNTRVYNILHVGDHNEFVRNLLPNSRMNGGSWSGESPRRNSHTFNCRMLVKPLTDSEEESHENQEAHQ
KYETMQCFAVSQPKSIKEDGEDLQSCLICVARRVPMKERPLLPSSETFSTRQDLQGKITSLDTSTMRAAMKPGW
EDVVRRCIQRFYAQHEGDISFAKRHHQEVLRQGLAFSPVYRFSLSDGTIVAAQTKSKLIRSQTTSEPQLVISLH
MLHREQNVCGMNQDLTGQVLGKPLNTISSNSPAHQAMCSGNPGQDMTINSNMNFAINGPKEQMGMPTSRFSSSG
GMNHVSSIQTSTPQGSNYALKMNSPSQNSPGMTPGQPNSMLSPRHRVSPGVAGSPRIPPNQFSPAGNLHSPVGV
CSSTGNSHSYTNSSLNALQALSEGHGVSLGSSLASPDIKMGNLQNSPVSMNPPQLTKMGNLDSKDSFGLHGEQS
EETTGQAESVCHSGEQKENNDNMSQVGSERPDGQNRLHDGKSQTKLLQLLTTKSDQMEPSSLANTMGDANKDSM
GSLSGSGSAHGTSLKEKHKILHRLLQDSSSPVDLAKLTAEATGKELNQETSSTAPGSEITCKQEPVSPKKKENA
LLRYLLDKDDTKEIGLPDITPKLERSDSKTDPSSSTVKPVAIKTEKEEMRFEPNEQPGSELDNLEEILDDLQNS
QLSQLFPDTRQVASAGSVDKQAIINDLMQITGDNSPGTPVGSQKPTGRISQSTFTNARQAQLGRMLPNQNLQLD
IALQNAPGAATFPPIRNNSPYSVIPQPSMLGSQGMMGNQGNLGNNNPGMIAGNTPRPAMSSGDWGSQAAAVRVT
CAATTSAMNRPIQGGMIRNPTPSLPLRSNSQAVPRQILQSQVMNMGGSELEMNMGAPQYTQQQAPPNQTAPWPD
SILPIDQASFSSQNSCSSSCALCRQPFVGSPDDLMCQHPVSESPSDEGALFDQLYMALRNFDGLEEIDRALGIP
ELVSQTQGVDPESFSNQEPNIILEQKAPVFTQQYASQAQMAQGNYTPLQDPNFHPMAQRPGYAALRMQPRPGLR
PTGLVQNPPNQLRLQLQHRLQAQQNRQPLINQIGNVSSMNLSLRPGVPTQGNINSQMLAQRQRELLSQHLRHRQ
MHQVQQRTLMMRGQSMNMTPSMVASGGIPATMSNPRIPQTNPQQFPFPPNYGINQQPDPGFTGATTPQSPLMSP
RMAHTQNPLIQQSQANPAYQSSTEINGWGQGNIGGNSMFSQQSPPHFGQQANTSIYNNNMSINVSMVANSAGLN
NMNQMTGQISMTSATSVPTSGLPSMGPEQVNDPAMRGGSLFPNQLPGMDMIKQEGDASRKYC
>200Bos indicus|A0A6P5CWT2|Mammalia|SRC2
MSGMGENTPDPSRAETRKRKECPDQLGPSPKRSTEKRNREQENKYIEELAELIFANFNDIDNFNFKPDKCAILK
ETVKQIRQIKEQEKAAAANIDEVQKSDVSSTGQGVIDKDALGPMMLEALDGFFFVVNLEGNVVFVSENVTQYLR
YNQEELMNKSVYSILHVGDHTEFVKNLLPKSIVNGGAWSGEPPRRNSHTFNCRMLVKPLPESEEEGHDNQEAHQ
KYETMQCFAVSQPKSIKEEGEDLQSCLICVARRVPMKERPALPSSESFTTRQDLQGKITSLDTSTMRAAMKPGW
EDLVRRCIQKFHXQHEGESVSYAKRHHHEVLRQGLAFSQIYRFSLSDGTLVAAQTKSKLIRSQTTNEPQLVISL
HVLHREQNVCVMNPDLTGQAMGKPLNPISSSSPAHPAMCSGNPGQDMTLSSNINFPMNGPKEQMGXXMGRFGGS
GGMNHVSSMQATTPQGSNYALKMNSPSQSSPGLNPGQPSSMLSPRHRMSPGVAGSPRIPPSQFSPAGSLHSPVG
VCSSTGNSHSYTNSSLNALQALSEGHGVSLGSSLASPDLKMGNLQNSPVNMNPPQLSKMGSLDSKDCFGLYGEP
PEGPAGPAESSCHPGEPKDPSEAGLPPAGSNERPDGQSRLLDNKGQTKLLQLLTTKSDQIEPSPLPSSLSDTNK
DSTGSLPGPGSTHGTSLKEKHKILHRLLQDSSSPVDLAKLTAEATGKELSQEASSTAPGSEATIKQEPVSPKKK
ENALLRYLLDKDDTKDIGLPEITPKLERLDSKTDPASNTKLIALKTEKEEMSFEPSDQPGSELDNLEEILDDLQ
NSQLPQLFPDTRPGAPAGSVDKQAIINDLMQLTAESSPVTPVGAQKTAMRIPQSSMIGSNAARPTMPSGEWAPQ
SPAVRVTCAATSNAMNRPIQGGMIRNPTASIPMRPNSQPGQRQMLQPQVMNMGPSELEMNMGGPQYSQQQAPPN
QTAPWPESILPIDQAPFASQNRQPFGSSPDDLLCPHPAAESPSDEGALLDQLYMALRNFDGLEEIDRALGIPEL
VSQSQGVDPEQFSSQDSSLMLEQKTPVFPQQYASQAQMAQGSYTPMQDPNFHTMGQRPSYATLRMQPRPGLRPT
GLVQSQPNQLRLQLQHRLQAQQNRQPLMNQISNVSNVNLTLRPGVPTQAPINAQMLAQRQREILNQHLRQRQMH
HQQQQVQQRTLMMRGQGLNMTPSVVAPSGVPAAMSNPRIPQANAQQFPFPPNYGISQQPDPGFTGAAAPQSPLI
SPRLAHTQSPMLQQSQANPAYQASSDMNGWAQGSMGANSMFSQQSPPHFAQQANTSMYNNNMNINVSMATNTGG
MNNNMNQMTGQISMTSVTSVPTSGLSSMGPEQVNDPALRGGGLFPSQLPGMDMIKQEGDASRKYC
>201Sus scrofa|B0FRD5|Aves|SRC2
MSGLGDSSSDPANPDSHKRKGSPCDTLASSTEKRRREQENKYLEELAELLSANISDIDSLSVKPDKCKILKKTV
DQIQLMKRMEQEKSTTDDEVQKSDISSSSQGVIEKESLGPLLLEALDGFFFVVNCEGRIVFVSENVTSYLGYNQ
EELMNTSVYSILHVGDHAEFVKNLLPKSLVNGVPWPQEATRRNSHTFNCRMLIHPPDEPGTENQEACQRYEVMQ
CFTVSQPKSIQEDGEDFQSCLICIARRLPRPPAIMGVESFMTKQDTTGKIISIDTSSLRAAGRTGWEEFMRKCI
YAFFQPQGREPSYARQLFQEVMTRGTASSPSYRFILNDGTMLSAHTKCKLCYPQSPDMQPFIMGIHIIDREHSG

LSPQDDTNSGMSIPRVNPPVNPSISPAHGVARSSSLPPSNSNMVSTRVNRQQSSDLHSSSHSNSSNSQGSFGCS
PGNQIVAGVALNQGQASSQSTNPPLNLNNSPMEGTGISLAQFMSPRRQVSSGLATRPRMPNNSFPPNIPTLNSP
VSMTSTACNNSNRSYSNIPVTSLQSMNEGPNNSVGFSAGSPVLRQMSSQNSPSRLNIQPAKAESKDNKETASIL
NEMIQSDNSSNDGKPLDSGLLHNNDRLSDGDNKYSQTSHKLVQLLTTTAEQQLRHADIDTSCKEVLSCTGTSSS
ASANSSSGSCPSSHSSLTERHKILHRLLQEGSPSDITTLSVEPDKKDSASTSVSVTGQVPGNSGIKLELDASKK
KESKDHQLLRYLLDKDEKDLRSTPNLSLDDVKVKVEKKEQMDPCNTNPTPMTKPPPEEIKLESQSQFTADLDQF
DQLLPTLEKAAQLPGLCETERMDGAVTSVTIKSEILPATLQSTTARPTSRLNRLPELELEAIDNQFGQPGTGDQ
IPWANNTVTAVNQNKPEDQCISSQLDELLCPPTTVEGRNDEKALLEQLVSFLSGKDETELAELDRALGIDKLVQ
GGGLDVLSERFPPQQATPPLMMEERPNLYSQPYSSPSPTANLSGPFQGMVRQKPSLGTMPVQVTPPRGAFSPGM
GMQPRQTLNRPPAAPNQLRLQLQQRLQGQQQLIHQNRQAILNQFAANAPVGINMRSGMQQQITPQPPLNAQMLA
QRQRELYSQQHRRRQLIQQQRAMLMRQQSFGNNLPPSSGLPVPMGNPRLPQGAPQQFPYPPNYGTNPGTPPAST
SPFSQLAENPEATLGNRNSMVNRGMTGNMGGQFGTGINPQMQQNVFQYPGSGMVPQGEANFAPSLSPGSSMVPM
PIPPPQSSLLQQTPPASGYQSPDMKAWQQGAMGNNNVFSQAVQNQPTPAQPGVYNNMSITVSMAGGNTNVQNMN
PMMGQMQMSSLQMPGMNTVCPEQINDPALRHTGLYCNQLSSTDLLKTEADGTQDKKTEEFFSVVTTD
>202Sus scrofa|B0FRD6|Aves|SRC2
MSGMGENTSDPSRAETRKRKECPDQLGPSPKRSTEKRNREQENKYIEELAELIFANFNDIDNFNFKPDKCAILK
ETVKQIRQIKEQEKAAAANIDEVQKSDVSSTGQGVIDKDALGPMMLEALDGFFFVVNLEGNVVFVSENVTQYLR
YNQEELMNKSVYSILHVGDHTEFVKNLLPKSIVNGGSWSGEPPRRSSHTFNCRMLVKPLPDPEEEGHDNQEAHQ
KYETMQCFAVSQPKSIKEEGEDLQSCLICVARRVPMKERPVLPSSESFTTRQDLQGKITSLDTSTMRAAMKPGW
EDLVRRCIQKFHAQHEGESVSYAKRHHHEVLRQGLAFSQIYRFSLSDGTLVAAQTKSKLIRSQTTNEPQLVISL
HMLHREQNVCVMNPDLTGQAVGKPLNPLSSSSPAHQAMCSGNPGQDMTLSSNINFPINGPKEQMGMPMGRFGGS
GGMNHVSSMQATTPQGSNYALKMNSPSQSSPGMTPGQPSSMLSPRHRMSPGVAGSPRIPPSQFSPAGSLHSPVG
VCSSTGNSHSYANSSLNALQALSEGHGVSLGSSLASPDLKTGNLQNSPVNMNPPPLSKMGSLDSKDCFGLYGEP
SEGTTGQAESSGHPGEQKEAGEPGLPPAVGSERPDGQSRLHDSKGQTKLLQLLTTKSDQMEPSPLASSLSDATK
DSVGSLPGSGSTHGTSLKEKHKILHRLLQDSSSPVDLAKLTAEATGKELNQEASSTAPGSEVTIKQEPVSPKKK
ENALLRYLLDKDDTKDIGLPEITPKLERLDSKTDPASNTKLIAMKTEKEEMSFEPSDQPGSELDNLEEILDDLQ
NSQLPQLFPDTRPGAPAGSVDKQAIINDLMQLTAENSPVTPVGAQKTALRISQSTFNNPRPGQLGRLLPNQNLP
LDITLQSPTGAGPFPPIRNNSPYSVIPQPGMMGNQGMIVNQGNLGNSSTGMIGSNAARPAMPSGEWAPQTSAVR
VTCAATTGAMNRPIQGGMIRNPTASIPMRPSSQPGQRQMLQPQVMNIGPSELEMNMGPQYSQQQAPPNQTAPWP
ESILPIDQAPFASQNRQPFGSSPDDLLCPHPAAESPSDEGALLDQLYLALRNFDGLEEIDRALGIPELVSQSQA
VDPEQFSSQDSNMMLEQKAPVFPQQYASQAQMAPGSYAPMQDPNFHTMGQRPSYATLRMQPRPGLRPTGLVQNQ
PNQLRLQLQHRLQAQQNRQPLMNQISNVSNVNLTLRPGVPTQAPINAQMLAQRQREILNQHLRQRQMHQQQQVQ
QRTLMMRGQGLNMTPSVVAPGGVPAAMSNPRLPQANAQQFPFPPNYGMSQQPDPGFTGAAAPQSPLMSPRMAHT
QSPMLQQSQPNPAYQASSDMNGWAQGSMGGNSMFSQQSPPHFGPQANTSMYNNNMNISVSMATSTGGMSNMNQM
TGQISMTSVTSVPTSGLSSMGPEQVNDPALRGGSLFPNQLPGMDMIKQEGDASRKYC
>203Xenopus tropicalis|B5DE09|Amphibia|SRC2
MSGMGENPSDPSRAEPRKRKESIDQLGPSPKRSTEKRNREQENKYIEELAELIFANFNDIDNLNFKPDKCAILK
ETVKQIRQIKEHEKTAAANEDEVQKADVSSTGQSVIDKDALGPMMLEALDGFFFVVNREGNVVFVSENVTQYLR
YNQEELMNTSVYSILHVGDHSEFIKNLLPKSIVNGGPRRNSHTFNCRMLVKPMMECEEEGHDGQETHQKYETMQ
CFAVSQPKSIKEEGEDFQSCLICVARRVPMKERPVPPPSESFTTRQDLQGKITSLDTTNMRALMRPGWEDLVRR
CIQRFHSQHDGEISYSKRHHQEVLRQGHATSPFYRFALSDGTTVLAHTKSKLMRSQTTNEPQLVLSLHVLQREQ
NMCGLNQDLAGQAMGKTLNPVQSSSPAHQAMYGGNPGQDTTISSNMNYAITGPKEQMGMAAGRFVGSGGMNHIS
SLQATTPQGNNYALKINSPSQGSPGMGQGQPNSMLSPRHRVSPGVAGSPRIAPSQFSPAGSLHSPVSVCSSTGN
SHSYTNSSLNALQALSEGHGVPLAPPLSSPDLKVGNVQHSPVNMNPPQLRKMGSIDSKESFGLYGEQPESAAGQ
GESGCHSNEQKDCSENLSSVGDQTEGQSRLLDSKGQQKLLKLLTTKSDQMEPSPLPSNTLGDMNKDSLSNFASN
SMSASAHGTSLKEKHKILHRLLQDSSSPVDLAKLTAEATGKELSQESNSTGPGSEVTIKQEPVSPKKKEHALLR
YLLDKDDTKDNVADITPKLERPDVKVEPTSCPKLLSVKAEKEEPSFGHSDQLMPPGSDLDNLDEILDDLQNSQL
SQLFPDARHDGSNSADKQAIMNDLMQLAGDNSTGLPAGAHKQRMIRIQQNNGFSSQLAAQLGRLPNQSLPLDIN
LQSQVSAGSFPPMRNSAPYTTVSQSVVMNNQAMMGSQGNVSNSSPGIIGVNGPRPALKPGDWGSQASAVRPACP
TPSTAINRPIQDLTRSPTASIPMRPGGQVCPRQVLQSPVINMGSSELDMNIGGPQYTQQQAPPNQTAPWPDSIL
PIDQPSFNNQNRQPFGSPADDLICQPIASQSPTDDGNLLDQLYMALRNFDGLEEIDRALGIPEMVSQGQAVEQE
SFVSPESNLMMEQKPPLYNHAYANQGQMAQNSYTPMQDPGFNPMGQRPSYGILRMQNRPGLRPTGMVQNQPNQL
RLQLQHRLQAQNRQPIMNPINNVSNMNLAMRPGVPGQLREQGPINAQMLAQRQRELLSQHLRQKQLQQQQQQQ
QQQQQVQQHRAMMMRGQGLAMPPNMVGSGGIPASINSPRIPQGNTQQFPFPPNYGMSQQSDPGFTGATTPQSPI

MSPRMGHIQSPMMQQSQASPAYQSELNGWAQGNPAGNSMFSQQSPPHFGQQSGTSMYNSNNMNISVSMAANGNG
MNSMNQMTGQINMTSVTSVPTSGLSSMGPEQKYC
>204 Pongo abelii |A0A2J8XV51|Mammalia|SRC3
MSGLGENLDPLATDSRKRKLPCDTPGQGLTCSGEKRRREQESKYIEELAELISANLSDID
NFNVKPDKCAILKETVRQIRQIKEQGKTISNDDDVQKADVSSTGQGVIDKDSLGPLLLQA
LDGFLFVVNRDGSIVFVSENVTQYLQYKQEDLVNTSVYNILHEEDRKDFLKNLPKSTVNG
VSWTNETQRQKSHTFNCRMLMKTPHDILEDINASPEMRQRYETMQCFALSQPRAMMEEGE
DLQSCMICVARRITTGERAFPSNPESFITRHDLSGKVVNIDTNSLRSSMRPGFEDIIRRC
IQRFFSLNDGQSWSQKRHYQEAYLNGHAETPVYRFSLADGTIVTAQTKSKLFRNPVTNDR
HGFVSTHFLQREQNGYRPNPNPVGQGIRPPMAGCNSSVGGMSMSPNQGLQMPSSRAYGLA
DPSTTGQMSGARYGGSSNIASLTPGPGMQSPSSYQNNNHGLNMSSPPHGSPGLAPNQQNI
MISPRNRGSPKIASHQFSPVAGVHSPMASSGNTGNHSFSSSSLSALQAISEGVGTSLLST
LSSPGPKLDNSPNMNITQPSKVSSQDSKSPLGFYCDQNPVESSMCQSNSRDHLSDKESKE
SSVEGAENQRGPLESKGHKKLLQLLTCSSDDRGHSSLTNSPLDSSCKESSVSVTSPSGVS
SSTSGGVSSTSNMHGSLLQEKHRILHKLLQNGNSPAEVAKITAEATGKDTSSITSCGDSN
VVKQEQLSPKKKENNALLRYLLDRDDPSDALSKELQPQVEGVDNKMSQCTSSTIPSSSQE
KDPKIKTETSEEGSGDLDNLDAILGDLNSSDFYNNSISSNSSHLGTKQQVFQGTNSLGLK
SPQTVQSIRPPYNRAVSLDSPVSVGSSLPVKNISAFPMLPKQPMLGGNPRMMDSQENYGS
SVGGPNRNVTGTQTPSSGDWGLPNSKAGRMEPMNSNSMGRPGGDYNTSLPRPALGGSIPT
LPLRSNSIPGARPILQPQQQQQQMLQMRPGEIPMGMGANPYGQAASSNQLGSWPDGMLSM
EQVPHGTQNRPLLRNSLDDLVGPPSNLEGQSDERALLDQLHTLLSNTDATGLEEIDRALG
IPELVNQGQALEPKQDAFQGQEAAVMMDQKAGLYGQTYPAQGPPMQGGFHLQGQSPSFNS
MMNQMNQQGNFPLQGMHPRANIMRPRTNTPKQLRMQLQQRLQGQQFLNQSRQALELKMEN
PTAGGAAVMRPMMQPQVSSQQGFLNAQMVAQRSRELLSHHFRQQRVAMMMQQQQQQQQQ
QQQQQQQQQQTQAFSPPPNVTASPSMDGLLAGPTMPQAPPQQFPYQPNYGMGQQPDPAFG
RVSSPPNAMMSSRMGPSQNPMMQHPQAASIYQSSEMKGWPSGNLARNSSFSQQQFAHQGN
PAAYSMVHVNGSSGHMGQMNMNPMPMSGMPMGPDQKYC
>205Gallus gallus|A0A3Q2UJ10|Aves|SRC3
MVRMSGLGESSLDSLTNESRKRKPLPCDTPGASLTCSGEKRRREQESKYIEELAELISAN
LSDIDNFNVKPDKCAILKETVRQIRQIKEQGKAVSNDDEVQKADVSSTGQGVIDKDSLGP
LLLQALDGFLFVVNRDGNIVFVSENVTQYLQYKQEDLVNTSVYGILHEEDRKDFLKNLPK
SSVNGVAWTNDAPRQKSHTFNCRMMVKTSHDLLEDVGSHQDTRQRYETMQCFALSQPRAM
MEEGEDLQSCMICVARRIATAERVFPTNPESFITRHDLSGKLVNIDTNSLRSSMRPGFED
TIRRCIQRFLCHNDGQSWSNKRHYHEAYTQGHAETPLYRFSLADGTIVTAQTKSKVFRNP
VTNDRHGFVSTHFLQREQNGYRPNPNTVGQNIRTTVAGNTNSVGLMNMSPGQGMPMQNRN
YGMGDPSTVGQLNSSRYGGPGNMGPVNSGPGMQSSAYQSNSYGLNISSPPHGSPGLTSNQ
QNLMISTRNRGSPKMNSHQFSPVTGMHSPMGSASNTSNSTFSSSSLSALQAISEGVGTSL
LSTLSSPGPKLDSSPNVSIAQQNKANNQDSKSPSGLYCEQNQVESSICQSNSRDVLSEKD
SKDGSLDASESQRGQSESKGHKKLLQLLTCSSDERGHSTASNSPLDSNCKESSTNVTSPS
GVSSSTSGGVSSSSNVHGSLLQEKHRILHKLLQNGNSPAEVAKITAEATGKDTYHDTSNT
VPCGESTVKQEQLSPKKKENNALLRYLLDKDDVKDPLSKELKPKVENVDNKMGQCSSSTI
PTSSQEKEMKIKTEPTEEMSGDLDNLDAILGDLTSSDFYNNAMSTSGNNFGMKQALFQGN
ASLTGMRSPQSVQAARPPFNRAMSLDSPVGSGASVRNVNAFSMLQKQNLMGGSPRMTENQ
ESFGANIASTPNRGVSMNQHQSGDWGLPNSKVNRLEPTSSTSMLRQGAEFSASLPRPTAG
GSMPGLPVRSNSIPGTRPMLQQQMMHMRSNEINIGMGGNPYGQTGPTSQPGSWPDSMLSM
EQASRGLQNRQLVRNSLDDLLSAAPSVEGQNDERALLDQLHTLLSNTDVTSLEEIDRALG
IPDLVNQGQTLEPKQDSFQGQESTVMMDQKPSLYGQPYQGQGTALPGGFNNIQGQQPSFN
SVMNQMSQQNNFPLQSMHPRANAMRPRTNTPKQLRMQLQQRLQGQQFMNQTRQALEMKME
NPGSSNSSVMRPVMQPQVGSQQQGFLNAQMVAQRNRELISHHFRQQRMAMMMQQQQPQAF
SPPPNVTASGSMDSGLTGPPMAQVPPQQFSYPPNYGISQQPDPAYSRVSSPPNPMMASRI
GPSQNPMMQHPQTAPMYQSPEMKGWPSGSMARSSSFPQQQFSHQGNPATYSMMHMNGSSG
HIGQMNITTVPMSGMPMGPDQVSCT
>206Gallus gallus|A0A3Q3B1M1|Aves|SRC3
MVRMSGLGESSLDSLTNESRKRKPLPCDTPGASLTCSGEKRRREQESKYIEELAELISAN

LSDIDNFNVKPDKCAILKETVRQIRQIKEQGKAVSNDDEVQKADVSSTGQGVIDKDSLGP
LLLQALDGFLFVVNRDGNIVFVSENVTQYLQYKQEDLVNTSVYGILHEEDRKDFLKNLPK
SSVNGVAWTNDAPRQKSHTFNCRMMVKTSHDLLEDVGSHQDTRQRYETMQCFALSQPRAM
MEEGEDLQSCMICVARRIATAERVFPTNPESFITRHDLSGKLVNIDTNSLRSSMRPGFED
TIRRCIQRFLCHNDGQSWSNKRHYHEAYTQGHAETPLYRFSLADGTIVTAQTKSKVFRNP
VTNDRHGFVSTHFLQREQNGYRPNPNTVGQNIRTTVAGNTNSVGLMNMSPGQGMPMQNRN
YGMGDPSTVGQLNSSRYGGPGNMGPVNSGPGMQSSAYQSNSYGLNISSPPHGSPGLTSNQ
QNLMISTRNRGSPKMNSHQFSPVTGMHSPMGSASNTSNSTFSSSSLSALQAISEGVGTSL
LSTLSSPGPKLDSSPNVSIAQQNKANNQDSKSPSGLYCEQNQVESSICQSNSRDVLSEKD
SKDGSLDASESQRGQSESKGHKKLLQLLTCSSDERGHSTASNSPLDSNCKESSTNVTSPS
GVSSSTSGGVSSSSNVHGSLLQEKHRILHKLLQNGNSPAEVAKITAEATGKDTYHDTSNT
VPCGESTVKQEQLSPKKKENNALLRYLLDKDDVKDPLSKELKPKVENVDNKMGQCSSSTI
PTSSQEKEMKIKTEPTEEMSGDLDNLDAILGDLTSSDFYNNAMSTSGNNFGMKQALFQGN
ASLTSTPNRGVSMNQHQSGDWGLPNSKVNRLEPTSSTSMLRQGAEFSASLPRPTAGGSMP
GLPVRSNSIPGTRPMLQQQMMHMRSNEINIGMGGNPYGQTGPTSQPGSWPDSMLSMEQAS
RGLQNRQLVRNSLDDLLSAAPSVEGQNDERALLDQLHTLLSNTDVTSLEEIDRALGIPDL
VNQGQTLEPKQDSFQGQESTVMMDQKPSLYGQPYQGQGTALPGGFNNIQGQQPSFNSVMN
QMSQQNNFPLQSMHPRANAMRPRTNTPKQLRMQLQQRLQGQQFMNQTRQALEMKMENPGS
SNSSVMRPVMQPQVGSQQQGFLNAQMVAQRNRELISHHFRQQRMAMMMQQQQPQAFSPPP
NVTASGSMDSGLTGPPMAQVPPQQFSYPPNYGISQQPDPAYSRVSSPPNPMMASRIGPSQ
NPMMQHPQTAPMYQSPEMKGWPSGSMARSSSFPQQQFSHQGNPATYSMMHMNGSSGHIGQ
MNITTVPMSGMPMGPDQVSCT

>207Homo sapiens|Q9Y6Q9|Mammalia|SRC3
MSGLGENLDPLASDSRKRKLPCDTPGQGLTCSGEKRRREQESKYIEELAELISANLSDIDNFNVKPDKCAILKE
TVRQIRQIKEQGKTISNDDDVQKADVSSTGQGVIDKDSLGPLLLQALDGFLFVVNRDGNIVFVSENVTQYLQYK
QEDLVNTSVYNILHEEDRKDFLKNLPKSTVNGVSWTNETQRQKSHTFNCRMLMKTPHDILEDINASPEMRQRYE
TMQCFALSQPRAMMEEGEDLQSCMICVARRITTGERTFPSNPESFITRHDLSGKVVNIDTNSLRSSMRPGFEDI
IRRCIQRFFSLNDGQSWSQKRHYQEAYLNGHAETPVYRFSLADGTIVTAQTKSKLFRNPVTNDRHGFVSTHFLQ
REQNGYRPNPNPVGQGIRPPMAGCNSSVGGMSMSPNQGLQMPSSRAYGLADPSTTGQMSGARYGGSSNIASLTP
GPGMQSPSSYQNNNYGLNMSSPPHGSPGLAPNQQNIMISPRNRGSPKIASHQFSPVAGVHSPMASSGNTGNHSF
SSSSLSALQAISEGVGTSLLSTLSSPGPKLDNSPNMNITQPSKVSNQDSKSPLGFYCDQNPVESSMCQSNSRDH
LSDKESKESSVEGAENQRGPLESKGHKKLLQLLTCSSDDRGHSSLTNSPLDSSCKESSVSVTSPSGVSSSTSGG
VSSTSNMHGSLLQEKHRILHKLLQNGNSPAEVAKITAEATGKDTSSITSCGDGNVVKQEQLSPKKKENNALLRY
LLDRDDPSDALSKELQPQVEGVDNKMSQCTSSTIPSSSQEKDPKIKTETSEEGSGDLDNLDAILGDLTSSDFYN
NSISSNGSHLGTKQQVFQGTNSLGLKSSQSVQSIRPPYNRAVSLDSPVSVGSSPPVKNISAFPMLPKQPMLGGN
PRMMDSQENYGSSMGGPNRNVTVTQTPSSGDWGLPNSKAGRMEPMNSNSMGRPGGDYNTSLPRPALGGSIPTLP
LRSNSIPGARPVLQQQQQMLQMRPGEIPMGMGANPYGQAAASNQLGSWPDGMLSMEQVSHGTQNRPLLRNSLDD
LVGPPSNLEGQSDERALLDQLHTLLSNTDATGLEEIDRALGIPELVNQGQALEPKQDAFQGQEAAVMMDQKAGL
YGQTYPAQGPPMQGGFHLQGQSPSFNSMMNQMNQQGNFPLQGMHPRANIMRPRTNTPKQLRMQLQQRLQGQQFL
NQSRQALELKMENPTAGGAAVMRPMMQPQVSSQQGFLNAQMVAQRSRELLSHHFRQQRVAMMMQQQQQQQQQQQ
QQQQQQQQQQQQQQQQQQTQAFSPPPNVTASPSMDGLLAGPTMPQAPPQQFPYQPNYGMGQQPDPAFGRVSSPP
NAMMSSRMGPSQNPMMQHPQAASIYQSSEMKGWPSGNLARNSSFSQQQFAHQGNPAVYSMVHMNGSSGHMGQMN
MNPMPMSGMPMGPDQKYC
>208Charadrius vociferus|A0A099ZVR3|Aves|SRC3
CMVKMSGLGENSLDSLTSESRKRKPLPCDTPGASLTCSGEKRRREQESKYIEELAELISANLSDIDNFNVKPDK
CAILKETVRQIRQIKEQGKAVSNDDDVQKADVSSTGQGVIDKDSLGPLLLQASALDGFLFVVNRDGNIVFVSEN
VTQYLQYKQEDLVNTSVYGILHEEDRKDFLKNLPKSSVNGVAWTNETPRQKSHTFNCRMMVKTSHDLLEDVGSH
QDTRQRYETMQCFALSQPRAMMEEGEDLQSCMICVARRITTAERVFPTNPESFITRHDLSGKLVNIDTNSLRSS
MRPGFEDIIRRCIQRFLCHNDGQSWSNKRHYHEAYTQGHAETPLYRFSLADGTIVTAQTKSKLFRNPVTNDRHG
FVSTHFLQREQNGYRPNPNSVGQNIRTPVAGNTNSVGGLMNMSPGQAMPMQNRNYSIGDPNAAGQLSSMRYGGP
GNMGPVNSGPGIQSSAYQNNSYGLNISSPPHGSPGLTSNQQNLMISPRNRGSPKLSSHQFSPVTGMHSPMGSAS
NTGNSTFSSSSLSALQAISEGVGTSLLSTLSSPGPKLDNSPNMSIGQQSKTSTQDSKSPSGLYCEQNQVESSIC
QSNSRDLLSEKDSKEGNVEASESQRGPIESKGHKKLLQLLTCSSDERGHSTASNSPLDSNCKESSTSVTSPSGV

SSSTSGGVSSSSNMHGSLLQEKHRILHKLLQNGNSPAEVAKITAEATGKDTYHDAGNTAPCGEGTVKQEQLSPK
KKENNALLRYLLDKDDVKDPLSKELKPKVESADNKMGQCSSSTIPTSSQEKEMKIKTEPAEEMSGDLDNLDAIL
GDLTSSEFYNNAMAANGNNLGTKQALFQGNALLGMRSPQSVPTTRPPFTRAMSLDSPVGSSAPVRNVNAFSTLQ
KQNLMGGSPRMIENQDNFGANLASAPNRNVAMNQHQSGDWGLPNSKVNRLEPTSSASMLRPGPEFSVSLPRPTV
GGSVSGLPVRSNSIPGTRPMLQPQMIHMRPNEMNIGMGGNPYGQPGPSNQPGSWPDNMLSVDQASRASQNRQLV
RNSLDDLLCPAPNMEGQNDERALLDQLHTLLSNTDVTGLEEIDRALGIPDLVNQGQALEPKQDSFQGQESSVMI
DQKPSLYGQPYQGQGAAMPGSFSNIQGQQPTFNSVMNQMSQQSNFPLQSMHPRANVMRPRTNTPKQLRMQLQQR
LQGQQFMNQTRQALEMKMENPVSGSTSVMRPVMQPQVGSQQGFLNAQMVAQRNRELISHHFRQQRMAMMMQQQQ
QPQAFSPPPNVTASGSMDSALTGPPMAQVAPQQFPYPPNYGISQQPDPAFSRVSSPPNPMMSSRIGPSQNPMMQ
HPQTAAMYQSPEMKGWPSGSMARSSSFPQQQFSHQGNPAAYSMMHMNGSSGHIGQMNINTMPMSGMPMGPDQVS
>209Xenopus laevis|A0A1L8ELC8|Amphibia|SRC3
MSGLGENSLDPLASETRKRKPSSCDTPGPGLTCSGEKRRREQESKYIEELADLISANLSDIDNFNVKPDKCAIL
KETVRQIRQIKEQGKASSNDDDVQKADVSSTGQGVIDKDSLGPLLLQALDGFLFVVNREGSIVFVSENVTQYLQ
YKQEDLVNTSVYSILHEEDRKDFLKNLPKSTVNGVPWSSETPRQKSHTFNCRMLVKTSHDHLEDGSNLDARQRY
ETMQCFALSQPRAMIEEGEDLQSCMICVARRITTAERAFSANPESFITRHDLTGKVVNIDANSLRSSMRPGFED
TIRRCIQRFLCHSEGQPWTYKRHYQEAYVHGLSETPLYRFSLADGTMVTAQTKSKLFRNPVTNDPHGFVSTHFL
QREQNGYRPNPNPMAQGIRPQMNPNPPNTMNSMPPQAMQQQNRNYGMGDPNSMAQMQGMRYKSPGNMAPVNQAP
GVQQSPYQNNSNYGLNMNSPPHGSPGMNANQPNLMVSPRNRASPKMASNQFSPVPGMNSPMGSSGNAGGGSFSS
SSLSALHAISEGVGSSLLSSLSSPGQKVENNSNMNMPQQGKICNQDCKSPSGLYCEQGQVESSVCQSSGREHLG
EKDVKENIFEGSESQRSQAESKGHKKLLQLLTCSTEERGQSLMSSSSMDCKDSSNVTSPSGVSSSTSIGVSSTS
NLHGSMLQEKHRILHKLLQNGNSPAEVAKITAEATGKDVFQETVSSAPCTEATVKREQLSPKKKENNALLRHLL
DKDDWKDPLAKDIKPKVEHMDIKMGSCSSSNVPTSSQDKEVKIKTEPGEEVPGDLDNLDAILGDLAGSDFYSNS
MSSRASDLGPKQPVFQDSPTLAMRSPDSMQGSRPPFNRAMSLDSRSSTPPVRNVNSFPMLPKQGMIGSPRMMDG
QDNFGVMMGSGPNRSMNQHPGGDWAMQNSAVNRLEPPNVGSVGRPGPDYSSAMTRPAMGGNMPGLPTRSNSIPG
SRPVMQQQQHILPMRPNDMAMSMGSNPYGQQAPSNPPGSWPDAIMMNQGRGGAQNRQLGRNSLDDLLCPPSTVE
GQTDEIALLDQLHTLLSNTDATGLEEIDRALGIPDLVSQGQALEPQPDSYQPQGSPVMIDQKPPMYGQHYAGQG
AAMSAGGFNNMQGQHPPFNTVMGQMNQQQGMHPLQGMHPRANLIRPRNNIPKQLRMQLQQRLQGQQFLNQNRQA
LEMKVDPMNPGGAGVMRPVMQTPVSQQGFLNAQMVAQRNRELISHQIRQHRMAMMMQQQQGQPQAFSPPPNVTA
SASMDNPLGGPPMPQAPPQQFSYPPNYGINQQTDPTFGRVSSPPNAMMSSRMAPSQNPHPQTTQMYPSPDMKGW
PSGNMARPNSFPQQQYSHQTNPATYNMMHMNGNGNHMGQMNMNSLPMSGMPMGPDQKYC
>210Alligator sinensis|A0A1U8DV95|Sarcopterygii|SRC3
MSGLGENSLDSLTNESRKRKSLPCDTPGPGLNCSGEKRRREQESKYIEELAELISANLSDIDNFNVKPDKCAIL
KETVRQIRQIKEQGKTASSDDDVQKADVSSTGQGVIDKDSLGPLLLQALDGFLFVVNRDGNIVFVSENVTQYLQ
YKQEDLVNTSVYSILHEEDRKDFLKNLPKAAVNGVAWTNETPRQKSHTFNCRMMVKTCHDLLEDVGSNQDTRQR
YETMQCFALSQPRAMMEEGEDLQSCMICVARRITTVERVFPANPESFITRHDLSGKLINIDTNSLRSSMRPGFE
DIIRRCIQRFLCHNDGQTWSNKRHYQEAYIQGHAETPLYRFSLADGTIVTAQTKSKLFRNPVTNDRHGFVSTHL
LQREQNGYRPNPNPVGQSIRAPTAGSTNSVGAIMNMPPGQSMPIQNRNYGMGDPSAVGQMSSTRYGGPGNMGPM
NSGPGMQSSPFQNNNYGLNMSSPPHGSPGLTSSQQNLMISPRHRGSPKMGSHQFSPVAGMHSPMGSATNTSNNS
FSSSSLSALQAISEGVGTSLLSTLSSPGPKLDNSPNMNIAQQSKMGNQDSKSPVLYCEQSQVESSMCQSNSRDL
LTDKDNKEGSLEGSESQRGPSESKGHKKLLQLLTCSSEERGHATLSNSPLDSNCKESSTSVTSPSGVSSSTSGG
VSSSSNMHGSLLQEKHRILHKLLQNGNSPAEVAKITAEATGKDTYHESSNTTPCGEGTIKQEQLSPKKKENNAL
LRYLLDKDDIKDPLSKDIKPKVESLDNKMGQCSSSSISTSNQEKEIKVKIEPSEEMSGDLDNLDAILGDLTSSD
FYSNANSANGSNLGTKQAIYQGNAPLGMRSPQSMQATRPPFNRAMSLDSPIPMGSSPPVRSVNAFSMLQKQNMM
GGNSRMIESQENFGANIGAPNRSMTMNQHPAGDWGLPNSKVNRLEPTSSSSMMRPGADYNTSLPRPTIGSSVPG
LPIRSNSIPGTRPILQQQMIHMRPNEINMGMGGNPYAQPGPSSQPASWPDSMLAIEQGSRGSQSRQLVRSSLDD
LLCPPPNVEGQNDERALLDQLHTLLSNTDVTGLEEIDRALGIPDLVNQGQTLEPKQDTFQGQESAVMIDQKPPL
YGQPYQGQGTAVPGGFSNMQGQQPSFNSMMNQISHQSNFPLPNVHPRASVMRPRTNTPKQLRMQLQQRLQGQQF
MNQTRQALEMKMENPGATVAPVMRPVMQPQVGSQQQGFLNAQMMAQRNRELLSHHIRQQRMAMIMQQQQQQQQP
QAFSPPPNVTASGSMDSALAGPPMAQVPPQQFPYPPNYGINQQPDPAFSRVSSPPNPMMSSRIGPSQNPMMQHP
QTAPMYQSPEMKGWPSGTMARSSSFSQQQFSHQGTPATYNMMHMNGTGGHIGQMNLNTMPMSGMPMGPDQKYC
>211Patagioenas fasciata monilis|A0A1V4KL73|Aves|SRC3
MVKMSGLGENSLDSLSSESRKRKPLPCDTPGASLSCSGEKRRREQESKYIEELAELISANLSDIDNFNVKPDKC
AILKETVKQIRQIKEQGKAVSNDDDVQKADVSSTGQGVIDKDSLGPLLLQALDGFLFVVNRDGNIVFVSENVTQ
YLQYKQEDLVNTSVYGILHEEDRKDFLKNLPKSSVNGVAWTNETPRQKSHTFNCRMMVKTSHDLLEDVGGLPDT

RQRYETMQCFALSQPRAMMEEGEDLQSCMICVARRITTAERAFPANPESFITRHDLSGKLVNIDTNSLRSSMRP
GFEDIIRRCIQRFLCHNDGQSWSNKRHYHEAYTQGHAETPLYRFSLADGTIVTAQTKSKLFRNPVTNDRHGFVS
THFLQREQNGYRPNPNSVGQNMRAAVAGNASSAGGVMAASPGHGLAAQSRSYGLAEPGAVGQLSSTRYGGPGSV
GPANSGPGMQPSAYQNSSYGLSMGSPPHGSPGLTSNQQNLMISPRNRGSPKMSSHQFSPVTGMHSPMGSASNTG
NNTFSSSSLSALQAISEGVGTSLLSTLSSPGPKLDNSPNMSVSQQSKTSTQDSKSPSGLYCEQNQVESSICQSN
SRDLLSEKDSKEGNVEASESQRAPSESKGHKKLLQLLTCSSDDRGHSTASNSPLDSSCKESSTSVTSPSGVSSS
TSGGVSSSSNVHGSLLQEKHRILHKLLQNGNSPAEVAKITAEATGKDTYHDVSNAAPCGEGTVKQEQLSPKKKE
NNALLRYLLDKDDVKDPLSKELKPKVENVDNKMGQCSSSTIPTSSQEKETKIKPEPPEEMSGDLDNLDAILGDL
TSSEFYNSAMSANGNNLGTKQALFQGNALMGTRSPQSAQAARAPFARAASLDSPGGPGRSVSAFSILQKQNLMG
GSPRMIENQENFGANMGGAPSRSVAMAQHPAGDWGLPSSKGSRAEPAAAAVGRAGPEFSASLPRPAAGGSVPGL
PARSSSVPGPRPVLQQQMIHMRPSEMNIGMGGNPYGQPGPSHQPGSWPDSVLPMEQASRGSQNRQLVRNSLDDL
LCPAPSMEGQNDERALLDQLHTLLSNTDVTSLEEIDRALGIPELVNQGQALEPKQDSFQGQESSVLIEQKPSLY
GQPYQGQGAALPGGFSNVQGQQPSFNSVMNQMSQQSNFPIQSMHPRANVMRPRTNAPKQLRMQLQQRLQGQQFM
NQTRQALEMKMENPVGGNSSMMRAVMQPQVGSQQGFLNAQMVAQRNRELISHHFRQQRMAMMMQQQQQPQAFSP
PPNVTASGSMDSALAAPPMAQIPPQQFPYPPNYGISQQPDPAYSRVPSPPNPMMASRMGPSQSPMMQHPQAAPM
YQSPEMKGWPSGSMARSSFPQQQFSHQGSPAAYSMMHVNGSGGHMGQMNISAMPMSGMPLGPDQQKYC
>212Patagioenas fasciata monilis|A0A1V4KL74|Aves|SRC3
MVKMSGLGENSLDSLSSESRKRKPLPCDTPGASLSCSGEKRRREQESKYIEELAELISANLSDIDNFNVKPDKC
AILKETVKQIRQIKEQGKAVSNDDDVQKADVSSTGQGVIDKDSLGPLLLQALDGFLFVVNRDGNIVFVSENVTQ
YLQYKQEDLVNTSVYGILHEEDRKDFLKNLPKSSVNGVAWTNETPRQKSHTFNCRMMVKTSHDLLEDVGGLPDT
RQRYETMQCFALSQPRAMMEEGEDLQSCMICVARRITTAERAFPANPESFITRHDLSGKLVNIDTNSLRSSMRP
GFEDIIRRCIQRFLCHNDGQSWSNKRHYHEAYTQGHAETPLYRFSLADGTIVTAQTKSKLFRNPVTNDRHGFVS
THFLQREQNGYRPNPNSVGQNMRAAVAGNASSAGGVMAASPGHGLAAQSRSYGLAEPGAVGQLSSTRYGGPGSV
GPANSGPGMQPSAYQNSSYGLSMGSPPHGSPGLTSNQQNLMISPRNRGSPKMSSHQFSPVTGMHSPMGSASNTG
NNTFSSSSLSALQAISEGVGTSLLSTLSSPGPKLDNSPNMSVSQQSKTSTQDSKSPSGLYCEQNQVESSICQSN
SRDLLSEKDSKEGNVEASESQRAPSESKGHKKLLQLLTCSSDDRGHSTASNSPLDSSCKESSTSVTSPSGVSSS
TSGGVSSSSNVHGSLLQEKHRILHKLLQNGNSPAEVAKITAEATGKDTYHDVSNAAPCGEGTVKQEQLSPKKKE
NNALLRYLLDKDDVKDPLSKELKPKVENVDNKMGQCSSSTIPTSSQEKETKIKPEPPEEMSGDLDNLDAILGDL
TSSEFYNSAMSANGNNLGTKQALFQGNALMGTRSPQSAQAARAPFARAASLDSPGGPGRSVSAFSILQKQNLMG
GSPRMIENQENFGANMGGAPSRSVAMAQHPAGDWGLPSSKGSRAEPAAAAVGRAGPEFSASLPRPAAGGSVPGL
PARSSSVPGPRPVLQQQMIHMRPSEMNIGMGGNPYGQPGPSHQPGSWPDSVLPMEQASRGSQNRQLVRNSLDDL
LCPAPSMEGQNDERALLDQLHTLLSNTDVTSLEEIDRALGIPELVNQGQALEPKQDSFQGQESSVLIEQKPSLY
GQPYQGQGAALPGGFSNVQGQQPSFNSVMNQMSQQSNFPIQSMHPRANVMRPRTNAPKQLRMQLQQRLQGQQFM
NQTRQALEMKMENPVGGNSSMMRAVMQPQVGSQQQGFLNAQMVAQRNRELISHHFRQQRMAMMMQQQQQPQAFS
PPPNVTASGSMDSALAAPPMAQIPPQQFPYPPNYGISQQPDPAYSRVPSPPNPMMASRMGPSQSPMMQHPQAAP
MYQSPEMKGWPSGSMARSSFPQQQFSHQGSPAAYSMMHVNGSGGHMGQMNISAMPMSGMPLGPDQQKYC
>213Patagioenas fasciata monilis|A0A1V4KLA5|Aves|SRC3
MVKMSGLGENSLDSLSSESRKRKPLPCDTPGASLSCSGEKRRREQESKYIEELAELISANLSDIDNFNVKPDKC
AILKETVKQIRQIKEQGKAVSNDDDVQKADVSSTGQGVIDKDSLGPLLLQALDGFLFVVNRDGNIVFVSENVTQ
YLQYKQEDLVNTSVYGILHEEDRKDFLKNLPKSSVNGVAWTNETPRQKSHTFNCRMMVKTSHDLLEDVGGLPDT
RQRYETMQCFALSQPRAMMEEGEDLQSCMICVARRITTAERAFPANPESFITRHDLSGKLVNIDTNSLRSSMRP
GFEDIIRRCIQRFLCHNDGQSWSNKRHYHEAYTQGHAETPLYRFSLADGTIVTAQTKSKLFRNPVTNDRHGFVS
THFLQREQNGYRPNPNSVGQNMRAAVAGNASSAGGVMAASPGHGLAAQSRSYGLAEPGAVGQLSSTRYGGPGSV
GPANSGPGMQPSAYQNSSYGLSMGSPPHGSPGLTSNQQNLMISPRNRGSPKMSSHQFSPVTGMHSPMGSASNTG
NNTFSSSSLSALQAISEGVGTSLLSTLSSPGPKLDNSPNMSVSQQSKTSTQDSKSPSGLYCEQNQVESSICQSN
SRDLLSEKDSKEGNVEASESQRAPSESKGHKKLLQLLTCSSDDRGHSTASNSPLDSSCKESSTSVTSPSGVSSS
TSGGVSSSSNVHGSLLQEKHRILHKLLQNGNSPAEVAKITAEATGKDTYHDVSNAAPCGEGTVKQEQLSPKKKE
NNALLRYLLDKDDVKDPLSKELKPKVENVDNKMGQCSSSTIPTSSQEKETKIKPEPPEEMSGDLDNLDAILGDL
TSSEFYNSAMSANGNNLGTKQALFQGNALMGTRSPQSAQAARAPFARAASLDSPGGPGRSVSAFSILQKQNLMG
GSPRMIENQENFGANMGGAPSRSVAMAQHPAGDWGLPSSKGSRAEPAAAAVGRAGPEFSASLPRPAAGGSVPGL
PARSSSVPGPRPVLQQQMIHMRPSEMNIGMGGNPYGQPGPSHQPGSWPDSVLPMEQASRGSQNRQLVRNSLDDL
LCPAPSMEGQNDERALLDQLHTLLSNTDVTSLEEIDRALGIPELVNQGQALEPKQDSFQGQESSVLIEQKPSLY
GQPYQGQGAALPGGFSNVQGQQPSFNSVMNQMSQQSNFPIQSMHPRANVMRPRTNAPKQLRMQLQQRLQGQQFM
NQTRQALEMKMENPVGGNSSMMRAVMQPQVGSQQGFLNAQMVAQRNRELISHHFRQQRMAMMMQQQQQPQAFSP

PPNVTASGSMDSALAAPPMAQIPPQQFPYPPNYGISQQPDPAYSRVPSPPNPMMASRMGPSQSPMMQHPQAAPM
YQSPEMKGWPSGSMARSSFPQQQFSHQGSPAAYSMMHVNGSGGHMGQMNISAMPMSGMPLGPDQKYC
>214Columba livia|A0A2I0M248|Aves|SRC3
MVKMSGLGENSLDSLSSESRKRKPLPCDTPGASLTCGGEKRRREQESKYIEELAELISANLSDIDNFNVKPDKC
AILKETVKQIRQIKEQGKAVSNDDDVQKADVSSTGQGVIDKDSLGPLLLQALDGFLFVVNRDGNIVFVSENVTQ
YLQYKQEDLVNTSVYGILHEEDRKDFLKNLPKSSVNGVAWTNETPRQKSHTFNCRMMVKTSHDLLEDVGSLPDT
RQRYETMQCFALSQPRAMMEEGEDLQSCMICVARRITTAERAFPTNPESFITRHDLSGKLVNIDTNSLRSSMRP
GFEDIIRRCIQRFLCHNDGQSWSNKRHYHEAYTQGHAETPLYRFSLADGTIVTAQTKSKLFRNPVTNDRHGFVS
THFLQREQNGYRPNPNSVGQNVRTAVAGNASSAGALVTASPGHGLAAQSRSYGLADPGAVGQLSSTRYGGPGSV
GPASSGPGMQPSAYQNNSYGLTMSSPPHGSPGLTSNQQNLMISPRNRGSPKMSSHQFSPVTGMHSPMGSASNTG
NNTFSSSSLSALQAISEGVGTSLLSTLSSPGPKLDNSPNMSVSQQSKTSTQESKSPSGLYCEQNQVESSICQSN
SRDLLSEKDSKEGNVEASESQRAPSESKGHKKLLQLLTCSSDDRGHSTASNSPLDSSCKESSTSVTSPSGVSSS
TSGGVSSSSNVHGSLLQEKHRILHKLLQNGNSPAEVAKITAEATGKDTYHDVSNTAPCGEGTVKQEQLSPKKKE
NNALLRYLLDKDDVKDPLSKELKPKVENVDNKMGQCSSSTVPTSSQEKETKIKPEPPEEMSGDLDNLDAILGDL
TSSEFYNNAMSANGNNLGTKQALFQGNALLGTRSPQSAQAARAPFARAVSLDSPGAAVRNVSAFSMLHKQNLMG
GSPRMIENQENFGANLGGAPSRSVAVGQHPAADWALPSSKGSRAGPAAASVGRPGPEFSASLPRPAAGGSVPGL
PVRSSSIPGARPVLQQQMIHMRPNEMNIGMGGNPYGQAGPSHQPSSWPDNVLSMEPASRGAQNRQLVRNSLDDL
LCPAPSMEGQNDERALLDQLHTLLSNTDVTSLEEIDRALGIPELVNQGQALEPKQDSFQGQESSVMIEQKPSLY
GQPYQGQGAAMPGGFSSIQGQQPSFNSVMNQMSQQSSFPVQSMHPRASVMRPRTNAPKQLRMQLQQRLQGQQFM
NQTRQALEMKMENPGGGNTSVMRPVMQPQVGSQQQGFLNAQMVAQRNRELISHHFRQQRMAMMMQQQQQPQAFS
PPPNVTASGSMDSALAGPPMAQVPAQQFPYPPNYGLSQQPDPAYSRVPSPPNPMITSRMGPAQNPMMQHPQAAP
MYPSPEMKGWPSGSMARSSFPQQQFSHQGNPAAYSMMHMNGSGGHMGQMNINTMPMSGMPLGPDQVSCT
>215Cebus imitator|A0A2K5PKD5|Mammalia|SRC3
MSGLGENLDPLATDSRKRKLPCDTPGQGLICSGEKRRREQESKYIEELAELISANLSDIDNFNVKPDKCAILKE
TVRQIRQIKEQGKTISSDDDVQKADVSSTGQGVIDKDSLGPLLLQALDGFLFVVNRDGNIVFVSENVTQYLQYK
QEDLVNTSVYNILHEEDRKDFLKNLPKSTVNGVSWTNESQRQKSHTFNCRMLMKTHDILEDINTSPEMRQRYET
MQCFALSQPRAMMEEGEDLQSCMICVARRITTGERVYPSNPESFITRHDLSGKVVNIDTNSLRSSMRPGFEDII
RRCIQRFFSLNDGQSWSQKRHYQEAYLNGHAETPVYRFSLADGTIVTAQTKSKLFRNPVTNDRHGFVSTHFLQR
EQNGYRPNPNPVGQGIRPPMAGCNSSVGGMSMSPNQGLQMPSSRAYGLADPSTTGQMSGARYGGSSNIASVTSG
PGMQSPSSYQNNNYGLNMSSPPHGSPGLAPNQQNIMISPRNRGSPKMASHQFSPVAGVHSPMGSSGSTGSHSFS
SSSLSALQAISEGVGTSLLSTLSSPGPKLDNSPNMNITQPSKVSSQDSKSPLGFYCDQNPVESSMCQSNSRDHL
SDKESKESSIEGSENQRGPLESKGHKKLLQLLTCSSDDRGHSSLTNSPLDSSCKESSVSVTSPSGVSSSTPGGV
SSTSSMHGSLLQEKHRILHKLLQNGNSPAEVAKITAEATGKDTSSITSCGDGNVVKQEQLSPKKKENNALLRYL
LDRDDPSDALSKELQPQVEGVDNKMSQCTSSTIPSSSQEKDPKIKTETSEEGSGDLDNLDAILGDLTSSDFYNN
SVSTNGSHLGTKQQIFQGTNSLGLKSPQSVQPIRPPYNRAVSLDSPVSVGSSPPVKNISAFPMLPKQPMLGGNP
RMMDSQENYGSSMGGPNRNVTVTQTPSSGDWGLPNSKASRMEPMNSNSMGRPGGDYNTSLPRAALGGSVPTLPL
RSNSIPGARPMLQPQQQQQQMLQMRPGEIPMGMGANPYGQAASNQLGSWSDGMLSMEQVPHGTQNRPLLRNSLD
DLVGPPSNLEGQSDERALLDQLHTLLSNTDATGLEEIDRALGIPELVNQGQALEPKQDAFQGQEAAVMMDQKAG
LYGQTYPAQGPPMQGGFHLQGQSPSFNSMMNQMNQQGSFPLQGMHPRANIMRPRTNTPKQLRMQLQQRLQGQQF
LNQSRQALELKMENPTAGGAAVMRPMMQPQVSSQQGFLNAQMVAQRSRELLSHHFRQQRVAMMMQQQQQQQQQQ
QQQQQQQQQQQTQAFSPPPNVTASPSMDGLLAGPAMPQAPPQQFTYQPNYGMGQPPDPAFGRVSSPPNAMMSSR
MGPSQNPMMQHPQAASMFQSSEMKGWPSGNLARNSSFSQQQFAHQGNPAVYSMVHVNGSSGHMGPMNMNPMPMS
GMPMGPDQKYC
>216Rhinopithecus roxellana|A0A2K6RNV1|Sarcopterygii|SRC3
MSGLGENLDPLATDSRKRKLPCDAPGQGLTCSGEKRRREQESKYIEELAELISANLSDIDNFNVKPDKCAILKE
TVRQIRQIKEQGKTISNDDDVQKADVSSTGQGVIDKDSLGPLLLQALDGFLFVVNRDGNIVFVSENVTQYLQYK
QEDLVNTSVYSILHEEDRKDFLKNLPKSTVNGVSWTNETQRQKSHTFNCRMLMKTPHDILEDINASPEMRQRYE
TMQCFALSQPRAMMEEGEDLQSCMICVARRITTGERAFPSNPESFITRHDLSGKVVNIDTNSLRSSMRPGFEDI
IRRCIQRFFSLNDGQSWSQKRHYQEAYLNGHAETPVYRFSLADGTIVTAQTKSKLFRNPVTNDRHGFVSTHFLQ
REQNGYRPNPNPVGQGIRPPMAGCNSSVGSMSMSPNQGLQMPSSRAYGLADPSTTGQMSGARYGGSSNIASLTP
GPGMQSPSSYQNNNYGLSMSSPPHGSPGLAPNQQNIMISPRNRGSPKIASHQFSPVAGVHSPMGSSGNSGNHSF
SSSSLSALQAISEGVGTSLLSTLSSPGPKLDNSPNMNITQPSKGSSQDSKSPLGFYCDQNPVESSMCQSNSRDH
LSDKESKESSVEGSENQRGPLESKGHKKLLQLLTCSSDDRGHSSLTNSPLDSSCKESSVSVTSPSGVSSSTSGG
VSSTSNMHGSLLQEKHRILHKLLQNGNSPAEVAKITAEATGKDTSSITSCGDGNVVKQEQLSPKKKENNALLRY

LLDRDDPSDALSKELQPQVEGVDNKISQCASSTLPSSSQEKDPKIKTETSEEGSGDLDNLDAILGDLTSSDFYN
NSVSSNGSHLGTKQQVFQGTNSLGLKSPQSVQSIRPPYNRAVSLDSPVSVGSSPPVKNISAFPMLPKQPMLGGN
PRMMMDSQENYGSSMGGPNRNVTVTQTPSSGDWGLPNSKASRMEPMNSNSMGRPGGDYNTSLPRPALGGSMPTL
PLRSNSIPGARPILQPQQQQMLQMRPGEIPMGMGANPYGQAAASNQLGSWPDGMLSIEQVPHGTQNRPLLRNS
LDDLVGPPSNLEGQSDERALLDQLHTLLSNTDATGLEEIDRALGIPELVNQGQALEPKQDAFQGQEAAVMMDQK
AGLYGQTYPAQGPPMQGGFHLQGQSPSFNSMMNQMNQQGNFPLQGMHPRANIMRPRTNTPKQLRMQLQQRLQGQ
QFLNQSRQALELKMENPTAGGAAVMRPMMQPQGFLNAQMVAQRSRELLSHHFRQQRVAMMMQQQQQQQQQQQQQ
QQQQQQQQQQQQTQAFSPPPNVTASPSMDGLLAGPAMPQAPPQQFPYQPNYGMGQQPDPAFGRVSSPPSAMISS
RMGPSQNPMMQHPQAASIYQSSEMKGWPSGNLARNSSFSQQQFAHQGNPAVYSMVHMNGSSGHMGQMNMNPMPM
SGMPMGPDQKYC
>217Esox lucius|A0A3P8YJ22|Actinopteri|SRC3
MSGLGDNSMEPLSSENRKRKLSTCDTPGLGCERRRREQESKYIEELAELISANLSDIDSFNVKPDKCAILKETV
RQIRQIKEQGKASSNDDDVQKSDVSSTGQGVIDKDHLGPLLLQALDGFLFVVNREGSVVFVSDNVTQYLQYKQE
ELINTSVYNILHEDDREEFHKNLPKTNVNGVSWGGEAPRQKSHTFTCRMLVKFGHAHGPIEEGPGGPRYETMQC
FALTQPKAMIEEGEDLQSCMICVARRVTAVERTESFNTRHDLSGETNSNTTLIYILLYQNSLRASMRPGWEELL
RRCIHMFLQQNDGQPWSHKRHYHEAFLHGHAETPLYRFSLSDGTPVTAQTKSKLYRHPMSNEPQGFISTHLLQR
EQNGYRSTQGGGMMPGQMRQPGIGGPNPNTQMNMPPGGGGMGMGIMNRGYGPMNEQNHMGQMGSGSMYGGNGGM
GNRMMQMNPMNQMNQMGQVNQMNQMGHQMNHPGPGNHPGMQHSQQPPFQSSGGYGLGGMNSPMGSPRLSGPQQG
LLMSPRNRCSPKMGASQFSPGGMHSPMGPHGGSGGGGTSFSSSSLNALQAISEGVGSSLPSTLTSPSPAHKPDS
SPSIHSSSQSSQPMQPAKPESSGDRLDNQSAAGGKEPREGASEAGVASSEPARRVPDSKGHKKLLQLLTSPTEE
LGLGGSGPAVPSIQPPPTNPPAGSNTKDPAGGMTSPSSTGVSSSSSASGSQSQSSGPGGAALANALASAHYTAS
LQEKHKILHKLLQNSNTPDEVAKITAEATGKVTLGGQEIPGETPGGVPAAGGVTETKQEQHSPKKEKTHALLHY
LLNKDTKEPVDVKPKPKELEGKGPPGASAGPPPRGPGPGHTPEHPESKIKSEPPDELENLESILGGLRGSGSEF
YPEQGDGGANEGGNKAPGGLDDNLQGRNIKHLMWVFCVPSGPMNRGMGVPQRSPMGGSGDWGMPRSSTSPVGSA
GHPSMMRPGMDYNSKSMMGGGPMVARSNSVPGTRSMLQQLMDMGSGEMGMGMSPFNQQGQPNQSPSWPDSVMG
MDSNHSRRQFGNALDELLAPPTTSEGQSDERALLDQLDSLLNNTDGIALEEIDRALGIPDLVSQGQGPEQHAEP
FPGQDPSMVLEQKPLYGQGYPGPPSMAMQSAYGGGPMQGQPQQGGFGPMLSQMGQGGSFPGMGGMVHPRSNLMR
PRMISATKPMRMQLQQRLQGQQFLSQTRQGMAMKMENPGPGNPGLTAGMNPGMRPGMQPGMGGQPSFLNSQMMA
QRSREMVTMQMRRQRMMMMMQQQQAAAAGGFSPPPNVTAPAGMDAPMGGPNMGQPGPQPFGYGGNYGMGQQGDP
SFGPAGGSPPNTMMSGRLGPQNPMMQQHPQGGPMYQSAEMKGWSQGGMGRNSSYPQQQFAQQGNPGQYGGMMMN
GGMPSNGGGAHMGQMGGQMGMNPMVIGRMPMGPDQKYC
>218Esox lucius|A0A3P8ZJS9|Actinopteri|SRC3
MSGVGDNSLEPMCSERKRKISTCDTPGLGCDKRRREQESKYIEELAELISANLGDIDSFNVKPDKCAILKETVR
QIRQIKEQGKSSCSDDDVQKADVSSTGQGVIDKDHLGPLLLQALDGFLFVVNREGSIVFVSDNVTQYLQYTQEE
LINTSVYNILHEDDREELHKNLPKNTAPNGGPWGGEASRQKSNTFNCRMLVKYGHGQSHQNHRPSEEGPNRQRY
ETMQCFALTQPRAMMEEGEDLQSCMICVARCITAVERTERFSTRHELSGKLIEIEQQSSLHTTMRPGWEDLVRR
CMQMFLYLSEGQPWFYKRHYKEALMHGRAETPSYRFSLSDGTLVTAQTRSELCRNQGSNEQHTFLSTHLLQREP
NGYRANQGGMMRPQGLGGTNAPMNMAPGGGMAGMNRSYGMGVEPGHMAQRGGHPYTGGNRMHAVNQMNAVNQMN
TVNQMNPSMHQMNSQMNSMNPVNQMSSMNQMNQMNQRSQMNHPGMHPHNPHPQQQQSAPFHGTSYGVGGVSGPS
QGGPGMNGPQNIMGSLRVRGSPKMAVGPFSPGGMHSPMTSGHPGSTGGTTSFSSSSLNALQAISEGVGSSIPSA
LPSALPSALTSPPTHKPDSSPGINSSQQGQSKPGGSDSKSPGSSLANGGEHPHTPTGEGIPDRPDSQASREGPA
RGEASRRDNKGGHKKLLQLLTSPTEELGPPNPQAGPGHAPMGSEAKEGAGGTTSPSSTGVSSSTAAAAHLANQS
LQEKHKILHKLLQNGNTPDEVARITAEATGKVTDGGVPEAGQGGPGARGAEVKQEQHSPKKEKAHALLHYLLNK
DDSKAGRGDPKPKPEDLDSRGTQGVCSGITISEPGNMVGKVKTEPPDELETLETILGGRRNSSSGFYTELDSRA
GKEIGNKPGNGPDSLQDGERGPMLPAQRGPLQRALSVDAKPLGGGGGLAGRRNVTCPTLIKQEKVEAPVRPGGM
NRAMGMPQRPPMAGQADWGMPRSTGSPVGVQGHPTLSRPGMDFNSKGMMGGPMIGRSNSVPGNTRSMLSQHLEM
GSGEVNMGMSPFSGQGPPPQSPSWPDSAMGMDRPPTNPNRRQFGNPLDELLVPPSTSQGQSDELALLDQLDCLL
NNTDVIALEEIDRALGIPDLPPPGPGQRPPSEPFPGPGPDTPMGMEPKPMYGHGYPGPPSMQSNYGGNPMQAQS
PAGFNSVMNQMGGQTAPGGFPGMGAMGHPRAGLMRPRLMGPANPLRQQLQQSASPGGNPGMRPGIQPSMGSQPG
YLNSQMMAQRSREMMTLQIRRQRMMMMMQQQTAAGGFSPPPNVTAPTGMDNPMTGPPMPGPPQFPYAGNYGMSQ
QGEPSFVGAGSSPPSNMMPGRMGGPQNAMMHPQSAPMYQSADMKGWPQSGMTRNSSYPQQQFTQQGNTGQFGGP
MMMNGSMGGPSPVSGAVGAQMGQMQGQLAMNSMGMGRMPMGPEQVRRLSLNKNAYK
>219Alligator sinensis|A0A3Q0FJH7|Sarcopterygii|SRC3

MSGLGENSLDSLTNESRKRKSLPCDTPGPGLNCSGEKRRREQESKYIEELAELISANLSDIDNFNVKPDKCAIL
KETVRQIRQIKEQGKTASSDDDVQKADVSSTGQGVIDKDSLGPLLLQALDGFLFVVNRDGNIVFVSENVTQYLQ
YKQEDLVNTSVYSILHEEDRKDFLKNLPKAAVNGVAWTNETPRQKSHTFNCRMMVKTCHDLLEDVGSNQDTRQR
YETMQCFALSQPRAMMEEGEDLQSCMICVARRITTVERVFPANPESFITRHDLSGKLINIDTNSLRSSMRPGFE
DIIRRCIQRFLCHNDGQTWSNKRHYQEAYIQGHAETPLYRFSLADGTIVTAQTKSKLFRNPVTNDRHGFVSTHL
LQREQNGYRPNPNPVGQSIRAPTAGSTNSVGAIMNMPPGQSMPIQNRNYGMGDPSAVGQMSSTRYGGPGNMGPM
NSGPGMQSSPFQNNNYGLNMSSPPHGSPGLTSSQQNLMISPRHRGSPKMGSHQFSPVAGMHSPMGSATNTSNNS
FSSSSLSALQAISEGVGTSLLSTLSSPGPKLDNSPNMNIAQQSKMGNQDSKSPVLYCEQSQVESSMCQSNSRDL
LTDKDNKEGSLEGSESQRGPSESKGHKKLLQLLTCSSEERGHATLSNSPLDSNCKESSTSVTSPSGVSSSTSGG
VSSSSNMHGSLLQEKHRILHKLLQNGNSPAEVAKITAEATGKDTYHESSNTTPCGEGTIKQEQLSPKKKENNAL
LRYLLDKDDIKDPLSKDIKPKVESLDNKMGQCSSSSISTSNQEKEIKVKIEPSEEMSGDLDNLDAILGDLTSSD
FYSNANSANGSNLGTKQAIYQGNAPLGMRSPQSMQATRPPFNRAMSLDSPIPMGSSPPVRSVNAFSMLQKQNMM
GGNSRMIESQENFGANIGAPNRSMTMNQHPAGDWGLPNSKVNRLEPTSSSSMMRPGADYNTSLPRPTIGSSVPG
LPIRSNSIPGTRPILQQQMIHMRPNEINMGMGGNPYAQPGPSSQPASWPDSMLAIEQGSRGSQSRQLVRSSLDD
LLCPPPNVEGQNDERALLDQLHTLLSNTDVTGLEEIDRALGIPDLVNQNLTLFEPYSLKITSEHSLSLVRKALE
IYFYKNAGQTLEPKQDTFQGQESAVMIDQKPPLYGQPYQGQGTAVPGGFSNMQGQQPSFNSMMNQISHQSNFPL
PNVHPRASVMRPRTNTPKQLRMQLQQRLQGQQFMNQTRQALEMKMENPGATVAPVMRPVMQPQVGSQQQGFLNA
QMMAQRNRELLSHHIRQQRMAMIMQQQQQQQQPQAFSPPPNVTASGSMDSALAGPPMAQVPPQQFPYPPNYGIN
QQPDPAFSRVSSPPNPMMSSRIGPSQNPMMQHPQTAPMYQSPEMKGWPSGTMARSSSFSQQQFSHQGTPATYNM
MHMNGTGGHIGQMNLNTMPMSGMPMGPDQKYC
>220Alligator sinensis|A0A3Q0FJK3|Sarcopterygii|SRC3
MSGLGENSLDSLTNESRKRKSLPCDTPGPGLNCSGEKRRREQESKYIEELAELISANLSDIDNFNVKPDKCAIL
KETVRQIRQIKEQGKTASSDDDVQKADVSSTGQGVIDKDSLGPLLLQALDGFLFVVNRDGNIVFVSENVTQYLQ
YKQEDLVNTSVYSILHEEDRKDFLKNLPKAAVNGVAWTNETPRQKSHTFNCRMMVKTCHDLLEDVGSNQDTRQR
YETMQCFALSQPRAMMEEGEDLQSCMICVARRITTVERVFPANPESFITRHDLSGKLINIDTNSLRSSMRPGFE
DIIRRCIQRFLCHNDGQTWSNKRHYQEAYIQGHAETPLYRFSLADGTIVTAQTKSKLFRNPVTNDRHGFVSTHL
LQREQNGYRPNPNPVGQSIRAPTAGSTNSVGAIMNMPPGQSMPIQNRNYGMGDPSAVGQMSSTRYGGPGNMGPM
NSGPGMQSSPFQNNNYGLNMSSPPHGSPGLTSSQQNLMISPRHRGSPKMGSHQFSPVAGMHSPMGSATNTSNNS
FSSSSLSALQAISEGVGTSLLSTLSSPGPKLDNSPNMNIAQQSKMGNQDSKSPVLYCEQSQVESSMCQSNSRDL
LTDKDNKEGSLEGSESQRGPSESKGHKKLLQLLTCSSEERGHATLSNSPLDSNCKESSTSVTSPSGVSSSTSGG
VSSSSNMHGSLLQEKHRILHKLLQNGNSPAEVAKITAEATGKDTYHESSNTTPCGEGTIKQEQLSPKKKENNAL
LRYLLDKDDIKDPLSKDIKPKVESLDNKMGQCSSSSISTSNQEKEIKVKIEPSEEMSGDLDNLDAILGDLTSSD
FYSNANSANGSNLGTKQAIYQGNAPLGMRSPQSMQATRPPFNRAMSLDSPIPMGSSPPVRSVNAFSMLQKQNMM
GGNSRMIESQENFGANIGAPNRSMTMNQHPAGDWGLPNSKVNRLEPTSSSSMMRPGADYNTSLPRPTIGSSVPG
LPIRSNSIPGTRPILQQQMIHMRPNEINMGMGGNPYAQPGPSSQPASWPDSMLAIEQGSRGSQSRQLVRSSLDD
LLCPPPNVEGQNDERALLDQLHTLLSNTDVTGLEEIDRALGIPDLVNQNLTLFEPYSLKITSEHSLSLVRKALE
IYFYKNAGQTLEPKQDTFQGQESAVMIDQKPPLYGQPYQGQGTAVPGGFSNMQGQQPSFNSMMNQISHQSNFPL
PNVHPRASVMRPRTNTPKQLRMQLQQRLQGQQFMNQTRQALEMKMENPGATVAPVMRPVMQPQVGSQQGFLNAQ
MMAQRNRELLSHHIRQQRMAMIMQQQQQQQQPQAFSPPPNVTASGSMDSALAGPPMAQVPPQQFPYPPNYGINQ
QPDPAFSRVSSPPNPMMSSRIGPSQNPMMQHPQTAPMYQSPEMKGWPSGTMARSSSFSQQQFSHQGTPATYNMM
HMNGTGGHIGQMNLNTMPMSGMPMGPDQKYC
>221Callorhinchus milii|A0A4W3JDD5|Chondrichthyes|SRC3
SDAIGNVNCSIEKRRREQESKYIEELAELISANLSDIDNFNVKPDKCAILKETVRQIRQIKEQGKASSNEDDVQ
KADVSSTGQGVIDKDSLGPLLLKALDGFLFVVNRDGNIVFVSENVTQYLQYKQEELMNTSVYNILHQEDRDEFL
RNLPKSAVNGVSWTNETQRQKSHTFNCRMLVQMPYVSLDESSGSHEPRQKHETMQCFALSQPKAITEEGEDLQS
CMICVARRITPVERSQFPSSTESFVTRHDMTGKITSIDPSSLRASMKPGWEDLVRRCIQRFFHHNDGQPWSLKR
HYHEALMQGQAESSFYRFSLSDGTVVTAQTKTKLLRNPVTNEPLGFSSTHVLQREQNGCRMNQNTMGQEMMKTL
NNMNDPNSMGQMASNRFSGGGGGAMSHGAGMQAPALQGSNYALSMNSPPHSSPGMSSTQQNLMLSPRHRMSPRM
GPSQYSSSGGMQSPVGHMNNMGSSTFSSSSLNALQAISEGLGVSLLSSLSSPGQKMENSPNVSLSQPPKLGSVD
LKDSPGLFGEHSQVESTVGRPESRDAHSEQKNIKEGNLNHFVGGDNPEGQRRFHENKNHTKLLQLLTSPSDDMG
LTSLTNSSLDSGIKDSSGGITSPSGVSSTSVAAEKHKILHKLLKNGNSPAEVAQLTAEATGKESCQDSGNTTAV
GEAVIKQEQLSPKKKESNNLLRYLLDKDDPKDPALNDIKPKIEGMDGKMDACNAGKPTGSIQVKDELHIKTERP
DQVREATKYFWENECHRVNLWRTHGGLSVAQQSAMATGEWGLANVAAGNASMMRPGADYSSSIQRAMVGNPALA
MQMRTNGQPGPRTMLQQQMMGMRPSEMDMSLGGPQFGQQQVPPNQTTSWSDNVLQMEQVHSGGQGRQPFGGSLD

DLLCPPTTAEAQSDEKALLDQLDTLLSNADEMGLEEIDRALGIPDLISQVQPLEAHADQFSVQDTALMLEQKAM
YGQQFNSQAGPMQGCYPSMQGQQPGFNAMMNQMGQQGSFPLASFHPRATFARHRMMNAPAPKQLRLQLQQRLQG
QQVGAGFLNAQMMQRHREILTQQLRQRQMMHRAMLMRQQQQQQQQQQQQGFSPPPNVTAPTGMEGPMGGPQLTP
ASPQQFSYPANYGMNQQSDPAFGRAPSPHNTLMSRMAPPQNPMMQHPQTAAVYQTTTDMKGWSTGNMGGNRCD
>222Pangasianodon hypophthalmus|A0A5N5LTS3|Actinopteri|SRC3
MSGVGENSMEPLCPDRKRSLSTCDTPGLGCEKRRREQESKYIEELAELISANLSDIDSFNVKPDKCAILKETVR
QIRQIKEQGKASSSDDDVQKADVSSTGQGVIDKDHLGPLLLQALDGFLFVVNREGSIVFVSDNVTQYLQYTQEE
LINTSVYNILHEDDRDDFHKNLPRNNVNSMAWGGEAPRQKSHTFNCRMLVKFSSGPAEENPGTPRYETMQCFAL
TQPKAMMEEGEDLQSCMICVARRVTTLERTESFNTRHDPSGKLIQIDQNSLRASMRPGWEDLLRRCIQMFLQHS
DGQPWSNKRHYHEAFLHGHAETPVYRFSLSDGTPVTAQTKSKLYRNPMTNEPQGFISTHLLQREPNGCRTNQSG
GMMQGGMRQQGMAGTNSNSQMNMGPGGGMGMGSMGGMGGMGMNRGYGMKDMGHMGHMGGGGSMYAASMGNRMMQ
MNQMNQMSQMGQINPMGQMNQMNPMAQMNPMNHPGPGMQHQHQQPLFQASGGYGLGGMNSPSQGSPGLNGPQQG
LLMSPRNRGSPKMASSQFSPGGMHSPMGPGSVGGSSGGGGGTTFSSSSLNALQAISEGVGASLPSTLTSPSTAH
KPDSSPSVHSSSSSSQPCKPGHGDSKSPAGAYSHGPGDQHHPLHHHHHHSHTHQAESSSADRPDSQAGGGSKES
GEATNEAGNASSEPPRKIPDGKGHKKLLQLLTSPTEELGLGVGSGPPGPPTGATLEGKEPGCVTSPSSTGVSSS
TNQSQVLGAAGGGPSEALGSSHYTASLQKKHKILHKLLQNGNTPDEVAKITAEATGKAMLGQDAGAAGGETGGT
SAGPGGVSGTSEPKQEQHSPKEKKTNALLHYLLNNKDDSNSKESSDIKPKPEDLEGKGVTGGNSGTPLSRGAQE
NMEGKIKSEPPDELDTILGGLRGSGTDFPEQGDAGGAPDGGNKPHSFADDGMMNRGMGMPERSPMGPAGDWGMP
RSTGSPSIMRPAIDSYNSKGMMGGPMVNRSNSVPGTRSMLQQQLMDMGSSDMSMAMGPFSQQQCPDSQSPSWPD
NMMGMDNRRQFGNTLDELLVPPSTSEGQSDERALLDQLDSLLNNTDVLALEEIDRALGIPDIISQGQGGDLQPE
PFPTQDPSMVMEQKPMYPQGYPGPPSMPMPSGYGASPMQGAPPPAPYSPMLNQMGQQGNYPGMGGMGPMGGMGH
PRAAMMRPRMMSATKPTRLQLQQRLQGQQFMSQARQGMGLKMENPGGGAGPGMRPGLQPAMGGQPGFLNAQMIA
QRNREMQMRRHRMMLLMQQQAAGGGFSPPPNVTAPTGMEGMGGPGMGQPGQQQFSYGGNYGMGQQGEPSFAPAG
GPSPPTAMMPGRMVPPQNPMMQQHPQGGPMYQSPDMKGWPQGGMTRNSSYPQQAFAQQGSAAPYGSMMMNGANA
GHMGQMSMGMMGRMPMGPDQKYL
>223Athene cunicularia|A0A663N3U3|Aves|SRC3
MSGLGENSLDSLTNESRKRKPLPCDMPGASLTCSGEKRRREQESKYIEELAELISANLSDIDNFNVKPDKCAIL
KETVRQIRQIKEQGKAVSNDDDVQKADVSSTGQGVIDKDSLGPLLLQALDGFLFVVNRDGNIVFVSENVTQYLQ
YKQEDLVNTSVYGILHEEDRKDFLKNLPKSSVNGVTWTNETPRQKSHTFNCRMMVKTSHDILEDVGSHQDIRQR
YETMQCFALSQPRAMMEEGEDLQSCMICVARRITTAERVFPTNPESFITRHDLSGKLVNIDTNSLRSSMRPGFE
DIIRRCIQRFLCHNDGQSWSNKRHYHEAYTQGHAETPLYRFSLADGTIVTAQTKSKLFRNPVTNDRHGFVSTHF
LQREQNGYRPNPNSVGQNIRTPVAGNTNSVGGLMNMSPGQGMAMQNRNYGMGDPSAVGQLSSTRYGGPGNMGPV
NSGPGMQSSAYQNNSYGLNISSPPHGSPGLTSNQQNLMISPRNRGSPKMSSHQFSPVTGMHSPMGSASNTGNST
FSSSSLSALQAISEGVGTSLLSTLSSPGPKLDNSPNMSIAQQSKTSTQDSKSPSGLYCEQNQVESSICQSNSRD
LLSEKDNKEGNLESSESQRGPSESKGHKKLLQLLTCSSDERGHSTTSNSPLDSNCKESSTNVTSPSGVSSSTSG
GVSSSSNMHGSLLQEKHRILHKLLQNGNSPAEVAKITAEATGKDTYHDASNTAPCGEGTVKQEQLSPKKKENNA
LLRYLLDKDDVKDPLSKELKPKVESVDNKMGQCSSSTIPTSSQEKEMKIKTEPAEEMTGDLDNLDAILGDLTSS
EFYNTAMSANGNNLATKQALFQGNAILGMRSPQSVQTTCPPFSRAMSLDSPVGSSASTRNVNAFSMLQKQNLMG
GSPRMIENQENFGSNLGFAPNRNVAMNQHQSGDWGLPNSKVNRLESTSSASMLRPGAEFSASLPRPTVGGTMAG
LPVRSNSIPGARPMLQQQMIHMRPNEINIGMGGNPYGQPGPSNQPGSWPDNMLSMEQASRGSQNRQLVRNSLDD
LLCPAPNMEGQNDERALLDQLHTLLSNTDVTGLEEIDRALGIPDLVNQGQALEPKQDSFQGQESSVMIDQKPSL
YGQPYQGQGAAMPGGFTNIQGQQPSFNSVMNQMSQQSNFPLQSMHPRANVMRPRTNTPKQLRMQLQQRLQGQQF
MNQTRQALEMKMENPVSGNTSVMRPVMQPQVGSQQGFLNAQMVAQRNRELISHHFRQQRMAMMMQQQQQPQAFS
PPPNVTASGSMDSALTGPPMAQVPPQQFPYPPNYGLSQQPDPAFSRVSSPPNPMMSSRIGPSQNPMMQHPQTAT
MYQSPEMKGWPSGSMARSSSFPQQQFSHQGNPAAYSMMHMNGSSGHIGQMNINTMPMSGMPMGPDQVNCT

>224Myripristis murdjan|A0A668AI00|Actinopteri|SRC3
MSGVGENPLEPLCSDRKRKLSTCDTPGLGCDKRRREQESKYIEELAELISANLSNIDSFNVKPDKCAILKETVR
QIRQIKEQGKSSCNNDDVQKADVSSTGQGVIDKDHLGPLLLQALDGFLFVVNRDGSIVFVSDNVTQYLQYKQEE
LINTSVYNILHEEDREEFHKNLPKSNAPNGASWGGEAPRQKSHTFNCRMLVKFAHGQSHGPSEEGPGGQRYETM
QCFALTQPRAMMEEGEDLQSCMICVARRITAVERTERFSTRHELSGKLIEIEQQSSLHTTMRPGWEDLVRRCMQ
MFLHRSEGQAWSYKRHFHDAFLHGHAETPLYRFSLSDGTPVTAQTRSDLCRNPNSNEPPTFLSTHLLQREQNGY
RSNQAGGMRPQGMGVNNPNPQMNMGPGGGMGMGMNRGYVMGEHGNMPPRGVPPYAGGNRMNPMNQMNPMHQMNQ
MNSMHQMNNMGQMNQMNQMNSMHPMNSSMNSVNQMGSMNQMNQMNQMQMGQFHGGGGGGGYGMGMTSPPQASPG

MNGPPHNVMGSPRVRGSPKMGARPFSPGGMNSPISSSHPGNSGSGGGGTTFSSSSLNALQAISEGVGNPMPSPL
TSPPPHKPDSSPSINSTNQSQAQGGPCKPGLSAHSDSKSPGTSLAAGGEQQTQQHPHTPITEGPPDKPDSQASR
EGGQTGGEPNRRVPDTKCHKKLLQLLTAPTDELVPPNPTPSSGPNSTPEAKEGATSVTSPSSSTGVSSSTGGIG
QQSLGTVSCSSGTGHCTNQSLQEKHKILHKLLQNGNTPDEVARITAEATGKSTLDTGGPEAGATAIGGVRGSES
KQEQHSPKKEKTHALLHYLLNKDDPIESGDIKPKLEELEGRGTQGPGISSSDPHSTDGKVKMEPPDEVKLLTQS
MGRGIGMQQRPPMAGTGDWGMPRPNASPVGGPGHPGMGRSGMMGGPMINRSNSVPGNTRSMLQQQLMDMGMSHV
HGNMGMSPFGGQGPPPQSPSWPDSAMGLDRPFGNPLDELLVPPSTSEGQSDERALLDQLDSLLNNTDVIALEEI
DRALGIPDLVGQGQGQGQCPPSEPFQGPDSSMGMDQKPMYGQGYPGPPGPPSMGMQPGYGSNPMQGQSPGGFNP
MMNQMGQPGGFPGMGGMGGMGNPRANMMRPRMMNATKPLRLQLQQRLQGQQFMNQTRQGMKMENAPGGNPAMRP
GMQPGMQPPGMGGQPGFLNAQMMAQRSREMMTMQMRRQRMMMLMQQQQQQQQQQQGQGAAGGFSPPPNVTAPGG
MDSPMGGPPMNQPGQQAFNYGGNYGMNQQGDPSFMGPGSSPPGNMMPGRMGGPPQNAMMPGMQGNPQGGPMYQS
GEMKGWPQGNMPRNSSYPQQQFPQQGNQGQFGPMMMNSPMGGPGPVSGPGAGQMGQMPGQMQSQMQGQMGMNTM
GMGRMPVGPDQVNEECSGFK
>225Phasianus colchicus|A0A669NWK9|Aves|SRC3
MSGLGENSLDSLTSESRKRKPLPCDTPGASLTCSGEKRRREQESKYIEELAELISANLSDIDNFNVKPDKCAIL
KETVRQIRQIKEQGKAVSNDDDVQKADVSSTGQGVIDKDSLGPLLLQALDGFLFVVNRDGNIVFVSENVTQYLQ
YKQEDLVNTSVYGILHEEDRKDFLKNLPKSSGNEINGVAWTNDTPRQKSHTFNCRMMVKTSHDLLEDVGNHQDT
RQRYETMQCFALSQPRAIMEEGEDLQSCMICVARRIATAERVFSTNPESFITRHDLSGKLVNIDTNSLRSSMRP
GFEDTIRRCIQRFLCHNDGQSWSNKRHYHEAYTQGHAETPLYRFSLADGTIVTAQTKSKVFRNPVTNDRHGFVS
THFLQREQNGYRPNPNAVGQNIRTTVAGNTNSVGLMNMSPGQGMPMQNRNYGMGDPNTVGQLSSSRYGGPGNMG
PVNSGPGMQSSAYQSNSYGLNISSPPHGSPGLTSNQQNLMISTRNRGSPKMNSHQFSPVTGMHSPMGSASNTSN
STFSSSSLSALQAISEGVGTSLLSTLSSPGPKLDSSPNVSIAQQNKANNQDSKSPSGLYCEQNQVESSICQSNS
RDVLSEKDSKEGSLDASESQRGQSESKGHKKLLQLLTCSSDERGHSTASNSPLDSNCKESSTNVTSPSGVSSST
SGGVSSSSNVHGSLLQEKHRILHKLLQNGNSPAEVAKITAEATGKDTYHDTSNTVPCGEGAVKQEQLSPKKKEN
NALLRYLLDKDDVKDPLSKELKPKVENVDNKMGQCSSSTIPTSSQEKEMKIKTEPTEEMSGDLDNLDAILGDLT
SSDFYNNAMSTSGSNFGTKQALFQGSAPLTGMRSPQSVQTARPPFNRAMSLDSPVGSGASVRNVNAFSMLQKQN
LMGGSPRMTENQESFGANIGFTPNRSVSMNQHQSGDWGLSNSKVNRLDPTSSTSMLRQGAEFSASLPRPTAGGS
VPGLPIRSNSIPGTRPMLQQQMMHMRSNEINIAMGGNPYGQPGPTSQPGSWPDNMLSMEQASRGLQNRQLVRNS
LDDLLSAAPSVEGQNDERALLDQLHTLLSNTDVTSLEEIDRALGIPDLVNQGQTLESKQDSFQSQESAVMIDQK
PSLYGQPYQGQGTALPGGFNNIQGQQPSFNSVMNQMSQQNNFPLQSMHPRANAMRPRTNTPKQLRMQLQQRLQG
QQFMNQTRQALEMKMENPGSSNSSVMRPVMQPQQGFLNAQMVAQRNRELISHHFRQQRMAMMLQQQQPQAFSPP
PNVTASGSMDSLTGPPMAQVPPQQFSYPPNYGISQQPDPAYGRVSSPPNPMIASRIGPSQNPMMQHPQTAPMYQ
SPEMKGWPSGSMARSSSFPQQQFSHQGNPATYSMMHMNGSSGHIGQMNISTVPMSGMPMGPDQVSCT

>226Hypotaenidia okinawae|A0A6G1S071|Aves|SRC3
MFKMSGLGENSLDSLTNESRKRKSLPCDTPGASLSCSGEKRRREQESKYIEELAELISANLSDIDNFNVKPDKC
AILKETVRQIRQIKEQGKAVSNDDDVQKADVSSTGQGVIDKDSLGPLLLQALDGFLFVVNRDGNIVFVSENVTQ
YLQYKQEDLVNTSVYGILHEEDRKDFLKNLPKSSVNGVAWTNETPRQKSHTFNCRMMVKTHDLLEDVGSHQDIH
QRYETMQCFALSQPRAMMEEGEDLQSCMICVARRITTAERVFLTNPESFITRHDLSGKLVNIDTNSLRSSMRPG
FEDIIRRCIQRFLCHNDGQSWSNKRHYHEAYTQGHAETPLYRFSLADGTIVTAQTKSKLFRNPVTNDRHGFVST
HFLQREQNGYRPNPNSVGQNIRTPVAGNTNSVGGLMNMSPGQGMPVQNRNYGMGDPSAVGQLSSARYGGPGNMG
PVNSGANMQSSAYQNNNYGLNISSPPHGSPGLTSNQQNLMISPRNRGSPKMSSHQFSPVTGMHSPMGSASNTSN
STFSSSSLSALQAISEGVGTSLLSTLSSPGPKLDNSPNMGMTQQSKASTQDSKSPSGLYCEQNQVESSICQTNS
RDLLSEKDSKEGNLEASESQRGPSESKGHKKLLQLLTCSSEERGHSSNSPLDSNCKESSTNVTSPSGVSSSTSG
GVSSSSNMHGSLLQEKHRILHKLLQNGNSPAEVAKITAEATGKDTYHDASNAAPCGEGTVKQEQLSPKKKENNA
LLRYLLDKDDVKDPLSKELKPKVENVDNKMGQCSSSTIPTSSQEKEMKIKTEPAEEMSGDLDNLDAILGDLTSS
EFYSNAMSANGNNLGTKQALFQGNALLAGMRSPQSVQATRPTFNRAMSLDSPVGSSAPVRNVNPFSMLQKQNLM
GGSPRMIESQENFGANLASAPNRNVAMNQHQAGDWGLPNSKVNRLEPTSSASMLRPGPEFSASLPRPTVGGGSM
SGLPVRSNSLPGTRAMLQQQMIHMRPNEINIGMGGNLYGQPGPSNQPGSWPENMLSMEQASRGSQNRQLVRNSL
DDLLCPAPTMEGQTDERALLDQLHTLLSNTDVTGLEEIDRALGIPDLVNQGQALEPKQDSFQGQESSVMIDQKP
SLYGQPYQGQGAAMPGGFSNIQGQQPSFNSVMNQMSQQSNFPLQSMHPRTNVMRPRTNTPKQLRMQLQQRLQGQ
QFMNQTRQALEMKMENPVSSNTSVMRPVMQAQVGSQQQGFLNAQMVAQRNRELISHHFRQQRMAMMMQQQQPQ
AFSPPPNVTASGSMDSALTGPPMAQVPPQQFPYPPNYGISQQPDPAFSRVPSPPNPMMSSRIGPSQNPMMQHPQ
TAAMYQSPEMKGWPSGSMARSSSFPQQQFSHQGNPAAYSMMHMNGSSGHIGQMNINTMPMSGMPMGPDQKYC

>227Ornithorhynchus anatinus|A0A6I8NK20|Mammalia|SRC3
MFKMSGLGENSLDPLTADSRKRKLPCDTPGQGLTCSGEKRRREQESKYIEELAELISANLSDIDNFNVKPDKCA
ILKETVRQIRQIKEQGKATPNDDDVQKADVSSTGQGVIDKDSLGPLLLQALDGFLFVVNRDGNIVFVSENVTQY
LQYKQEDLVNTSVYSILHEEDRKDFLKNLPKSAVNGVAWTNETPKQKSHTFNCRMSVKTSHDLLEDVGSSPDPR
QRYETMQCFALSQPRAMMEEGEDLQSCMICVARRLTTVERVFPSNPESFITRHDLSGKVVNIDTNSLRSSMRPG
FEDIIRRCIQRFFCLNDGQPWSHKRHYQEAYIQGHAETPVYRFSLADGTIVTAQTKSKLFRNPVTNDRHGFVST
HFLQREQNGYRPNANPLGQGGRPPPAGCPSSVSGVNVSPGQGVPVPTRSYGAGDPGALGQMGGPRYGGAGGGGG
GGGPNPLGPVNPGPAMQPAPYQTSGNGGGGGGGYGLNMSSPPHGSPGLSSSQPNIMISPRNRGSPKMAPHQFSP
VAGLHSPMGSAPNAGGPSFSSSSLSALQAISEGVGTSLLSTLSSPGPKLDHSPNVPPAQPAKTGGQESKSPPGP
YREPSPAESSVCPSGGRDHPGDKEAKESGPEGAEHPRGPPESKGHKKLLQLLTCSSDDRGHSALTGSPLDPGCK
DAAGAASPSGVSSSTSGGVSSTSNVHGSLLQEKHRILHKLLQNGNSPAEVAKITAEATGKDTGNGSAPCGDGPV
KQEQLSPKKKENNALLRYLLDRDDPTDPLSKEINPKVEGADNKSGQCSSSSGGSAVPTSSQEKEVKVKTEPAEE
VSGDLDNLDAILGDLTSSDFYNNAMSANGNNLGTKQGMFQGNTPLGLRSPQSVQPTRPPFNRAVSLDSPIAVGS
SPAVRNVNAFSMLPKQPIMGGSPRMMESQDGFGPNAGGPNRNMAMSQLASGDWGLATSKVNRMEASAAGPIARA
GSDYGLPRSAMGGSVPGLPMRSNSIPGARPIPQQQMIHMRPTEMSMGMGVNPYSQQAPANPPNSWAESMLSMEQ
GPRGTQNSRQLVRNSLDDLLCPPPNVEGQSDERALLDQLHTLLSNTDVTGLEEIDRALGIPDLVNQGQALEPKQ
DSFQGQESAVLMDQKTALYGQSYQTQGAAMQGGFSNLQGQQQSFNTMMTQMGQQGNFPLQGMHPRASVMRPRSN
TPKQLRMQLQQRLQGQQFLNQNRQALEMKLDNPAGGGSAVMRPMMQAQVNSQQGFLNAQMVAQRNRELISHHIR
QQRVAMMMQQQQQQQQQQQQQQQQQQQQQQQQQQQQQQQQPQTFSPPPNVTAAAGLDGGLAGPAMPPAPPQQYPY
QPNYGMSQQPDPAFSRVSSPPGAMMSTRMGPSQNALMQHPQAASMYQSPEIKGWPSGNMARNSSFSQQQFGHQS
NPAAYGMMHMNGGGGGGGGHMGPMNMNSMAMSGMPMGPDQVTGFYPLAFQGPAGDRRDRPSPHANPQLSSNVHL
VPPGRGWAVDEQANL
>228Chanos chanos|A0A6J2UZQ9|Actinopteri|SRC3
MSSLGENSMEPLCSDRKRKLSTCDTPGLGCDKRRREQESKYIEELAELISANLSDIDSFNVKPDKCAILKETVR
QIRQIKEQGKSVSSDDDVQKADVSSTGQGVIDKDHLGPLLLQALDGFLFVVNREGNIVFVSDNVTQYVQYKQEE
LINTSVYNILHEEDREEFHKNLPKNNVNGVSWGGETPRQKSHTFNCRMLVKYGHDEAPGGPRYETMQCFALTQP
KAMMEEGEDLQSCMICVARRVTAVERTESFNTRHELSGKLIQIDQNSLRASMRPGWEDLLRRCIQMFLQHSDGQ
PWSHKRHYREAFLQGHAETPVYRFSLSDGTPVAAQTKSKLYRHPMTNEPQGFISTHLLQREQNGCRTSQAAGMI
QGGMRQQGMGGPNPGAQMNMGPGGGMGGMGGMGMNRGYGMNDMNHMSHMGQMNSGSMYGSNGGNGNNMGNRMMQ
MNQMNQMNQMNQMNQMSQMNQMSQMNQMSQMNQINHPGPGMPQQQHQQPPFQASGGYGLAGMNSPSQGSPGMSG
PQQNLLMSPRNRGSPKMASSQFSPGGMHSPIGPGSGGGVGGGSSFSSSSLNALQAISEGVGASLPSLPSALTSP
SPAHKPDSSPSVHSSSQSNQTCKQSHGDSKSPAGAFGSGAGDHHHPLHHQHHHHHNHSHPAESSGDRPDSQAGA
GAKESGEGSGEAGVTPAEPPRRVPDSKGHKKLLQLLTSPTEELSLGVGSGPSLPIPPGASSNSVEGKEAGCVTS
PTSTGVSSSSNQGQGLGAGGGSSALNSSNYTASLQEKHKILHKLLQNGNTPDEVAKITAEATGKVVLGQEAGGG
AGEPGGGPGMASGSSAGSTEPKQEQHSPKKEKTHALLHYLLNKDDSKEPADVKPKPEDMEGKGPTGGSSGPTAT
GAPPDNLEGKIKSEPPDELDSILGGLRSSGPEFPSEQGTVGGAAEGGSKPNCFPDESTQVSSPGMGSGGPRVPF
QRAMSVDGKAQGEPGLAGRRSAPCPMPVKQESLDSQRMMGASDSFPGNMGMMNRGMGVPQQSPMGGNNEWAMQR
SSSSPVGSAGHPSMVRPGMDSNFKGMMGGPTVSRSNSMPGTRSMLQQQLMDMGSGDMNMGMSPFNQQCPSGQSP
SWPDNVMGVDNNRRQFGNTLDELLVPPSTSEGQNDERALLDQLDSLLNNTDVIALEEIDRALGIPDLISQGQGM
EPQGDPFPGQDISMAMEQKPPYGQGYPGPPSMGMPTGYGGNPMQGQPPPAGYGPMMSQMGQQGGFPGMAGMGGL
GPMGGMGHPRASMMRPRMMTANKPMRLQLQQRLQGQQFMNQARQGMGMKMEPPGGGNPAMRPGMQPGMSGQAGY
LNAQIMRNREMMRRHRMMMMMQQQQQAAAAGGFSPPPNVTAPPGMDGGLAGPPMNQQQFGYGGSYGMGQQGDPS
FVPSGGSPPNAMMSGRLGPPQNPMMQQHPQGGPMYQTADMKGWPQGGMGRNSSYTQQQFGQQGAAGQYGGMMMN
GGMGGNGGHMGQMPGQMNMNSMVMSRMPMGPDQKY
>229Chanos chanos|A0A6J2VTP0|Actinopteri|SRC3
MSGVGENSLEPLGSDRKRKLSTCETPGLGCDKRRREQESKYIEELAELISANLSDIDSFNVKPDKCAILKETVR
QIRQIKEQGKSSCGDDDVQKADVSSTGQGVIDKDHLGPLLLQALDGFLFVVNREGSIVFVSDNVTQYLQYKQEE
LINTNVYNILHEDDREELHKNLPKGNVPSGVSWAGDTSRQKSHTFNCRMLVKFGRGPGEEGPNAPRYETMQCFA
LTQPRAMMEEGEDLQSCMICVARRVTAVERTERFSTRHELSGKLIEIEQQSSLHTTMRPGWEDLVRRCMQMFLH
RSESQPWSYKRHYNEALMHGRAETPLYRFSLSDGTPVTAQTRSELCRNPATNEPHSFLSTHLLQREQNGYRPNQ
GSVMRAQGMGGAQPNAQMNMMPGGGMGMGGNRGYGMNEPGHMGPRGGPMYGAGSRMNQMNPMHPMNQMNQMNQM
NQMNQMNSMNQMNQMNQMNSMGHLNQMNSMNQMNSMNHINQMNQMNQMNHMNQMNSMNQMNSMNQMNHPGMQPH
QQPPFHGGNYGLMGNSPSQASPGINVSQQNIMGSPRIRGSPKMGASPFSPGGMPSPMGSVSAGGTGGTSGSTGG

GSFSSDSLNALQAISEGVGSSLPSTLTSPAHKSDGSPSVNSTQPNQGQTGVCKSSHSDSKSPASAHSGGVEQQQ
QHHHHTSASETAMEKPDSQASKEGQAGTEPSRRLSDTKGHKKLLQLLTSPPEELNLAAGSGGSNVPPSDRASTP
MGADAKEPGGCMTSPSSTGVSSSSSSGNAANQQGSGGVSSTACSGHYPNHSLQEKHKILHKLLQNGNTPDEVAR
ITAEATGKVPVGQEHTGADSRGSEVKLEQHSPKKEKTHALLHYLLNKDDSKEPADSKPKLEELKGRGAAGPGSG
VTSSNLNTQEGKVKMEPPDELDNLESILGGLRDSGSGMFGDSGAGGRAERDNKQGNGPNSTAHDREGVGMGPGP
RVPFQRAMSMDAKPLSGPGMTGRRSAPCPSHIKQENSDMPMGMGAPNRTMGVGQRPPMMGPGEWGMPNSSGSPV
GPMDHPSMGRPGMDHNSKGMLGGPMISRSNSLPATRSMLQQQLMDMGPNDVGMGMNAFRGQGPPPQSPSWADSG
IGMEGPPNSNRPPFGNPLDELLVPPSTSEGQSNERELLDQLDSLLNNTDVIGLEEIDRALGIPDIVSQGHNTEQ
QQQQQQQQQLPADPFPGPDPSMSMDPKAMYGQGYPGPQSVGMQSGYGGGPMQGQSPAGFNPMMNQMGQQGGFPG
MGGMHPRASMMRPRMMSATKPLRLQLQQRLQGQQFMNQTRQAMGMKMDNMPGGNPGLRPGMQPGMGGQPSFLNA
QMLAQRNREMMMMRRQRMMMLVQQQQQQAAAGGFSPPPNVTAPGGMDNSMGGPPMNQPGQQPFSYGGNYGMNQQ
GDPSFVDTGSSPPSGMMPGRMAGPQNPMMQQHPQSGPMYQSAEMKGWGPGGMPMNNPYPQQQFPQQGNPGQYGG
MMMNGSMQGTVNGAGAGQMPPMQGQMGMNSMGMGPMPMGPDQKYC
>230Sapajus apella|A0A6J3G1H8|Mammalia|SRC3
MSGLGENLDPLATDSRKRKLPCDTPGQGLICSGEKRRREQESKYIEELAELISANLSDIDNFNVKPDKCAILKE
TVRQIRQIKEQGKTISSDDDVQKADVSSTGQGVIDKDSLGPLLLQALDGFLFVVNRDGNIVFVSENVTQYLQYK
QEDLVNTSVYNILHEEDRKDFLKNLPKSTVNGVSWTNESQRQKSHTFNCRMLMKTHDILEDINTSPEMRQRYET
MQCFALSQPRAMMEEGEDLQSCMICVARRITTGERVYPSNPESFITRHDLSGKVVNIDTNSLRSSMRPGFEDII
RRCIQRFFSLNDGQSWSQKRHYQEAYLNGHAETPVYRFSLADGTIVTAQTKSKLFRNPVTNDRHGFVSTHFLQR
EQNGYRPNPNPVGQGIRPPMAGCNSSVGGMSMSPNQGLQMPSSRAYGLADPSTTGQMSGARYGGSSNIASMTSG
PGMQSPSSYQNNNYGLNMSSPPHGSPGLAPNQQNIMISPRNRGSPKMASHQFSPVAGVHSPMGSSGSTGSHSFS
SSSLSALQAISEGVGTSLLSTLSSPGPKLDNSPNMNITQPSKVSSQDSKSPLGFYCDQNPVESSMCQSNSRDHL
SDKESKESSIEGSENQRGPLESKGHKKLLQLLTCSSDDRGHSSLTNSPLDSSCKESSVSVTSPSGVSSSTSGGV
SSTSSMHGSLLQEKHRILHKLLQNGNSPAEVAKITAEATGKDTSSITSCGDGNVVKQEQLSPKKKENNALLRYL
LDRDDPSDALSKELQPQVEGVDNKMSQCTSSTIPSSSQEKDPKIKTETSEEGSGDLDNLDAILGDLTSSDFYNN
SVSTNGSHLGTKQQIFQGTNSLGLKSPQSVQSIRPPYNRAVSLDSPVSVGSSPPVKNISAFPMLPKQPMLGGNP
RMMDSQENYGSSMGGPNRNVTVTQTPSSGDWGLPNSKASRMEPMNSNSMGRPGGDYNTSLPRAALGGSVPTLPL
RSNSIPGARPMLQPQQQQQQMLQMRPGEIPMGMGANPYGQAASNQLGSWSDGMLSMEQVPHGTQNRPLLRNSLD
DLVGPPSNLEGQSDERALLDQLHTLLSNTDATGLEEIDRALGIPELVNQGQALEPKQDAFQGQEAAVMMDQKAG
LYGQTYPAQGPPMQGGFHLQGQSPSFNSMMNQMNQQGSFPLQGMHPRANIMRPRTNTPKQLRMQLQQRLQGQQF
LNQSRQALELKMENPTAGGAAVMRPMMQPQVSSQQGFLNAQMVAQRSRELLSHHFRQQRVAMMMQQQQQQQQQQ
QQQQQQQQQQQQTQAFSPPPNVTASPSMDGLLAGPAMPQAPPQQFTYQPNYGMGQPPDPAFGRVSSPPNAMMSS
RMGPSQNPMMQHPQAASMFQSSEMKGWPSGNLARNSSFSQQQFAHQGNPAVYSMVHVNGSSGHMGPMNMNPMPM
SGMPMGPDQKYC
>231Sapajus apella|A0A6J3G1H9|Mammalia|SRC3
MSGLGENLDPLATDSRKRKLPCDTPGQGLICSGEKRRREQESKYIEELAELISANLSDIDNFNVKPDKCAILKE
TVRQIRQIKEQGKTISSDDDVQKADVSSTGQGVIDKDSLGPLLLQALDGFLFVVNRDGNIVFVSENVTQYLQYK
QEDLVNTSVYNILHEEDRKDFLKNLPKSTVNGVSWTNESQRQKSHTFNCRMLMKTHDILEDINTSPEMRQRYET
MQCFALSQPRAMMEEGEDLQSCMICVARRITTGERVYPSNPESFITRHDLSGKVVNIDTNSLRSSMRPGFEDII
RRCIQRFFSLNDGQSWSQKRHYQEAYLNGHAETPVYRFSLADGTIVTAQTKSKLFRNPVTNDRHGFVSTHFLQR
EQNGYRPNPNPVGQGIRPPMAGCNSSVGGMSMSPNQGLQMPSSRAYGLADPSTTGQMSGARYGGSSNIASMTSG
PGMQSPSSYQNNNYGLNMSSPPHGSPGLAPNQQNIMISPRNRGSPKMASHQFSPVAGVHSPMGSSGSTGSHSFS
SSSLSALQAISEGVGTSLLSTLSSPGPKLDNSPNMNITQPSKVSSQDSKSPLGFYCDQNPVESSMCQSNSRDHL
SDKESKESSIEGSENQRGPLESKGHKKLLQLLTCSSDDRGHSSLTNSPLDSSCKESSVSVTSPSGVSSSTSGGV
SSTSSMHGSLLQEKHRILHKLLQNGNSPAEVAKITAEATGKDTSSITSCGDGNVVKQEQLSPKKKENNALLRYL
LDRDDPSDALSKELQPQVEGVDNKMSQCTSSTIPSSSQEKDPKIKTETSEEGSGDLDNLDAILGDLTSSDFYNN
SVSTNGSHLGTKQQIFQGTNSLGLKSPQSVQSIRPPYNRAVSLDSPVSVGSSPPVKNISAFPMLPKQPMLGGNP
RMMDSQENYGSSMGGPNRNVTVTQTPSSGDWGLPNSKASRMEPMNSNSMGRPGGDYNTSLPRAALGGSVPTLPL
RSNSIPGARPMLQPQQQQQQMLQMRPGEIPMGMGANPYGQAASNQLGSWSDGMLSMEQVPHGTQNRPLLRNSLD
DLVGPPSNLEGQSDERALLDQLHTLLSNTDATGLEEIDRALGIPELVNQGQALEPKQDAFQGQEAAVMMDQKAG
LYGQTYPAQGPPMQGGFHLQGQSPSFNSMMNQMNQQGSFPLQGMHPRANIMRPRTNTPKQLRMQLQQRLQGQQL
VSPQFLNQSRQALELKMENPTAGGAAVMRPMMQPQQGFLNAQMVAQRSRELLSHHFRQQRVAMMMQQQQQQQQQ
QQQQQQQQQQQQQTQAFSPPPNVTASPSMDGLLAGPAMPQAPPQQFTYQPNYGMGQPPDPAFGRVSSPPNAMMS

SRMGPSQNPMMQHPQAASMFQSSEMKGWPSGNLARNSSFSQQQFAHQGNPAVYSMVHVNGSSGHMGPMNMNPMP
MSGMPMGPDQKYC
>232Sapajus apella|A0A6J3G234|Mammalia|SRC3
MSGLGENLDPLATDSRKRKLPCDTPGQGLICSGEKRRREQESKYIEELAELISANLSDIDNFNVKPDKCAILKE
TVRQIRQIKEQGKTISSDDDVQKADVSSTGQGVIDKDSLGPLLLQALDGFLFVVNRDGNIVFVSENVTQYLQYK
QEDLVNTSVYNILHEEDRKDFLKNLPKSTVNGVSWTNESQRQKSHTFNCRMLMKTHDILEDINTSPEMRQRYET
MQCFALSQPRAMMEEGEDLQSCMICVARRITTGERVYPSNPESFITRHDLSGKVVNIDTNSLRSSMRPGFEDII
RRCIQRFFSLNDGQSWSQKRHYQEAYLNGHAETPVYRFSLADGTIVTAQTKSKLFRNPVTNDRHGFVSTHFLQR
EQNGYRPNPNPVGQGIRPPMAGCNSSVGGMSMSPNQGLQMPSSRAYGLADPSTTGQMSGARYGGSSNIASMTSG
PGMQSPSSYQNNNYGLNMSSPPHGSPGLAPNQQNIMISPRNRGSPKMASHQFSPVAGVHSPMGSSGSTGSHSFS
SSSLSALQAISEGVGTSLLSTLSSPGPKLDNSPNMNITQPSKVSSQDSKSPLGFYCDQNPVESSMCQSNSRDHL
SDKESKESSIEGSENQRGPLESKGHKKLLQLLTCSSDDRGHSSLTNSPLDSSCKESSVSVTSPSGVSSSTSGGV
SSTSSMHGSLLQEKHRILHKLLQNGNSPAEVAKITAEATGKDTSSITSCGDGNVVKQEQLSPKKKENNALLRYL
LDRDDPSDALSKELQPQVEGVDNKMSQCTSSTIPSSSQEKDPKIKTETSEEGSGDLDNLDAILGDLTSSDFYNN
SVSTNGSHLGTKQQIFQGTNSLGLKSPQSVQSIRPPYNRAVSLDSPVSVGSSPPVKNISAFPMLPKQPMLGGNP
RMMDSQENYGSSMGGPNRNVTVTQTPSSGDWGLPNSKASRMEPMNSNSMGRPGGDYNTSLPRAALGGSVPTLPL
RSNSIPGARPMLQPQQQQQQMLQMRPGEIPMGMGANPYGQAASNQLGSWSDGMLSMEQVPHGTQNRPLLRNSLD
DLVGPPSNLEGQSDERALLDQLHTLLSNTDATGLEEIDRALGIPELVNQGQALEPKQDAFQGQEAAVMMDQKAG
LYGQTYPAQGPPMQGGFHLQGQSPSFNSMMNQMNQQGSFPLQGMHPRANIMRPRTNTPKQLRMQLQQRLQGQQL
VSPQFLNQSRQALELKMENPTAGGAAVMRPMMQPQVSSQGFLNAQMVAQRSRELLSHHFRQQRVAMMMQQQQQQ
QQQQQQQQQQQQQQQQTQAFSPPPNVTASPSMDGLLAGPAMPQAPPQQFTYQPNYGMGQPPDPAFGRVSSPPNA
MMSSRMGPSQNPMMQHPQAASMFQSSEMKGWPSGNLARNSSFSQQQFAHQGNPAVYSMVHVNGSSGHMGPMNMN
PMPMSGMPMGPDQKYC
>233Sapajus apella|A0A6J3G238|Mammalia|SRC3
MSGLGENLDPLATDSRKRKLPCDTPGQGLICSGEKRRREQESKYIEELAELISANLSDIDNFNVKPDKCAILKE
TVRQIRQIKEQGKTISSDDDVQKADVSSTGQGVIDKDSLGPLLLQALDGFLFVVNRDGNIVFVSENVTQYLQYK
QEDLVNTSVYNILHEEDRKDFLKNLPKSTVNGVSWTNESQRQKSHTFNCRMLMKTHDILEDINTSPEMRQRYET
MQCFALSQPRAMMEEGEDLQSCMICVARRITTGERVYPSNPESFITRHDLSGKVVNIDTNSLRSSMRPGFEDII
RRCIQRFFSLNDGQSWSQKRHYQEAYLNGHAETPVYRFSLADGTIVTAQTKSKLFRNPVTNDRHGFVSTHFLQR
EQNGYRPNPNPVGQGIRPPMAGCNSSVGGMSMSPNQGLQMPSSRAYGLADPSTTGQMSGARYGGSSNIASMTSG
PGMQSPSSYQNNNYGLNMSSPPHGSPGLAPNQQNIMISPRNRGSPKMASHQFSPVAGVHSPMGSSGSTGSHSFS
SSSLSALQAISEGVGTSLLSTLSSPGPKLDNSPNMNITQPSKVSSQDSKSPLGFYCDQNPVESSMCQSNSRDHL
SDKESKESSIEGSENQRGPLESKGHKKLLQLLTCSSDDRGHSSLTNSPLDSSCKESSVSVTSPSGVSSSTSGGV
SSTSSMHGSLLQEKHRILHKLLQNGNSPAEVAKITAEATGKDTSSITSCGDGNVVKQEQLSPKKKENNALLRYL
LDRDDPSDALSKELQPQVEGVDNKMSQCTSSTIPSSSQEKDPKIKTETSEEGSGDLDNLDAILGDLTSSDFYNN
SVSTNGSHLGTKQQIFQGTNSLGLKSPQSVQSIRPPYNRAVSLDSPVSVGSSPPVKNISAFPMLPKQPMLGGNP
RMMDSQENYGSSMGGPNRNVTVTQTPSSGDWGLPNSKASRMEPMNSNSMGRPGGDYNTSLPRAALGGSVPTLPL
RSNSIPGARPMLQPQQQQQQMLQMRPGEIPMGMGANPYGQAASNQLGSWSDGMLSMEQVPHGTQNRPLLRNSLD
DLVGPPSNLEGQSDERALLDQLHTLLSNTDATGLEEIDRALGIPELVNQGQALEPKQDAFQGQEAAVMMDQKAG
LYGQTYPAQGPPMQGGFHLQGQSPSFNSMMNQMNQQGSFPLQGMHPRANIMRPRTNTPKQLRMQLQQRLQGQQF
LNQSRQALELKMENPTAGGAAVMRPMMQPQVSSQGFLNAQMVAQRSRELLSHHFRQQRVAMMMQQQQQQQQQQ
QQQQQQQQQQQTQAFSPPPNVTASPSMDGLLAGPAMPQAPPQQFTYQPNYGMGQPPDPAFGRVSSPPNAMMSSR
MGPSQNPMMQHPQAASMFQSSEMKGWPSGNLARNSSFSQQQFAHQGNPAVYSMVHVNGSSGHMGPMNMNPMPMS
GMPMGPDQKYC
>234Sapajus apella|A0A6J3G2U0|Mammalia|SRC3
MSGLGENLDPLATDSRKRKLPCDTPGQGLICSGEKRRREQESKYIEELAELISANLSDIDNFNVKPDKCAILKE
TVRQIRQIKEQGKTISSDDDVQKADVSSTGQGVIDKDSLGPLLLQALDGFLFVVNRDGNIVFVSENVTQYLQYK
QEDLVNTSVYNILHEEDRKDFLKNLPKSTVNGVSWTNESQRQKSHTFNCRMLMKTHDILEDINTSPEMRQRYET
MQCFALSQPRAMMEEGEDLQSCMICVARRITTGERVYPSNPESFITRHDLSGKVVNIDTNSLRSSMRPGFEDII
RRCIQRFFSLNDGQSWSQKRHYQEAYLNGHAETPVYRFSLADGTIVTAQTKSKLFRNPVTNDRHGFVSTHFLQR
EQNGYRPNPNPVGQGIRPPMAGCNSSVGGMSMSPNQGLQMPSSRAYGLADPSTTGQMSGARYGGSSNIASMTSG
PGMQSPSSYQNNNYGLNMSSPPHGSPGLAPNQQNIMISPRNRGSPKMASHQFSPVAGVHSPMGSSGSTGSHSFS
SSSLSALQAISEGVGTSLLSTLSSPGPKLDNSPNMNITQPSKVSSQDSKSPLGFYCDQNPVESSMCQSNSRDHL
SDKESKESSIEGSENQRGPLESKGHKKLLQLLTCSSDDRGHSSLTNSPLDSSCKESSVSVTSPSGVSSSTSGGV

SSTSSMHGSLLQEKHRILHKLLQNGNSPAEVAKITAEATGKDTSSITSCGDGNVVKQEQLSPKKKENNALLRYL
LDRDDPSDALSKELQPQVEGVDNKMSQCTSSTIPSSSQEKDPKIKTETSEEGSGDLDNLDAILGDLTSSDFYNN
SVSTNGSHLGTKQQIFQGTNSLGLKSPQSVQSIRPPYNRAVSLDSPVSVGSSPPVKNISAFPMLPKQPMLGGNP
RMMDSQENYGSSMGGPNRNVTVTQTPSSGDWGLPNSKASRMEPMNSNSMGRPGGDYNTSLPRAALGGSVPTLPL
RSNSIPGARPMLQPQQQQQQMLQMRPGEIPMGMGANPYGQAASNQLGSWSDGMLSMEQVPHGTQNRPLLRNSLD
DLVGPPSNLEGQSDERALLDQLHTLLSNTDATGLEEIDRALGIPELVNQGQALEPKQDAFQGQEAAVMMDQKAG
LYGQTYPAQGPPMQGGFHLQGQSPSFNSMMNQMNQQGSFPLQGMHPRANIMRPRTNTPKQLRMQLQQRLQGQQL
VSPQFLNQSRQALELKMENPTAGGAAVMRPMMQPQGFLNAQMVAQRSRELLSHHFRQQRVAMMMQQQQQQQQQQ
QQQQQQQQQQQQTQAFSPPPNVTASPSMDGLLAGPAMPQAPPQQFTYQPNYGMGQPPDPAFGRVSSPPNAMMSS
RMGPSQNPMMQHPQAASMFQSSEMKGWPSGNLARNSSFSQQQFAHQGNPAVYSMVHVNGSSGHMGPMNMNPMPM
SGMPMGPDQKYC

>235Sapajus apella|A0A6J3G3H1|Mammalia|SRC3
MSGLGENLDPLATDSRKRKLPCDTPGQGLICSGEKRRREQESKYIEELAELISANLSDIDNFNVKPDKCAILKE
TVRQIRQIKEQGKTISSDDDVQKADVSSTGQGVIDKDSLGPLLLQALDGFLFVVNRDGNIVFVSENVTQYLQYK
QEDLVNTSVYNILHEEDRKDFLKNLPKSTVNGVSWTNESQRQKSHTFNCRMLMKTHDILEDINTSPEMRQRYET
MQCFALSQPRAMMEEGEDLQSCMICVARRITTGERVYPSNPESFITRHDLSGKVVNIDTNSLRSSMRPGFEDII
RRCIQRFFSLNDGQSWSQKRHYQEAYLNGHAETPVYRFSLADGTIVTAQTKSKLFRNPVTNDRHGFVSTHFLQR
EQNGYRPNPNPVGQGIRPPMAGCNSSVGGMSMSPNQGLQMPSSRAYGLADPSTTGQMSGARYGGSSNIASMTSG
PGMQSPSSYQNNNYGLNMSSPPHGSPGLAPNQQNIMISPRNRGSPKMASHQFSPVAGVHSPMGSSGSTGSHSFS
SSSLSALQAISEGVGTSLLSTLSSPGPKLDNSPNMNITQPSKVSSQDSKSPLGFYCDQNPVESSMCQSNSRDHL
SDKESKESSIEGSENQRGPLESKGHKKLLQLLTCSSDDRGHSSLTNSPLDSSCKESSVSVTSPSGVSSSTSGGV
SSTSSMHGSLLQEKHRILHKLLQNGNSPAEVAKITAEATGKDTSSITSCGDGNVVKQEQLSPKKKENNALLRYL
LDRDDPSDALSKELQPQVEGVDNKMSQCTSSTIPSSSQEKDPKIKTETSEEGSGDLDNLDAILGDLTSSDFYNN
SVSTNGSHLGTKQQIFQGTNSLGLKSPQSVQSIRPPYNRAVSLDSPVSVGSSPPVKNISAFPMLPKQPMLGGNP
RMMDSQENYGSSMGGPNRNVTVTQTPSSGDWGLPNSKASRMEPMNSNSMGRPGGDYNTSLPRAALGGSVPTLPL
RSNSIPGARPMLQPQQQQQQMLQMRPGEIPMGMGANPYGQAASNQLGSWSDGMLSMEQVPHGTQNRPLLRNSLD
DLVGPPSNLEGQSDERALLDQLHTLLSNTDATGLEEIDRALGIPELVNQGQALEPKQDAFQGQEAAVMMDQKAG
LYGQTYPAQGPPMQGGFHLQGQSPSFNSMMNQMNQQGSFPLQGMHPRANIMRPRTNTPKQLRMQLQQRLQGQQF
LNQSRQALELKMENPTAGGAAVMRPMMQPQGFLNAQMVAQRSRELLSHHFRQQRVAMMMQQQQQQQQQQQQQQQ
QQQQQQQTQAFSPPPNVTASPSMDGLLAGPAMPQAPPQQFTYQPNYGMGQPPDPAFGRVSSPPNAMMSSRMGPS
QNPMMQHPQAASMFQSSEMKGWPSGNLARNSSFSQQQFAHQGNPAVYSMVHVNGSSGHMGPMNMNPMPMSGMPM
GPDQKYC
>236Esox lucius|A0A6Q2YP93|Actinopteri|SRC3
MSGVGDNSLEPMCSERKRKISTCDTPGLGCDKRRREQESKYIEELAELISANLGDIDSFNVKPDKCAILKETVR
QIRQIKEQGKSSCSDDDVQKADVSSTGQGVIDKDHLGPLLLQALDGFLFVVNREGSIVFVSDNVTQYLQYTQEE
LINTSVYNILHEDDREELHKNLPKNTAPNGGPWGGEASRQKSNTFNCRMLVKYGHGQSHQNHRPSEEGPNRQRY
ETMQCFALTQPRAMMEEGEDLQSCMICVARCITAVERTERFSTRHELSGKLIEIEQQSSLHTTMRPGWEDLVRR
CMQMFLYLSEGQPWFYKRHYKEALMHGRAETPSYRFSLSDGTLVTAQTRSELCRNQGSNEQHTFLSTHLLQREP
NGYRANQGGMMRPQGLGGTNAPMNMAPGGGMAGMNRSYGMGVEPGHMAQRGGHPYTGGNRMHAVNQMNQSAPFH
GTSYGVGGVSGPSQGGPGMNGPQNIMGSLRVRGSPKMAVGPFSPGGMHSPMTSGHPGSTGGTTSFSSSSLNALQ
AISEGVGSSIPSALPSALPSALTSPPTHKPDSSPGINSSQQGQSKPGGSDSKSPGSSLANGGEHPHTPTGEGIP
DRPDSQASREGPARGEASRRDNKGGHKKLLQLLTSPTEELGPPNPQAGPGHAPMGSEAKEGAGGTTSPSSTGVS
SSTAAAAHLANQSLQEKHKILHKLLQNGNTPDEVARITAEATGKVTDGGVPEAGQGGPGARGAEVKQEQHSPKK
EKAHALLHYLLNKDDSKAGRGDPKPKPEDLDSRGTQGVCSGITISEPGNMVGKVKTEPPDEVGRTLYLVLITLL
FSLGAACDVCYSAVDGERGPMLPAQRGPLQRALSVDAKPLGGGGGLAGRRNVTCPTLIKQEKVERPPMAGQADW
GMPRSTGSPVGVQGHPTLSRPGMDFNSKGMMGGPMIGRSNSVPGNTRSMLSQHLEMGSGEVNMGMSPFSGQGPP
PQSPSWPDSVGLQKDFRWMIMRQFGNPLDELLVPPSTSQGQSDELALLDQLDCLLNNTDVIALEEIDRALGIPD
LVGQRPPSEPFPGPGPDTPMGMEPKPMYGHGYPGPPSMQSNYGGNPMQAQSPAGFNSVMNQMGGQTAPGGFPGM
GAMGHPRAGLMRPRLMGPANPLRQQLQQRLQGQQFMNQTRQSMGIKMENSPAGNPGMRPGIQPSMGSQPGYLNS
QMMAQRSREMMTLQIRRQRMMMMMQQQTAAGGFSPPPNVTAPTGMDNPMTGPPMPGPPQFPYAGNYGMSQQGEP
SFVGAGSSPPSNMMPGRMGGPQNAMMHPQSAPMYQSADMKGWPQSGMTRNSSYPQQQFTQQGNTGQFGGPMMMN
GSMGGPSPGQLAMNSMGMGRMPMGPEQKYC
>237Pipistrellus kuhlii|A0A7J7Y9M3|Mammalia|SRC3

MSGLGENSLDPLVPDSRKRKLPCDTPGQGLTCSGEKRRREQESKYIEELAELISANLSDIDNFNVKPDKCAILK
ETVRQIRQIKEQGKAISNDDDVQKADVSSTGQGVIDKDSLGPLLLQALDGFLFVVNRDGNIVFVSENVTQYLQY
KQEDLVNTSVYNILHEEDRKDFLKNLPKSTVNGVTWTNETQRQKSHTFNCRMLMKAPHDIVEDMNTSPEMRQRY
ETMQCFALSQPRAMMEEGEDLQSCMICVARRITTGERAFASNPESFITRHDLTGKLVNIDTNSLRSSMRPGFED
IIRRCIQRFFSLNDGQSWSQKRHYQEAYIHGHAETPVYRFSLADGTIVTAQTKSKLFRNPVTNDRHGFVSTHFL
QREQNGYRQNPNPVGQGIRPPVAGCNSVGGMSMSPNQGLQMLNTRAYGLPDPGTTGQMSGVRYGASSNIATMNP
GPAMQSPSSYQNNNYGLNMSSPPHGSPGLGSSQQNIMISPRNRGSPKMASHQFSPVAGVHSPIGSSGNTGSHSF
SSSSLSALQAISEGVGTSLLSTLSSPGPKLDNSPNMNITQPSKVNSQDSKSPLSLYCDQNPVESSMCQSNSRDH
LNDKDSKENSVEGSENQRGPLESKGHKKLLQLLTCSSDDRGHSSLTNSPLDSSCKDSSISVTSPSGVSSSTSGG
VSSTSNMHGSLLQEKHRILHKLLQNGNSPAEVAKITAEATGKDTSSATFCVEGNVKQEQLSPKKKENNALLRYL
LDRDDPSDTLSKEVQPKVEGVDNKMSQCSSSTVPSSSQEKDAKIKTEANEEGSGDLDNLDAILGDLTSSDFYNN
SVSTNGSNLGNKQQMFQGTNSLGLRSPQSVQSIRPPYSRAASLDSPVSVGSSPPVKNVTAFPMLPKQPLLGGNP
IMMDSQENYGSNMGGPNRNVTVNQTPPSGNWGLSNSKASRMESMSSNSMGRPGADYNTALPRPTMGGSMSTLPL
RSNSIPGARPILQQQQQQLQPQPQQQQQQQQQHQQMLQMRPGEIPMGMGVSPYGPAAPSHQPGSWPDGMLSMEQ
GPHGTQNRPLFRNSLDDLLGPPSNLEGQNDERALLDQLHTLLSNTDATGLEEIDRALGIPELVNQGQALEPKQD
VFQGQEAPVMMDQKAALYGQTYPAQGPPMQGSFNLQGQSPSFNSMMSQMNQQGSFPLQGMHPRANMMRPRTNNP
KQLRMQLQQRLQGHQFLNQSRQALEMKMENPTAGAAAMMRPMMQPQQGFLNAQMVAQRSRELLSHHFRQQRVAA
MMMQQQQQQQQQQQQQTQAFSPPPNVTASPSMDGVLAGPAMPQAPPQQFPYQPNYGMGQQPDPAFGRVSSPPNA
MMSSRMGPSQNPMMQHPQTAPMYQSSEMKGWPSGNLARSSSFPQQQYAHQGNPAAYNMVHMNGSSGPMGQMNMN
SMPMSGMPMGPEQKYC
>238Pipistrellus kuhlii|A0A7J7Y9N1|Mammalia|SRC3
MSGLGENSLDPLVPDSRKRKLPCDTPGQGLTCSGEKRRREQESKYIEELAELISANLSDIDNFNVKPDKCAILK
ETVRQIRQIKEQGKAISNDDDVQKADVSSTGQGVIDKDSLGPLLLQALDGFLFVVNRDGNIVFVSENVTQYLQY
KQEDLVNTSVYNILHEEDRKDFLKNLPKSTVNGVTWTNETQRQKSHTFNCRMLMKAPHDIVEDMNTSPEMRQRY
ETMQCFALSQPRAMMEEGEDLQSCMICVARRITTGERAFASNPESFITRHDLTGKLVNIDTNSLRSSMRPGFED
IIRRCIQRFFSLNDGQSWSQKRHYQEAYIHGHAETPVYRFSLADGTIVTAQTKSKLFRNPVTNDRHGFVSTHFL
QREQNGYRQNPNPVGQGIRPPVAGCNSVGGMSMSPNQGLQMLNTRAYGLPDPGTTGQMSGVRYGASSNIATMNP
GPAMQSPSSYQNNNYGLNMSSPPHGSPGLGSSQQNIMISPRNRGSPKMASHQFSPVAGVHSPIGSSGNTGSHSF
SSSSLSALQAISEGVGTSLLSTLSSPGPKLDNSPNMNITQPSKVNSQDSKSPLSLYCDQNPVESSMCQSNSRDH
LNDKDSKENSVEGSENQRGPLESKGHKKLLQLLTCSSDDRGHSSLTNSPLDSSCKDSSISVTSPSGVSSSTSGG
VSSTSNMHGSLLQEKHRILHKLLQNGNSPAEVAKITAEATGKDTSSATFCVEGNVKQEQLSPKKKENNALLRYL
LDRDDPSDTLSKEVQPKVEGVDNKMSQCSSSTVPSSSQEKDAKIKTEANEEGSGDLDNLDAILGDLTSSDFYNN
SVSTNGSNLGNKQQMFQGTNSLGLRSPQSVQSIRPPYSRAASLDSPVSVGSSPPVKNVTAFPMLPKQPLLGGNP
IMMDSQENYGSNMGGPNRNVTVNQTPPSGNWGLSNSKASRMESMSSNSMGRPGADYNTALPRPTMGGSMSTLPL
RSNSIPGARPILQQQQQQLQPQPQQQQQQQQQHQQMLQMRPGEIPMGMGVSPYGPAAPSHQPGSWPDGMLSMEQ
GPHGTQNRPLFRNSLDDLLGPPSNLEGQNDERALLDQLHTLLSNTDATGLEEIDRALGIPELVNQGQALEPKQD
VFQGQEAPVMMDQKAALYGQTYPAQGPPMQGSFNLQGQSPSFNSMMSQMNQQGSFPLQGMHPRANMMRPRTNNP
KQLRMQLQQRLQGHQFLNQSRQALEMKMENPTAGAAAMMRPMMQPQVNSQGFLNAQMVAQRSRELLSHHFRQQR
VAAMMMQQQQQQQQQQQQQTQAFSPPPNVTASPSMDGVLAGPAMPQAPPQQFPYQPNYGMGQQPDPAFGRVSSP
PNAMMSSRMGPSQNPMMQHPQTAPMYQSSEMKGWPSGNLARSSSFPQQQYAHQGNPAAYNMVHMNGSSGPMGQM
NMNSMPMSGMPMGPEQKYC

>239Pipistrellus kuhlii|A0A7J7YA00|Mammalia|SRC3
MSGLGENSLDPLVPDSRKRKLPCDTPGQGLTCSGEKRRREQESKYIEELAELISANLSDIDNFNVKPDKCAILK
ETVRQIRQIKEQGKAISNDDDVQKADVSSTGQGVIDKDSLGPLLLQALDGFLFVVNRDGNIVFVSENVTQYLQY
KQEDLVNTSVYNILHEEDRKDFLKNLPKSTVNGVTWTNETQRQKSHTFNCRMLMKAPHDIVEDMNTSPEMRQRY
ETMQCFALSQPRAMMEEGEDLQSCMICVARRITTGERAFASNPESFITRHDLTGKLVNIDTNSLRSSMRPGFED
IIRRCIQRFFSLNDGQSWSQKRHYQEVISNRTFSPTAYIHGHAETPVYRFSLADGTIVTAQTKSKLFRNPVTND
RHGFVSTHFLQREQNGYRQNPNPVGQGIRPPVAGCNSVGGMSMSPNQGLQMLNTRAYGLPDPGTTGQMSGVRYG
ASSNIATMNPGPAMQSPSSYQNNNYGLNMSSPPHGSPGLGSSQQNIMISPRNRGSPKMASHQFSPVAGVHSPIG
SSGNTGSHSFSSSSLSALQAISEGVGTSLLSTLSSPGPKLDNSPNMNITQPSKVNSQDSKSPLSLYCDQNPVES
SMCQSNSRDHLNDKDSKENSVEGSENQRGPLESKGHKKLLQLLTCSSDDRGHSSLTNSPLDSSCKDSSISVTSP
SGVSSSTSGGVSSTSNMHGSLLQEKHRILHKLLQNGNSPAEVAKITAEATGKDTSSATFCVEGNVKQEQLSPKK
KENNALLRYLLDRDDPSDTLSKEVQPKVEGVDNKMSQCSSSTVPSSSQEKDAKIKTEANEEGSGDLDNLDAILG

DLTSSDFYNNSVSTNGSNLGNKQQMFQGTNSLGLRSPQSVQSIRPPYSRAASLDSPVSVGSSPPVKNVTAFPML
PKQPLLGGNPIMMDSQENYGSNMGGPNRNVTVNQTPPSGNWGLSNSKASRMESMSSNSMGRPGADYNTALPRPT
MGGSMSTLPLRSNSIPGARPILQQQQQQLQPQPQQQQQQQQQHQQMLQMRPGEIPMGMGVSPYGPAAPSHQPGS
WPDGMLSMEQGPHGTQNRPLFRNSLDDLLGPPSNLEGQNDERALLDQLHTLLSNTDATGLEEIDRALGIPELVN
QGQALEPKQDVFQGQEAPVMMDQKAALYGQTYPAQGPPMQGSFNLQGQSPSFNSMMSQMNQQGSFPLQGMHPRA
NMMRPRTNNPKQLRMQLQQRLQGHQFLNQSRQALEMKMENPTAGAAAMMRPMMQPQQGFLNAQMVAQRSRELLS
HHFRQQRVAAMMMQQQQQQQQQQQQQTQAFSPPPNVTASPSMDGVLAGPAMPQAPPQQFPYQPNYGMGQQPDPA
FGRVSSPPNAMMSSRMGPSQNPMMQHPQTAPMYQSSEMKGWPSGNLARSSSFPQQQYAHQGNPAAYNMVHMNGS
SGPMGQMNMNSMPMSGMPMGPEQKYC
>240Pipistrellus kuhlii|A0A7J7YAK1|Mammalia|SRC3
MSGLGENSLDPLVPDSRKRKLPCDTPGQGLTCSGEKRRREQESKYIEELAELISANLSDIDNFNVKPDKCAILK
ETVRQIRQIKEQGKAISNDDDVQKADVSSTGQGVIDKDSLGPLLLQALDGFLFVVNRDGNIVFVSENVTQYLQY
KQEDLVNTSVYNILHEEDRKDFLKNLPKSTVNGVTWTNETQRQKSHTFNCRMLMKAPHDIVEDMNTSPEMRQRY
ETMQCFALSQPRAMMEEGEDLQSCMICVARRITTGERAFASNPESFITRHDLTGKLVNIDTNSLRSSMRPGFED
IIRRCIQRFFSLNDGQSWSQKRHYQEAYIHGHAETPVYRFSLADGTIVTAQTKSKLFRNPVTNDRHGFVSTHFL
QREQNGYRQNPNPVGQGIRPPVAGCNSVGGMSMSPNQGLQMLNTRAYGLPDPGTTGQMSGVRYGASSNIATMNP
GPAMQSPSSYQNNNYGLNMSSPPHGSPGLGSSQQNIMISPRNRGSPKMASHQFSPVAGVHSPIGSSGNTGSHSF
SSSSLSALQAISEGVGTSLLSTLSSPGPKLDNSPNMNITQPSKVNSQDSKSPLSLYCDQNPVESSMCQSNSRDH
LNDKDSKENSVEGSENQRGPLESKGHKKLLQLLTCSSDDRGHSSLTNSPLDSSCKDSSISVTSPSGVSSSTSGG
VSSTSNMHGSLLQEKHRILHKLLQNGNSPAEVAKITAEATGKDTSSATFCVEGNVKQEQLSPKKKENNALLRYL
LDRDDPSDTLSKEVQPKVEGVDNKMSQCSSSTVPSSSQEKDAKIKTEANEEGSGDLDNLDAILGDLTSSDFYNN
SVSTNGSNLGNKQQMFQGTNSLGLRSPQSVQSIRPPYSRAASLDSPVSVGSSPPVKNVTAFPMLPKQPLLGGNP
IMMDSQENYGSNMGGPNRNVTVNQTPPSGNWGLSNSKASRMESMSSNSMGRPGADYNTALPRPTMGGSMSTLPL
RSNSIPGARPILQQQQQQLQPQPQQQQQQQQQHQQMLQMRPGEIPMGMGVSPYGPAAPSHQPGSWPDGMLSMEQ
GPHGTQNRPLFRNSLDDLLGPPSNLEGQNDERALLDQLHTLLSNTDATGLEEIDRALGIPELVNQGQALEPKQD
VFQGQEAPVMMDQKAALYGQTYPAQGPPMQGSFNLQGQSPSFNSMMSQMNQQGSFPLQGMHPRANMMRPRTNNP
KQLRMQLQQRLQGHQFLNQSRQALEMKMENPTAGAAAMMRPMMQPQVNSQQGFLNAQMVAQRSRELLSHHFRQQ
RVAAMMMQQQQQQQQQQQQQTQAFSPPPNVTASPSMDGVLAGPAMPQAPPQQFPYQPNYGMGQQPDPAFGRVSS
PPNAMMSSRMGPSQNPMMQHPQTAPMYQSSEMKGWPSGNLARSSSFPQQQYAHQGNPAAYNMVHMNGSSGPMGQ
MNMNSMPMSGMPMGPEQKYC
>241Pipistrellus kuhlii|A0A7J7YAZ1|Mammalia|SRC3
MSGLGENSLDPLVPDSRKRKLPCDTPGQGLTCSGEKRRREQESKYIEELAELISANLSDIDNFNVKPDKCAILK
ETVRQIRQIKEQGKAISNDDDVQKADVSSTGQGVIDKDSLGPLLLQALDGFLFVVNRDGNIVFVSENVTQYLQY
KQEDLVNTSVYNILHEEDRKDFLKNLPKSTVNGVTWTNETQRQKSHTFNCRMLMKAPHDIVEDMNTSPEMRQRY
ETMQCFALSQPRAMMEEGEDLQSCMICVARRITTGERAFASNPESFITRHDLTGKLVNIDTNSLRSSMRPGFED
IIRRCIQRFFSLNDGQSWSQKRHYQEAYIHGHAETPVYRFSLADGTIVTAQTKSKLFRNPVTNDRHGFVSTHFL
QREQNGYRQNPNPVGQGIRPPVAGCNSVGGMSMSPNQGLQMLNTRAYGLPDPGTTGQMSGVRYGASSNIATMNP
GPAMQSPSSYQNNNYGLNMSSPPHGSPGLGSSQQNIMISPRNRGSPKMASHQFSPVAGVHSPIGSSGNTGSHSF
SSSSLSALQAISEGVGTSLLSTLSSPGPKLDNSPNMNITQPSKVNSQDSKSPLSLYCDQNPVESSMCQSNSRDH
LNDKDSKENSVEGSENQRGPLESKGHKKLLQLLTCSSDDRGHSSLTNSPLDSSCKDSSISVTSPSGVSSSTSGG
VSSTSNMHGSLLQEKHRILHKLLQNGNSPAEVAKITAEATGKDTSSATFCVEGNVKQEQLSPKKKENNALLRYL
LDRDDPSDTLSKEVQPKVEGVDNKMSQCSSSTVPSSSQEKDAKIKTEANEEGSGDLDNLDAILGDLTSSDFYNN
SVSTNGSNLGNKQQMFQGTNSLGLRSPQSVQSIRPPYSRAASLDSPVSVGSSPPVKNVTAFPMLPKQPLLGGNP
IMMDSQENYGSNMGGPNRNVTVNQTPPSGNWGLSNSKASRMESMSSNSMGRPGADYNTALPRPTMGGSMSTLPL
RSNSIPGARPILQQQQQQLQPQPQQQQQQQQQHQQMLQMRPGEIPMGMGVSPYGPAAPSHQPGSWPDGMLSMEQ
GPHGTQNRPLFRNSLDDLLGPPSNLEGQNDERALLDQLHTLLSNTDATGLEEIDRALGIPELVNQGQALEPKQD
VFQGQEAPVMMDQKAALYGQTYPAQGPPMQGSFNLQGQSPSFNSMMSQMNQQGSFPLQGMHPRANMMRPRTNNP
KQLRMQLQQRLQGHQFLNQSRQALEMKMENPTAGAAAMMRPMMQPQGFLNAQMVAQRSRELLSHHFRQQRVAAM
MMQQQQQQQQQQQQQTQAFSPPPNVTASPSMDGVLAGPAMPQAPPQQFPYQPNYGMGQQPDPAFGRVSSPPNAM
MSSRMGPSQNPMMQHPQTAPMYQSSEMKGWPSGNLARSSSFPQQQYAHQGNPAAYNMVHMNGSSGPMGQMNMNS
MPMSGMPMGPEQKYC
>242Neopipo cinnamomea|A0A7K4QQE5|Aves|SRC3
CVVTMSGLGENSLDSLTNESRKRKPLPCDAPGASLTCSGEKRRREQESKYIEELAELISANLSDIDNFNVKPDK
CAILKETVRQIRQIKDQGKAVSNDDDVQKADVSSTGQGVIDKDSLGPLLLQAALDGFLFVVNRDGNIVFVSENV

TQYLQYKQEDLVNTSVYGILHEEDRKDFLKNLPKSSVNGVAWTSETPRQKSHTFNCRMMVKSSHDLLEDVGSHQ
DGRQRYETMQCFALSQPRAMMEEGEDLQSCMICVARRITTAERVFPTNPESFITRHDLSGKLVNIDTNSLRSSM
RPGFEDIIRRCIQRFLCHNDGQSWSNKRHYHEAYTQGHAETPLYRFSLADGTIVTAQTKSKLFRNPVTNDRHGF
VSTHFLQREQNGYRPNPNSVGQNVRTPVAGNTNSVGGLMNMSPGQGMAVQNRNYGMGDPSAVGQLSSTRYGGPG
NVGPVSSGPGMQSSAYQNSSYGLNISSPPHGSPGLTSNQQNLMISPRNRGSPKLSSHQYSPVTGMHSPMGSASN
TGNSTFSSSSLSALQAISEGVGNSLLSTLSSPGPKLDNSPNMSVTPQSKTSTQDSKSPSGLYCEQNQVESSICQ
SNSRDLLGEKESKEGNLEASDSQRGPSESKGHKKLLQLLTCSSDERGHSTASNSPLDSNCKEPSTSVTSPSGVS
SSTSGGVSSSSNVHGSLLQEKHRILHKLLQNGNSPAEVAKITAEATGKDMYHDAGNTTSCGESTVKQEQLSPKK
KENNALLRYLLDKDDVKDPLSKELKPKVESVDNKMGQCTSSAIPTSSQDKEMKIKMEPAEEMNGDLDNLDAILG
DLTSSEFYNNAMSANGNNLGTKQALFQGNALLGVKSSQSVQAARPPFNRAMSLDSPVGSSASVRNVNAFSMLQK
QNLMGGSPRMIENQENFGANLGFAPSRNVATNQHHPDWGLPSSKGSRLEGAGSAGPGRAGAESSAALPRPAGAV
PALPLRSSSLPAARPGLQQPLLHIRPNEINIGMAGNPYGQPGPSNQPGLWPDNVLSVEQASRGSQNRQLVRNCL
DDLLCSAPNMEGQNDERALLDQLHTLLSNTDVTGLEEIDRALGIPDLVSQGQALEPKQDSFQGQESSVLIDQKP
SLYAQPYQGQGAAMPGGFSNIQGQQPSFNSVMNQMGQPSNFPLQSVHPRANVMRPRTNTPKQLRMQLQQRLQGQ
QFMNQTRQTLEMKMESPVSGNTSAMRPVMQPQVGSQQQGFLNAQMVAQRNRELISHHFRQQRMAMMMQQQQQPQ
AFSPPPNVTATGSMDSALTGPPMAQVPPQQFPYPPNYGLSQQPDPAFSRVSSPPNPMISSRIGPSQNPVMQHPQ
PATMYQSSEMKGWPSGSMARNSSFPQQQFSHQGNAAAYSMMHMNGSSGHMGQMSINAMPMSGMPMGPDQVSS
>243Tachuris rubrigastra|A0A7K4WF23|Aves|SRC3
CMVTMSGLGENSLDSLTNESRKRKPLPCDTPGASLTCSGEKRRREQESKYIEELAELISANLSDIDNFNVKPDK
CAILKETVRQIRQIKDQGKAVSNDDDVQKADVSSTGQGVIDKDSLGPLLLQAALDGFLFVVNRDGNIVFVSENV
TQYLQYKQEDLVNTSVYGILHEEDRKDFLKNLPKSSVNGVAWTSETPRQKSHTFNCRMMVKSSHDLLEDVGSHQ
DGRQRYETMQCFALSQPRAMMEEGEDLQSCMICVARRITTAERVFPTNPESFITRHDLSGKLVNIDTNSLRSSM
RPGFEDIIRRCIQRFLCHNDGQSWSNKRHYHEAYTQGHAETPLYRFSLADGTIVTAQTKSKLFRNPVTNDRHGF
VSTHFLQREQNGYRPNPNSVGQNIRTPVAGNTNSVGGLMNMSPGQGMPVQNRNYGMGDPSAVGQLSSTRYGGPG
NMGPVTSGAGMQSSAYQNNSYGLNISSPPHGSPGLTSNQQNLMISPRNRGSPKMSSHQYSPVTGMHSPMGSASN
ASNSTFSSSSLSALQAISEGVGNSLLSTLSSPGPKLDNSPNMSVTPQSKTGTQDSKSPSGLYCEQNQVESSVCQ
SNSRDLLSEKESKEGNLEASESQRGPSESKGHKKLLQLLTCSSDERGHSTASNSPLDSNCKEPSTSVTSPSGVS
SSTSGGVSSSSNVHGSLLQEKHRILHKLLQNGNSPAEVAKITAEATGKDTYHDAGNTTSCGEGTVKQEQLSPKK
KENNALLRYLLDKDDVKDPLSKELKPKVESVDNKMGQCTSSTIPTSSQDKEMKIKMEPAEEMNGDLDNLDAILG
DLTSSEFYNNAMSANGNNLGTKQALFQGNALLGVKSSQSVQNARPPYNRAMSLDSPVGSSASVRNVNAFSMLQK
QNLMGGSPRMIENQDNFGANLAGAPNRNVAMNQHQPDWALPSSKGSRLEPAGSAGLVRAGAELSAALPRPAGSV
PALPLRSNSLPAARPGLQQPLLHIRPNEMNIGMAGNPYGQPGPSNQPGLWPDNVLSMEQASRGSQNRQLVRNSL
DDLLCSAPNMEGQNDERALLDQLHTLLSNTDVTGLEEIDRALGIPDLVSQGQALEPKQDSFQGQESSIMIDQKP
SLYAQPYQGQGATMPGGFSNIQGQQPSFNSVMNQMGQASNFPLQSVHPRANVMRPRTNTPKQLRMQLQQRLQGQ
QFMNQTRQTLEMKMESPVSGNTSAMRPVMQPQVGSQQQGFLNAQMVAQRNRELISHHFRQQRMAMMMQQQQQPQ
AFSPPPNVTATGSMDSALTGPPMAQVPPQQFPYPPNYGLSQQPDPAFSRVSSPPNPMISSRIGPSQNPVMQHPQ
PATMYQSSEMKGWPSGSMARNSSFSQQQFSHQGNPAAYSMMHMNGSSGHMGQMSINAMPMSGMPMGPDQVSC
>244Bucorvus abyssinicus|A0A7K4YJW2|Aves|SRC3
CMVKMSGLGENSLDSLTNESRKRKPLPCDTPGASLTCSGEKRRREQESKYIEELAELISANLSDIDNFNVKPDK
CAILKETVRQIRQIKEQGKAVSNDDDVQKADVSSTGQGVIDKDSLGPLLLQAALDGFLFVVNRDGNIVFVSENV
AQYLQYKQEDLVNTSVYGILHEEDRKDFLKNLPKSSVNGVAWSNETPRQKSHTFNCRMMVKTSHDLLEDVGSHQ
DIRQRYETMQCFALSQPRAMMEEGEDLQSCMICVARRITTAERVFPTNPESFITRHDLSGKLVNIDTNSLRSSM
RPGFEDIIRRCIQRFLCHNDGQSWSNKRHYHEAYTQGHAETPLYRFSLADGTVVTAQTKSKLFRNPVTNDRHGF
VSTHFLQREQNGYRPNPNSVGQNIRTPVAGNTNSVGGLMNMSPGQGMPMQNRSYGMGDPNAVGQLSSTRYGGPG
NMGPVNSGPGMQSSAYQNNSYGLNISSPPHGSPGLTSNQQNLMISPRNRGSPKMSSHQFSPVTGMHSPMGSASN
TSNSTFSSSSLSALQAISEGVGTSLLSTLSSPGPKLDNSPNMSIAQQSKTSTQDSKSPSGLYCEQNQVESSICQ
SNSRELLSEKESKEGNLEASESQRGPSESKGHKKLLQLLTCSSDERGHSTASNSPLDSNCKESSTNVTSPSGVS
SSTPGGVSSSSNMHGSLLQEKHRILHKLLQNGNSPAEVAKITAEATGKDTYHDASNTAPCGEGTVKQEQLSPKK
KENNALLRYLLDKDDVKDPLSKELKPKVESVDNKMGQCSSSTIPTSSQEKEMKIKTEPVEEMSGDLDNLDAILG
DLTSSEFYSNAMSANGNNLGTKQALFQGNAILGMRSPQAVQTTRPPFNRAMSLDSPMGSSAPVRNVNAFSMLQK
QNLMGGSPRMIENQENFGANLAGAPNRNVAMNQHQSGDWALPNSKVNRLEPTSSASMLRPGPEFSASLPRPTVG
GSMSGLPVRSNSIPGTRPMLQQQMIHMRPNEINIGIGGNPYGQPGPSNQPGSWPDNMLSMEQASRGSQNRQLVR
NSLDDLLCPAPNMEGQSDERALLDQLHTLLSNTDVTGLEEIDRALGIPDLVNQGQALEPKQDSFQGQESSVMMD
QKTSLYGQPYQGQGAAMPGGFSNIQGQQPSFNSVMNQMSQQSNFPLQSMHPRANVMRPRTNTPKQLRMQLQQRL

QGQQFMNQTRQALEMKMENPVSGNTSVMRPVMQPQVGSQQQGFLNAQMVAQRNRELISHHFRQQRMAMMMQQQQ
QPQAFSPPPNVTASGSMDSALTGPPMAQVPPQQFPYPPNYGISQQPDPAFSRVSSPPNPMMSSRIGPSQNPMMQ
HPQTATMYQSPEMKGWPSGSMARSSSFPQQQFSHQGNPAAYSMMHMNGSSGHMGQMNINTIPMSGMPMGPDQVS
C
>245Centropus unirufus|A0A7K4ZYQ5|Aves|SRC3
CVDKMSGLGENSLDSLTNESRKRKPLPCDTPGASLTCSGEKRRREQESKYIEELAELISANLSDIDNFNVKPDK
CAILKETVRQIRQIKEQGKAVSNDDDVQKADVSSTGQGVIDKDSLGPLLLQAALDGFLFVVNRDGNIVFVSENV
TQYLQYKQEDLVNTSVYGILHEEDRKDFLKNLPKSSVNGVTWTNEAPRQKSHTFNCRMMVKTSHDLLEDVGSHQ
DVRQRYETMQCFALSQPRAMMEEGEDLQSCMICVARRITTAERVFPANPESFITRHDLSGKLVNIDTNSLRSSM
RPGFEDIIRRCIQRFLCHSDGQSWSNKRHYHEAYTQGHAETPLYRFSLADGTIVTAQTKSKLFRNPVTNDRHGF
VSTHFLQREQNGYRPTPTAVGQTIRTPVAGNTNSAGGLMNMSPGQGMAVQNRTYGMGDANAVGQLSSARYGGPG
SLGTVNSVPGMQTSAYQNNSYGLNINSPPHGSPGLTSNQQNLMISPRNRGSPKMTSHQFSPVTGMHSPMGSASN
TGNSTFSSSSLSALQAISEGVGTSLLSTLSSPGPKLDNSPNMSIAQQSKTNTQDSKSPSGLYCEQNQVEGSICQ
SNGRDVLSEKDSKEGNLEAPESQRGPSESKGHKKLLQLLTCSSDERGHSTASNSPLDSNCKESSTNVTSPSGVS
SSTSGGVSSSSNMHGSLLQEKHRILHKLLQNGNSPAEVAKITAEATGKDTYHDASNTAPCGDGTVKQEQLSPKK
KENNALLRYLLDKDDVKDPLSKELKPKVESVDNKMGQCSSSTIPTSSQEKEMKIKTEPPEEMSGDLDNLDAILG
DLTSSEFYNNAMSANGNNLGTKQALFQGNALLGMRSPQAMQAARPPFTRAMSLDSPMGSGGPLRNINTFSMLQK
QNLMGESPRMIENQDNFGANLGFAPNRNVVMNQHQAGDWGLPNSKVTRLEPTSSASMLRPGAEFSASLPRPPVA
GLPVRSNSVPGTRPMLQQQMIHMRPNEINISMGGNPYGQPGPSNQPGSWPDNMLSMEQASRGSQNRQLVRNSLD
DLLCPAPSMEGQNDERALLDQLHTLLSNTDVTGLEEIDRALGIPDLVNQGQGLEPKQDSFQGQESSVMIDQKSA
LYGQPYHGQGAAMPGGFSNMQGQQPSFNSVMNQMSQQSSFPLQSMHPRANVMRPRTNTPKQLRMQLQQRLQGQQ
FVNQSRQALEMKMENPVGGNASVMRPVMQPQVGSQQQGFLNAQMVAQRNRELISHHFRQQRMAMMMQQQQQPQA
FSPPPNVTASGSMDGALTGPPMTQVPPQQFPYPPNYGISQQPDPAFSRVPSPPNPMLSSRIGPSQNPMMQHPQT
AAMYQSAEMKGWPSGSMARSSSFPQQQFSHQGNPAAYSMMHMNGSSSHIGQMNINTMSMSGMPMAPDQVSC
>246Pachyramphus minor|A0A7K5DFV7|Aves|SRC3
CMFKMSGLGENSLDSLTNESRKRKPLPCDTPGASLTCSGEKRRREQESKYIEELAELISANLSDIDNFNVKPDK
CAILKETVRQIRQIKDQGKAVSNDDDVQKADVSSTGQGVIDKDSLGPLLLQAALDGFLFVVNRDGNIVFVSENV
TQYLQYKQEDLVNTSVYGILHEEDRKDFLKNLPKSSVNGVAWTSETPRQKSHTFNCRMMVKSSHDLLEDVGSHQ
DGRQRYETMQCFALSQPRAMMEEGEDLQSCMICVARRITTAERVFLTNPESFITRHDLSGKLVNIDTNSLRSSM
RPGFEDIIRRCIQRFLCHNDGQSWSNKRHYHEAYTQGHAETPLYRFSLADGTIVTAQTKSKLFRNPVTNDRHGF
VSTHFLQREQNGYRPNPNSVGQNIRTPVAGNTNSVGGLMNMSPGQGLPVQNRNYGMGDPNAVGQLSSTRYGGPG
NMGPVTSGPGMQSSAYQNNSYGLNISSPPHGSPGLTSNQQNLMISPRNRGSPKMSSHQYSPVTGMHSPMGSASN
TSNSTFSSSSLSALQAISEGVGNSLLSTLSSPGPKLDNSPNMSVTPQSKTSTQDSKSPSGLYCEQNQVESSVCQ
SNSRDLLSEKESKEGNLEASESQRGPSESKGHKKLLQLLTCSSDERGHSTASNSPLDSNCKEPSTSVTSPSGVS
SSTSGGVSSSSNVHGSLLQEKHRILHKLLQNGNSPAEVAKITAEATGKDTYHETGNTSSCGEGTVKQEQLSPKK
KENNALLRYLLDKDDVKDPLSKELKPKVESVDNKMGQCTSSTIPTSSQDKEMKIKMEPAEEMNGDLDNLDAILG
DLTSSEFYNNAMSANGNNLGTKQALFQGNALLGVKNSQSVQTARPPFNRAMSLDSPVGSSPSVRNVNAFSMLQK
QNLMGGSPRMIENQENFGANLGFGPSRSVAMSQQQPDWAVPGCKGSRLEAAGAAALGRAGPEFSAVLPRPPGSV
PGLPLRSNSLPASRPGLQQPLIHMRPNELNMGMAGNPYGQPGPSSQPGLWPDNVLSVEQASRGSQNRQLVRNSL
DDLLCSAPSMEGQNDERALLDQLHTLLSNTDVTGLEEIDRALGIPDLVSQGQALEPKQDSFQGQESSVMMDQKA
SLYAQPYQGQGATMPGGFSNIQGQQPSFNSVMNQMGQPSNFPLQSVHPRANVMRPRTNTPKQLRMQLQQRLQGQ
QFMNQTRQTLEMKMESPVSGNTSAMRPVMQPQVGSQQQGFLNAQMVAQRNRELISHHFRQQRMAMMMQQQQQPQ
AFSPPPNVTATGSMDSALTGPPMAQVPPQQFPYPPNYGLSQQPDPAFTRVSSPPNPMISSRIGASQNPVMQHPQ
PATMYQSSEMKGWPSGSMARNSSFPQQQFSHQGNPAAYSMMHMNGSSGHMGQMSINAMPMSGMPMGPDQVSC
>247Chunga burmeisteri|A0A7K5G540|Aves|SRC3
CMVKMSGLGENSLDSLTNESRKRKPLPCDTPGASLTCSGEKRRREQESKYIEELAELISANLSDIDNFNVKPDK
CAILKETVRQIRQIKEQGKAVSNDDDVQKADVSSTGQGVIDKDSLGPLLLQAALDGFLFVVNRDGNIVFVSENV
TQYLQYKQEDLVNTSVYGILHEEDRKDFLKNLPKSSVNGVAWTNETPRQKSHTFNCRMMVKTSHDLLEDVGNHQ
EIRQRYETMQCFALSQPRAMMEEGEDLQSCMICVARRITTAERVFPTNPESFITRHDLSGKLVNIDTNSLRSSM
RPGFEDIIRRCIQRFLCHNDGQSWSNKRHYHEAYTQGHAETPLYRFSLADGTIVTAQTKSKLFRNPVTNDRHGF
VSTHFLQREQNGYRPNPNSVGQSIRTPVPGNTNSVGGLMNMSPGQVMPMQNRSYGMGDPSAVGQLSSTRYGGPG
NMGPVNSGPGMQSSAYQNNSYGLNISSPPHGSPGLTSNQQNLMISPRNRGSPKMSSHQFSPVTGMHSPMGSASN
TGNSTFSSSSLSALQAISEGVGTSLLSTLSSPGPKLDNSPNMNIAQQNKTSSQDSKSPSGLYCEQNQVESSICQ
SNSRDLLSEKDSKEGNLEASESQRGPPESKGHKKLLQLLTCSSDDRGHSTASNSPLDSNCKESSTNVTSPSGVS

SSTSGGVSSSSNVHGSLLQEKHRILHKLLQNGNSPAEVAKITAEATGKDTYHEASNTAPCGEGTVKQEQLSPKK
KENNALLRYLLDKDDGKDLLSKELKPKVESVDNKMGQCSSSTIPTSSQEKEMKIKTEPAEEMSGDLDNLDAILG
DLTSSEFYNNAMSANGNNLGTKQALFQGNALLGMRSPQSVQTTRPPFNRAMSLDSPVGSSAPVRNVNAFSMLQK
QNLMGGSPRMIENQENFGTNLGFAPNRNVAINQHQSGDWGLPHSKVNRLEPTSSASMLRPGPEFSASLTRPTMG
GSVSGLPVRSNSIPGTRPMLQQQMIHMRPNEINIGMGGNPYGQPGPSNQPGSWPDNMLSMEQAPRGSQNRQLVR
NSLDDLLCPTPNMEGQTDERALLDQLHTLLSNTDVTGLEEIDRALGIPDLVNQGQALEPKQDSFQGQESSVMID
QKPSLYGQPYQGQGAAMPGGFSNIQGQQPSFNSVMNQMSQQSNFPLQSMHPRANVMRPRTNTPKQLRMQLQQRL
QGQQFMNQTRQALEMKMENPVSGNSAVMRPVMQPQLGSQQQGFLNAQMVAQRNRELISHHFRQQRMAMMMQQQQ
QPQAFSPPPNVTASGGMDSALTGPPMAQVPPQQFPYPPNYGISQQPDPAFSRVSSPPNPMMSSRIGPSQNPMMQ
HPQTTTMYQSPEMKGWPSGSMARSSSFPQQQFSHQGNPAAYSMMHMNGSSGHIGQMNINTMPMSGMPMGPDQVS
C
>248Crotophaga sulcirostris|A0A7K5HQ72|Aves|SRC3
CVVRMSGLGENSLDNLTNESRKRKPLPCDTPGASLTCSGEKRRREQESKYIEELAELISANLSDIDNFNVKPDK
CAILKETVRQIRQIKEQGKAVSNDDDVQKADVSSTGQGVIDKDSLGPLLLQAALDGFLFVVNRDGNIVFVSENV
TQYLQYKQEDLVNTSVYSILHEEDRKDFLKNLPKSSVNGVAWTNETPRQKSHTFNCRMMVKTSHDLLEDVGNHQ
DLRQRYETMQCFALSQPRAMMEEGEDLQSCMICVARRITTAERVFPTNPESFITRHDLSGKLVNIDTNSLRSSM
RPGFEDIIRRCIQRFLCHNDGQSWSNKRHYHEAYTQGHAETPLYRFSLADGTIVTAQTKSKLFRNPVTNDRHGF
VSTHFLQREQNGYRQTPNAVGQNIRTPVAGNTNAVGGLMNMSPSQGMPVQNRNYGMGDTNAVGQLSSARYGGPA
SMGPVNSGPGMQSSAYQNNSYGLNINSPPHGSPGLTSNQQNLMISPRNRGSPKMNSHQFSPVTGMHSPMGSASN
TGNSTFSSSSLSALQAISEGVGTSLLSTLSSPGPKLDNSPNMTVAQQSKASTQDSKSPSGLYCEQNQVESSVCQ
SNSRDLLSEKDSKEGNLETPESQRGPSESKGHKKLLQLLTCSSDERGLSTASNSPLDSNCKESSSNVTSPSGVS
SSTSGGVSSSSNMHGSLLQEKHRILHKLLQNGNSPAEVAKITAEATGKDTYHDASNPAPCGESTVKQEQLSPKK
KENNALLRYLLDKDDVKDPLSKELKPKVESVDNKMGQCSSSTIPTSSQEKEMKIKTEPTEEMSGDLDNLDAILG
DLTSSEFYNNAMSANGNNLGTKQALFQGNALLGMRSPQPVQTARPPFTRAMSLDSPVGSGASVRNVNAFSMLQK
HNLMGESPRMIENQENFGANLGFAPNRNVSMNQHQAGDWGLPNSKVNRLEPASSASMLRPGPEFGASLPRPTVG
GSVSGLPVRSNSVPGTRPVLQQQMIHMRPNEMNIGMGGNPYGQPGPSTQPGSWPDNVLSMEQAARSSQNRQLVR
NSLDDLLCPAPNMEGQNDERALLDQLHTLLSNTDVTSLEEIDRALGIPDLVNQGQGLEPKQDSFQGQESSVMID
QKSSLYGQPYQGQGAAMPGGFSNMQGQQPSFNSVMNQMSQQSSFPLQSIHPRANVMRPRTNTPKQLRMQLQQRL
QGQQFMNQTRQAMEMKMESPVSGNTSVMRPVMQPQVGSQQQGFLNAQMVAQRNRELISHHFRQQRMAMMMQQQQ
QPQTFSPPPNVTASGSMDSALTGPPMAQVPPQQFPYPPNYGISQQPDPAFSRVPSPPNPMMSSRMGPSQNPMMQ
HPQTATMYQSPEMKGWPSGSMARSSSFPQQQFSHQGNPAAYSMMHMNGTSGHIGQMNINTMSMSGMPMGPDQVS
C
>249Mionectes macconnelli|A0A7K5KDH1|Aves|SRC3
CMVTMSGLGENSLDSLTSESRKRKPLPCDTPGASLTCSGEKRRREQESKYIEELAELISANLSDIDNFNVKPDK
CAILKETVRQIRQIKDQGKAVSNDDDVQKADVSSTGQGVIDKDSLGPLLLQAALDGFLFVVNRDGNIVFVSENV
TQYLQYKQEDLVNTSVYGILHEEDRKDFLKNLPKSSVNGVAWTSETPRQKSHTFNCRMMVKSSHDLLEDVGSHQ
DGRQRYETMQCFALSQPRAMMEEGEDLQSCMICVARRITTAERVFPTNPESFITRHDLSGKLVNIDTNSLRSSM
RPGFEDIIRRCIQRFLCHNDGQSWSNKRHYHEAYTQGHAETPLYRFSLADGTIVTAQTKSKLFRNPVTNDRHGF
VSTHFLQREQNGYRPNPNSVGQNIRTPVAGNTNSVGGLMNMSPGQGMPVQSRNYGMGDPNAVGQLGSTRYGGPG
NVGPVTSGTGVQSSAYQNNSYGLNISSPPHGSPGLTSNQQNLMISPRNRGSPKMSSHQYSPVTGMHSPMGSASN
TSNSTFSSSSLSALQAISEGVGNSLLSTLSSPGPKLDNSPNMSVTPQSKTSTQDSKSPSGLYCEQNQVESSICQ
SNSRDLLSEKESKEGNLEASDSQRGPSESKGHKKLLQLLTCSSDERGHSTASSSPLDSNCKEPSTSVTSPSGVS
SSTSGGVSSSSNVHGSLLQEKHRILHKLLQNGNSPAEVAKITAEATGKDTYHDAGNTTSCGEGTIKQEQLSPKK
KENNALLRYLLDKDDVKDPLSKELKPKVESVDNKMGQCTSSTIPTSSQDKEMKIKMEPAEEMNGDLDNLDAILG
DLTSSEFYNNAMSANGNNFGTKQALFQGNALLGVKSSQSVQTARPPFNRAMSLDSPVGSSASVRNVSAFSMLQK
QNLMGGSPRMIENQENFGANLGFAPSRSVAMNQHQPEWGLPSSKGSRLDPAGSAGLGRAGPELSTALPRPTGSV
PALPLRSNSLPASRPGLQQPLVHMRPNELNIGMAGNPYGQPGPSNQPGLWPDNVLSVEQASRVSQNRQLVRNSL
DDLLCSAPNMEGQNDERALLDQLHTLLSNTDVTGLEEIDRALGIPDLVSQGQALEPKQDSFQGQESSVMIDQKP
SLYAQPYQGQGATMPGGFSNIQGQQPSFNSVMNQMGQPSNFPLQSVHPRANVMRPRTNTPKQLRMQLQQRLQGQ
QFMNQTRQTLEIKMESPVSGNTSAMRPVMQPQVGSQQQGFLNAQMVAQRNRELISHHFRQQRMAMMMQQQQQPQ
AFSPPPNVTATGSMDSALTGPPMAQVPPQQFPYPPNYGISQQPDPAFSRVSSPPNPMISSRIGPSQNPVMQHPQ
PATMYQSSEMKGWPSGSMARNSSFPQQQFSHQGNPAAYSMMHMNGSSGHMGQMSINAMPMSGMPMGPDQVSC

>250Pterocles burchelli|A0A7K5YNA6|Aves|SRC3

CMVKMSGLGENSLDSLTNESRKRKPLPCDTPGASLTCSGEKRRREQESKYIEELAELISANLSDIDNFNVKPDK
CAILKETVRQIRQIKEQGKAVSNDDDVQKADVSSTGQGVIDKDSLGPLLLQAALDGFLFVVNRDGNIVFVSENV
TQYLQYKQEDLVNTSVYGILHEEDRKDFLKNLPKSSVNGVAWTNEAPRQKSHTFNCRMMVKTSHDLLEDVGSHQ
DTRQRYETMQCFALSQPRAMMEEGEDLQSCMICVARRITTAERVFPTNPESFITRHDLSGKLVNIDTNSLRSSM
RPGFEDIIRRCIQRFLCHNDGQSWSNKRHYHEAYTQGHAETPLYRFSLADGTIVTAQTKSKLFRNPVTNDRHGF
VSTHFLHREQNGYRPNPNSVGQNIRTPVAGNTNSVGGLMNMSPGQGMPVQNRNYGVGDPNAVGQLSSTRYGGPG
NMGPVNSGPGMQSSAYQNNSYGLNISSPPHGSPGLTSNQQNLMISPRNRGSPKMSSHQFSPVTGMHSPMGSASN
TGNSSFSSSSLSALQAISEGVGTSLLSTLSSPGPKLDNSPNMNIAQQSKTSAQDSKSPSGLYCEQNQVESSVCQ
SNSRDLLSEKDSKEGNVEASESQRGPSESKGHKKLLQLLTCSSDERGHSTASNSPLDSNCKESSTNVTSPSGVS
SSTSGGVSSSSNMHGSLLQEKHRILHKLLQNGNSPAEVAKITAEATGKDTYLDASNTAPCGEGTVKQEQLSPKK
KENNALLRYLLDKDDVKDPLSKELKPKVESVDNKMGQCSSSTIPTSSQEKEMKIKTEPAEEMSGDLDNLDAILG
DLTSSEFYNNAMSANGNNLGTKQALFQGNALLGMRSPQSVQTARPPFNRSMSLDSPVGSSAPVRNVNAFSMLQK
QNLMGGSPRMIENQENFGANLGFAPNRNVAMNQHQTTPGDWGLPNSKVNRLEPTSSASMLRPGPEYDASLPRPA
VGGSLSGLPVRSNSIPGTRPMLQQQMMHMRPNEINLGMGGNPYGQPGPSNQPGSWPDNMLSMEQASRGSQNRQL
VRNSLDDLLCPAPNMEGQNDERALLDQLHTLLSNTDVTGLEEIDRALGIPDLVNQGQALEPKQDSFQGQESSVM
IDQKPSLYGQPYQGQGAAMPGGFSNIQGQQPSFNSVMNQMSQQSNFPLQSMHPRANVMRPRANTPKQLRMQLQQ
RLQGQQFMNQTRQALEMKMENPVSGNTSVMRPVMQPQVGSQQQGFLNAQMVAQRNRELISHHFRQQRMAMMMQQ
QQQPQAFSPPPNVTASGSMDTALTGPPMAQVPPQQFPYPPNYGISQQPDPAFSRVSSPPNPMMSSRIGPSQNPM
MQHPQAATMYQSPEMKGWPSGSMARSSTFPQQQFSHQGNPAAYSMMHMNGSGGHIGQMNINTMPMAGMPMGPDQ
VSC
>251Pedionomus torquatus|A0A7K6P1G9|Aves|SRC3
CMFKMSGLGENSLDGLTNESRKRKPLPCDTPGASLTCSGEKRRREQESKYIEELAELISANLSDIDNFNVKPDK
CAILKETVRQIRQIKEQGKAVSNDDDVQKADVSSTGQGVIDKDSLGPLLLQASALDGFLFVVNRDGNIVFVSEN
VTQYLQYKQEDLVNTSVYGILHEEDRKDFLKNLPKSSVNGVAWTNETPRQKSHTFNCRMMVKTSHDLLEDVGSH
QDARQRYETMQCFALSQPRAMMEEGEDLQSCMICVARRITTAERAFPTNPESFITRHDLSGKLVNIDTNSLRSS
MRPGFEDIIRRCIQRFLCHNDGQSWSNKRHYHEAYTQGHAETPLYRFSLADGTIVTAQTKSKLFRNPVTNDRHG
FVSTHFLQREQNGYRPNSNPVGQNIRTPVTGNTNSVGGLMNMSPGQGMPMQNRSYGMGDPSAVGQLSNTRYGGP
GSMGPVNSGPGMQSSAYQNNSYNLNISSPPHGSPGLTSNQQNLMISPRNRGSPKMSSHQFSPVTGMHSPMGSAS
NTGNSTFSSSSLSALQAISEGVGTSLLSTLSSPGPKLDNSPNMSIAQQSKTSTQDSKSPSGLYCEQNQVESSIC
QSNSRDLLSEKDSKEGNLESSESQRGPSESKGHKKLLQLLTCSSDERGHSTASNSPLDSNCKESSTNVTSPSGV
SSSTSGGVSSSSNMHGSLLQEKHRILHKLLQNGNSPAEVAKITAEATGKDTYHDASNTAPCGEGTVKQEQLSPK
KKENNALLRYLLDKDDVKDPLSKELKPKVESVDNKMGQCSSSTIPTSSQEKEMKIKMEPAEEMSGDLDNLDAIL
GDLSSSEFYNNAMSANGNNLGTKQALFQGNALLGVRSPQSVPTTRPPFTRAMSLDSPMGSGAPARNANAFSMLQ
KQNLMGGSPRMIESQENFGANLGFAPNRNVVMNQHQAGDWGLSNSKVNRLEPTSSASMLRAGPEFSATLPRASV
CGSMPGLPARSNSIPGTRPVLQQQMVHMRPNEMNIAMAGNPYGQPGPSNQPGSWPDNMLSMEQASRGSQNRQLV
RSSLDDLLCPAPNMEGQTDERALLDQLHTLLSNTDVTSLEEIDRALGIPDLVNQGQALEPKQDSFQGQESSVML
DQKPSLYGQPYQGQGAAMPGSFSNIQGQQPTFNSVMNQMSQQSNFPLQSMHPRANVMRPRTNTPKQLRMQLQQR
LQGQQFMNQTRQALEMKMENPVSGNTSVMRPVMQPQVGSQQQGFLNAQMVAQRNRELISHHFRQQRMAMMMQQQ
QQPQAFSPPPNVTASGSMDSALTGPPMAQVPPQQFPYPPNYGISQQPDPAFSRVSSPPNPMMSSRIGASQNPMM
QHPQTAAMYQSPEMKGWPSGSMARSSSFPQQQFSHQGNPAAYSMMHMNGSSGHIGQMNINTMPMSGMPMGPDQV
SC
>252Caloenas nicobarica|A0A7K6SP55|Aves|SRC3
CMVKMSGLGENSLDSLTNESRKRKPLPCDTPGASLTCSGEKRRREQESKYIEELAELISANLSDIDNFNVKPDK
CAILKETVKQIRQIKEQGKAVSNDDDVQKADVSSTGQGVIDKDSLGPLLLQAMCLTCCRINIQALDGFLFVVNR
DGNIVFVSENVTQYLQYKQEDLVNTSVYGILHEEDRKDFLKNLPKSSVNGVAWTNETPRQKSHTFNCRMMVKTS
HDLLEDVGSHQDTRQRYETMQCFALSQPRAMMEEGEDLQSCMICVARRITTAERVFPTNPESFITRHDLSGKLV
NIDTNSLRSSMRPGFEDIIRRCIQRFLCHNDGQSWSNKRHYHEAYTQGHAETPLYRFSLADGTIVTAQTKSKLL
RNPVTNDRHGFVSTHFLQREQNGYRPNPNSVGQNIRAPVAGNTNSAGGLMNMSAGQGLPAQSRSYGMADPSAVG
QLSSTRYGGPGNAGPANPGPGMQSSAYQNNSYGLNISSPPHGSPGLTSNQQNLMISPRNRGSPKMSSHQFSPVT
GMHSPMGSASNAGNNTFSSSSLSALQAISEGVGTSLLSTLSSPGPKLDNSPNMSVSQQSKTSTQDSKSPSGLYC
EQNQVESSICQSNSRDLLSEKDSKEGNLEASESQRAPSESKGHKKLLQLLTCSSDERGHSTASNSPLDSSCKES
STNVTSPSGVSSSTSGGVSSSSNMHGSLLQEKHRILHKLLQNGNSPAEVAKITAEATGKDTYHDVSNIAPCGEG
TVKQEQLSPKKKENNALLRYLLDKDDVKDPLSKELKPKVENVDNKMGQCSSSTIPTSSQEKETKIKPEPTEEMS
GDLDNLDAILGDLTSSEFYNNAMSANGNNLGTKQALFQGNALLGTRSPQSVQTARPPFSRAVSLDSPVGSGAPV

RNVNAFSMLQKQNLMGGSPRMIENQENFGANLGGAPNRNVAMSQHQAGDWGLPSSKVSRGEPAGSASMGRPAAE
FSASLPRPAAGGSVSGLPVRSNSIPGTRPMLQQPMIHMRPNEINIGMGGNPYGQPGPSNQPSSWPDSMLSMEQA
SRGSQNRQLVRNSLDDLLCPAPSMEGQSDERALLDQLHTLLSNTDVTSLEEIDRALGIPELVNQGQGLEAKQDS
FQGQESSVMIDQKPSLYGQPYQGQGAAMPGGFSNIQGQQPSFNSVMNQMSQQSSFPIQSMHPRANVMRPRTNTP
KQLRMQLQQRLQGQQFMNQTRQALEMKMENPVSGNTSVMRPVMQPQVGSQQGFLNAQMVAQRNRELISHHFRQQ
RMAMMMQQQQQPQAFSPPPNVTASGSMDSALAGPPMAQVPPQQFPYPPNYGISQQPDPAYSRVSSPPNPMMSSR
MGPSQNPMMQHPQPAPMYQSPEMKGWPSGSMARSSFPQQQFSHQGNPAAYSMMHMNGSSGHMGQMNISTMPMSG
MPMGPDQVSC
>253Aegotheles bennettii|A0A7K6U7V4|Aves|SRC3
CMVKMSGLGENSLDSLTNESRKRKPLPCDTPGASLTCSGEKRRREQESKYIEELAELISANLSDIDNFNVKPDK
CAILKETVRQIRQIKEQGKAVSSDDDVQKADVSSTGQGVIDKDSLGPLLLQAALDGFLFVVNRDGSIVFVSENV
TQYLQYKQEDLVNTSVYGILHEEDRKDFLKNLPKSSVNGVAWTNEAPRQKSHTFNCRMMVKTSHDVLEDVGSHQ
DTRQRYETMQCFALSQPRAMMEEGEDLQSCMICVARRITTAERVFPTNPESFITRHDLSGKLVNIDTNSLRSSM
RPGFEDIIRRCIQRFLCHNDGQSWSNKRHYHEAYTQGHAETPLYRFSLADGTIVTAQTKSKLFRNPVTNDRHGF
VSTHFLQREQNGYRPNPNSVGQNIRTPVAGNTNSVGGLMNMSPGQGMPMQNRNYGIGDPNTVSQLSSTRYGGPG
NMGPVNSGPGMQSSAYQNNSYGLNISSPPHGSPGLTSNQQNLMISPRNRGSPKMSSHQFSPVTGMHSPMGSASN
TSNSTFSSSSLSALQAISEGVGTSLLSTLSSPGPKLDNSPNMNIAQQSKTNIQDSKSPSGLYCEQNQVESSICQ
SNSRDLGEKDSKEGNLEASESQRGPSETKGHKKLLQLLTCSSDERGHSTASNSPLDSNCKESSTNVTSPSGVSS
STPGGVSSSSNMHGSLLQEKHRILHKLLQNGNSPAEVAKITAEATGKDTYHDASNTTLCAEGTVKQEQLSPKKK
ENNALLRYLLDKDDVKDPLSKELKPKIESVENKMGQCSSSTISTSSQEKDKKIKTEPTEEVSGDLDNLDVILGD
LTSSEFYNNAMSANGNNLGTKQALCQGNALLGTRSPQSVQTRPPFTRAMSLDSPVGSSAAVRNVNAFSMLQKQN
LMGGSPRMIENQENFGANLGFAPNRNVAMNQHQSGDWGLPNSKVNRLEPTTSASMLRPGPEFSTSLPRPLVGGS
MSGLPVRSNSIPGTRPMLQQQMIHMRPNEINIGMGGNPYGQPGPSNQPGSWPDSMLSMEQASRGSQNRQLVRNS
LDDLLCPAPNMEGQNDERALLDQLHTLLSNTDVTGLEEIDRALGIPDLVNQGQALEPKQDSFQGQESSVMIDQK
PSLYGQPYQGQGAGMPGGFSNIQGQQPSFNSVMNQMSQQSNFPLQSMHPRANAMRPRTSTPKQLRMQLQQRLQG
QQFMNQTRQALEMKMENPVSGNTSVMRPVMQPQVASQQQGFLNAQMVAQRNRELISHHFRQQRMAMMMQQQQQP
QAFSPPPNVTASGSMDSALTGPPMAQVPPQQFPYPPNYGISQQPDPAFSRVSSPPNPMMASRIGPTQNPMMQHP
QTATMYQSPEMKGWPSGSMARSSSFQQQQFSHQGNAAAYSMMHMNGSSGHIGQININAMPMSGMPMGPDQVSC
>254Promerops cafer|A0A7K6XCM5|Aves|SRC3
CMVKMSGLGENSVDSLTNESRKRKPLPCDTPGASLACSGEKRRREQESKYIEELAELISANLSDIDNFNVKPDK
CAILKETVRQIRQIKEQGKAVSNDDDVQKADVSSTGQGVIDKDSLGPLLLQAALDGFLFVVNRDGNIVFVSENV
TQYLQYKQEDLVNTSVYGILHEEDRKDFLKNVPKSSVNGVAWTNETPRQKSHTFNCRMMVKSSHDLFEDIGGHQ
DIRQRYETMQCFALSQPRAMMEEGEDLQSCMICVARRITTAERVFPANPESFITRHDLSGKLVNIDTNSLRSSM
RPGFEDIIRRCIQRFLCHNDGQSWSNKRHYHEAYTQGHAETPLYRFSLADGTIVTAQTKSKLFRNPVTNDRHGF
VSTHFLQREQNGYRPNPNSVGQNIRTPVTGNTNSVNGLMNMSPGQAMPMQNRNYGMGDPNAVGQLTSTRYGGPG
NMGPVNSGPGMQSSAYQSNSFGLNISSPPHGSPGLTSNQQNLMVSPRNRGSPKMSSHQYSPVTGMHSPMGSASN
TSNSTFSSSSLSALQAISEGVGNSLLSTLSSPGPKLDNSSNISITQQIKTSTQDSKSPSGLHCEQNQVESSICQ
SNSKDLLSEKDSKEGIPETSESQRGPSESKGHKKLLQLLTCSSDERGHSTASNSPLDSNCKESSTSVTSPSGVS
SSTSGGVSSSSNMHGSLLQEKHRILHKLLQNGNSPAEVAKITAEATGKDTYHDASNTVSCGEGTVKQEQLSPKK
KENNALLRYLLDKDDIKDPLSKELKPKVEGVDKMGQCTSSTIPASSQEKEMKIKMEPAEEMNGDLDNLDAILGD
LTSSEFYNNAMSANGNNPGTKQALFQGNALLGVKSPQSVQTTRPPFNRALSLDSPVGSGAPVRNVNAFSMLQKQ
NLMGGSPRMIENQENFGANLGFAPNRNVALNRHQSGDWGLPNSKVNRLESASSASMLRPGPEFSTTLPRPPEGG
SVSGLPVRSNSIPGTRPMLQQQMIHMRPNEVSIDMGGNLYGQPGPSTQPGLWPDNMISMEQTSRGSQNRQLVRS
SLDDLLCSAPIMEGQNDERALLDQLHTLLSNTDVTSLEEIDRALGIPDLVNQGQALEPKQDSFQGQESSVMIDQ
KPFIYGQPYQGQGAAMPGAFSNIQGQQPSFNSVMNQMSQPSNFSLQSMHPRANMMRPRTNTPKQLRMQLQQRLQ
GQQFMNQTRQTLEIKMENPVSGTSSVMRPVMQPQVGSQQQGFLNAQMVAQRNRELISHHFRQQRMAMMMQQQQQ
PQAFSPPPNVTASGSMDSALTGPPMAQVPSQQFPYPPNYGISQQPDPAFSRVSSPPNPMIPSRIGPSQNPMLQH
PQSATMYQSSEMKGWPSGSMPRNSSFPQQQFSHQGSPTAYSMMHMNGSGGHIGQMSINTMPMSGLPMGPDQVSC

>255Chionis minor|A0A7K7FBR4|Aves|SRC3
CMVKMSGLGENSLDSLTSESRKRKPLPCDTPGASLTCSGEKRRREQESKYIEELAELISANLSDIDNFNVKPDK
CAILKETVRQIRQIKEQGKAVSNDDDVQKADVSSTGQGVIDKDSLGPLLLQASALDGFLFVVNRDGNIVFVSEN
VTQYLQYKQEDLVNTSVYGILHEEDRKDFLKNLPKSSVNGVAWSNETPRQKSHTFNCRMMVKTSHDLLEDVGSH
QDTRQRYETMQCFALSQPRAMMEEGEDLQSCMICVARRITTAERVFPTNPESFITRHDLSGKLVNIDTNSLRSS

MRPGFEDIIRRCIQRFLCHNDGQSWSNKRHYHEAYTQGHAETPLYRFSLADGTIVTAQTKSKLFRNPVTNDRHG
FVSTHFLQREQNGYRANPTSVGQNIRTPVAGNTNSVGGLMNMSPGQGMSMQNRNYGMGDPSAVGQLSTTRYGGP
GSMGPVNSGPGMQPAAYQNSSYGLNISSPPHGSPGLTSNQQNLMISPRNRGSPKMSSHQFSPVTGMHSPMGSAT
STGNSTFSSSSLSALQAISEGVGTSLLSTLSSPGPKLDNSPNMNIAQQGKTSTQDSKSPSGLYCEQNQVESSIC
QSNSRDLLSEKDSKEGNLEASESQRGPSESKGHKKLLQLLTCSSDERGHSTASNSPLDSNCKESSTNVTSPSGV
SSSTSGGVSSSSNMHGSLLQEKHRILHKLLQNGNSPAEVAKITAEATGKDTYHDAGNTAPCGEGAVKQEQLSPK
KKENNALLRYLLDKDDVKDPLSKELKPKVESVDNKMGQCSSSTIPTSSQEKDMKIKTEPAEEMSGDLDNLDAIL
GDLTSSEFYNNAMSANGNNLGTKQALFQGNALLGTRSPQSAPTTRPPFTRAMSLDSPVGSSAPVRNVNAFSMLQ
KQNLMGGSPRMIENQENFGANLGSAPNRNVALNQHQSGDWGLPNSKANRLEPASSASMLRPGPEFSASLPRATV
GGSVSGLPVRSNSIPGTRPMLQQQMIHMRPNEINLGMGGNPYGQPGPSNQPGSWPDNILSMEQASRGSQNRQLV
RNSLDDLLCPAPNMEGQNDERALLDQLHTLLSNTDVTGLEEIDRALGIPDLVNQGQALEPKQDSFQGQESSVMI
DQKPSLYGQPYQGQGTAMPGSFSNIQGQQPTFNSVMNQMSHQSNFPLQSMHPRANVMRPRTNTPKQLRMQLQQR
LQGQQFMNQTRQALEMKMENPVSGNTSVMRPVMQPQVGSQQQGFLNAQMVAQRNRELISHHFRQQRMAMMMQQQ
QQPQAFSPPPNVTAAGSMDSALTGPPMAQVPPQQFPYPPNYGISQQPDPAFSRVSSPPNPMMSSRIGPSQNPMM
QHPQTATMYQSPEMKGWPSGSMARSSSFPQQQFSHQGNAAAYSMMHMNGSTGHMGQMNINTMPMAGMPMAPDQV
SC

>256Lophotis ruficrista|A0A7K8JNR4|Aves|SRC3
CVVKMSGLGENSLDNLTNESRKRKPLPCDTPGASLTCSGEKRRREQESKYIEELAELISANLSDIDNFNVKPDK
CAILKETVRQIRQIKEQGKAVSNDDDVQKADVSSTGQGVIDKDSLGPLLLQAALDGFLFVVNRDGNIVFVSENV
TQYLQYKQEDLVNTSVYGILHEEDRKDFLKNLPKSSVNGVAWTNETPRQKSHTFNCRMMVKTSHDLLEDVGSHQ
DTRQRYETMQCFALSQPRAMMEEGEDLQSCMICVARRITTAERVFLTNPESFITRHDLSGKLVNIDTNSLRSSM
RPGFEDIIRRCIQRFLCHNDGQSWSNKRHYHEAYTQGHAETPLYRFSLADGTIVTAQTKSKLFRNPVTNDRHGF
VSTHFLQREQNGYRPNPNSVGQNIRTPVAGNTNSGGGLMNMSPGQGMPMQNRNYGMGDPNAVGQLSSTRYGGPG
SMGPVNSGPGMQSSAYQNNSYGLNISSPPHGSPGLTSNQQNLMISPRNRGSPKMSSHQFSPVTGMHSPMGSASN
TGSSTFSSSSLSALQAISEGVGTSLLSTLSSPGPKLDNSPNMSIAQQSKTSTQDSKSPSGLYCEQNQVESSVCQ
SNSRDLLSEKDSKEGNLEASESQRGPSESKGHKKLLQLLTCSSDDRGHSTASNSPLDSNCKESSTNVTSPSGVS
SSTSGGVSSSSNVHGSLLQEKHRILHKLLQNGNSPAEVAKITAEATGKDTYHDASNAAPCGEGTVKQEQLSPKK
KENNALLRYLLDKDDVKDPLSKELKPKIESVDNKTGQCSSSTIPTSSQEKEMKIKTEPTEEMSGDLDNLDAILG
DLTSSEFYSNAMSANGNNLGTKQALFQGNALLGMRSPQSVQTTRPPFNRAMSLDSPVGSGAAARNVNAFSMLQK
QNVMGGSPRMIENQENFGANLGFAPNRNVAINQHQSGDWGLPNSKVNRLEPTSSASMLRPGPEFSASLPRPTVG
ASMSGLPVRSNSMPGTRPMLQQQMIHMRPNEINIGMGGNPYGQPGPSNQPGSWPDSMLSMEQASRGSQNRQLVR
NSLDDLLCPAPSMEGQNDERALLDQLHTLLSNTDVTGLEEIDRALGIPDLVNQGQALEPKQDSFQGQESSVMID
QKPSLYGQPYQGQGAAMPGGFSNIQGQQPSFNSVMNQMGQQSNFPLQSIHPRANAMRPRTNTPKQLRMQLQQRL
QGQQFMNQTRQALEMKMENPVSGNTSVMRPVMQPQVGSQQGFLNAQMVAQRNRELISHHFRQQRMAMMMQQQQQ
PQAFSPPPNVTASGSMDSALTGPPMAQVPAQQFPYPPNYGISQQPDPAFSRVSSPPNPMMSSRLGPSQNPMMQH
PQTAAMYQSPEMKGWPSGSMARSSSFPQQQFSHQGNPAAYSMMHMNGSSGHIGQMSINTMPMAGMPMGPDQVSS

>257Ardeotis kori|A0A7K8L4F9|Aves|SRC3
CVVKMSGLGENSLDNLTNESRKRKPLPCDTPGASLTCSGEKRRREQESKYIEELAELISANLSDIDNFNVKPDK
CAILKETVRQIRQIKEQGKAVSNDDDVQKADVSSTGQGVIDKDSLGPLLLQAALDGFLFVVNRDGNIVFVSENV
TQYLQYKQEDLVNTSVYGILHEEDRKDFLKNLPKSSVNGVAWTNETPRQKSHTFNCRMMVKTSHDLLEDVGSHQ
DTRQRYETMQCFALSQPRAMMEEGEDLQSCMICVARRITTAERVFPTNPESFITRHDLSGKLVNIDTNSLRSSM
RPGFEDIIRRCIQRFLCHNDGQSWSNKRHYHEAYTQGHAETPLYRFSLADGTIVTAQTKSKLFRNPVTNDRHGF
VSTHFLQREQNGYRPNSNSVGQNIRTPVAGNTNSVGGLMNMSPGQGMPMQNRNYGMGDPNAVGQLSSTRYGGPG
SMGPVNSGPGMQSSAYQNNSYGLNISSPPHGSPGLTSNQQNLMISPRNRGSPKMSSHQFSPVTGMHSPMGSASN
TGSSTFSSSSLSALQAISEGVGTSLLSTLSSPGPKLDNSPNMSIAQQSKTSTQDSKSPSGLYCEQNQVESSVCQ
SNSRDLLSEKDSKEGNLEASESQRGPSESKGHKKLLQLLTCSSDDRGHSTASNSPLDSNCKESSTNVTSPSGVS
SSTSGGVSSSSNVHGSLLQEKHRILHKLLQNGNSPAEVAKITAEATGKDTYHDASNATPCGEGTVKQEQLSPKK
KENNALLRYLLDKDDVKDPLSKELKPKIESVDNKTGQCSSSAIPTSSQEKEMKIKTEPTEEMSGDLDNLDAILG
DLTSSEFYNNAMSANGNNLGTKQALFQGNALLGMRSPQSVQTTRPPFNRAVSLDSPVGSGAAARNVNAFSMLQK
QNVMSGSPRMVESQETFGANLGFAPNRNVAMNQHQSGDWGLPNSKVNRLEPTSSTSMLRPGPEFSASLPRPTVG
GSMSGLPVRSNSMPGTRPMLQQQMIHMRPNEINIGMGGNPYGQPGPSNQPGSWPDNMLSMEQASRGSQSRQLVR
NSLDDLLCPAPSMEGQNDERALLDQLHTLLSNTDVTGLEEIDRALGIPDLVNQGQALEPKQDSFQGQESSVMID

QKPSLYGQPYQGQGAAMPGGFSNIQGQQPSFNSVMNQMGQQSNFPLQSMHPRANVMRPRTNTPKQLRMQLQQRL
QGQQFMNQTRQALEMKMENPVGGSTSVMRPVMQPQVGSQQQGFLNAQMVAQRNRELISHHFRQQRMAMMMQQQQ
QPQAFSPPPNVTASGSMDSALTGPPMAQVPAQQFPYPPNYGISQQPDPAFSRVSSPPNPMMSSRLGPSQNPMMQ
HPQAAAMYQSPEMKGWPSGSMARSSSFPQQQFSHQGNPAAYSMMHMNGSSGHIGQMNINTMPMSGMPMGPDQVS
S

>258Casuarius casuarius|A0A7K8NI38|Aves|SRC3
CVIKMSGLGENSLDSLTNESRKRKPLPCDTPGASLTCSGEKRRREQESKYIEELAELISANLSDIDNFNVKPDK
CAILKETVRQIRQIKEQGKAVSNDDDVQKADVSSTGQGVIDKDSLGPLLLQASTNYALDGFLFVVNRDGNIVFV
SENVTQYLQYKQEDLVNTSVYGILHEEDRKDFLKNLPKSSVNGVAWTNETPRQKSHTFNCRMMVKTSHDLLEDV
GSHQDTRQRYETMQCFALSQPRAMMEEGEDLQSCMICVARRITTVERVFPTNPESFITRHDLSGKLVNIDTNSL
RSSMRPGFEDTIRRCIQRFLCHNDGQSWSNKRHYHEAYTQGHAETPLYRFSLADGTIVTAQTKSKLFRNPVTND
RHGFVSTHFLQREQNGYRPNPNSVGQNIRAPVAGSTNSVGGVMNMSPGQGMPMQNRNYGMGDPSAVGQLSSTRY
GGPGNMGPMNSGPGMQSSTYQNNNYGLNISSPPHGSPGLTSNQQNLMISPRNRGSPKMSSHQFSPVTGMHSPMG
SASNTSNSTFSSSSLSALQAISEGVGTSLLSTLSSPGPKLDNSPNMNIAQQSKTSTQDSKSPSGLYCEQNQVES
SVCQSNSRELLSEKDSKEGNLEASESQRGPSESKGHKKLLQLLTCSSDDRGHSTASSSPLDSNCKESSTNVTSP
SGVSSSTSGGVSSSSNMHSSLLQEKHRILHKLLQNGNSPAEVAKITAEATGKDTYHDASNTAPCGEGTVKQEQL
SPKKKENNALLRYLLDKDDIKDPLSKELKPKVEGVDNKMGQCSSSTLPTSSQEKEIKIKAEPTEEMSGDLDNLD
AILGDLTSSDFYNNAMSANGNSLGTKQALFPGTAPLSMRSPQAVQTARPPFSRAMSLDSPVGSSPPVRSVNAFS
MLQKQNLMGGSPRMIENQENFGANLGLGPNRNVAMNQHPSGDWGLPNSKVNRLEPTSSASMLRPGPEFNASLPR
AAVGGSVPGLPIRSNSVPGTRPMLQQQMIHMRPNEINIGMGGNPYGQPGPSNQPGSWPDNMLSMEQASRGSQNR
QLVRSSLDDLLCPAPNVEGQSDERALLDQLHTLLSNTDVTSLEEIDRALGIPDLVNQGQALEPKQDSFQGQESA
VMIDQKSSLYGQPYQGQGAAMPGGFSNIQGQQPSFNSVMNQMSQQSNFPLQSMHPRANVMRPRTNTPKQLRMQL
QQRLQGQQFMNQTRQALEMKMENPGSGSTSVMRPVMQPQVGSQQQGFLNAQMVAQRNRELISHHFRQQRMAMMM
QQQQQPQAFSPPPNVTASGSMDGALTGPPMAQVPPQQFPYPPNYGLSQQPDPAFSRVSSPPNPMMSSRIGASQN
PLMQHPQTATMYQSPEMKGWPSGSMARSSSFPQQQFSHQGNPATYSMMHMNGSSGHIGQMNINTMPMSGMPMGP
DQVSC
>259Cochlearius cochlearius|A0A7K8PJX3|Aves|SRC3
CMVKMSGLGENSLDSFTNESRKRKPLPCDTPGASLTCSGEKRRREQESKYIEELAELISANLSDIDNFNVKPDK
CAILKETVRQIRQIKEQGKAVSNDDDVQKADVSSTGQGVIDKDSLGPLLLQASTNEALALDGFLFVVNRDGNIV
FVSENVTQYLQYKQEDLVNTSVYGILHEEDRKDFLKNLPKSSVNGVAWTNETPRQKSHTFNCRMMVKTSHDLLE
DVSGHQDTRQRYETMQCFALSQPRAMMEEGEDLQSCMICVARRITTAERVFPTNPESFITRHDLSGKLVNIDTN
SLRSSMRPGFEDIIRRCIQRFLCHNDGQSWSNKRHYHEAYTQGHAETPLYRFSLADGTIVTAQTKSKLFRNPVT
NDRHGFVSTHFLQREQNGYRPNANSVGQNIRTPVAGNANSVGGLMNMSPGQGMPMQNRNYGMGDPNAVGQLSST
RYGGPGNIGPVNSGPGMQSSAYQNNSYGLTISSPPHGSPGLTSNQQNLMISPRNRGSPKMSSHQFSPVTGMHSP
MGSASNTGNSTFSSSSLSALQAISEGVGTSLLSTLSSPGPKLDNSPNMSIAQQSKTSTQDSKSPSGLYCEQNQV
ESSICQSNSRDLLSEKDSKEGNLEASESQRGPSESKGHKKLLQLLTCSSDERGHSTASNSPLDSNCKESSTNVT
SPSGVSSSTSGGVSSSSNMHGSLLQEKHRILHKLLQNGNSPAEVAKITAEATGKDTYHDASNTAPCGEGTVKQE
QLSPKKKENNALLRYLLDKDDVKDPLSKELKPKVESVDNKMGPCSSSTIPTSSQEKDMKIKTEPAEEMSGDLDN
LDAILGDLTSSEFYNNAISANGNNLGTKQAAFQGNALLGMRSPQSVQTTRPPFNRAMSLDSPVGSSAPARNVNA
FSVLQKQNLMGGSPRRIENQEIFGANLGFAPNRNVAMNQNQSGDWGLPNSKVNRLEPTSSASMLRPGPEFSASL
PRPTVGGSISGLPVRSNSIPGTRPMLQQQMIHMRPNEINIGMGGNPYGQPGPSNQPGSWPDNMLSMEQASRGSQ
NRQLVRNSLDDLLCPAPNMEGQNDERALLDQLHTLLSNTDVTGLEEIDRALGIPDLVNQGQALEPKQDSFQGQE
SSVMIDQKPSLYGQPYQGQGAAMPGGFSNIQGQQPSFNSVMNQMSQQSNFPLQSMHPRANVMRPRTNTPKQLRM
QLQQRLQGQQFMNQTRQALEMKMENPVSGNTSVMRPVMQPQVGSQQQGFLNAQMVAQRNRELISHHFRQQRMAM
MMQQQQQPQAFSPPPNVTASGSMDSALTGPPMAQVPPQQFPYPPNYGMSQQPDPTFSRVSSPPNPMMSSRIGPS
QNPMMQHPQTATMYQSPEMKGWPSGSMARSSSFPQQQFSHQGNPAAYSMMHMNGSSGHIGQMNINTMPMSGMPM
GPDQVSC

>260Nyctibius bracteatus|A0A7K8SFK5|Aves|SRC3
CMVKMSGLGENSLDSLTNESRKRKPLPCDTPGASLTCSGEKRRREQESKYIEELAELISANLSDIDNFNVKPDK
CAILKETVRQIRQIKEQGKAVSNDDDVQKADVSSTGQGVIDKDSLGPLLLQASTNWALDGFLFVVNRDGNIVFV
SENVTQYLQYKQEDLVNTSVYGILHEEDRKDFLKNLPKSSVNGVAWTNETPRQKSHTFNCRMMVKTSHDLLEDV
GSHQDTRQRYETMQCFALSQPRAMMEEGEDLQSCMICVARRITTAERVFPTNPESFITRHDLSGKLVNIDTNSL

RSSMRPGFEDIIRRCIQRFLCHNDGQSWSNKRHYHEAYTQGHAETPLYRFSLADGTIVTAQTKSKLFRNPVTND
RHGFVSTHFLQREQNGYRPNPNSVGQNVRTPVAGNTNSVGGLMNMSPGQGMPVQNRNYGMGDPNAVGQLSSTRY
GGPGNMGPVNSGPGMQSSAYQNNSYGLNISSPPHGSPGLTSNQQNLMISPRNRGSPKMSSHQFSPVTGMHSPMG
SASNTGNSTFSSSSLSALQAISEGVGSSLLSTLSSPGPKLDNSPNVSIAQQSKTSTQDSKSPSGLYCEQNQVES
SICQSNSRDLLSEKDSKEGNQEASESQRGPSESKGHKKLLQLLTCSSDERGHSTASNSPLDSNCKESSTNVTSP
SGVSSSTSGGVSSSSNMHGSLLQEKHRILHKLLQNGNSPAEVAKITAEATGKDTYHDASNAAPCGEGTVKQEQL
SPKKKENNALLRYLLDKDDVKDPLSKELKPKVESVDNKMGQCSSSTIPTSSQEKEMKIKTEPTEEMSGDLDNLD
AILGDLTSSEFYNNAMSANGNNLGTKQALFQGNALLGVRSPQSAQATRPPFNRAMSLDSPVGPSAPARNVNAFS
MLQKQNLMGGSPRMIENQENFAANLGFAPNRNVAMNQHQAGDWGLPNSKGNRLEPTSSASMLRPGPEFSASLPR
PAVGGSIGLPVRSNSIPGTRPMLQQQMIHMRPNEINIGMGGNPYGQPAPSTQPGSWPDNMLSMEQASRGSQNRP
LVRNSLDDLLCPAPNMEGQNDERALLDQLHTLLSNTDVTGLEEIDRALGIPDLVNQGQALEPKQDSFQGQESSV
MIDQKPSLYGQPYQGQGAAMPGGFSNIQGQQPSFNSVMNQMSQQSNFPLQSMHPRASVMRPRTNTPKQLRMQLQ
QRLQGQQFMNQTRQALEMKMENPVSGNTSVMRPVMQPQVGSQQQGFLNAQMVAQRNRELISHHFRQQRMAMMMQ
QQQQPQAFSPPPNVTASGSMDSALTGPPMAQVPPQQFPYPPNYGISQQPDPAFSRVSSPPNPMMSSRIGPSQNP
MMQHPQTATMYQSPEMKGWPSGSMARSSSFPQQQFSHQGNPAAYSMMHMNGSSGHIGQMNINTMPMSGMPMGPD
QVSC

>261Dromaius novaehollandiae|A0A7K9B6A2|Aves|SRC3
CVIKMSGLGENSLDSLTNESRKRKPLPCDTPGANLTCSGEKRRREQESKYIEELAELISANLSDIDNFNVKPDK
CAILKETVRQIRQIKEQGKAVSNDDDVQKADVSSTGQGVIDKDSLGPLLLQASTNYALDGFLFVVNRDGNIVFV
SENVTQYLQYKQEDLVNTSVYGILHEEDRKDFLKNLPKSSVNGVAWSNETPRQKSHTFNCRMMVKTSHDLLEDV
GSHQDTRQRYETMQCFALSQPRAMMEEGEDLQSCMICVARRITTVERVFPTNPESFITRHDLSGKLVNIDTNSL
RSSMRPGFEDTIRRCIQRFLCHNDGQSWSNKRHYHEAYTQGHAETPLYRFSLADGTIVTAQTKSKLFRNPVTND
RHGFVSTHFLQREQNGYRPNPNSVGQNIRAPVAGSTNSVGGVMNMSPGQGMPMQNRSYSMGDPSAVGQLSSARY
GGPGNMGPMNSGPGMQSSTYQNNNYGLNISSPPHGSPGLNSNQQNLMISPRNRGSPKMSSHQFSPVTGMHSPMG
SASNTGNSTFSSSSLSALQAISEGVGTSLLSTLSSPGPKLDNSPNMSIAQQSKTSTQDSKSPSGLYCEQNQVES
SVCQSNSRELLSEKDSKEGNLEASESQRGPSESKGHKKLLQLLTCSSDDRGHSTASNSPLDSNCKESSTNVTSP
SGVSSSTSGGVSSSSNMHGSLLQEKHRILHKLLQNGNSPAEVAKITAEATGKDTYHDASNTAPCGEGTVKQEQL
SPKKKENNALLRYLLDKDDIKDPLSKELKPKVEGVDNKMGQCSSSTLPTSSQEKEIKIKAEPTEEMSGDLDNLD
AILGDLTSSDFYNNAMSANGNSLGTKQALFPGTAPLSMRSPQAVQTARPPFSRAMSLDSPVGSSPPVRSVNAFS
MLQKQNLMGGSPRMIENQENFGANLGFGPNRNVAMNQHPSGDWGLPNSKVNRLEPTSSASMLRPGPEFNASLPR
AAVGGSVPGLPIRSNSVPGTRPMLQQQMIHMRPNEINIGMGGNPYGQPGPSNQPGSWPDNMLSMEQASRGSQNR
QLVRSSLDDLLCPAPNVEGQSDERALLDQLHTLLSNTDVTGLEEIDRALGIPDLVNQGQALEPKQDSFQGQESA
VMIDQKSSLYGQPYQGQGAAMPGGFSNIQGQQPSFNSVMNQMSQQSNFPLQSMHPRANVMRPRTNTPKQLRMQL
QQRLQGQQFMNQTRQALEMKMENPGSGSTSVMRPVMQPQVGSQQQGFLNAQMVAQRNRELISHHFRQQRMAMMM
QQQQQPQAFSPPPNVTASGSMDGALTGPPMAQVPPQQFPYPPNYGLSQQPDPAFSRVSSPPNPMMPSRIGASQN
PMMQHPQTATMYQSPEMKGWPSGSMARSSSFPQQQFSHQGNPATYSMMHMNGSSGHIGQMNINTMPMSGMPMGP
DQVSC
>262Hemiprocne comata|A0A7K9DGF6|Aves|SRC3
CMVKMSGLGENSLDSLTNESRKRKTLPCDTPGASLTCSGEKRRREQESKYIEELAELISANLSDIDNFNVKPDK
CAILKETVRQIRQIKEQGKAASNDDDVQKADVSSTGQGVIDKDSLGPLLLQAALDGFLFVVNRDGNIVFVSENV
TQYLQYKQEDLVNTSVYGILHEEDRKDFLKNLPKSSVNGVAWTNETPRQKSHTFNCRMMVKTSPDLLEDVGSHQ
DTRQRYETMQCFALSQPRAMMEEGEDLQSCMICVARRITTAERVFPTNPESFITRHDLSGKLVNIDTNSLRSSM
RPGFEDIIRRCIQRFLCHNDGQSWSNKRHYHEAYTQGHAETPLYRFSLADGTIVTAQTKSKLFRNPVTNDRHGF
ISTHFLQREQNGYRPNPSSVGQNIRTPVAGNTSSVGGLMNMSPGQGMAMQNRSYSLGDPSTAGQLSSARYGGPG
NMGPVNPGPGMQSSAYQNNSYGLNISSPPHGSPGLTSNQQNLMISPRNRGSPKMSSHQFSPVTGMHSPMGSASN
TSNSTFSSSSLSALQAISEGVGTSLLSTLSSPGPKLDNSPNMNIAQQSKTSAQDSKSPSGLYCEQNQVESSICQ
SNSRDLLSEKDSKEGSLEASENQRGPSESKGHKKLLQLLTCSSDERGHSTASNSPLESNCKESSTNVTSPSSGV
SSSTSGGVSSSSNMHGSLLQEKHRILHKLLQNGNSPAEVAKITAEATGKDTYHDASNTAPCGEGTVKQEQLSPK
KKENNALLRYLLDKDDVKDPLSKEVKPKVESVDNKMGQCSSSTIPTSSQEKDAKIKTEPTEEMSGDLDNLDAIL
GDLTSSEFYNNAMSANGNNLGTKQALFQGNALLGMRSPQSVQTTRPPFNRAVSLDSPMGSSAPGRNVNAFSMLQ
KQNLMGGSPRMVENQENFGPNLGFAPNRNVAMNQQQSGDWGLPNSKVNRLEPTSSASMLRPGPEFSASLPRPAV
GGSVPGLPVRSNSVPGARPMLQQQMIHMRSNEISIGMGGNPYGQAGPASQPGSWPDNLLSMEQASRGSQNRQLV
RNSLDDLLCPAPNMEGQNDERALLDQLHTLLSNTDVTGLEEIDRALGIPDLVNQGQALEPKQDSFQGQESSVMI

DQKPSLYGQPYQGQAAAMPGGFSNIQGQQPSFNSVMNQMNQQSNFPLQSIHPRANVMRPRSSTPKQLRMQLQQR
LQGQQFMNQTRQALEMKMENPVSGNTSVMRPVMQPQVGSQQQGFLNAQMVAQRNRELISHHFRQQRMAMMMQQQ
QQPQAFSPPPNVTASGSMDSALTGPPMAQVPPQQFSYPPSYGISQQPDPAFSRVSSPPNPMMSSRIGPSQNPMM
QHPQPTTMYQSPEMKGWPSGSMARSSSFPQQQFSHQGNPAAYSMMHMNGSTGHMGQMNMSSMPLAGMPMGPDQV
RC

>263Stercorarius parasiticus|A0A7K9FEW8|Aves|SRC3
CMFKMSGLGENSLDSLTNESRKRKPLPCDTPGPSLTCSGEKRRREQESKYIEELAELISANLSDIDNFNVKPDK
CAILKETVRQIRQIKEQGKAVSNDDDVQKADVSSTGQGVIDKDSLGPLLLQASALDGFLFVVNRDGNIVFVSEN
VTQYLQYKQEDLVNTSVYGILHEEDRKDFLKNLPKSSVNGVAWSNETPRQKSHTFNCRMMVKTSHDLLEDVGSH
QDTRQRYETMQCFALSQPRAMMEEGEDLQSCMICVARRITTAERVFPTNPESFITRHDLSGKLVNIDTNSLRSS
MRPGFEDIIRRCIQRFLCHNDGQSWSNKRHYHEAYTQGHAETPLYRFSLADGTIVTAQTKSKLFRNPVTNDRHG
FVSTHFLQREQNGYRPNPNSVGQNIRTPVAGNTNSVGGLMNMSPGQGMPMQNRNYGIGDPSAVGQLSSTRYGGP
GNMGPVNSGPGMQSSAYQNNSYGLNISSPPHGSPGLASNQQNLMISPRNRGSPKMSSHQFSPVTGMHSPMGSAS
NTSNSTFSSSSLSALQAISEGVGTSLLSTLSSPGPKLDNSPNMGIAQQSKTSTQDSKSPSGLYCEQNQVESSIC
QSNSRDLLSEKDSKEGNLEAPESQRGPSESKGHKKLLQLLTCSSDERGHSTASNSPLDSNCKESSTNVTSPSGV
SSSTSGGVSSSSSNMHGSLLQEKHRILHKLLQNGNSPAEVAKITAEATGKDTYHDASNTAPCGEGTVKQEQLSP
KKKENNALLRYLLDKDDVKDPLSKELKPKVESVDNKTGQCSSSAISTSSQEKEMKIKTEPAEEMSGDLDNLDAI
LGDLTSSEFYNNAMSANGNNLGTKQALFQGNALLGVRSPQSVTTTRPPFTRAMSLDSPVGSSAPVRNVNAFSML
QKQNLMGGSPRMIESQENFGANLGFAPNRNVAMNQHQSGEWGLPNSKVNRLEPTSSASMLRPGPEFSASLPRSS
VGGSMSGLPVRSNSIPGTRPMLQQQMIHMRPNEINIGMGGNPYGQPGPSNQPGSWPDNVLSMEQASRGSQNRQL
VRNSLDDLLCPPPNMEGQNDERALLDQLHTLLSNTDVTGLEEIDRALGIPDLVNQGQALEPKQDSFQGQESSVM
IDQKPSLYGQPYQGQGAAMPGSFSNIQGQQPTFNSVMNQMSQQSNFPLQSMHPRANVMRPRTNTPKQLRMQLQQ
RLQGQQFMNQTRQALEMKMENPVSGNTSVMRPVMQPQVGSQQQGFLNAQMVAQRNRELISHHFRQQRMAMMMQQ
QQQPQAFSPPPNVTASGSMDSALTGPPMAQVPPQQFPYPPNYGIGQQPDPAFSRVSSPPNPMMSSRIGASQNPM
MQHPQTAAMYQSPEMKGWPSGSMARSSSFPQQQFSHQGNPAAYSMMHMNGSSGHIGQMNINTMPMSGMPMGPDQ
VSR
>264Rostratula benghalensis|A0A7L0D1Q0|Aves|SRC3
CMFKMSGLGENSLDSLTNESRKRKPLPCDTPGANLTCSGEKRRREQESKYIEELAELISANLSDIDNFNVKPDK
CAILKETVRQIRQIKEQGKAVSNDDDVQKADVSSTGQGVIDKDSLGPLLLQASALDGFLFVVNRDGNIVFVSEN
VTQYLQYKQEDLVNTSVYGILHEEDRKDFLKNLPKSSVNGVAWTNETPRQKSHTFNCRMMVKTSHDLLEDVGSH
QDARQRYETMQCFALSQPRAMMEEGEDLQSCMICVARRITTAERAFLTNPESFITRHDLSGKLVNIDTNSLRSS
MRPGFEDIIRRCIQRFLCHNDGQSWSNKRHYHEAYTQGHAETPLYRFSLADGTIVTAQTKSKLFRNPVTNDRHG
FVSTHFLQREQNGYRPNPNPVGQNIRAPVAGNTNSGGGLMNMSPGQGVPMQNRSYGMGDPSAVGPLGSARYGGP
ANMGPVNSGPGMQPSAYQNNSYGLNISSPPHGSPGLTSNQQNLMISPRNRGSPKMSSHQFSPVTGMHSPMGSAS
NTGNSTFSSSSLSALQAISEGVGTSLLSTLSSPGPKLDNSPNMSIAQQSKTSTQDSKSPSGLYCEQNQVESSIC
QSNSRDLLSEKDSKEGNLETSESQRGPSESKGHKKLLQLLTCSSDERGHSTASNSPLDSNCKESSTNVTSPSGV
SSSTSGGVSSSSNMHGSLLQEKHRILHKLLQNGNSPAEVAKITAEATGKDTYHEASNTAPCGEGSVKQEQLSPK
KKENNALLRYLLDKDDVKDPLSKELKPKVESVDNKMGQCSSSTIPTSSQEKEMKIKTEPAEEMSGDLDNLDAIL
GDLTSSEFYNNAMSANGNNLGTKQALFQGNALLGVRSPQSAPMARPPFTRAMSLDSPVGSGAPMRNVNAFSMLQ
KQNLMGGSPRMIESQENFAANLGFAPNRNVAMNQLQAGDWGLPNSKLNRLEPTSSASALRAGPEFSASLPRAPL
GGSMPGLPARSNSIPGTRPVLQQQMVHMRPNEMNIAMGGSPYGQPVPSNQPGSWPDNMLSMEQASRGSQNRQLV
RNSLDDLLCPAPNMEGQTDERALLDQLHTLLSNTDVTGLEEIDRALGIPDLVNQGQALEPKQDSFQGQESSVML
DQKPSLYGQPYQGQGAAMPGSFSNIQGQQPTFNSVMNQMSQQSNFPLQSMHPRANVMRPRTNTPKQLRMQLQQR
LQGQQFMNQTRQPLEMKMENPVSNTSVMRPVMQPQVGSQQQGFLNAQMVAQRNRELISHHFRQQRMAMMMQQQQ
QPQAFSPPPNVTASGSMDSALTGPPMAQVPPQQFPYPPNYGISQQPDPAFGRVPSPPNPMMSSRIGASQNPMMQ
HPQSAAMYQAPEMKGWPSGSMARSSSFPQQQFSHQGNPAAYSMMHMNGSSGHIGQMNMNTMPMSGMPMGPDQVR
C
>265Trogon melanurus|A0A7L0E661|Aves|SRC3
CVVKMSGLGENSLDSLTNESRKRKPLPCDTPGASLTCSGEKRRREQESKYIEELAELISANLSDIDNFNVKPDK
CAILKETVRQIRQIKEQGKAVANDDDVQKADVSSTGQGVIDKDSLGPLLLQAALDGFLFVVNRDGNIVFVSENV
TQYLQYKQEDLVNTSVYGILHEEDRKDFLKNLPKSSVNGVAWTNETPRQKSHTFNCRMMVKPSHELLEDVGSHQ
DIRQRYETMQCFALSQPRAMMEEGEDLQSCMICVARRITTAERVYPTNPESFITRHDLSGKLVNIDTNSLRSSM
RPGFEDIIRRCIQRFLCHNDGQSWSNKRHYHEAYTQGHAETPLYRFSLADGTIVTAQTKSKLFRNPVTNDRHGF

VSTHFLQREQNGYRPNPNSAAQNIRTPVAGNTNSVGGLMSMSPGQGVPVQNRSYAVGDPSAVGQLSSTRYGGPG
NMGPVNSGPGMPSSAYQNNSYGLNMSSPPHGSPGLTSNQQNLMISPRNRGSPKMSSHQFSPVTGMHSPMGSASN
TGNSSFSSSSLSALQAISEGVGTSLLSTLSSPGPKLDNSPNMSIAQQSKPSSQDSKSPSGLYCEQNQVESSICQ
SNSRDLLGEKDNKEGNPEASESQRGPSESKGHKKLLQLLTCSSDERGHSTAVNSPLDSNCKESSTNVTSPSGVS
SSTSGGVSSSSNMHGSLLQEKHRILHKLLQNGNSPAEVAKITAEATGKDTYHDAGSAAPCGEGPVKQEQLSPKK
KENNALLRYLLDKDDVKDPLSKELKPKVESVDNKMGQCSSSTIPTSSQEKEMKIKTEPAEEMSGDLDNLDAILG
DLTSSEFYNNAMSANGNNLGTKQALFQGNTLLGMRSPQSVQTTRPPFSRAMSLDSPVGSSAPARNVNAFSMLQK
QNVMGGSPRMIENQENFGANLGFAPNRNVAMNQHQSGDWGLPTSKANRMEPTSSASMLRAGPEFSASLPRPTVG
SSMAGLPVRSNSLPGTRPMLQQQMMHMRPNEMSVGMGGNLYGQPAPSNQPGSWPDNMLSMEQASRASQNRQLVR
NSLDDLLCPAPNMEGQNDERALLDQLHTLLSNTDVTGLEEIDRALGIPDLVNQGQALEPKQDSFQGQESSVMID
QKPSLYGQAYQGQGAAMPGGFSNIQGQQPAFNSVMNQMSQQSSFPLQSMHPRANVMRPRTNTPKQLRMQLQQRL
QGQQFMNQTRQAIEMKMENAVSGNTSVMRPVMQPQVGSQQQGFLNAQMVAQRNRELISHHFRQQRMAMMMQQQQ
QPQAFSPPPNVTASGSMDSALTGPPMAQVPPQQFPYPPNYGISQQPDPAFSRVSSPPTPMMSSRIGPSQNPMMQ
HPQTATMYQSPEMKGWPSGSMARSSSFPQQQFSHQGTAAAYSMMHMNGSSGHLGQMNINAMPMSGMPMGPDQVS
C
>266Herpetotheres cachinnans|A0A7L0GGY8|Aves|SRC3
CMVKMSGLGENSLDSLTNESRKRKPLPCDTPGASLTCSGEKRRREQESKYIEELAELISANLSDIDNFNVKPDK
CAILKETVRQIRQIKEQGKAVSNDDDVQKADVSSTGQGVIDKDSLGPLLLQAALDGFLFVVNRDGNIVFVSENV
TQYLQYKQEDLVNTSVYAILHEEDRKDFLKNLPKSSVNGVTWTNEAPRQKSHTFNCRMMVKTSHDLLEDVGSHQ
DIRQRYETMQCFALSQPRAMMEEGEDLQSCMICVARRITTAERVFPTNPESFITRHDLSGKLVNIDTNSLRSSM
RPGFEDIIRRCIQRFLCHNDGQSWSNKRHYHEAYTQGHAETPLYRFSLADGTIVTAQTKSKLFRNPVTNDRHGF
VSTHFLQREQNGYRPNPNSVGQNIRTPVAGNTNSVGGLMNMSPGQGMPMQNRNYGMGDPSAVGQLSSTRYGGPG
NMGPVNSGPGMQSSAYQNNSYGLNISSPPHGSPGLTSSQQNLMISPRNRGSPKMSSHQFSPVTGMHSPMGSASN
TSNSTFSSSSLSALQAISEGVGTSLLSTLSSPGPKLDNSPNMSIAQQSKTSTQDSKSPSGLYCEQNQVESSICQ
SNSRDLLSEKDSKEGNLEASESQRGPSESKGHKKLLQLLTCSSDERGHSTASNSPLDSNCKESSTNVTSPSGVS
SSTSGGVSSSSNMHGSLLQEKHRILHKLLQNGNSPAEVAKITAEATGKDTYHDASNTATCGEGTVKQEQLSPKK
KENNALLRYLLDKDDVKDPLSKELKPKVESVDNKMGQCSSSTIPTSSQEKEMKIKTEPAEELSGDLDNLDAILG
DLTSSEFYSNAMSANGNNLGTKQALFQGNALLGMRSPQSVQPTRPPFNRAMSLDSPVGSSASVRSVNAFSMLQK
QNLMGGSPRMIENQENFGANLANAPNRNVMMNQHQSGDWGLPNSKVNRLEPPSSASMLRPGPEFSASLPRPAVG
SSMSGLPVRSNSIPGTRPMLQQQMIHMRPNEINIGMGGNPYGQPGPSNQPGSWPDNVLSMEQASRGSQNRQLVR
NSLDDLLCPAPNMEGQSDERALLDQLHTLLSNTDVTGLEEIDRALGIPDLVNQGQALEPKQDSFQGQESSVMID
QKPSVYGQPYQGQGAAMPGGFSNIQGQQPSFNSVMNQMSQQSNFPLQSMHPRANVMRPRTNTPKQLRMQLQQRL
QGQQFMNQTRQALEMKMENPVSGNTSVMRPVMQPQVGSQQQGFLNAQMVAQRNRELISHHFRQQRMAMMMQQQQ
QPQAFSPPPNVTASGSMDSALTGPPMAQVPPQQFPYPPNYGISQQPDTAFSRVSSPPNPMMSSRIGPSQNPMMQ
HPQTATMYQSPEMKGWPSGSMARSSSFPQQQFSHQGNPAAYSMMHMNGSSAHIGQMNINTMPMSGMPMGPDQVS
C
>267Arenaria interpres|A0A7L0HKT3|Aves|SRC3
CMFKMSGLGENSLDSLTNESRKRKPLPCDTPGASLTCSGEKRRREQESKYIEELAELISANLSDIDNFNVKPDK
CAILKETVRQIRQIKEQGKAVSSDDDVQKADVSSTGQGVIDKDSLGPLLLQASANPYGNKYLRQALDGFLFVVN
RDGNIVFVSENVTQYLQYKQEDLVNTSVYGILHEEDRKDFLKNLPKSSVNGVAWTNETPRQKSHTFNCRMMVKT
SHDLLEDVGSHQDARQRYETMQCFALSQPRAMMEEGEDLQSCMICVARRIATAERAFPTNPESFITRHDLSGKL
VNIDTNSLRSSMRPGFEDIIRRCIQRFLCHNDGQSWSNKRHYHEAYTQGHAETPLYRFSLADGTIVTAQTKSKL
FRNPVTNDRHGFVSTHFLQREQNGYRPNPNSVGQNIRTPVAGNTNSVGGLMNMSPGQGMPVQNRSYGMGDPSAV
GQLSSTRYGGPGNMGPVNSGPGMQSSAYQNNSYGLNISSPPHGSPGLTSNQQNLMISPRNRGSPKMSSHQFSPV
TGMHSPMGSASNTGNSTFSSSSLSALQAISEGVGTSLLSTLSSPGPKLDNSPNMSIAQQSKTSTQDSKSPSGLY
CEQNQVESSICQSNSRDLLSEKDSKEGNLETPESQRGPSESKGHKKLLQLLTCSSDERGHSTASNSPLDSNCKE
SSTNVTSPSGVSSSTSGGVSSSSNMHGSLLQEKHRILHKLLQNGNSPAEVAKITAEATGKDTYHDASNTAPCGE
GTVKQEQLSPKKKENNALLRYLLDKDDVKDPLSKELKPKVESVDNKMGQCSSSTIPTSSQEKEMKIKTEPAEEM
SGDLDNLDAILGDLTSSEFYNNAMSANGNNLGTKQALFQGNALLGVRSPQSVPTTRPPFTRAMSLDSPVGSSAP
VRNVNAFSMLQKQNLMGGSPRMIESQENFGANLASAPNRNVAMNQHQAGDWGLPNSKVSRLEPTSSASMLRAGP
EFSTSLPRPSVGGSMPGLPVRSNSIPGTRPVLQQQMVHMRPNEMNIGMGGNPYGQPGPPNQPGSWPDNMLSMDQ
ASRGSQNRQLVRNSLDDLLCPAPNMEGQTDERALLDQLHTLLSNTDVTGLEEIDRALGIPDLVNQGQALEPKQD
SFQGQESSVMLDQKPSLYGQPYQGQGAAMPGSFSNIQGQQPTFNSVMNQMSQQSNFPLQSMHPRANVMRPRTNT
PKQLRMQLQQRLQGQQFMNQTRQALEMKMENPVSGNTSVMRPVMQPQVGSQQGFLNAQMVAQRNRELISHHFRQ

QRMAMMMQQQQQPQAFSPPPNVTASGSMDSALTGPPMAQVAPQQFPYPPNYGISQQPDPAFSRVSSPPNPMMSS
RMGPSQNPMMQHPQTAAMYQSPEMKGWPSGSMARSSSFPQQQFSHQGNPAAYSMMHMNGSSGHIGQMNINTMPM
SGMPMGPDQVSC
>268Formicarius rufipectus|A0A7L0N7R9|Aves|SRC3
CLIKMSGLGENSLDSLTNESRKRKPLPCDTPAASLTCSGEKRRREQESKYIEELAELISANLSDIDNFNVKPDK
CAILKETVRQIRQIKDQGKAVSNDDDVQKADVSSTGQGVIDKDSLGPLLLQAALDGFLFVVNRDGNIVFVSENV
TQYLQYKQEDLVNTSVYGILHEEDRKDFLKNLPKSSVNGVAWTNETPRQRSHTFNCRMMVKSSHDLLEDVGSHQ
DVRQRYETMQCFALSQPRAMMEEGEDLQSCMICVARRIATAERVFPTNPESFITRHDLSGKLVNIDTNSLRSSM
RPGFEDIIRRCIQRFLCHNDGQSWSNKRHYHEAYTQGHAETPFYRFSLADGTIVTAQTKSKLFRNPVTNDRHGF
VSTHFLQREQNGYRTNPNSVGQNIRTPVAGNTNSVAGLMNMSPGQGMPVQNRNYGMGDPNAVSQLSSTRYGGPG
NMGPVTSGPGMQSSAYQNNNYGLNISSPPHGSPGLTSNQQNLMISPRNRGSPKMSSHQYSPVTGMHSPMGSASN
TSNSTFSSSSLSALQAISEGVGNSLLSTLSSPGPKLDNSPNVSITQQSKTSNQDSKSPSGLYCEQNQGESSICQ
SNSRDLLGEKDSKEGNLEASESQRGPSESKGHKKLLQLLTCSSDERGHSTASNSPLDSNCKESSTSVTSPSGVS
SSTSAGVSSSSNMHGSLLQEKHRILHKLLQNGNSPAEVAKITAEATGKDTYHDASNTPSCGEGIVKQEQLSPKK
KENNALLRYLLDKDDVKDPLSKELKPKVESVDSKMGQCTGSTIPTSSQEKEMKIKMEPAEEMNGDLDNLDAILG
DLTSSEFYSNAMAANGNNLGTKQALFQGNALLGVKSSQSVQITRPPFNRAMSLDSPVGSSPSVRNINAFSILQK
QNLMGENPRVVENQENFGANLGFTPSRNMAMNSHQSGDWSLPNSKVTRLEPTSSATMLRPGPEFSAALPRPSVG
GSVSGLSVRSNSLPGTRPMLQQPIIQMRPSEVNIGMGGNPYGQLGPSNQSGLWPDNVLTMEQVSRGSQNRQLVR
NSLDELLCSAPNMEGQNDERALLDQLHTLLSNTDVTSLEEIDRALGIPDLVSQGQALEPKQDSFQGQESSVMID
PKSSLYGQPYQGQGATMPGGFSNIQGQQPSFNSVMNQMSQPSNFPLQSLHPRASVMRPRTNTPKQLRMQLQQRL
QGQQFMNQTRQTLEMKMESPVSGNTSAMRPVMQPQVGSQQQGFLNAQMVAQRNRELISHHFRQQRMAMMMQQQH
QPQAFSPPPNVTATGSMDSALTGPSMAQVPSQQFPYPPNYGISQQPDPAFSRVSSPPNTMISSRIGPSQNPVMQ
HPQSATMYQSSEMKGWPSGSIARNSSFSQQQFSHQGNPAAYSVMHMNGSSGHIGQMSINTMPMSGMPMGPDQVN
F

>269Glaucidium brasilianum|A0A7L0RDC1|Aves|SRC3
CMVKMSGLGENSLDSLTNESRKRKPLPCDTPGASLTCSGEKRRREQESKYIEELAELISANLSDIDNFNVKPDK
CAILKETVRQIRQIKEQGKAVSSDDDVQKADVSSTGQGVIDKDSLGPLLLQASTNQALDGFLFVVNRDGNIVFV
SENVTQYLQYKQEDLVNTSVYGILHEEDRKDFLKNLPKSSVNGVAWTNETPRQKSHTFNCRMMVKTSHDLLEDV
GSHQDIRQRYETMQCFALSQPRAMMEEGEDLQSCMICVARRITTAERVFPTNPESFITRHDLSGKLVNIDTNSL
RSSMRPGFEDIIRRCIQRFLCHNDGQSWSNKRHYHEAYTQGHAETPLYRFSLADGTIVTAQTKSKLFRNPVTND
RHGFVSTHFLQREQNGYRPNPNPVGQNIRTPVAGNTNSVGGLMNMSPGQGMAMQNRNYGMGDPSAVGQLSSTRY
GGPGNMGPVNSGPGMQSSAYQNSGYGLNISSPPHGSPGLTSNQQNLMVSPRNRGSPKMSSHQFSPVTGMHSPMG
SASNTGNSTFSSSSLSALQAISEGVGTSLLSTLSSPGPKLDNSPNMSIAQQGKTSTQDSKSPSGLYCEQNQVES
SICQSNSRDLLSEKDNKEGNLESSESQRGPSESKGHKKLLQLLTCSSDERGHSTASNSPLDSNCKESSTNVTSP
SGVSSSTSGGVSSSSNMHGSLLQEKHRILHKLLQNGNSPAEVAKITAEATGKDTYHDASNTGPCGEGTVKQEQL
SPKKKENNALLRYLLDKDDVKDPLSKELKPKVESVDNKMGQCSSSTIPTSSQEKEMKIKTEPAEEMTGDLDNLD
AILGDLTSSEFYNNAMSANGNNLATKQALYQGNAILAGMRSPQSVQTTRPPFSRAMSLDSPVGSSAPARNVNAF
SMLQKQNLMGGSPRMIENQENFGSNLGFAPNRNVAISQHQSGDWGLPNSKVNRLEPTSSASMLRPGPEFSASLP
RPTVGGSMAGLPVRSNSIPGTRPMLQQQMIHMRPNEINIGMGGNPYGQPGPSNQPGSWPDNMLSMEQASRGSQN
RQLVRNSLDDLLCPAPNMEGQNDERALLDQLHTLLSNTDVTGLEEIDRALGIPDLVNQGQALEPKQDSFQGQES
SVMIDQKPSLYGQPYQGQGAAMPGGFTNIQGQQPSFNSVMNQMSQQSNFPLQSMHPRANVMRPRTNTPKQLRMQ
LQQRLQGQQFMNQTRQALEMKMENPVSGNTSVMRPVMQPQVGSQQQGFLNAQMVAQRNRELISHHFRQQRMAMM
MQQQQQPQAFSPPPNVTASGSMDSALTGPPMAQVPPQQFPYPPNYGLSQQPDPAFSRVSSPPNPMMSSRIGPSQ
NPMMQHPQTATMYQSPEMKGWPSGSMARSSSFPQQQFSHQGNPAAYSMMHMNGSSGHIGQMNINTMPMSGMPMG
PDQVNC
>270Podilymbus podiceps|A0A7L0SI47|Aves|SRC3
CMVKMSGLGENSLDSLTNESRKRKPLPCDTPGASLTCSGEKRRREQESKYIEELAELISANLSDIDNFNVKPDK
CAILKETVRQIRQIKEQGKAVSNDDDVQKADVSSTGQGVIDKDSLGPLLLQASTNQALALDGFLFVVNRDGNIV
FVSENVTQYLQYKQEDLVNTSVYGILHEEDRKDFLKNLPKSSVNGVAWTNETPRQKSHTFNCRMMVKTSHDLLE
DVGSHQDTRQRYETMQCFALSQPRAMMEEGEDLQSCMICVARRITTAERVFPTNPESFITRHDLSGKLVNIDTN
SLRSSMRPGFEDIIRRCIQRFLCHNDGQSWSNKRHYHEAYTQGHAETPLYRFSLADGTIVTAQTKSKLFRNPVT
NDRHGFVSTHFLQREQNGYRPNPSSVGQNIRTPVAGNTNSVGGLMNMSPGQGMPMPNRNYGMGDPSAVGQLSNT
RYGGPGNMGPVNSGTGMQSSAYQNNSYGLNISSPPHGSPGLTSNQQNLMISPRNRGSPKMSSHQFSPVTGMHSP

MGSASNTGNSTFSSSSLSALQAISEGVGTSLLSTLSSPGPKLDNSPNMSIGQQSKTSTQDSKSPPGLYCEQNQV
ESSICQSNSRDLLSEKESKEGNLEAPESQRGPSESKGHKKLLQLLTCSSDERGHSTASNSPLDSNCKESSTNVT
SPSGVSSSTSGGVSSSSNVHGSLLQEKHRILHKLLQNGNSPAEVAKITAEATGKDTYHDASNTATCGEGTVKQE
QLSPKKKENNALLRYLLDKDDVKDPLSKELKPKVEAVDNKMGQCSSSTIPTSSQEKEMKIKTEPTEEMSGDLDN
LDAILGDLTSSEFYNNAMSANGNNLGTKQALFQGNALLGMRSPQSVQTPRPPFNRAMSLDSPVGSSAPARNVNT
FSMLQKQNLMGGSPRMIENQDSFGANLGFAPNRNVAMNQHQSGDWGLPNSKVNRLEPTSSASMLRPGPEFSASL
PRATVGGSMAGLPVRSNSIPGTRPMLQQQMIHMRPNEINIGMGGNPYGQPGPSNQPGSWPDNMLSMEQASRGSQ
NRQLVRNSLDDLLCPAPNMEGQNDERALLDQLHTLLSNTDVTGLEEIDRALGIPDLVNQGQALEPKQDSFQGQE
SSVMIDQKSSLYGQPYQGQGAAMPGGFSSIQGQQPSFNSVMNQMSQQSNFPLQSMHPRANVMRPRTNTPKQLRM
QLQQRLQGQQFMNQTRQALEMKMENPVSGNTSVMRPVMQPQVGSQQQGFLNAQMVAQRNRELISHHFRQQRMAM
MMQQQQQPQAFSPPPNVTASGSMDSALSGPPMAQVPPQQFPYPPNYGISQQPDPAFSRVSSPPNTMMSSRIGPS
QNPMMQHPQTATMYQSPEMKGWPTGSMARSSSFPQQQFSHQGNPAAYSMMHMNGSSGHIGQMNINTMPMSGMPM
GPDQVRC

>271Chordeiles acutipennis|A0A7L0TKX1|Aves|SRC3
CMVRMSGLGENSLDSLTNESRKRKPLPCDTPGASLTCSGEKRRREQESKYIEELAELISANLSDIDNFNVKPDK
CAILKETVRQIRQIKEQGKAVSNDDDVQKADVSSTGQGVIDKDSLGPLLLQAALDGFLFVVNRDGNIVFVSENV
TQYLQYKQEDLVNTSVYGILHEEDRKDFLKNLPKSAVNGVAWTNETPRQKSHTFNCRMLVKTSHDLLEDVGSHQ
DIRQRYETMQCFALSQPRAMMEEGEDLQSCMICVARRITTAERVFPTNPESFITRHDLSGKLVNIDTNSLRSSM
RPGFEDIIRRCIQRFLCHNDGQSWSNKRHYHEAYTQGHAETPLYRFSLADGTIVTAQTKSKLFRNPVTNDRHGF
VSTHFLQREQNGYRPNPNSVGQNMRTPVAGNTNSVGGLMNMSPGQGMPVQNRNYGMGDPNAVGQLSSTRYGGPG
NMGPVNSGPGMQSSAYQNNSYGLNISSPPHGSPGLTSNQQNLMISPRNRGSPKMNSHQFSPVTGMHSPMGSASN
TSNSTFSSSSLSALQAISEGVGTSLLSTLSSPGPKLDNSPNVSIAQQSKTSTQDSKSPSSLYCEQNQVESSICQ
SNSRDLLSEKDSKEGNLEASESQRGPSESKGHKKLLQLLTCSSDERGHSTASNSPLDSNCKESSTNVTSPSGVS
SSTSGGVSSSSNMHGSLLQEKHRILHKLLQNGNSPAEVAKITAEATGKDTYHDTSNTAPCGEGTVKQEQLSPKK
KENNALLRYLLDKDDVKDPLSKELKPKVESVDNKMGQCSSSTIPTSSQEKEMKIKTEPTEEMSGDLDNLDAILG
DLTSSEFYNNAMTANGNNLGTKQALFPGNALSGMRSPQSVQNTRPPFNRAMSLDSPAVSSAPVRNVNAFSMLQK
QNLMGGSPRMIENQENFGANLGFAPNRTVAMNQHQSGDWGLPNSKVNRLEPTSSASMLRPGAEFSASLPRATVG
GSMSGLPVRSNSIPGTRPMLQQQMIHMRPNEINIGMGGNPYGQPGPSNQPGSWPDNMLSMEQTSRGSQNRQLVR
NSLDDLLCPAPNMEGQNDERALLDQLHTLLSNTDVTSLEEIDRALGIPDLVNQGQALEPKQDAFQGQESSVMID
QKPSLYGQPYQGQGTAMPGGFSTIQGQQPSFNAVMNQMSQQSNFPLQSMHPRANVMRPRTNTPKQLRMQLQQRL
QGQQFMNQTRQALEMKMENPVSGNTSVMRPVMQPQVGSQQQGFLNAQMVAQRNRELISHHFRQQRMAMMMQQQQ
QPQAFSPPPNVTASGSMDSALTGPPMAQVPPQQFSYSPNYGISQQPDPAFSRVSSPPNPMMSSRIGPSQNPMMQ
HPQTATMYQSPEMKGWPSGTMARSSSFPQQQFSHQGNPAAYSMMHMNGSSGHIGQMNINTMPMSGMPMGPDQVS
C
>272Himantopus himantopus|A0A7L1LMV6|Aves|SRC3
CMVKMSGLGENSLDNLTNESRKRKPLPCDTPGASLTCSGEKRRREQESKYIEELAELISANLSDIDNFNVKPDK
CAILKETVRQIRQIKEQGKAVSNDDDVQKADVSSTGQGVIDKDSLGPLLLQASALDGFLFVVNRDGNIVFVSEN
VTQYLQYKQEDLVNTSVYGILHEEDRKDFLKNLPKSSVNGVAWTNEAPRQKSHTFNCRMMVKTSHDLLEDVGSH
QDTRQRYETMQCFALSQPRAMMEEGEDLQSCMICVARRITTAERVFPTNPESFITRHDLSGKLVNIDTNSLRSS
MRPGFEDIIRRCIQRFLCHNDGQSWSNKRHYHEAYTQGHAETPLYRFSLADGTIVTAQTKSKLFRNPVTNDRHG
FVSTHFLQREQNGYRPNPNSVGQNIRTPVAGNTNSVGGLMNMSPGQGMPMQNRSYGIGDPNAVGQLSSTRYGGP
GNMGPVNSGPGMQSSAYQNNSYGLNISSPPHGSPGLTSNQQNLMISPRNRGSPKMSSHQFSPVTGMHSPMGSAS
NTSNSTFSSSSLSALQAISEGVGTSLLSTLSSPGPKLDNSPNMSIAQQSKTSTQDSKSPSGLYCEQNQVESSIC
QSNSRDLLSEKDSKEGNVEASESQRGPSESKGHKKLLQLLTCSSDERGHSTASNSPLDSNCKESSTNVTSPSGV
SSSTSGGVSSSSNVHGSLLQEKHRILHKLLQNGNSPAEVAKITAEATGKDPYHDASNTAPCGEGTVKQEQLSPK
KKENNALLRYLLDKDDVKDPLSKELKPKVESVDNKMGQCSSSTIPTSSQEKEMKIKTEPVEEISGDLDNLDAIL
GDLTSSEFYNNPMSANGNNLGTKQALFQGNALLGARSPQSVPTTRPPFTRAMSLDSPVGSSAPVRNVNAFSTLQ
KQNLMGGSPRMIENQENFGANLGFAPNRNVAMTQHQSGDWGLPNSKVNRLEPTSSASMLRPGPEFSASLPRPTV
GGSMSGLPVRSNSIPGTRPMLQPQMIHMRPNEMNIGMGGNPYGQPGPSNQPGSWPDNMLSMEQASRGSQNRQLV
RNSLDDLLCPAPNMEGQNDERALLDQLHTLLSNTDVTGLEEIDRALGIPDLVNQGQALEPKQDSFQGQESSVLI
DQKPSLYGQPYQGQGAAMPGSFSNIQGQQPTFNSVMNQMSQQSNFPLQSMHPRANVMRPRTNTPKQLRMQLQQR
LQGQQFMNQTRQALEMKMENPVSGNTSVMRPVMQPQVGSQQQGFLNAQMVAQRNRELISHHFRQQRMAMMMQQQ
QQPQAFSPPPNVTASGSMDSALTGPPMAQVAPQQFPYPPNYGISQQPDPAFSRVSSPPNPMMSSRIGPSQNPMM

QHPQTATMYQSPEMKGWPSGSMARSSSFPQQQFSHQGNPAAYSMMHMNGGSGHIGQMNINTMPMSGMPMGPDQV
SC
>273Pandion haliaetus|A0A7L2WK28|Aves|SRC3
MSGLGDSSTDPANPDSRKRKGSPCDTAQSNEKRRREQENKYLEELAELLSANIGDIDTLSVKPDKCKILKKTVD
QIQQMKRLEQEKAADDEVQKSDISSSSQGVIEKESLGPLLLEALDGFFFVVNREGRIVFVSENVTSYLGYNQEE
LMNTSVYSILHVGDHAEFVKNLLPKSLVNGVPWPQEATRRNSHTFSCRMLIRPPDEPGAENQEARQRYEVMQCF
TVSQPKSFKEEGEDFQSCLICIARRLPRPAAVTPSESFVTKQDTTGKIISIDTSSLRAAGRTGWEDLVRKCIYA
FFQPQGREPSYAKQLFQEVMTRGTAFSPSYRFTLSDGTVLSAHTKCKLCYPSSPEMQPFIMGIHVIDRDHGMLS
PQENTNSTMSLPRVSPSLNPSISPGQGMALPPSIPPSNGSMLATNVNQQPGAGLHNSNSSTSQTSFGCSPGNQI
GANVALSQGQAGPQNSNHTLNLNSSPMNSPGITPPQFMSPRHRVSPGLVPRPRVPSNPFSPTMPTMHSPVGMAN
SGSGGSCGTSGTRPFPSAPINSMQGLTEGPSTPVGYSTPPPVLRQLSSQSSPGRLAMQPMKAESKDSKEIASIL
SEMIQSDGGSGDGKPLDSGMLHAGDRLSEGDSKCSQATSHKLVQLLATTAEQQLRHADADTSCKDSLACAGTAG
TLASSNPPSSTCPSSHSSLTERHKILHRLLQEGSPSDITTLAMEHEKKENAPNPTTTPTAQTQLPGTPDIKLEP
DALKKKDVKDHQLLRYLLDKDEKELAAAPALTLDDVKVKVEKTEQMEPCNTAPVPLAKPPATEEVKLETQGQFA
AELEQLDQLLPSLEKAAQLPSLCGPERSESSVTSVAVKPEMLAAPLQPSTAARAPTGLNPMNSGQGVQAGRTPF
EFCDPLEPAQLRPVPCPASNGSSPHTRGPSQQGRGTVTAPNPFPPDTGMPELEMSVPDHQFGQPAMGEQIPWTD
NSMAAMNRGVLNKPEDQCITSQLDELLCPPTTPEGRNDEKALLEQLVSFLSGKDETELAELDRALGIDKLVQGG
GLEALTERFQPQQATPPLMMEQKPGMYPQPYSSASPTTNLPGAFPGMVRQKPSFGPMPVQVPPPRGAFPTNMAM
QPRQTLNRPPTAPNQLRLQLQQRLQGQQQLIHQNRQAILNQFAASSPVGINMRAGMQQQITPQPPLNAQMLAQR
QRELYSQQHRQRQLMQQRAILMRQQSFGNNLPPSAGLPVQIGAARLPQAPPQQFPYPPNYGTTPGNPPTSTSPF
SPLASNPEAALANRSSMVNRGMMGGVGGQFGAGMTPQMQQNIFQYSGSGMGQQSEPAFAPSLSPSSPLMSPQLP
PSQSPMLQPAPPTPGYQSPDMKTWQQGAMGSSNVFSQAGQSQPAPAQQGMYNNMSITVSMAGGNTNVQNMNPMA
GQMQMNSLQMPGMNSMCSEQVNDPALRPAGLYCNQLSSTDLLKTEADGAQQVQQVQVFADVQCTVNLVGDPYLN
PPAPLGTPKNAAGPPPPPGQQKSLLQQLLTE
>274Syrrhaptes paradoxus|A0A7L3BEG5|Aves|SRC3
CVVKMSGLGENSLDSLTNESRKRKPLPCDTPGASLTCSGEKRRREQESKYIEELAELISANLSDIDNFNVKPDK
CAILKETVRQIRQIKEQGKAVSNDDDVQKADVSSTGQGVIDKDSLGPLLLQAALDGFLFVVNRDGNIVFVSENV
TQYLQYKQEDLVNTSVYGILHEEDRKDFLKNLPKSSVNGVAWTNETPRQKSHTFNCRMMVKTSHDLLEDVGSHQ
DTRQRYETMQCFALSQPRAMMEEGEDLQSCMICVARRITTAERVFPTNPESFITRHDLSGKLVNIDTNSLRSSM
RPGFEDIIRRCIQRFLCHNDGQSWSNKRHYHEAYTQGHAETPLYRFSLADGTIVTAQTKSKLFRNPVTNDRHGF
VSTHFLHREQNGYRPNPNSVGQNIRTPVPGNTNSVGGLMNMSPGQGMPVQNRNYGMGDPNAVGQLSSTRYGGPG
NMGPVNSGPAMQSSAYQNNSYGLNINSPPHGSPGLTSNQQNLMISPRNRGSPKMSSHQFSPVTGMHSPMGSASN
TGNSSFSSSSLSALQAISEGVGTSLLSTLSSPGPKLDNSPNMSIAQQSKTSAQDSKSPSGLYCEQNQVESSICQ
SNSRDLLSEKDSKEGNVEASESQRGPSESKGHKKLLQLLTCSSDERGHSTASNSPLDSNCKESSTNVTSPSGVS
SSTSGGVSSSSNMHGSLLQEKHRILHKLLQNGNSPAEVAKITAEATGKDTYLDASNTAPCGEGTVKQEQLSPKK
KENNALLRYLLDKDDVKDPLSKDLKPKVESVDNKMGQCSSSTIPTSSQEKEMKIKTEPAEEMSGDLDNLDAILG
DLTSSEFYNNAMSANGNNLGTKQALFQGNALLGMRSPQSVQTARPPFNRSMSLDSPVGSSAPARNVNAFSMLQK
QNLMGGSPRMIENQENFGANLGFAPNRNVAMSQHQTTPGDWGLPNSKVNRLEPTSSASMLRPGPEYDASLPRPA
VGGSLSGLPVRSNSIPGTRPMLQQQMMHMRPNEINLGMGGNPYGQPGPSNQPGSWPDNMLSMEQASRGSQNRQL
VRNSLDDLLCPAPNMEGQNDERALLDQLHTLLSNTDVTGLEEIDRALGIPDLVNQGQALEPKQDSFQGQESSVM
IDQKPSLYGQPYQGQGAAMPGGFSNIQGQQPSFNSVMNQMSQQSNFPLQSMHPRANVMRPRTNTPKQLRMQLQQ
RLQGQQFMNQTRQALEMKMENTVSGNTSVMRPVMQPQVGSQQQGFLNAQMVAQRNRELISHHFRQQRMAMMMQQ
QQQPQAFSPPPNVTASGSMDTALTGPPMAQVPPQQFPYPPNYGISQQPDPAFSRVSSPPNPMMSSRIGPSQNPM
MQHPQAAAMYQSPEMKGWPSGSMARSSSFPQQQFSHQGNPAAYSMMHMNGSSGHIGQMNINTMPMAGMPMGPDQ
VSC
>275Pelecanoides urinatrix|A0A7L3CD50|Aves|SRC3
MVKMSGLGENSLDSLTNESRKRKPLPCDTPGASLTCSGEKRRREQESKYIEELAELISANLSDIDNFNVKPDKC
AILKETVRQIRQIKEQGKSVSNDDDVQKADVSSTGQGVIDKDSLGPLLLQAALDGFLFVVNRDGNIVFVSENVT
QYLQYKQEDLVNTSVYGILHEEDRKDFLKNLPKSSVNGVAWTNETPRQKSHTFNCRMMVKTSHDLLEDVGSHQD
TRQRYETMQCFALSQPRAMMEEGEDLQSCMICVARRITTAERVFPTNPESFITRHDLSGKLVNIDTNSLRSSMR
PGFEDIIRRCIQRFLCHNDGQSWSNKRHYHEAYTQGHAETPLYRFSLADGTIVTAQTKSKLFRNPVTNDRHGFV
STHFLQREQNGYRPNPNSTGQNIRTPVAGNTNSVGGLMNMSPGQGMPMQNRNYGMGDPNAVGQLSSTRYGGPGS
MGPATSGPGIQSSAYQNNSYGLNISSPPHGSPGLTSNQQNLMISPRNRGSPKMSSHQFSPVTGMHSPMGSASNT
GNSTFSSSSLSALQAISEGVGTSLLSTLSSPGPKLDNSPNMSIAQQSKTSTQDSKSPSGLYCEQNQVESSICQS

NSRDLLSEKDSKEGNLEVSESQRGPSESKGHKKLLQLLTCSSDERGNSTASNSPLDSNCKESSTNVTSPSGVSS
STSGGVSSSSNMHGSLLQEKHRILHKLLQNGNSPAEVAKITAEATGKDTYHDPSNTAPCGEGTVKQEQLSPKKK
ENNALLRYLLDKDDVKDSLSKELKPKVESVDNKMGQCSSSTIPTSSQEKEMKIKTEPAEEMSGDLDNLDAILGD
LTSSEFYNNAMSANGNNLGTKQALFQGNALLGMRSPQSVQTTRPPFNRAMSLDSPVGSSAPVRNVNAFSMLQKQ
NLMGGSPRRIENQENFGANLGFAPNRNVAMNQHQSGDWGLPNSKVNRLEPTSSASMLRPGPEFSASLPRPTVGG
SMSGLPVRSNSIPGTRPGLQQQMIHMRPNEINIGMGGNLYGQPGPSNQPGSWPDNMLSMEQASRGSQNRQLVRN
SLDDLLCPAPNMESQNDERALLDQLHTLLSNTDVTGLEEIDRALGIPDLVNQGQALEPKQDSFQGQESSVMIDQ
KPSLYGQPYQGQGTAMPGGFSNIQGQQPSFNSVMNQMSQQSNFPLQSMHPRANVMRPRTNTPKQLRMQLQQRLQ
GQQFMNQTRQTLDMKMENPVSGNTSVMRPVMQPQVGSQQQGFLNAQMVAQRNRELISHHFRQQRMAMMMQQQQQ
PQAFSPPPNVTASGNMDSALTGPPMAQVPPQQFPYPPNYGISQQPDPAFSRVSSPPNPMMSSRIGPSQNPMMQH
PQTATMYQSPEMKGWPSGSVARSSSFPQQQFSHQGNPAVYSMMHMNGSSGHIGQMNINTMPMSGMPMGPDQVSC
>276Pluvianellus socialis|A0A7L3D838|Aves|SRC3
CMVKMSGLGENSLDSLTSESRKRKPLPCDTPGASLTCSGEKRRREQESKYIEELAELISANLSDIDNFNVKPDK
CAILKETVRQIRQIKEQGKAVSNDDDVQKADVSSTGQGVIDKDSLGPLLLQASALDGFLFVVNRDGNIVFVSEN
VTQYLQYKQEDLVNTSVYGILHEEDRKDFLKNLPKSSVNGVAWSNETPRQKSHTFNCRMMVKTSHDLLEDVGSH
QDARQRYETMQCFALSQPRAMMEEGEDLQSCMICVARRITTAERVFPTNPESFITRHDLSGKLVNIDTNSLRSS
MRPGFEDIIRRCIQRFLCHNDGQSWSNKRHYHEAYTQGHAETPLYRFSLADGTIVTAQTKSKLFRNPVTNDRHG
FVSTHFLQREQNGYRANPTSVGQNIRTPVAGNTNSVGGLMNMSPGQGMSMQNRNYGMGDPSAVGQLSTTRYGGP
GSMGPVNSGPGMQPAAYQNSSYGLNISSPPHGSPGLTSNQQNLMISPRNRGSPKMSSHQFSPVTGMHSPMGSAT
STGNSTFSSSSLSALQAISEGVGTSLLSTLSSPGPKLDNSPNMNIAQQGKTSTQDSKSPSGLYCEQNQVESSIC
QSNSRDLLSEKDSKEGNLEASESQRGPSESKGHKKLLQLLTCSSDERGHSTASNSPLDSNCKESSTNVTSPSGV
SSSTSGGVSSSSNMHGSLLQEKHRILHKLLQNGNSPAEVAKITAEATGKDTYHDAGNTAPCGEGAVKQEQLSPK
KKENNALLRYLLDKDDVKDPLSKELKPKVESVDNKMGQCSSSTIPTSSQEKDMKIKTEPAEEMSGDLDNLDAIL
GDLTSSEFYNNAMSANGNNLGTKQALFQGNALLGTRSPQSAPTTRPPFTRAMSLDSPVGSSAPVRNVNAFSMLQ
KQNLMGGSPRMIENQENFGANLGSAPNRNVALNQHQSGDWGLPNSKANRLEPTSSASMLRPGPEFSASLPRATV
GGSVSGLPVRSNSIPGTRPMLQQQMIHMRPNEINLGMGGNPYGQPGPSNQPGSWPDNMLSMEQASRGSQNRQLV
RNSLDDLLCPAPNMEGQNDERALLDQLHTLLSNTDVTGLEEIDRALGIPDLVNQGQALEPKQDSFQGQESSVMI
DQKPSLYGQPYQGQGTAMPGSFSNIQGQQPTFNSVMNQMSQQSNFPLQSMHPRANVMRPRTNTPKQLRMQLQQR
LQGQQFMNQTRQALEMKMENPVSGNTSVMRPVMQPQVGSQQQGFLNAQMVAQRNRELISHHFRQQRMAMMMQQQ
QQPQAFSPPPNVTAAGSMDSALTGPPMAQVPPQQFPYPPNYGISQQPDPAFSRVSSPPNPMMSSRIGPSQNPMM
QHPQTATMYQSPEMKGWPSGSMARSSSFPQQQFSHQGNAAAYGMMHMNGSTGHMGQMNINTMPMAGMPMAPDQV
SC
>277Thalassarche chlororhynchos|A0A7L3JIM2|Aves|SRC3
MSGLGDSSTDPANPDSRKRKGSPCDTAQSNEKRRREQENKYLEELAELLSANIGDIDTLSVKPDKCKILKKTVD
QIQQMKRLEQEKAADDEVQKSDISSSSQGVIEKESLGPLLLEALDGFFFVVNREGRIVFVSENVTSYLGYNQEE
LMNTSVYSILHVGDHAEFVKNLLPKSLVNGVPWPQEATRRNSHTFSCRMLIRPPDEPGAENQEARQRYEVMQCF
TVSQPKSFKEEGEDFQSCLICIARRLPRPAAVTPSESFVTKQDTTGKIISIDTSSLRAAGRTGWEDLVRKCIYA
FFQPQGREPSYAKQLFQEVMTRGTAFSPSYRFTLSDGTVLSAHTKCKLCYPSSPEMQPFIMGIHVIDRDHGMLS
PQENTNSTMSLPRVSPSLNPSISPGQGMALPPSIPPSNGSMLATNVNQQPGAGLHNSNSSTSQTSFGCSPGNQI
GANVALSQGQAGPQNSNHTLNLNSSPMNSPGITPPQFMSPRHRVSPGLVPRPRVPSNPFSPTMPTMHSPVGMAN
SGSGGGSGTGGTRPFPSAPINSMQGLTEGPSTPVGYSTPPPVLRQLSSQSSPGRLAMQPMKAESKDSKEIASIL
SEMIQSDGGSGDGKPLDSGMLHANDRLSEGDSKCSQATSHKLVQLLATTAEQQLRHADADTSCKDSLACAGTAG
TLASGNPPSSTCPSSHSSLTERHKILHRLLQEGSPSDITTLAMEHEKKENTPNPATTPTAQSQLPGTPDIKLEP
DTLKKKDVKDHQLLRYLLDKDEKELAAAPALSLDDVKVKVEKTEQMEPCNTAPVPLAKPPATEEVKLETQGQFA
AELEQLDQLLPSLEKAAQLPGLCGPERNESGVAGVTVKPEMLAAPLQPSTAARAPTGLNPMNSGQGVQAGRTPF
EFCDPLEPAQLRPVPCPTSNGSSPHARGPSQQGRGTVTAPNPFPPDTGMPELEMGVPDHQFGQPGMGEQIPWTD
NSMAAMNRGALNKPEDQCITSQLDELLCPPTTPEGRNDEKALLEQLVSFLSGKDETELAELDRALGIDKLVQGG
GLEALTERFQPQQATPPLMMEQKPGMYPQPYSSASPTTNLPGAFPGMVRQKPSFGPMPVQVPPPRGAFPTSMAM
QPRQTLNRPPTAPNQLRLQLQQRLQGQQQLIHQNRQAILNQFAANSPVGINMRAGMQQQITPQPPLNAQMLAQR
QRELYSQQHRQRQLMQQRAILMRQQSFGNNLPPSAGLPVQIGAARLPQAPPQQFPYPPNYGTTPGNPPTSTSPF
SPLASNPEAALANRSSMVNRGMMGGVGGQFGAGMTPQMQQNIFQYSGSGMGQQSEPAFAPSLSPSSPLMSPQLP
PSQSPMLQPAPPTPGYQSPDMKTWQQGAMGNSNVFSQAGQSQPAPAQQGMYNNMSITVSMAGGNTNVQNMNPMA
GQMQMNSLQMPGMNSMCSEQVNDPALRPAGLYCNQLSSTDLLKTEADGAQQVQQVQVFADVQCTVNLVGDPSLT
PPAPLGTPKNAPGPPPPPGQQKSLLQQLLTE

>278Xiphorhynchus elegans|A0A7L3NTS3|Aves|SRC3
CVVKMSGLGENSSDSLTNESRKRKPLPYDTPGASLTCSGEKRRREQESKYIEELAELISANLSDIDNFNVKPDK
CAILKETVRQIRQIKDQGKAVSNDDDVQKADVSSTGQGVIDKDSLGPLLLQAALDGFLFVVNRDGNIVFVSENV
TQYLQYKQEDLVNTSVYGILHEEDRKDFLKNLPKSSVNGVAWTNETPRQKSHTFNCRMMVKSSQDLLEDVGSQQ
DVHQRYETMQCFALSQPRAMMEEGEDLQSCMICVARRIVAAERMFPTNPESFITRHDLSGKLVNIDTNSLRSSM
RPGFEDIIRRCIQRFLCHNDGQSWSNKRHYHEAYTQGHAETPFYRFSLADGTIVTAQTKSKLFRNPVTNDRHGF
VSTHFLQREQNGYRPNPNSVGQNIRTPVAGNTNSVGGLMNMSPGQGMPVQNRNYVMGDPNAAGQLNSTRYGGPG
SMGPVTSGPGMQSSAYQSNSYGLNISSPPHGSPGLTSNQQNLMISPRNRGSPKMSSHQYSPVTGMHSPMGSASN
TSNSTFSSSSLSALQAISEGVGNSLLSTLSSPGPKLDNSPNVNITQQSKTSNQDSKSPSGLYCEQNQVESSICQ
SNSRDLLSEKDSKEANLEASESQRGPSESKGHKKLLQLLTCSSDERGHSTTSNPPLDSNCKESSTSVTSPSGVS
SSTSAGVSSSSNVHGSLLQEKHRILHKLLQNGNSPAEVAKITAEATGKDTYHDASNTASCGEGIVKQEQLSPKK
KENNALLRYLLDKDDIKDPLSKELKPKVESVDSKMGQCTSSAVPTSSQEKEMKIKMEPAEEMNEDLDNLDAILG
DLTSSEFYNNAMAANGNNLGTKQAFFQGNALLGVKSSQSITRPPFNRAISLDSPVSSSPSVRNVNAFSMFQKQN
LMGESPRMIENQENFGANLGFAPSRTVAMTQHQAGDWALPNSRGTRLDPAGSAALLRAGAEFGAALGRPAGGSV
PGLPGRSSSTPGTRPGLPQPLTHMRPNEINIGMGGNPYGQPGPSNQPGLWPDNVLSVEQMSRGSQNRQLVRNSL
DDLLCSAPNMEGQNDERALLDQLHTLLSNTDATGLEEIDRALGIPDLVSQGQALEPKQDSFQGQESSIMIDPKS
SLYGQPYQGQGATMPGGFSNMQGQQPSFNSVMNQMSQPSNFPLQSMHPRASAMRPRTSTPKQLRMQLQQRLQGQ
QFMNQTRQTLEMKMESPVSGNTSAMRPVMQPQVGSQQQGFLNAQMVAQRNRELISHHFRQQRMAMMMQQQQQPQ
AFSPPPNVTTTGSMDSALTGPPMAQLPPQQFPYPPNYGISQQPDPAFSRVSSPPNPMISSRIGPSQNPVMQHPQ
SATVYQSSEMKGWPSGSMARNSSFPQQQFSHQGNPAAYSVVHMNGSTSHIGQMSINTLPMSGMPMGPDQVSY
>279Cepphus grylle|A0A7L3S6M9|Aves|SRC3
CMFKMSGLGENSLDSLTNESRKRKPLPCDTPGAGLTCSGEKRRREQESKYIEELAELISANLSDIDNFNVKPDK
CAILKETVRQIRQIKEQGKAVSNDDDVQKADVSSTGQGVIDKDSLGPLLLQASALDGFLFVVNRDGNIVFVSEN
VTQYLQYKQEDLVNTSVYGILHEEDRKDFLKNLPKSSVNGVAWTNETPRQKSHTFNCRMMVKTSHDLLEDVGSH
QDTRQRYETMQCFALSQPRAMMEEGEDLQSCMICVARRITTAERVFPTNPESFITRHDLSGKLVNIDTNSLRSS
MRPGFEDIIRRCIQRFLCHNDGQSWSNKRHYHEAYTQGHAETPLYRFSLADGTIVTAQTKSKLFRNPVTNDRHG
FVSTHFLQREQNGYRPNPNSVGQNIRTPVAGNTNSVGGLMNMSPGQGMPMQNRNYGMGDPSAVGQLSTTRYGGP
GNMGPVNSGPGMQSSAYQNNSYGLNISSPPHGSPGLASNQQNLMISPRNRGSPKMSSHQFSPVTGMHSPMGSAS
NTGNSTFSSSSLSALQAISEGVGTSLLSTLSSPGPKLDNSPNMSIAQQSKTNTQDSKSPSGLYCEQNQVESSIC
QSNSRDLLSEKDSKEGNLEAPESQRGPSESKGHKKLLQLLTCSSDERGHSTASNSPLDSNCKESSTNVTSPSGV
SSSTSGGVSSSSSNMHGSLLQEKHRILHKLLQNGNSPAEVAKITAEATGKDTYHDASNTAPCGEGTVKQEQLSP
KKKENNALLRYLLDKDDVKDPLSKELKPKVESVDNKMGQCSSSTIPTSSQEKEMKIKTEPAEEMSGDLDNLDAI
LGDLTSSEFYNNAMSANGNNLGTKQALFQGNALLGVRSPQSVPTTRPPFTRAMSLDSPVGSSAPVRNVNAFSML
QKQNLMGGSPRMIESQENFGANLAGAPNRNVAMNQHQSGEWGLPNSKVNRLEPTSSASMLRPGPEFSASLPRSS
VGGSMSGLPVRSNSIPGTRPMLQQQMIHMRPNEINIGMGGNPYGPPGPSNQPGSWPDNMLSMEQASRASQNRQL
VRNSLDDLLCPAPNMEGQNDERALLDQLHTLLSNTDVTGLEEIDRALGIPDLVNQGQALEPKQDSFQGQESSVM
IDQKPSLYGQPYQGQGAAMPGSFSNIQGQQPTFNSVMNQMSQQSNFPLQSMHPRANVMRPRTNTPKQLRMQLQQ
RLQGQQFMNQTRQALEMKMENPVSGNTSVMRPVMQPQVGSQQQGFLNAQMVAQRNRELISHHFRQQRMAMMMQQ
QQQPQAFSPPPNVTASGSMDSALTGPPMAQVPPQQFTYPPNYGISQQPDPAFSRVSSPPNPMMSSRIGASQNPM
MQHPQTAAMYQSPEMKGWPSGSMARSSSFPQQQFSHQGNPAAYGMMHMNGSSGHIGQMNINPMPMSGMPMGPDQ
VSR

>280Uria aalge|A0A7L3UGN6|Aves|SRC3
CMFKMSGLGENSLDSLTNESRKRKPLPCDTPGASLTCSGEKRRREQESKYIEELAELISANLSDIDNFNVKPDK
CAILKETVRQIRQIKEQGKAVSNDDDVQKADVSSTGQGVIDKDSLGPLLLQASALDGFLFVVNRDGNIVFVSEN
VTQYLQYKQEDLVNTSVYGILHEEDRKDFLKNLPKSSVNGVAWTNETPRQKSHTFNCRMMVKTSHDLLEDVGSH
QDTRQRYETMQCFALSQPRAMMEEGEDLQSCMICVARRITTAERVFQTNPESFITRHDLSGKLVNIDTNSLRSS
MRPGFEDIIRRCIQRFLCHNDGQSWSNKRHYHEAYTQGHAETPLYRFSLADGTIVTAQTKSKLFRNPVTNDRHG
FVSTHFLQREQNGYRPNPNSVGQNIRTPVAGNTNSVGGLMNMSPGQGMPMQNRNYGMGDPSAVGQLSTTRYGGP
GNMGPVNSGPGMQSSAYQNNSYGLNISSPPHGSPGLASNQQNLMISPRNRGSPKMSSHQFSPVTGMHSPMGSAS
NTGNSTFSSSSLSALQAISEGVGTSLLSTLSSPGPKLDNSPNMSIAQQSKTSTQDSKSPSGLYCEQNQVESSIC
QSNSRDLLSEKDSKEGNLEAPESQRGPSESKGHKKLLQLLTCSSDERGHSTASNSPLDSNCKESSTNVTSPSGV
SSSTSGGVSSSSSNMHGSLLQEKHRILHKLLQNGNSPAEVAKITAEATGKDTYHDASNTAPCGEGTVKQEQLSP
KKKENNALLRYLLDKDDVKDPLSKDLKPKVESVDNKMGQCSSSTIPTSSQEKEMKIKTEPAEEMSGDLDNLDAI

LGDLTSSEFYNNAMSANGNNLGTKQALFQGNALLGVRSPQSVPTTRPPFTRAMSLDSPVGSSAPVRNVNAFSML
QKQNLMGGSPRMIESQENFGANLAGAPNRNVAMNQHQSGEWGLPNSKVNRLEPTSSASMLRPGPEFSASLPRSS
VGGSMSGLSVRSNSIPGTRPVLQQQMIHMRPNEINIGMGGNPYGQPGPSNQPGSWPDNMLSMEQASRGSQNRQL
VRNSLDDLLCPAPNMEGQNDERALLDQLHTLLSNTDVTGLEEIDRALGIPDLVNQGQALEPKQDSFQGQESSVM
IDQKPSLYGQPYQGQGAAMPGSFSNIQGQQPTFNSVMNQMSQQSNFPLQSMHPRANVMRPRTNTPKQLRMQLQQ
RLQGQQFMNQTRQALEMKMENPVSGNTSVMRPVMQPQVGSQQQGFLNAQMVAQRNRELISHHFRQQRMAMMMQQ
QQQPQAFSPPPNVTASGSMDSALTGPPMAQVPPQQFPYPPNYGISQQPDPAFSRVSSPPNAMMSSRIGASQNPM
MQHPQTAAMYQSPEMKGWPSGSMARSSSFPQQQFSHQGNPAAYTMMHMNGSSGHIGQMNINTMPMSGMPMGPDQ
VSC
>281Atlantisia rogersi|A0A7L3WIE6|Aves|SRC3
CMVKMSGLGENSLDSLTNESRKRKSLPCDTPGASLSCSGEKRRREQESKYIEELAELISANLSDIDNFNVKPDK
CAILKETVRQIRQIKEQGKAVANDDDVQKADVSSTGQGVIDKDSLGPLLLQAALDGFLFVVNRDGNIVFVSENV
TQYLQYKQEDLVNTSVYGILHEEDRKDFLKNLPKSSVNGVAWTNETPRQKSHTFNCRMMVKTHDLLEDVGSHQD
IHQRYETMQCFALSQPRAMMEEGEDLQSCMICVARRITTAERVFLTNPESFITRHDLSGKLVNIDTNSLRSSMR
PGFEDIIRRCIQRFLCHNDGQSWSNKRHYHEAYTQGHAETPLYRFSLADGTIVTAQTKSKLFRNPVTNDRHGFV
STHFLQREQNGYRPNPNSVGQNIRTPMAGNTNSVGALMNMSPGQGMPVQNRNYGMGDPNAVGQLSNSRYGGPGN
MGPVNSGATMQSSAYQNNNYGLNISSPPHGSPGLTSNQQNLMISPRNRGSPKMSSHQFSPVTGMHSPMGSASNT
SNSTFSSSSLSALQAISEGVGTSLLSTLSSPGPKLDNSPNMSMTQQSKASTQDSKSPSGLYCEQNQVESSICQT
NSRDLLSEKDSKEGNLEASESQRGPSESKGHKKLLQLLTCSSEERGHSSNSPLDSNCKESSTNVTSPSGVSSST
SGGVSSSSNMHGSLLQEKHRILHKLLQNGNSPAEVAKITAEATGKDTYHDASNTAPCGEGTVKQEQLSPKKKEN
NALLRYLLDKDDVKDPLSKELKPKVENVDNKMGQCSSSTIPTSSQEKEMKIKTEPAEEMSGDLDNLDAILGDLT
SSEFYSNAMSANGNNLGTKQALFQGNALLGMRSPQSVQATRPTFNRAMSLDSPVGSSAPVRNVNPFSMLQKQNL
MGGSPRMIENQENFGANLGFAPNRNVAMSQHQSGDWGLPNSKVNRLEPTSSASMLRPGPEFSASLPRPTVGGGS
MSGLPVRSNSLPGTRAMLQQQMIHMRPNEINIGMGGNLYGQPGPSNQPGSWPDNMLSMEQASRGSQNRQLVRNS
LDDLLCPAPNMEGQTDERALLDQLHTLLSNTDVTGLEEIDRALGIPDLVNQGQALEPKQDSFQGQESSVMIDQK
PSLYGQPYQGQGAAMPGGFSNIQGQQPSFNSVMNQMSQQSNFPLQSMHPRTNVMRPRTNTPKQLRMQLQQRLQG
QQFMNQTRQALEMKMENPVSSNTSVMRPVMQPQVGSQQQGFLNAQMVAQRNRELISHHFRQQRMAMMMQQQQQP
QAFSPPPNVTASGSMDSALTGPPMAQVPPQQFPYPPNYGISQQPDPTFSRVPSPPNPMMSSRIGPSQNPMMQHP
QTAAMYQSPEIKGWPSGSMARSSSFPQQQFSHQGNPAAYSMMHMNGSSGHIGQMNINTMPMSGMPMGPDQVSC
>282Eurystomus gularis|A0A7L4CVM0|Aves|SRC3
CMVKMSGLGENSLDSLTNESRKRKPLPCDTPGASLTCSGEKRRREQESKYIEELAELISANLSDIDNFNVKPDK
CAILKETVRQIRQIKEQGKAVSNDDDVQKADVSSTGQGVIDKDSLGPLLLQASTNQALALDGFLFVVNRDGNIV
FVSENVTQYLQYKQEDLVNTSVYGILHEDDRKDFLKNLPKSSVNGVAWTNETPRQKSHTFNCRMMVKTSHDLLE
DVGSHQDIRQRYETMQCFALSQPRAMMEEGEDLQSCMICVARRITTAERVFPANPESFITRHDLSGKLVNIDTN
SLRSSMRPGFEDTIRRCIQRFLCHNDGQSWSNKRHYHEAYTQGHAETPLYRFSLADGTIVTAQTKSKLFRNPVT
NDRHGFVSTHFLQREQNGYRPNPNPVGQNIRTPVAGNTNSVGGLMNMSPGQGMPMQNRNYGVGDPGAVGQLSST
RYGGPGNMGQVNSGPGMQSSTYQNNSYGLNISSPPHGSPGLTSNQQNLMISPRNRGSPKMSSHQFSPVTGMHSP
MASTSNTGNSTFSSSSLSALQAISEGVGTSLLSTLSSPGPKLDNSPNMTIAQQSKTSTQDSKSPSGLYCEQNQV
ESSVCQSNSRDVLSEKDSKEGNLETSESQRGPSESKGHKKLLQLLTCSSDERGHSTASNSPLDSNCKESSTNVT
SPSGVSSSTSAGVSSSSNMHGSLLQEKHRILHKLLQNGNSPAEVAKITAEATGKDTYHDASNTAPCGEGTVKQE
QLSPKKKENNALLRYLLDKDDVKDPLSKELKPKVESVDNKMGQCSSSTIPTSSQEKEMKIKAEPTEEMSGDLDN
LDAILGDLTSSEFYNNAMSANGNNLGTKQALFQGNALLGMRSPQSVQTARPPFSRAMSLDSPVGSGAPGRNVNA
FSMLQKQNLMGGSPRMIENQENFGANLASAPNRNVAMNQHQSGDWGLPNSKVNRLDSTGSASMLRAGPEFSASL
PRPAVGGSVPGLPVRSNSIPGTRPMLQQQMIHMRPNEINIGMGGNPYGQPGPSNQPGSWPDNMLSMEQASRASQ
NRQLVRNSLDDLVCPAPNMEGQNDERALLDQLHTLLSNTDVTGLEEIDRALGIPDLVNQGQALEPKQDSFQGQE
SSVMIDQKPSLYGQPYQGQGAAMPGGFSNIQGQQPSFNSVMNQMSQQSNFPLQSMHPRANVMRPRTSTPKQLRM
QLQQRLQGQQFMNQTRQALEMKMENPVSGNTSVMRPVMQPQVGSQQGFLNAQMVAQRNRELISHHFRQQRMAMM
MQQQQQPQAFSPPPNVTASGNMDSALTGPQMAQVPPQQFPYPPNYGISQQPDPAFTRVSSPPNPMMSSRIGPSQ
NPMMQHPQTATMYQSPEMKGWPSGSMARSSSFPQQQFSHQGNPAAYSMMHMNGSSGHIGQMNINTMPMSGMPMG
PDQVS
>283Eurystomus gularis|A0A7L4D0X8|Aves|SRC3
MSGLGDSSTDPANPDSRKRKGSPCDTAQSNEKRRREQENKYLEELAELLSANIGDIDTLSVKPDKCKILKKTVD
QIQQMKRLEQEKAADDEVQKSDISSSSQGVIEKESLGPLLLEALDGFFFVVNREGRIVFVSENVTSYLGYNQEE
LMNTSVYSILHVGDHAEFVKNLLPKSLVNGVPWPQEATRRNSHTFSCRMLIRPPDEPGTENQEARQRYEVMQCF

TVSQPKSFKEEGEDFQSCLICIARRLPRPAAVTSSESFVTKQDTTGKIISIDTSSLRAAGRTGWEDLVRKCIYA
FFQPQGRELSYARQLFQEVMTRGTAFSPSYRFTLSDGTVLSAHTKCKLCYPSSPEMQPFIMGIHVIDRDHGMLS
PQENTNSTMSLPRVSPSLNPSISPGQGMALPPSIPPSNGSMLATNVTQQPGAGLHNNNSSTSQTSFGCSPGNQI
GANVALSQGQAGSQNSNHTLNLNSSPMNSPGITPPQFMSPRHRVSPGLVPRPRVPSNPFSPTMPTMHSPVGMAN
SGSGSGCGTGGTRPFPSGPINSMQSLTEGPSTPVGYSTPPPVLRQLSSQSSPGRLAMQPMKAESKDSKEIASIL
SEMIQSDGGSGDSKPLDSGMLHASDRLSEGDSKCSQATSHKLVQLLATTAEQQLRHADADTSCKDSLACAGTSG
TLATSNPPSSTCPSSHSSLTERHKILHRLLQEGSPSDITTLGMEHEKKENSSNSSTTPTAQSQLPSTPDIKLEP
DALKKKDVKDHQLLRYLLDKDEKELAAAPALSLDDVKVKVEKTEQMEPCNTAPVPLAKPPAAEEVKLETQGQFA
AELEQLDQLLPSLEKASQLPGLCGPERNESGVAVKPEMLTAPLQPSTAARAPAGLNPLNSGQGVQAGRTPFEFC
DPLEPAQLRPVPCPASNGSSPHGRGPPQQGRGTVTAPNPFPPDTGMPELEMGVPDHQFGQPGMGEQIPWTDNSM
AAMNRGALNKPEDQCITSQLDELLCPPTTPEGRNDEKALLEQLVSFLSGKDETELAELDRALGIDKLVQGGGLE
ALTERFQPQQATPPLMMEQKPGMYPQPYSSASPTTNLPGAFPGMVRQKPSFGPMPVQVPPPRGAFPTNMAMQPR
QTLNRPPTAPNQLRLQLQQRLQGQQQLIHQNRQAILNQFAATSPVGINMRAGMQQQITPQPPLNAQMLAQRQRE
LYSQQHRQRQLMQQRAILMRQQSFGNNLPPSTGLPVQIGAARLPQAPPQQFPYPPNYGTTPGNPPTSTSPFSPL
ASNPEAALANRSSMVNRGMMGGVGGQFGAGMTPQMQQNIFQYSGSGMGQQSEPAFAPSLSPSSPLMSPQLPPSQ
SPMLQPAPPTPGYQSPDMKTWQQGAMGNSNVFSQAGQSQPAPAQQGMYNNMSITVSMAGGNANVQNMNPMAGQM
QMNSLQMPGMNSMCSEQVNDPALRPAGLYCNQLSSTDLLKPEADGAQQVQQVQVFADVQCTVNLVGDPYLNPPA
PLGTPKNAAGPPPPPGQQKSLLQQLLTE
>284Podargus strigoides|A0A7L4GFF7|Aves|SRC3
CMVKMSGLGENSLDSLTNESRKRKPLPCDTPGASLTCSGEKRRREQESKYIEELAELISANLSDIDNFNVKPDK
CAILKETVRQIRQIKEQGKAVSNDDDVQKADVSSTGQGVIDKDSLGPLLLQAALDGFLFVVNRDGNIVFVSENV
TQYLQYKQEDLVNTSVYGILHEEDRKDFLKNLPKSSVNGVAWTNETPRQKSHTFNCRMMVKTSHDLLEDVGSHQ
DTRQRYETMQCFALSQPRAMMEEGEDLQSCMICVARRITTAERVFPTNAESFITRHDLSGKLVNIDTNSLRSSM
RPGFEDIIRRCIQRFLCHNDGQSWSNKRHYHEAYTQGHAETPLYRFSLADGTIVTAQTKSKLFRNPVTNDRHGF
VSTHFLQREQNGYRPNPNSVGQNIRTPVAGNTNSVGGLMNMSPGQGMPLQNRNYGMGDPSAVAQLSSTRYGGPG
NMGPVNSGPGMQSSAYQNNSYGLNISSPPHGSPGLTSNQQNLMISPRNRGSPKMSSHQFSPVTGMHSPMGSASN
TGNSTFSSSSLSALQAISEGVGTSLLSTLSSPGPKLDNSPNMSIAQQSKTSTQDSKSPSGLYVEQNQVESSICQ
SNSRDLLSEKDSKEGNLEASESQRGPSESKGHKKLLQLLTCSSDERGHSTASNSPLDSNCKESSSNVTSPSGVS
SSTSAGVSSSSNMHGSLLQEKHRILHKLLQNGNSPAEVAKITAEATGKDTYHEASNAAPCGEGTVKQEQLSPKK
KENNALLRYLLDKDDVKDPLSKELKPKVESVDNKMGQCSSSTIPTSSQEKEMKIKTEPTEEMSGDLDNLDAILG
DLTSSEFYNNAMSANGNNLGTKQALFQGNAVLGLRSPQSVQTTRPPFSRAMSLDSPVGPGAPVRNVTAFSMLQK
QNLMGGSPRMIENQENFGANLGFAPNRNVAMNQHQSGDWGLPNSKVNRLEPTSSASMLRPAPEFSVSLPRPTVG
GSMSGLPVRSNSIPGTRPVLQQQMIHMRPNEMNIGMGGNPYAQPGPSNQPGSWPDNMLSMEQASRGSQNRQLVR
NSLDDLICTAPNLEGQNDERALLDQLHTLLSNTDVTGLEEIDRALGIPDLVNQGQALEAKQDSFQGQESSVMID
QKPSLYGQPYQGQGAAMPGGFSNIQGQQPSFNSVMNQMSQQSNFPLQSMHPRANVMRPRTNTPKQLRMQLQQRL
QGQQFMNQTRQALEMKMENPVSGNTSVMRPVLQPQVGSQQQGFLNAQMVAQRNRELISHHFRQQRMAMMMQQQQ
QPQAFSPPPNVTASGSMDTALTGPPMAQVPPQQFPYPPNYGLSQQPDPAFSRVSSPPNPMMSSRIGASQNAMMQ
HPQTAPMYQSPEMKGWPSGSMARSSSFPQQQFSHQGNAAAYSMMHMNGSSGHIGQMNINTMPMSGMPMGPDQVS
C
>285Pomatorhinus ruficollis|A0A7L4J6G9|Aves|SRC3
CMVKMSGLGENSLDSLTNESRKRKPLPCDTPGASVVCSGEKRRREQESKYFEELAELISANLSDIDNFNVKPDK
CAILKETVRQIRQIKEQGKAVSNDDDVQKADVSSTGQGVIDKDSLGPLLLQAALDGFLFVVNRDGNIVFVSENV
TQYLQYKQEDLVNTSVYGILHEEDRKDFLKNLPKSSVNGVAWTNETPRQKSHTFNCRMMVKSSHDLLEDVGGYQ
DIHQRYETMQCFALSQPRAMMEEGEDLQSCMICVARRITTAERVFPANPESFITRHDLSGKLVNIDTNSLRSSM
RPGFEDIIRRCIQRFLCHNDGQSWSNKRHYHEAYTQGHAETPLYRFSLADGTIVTAQTKSKLFRNPITNDRHGF
VSTHFLHREQNGYRPNPNSVGQNIRTPVTGNTNSVNGLMNMSPGQAMSMQNRNYGMGDPNAVGQLTSTRYGAPG
NMGLVNSGPGMQSSAYQSNSFGLNISSPPHGSPGLTSNQQNLMVSPRNRGSPKMSSHQYSPVTGMHSPMGSASN
TSNSTFSSSSLSALQAISEGVGNSLLSTLSSPGPKLDNSPNISITQQSKTSTQDSKSPSGLYCEQNQVESSICQ
SNSRDLFSEKDSKEGNLEASESQRGPSESKGHKKLLQLLTCSSDERGHSTASNSPLDSNCKESSTSATSPSGVS
SSTSGGVSSSSNVHGSLLQEKHRILHKLLQNGNSPAEVAKITAEATGKDTYHDTSNTASCGEGTVKHEQLSPKK
KENNALLRYLLDKDDIKDPLSRELKPKVEGVDNKMGQCTSSTIPTSSQEKEMKIKMEPTEEMNGDLDNLDAILG
DLTGSEFYNNAMSANGNNPGTKQALFQGNALLGVKSPQSVQTTHPPFNRALSLDSPIGSGASMRNVNAFSMLQK
QNLMGGSPRMIENQENFGANLGFTPNRNVAMNQHQSGDWGLPNSKVNRLESASSASMLRPGPEFSTALARPPEG
GLPVRSNSIPGTRPVPQQQVIHMRPNEINIDMGGNLYGQPGPCNQPGLWPDNMMSVEQASRGSQNRQLVRSSLD

DLLCSAPNVEGQNDERALLDQLHTLLNNTDVTGLEEIDRALGIPDLVSQGQALEPKQDSFQGQESSVMIDQKPL
LYGQPYQGQGAAMPGGFSNIQGQQPSFNAVMNQMSQLSNFPLQSMHPRANVMRPRTNTPKQLRMQLQQRLQGQQ
FMNQSRQTLEIKIENPVSGTNSVMRPVMQPQVGSQQQGFLNAQMVAQRNRELISHHFRQQRMAMMMQQQQQPQA
FSPPPNVTASGSMDNALTGPPMAQVPSQQFPYPPNYGISQQPDPAFSRVSSPPNPMISSRIGPSQNPMLQHPQS
ATMYQSSEMKGWPSGSMPRNSSFPQQQFSHQGNPAAYSMMHMNGSSGHMGQMSINTMPMSGLPMGPDQVSC
>286Ceuthmochares aereus|A0A7L4JIA9|Aves|SRC3
CMVKMSGLGENSLDSLTNESRKRKPLPCDTPGASLTCSGEKRRREQESKYIEELAELISANLSDIDNFNVKPDK
CAILKETVRQIRQIKEQGKAVSNDDEVQKADVSSTGQGVIDKDSLGPLLLQAALDGFLFVVNRDGNIVFVSENV
TQYLQYKQEDLVNTSVYGILHEEDRKDFLKNLPKSSVNGVTWTNETPRQKSHTFNCRMTVKTSHDLLEDVGSHQ
DLRQRYETMQCFALSQPRAMMEEGEDLQSCMICVARRITTAERVFPANPESFITRHDLSGKLVNIDTNSLRSSM
RPGFEDIIRRCIQRFLCHNDGQSWSNKRHYHEAYTQGHAETPLYRFSLADGTIVTAQTKSKLFRNPVTNDRHGF
VSTHFLQREQNGYRPTPNAVGQNIRTAVAGNTNSVGGLMNVSPGQGMPVQNRNYGMGDANAVGQLSNTRYGGPG
SMGPVNSGPGMQSSAYQNNTYGLNINSPPHGSPGLTSNQQNLMISPRNRGSPKMSSHQFSPVTGMHSPMGSASN
TGNSTFSSSSLSALQAISEGVGTSLLSTLSSPGPKLDNSPNMNIAQQSKTSIQDSKSPSGLYCEQNQVESSICQ
SNSRDLLSEKDSKEGNLEAPESQRGPSESKGHKKLLQLLTCSSDERGHSTASNSPLDSNCKESSTNVTSPSGVS
SSTSGGVSSSSNMHGSLLQEKHRILHKLLQNGNSPAEVAKITAEATGKDTYHDASNTAPCGEGTVKQEQLSPKK
KENNALLRYLLDKDDVKDPLSKELKPKVESVDNKVGQCSSSTIPTSSQEKEMKIKTEPTEEMSGDLDNLDAILG
DLTSSEFYNNAMSANGNNLGTKQALFQGNTLLGMRNPQSVQTTRPPFTRAMSLDSPMGSGAQVRNVNAFSMLQK
QSLMGESPRMIENQENFGANLGFASNRNVAMNQHQAGDWGLPSSKVNRLEPTSSASMLRPGPEFGASLPRPTVG
GSVSGLPVRSNSVPGTRPMLQQQMIHMRPNEINIGMGGNPYGQPGPSNQPGSWPDNMLSMEQASRGSQNRQLVR
NSLDDLLCPAPNMEGQNDERALLDQLHTLLSNTDVTGLEEIDRALGIPDLVNQGQGLEPKQDSFQGQESSVMID
QKSSLYGQPYQGQGAAMPGGFSNMQGQQPSFNSVMNQMSQQSSFPLQSMHPRANVMRPRTNTPKQLRMQLQQRL
QGQQFMNQTRQALEMKMENPVSANTSVMRPVMQPQVGSQQQGFLNAQMVAQRNRELISHHFRQQRMAMMMQQQQ
QPQAFSPPPNVTASGSMDSALTGPPMAQVPPQQFPYPPNYGISQQPDPAFSRVPSPPNPMMSSRIGPSQNPMMQ
HPQTATMYQSPEMKGWPSGSMARSSSFPQQQFSHQGNPAAYSMMHMNGSSGHIGPMNINTMSMSGMPMGPDQVS
C
>287Glareola pratincola|A0A7L4M9U6|Aves|SRC3
CMFKMSGLGENSLDSLTNESRKRKPLPCDTPGASLTCSGEKRRREQESKYIEELAELISANLSDIDNFNVKPDK
CAILKETVRQIRQIKEQGKAVSNDDDVQKADVSSTGQGVIDKDSLGPLLLQASALDGFLFVVNRDGNIVFVSEN
VAQYLQYKQEDLVNTSVYGILHEEDRKDFLKNLPKSSVNGVAWTNETPRQKSHTFNCRMMVKTSHDLLEDVGSH
QDTRQRYETMQCFALSQPRAMMEEGEDLQSCMICVARRITTAERVFPTNPESFITRHDLSGKLVNIDTNSLRSS
MRPGFEDIIRRCIQRFLCHNDGQSWSNKRHYHEAYTQGHAETPLYRFSLADGTIVTAQTKSKLFRNPVTNDRHG
FVSTHFLQREQNGYRPNPNSVGQNIRTPVAGNTNSVGGLMNMSPGQGMLMQNRNYGMGDPSAVGQLSSTRYGGP
ANMGPVNSGPGMQSSAYQNSSYGLNISSPPHGSPGLASNQQNLMISPRNRGSPKMSSHQFSPVTGMHSPMGSAS
NTSNSTFSSSSLSALQAISEGVGTSLLSTLSSPGPKLDNSPNMSIAQQSKTSTQDSKSPSGLYCEQNQVESSIC
QSNSRDLLSEKDSKEGNLEASESQRGPSESKGHKKLLQLLTCSSDERGNSTASNSPLDSNCKESSTNVTSPSGV
SSSTSGGVSSSSSNMHGSLLQEKHRILHKLLQNGNSPAEVAKITAEATGKDTYHDASNTAPCGEGTVKQEQLSP
KKKENNALLRYLLDKDDVKDPLCKELKPKVESVDNKMGQCSSSTVPTSSQEKEMKIKTEPVEEMSGDLDNLDAI
LGDLTSSEFYNNAMSANGNNLGTKQALFQGNALLGVRSPQSVPTTRPPFTRAMSLDSPVGSSAPVRNVNAFSML
QKQNLMGGSPRMIESQENFGANLGFTPNRNVAMNQHQSGEWGLPNSKVNRLEPTSSASMLRPGPEFSASLPRPS
AGGSMSGLPVRSNSIPGTRPMLQQQMIHMRPNELNIGMSGNPYGQPGPSNQPGSWPDNMLSMEQASRGSQNRQL
VRNSLDDLLCPAPNMEGQNDERALLDQLHTLLSNTDVTGLEEIDRALGIPDLVNQGQALEPKQDSFQGQESSVM
IDQKSSLYGQPYQGQGAAMPGSFSSIQGQQPTFNSVMNQMSQQSNFPLQSMHPRANVMRPRTNTPKQLRMQLQQ
RLQGQQFMNQTRQALEMKMENPVSGNTSVMRPVMQPQVGSQQQGFLNAQMVAQRNRELISHHFRQQRMAMMMQQ
QQQPQAFSPPPNVTASGSMDTALTGPPMAQVPPQQFPYPPNYGISQQPDPAFSRVSSPPNSMMSSRIGASQNPM
MQHPQTAAMYQSPEMKGWPSGSMARSSSFPQQQFSHQGNPVAYSMMHMNGSSGHIGQMNINTMPMSGMPMGPDQ
VSC
>288Piaya cayana|A0A850X1H1|Aves|SRC3
CMVKMSGLGENSLDGLTNESRKRKPLPCDIPGASLTCSGEKRRREQESKYIEELAELISANLSDIDNFNVKPDK
CAILKETVRQIRQIKEQGKAVSNDDDVQKADVSSTGQGVIDKDSLGPLLLQAALDGFLFVVNRDGNIVFVSENV
TQYLQYKQEDLVNTSVYGILHEEDRKDFLKNLPKSSVNGVTWTNETPRQKSHTFNCRMMVKTSHDLLEDVGSHQ
DLRLRYETMQCFALSQPRAMMEEGEDLQSCMICVARRITTAERVFQANPESFITRHDLSGKLVNIDTNSLRSSM
RPGFEDIIRRCIQRFLCHNDGQSWSNKRHYHEAYTQGHAETPLYRFSLADGTIVTAQTKSKLFRNPVTNDRHGF
VSTHFLQREQNGYRSTPNAVGQNIRTAVTGNTNSLGGLMNVSPGQGMPVQNRTYGMGDANAVGQLSNTRYGGAG

SMGPGNSGPGMQSSAYQNNAYGLNINSPPHGSPGLTSNQQNLMISPRNRGSPKMSSHQFSPVAGMHSPMGSASN
TSNSTFSSSSLSALQAISEGVGTSLLSTLSSPGPKLDNSPNMNIAQQSKTSTQDSKSPSGLYCEQNQVESSICQ
SNSRDLLSEKDSKEGNLEVPESQRGPSESKGHKKLLQLLTCSSDERGHSTASNSPLDSNCKESSTNVTSPSGVS
SSTSGGVSSSSNMHGSLLQEKHRILHKLLQNGNSPAEVAKITAEATGKDTYHDASNTAPCGEGTVKQEQLSPKK
KENNALLRYLLDKDDVKDPLSKELKPKVESLDNKVGQCSSSTIPTSSQEKEMKIKTEPTEEMSGDLDNLDAILG
DLTSSEFYNNAMSANGNNLGTKQVLFQGNTLLGMRSPQSVQATRPPFTRATSLDSPMGSGAQMRNVNAFSMLQK
QNLMVESPRMLENQENFGANLGFAPNRNVAMNQHQAGDWGLPNSKVNRLEPTSSASMLRPGPEFGASLPRPTVG
GPVSGLPVRSNSVPGTRPMLQQQMIHMRPNEINIGMGGNPYGQPGPSNQPGSWPDNMLSMEQASRSSQNRQLVR
NSLDDLLCPAPNMEGQNDERALLDQLHTLLSNTDVTGLEEIDRALGIPDLVNQGQGLEPKQDSFQGQESSVMID
QKSSLYGQPYQGQGAAMPGGFSNMQGQQPSFNSVMNQMSQQSSFPLQSMHPRANVMRPRTNTPKQLRMQLQQRL
QGQQFMNQTRQALEMKMENPVSANTSVMRPVMQPQVGSQQQGFLNAQMVAQRNRELISHHFRQQRMAMMMQQQQ
QPQTFSPPPNVTASGSMDSALTGPPMAQVPPQQFPYPPNYGISQQPDPAFSRVPSPPNPMMSSRIGPSQNPMMQ
HPQTATMYQSPEMKGWPSGSLARSSSFPQQQFSHQGNPAAYSMMHMNGSSGHIGQMNINTISMSGMPMGPDQVS
C
>289Anhinga anhinga|A0A851PPB8|Aves|SRC3
CMVKMSGLGENSLDSLTNESRKRKPLPCDTPGASLTCSGEKRRREQESKYIEELAELISANLSDIDNFNVKPDK
CAILKETVRQIRQIKEQGKAVSNDDDVQKADVSSTGQGVIDKDSLGPLLLQAALDGFLFVVNRDGNIVFVSENV
TQYLQYKQEDLVNTSVYGILHEEDRKDFLKNLPKSSVNGVAWTNETPRQKSHTFNCRMMVKTSHDLLEDVSSHQ
DTRQRYETMQCFALSQPRAMMEEGEDLQSCMICVARRITTAERVFPTNPESFITRHDLSGKLVNIDTNSLRSSM
RPGFEDIIRRCIQRFLCHNDGQSWSNKRHYHEAYTQGHAETPLYRFSLADGTIVTAQTKSKLFRNPVTNDRHGF
VSTHFLQREQNGYRPNPNSVGQNIRTPVAGNTNSVGGLMNMSPGQGMPMQNRSYGMGDPNAVGQLSNTRYGGPG
NMGPVNSAPGMQSTAYQNSSYGLNISSPPHGSPGLTSNQQNLMISPRNRGSPKMSSHQFSPVTGMHSPMGSASN
TGNSTFSSSSLSALQAISEGVGTSLLSTLSSPGPKLDNSPNMSIAQQSKTSTQDSKSPSGLYCEQNQVESSICQ
SNSRDLLSEKDSKEGNLEACESQRGPSESKGHKKLLQLLTCSSDERGHSTASNSPLDSNCKEPSTNVTSPSGVS
SSTSGGVSSSSNMHGSLLQEKHRILHKLLQNGNSPAEVAKITAEATGKDTYHDASNTAPCGEGTVKQEQLSPKK
KENNALLRYLLDKDDVKDPLSKELKPKVESVDTKMGQCSSSMIPTSSQEKEMKIKTEPAEEMSGDLDNLDAILG
DLTSSEFYNNAMSANGNNLGTKQALFQGNALLAGMRSPQPVQTTRPPFNRAMSLDSPVGSSAPVRNVNAFSMLQ
KQNLMGGSPRRIENQENFGASLGFAPNRNVVMNQHQSGDWGLPNSKVNRLEPTSSASMLRPGPEFSASLPRPTV
GGSMSGLPVRSNSIPGTRPMLQQQMIHMRPNEINIAMGGNPYGQPGPASQPGSWPDNMLSVEQASRGSQNRQLV
RNSLDDLLCPAPNMEGQNDERALLDQLHTLLSNTDVTGLEEIDRALGIPDLVNQGQALEPKQESFQGQESSVMI
DQKPSLYGQPYQGQGAAMPGGFSNIQGQQPSFNSVMNQMSQQSNFPLQSMHPRANVMRPRTNTPKQLRMQLQQR
LQGQQFMNQTRQALEMKMENPVSGSTSVMRPVMQPQVGSQQQGFLNAQMVAQRNRELISHHFRQQRMAMMMQQQ
QQPQAFSPPPNVTASGSMDSALTGPPMAQVPPQQFPYPPNYGISQQPDPAFSRVSSPPNPMMSSRLGPSQNSMM
QHPQTATMYQSPEMKGWPSGSMARSSSFPQQQFSHQGNPAAYSMMHMNGSSGHIGQMNINTMPMSGMPMGPDQV
SC
>290Ramphastos sulfuratus|A0A852C4B5|Aves|SRC3
CMVKMSGLGENSLDSLTNESRKRKALPCDTPGASLTCSGEKRRREQESKYIEELAELISANLSDIDNFNVKPDK
CAILKETVRQIRQIKEQGKAVSNDDDVQKADVSSTGQGVIDKDSLGPLLLQAALDGFLFVVNRDGNIVFVSENV
TQYLQYKQEDLVNTSVYGILHEEDRKDFLKNLPKSSVNGVSWSNETPRQKSHTFNCRMMVKTSHELLEDVGSHQ
DIRQRYETMQCFALSQPRAMMEEGEDLQSCMICVARRITTAERVFPTNPESFITRHDLSGKLVNIDTNSLRSSM
RPGFEDIIRRCIQRFLCHNDGQSWSNKRHYHEAYTQGHAETPLYRFSLADGTIVTAQTKSKLFRNPVTNDRHGF
VSTHFLQREQNGYRPNSNSVGQNIRTPVAGNTNSVGGLMNMSPGQGMPMQNRNYGMGDPSAVGQLSSTRYGGPG
NMGPVNSGPGMQSSAYQNNSYGLNISSPPHGSPGLPSNQQNLMISPRNRGSPKMNSHQFSPVTGMHSPMGSASN
TSNNTFSSSSLSALQAISEGVGTSLLSTLSSPGPKLDNSPNMSMAQQSKTSTQDSKSPSGLYCEQNQVESSICQ
SNSRDLLSEKDSKEGNLEASESQRGPSESKGHKKLLQLLTCSSDERGHSTASNSPLDSNCKESSTSVTSPSGVS
SSTSGGVSSSSNMHGSLLQEKHRILHKLLQNGNSPAEVAKITAEATGKDTYHDAGNPAPCGEGTVKQEQLSPKK
KENNALLRYLLDKDDVKDPLSKELKPKVENVDTKMGQCTSSTVPTSSHEKAMKIKAEPAEEMSGDLDNLDAILG
DLTSSEFYSNAMSANGNNLGGKQALFQGNALLAGMRSPQSMQTTRPPFNRAMSLDSPMGSSAPARNVNTFSMLQ
KQNLMGGSPRMMENQENFGANLGFAPNRNVAMNQHQSADWGLSNSKVNRMEQTNSSSLLRPGPEFSTTLPRPAV
GGSMPGLPVRSNSLPGTRPMLQQQMIHMRPNEINIGMGGNPYGQPGPSNQPGAWPDNMLSMEQASRGSQTRQLV
RNSLDDLLCPAPNMEGQNDERALLDQLHTLLSNTDVTSLEEIDRALGIPDLVNQGQALEAKQDSFQGQESSVMI
DQKASLYGQPYQGQGAAMPGGFSNIQGQQPSFNSVMNQMSQQSNFPLQSMHPRANVMRARTSTPKQLRMQLQQR
LQGQQFMNQTRQTLEMKMENPVSGNTSVMRPVMQPQVGSQQQGFLNAQMVAQRNRELISHHFRQQRMAMMMQQQ
QQPQAFSPPPNVTASGSMDSALTGPPMAQVPPQQFPYPPNYGISQQPDPAFSRVSSPPNPMMSSRIGPSQNPMM

QHPQTATMYQSPEMKGWPSGSMARSSSFPQQQFSHQGNPAAYSMMHMNGNSGHIGQMNINTMPMSGMPMAPDQV
SC
>291Centropus bengalensis|A0A852LQJ7|Aves|SRC3
CVDKMSGLGENSLDTLTNESRKRKPLPCDTPGASLTCSGEKRRREQESKYIEELAELISANLSDIDNFNVKPDK
CAILKETVRQIRQIKEQGKAVSNDDDVQKADVSSTGQGVIDKDSLGPLLLQAALDGFLFVVNRDGNIVFVSENV
TQYLQYKQEDLVNTSVYGILHEEDRKDFLKNLPKSSVNGVTWTNEAPRQKSHTFNCRMMVKTSHDLLEDVGSHQ
DVRQRYETMQCFALSQPRAMMEEGEDLQSCMICVARRITTAERVFPANPESFITRHDLSGKLVNIDTNSLRSSM
RPGFEDIIRRCIQRFLCHSDGQSWSNKRHYHEAYTQGHAETPLYRFSLADGTIVTAQTKSKLFRNPVTNDRHGF
VSTHFLQREQNGYRPTPTAVGQTIRTPVAGNTNSAGGLMNMSPGQGMAVQNRTYGMGDANAVGQLSSARYGGPG
SLGPVNSGPGMQSSAYQNNSYGLNINSPPHGSPGLTSNQQNLMISPRNRGSPKMTSHQFSPVTGMHSPMGSASN
TGNSTFSSSSLSALQAISEGVGTSLLSTLSSPGPKLDNSPNMSIAQQSKTNTQDSKSPSGLYCEQNQVESSICQ
SNGRDVLSEKDSKEGNLEAPESQRGPSESKGHKKLLQLLTCSSDERGHSTASNSPLDSNCKESSTNVTSPSGVS
SSTSGGVSSSSNMHGSLLQEKHRILHKLLQNGNSPAEVAKITAEATGKDTYHDASNTAPCGDGTVKQEQLSPKK
KENNALLRYLLDKDDVKDPLSKELKPKVESVDNKMGQCSSSTIPTSSQEKEMKIKTEPPEEMSGDLDNLDAILG
DLTSSEFYNNAMSANGNNLGTKQALFQGNALLGMRSPQAMQTARPPFTRAMSLDSPMGSGGPLRNINTFSMLQK
QNLMGESPRMIENQENFGANLGFAPNRNVVMNQHQAGDWGLPNSKVTRLEPTSSASMLRPGAEFGASLPRPPVA
GLPVRSNSVPGTRPMLQQQMIHMRPNEINISMGGNPYGQPGPSNQPGSWPDNMLSMEQASRGSQNRQLVRNSLD
DLLCPAPSMEGQNDERALLDQLHTLLSNTDVTGLEEIDRALGIPDLVNQGQGLEPKQDSFQGQESSVMIDQKSA
LYGQPYHGQGAAMPGGFSNMQGQQPSFNSVMNQMSQQSSFPLQSMHPRANVMRPRTNTPKQLRMQLQQRLQGQQ
FVNQSRQALEMKMENPVGGNASVMRPVMQPQVGSQQQGFLNAQMVAQRNRELISHHFRQQRMAMMMQQQQQPQA
FSPPPNVTASGSMDGALTGPPMTQVPPQQFPYPPNYGISQQPDPAFSRVPSPPNPMLSSRIGPSQNPMMQHPQT
AAMYQSAEMKGWPSGSMARSSSFPQQQFSHQGNPAAYSMMHMNGSSSHIGQMNINTMSMSGMPMAPDQVSC
>292Takifugu rubripes|H2SDV4|Actinopteri|SRC3
MSGLGENSMEPLSSENRKRKLSTCDTPGLGCERKRREQESKYIEELAELISANLSDIDSFNVKPDKCAILRETV
RQIRQIKEQGKASSSDDDVQKSDVSSTGQGVIDKDHLGPLLLQALDGFLFVVNREGSIVFVSDNVTQYLQYKQE
ELINTSVYNILHEDDREELHKNLPKTNLNGVSWGGETARQKSHTFTCRMLVKFGHGSMEEGPGGPRYETMQCFA
LTQPKAMIEEGEDLQSCMICVARRVTAMERTESFSTRHELSGKLIQIDQNSVRASMRPGWEDLLRRCIQMFLQH
SDGQPWSHKRHYHEAFLQGHAETPLYRFSMSDGTPVTAQTKSKLYRHPMSNEPQGFISTHLLQREPNGYRSAQS
GNMMPNTMRQQGMGGPQMNPQMSMNPGGGMGGMSRGFGMSEQGHMGHMGPMGGGSMYGGGGSGGGGNRMMQMNQ
MGPMNQVGHMNQMNQMGPMNHPGQAMQQHPQQPPFQSSGGFGLSGMNSPSGSPRMSGPQQGLLMSPRNRGSPKM
GANQFSPGGLHSPMSGIGSGLGSGGSGTTFSSSSLNALQAISEGVGSSLPSTLTSPSPVLKPDSSPSIHSSSSS
SQLTQTAKGGPDGSKSPAGGLGPGPGDHHHPVHHHHHLQHPQAESSGDRPDSQATAAPKESGEGSSEAGLASSE
PQRRVPDSKGHKKLLQLLTSPTEELGIGGSGPTAPPLQPPPTSSNTPASSDSKDPTGGMTSPSSSGVSSSSAIV
NQGQASAVASLSSAHYTGSLQEKHKILHKLLQNGNTPDEVAKITAEATGKVTLGSQEGGEGGAAGTGGGAGPGM
TETKQEQHSPKKEKTHALLHYLLNKDDSKEPADVKPKMEELDGKVAPGAVGGPVRAGPGSGPSSVPDHPESKIK
SEPPDDLHNLESILGVLSGSGSDLFPDQTGGGGANDAGNKPQGCLDDNLRGSPGIGPGPQGPFQRSMSMDGKPP
GGPGGAGRRPMLIKQENMMGSPDGYPGNMGPMGRGMVPQRSPMGGSGDWGMPRSSASPVGSMMRPGLEYTNNKA
MMSGPMVGRSNSVPGGSADMGMGMSPFNQQGQPSQSPSWSDPMMGMEGNRRQFGNTLDDLLVPPTTSEGQSDER
ALLDQLDSLLNNTDGIALEEIDRALGIPDLVSQTQGAEPPIESFPGQDPAMVLDQKPIYGQGYPGPPAMPMQPG
YGGNPMQGQAPQGGFGPMLSQMGQGGSYPGMGAMGGMGHPRANMMRPRMMAANKPMRLQLQQRLQGQQFMNQTR
QGMAMKMENMGNPGMRPAMQPGMGGQPGFLNAQMMAQRSREMVTMQMRRQRMMMLMQQQQQAGAAAGGFSPPPN
VTAPGGMDNPMGGPSMGQPGPQQFGYGGNYGMGQQGDPSFGPSGGSPPNAMMPGRMGPQNPMMQQHPQGGPMYQ
GTDMKGWGQGGMARNSSYAQQQFGQQGNPGQYGGMMMNGGMPPNGGGGHMGQMGGQMGMNPMAMGRMPMGPDQK
YC
>293Latimeria chalumnae|H3ADQ2|Sarcopterygii|SRC3
MSGLGENSLDSVTIETRKRKPSCDTQGGSLNCSGEKRRREQESKYIEELAELISANLSDIDNFNVKPDKCAILK
ETVRQIRQIKEQGKSSFNDDDVQKADVSSTGQGVIDKDSLGPLLLQALDGFLFVVNCDGNIVFVSENVTQYLQY
KQEELVNTSVYNILHEEDRDEFLKNLPKTTVNGVPWSSETRRQKSHTFNCRMLVKTSRDPLEDVGGNPELRHRY
EMMQCFALSQPRAMMEEGEDLQSCMICVARRITVVERSFSSSPESFITRHDLLGNVLNIDNNSLRASMRPGCED
LVRRCIQRLSYQSEGQQWSNKRHFREAYLQGQAESPLYRFSLADGTIVTAQTKSKLYRSPQTNEAQGFVSTHFL
QREQNGYRPSQNPMVYGMRVPTAGGTNSVAAMNMPPGQGIRNYCINDPSAMGQMAGMRYGGPGVSGGMGPMNHG
SGMQASPYQSNNYGLSMSSPPHGSPGLNSAQQNLMVSPRSRGSPKMNASQFSPAAGIHSPMGPTSSTGSNTFCN
SSLNALQAISEGVGTSLLSTLSSPGQKMENSPNMNLPQQSKISSQDCKSPSGLFGEHNHLENSVCRPNSRELHG
DQKDDKETNVEGTDNQRGASESKGHKKLLQLLTSPSEDRGHSSSSSALDSNSKEPFTSVTSPSGVSSTSVPGGV

TSTSNIHTTSSSLQEKHKILHKLLQNGNSPAEVAKITAEATGKDTLHESPSTATSGEVIVKQEQMSPKNKECRA
LLKYLLDKDASKDHSLKDIKPKIEGLDNKIGSCCSSTIAASTHQEKEEMKIKTEPSDELSGDLDNIEAILGDLA
SSEFYPETITGNVNDLISKQPLFQESTSFGMRSPQTVQSSKLPFNRAMSLDSQVTNVGSGPPVRSSTPFTAMSK
QDMVGNPRMMGSQETFPGSMDAPNRNMGIPQRPTMASGDWGLQNSNAKSADSAGPGSIIRPDYNQSMPRQMMSN
SMPGIPARSNSLPSGRPMMVDMAMGGNTYGQQGPPSHSSSWPDRMLHMEQGPRAIQNRQPFRGSLDDLLCPPST
AEGQSDERALLDQLDSLLSNTDVTGLEEIDRALGIPDLVSQRQSLEPHSEPFQGQEPVLLMEQKPPIYSQPYPA
QGAALQGSYTAMQGQQTSFTTMVNPMGQQGNFPMQGMHPRPSLIRPRMTNAPKHLRMQLQQRLQGQQFLNQSRQ
ALGIKLDGPTGGNPGVRPGMQTPMTPQLQQNFINGQMMVPRSREMILNQQLRHQRIMMLMQQQQQQSQGFSPPP
NVTAPAGMDSPMGGPPMSQAPPQQFPYPPNYGLSQQPDPAFSRAPSPSNPMMPSRMAPSQNPVMQHPQTTPMYQ
SPDMKGWPSASMTRTSSFPQQQQFASQGNPGTYNTMMHMNGSGGHMGQIPGQMGMNPVPMPRMPMGPDPKYC

>294Latimeria chalumnae|H3ADQ3|Sarcopterygii|SRC3
MSGLGENSLDSVTIETRKRKPSCDTQGGSLNCSGEKRRREQESKYIEELAELISANLSDIDNFNVKPDKCAILK
ETVRQIRQIKEQGKSSFNDDDVQKADVSSTGQGVIDKDSLGPLLLQALDGFLFVVNCDGNIVFVSENVTQYLQY
KQEELVNTSVYNILHEEDRDEFLKNLPKTTVNGVPWSSETRRQKSHTFNCRMLVKTSRDPLEDVGGNPELRHRY
EMMQCFALSQPRAMMEEGEDLQSCMICVARRITVVERSFSSSPESFITRHDLLGNVLNIDNNSLRASMRPGCED
LVRRCIQRLSYQSEGQQWSNKRHFREAYLQGQAESPLYRFSLADGTIVTAQTKSKLYRSPQTNEAQGFVSTHFL
QREQNGYRPSQNPMVYGMRVPTAGGTNSVAAMNMPPGQGIRNYCINDPSAMGQMAGMRYGGPGVSGGMGPMNHG
SGMQASPYQSNNYGLSMSSPPHGSPGLNSAQQNLMVSPRSRGSPKMNASQFSPAAGIHSPMGPTSSTGSNTFCN
SSLNALQAISEGVGTSLLSTLSSPGQKMENSPNMNLPQQSKISSQDCKSPSGLFGEHNHLENSVCRPNSRELHG
DQKDDKETNVEGTDNQRGASESKGHKKLLQLLTSPSEDRGHSSSSSALDSNSKEPFTSVTSPSGVSSTSVPGGV
TSTSNIHTTSSSLQEKHKILHKLLQNGNSPAEVAKITAEATGKDTLHESPSTATSGEVIVKQEQMSPKNKECRA
LLKYLLDKDASKDHSLKDIKPKIEGLDNKIGSCCSSTIAASTHQEKEEMKIKTEPSDELSGDLDNIEAILGDLA
SSEFYPETITGNVNDLISKQPLFQESTSFGMRSPQTVQSSKLPFNRAMSLDSQVTNVGSGPPVRSSTPFTAMSK
QDMVGNPRMMGSQETFPGSMDAPNRNMGIPQRPTMASGDWGLQNSNAKSADSAGPGSIIRPDYNQSMPRQMMSN
SMPGIPARSNSLPSGRPMMVDMVLPCISKYFLKLDYSEIIIAMGGNTYGQQGPPSHSSSWPDRMLHMEQGPRAI
QNRNRQPFRGSLDDLLCPPSTAEGQSDERALLDQLDSLLSNTDVTGLEEIDRALGIPDLVSQRQSLEPHSEPFQ
GQEPVLLMEQKPPIYSQPYPAQGAALQGSYTAMQGQQTSFTTMVNPMGQQGNFPMQGMHPRPSLIRPRMTNAPK
HLRMQLQQRLQGQQFLNQSRQALGIKLDGPTGGNPGVRPGMQTPMTPQQNFINGQMMVPRSREMILNQQLRHQR
IMMLMQQQQQQSQGFSPPPNVTAPAGMDSPMGGPPMSQAPPQQFPYPPNYGLSQQPDPAFSRAPSPSNPMMPSR
MAPSQNPVMQHPQTTPMYQSPDMKGWPSASMTRTSSFPQQQQFASQGNPGTYNTMMHMNGSGGHMGQIPGQMGM
NPVPMPRMPMGPDPKFF
>295Tetraodon nigroviridis|H3CPC4|Sarcopterygii|SRC3
AVKMSGIGDNSLEPLCSDRKRKLSTCDTPGLGCDKRRREQESKYIEELAELISANLSNIDSFNVKPDKCAILKE
TVRQIRQIKEQGKSACSDDDVQKADVSSTGQGVIDKDHLGPLLLQALDGFLFVVNREGSIVFVSDNVTQYLQYK
QEELINTSVYNIIHDEDREEFHKNLPKSNVVSNGAWGGETPRQKSHTFNCRMLVNTVHGQNHGLTEERPPGGQR
YETMQCFALTQPRAMMEEGEDLHSCMICVARRIIVVERTERFSTRHELSGKLVEIEQQASLHTTMRPGWEDLVR
RCMQMFLHRTEGQSWSYKRHYQEAFHNGHAETPLYRFSLSDGTPVTAQTRTDLCRNPSNNEPHSFLSTHFLQRD
QNNYRGNQGGAMRPQNMAVNNPNQQMSVGGAMGMNRGYNMADQGNVSQRAMPSYTGGNRMNPMNPMQQMNPMGG
PVAGNYGMGMTSPTQASPAMNAPPHNVLSSPRVRGSPKMGASPFSPLSGLNSPMSSSHPGNAGGGSTFSSSSLN
ALQAISEGVGNPMPSPLTSPPPQRPDSSPSINSTNVPTGPCKLPTYADSKSPGSSLGGGAEHPTQQHLHTPTGE
VPPEKPDSQASREVGVSGGESSRRVPDSKSHKKLLQLLTSPTDELVPPNHPPTSGPSSTPEAKDGPPTVTSPSP
STGVSSSTGGNHSTVSSSGGAGQSSSQSLQEKHKILHKLLQNGNTPDEVARITAEATGKCALDSAAQEPAAVGT
RGTELKQEQLSPKKEKPNALLHYLLNKDDSKEGSDIKPKLDELEARGVQAAGVTSSDPHCINGKVKMEPPDEAE
TLETILGVSRSNSAFYPEPDSRTGKEVGHKPDSLHGVEFKSPFPYLIHISSHHLSVLRAAYQRALSLDSKPMGG
EGPAAAGGLAGRRNVACPTLVKQENVEAPIRFGSVPNGFPGGGGGMGRGMAVPQQRPPVSGPGDWGMTRPASTP
VVRPGHPGMGRANPMAGPMISRSNSLPGNTRSMLQQKLMDMAANETSMSRFGGPGPPPQSPSWQSSPLGVDGPQ
RNTSSRDQFGNPLDELLGPLTSSEGQSDERALLDQLDSLLNTADVGALQEIDRALGIPEIVGQQGHAQCPSAET
FPGQDTPLSMDQKPMYNQGYPGPPGPPAVNMQPGYGGSGMQGQPPGGFSPMMNQMGQTGSFAGMAGMSSPNANM
MRPRMMTATKPLRLQLQQRLQGQQFMNQTRQGVKMEHNPGGNPAMHPGVQPGMGAQPGFLNPQMVAQRNREMMT
ISQMRRQRMMMLMQQQGQGAAGGFNPPPNVTAPGGMDGPVGAPAMNQTGQQGFNYGANYGMSQQGEPPFMAPGS
SPPGNMMQGRMGGPQNSMMSGMQATPQGGSVYPSGDMKGWPQGNMPRSSSYSQAQFPQQGNQNQFGNMMMNNSR
QRPGQMGMNPMGMGRMPMGPDQKYC
>296Latimeria chalumnae|M3XL66|Sarcopterygii|SRC3

MSGLGENSLDSVTIETRKRKPSCDTQGGSLNCSGEKRRREQESKYIEELAELISANLSDIDNFNVKPDKCAILK
ETVRQIRQIKEQGKSSFNDDDVQKADVSSTGQGVIDKDSLGPLLLQALDGFLFVVNCDGNIVFVSENVTQYLQY
KQEELVNTSVYNILHEEDRDEFLKNLPKTTVNGVPWSSETRRQKSHTFNCRMLVKTSRDPLEDVGGNPELRHRY
EMMQCFALSQPRAMMEEGEDLQSCMICVARRITVVERSFSSSPESFITRHDLLGNVLNIDNNSLRASMRPGCED
LVRRCIQRLSYQSEGQQWSNKRHFREAYLQGQAESPLYRFSLADGTIVTAQTKSKLYRSPQTNEAQGFVSTHFL
QREQNGYRPSQNPMVYGMRVPTAGGTNSVAAMNMPPGQGIRNYCINDPSAMGQMAGMRYGGPGVSGGMGPMNHG
SGMQASPYQSNNYGLSMSSPPHGSPGLNSAQQNLMVSPRSRGSPKMNASQFSPAAGIHSPMGPTSSTGSNTFCN
SSLNALQAISEGVGTSLLSTLSSPGQKMENSPNMNLPQQSKISSQDCKSPSGLFGEHNHLENSVCRPNSRELHG
DQKDDKETNVEGTDNQRGASESKGHKKLLQLLTSPSEDRGHSSSSSALDSNSKEPFTSVTSPSGVSSTSVPGGV
TSTSNIHTTSSSLQEKHKILHKLLQNGNSPAEVAKITAEATGKDTLHESPSTATSGEVIVKQEQMSPKNKECRA
LLKYLLDKDASKDHSLKDIKPKIEGLDNKIGSCCSSTIAASTHQEKEEMKIKTEPSDELSGDLDNIEAILGDLA
SSEFYPETITGNVNDLISKQPLFQESTSFGMRSPQTVQSSKLPFNRAMSLDSQVTNVGSGPPVRSSTPFTAMSK
QDMVGNPRMMGSQETFPGSMDAPNRNMGIPQRPTMASGDWGLQNSNAKSADSAGPGSIIRPDYNQSMPRQMMSN
SMPGIPARSNSLPSGRPMMVDMAMGGNTYGQQGPPSHSSSWPDRMLHMEQGPRAIQNRQPFRGSLDDLLCPPST
AEGQSDERALLDQLDSLLSNTDVTGLEEIDRALGIPDLVSQRQSLEPHSEPFQGQEPVLLMEQKPPIYSQPYPA
QGAALQGSYTAMQGQQTSFTTMVNPMGQQGNFPMQGMHPRPSLIRPRMTNAPKHLRMQLQQRLQGQQFLNQSRQ
ALGIKLDGPTGGNPGVRPGMQTPMTPQLQQNFINGQMMVPRSREMILNQQLRHQRIMMLMQQQQQQSQGFSPPP
NVTAPAGMDSPMGGPPMSQAPPQQFPYPPNYGLSQQPDPAFSRAPSPSNPMMPSRMAPSQNPVMQHPQTTPMYQ
SPDMKGWPSASMTRTSFPQQQQFASQGNPGTYNTMMHMNGSGGHMGQIPGQMGMNPVPMPRMPMGPDPKYC
>297Lepisosteus oculatus|W5M8Q6|Actinopteri|SRC3
VFKMSGLGDNSLDPLCSDRKRKLSTCDTPGLGCDKRRREQESKYIEELAELISANLSDIDNFNVKPDKCAILKE
TVRQIRQIKEQGKASSNDDDVQKADVSSTGQGVIDKDHLGPLLLQALDGFLFVVNRDGSIVFVSDNVTQYLQFK
QEELINTSVYNILHEEDREEFHKNLPKSNRLNGVSWATEAPRQKSHTFNCRMLMKYGHSPPEEGAGSPRYETMQ
CFALTQPKAMMEEGDDLQSCMICVARRITAVERTESFITKHELSGKLIQIDHSSLRSTMRPGWEDLLRRCIQMF
MHHSEGQPWSHKRHYQEAFMQGHAETPLYRFSLSDGTPVTAQTKSKLYRNPMTNEPQGFISTHLLQREQNGYRA
NQGPMIQGMRPMGNSTPNASMNMPPGPGMGMGGNRGFSMNESGHMGQMGGSMYGATNRMMQMNPMSQMSQMGQQ
QPPYQGSGYGLGMNSPSQGSPGMNVPQQNLMVSPRTRGSPKMVPSQFSPGGMHSPMGPGSAGGAGGGGNSNFSS
SSLNALQAISEGVGNPMPSSLSSPAHKPDSSPSINSSAGRVDSPSPAGMYVPGGDHHHHLHHHHHTPTESATDR
PDSQEGSEVGTGGAEPQRRLSDSKGNKKLLQLLTSPTDDLGMVAGGVPPTGASTPSTLEPKEPAGCVTSPSSTG
VSSSSSSSSSAAQPPGGVSSSSSSAHHAASLQEKHKILHKLLQNGNSPDEVAKITAEATGKETSSHEAGVADLG
TIGTAGTGGGGVGGSGVPDIKQEQPSPKKTHALLHYLLNNDPKEPADIKPKLEELEGKTQQNACSSSGLMPSTP
ENGENKIKTEQPDELHDTLETILGGFRNSSSSFYQESGVGAGSDVANKQPACPDDAMHGLRSPAGLRSPELGPR
GPFQRAVSVDGKPPVGANSLGRRSAPCPMLVKQESMDNQRMMGGPENFPGNMGMVNRGLGVPQRSPMGGSGEWG
IQRSNASPVGTSSHPMVRPGMDYSSKPMMGGPMVGRSNSVPGNRSMLQQQLMDMGPNDINMGMTPFNQQGPPNQ
SPSWPENVMPIEHGPQNSQSRRQFANPLDDLLVPPATSEGQSDERALLDQLDSLLNNTDVMALEEIDRALGIPD
LVSQGQGVDPQSEPFPGQDPALVMDQKPPMYSQGYPGPPGMAMQPAYSSPMQGQPPGFNPMMNQMSQQGNFPMA
GMHPRPNMIRPRMMNALKPLRQQLVQRLQGQQFMNQSRQGLGMKMDNTTAGNPGLRPALQAPMGGQPGFLNAQM
MAQRNREMLNHQLRRQRMMMLMQQQQQAAAGGFSPPPNVTAPAGMDTPMGGPPMTQPGPQQFSYAANYGINQQG
DPSFVRAAGSPPNSIMSARLGPPQNPMMQQHPQGGPMYPTADMKSWPQGGMPRSSSYPQQQFPQQGNPGSYGNM
MMNGSLPVNGNGGHMGQMPGQMNMNPMAMGRMPMGPEQKYC
>298Lepisosteus oculatus|W5M8R6|Actinopteri|SRC3
MSGLGDNSLDPLCSDRKRKLSTCDTPGLGCDKRRREQESKYIEELAELISANLSDIDNFNVKPDKCAILKETVR
QIRQIKEQGKASSNDDDVQKADVSSTGQGVIDKDHLGPLLLQALDGFLFVVNRDGSIVFVSDNVTQYLQFKQEE
LINTSVYNILHEEDREEFHKNLPKSNMNGVSWATEAPRQKSHTFNCRMLMKYGHSPPEEGAGSPRYETMQCFAL
TQPKAMMEEGDDLQSCMICVARRITAVERTESFITKHELSGKLIQIDHSSLRSTMRPGWEDLLRRCIQMFMHHS
EGQPWSHKRHYQEAFMQGHAETPLYRFSLSDGTPVTAQTKSKLYRNPMTNEPQGFISTHLLQREQNGYRANQGP
MIQGMRPMGNSTPNASMNMPPGPGMGMGGNRGFSMNESGHMGQMGGSMYGATNRMMQMNPMSQMSQMGQMSQMN
HPGPGMQQQPPYQGSGYGLGMNSPSQGSPGMNVPQQNLMVSPRTRGSPKMVPSQFSPGGMHSPMGPGSAGGAGG
GGNSNFSSSSLNALQAISEGVGNPMPSSLSSPAHKPDSSPSINSSQQQQQQQQQQQQQQQQQQQQQQQQQNQQC
KAGRVDSPSPAGMYVPGGDHHHHLHHHHHTPTESATDRPDSQASLRVTKEGSEVGTGGAEPQRRLSDSKGNKKL
LQLLTSPTDDLGMVAGGVPPTGASTPSTLEPKEPAGCVTSPSSTGVSSSSSSSSSAAQPPGGVSSSSSSAHHAA
SLQEKHKILHKLLQNGNSPDEVAKITAEATGKETSSHEAGVADLGTIGTAGTGGGGVGGSGVPDIKQEQPSPKK
THALLHYLLNNDPKEPADIKPKLEELEGKTQQNACSSSGLMPSTPENGENKIKTEQPDELHDTLETILGGFRNS
SSSFYQESGVGAGSDVANKQPACPDDAMHGLRSPAGLRSPELGPRGPFQRAVSVDGKPPVGANSLGRRSAPCPM

LVKQESMDNQRMMGGPENFPGNMGMVNRGLGVPQRSPMGGSGEWGIQRSNASPVGTSSHPMVRPGMDYSSKPMM
GGPMVGRSNSVPGNRSMLQQQLMDMGPNDINMGMTPFNQQGPPNQSPSWPENVMPIEHGPQNSQSRRQFANPLD
DLLVPPATSEGQSDERALLDQLDSLLNNTDVMALEEIDRALGIPDLVSQGQGVDPQSEPFPGQDPALVMDQKPP
MYSQGYPGPPGMAMQPAYSSPMQGQPPGFNPMMNQMSQQGNFPMAGMHPRPNMIRPRMMNALKPLRQQLVQRLQ
GQQFMNQSRQGLGMKMDNTTAGNPGLRPALQAPMGGQPGFLNAQMMAQRNREMLNHQLRRQRMMMLMQQQQQAA
AGGFSPPPNVTAPAGMDTPMGGPPMTQPGPQQFSYAANYGINQQGDPSFVRAAGSPPNSIMSARLGPPQNPMMQ
QHPQGGPMYPTADMKSWPQGGMPRSSSYPQQQFPQQGNPGSYGNMMMNGSLPVNGNGGHMGQMPGQMNMNPMAM
GRMPMGPEQKYC
